# Functional Constraints on Insect Immune System Components Govern Their Evolutionary Trajectories

**DOI:** 10.1093/molbev/msab352

**Published:** 2021-12-10

**Authors:** Livio Ruzzante, Romain Feron, Maarten J M F Reijnders, Antonin Thiébaut, Robert M Waterhouse

**Affiliations:** Department of Ecology and Evolution, Swiss Institute of Bioinformatics, University of Lausanne, Lausanne, Switzerland

**Keywords:** *Anopheles* mosquito, evolutionary profiling, gene expression, gene families, innate immunity

## Abstract

Roles of constraints in shaping evolutionary outcomes are often considered in the contexts of developmental biology and population genetics, in terms of capacities to generate new variants and how selection limits or promotes consequent phenotypic changes. Comparative genomics also recognizes the role of constraints, in terms of shaping evolution of gene and genome architectures, sequence evolutionary rates, and gene gains or losses, as well as on molecular phenotypes. Characterizing patterns of genomic change where putative functions and interactions of system components are relatively well described offers opportunities to explore whether genes with similar roles exhibit similar evolutionary trajectories. Using insect immunity as our test case system, we hypothesize that characterizing gene evolutionary histories can define distinct dynamics associated with different functional roles. We develop metrics that quantify gene evolutionary histories, employ these to characterize evolutionary features of immune gene repertoires, and explore relationships between gene family evolutionary profiles and their roles in immunity to understand how different constraints may relate to distinct dynamics. We identified three main axes of evolutionary trajectories characterized by gene duplication and synteny, maintenance/stability and sequence conservation, and loss and sequence divergence, highlighting similar and contrasting patterns across these axes amongst subsets of immune genes. Our results suggest that where and how genes participate in immune responses limit the range of possible evolutionary scenarios they exhibit. The test case study system of insect immunity highlights the potential of applying comparative genomics approaches to characterize how functional constraints on different components of biological systems govern their evolutionary trajectories.

## Introduction

The concept of constraints in evolutionary biology encompasses a diverse array of interpretations and terminologies shaped by the approaches of different research fields (Antonovics and van Tienderen 1991). In general terms, constraints can be described as factors that limit or direct the process of natural selection leading to outcomes representing only a fraction of all theoretically possible scenarios. Constraints may impact the capacity to generate new variants as well as how selection either limits or promotes consequent phenotypic change, often considered in developmental biology (Richardson and Chipman 2003) and population genetics (Hoffmann 2013) contexts. Comparative genomics also recognizes the role of constraints, in shaping the evolution of gene and genome architectures, sequence evolutionary rates, and gene gains and losses, as well as on the molecular phenotypes governed by their functional products (Koonin and Wolf 2010). For example, protein sequence evolution is constrained by requirements for maintaining proper protein structure and function, including during folding and interactions with other macromolecules (Worth et al. 2009). Functional constraints also impact the evolution of gene families, for example, families of paralogs with or without essential genes exhibit dramatically different evolutionary regimes in terms of sequence divergence and duplication rates (Shakhnovich and Koonin 2006). These likely influence observed trends across the gene duplication spectrum that show a dichotomy of constrained single-copy control versus a multi-copy license for greatly relaxed copy-number restrictions (Waterhouse et al. 2011). Integrative analyses of evolutionary and functional constraints point to emergent properties such as a gene family’s “importance” or “status” characterized by low sequence divergence and propensity for gene loss with high expression levels, protein interactions, and essentiality; or a family’s “adaptability” manifested by high duplication levels, many genetic interaction partners, and a tendency of genes to be nonessential; or a family’s “reactivity” with high gain/loss and expression levels but low sequence divergence, a paucity of essential genes, and few physical or genetic interactions (Wolf et al. 2006). If such constraints limit the realm of possibilities in terms of allowed gene evolutionary trajectories then recurring patterns should be observable for genes evolving under similar constraints. Characterizing these patterns in the context of a relatively well-studied system, where putative functional roles and interactions of member genes are well described, offers an opportunity to explore whether genes with similar or analogous functions exhibit similar evolutionary trajectories, possibly governed by common constraints.

The insect innate immune system is relatively well characterized with respect to the functional roles and evolutionary histories of key implicated pathways and component gene families. It confers remarkable resilience to encountered pathogens through the activation of powerful responses to neutralize and clear infections (Rolff and Reynolds 2009; Ligoxygakis 2017). The immune system comprises both humoral and cellular responses with components dedicated to recognizing signs of infection, signaling cascades to activate primary defenses and induce transcriptional responses, modulators that control the intensity and direction of responses, and effector proteins and biomolecules for pathogen killing. Many of the genes and their protein products implicated in these complex processes were first identified in the fruit fly, *Drosophila melanogaster* (Lemaitre and Hoffmann 2007; Imler 2014). Classical receptor proteins that recognize pathogen-associated molecular patterns include peptidoglycan recognition proteins (PGRPs) (Wang et al. 2019) and β-1,3-glucan recognition or gram-negative bacteria-binding proteins (GNBPs) (Rao et al. 2018). Pathogen recognition may then trigger immune signaling through the Toll (Valanne et al. 2011), Imd (Myllymäki et al. 2014), or the JAnus kinase protein (JAK)/signal transducer and activator of transcription (STAT) (Myllymäki and Rämet 2014) pathways. Their activation leads to the translocation of transcription factors to the nucleus where the expression of effector genes such as those encoding antimicrobial peptides (AMPs) (Lazzaro et al. 2020) is upregulated. Defense responses are mediated by various cells and tissues including hemocytes, the fat body, and the midgut, and pathogen killing can occur via processes such as melanization, phagocytosis, lysis, autophagy, and apoptosis (Hillyer 2016; King 2020), with RNA interference (RNAi) facilitating major antiviral defenses (Mussabekova et al. 2017). These complex interactions collectively offer insects protection from a vast array of viruses, bacteria, fungi, protozoa, and nematodes.

Sequencing the *D. melanogaster* and *Anopheles gambiae* genomes provided the first opportunity for comparative genomic analysis of immune-related genes in insects (Christophides et al. 2002). Advances in genome sequencing technologies have facilitated an increasingly dense sampling of species to explore insect gene repertoires and perform cross-species comparisons to trace gene evolutionary histories (Waterhouse 2015). This has allowed comparisons beyond Diptera to include Hymenoptera (Evans et al. 2006; Brucker et al. 2012; Barribeau et al. 2015), Coleoptera (Zou et al. 2007), Lepidoptera (Tanaka et al. 2008), and Hemiptera (Gerardo et al. 2010), as well as expanded sampling of flies and mosquitoes (Sackton et al. 2007; Waterhouse et al. 2007; Bartholomay et al. 2010; Sackton et al. 2017). These comparative studies generally focused on the canonical immune-related gene repertoire, comprising genes that have been directly implicated in immune responses through experimental research, or indirectly linked to immunity through homology to known immune proteins (Bartholomay and Michel 2018; Waterhouse et al. 2020). Emerging patterns pointed to distinct evolutionary dynamics that characterize different immune phases: (1) gene and domain gains or losses (turnover) can create diversity in recognition modules; (2) core signaling pathway members are almost always maintained as single-copy orthologs often with elevated levels of sequence divergence; (3) modulators appear to form lineage-restricted units with members picked from large families often with high gene turnover rates; and (4) effectors like AMPs show dynamic gains and losses or are lineage-restricted whereas oxidative defense effectors are widespread with low levels of sequence divergence. These observations provide specific examples and strong expectations of types of genes with similar functions that exhibit similar evolutionary trajectories, within the established framework of insect innate immunity that classifies genes and families into broad functional categories of recognition, signal transduction, modulation, and effector components.

These trends are based on observations from cross-species comparisons of insect immune gene repertoires. Here we hypothesize that comprehensive quantitative multispecies and multifeature characterization of gene family evolutionary histories can define distinct dynamics associated with different functional roles in immune responses. Such detailed evolutionary profiling can then be used to address the question of whether gene families involved in common immune functional categories, modules, or processes exhibit similar evolutionary trajectories possibly driven by shared constraints. We take advantage of genomic resources available for 22 mosquito species (Holt et al. 2002; Nene et al. 2007; Arensburger et al. 2010; Lawniczak et al. 2010; Marinotti et al. 2013; Jiang et al. 2014; Chen et al. 2015; Neafsey et al. 2015; Matthews et al. 2018; Ruzzante et al. 2019) and 46 other insects to (1) develop a suite of metrics that quantify gene and gene family evolutionary histories, (2) employ these metrics to characterize the evolutionary features of mosquito and fly immune gene repertoires, and (3) explore the relationships between gene family evolutionary profiles and their functional roles in immunity to understand how different constraints may relate to distinct dynamics. The resolution afforded by multispecies comparative analyses and our suite of gene sequence and copy-number evolutionary metrics reveals the evolutionary features that most clearly distinguish each family, and highlights similar and contrasting patterns across all immune gene families. Complementing knowledge-based functional categorizations with gene coexpression analyses identifies immune families that function in concert, revealing evolutionary-functional correspondences where most prominently, families involved in mosquito complement system responses show both high evolutionary similarities and high expression similarities.

## Results and Discussion

### A Suite of Metrics to Quantify Gene and Gene Family Evolutionary Histories

The developed set of evolutionary feature metrics is designed to capture a broad spectrum of gene evolutionary dynamics including taxonomic spread, copy-number changes, protein- and DNA-level sequence divergence, conservation, and constraint, as well as genomic organization, and population-level sequence variation (table 1). The 18 metrics are computed using gene orthology delineated across 43 insect species (21 mosquitoes, 15 other dipterans, and 2 outgroup representatives each for Lepidoptera, Coleoptera, Hymenoptera, and Exopterygota), sets of whole genome alignments with 22 mosquitoes or with 36 *Drosophila*, or polymorphism data from *An. gambiae* (see Materials and Methods). Orthologous groups (OGs) comprised all genes descended from a single gene in the last common ancestor of the set of the extant species under consideration. As such they form the principal unit for which the suite of metrics is computed. OG compositions are used directly to quantify features such as universality (UNI; the proportion of species present in an OG) or duplicability (DUP; the proportion of species that have multicopy orthologs). They are used as inputs for gene copy-number turnover analysis to quantify gain (expansion) and loss (contraction) events. Their aligned sequences are used to compute protein- and DNA-level divergence metrics per OG. Nucleotide-level measurements from whole genome alignment or population variation data are computed over each gene’s coding-sequence length and averaged over multicopy orthologs in an OG. Compositions of families range from just a single OG for prophenoloxidases (PPO, 9 *An. gambiae* genes, 3 *D. melanogaster* genes), to 23 OGs with 28 *An. gambiae* genes for small regulatory RNA pathway members (SRRP) or 29 OGs with 37 *D. melanogaster* genes for C-type lectins (CTL) (table 2). The suite of metrics represents a comprehensive quantitative framework to enable detailed evolutionary feature profiling analyses, here applied to 298 OGs containing 420 *An. gambiae* immune-related genes and 276 OGs with 354 *D. melanogaster* immunity genes.

**Table 1.** Evolutionary Feature Metric Descriptions.

Evolutionary feature	Acronym	Description	Data source
Taxonomic age	AGE	Age of the last common ancestor of species in an OG, in terms of millions of years since divergence, computed from the ultrametric species phylogeny	43-insect orthology
Universality	UNI	The proportion of the total species present in an OG (all species, UNI = 1)	43-insect orthology
Duplicability	DUP	The proportion of species present in an OG that have multicopy orthologs	43-insect orthology
Average copy number	ACN	The average (mean) ortholog copy number across all species present in an OG	43-insect orthology
Copy number variation	CNV	The standard deviation of ortholog counts per species present in an OG divided by the ACN	43-insect orthology
Expansions	EXP	CAFE quantified proportions of gene gain nodes for an OG	43-insect orthology
Contractions	CON	CAFE quantified proportions of gene loss nodes for an OG	43-insect orthology
Stability	STA	CAFE quantified proportions of no copy-number change nodes for an OG	43-insect orthology
Synteny	SYN	The proportion of orthologs in an OG that maintains their orthologous neighbors in the genomes of the other species	43-insect orthology
Evolutionary rate	EVR	The average rate of protein sequence divergence normalized by the distance (% identity) between each pair of species as computed by OrthoDB	43-insect orthology
PAML’s dS	PDS	The number of synonymous substitutions per synonymous site as computed by PAML	19-*Anopheles* orthology
PAML’s dN	PDN	The number of nonsynonymous substitutions per nonsynonymous site as computed by PAML	19-*Anopheles* orthology
PAML’s dN/dS	SEL	The nonsynonymous to synonymous substitution ratio (dN/dS) as computed by PAML	19-*Anopheles* orthology
Nonsynonymous SNP proportion	NSP	The proportion of all coding-sequence SNPs that were nonsynonymous (averaged over genes per OG)	*An. gambiae* variation
Nonsynonymous SNP density	NSD	The density of nonsynonymous SNPs over a gene’s coding-sequence length (averaged over genes per OG)	*An. gambiae* variation
Synonymous SNP density	SSD	The density of synonymous SNPs over a gene’s coding-sequence length (averaged over genes per OG)	*An. gambiae* variation
Whole genome alignability	WGA	The number of species aligned, per nucleotide from the whole-genome alignment, averaged over coding-sequence length (averaged over genes per OG)	22 mosquitoes
			36 *Drosophila*
PhastCons constraint	PHC	PhastCons quantified constraint scores, per nucleotide from the whole-genome alignment, averaged over coding-sequence length (averaged over genes per PG)	22 mosquitoes
			36 *Drosophila*

Note.—For each evolutionary feature, the metric name, acronym, description, and source data are presented (see Materials and Methods for details).

**Table 2.** The *Anopheles gambiae* and *Drosophila melanogaster* Immunity Gene Catalogs.

Acronym	Summary description	*An. gambiae*		*D. melanogaster*	
		Genes	OGs	Genes	OGs
GALE	Galectins bind specifically to β-galactoside sugars and can function as pattern recognition receptors in innate immunity	9	6	6	5
GNBP	Gram-negative binding proteins (or β-1,3-glucan-binding proteins) are a family of carbohydrate-binding pattern recognition receptors	7	3	3	3
PGRP	Peptidoglycan recognition proteins are pattern recognition receptors capable of recognizing the peptidoglycan from bacterial cell walls	7	5	12	6
SCRA	Scavenger receptors are made up of different classes that function as pattern recognition receptors for a broad range of ligands including from pathogens	5	5	5	4
SCRB		13	10	14	9
CTL	C-type lectins are carbohydrate-binding proteins with roles in pathogen opsonization, encapsulation, and melanization, as well as immune signaling cascades	25	20	37	29
FREP	Fibrinogen-related proteins (also known as FBNs) are a family of pattern recognition receptors with homology to the C terminus of the fibrinogen β and γ chains	38	15	13	6
LRIM	Leucine-rich repeat immune proteins are mosquito immune factors that activate complement-like defense responses against pathogens	24	20	0	0
ML	MD-2-like proteins, also known as Niemann-Pick Type C-2 proteins, possess myeloid-differentiation-2-related lipid-recognition domains involved in recognizing lipopolysaccharide	16	7	8	5
NIMROD	Nimrods have been shown to bind bacteria leading to their phagocytosis by hemocytes, they contain epidermal growth factor-like domains	3	3	12	8
TEP	Thioester-containing proteins are related to vertebrate complement factors and α2-macroglobulin protease inhibitors, their activation through proteolytic cleavage leads to phagocytosis or killing of pathogens	10	5	5	5
IMDSIG	The immune deficiency pathway is characterized by peptidoglycan recognition protein receptors, intracellular signal transducers (IMDSIG) and modulators (IMDMOD), and the NF-κB transcription factor Relish	9	9	10	10
IMDMOD		6	6	6	6
JASTSIG	The JAK and the STAT are two core components of the JAK/STAT pathway, with signal transducers (JASTSIG) and modulators (JASTMOD) involved in cellular responses to stress or injury	3	3	6	6
JASTMOD		3	3	4	4
TOLLSIG	The intracellular components of the Toll pathway are homologous to the toll-like receptor innate immune pathway in mammals, with signal transducers (TOLLSIG) and modulators (TOLLMOD) culminating in activation of the NF-κB transcription factors Dorsal	5	5	6	6
TOLLMOD		8	8	8	8
CASP	Caspases are cysteine-aspartic proteases involved in immune signaling cascades and apoptosis	15	6	7	5
CLIPA	Subfamilies of CLIP-domain serine proteases are defined by patterns of cysteine residues, several CLIPs have roles as activators or modulators of immune signaling cascades	20	13	12	10
CLIPB		27	20	15	13
CLIPC		8	6	7	7
CLIPD		9	8	10	10
CLIPE		9	7	3	3
IAP	Inhibitors of apoptosis are important in antiviral responses and are involved in regulating immune signaling and suppressing apoptotic cell death	8	5	4	4
SRPN	Serine protease inhibitors, or serpins, modulate many signaling cascades; they act as suicide substrates to inhibit their target proteases	18	16	30	20
AMP	Antimicrobial peptides are the classical effector molecules of innate immunity; they include defensins, cecropins, and attacins that are involved in bacterial killing by disrupting their membranes	9	8	10	5
LYS	Lysozymes are key effector enzymes that hydrolyze peptidoglycans present in the cell walls of many bacteria, causing cell lysis	7	1	17	3
PPO	Prophenoloxidases are key enzymes in the melanization cascade that helps to kill invading pathogens and is important for wound healing	9	1	3	1
GPX	Glutathione, heme, and thioredoxin peroxidases are enzymes involved in the metabolism of reactive oxygen species that are toxic to pathogens	2	2	2	2
HPX		15	10	10	9
TPX		5	5	6	6
SOD	Superoxide dismutases are antioxidant enzymes involved in the metabolism of toxic superoxide into oxygen or hydrogen peroxide	4	4	4	4
APHAG	Autophagy-related genes participate in a form of cell death characterized by the formation of an internal autophagosome where pathogens are degraded	19	19	22	22
SRRP	Small regulatory RNA pathway members are involved in RNA interference and include argonautes, dicers, piwis, and helicases	28	23	22	20
SPZ	Spaetzle-like proteins contain a cysteine knot domain, the cleavage of Spaetzle results in binding of the product to the Toll receptor and subsequent activation of the Toll pathway	5	5	6	6
TOLL	Toll receptors connect extracellular pathogen recognition to intracellular Toll pathway signaling and activation of immune defense responses	12	6	9	6
**Totals:**		**420**	**298**	**354**	**276**

Note.—Brief descriptions of immune gene families or pathway components are presented along with counts of the numbers of genes and OGs for the mosquito and fly catalogs.

### The Evolutionary Feature Landscape of Mosquito Immunity

**Fig. 1.**
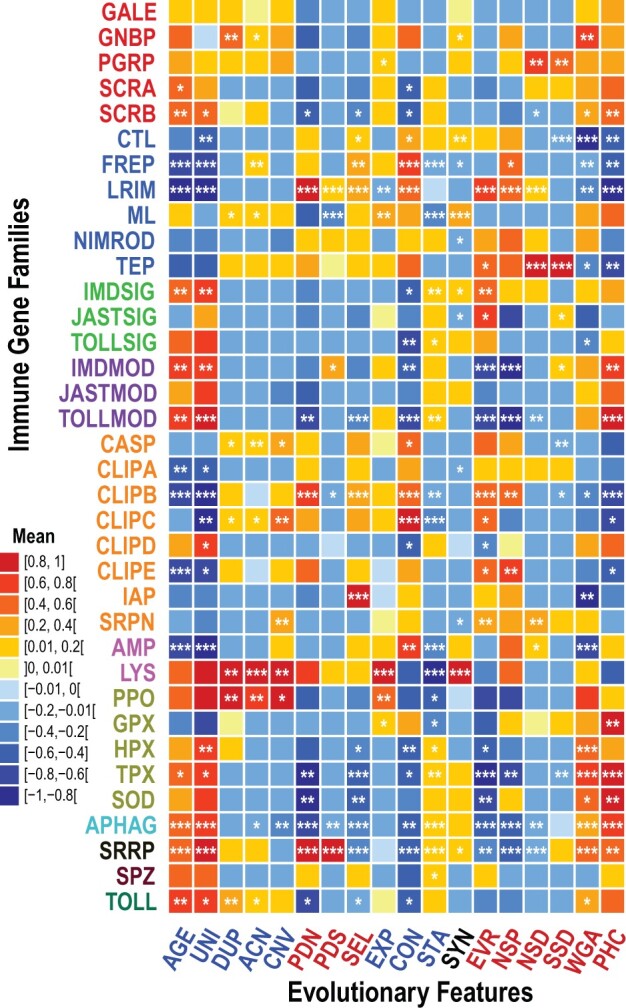
Evolutionary feature profiles of mosquito immune gene families. Evolutionary profiling highlights similar and contrasting patterns across all 36 immune gene families or subfamilies (rows). Deviations from the typical metric values for the suite of 18 evolutionary feature metrics (columns) are computed as the difference between the family mean and the average over all OGs from other immune-related gene families (Δ$\bar{\text{x}}$). For visualization, values of Δ$\bar{\text{x}}$ are scaled by the absolute maximum Δ$\bar{\text{x}}$ per metric, that is, for each metric the distribution is transformed by dividing all values by the absolute maximum Δ$\bar{\text{x}}$. Values therefore range from a minimum of –1 for metrics where the largest deviation is below the mean, that is lower than other families, and the maximum of 1 for metrics where the largest deviation is above the mean, that is higher than other families. The significance of the difference of the distribution of metric values (no scaling) for each family compared with all other families was assessed using the Wilcoxon rank-sum (Mann–Whitney *U*) test and a permutation test (asterisks correspond to the lower *P* value from these two tests; ***P ≤ 0.01, **P ≤ 0.05, *P ≤ 0.1). Feature acronyms are defined in table 1. Family acronyms are defined in table 2 and are colored according to categories defined based on their putative roles in the principal immune phases: classical recognition (red), other recognition (blue), pathway signaling (bright green), pathway modulation (purple), cascade modulation (orange), antimicrobial effectors (pink), effector enzymes (olive green), autophagy (dark cyan), RNAi (black), cytokines (brown), and toll receptors (dark green). See text for definitions of evolutionary feature acronyms: taxonomic spread and copy-number features in blue; sequence-based features in red. Evolutionary feature profiles of mosquito immune gene families with median differences (Δ$\bar{\text{x}}$) are presented in supplementary figure S1, Supplementary Material online.

Profiles built from the 18 quantified evolutionary features successfully delineate key similarities and differences amongst the catalog of 36 canonical mosquito immune-related gene families and subfamilies (fig. 1). The evolutionary feature profiles for all families are visualized by averaging the metrics over all OGs with genes belonging to each family. Contrasting the profile of a given family against the profiles of all other immune-related families reveals the evolutionary features that most clearly distinguish each family (fig. 1; supplementary fig. S1, Supplementary Material online). This is clearly illustrated by the leucine-rich repeat immune genes (LRIMs) comprising 24 *An. gambiae* genes from 20 OGs, members of which interact with thioester-containing proteins (TEPs) to activate complement-like responses against pathogens (Povelones et al. 2009; Levashina and Baxter 2018). Their taxonomic age (AGE) and UNI are significantly lower, consistent with there being no detectable LRIM orthologs beyond mosquitoes (Waterhouse et al. 2010). They also exhibit fairly typical low DUP, average copy-number (ACN), and copy-number variation (CNV), reflecting their mostly single-copy ortholog status across mosquitoes. These metrics describe the family as a whole although allowing for differences amongst members, for example, the gene duplications that gave rise to three *APL1/LRIM2* paralogs in one lineage of African *Anopheles* (Mitri et al. 2020). Estimates of nonsynonymous substitutions per nonsynonymous site (PDN) are higher than for other families, and significantly so. They are not as extreme, but still significantly higher than other families, for synonymous substitutions per synonymous site (PDS). Together this produces PDN:PDS ratios (SEL, i.e. dN/dS ratios) that are significantly higher than other families, consistent with positive selection or relaxed constraint as observed in previous genus-wide analyses (Neafsey et al. 2015).

Gene gain/loss estimates for the LRIMs show significantly fewer expansions (EXP) and significantly more contractions (CON), but overall stability (STA) close to the mean, in agreement with the copy-number metrics. Conservation of genomic neighborhood, or synteny (SYN), is slightly lower than average for LRIMs, although they notably show the most extreme significantly elevated protein sequence evolutionary divergence (EVR). Single nucleotide polymorphism (SNP) data also show a significantly elevated proportion of nonsynonymous SNPs (NSP) and significantly above average nonsynonymous SNP density (NSD), with synonymous SNP density (SSD) slightly below the mean. The family as a whole thus appears to reflect the natural diversity and polymorphism observed for some family members (Rottschaefer et al. 2011; Holm et al. 2012). Finally, whole genome alignment data show that LRIMs are significantly less alignable (whole genome alignability [WGA]) and significantly less constrained (per-nucleotide levels of constraint [PHC]) than other immune gene families, reflecting the patterns observed with protein- and DNA-based measures of sequence divergence.

Family profiles highlight the extent to which each family deviates from or matches the typical metric values for each evolutionary feature. GNBPs are characterized by high values for metrics capturing gene duplications (DUP and ACN) with high alignability across mosquito genomes (WGA), consistent with the birth of the B-type GNBP subfamily in the mosquito ancestor (Bartholomay et al. 2010). In contrast, Imd pathway signaling genes (IMDSIGs) are characterized as being relatively ancient (high AGE and UNI) and copy-number stable (low CON and high STA) with nevertheless a high protein sequence evolutionary rate (EVR), in agreement with previously observed evolutionary dynamics of immune signaling pathway members (Waterhouse et al. 2007). The subfamilies of CLIP-domain serine proteases are characteristically young (low AGE and UNI), except for CLIPDs which are older and significantly more taxonomically widespread (UNI), a contrast also reflected by several other evolutionary features. Differences amongst CLIP subfamilies could relate to the roles of catalytic and noncatalytic members in modulatory cascades and their hierarchies (El Moussawi et al. 2019).

The autophagy (APHAG) and SRRPs share many features that are significantly different from the mean: They are ancient (high AGE and UNI), stable (low CON and high STA), and constrained (low SEL, EVR, NSP, NSD with high WGA and PHC). However they differ markedly with respect to estimates of dN and dS with both PDN and PDS being significantly lower for APHAGs and significantly higher for SRRPs. Their overall conservation and stability is consistent with both autophagy and RNAi being ancient cellular processes with roles beyond immunity, although their contrasting levels of substitutions could reflect different structural constraints on protein–protein versus protein–RNA interactions. The SRRPs do show above average DUP and ACN values, but not significantly so, consistent with reported single-copy orthologs of *Argonautes 1* and *2* and duplications of *Piwi/Aubergine* in mosquitoes (Lewis et al. 2016). Indeed previous analyses of SRRPs suggested faster evolution in *Aedes* and *Culex* rather than *Anopheles* mosquitoes (Campbell et al. 2008), so conservative patterns observed here could be driven by the data set consisting mainly of anophelines.

The distributions of computed OG metrics for all of the mosquito immune gene families for each evolutionary feature are presented in supplementary additional file 1, Supplementary Material online together with statistical assessments of the significance of deviations from the typical metric values. The trends and significant differences observed across the suite of quantified features facilitate evolutionary profiling that recovers previous mostly qualitative observations and highlights similar and contrasting patterns across all immune gene families (table 3).

**Table 3.** Characteristic Evolutionary Features of Immune Gene Families and Subfamilies.

Family	Significantly higher	Significantly lower	Interpretation summary
GALE	–	–	No extreme features
GNBP	DUP, ACN, SYN, WGA	–	Duplications, maintained neighborhood, widely alignable
PGRP	EXP, NSD, SSD	–	Duplications, population variation
SCRA	AGE	CON	Ancient, stable copy-number
SCRB	AGE, UNI, WGA, PHC	PDN, SEL, CON, NSD	Ancient, widespread, widely alignable, constrained sequence, constrained substitutions, stable copy-number, population conservation
CTL	SEL, CON, SYN	UNI, SSD, WGA, PHC	Relaxed substitutions, losses, maintained neighborhood, widespread, population conservation, sparsely alignable, relaxed sequence
FREP	ACN, SEL, CON, NSP	AGE, UNI, STA, SYN, WGA, PHC	Duplications, relaxed substitutions, losses, amino acid divergence, young, sparse, unstable copy-number, shuffled neighborhood, sparsely alignable, relaxed sequence
LRIM	PDN, PDS, SEL, CON, EVR, NSP, NSD	AGE, UNI, EXP, WGA, PHC	Relaxed substitutions, losses, amino acid divergence, population variation, young, sparse, stable copy-number, sparsely alignable, relaxed sequence
ML	DUP, ACN, EXP, SYN	PDS, STA	Duplications, maintained neighborhood, constrained substitutions, unstable copy-number
NIMROD	–	SYN	Shuffled neighborhood
TEP	EVR, NSD, SSD	WGA, PHC	Amino acid divergence, population variation, sparsely alignable, relaxed sequence
IMDSIG	AGE, UNI, STA, SYN, EVR	CON	Ancient, widespread, stable copy-number, maintained neighborhood, amino acid divergence
JASTSIG	EVR, SSD	SYN	Amino acid divergence, population variation, shuffled neighborhood
TOLLSIG	STA	CON, WGA	Stable copy-number, sparsely alignable
IMDMOD	AGE, UNI, PDS, SSD, PHC	CON, EVR, NSP	Ancient, widespread, relaxed synonymous substitutions, population variation, constrained sequence, stable copy-number, amino acid conservation
JASTMOD	–	–	No extreme features
TOLLMOD	AGE, UNI, STA, PHC	PDN, SEL, CON, EVR, NSP, NSD	Ancient, widespread, stable copy-number, constrained sequence, relaxed substitutions, amino acid divergence, population variation
CASP	DUP, ACN, CNV, CON	SSD	Duplications, losses, population conservation
CLIPA	–	AGE, UNI, SYN	Young, sparse, shuffled neighborhood
CLIPB	PDN, SEL, CON, EVR, NSP	AGE, UNI, PDS, STA, SSD, WGA, PHC	Relaxed substitutions, losses, amino acid divergence, young, sparse, constrained synonymous substitutions, unstable copy-number, population conservation, sparsely alignable, relaxed sequence
CLIPC	DUP, ACN, CNV, CON, EVR	UNI, STA, PHC	Duplications, losses, amino acid divergence, sparse, unstable copy-number, relaxed sequence
CLIPD	UNI	CON, EVR	Widespread, stable copy-number, amino acid conservation
CLIPE	EVR, NSP	AGE, UNI, PHC	Amino acid divergence, young, sparse, relaxed sequence
IAP	SEL	WGA	Relaxed substitutions, sparsely alignable
SRPN	CNV, EVR, NSD	SYN	Duplications, amino acid divergence, shuffled neighborhood
AMP	CON, NSD	AGE, UNI, STA, WGA	Losses, amino acid divergence, young, sparse, unstable copy-number, sparsely alignable
LYS	DUP, ACN, CNV, EXP, SYN	STA	Duplications, maintained neighborhood, unstable copy-number
PPO	DUP, ACN, CNV, EXP	STA	Duplications, unstable copy-number
GPX	EXP, PHC	STA	Duplications, constrained sequence, unstable copy-number
HPX	UNI, STA, WGA	SEL, CON, EVR	Widespread, stable copy-number, widely alignable, relaxed substitutions, amino acid conservation
TPX	AGE, UNI, STA, WGA, PHC	PDN, SEL, CON, EVR, NSP, SSD	Ancient, widespread, stable copy-number, widely alignable, constrained sequence, constrained substitutions, amino acid conservation, population conservation
SOD	WGA, PHC	PDN, SEL, EVR	Widely alignable, constrained sequence, constrained substitutions, amino acid conservation
APHAG	AGE, UNI, STA, WGA, PHC	ACN, CNV, PDN, PDS, SEL, CON, EVR, NSP, NSD	Ancient, widespread, stable copy-number, widely alignable, constrained sequence, constrained substitutions, amino acid conservation, population conservation
SRRP	AGE, UNI, PDN, PDS, STA, SYN, WGA, PHC	SEL, CON, EVR, NSP, NSD	Ancient, widespread, relaxed substitutions, stable copy-number, maintained neighborhood, widely alignable, constrained sequence, amino acid conservation, population conservation
SPZ	STA	–	Stable copy-number
TOLL	AGE, UNI, DUP, ACN, WGA	PDN, SEL, CON	Ancient, widespread, duplications, widely alignable, constrained substitutions

Note.—For each immune-related immune family, evolutionary features with significantly higher or significantly lower metrics compared with other immune families are listed with summarized interpretations.

### Families with Similar Functional Roles Exhibit Similar Evolutionary Profiles

**Fig. 2.**
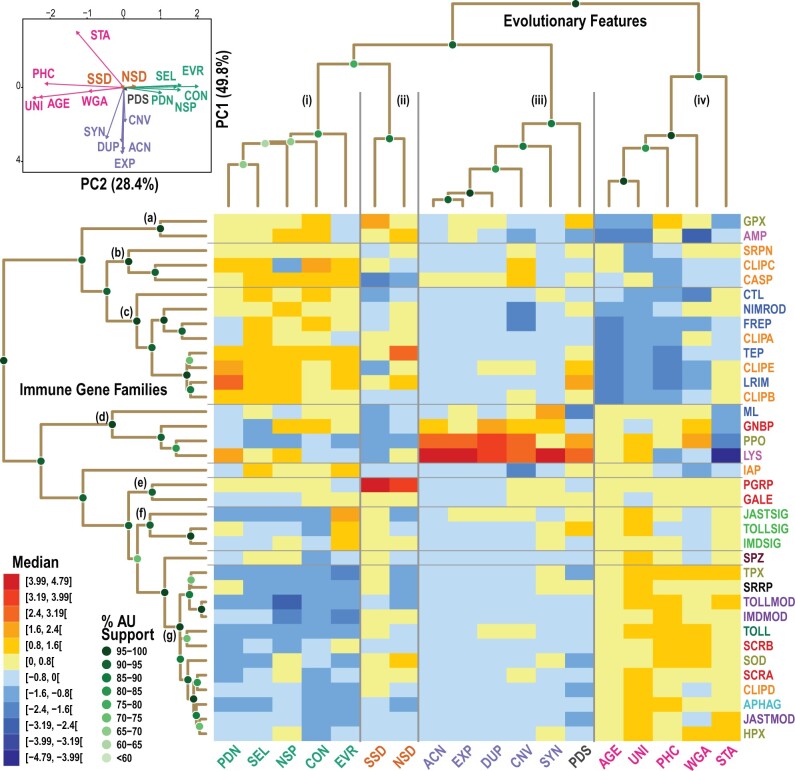
Clustering heatmap and dendrograms of immune families and their evolutionary features. Groupings of families and subsets of features delineated by hierarchical clustering using the matrix of evolutionary feature profiles of all immune gene families. Hierarchical clustering results are visualized for the immune families (*n* = 36) and evolutionary features (*n* = 18) using scaled median metrics with a Pearson’s correlation-based distance matrix and average linkage agglomerative clustering. The heatmap displays the relative values of the scaled metrics from low in blue to high in red. The dendrograms show the quantified distances (similarities) between each of the families, and between each of the features, and their groupings, determined by the clustering algorithm and distance method. Support for each node of the two dendrograms is shown with green-filled circles, using multiscale bootstrap resampling to estimate AU support values. PCA supports three major groupings of the four subsets of evolutionary features with PC1 and PC2 capturing 78.2% of the variance. Feature acronyms are defined in table 1. Family acronyms are defined in table 2 and are colored according to categories defined based on their putative roles in the principal immune phases: classical recognition (red), other recognition (blue), pathway signaling (bright green), pathway modulation (purple), cascade modulation (orange), antimicrobial effectors (pink), effector enzymes (olive green), autophagy (dark cyan), RNAi (black), cytokines (brown), and toll receptors (dark green). See text for definitions of evolutionary feature acronyms, colored according to groupings in the dendrogram and PCA. Clustering heatmap and dendrograms of immune families and their evolutionary features using mean metrics are presented in supplementary figure S2, Supplementary Material online.

Several bootstrap-supported groupings of families and subsets of features are revealed when hierarchical clustering is applied to the matrix of evolutionary feature profiles of all mosquito immune gene families (fig. 2). Clustering aims to objectively delineate the hierarchical similarities amongst families and features to identify subsets of features that vary in concert, and groups of evolutionarily similar families (see Materials and Methods). Employing family median (fig. 2) and mean (supplementary fig. S2, Supplementary Material online) metric values to build a dissimilarity matrix with Pearson’s correlation distances and performing bootstrapped clustering with the average linkage method results in several well-supported subsets and groupings. Using Pearson’s correlation distances for clustering aims to give weight to the metric directionalities rather than their magnitudes or ranks (Kassambara 2017), to identify families with similar evolutionary feature profiles. Nevertheless, clustering with alternative distance functions (Spearman’s and Kendall’s correlation, and Euclidean distances) and additional agglomerative clustering methods (Single, Complete, and Median linkage) confirms support for many of the observed hierarchical similarities (supplementary figs. S3–S6, additional file 2, Supplementary Material online). Furthermore, clustering using principal components instead of the metric values themselves also identifies several of the observed family groupings (supplementary fig. S7, Supplementary Material online). Overall, there are four main subsets of evolutionary features that consistently cluster together and somewhat more variable groupings of gene families depending on the combinations of metrics and methods applied.

First, with respect to evolutionary features (see table 1 for feature summary descriptions), four subsets of features are repeatedly and robustly recovered: (i) PAML’s dN, PAML’s dN/dS (SEL), the proportion of nonsynonymous SNPs, CON (gene losses), and evolutionary rate (protein sequence divergence); (ii) densities of synonymous and nonsynonymous SNPs; (iii) ACN, EXP, duplications, and CNV, often also including SYN as in figure 2; and (iv) age, universality, constraint, and alignability, often also including stability as in figure 2. These subsets are also recovered when clustering using metric means rather than medians, with the exception of PAML’s dS (supplementary fig. S2, Supplementary Material online). Principal component analysis (PCA) of both the median and mean metrics supports three major groupings of the four subsets, with PC1 dominated by set (iii) features contrasted by stability, and with PC2 clearly separating set (i) from set (iv) features (fig. 2; supplementary figs. S2 and S8, Supplementary Material online).

Set (i) captures both gene losses and several features related to protein sequence divergence. PAML’s dN and dN/dS are computed from codon analysis of multiple protein sequence alignments, and the evolutionary rates are computed from amino acid similarities from all-against-all protein alignments, thus they are expected to vary in concert. The observed grouping of the proportion of nonsynonymous SNPs with these protein-alignment-based metrics suggests that long-term divergence over millions of years of mosquito evolution is reflected in population-level polymorphism today. The grouping of gene losses with these sequence divergence and diversity features may appear less intuitive; however, correlations between the propensity for gene loss and sequence evolutionary rates have been observed previously from analyses of orthologs from seven distantly related eukaryotes (Krylov 2003). Here with a larger set of more closely related species (43 insects but mostly mosquitoes and other dipterans) this pattern is recovered while focusing exclusively on immune-related genes. Set (ii) groups together the expected correlated densities of genome-wide synonymous and nonsynonymous SNPs.

Set (iii) captures all the copy-number features related to gene birth, linked to local genomic organization (SYN). Gene duplications lead to higher and often more variable copy-numbers that are identified by computational analysis of gene family evolution (CAFE) as EXP, so these metrics should define different aspects of these features arising from the same underlying process and hence are expected to vary in concert. The link with maintained SYN suggests that duplicated genes often also maintain their local genomic neighborhoods. However, this phenomenon is driven by only a small subset of families with both elevated DUP and SYN metrics: GNBPs, MD-2-like proteins (MLs), and particularly lysozymes (LYSs; fig. 1). For these immune genes it appears that retaining their relative genomic locations played an important role in maintaining their functionalities after duplicating in the mosquito or anopheline ancestor. Set (iv) captures the taxonomic spread features together with DNA-level sequence conservation and constraint, linked to gene family copy-number stability. This grouping clearly connects conservation at whole-gene and nucleotide levels, with older widespread immunity genes generally showing signs of greater constraints. In general, older genes do appear to evolve more slowly than younger ones (Albà and Castresana 2005); they are also longer, more highly expressed, and subject to stronger purifying selection (Wolf et al. 2009). In addition to constrained sequence evolution, genes functionally characterized as essential are more likely to be ancient and widespread (Waterhouse et al. 2011). This highlights the ancient origins and essential roles of several core components of the insect immune system that have been maintained over millions of years of evolution.

Clustering with a subset of 12 evolutionary features after excluding PAML-based (dN, dS) and variation-based (SNPs) metrics recovers sets (i), (iii), and (iv) observed with the full suite of metrics (supplementary figs. S9 and S10, Supplementary Material online). Thus the associations between gene loss and protein sequence divergence, between DUP and SYN, and between taxonomic spread and DNA-level sequence conservation, are identifiable using this subset of features. Performing the same clustering analyses with the *D. melanogaster* immune gene catalog also recovers the links between gene loss and protein sequence divergence, and between taxonomic spread and DNA-level sequence conservation (supplementary figs. S11 and S12, Supplementary Material online). However, despite MLs and LYSs showing the same trend as for *An. gambiae*, SYN is no longer associated with copy-number features related to gene birth, indicating that maintaining genomic neighborhoods after gene duplication events is a family-dependent phenomenon rather than a global trend. The GNBPs offer a specific example, where the birth of the B-type GNBPs in the mosquito ancestor produced a new subfamily with members showing elevated conservation of their genomic neighborhoods.

The evolutionary profiles describe contrasting features of gene families and pathway members implicated in immune responses. The suite of features covers a wide spectrum of gene family evolutionary dynamics that can be broadly summarized by three main axes delineated by the major PCA groupings: axis 1, DUP and SYN; axis 2, maintenance/stability and sequence conservation; and axis 3, loss and sequence divergence. Axis 1 might be driven by only a subset of families, but the pattern is intuitive when considering the advantage of maintaining expression regulatory coordination across sets of duplicated genes. Axes 2 and 3 appear to reflect global trends in gene evolutionary dynamics observed in different taxa and over different timescales, suggesting that a broadly common set of rules also applies to the evolution of components of the immune system.

With respect to gene families (see table 2 for family summary descriptions), several groupings of different sizes are recovered: from top to bottom in figure 2 (a) AMPs and glutathione peroxidases; (b) cysteine-aspartic and CLIPC proteases with serine protease inhibitors; (c) LRIMs, TEPs, CLIPA protease homologs, CLIPB&E proteases, CTL, and fibrinogen-related and Nimrod proteins; (d) GNBPs, MLs, lysozymes, and PPOs; (e) PGRPs and galectins; (f) Toll, Imd, and JAK/STAT signaling proteins; and (g) a large set comprising autophagy and RNAi-related proteins, Toll, Imd, and JAK/STAT pathway modulators, toll receptors, scavenger receptors A and B, CLIPD proteases, superoxide dismutases, as well as heme and thioredoxin peroxidases. Clustering with metric means rather than medians results in different hierarchies but with several broadly similar groupings including: LRIMs, CLIPA protease homologs, CLIPB&E proteases, and fibrinogen-related proteins; cysteine-aspartic and CLIPC proteases; GNBPs, MLs, lysozymes, and PPOs; and a large set comprising autophagy and RNAi-related proteins, Toll, Imd, and JAK/STAT pathway modulators, toll receptors and galectins, scavenger receptors A and B, CLIPD proteases, superoxide dismutases, as well as heme and thioredoxin peroxidases (supplementary fig. S2, Supplementary Material online). Similar variations of these groupings are obtained when clustering means or medians using alternative distance-clustering method combinations (supplementary additional file 2, Supplementary Material online). Combining this variation with results from bootstrapping provides a measure of evolutionary profile similarity between all pairs of families (see Materials and Methods). The families that most frequently cluster together using metric means (supplementary fig. S5, Supplementary Material online) or medians (supplementary fig. S6, Supplementary Material online) include: PGRPs, galectins, GNBPs, MLs, lysozymes, and PPOs; cysteine-aspartic and CLIPC proteases; LRIMs, TEPs, CLIPA protease homologs, CLIPB&E proteases, and fibrinogen-related proteins; and a large set comprising autophagy and RNAi-related proteins, Toll, Imd, and JAK/STAT pathway modulators, toll receptors, scavenger receptors A and B, CLIPD proteases, superoxide dismutases, as well as heme and thioredoxin peroxidases. Thus, although the gene family groupings are more variable across different distance-clustering method combinations than those of the evolutionary features, the results identify families with consistently similar evolutionary profiles.

Evolutionary profile clustering identifies features that are shared by genes and families within each of the major immune phases. Pairs of recognition protein families with similar profiles include PGRPs and galectins, A- and B-type scavenger receptors, and GNBPs and MLs, also indicating that MLs more closely resemble classical than other recognition families, thereby warranting their reclassification (fig. 2). PGRPs can bind bacterial cell wall Dap- or Lys-type peptidoglycans (Wang et al. 2019), whereas galectins can bind surface β-galactosides (Vasta 2020). Similarly, GNBPs can recognize β-1,3-glucans that make up structural polysaccharides of yeast cell walls (Rao et al. 2018), whereas MLs can bind lipopolysaccharides from the outer membrane of Gram-negative bacteria (Shi et al. 2012). A- and B-type scavenger receptors may have broader ligand specificities including lipoproteins and surface molecules of Gram-negative and Gram-positive bacteria (Alquraini and El Khoury 2020). As important pattern recognition receptors in animal immunity, these are all expectedly old families; however, despite interacting with pathogens they remain relatively constrained (DNA-level) and do not show extreme protein sequence divergence (fig. 2). This apparent lack of evidence for an arms race scenario may in fact reflect the relatively limited structural diversity of the main microbial ligands—peptidoglycan, β-1,3-glucan, lipopolysaccharide—they must bind to or cleave.

Signaling genes of the Toll, Imd, and JAK/STAT pathways group together, being generally ancient and stable but with remarkably elevated rates of protein sequence divergence. Their copy-number stability is possibly a reflection of constraints imposed by the large disruptive potential of duplicates on core signal transduction functionality. Their protein products work together as interacting partners, including the death-domain-mediated MyD88-Tube-Pelle complex of the Toll pathway (Valanne et al. 2011), the Imd pathway’s Imd–Fadd–Dredd, Tab 2–Tak1, and IκB kinase complexes (Myllymäki et al. 2014), and the Domeless–Hopscotch complex of the JAK/STAT pathway (Myllymäki and Rämet 2014). Their greater sequence divergence could therefore be explained by the accumulation of compensatory amino acid changes that maintain key interactions amongst these partners, and overall pathway functionality. The signaling pathway modulators are also old and stable, but instead show constrained sequence evolution. These include several enzymes, such as ubiquitinases like Effete and Bendless, or E3 ligases like Pellino and Pias, which are under strong constraints to maintain their enzymatic activities. They are involved in proteasomal degradation and are therefore also critical for many other processes beyond immune signaling (Glickman and Ciechanover 2002). Other enzymes including the superoxide dismutases as well as the heme and thioredoxin peroxidases involved in reactive oxygen species metabolism (Hillyer 2016), show similarly conservative evolutionary profiles (fig. 2). Proteolytically activated PPOs oxidize phenols in the melanin production process (Nakhleh, El Moussawi, et al. 2017) and also show similar sequence constraints; however, multiple gene duplications result in an evolutionary profile that is radically different. Thus although there is some variation, in general the functional constraints on these types of enzymes appear to restrict their patterns of molecular divergence.

Members of ancient pathways controlling RNAi (SRRP) and autophagy (APHAG) responses group with other conservative evolutionary profiles characterized by low gene turnover and low sequence evolutionary rates (fig. 2). In contrast, much more dynamic evolutionary profiles characterize the grouping of families of immune cascade modulators like CTL, CLIPA protease homologs and CLIPB&E proteases, regulators of melanization responses like serine protease inhibitors, and key players in mosquito complement-like responses, like TEPs and LRIMs. Although melanization is conserved across arthropods (Hillyer 2016), the proteolytic cascades that trigger or dampen melanin production often involve lineage-specific members of large gene families including these dynamically evolving modulators (Gulley et al. 2013; Meekins et al. 2017; Bishnoi et al. 2019; El Moussawi et al. 2019). The complement-like responses centered on TEPs and LRIMs are specific to mosquitoes, and are also triggered and regulated by members of these large and dynamic families (Blandin et al. 2008; Fraiture et al. 2009; Povelones et al. 2009, 2013, 2016). Based on understanding molecular functions of only a limited number of genes from these families, it appears that immune responses requiring such finely tuned activation, amplification, and deactivation processes source components from dynamically evolving families from which to build functional modules. The families involved are characterized with evolutionary profiles showing a pattern of younger and less widespread orthologs, with lower-sequence constraints, and often elevated signatures of selection and population-level variation. This dynamism is more consistent with an arms race scenario, where the effectiveness of such functional modules is continuously being tested by evolving pathogen attacks and evasion strategies.

### Coexpression Analyses Identify Immune Families That Function in Concert

**Fig. 3.**
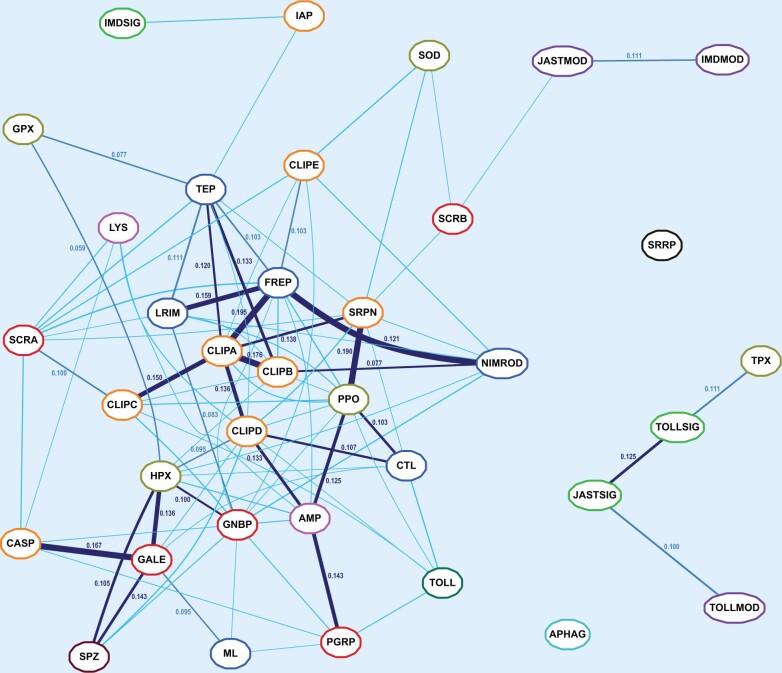
Network of immune family expression similarities based on the VectorBase Expression Map. The network layout optimized with a spring model provides a 2D visualization of expression similarities for pairwise comparisons of all 36 immune-related gene families computed as gene co-occurrence scores across the VectorBase Expression Map (AgamP4.11 VB-2019-02). Families with more similar gene expression profiles are placed closer together in the graph. Significant co-occurrences are indicated with connecting lines: light blue <0.05, royal blue <0.01, and dark blue <0.005 with line thickness scaled to the *P* value and co-occurrence scores indicated for all pairs with *P*<0.01. Family acronyms are defined in table 2 and are colored according to categories defined based on their putative roles in the principal immune phases: classical recognition (red), other recognition (blue), pathway signaling (bright green), pathway modulation (purple), cascade modulation (orange), antimicrobial effectors (pink), effector enzymes (olive green), autophagy (dark cyan), RNAi (black), cytokines (brown), and toll receptors (dark green).

Analysis of multisample gene expression data shows that families with the strongest fine-scale or broad-scale expression similarities include many pairs whose members are known to function together in vivo (fig. 3; supplementary fig. S13, Supplementary Material online). Thus, without presupposing any functional categorizations, the similar expression profiles highlight families whose members are likely working together across different conditions. Gene expression-based quantification of functional similarities amongst immune gene families provides an alternative objective classification that complements the classical categorizations based on their putative roles in key immune responses. The VectorBase Expression Map (MacCallum et al. 2011) defines clusters of genes with similar expression profiles for 12,672 genes using normalized data across 202 conditions, enabling the quantification of fine-scale or broad-scale gene expression similarities amongst all pairs of immune-related families. Pairwise family similarities are computed as the frequency of co-occurrences of gene family members in the same region of the map, with significance assessed taking into account family sizes and expression cluster sizes (see Materials and Methods). Visualizing pairwise family similarities as a spring model layout network optimized with the *neato* tool from the Graphviz package (Gansner and North 2000; Gansner et al. 2005) identifies subsets of families with putative roles in common immune processes (fig. 3). These prominently include a quintet of families with highly and significantly overlapping expression patterns: LRIMs, TEPs, FREPs, CLIPAs, and CLIPBs (fig. 3), with members implicated in coordinating and executing mosquito complement system responses (Povelones et al. 2016; Reyes Ruiz et al. 2019).

*Anopheles* *gambiae* TEP1 forms a stable protein complex with a heterodimer of LRIM1 and APL1A/B/C (LRIM2 paralogs) in the hemolymph until the complement response is activated (Fraiture et al. 2009; Povelones et al. 2009; Williams et al. 2015), so coordinated coexpression of these genes is important for their functions. Like the LRIMs and TEPs, the FREPs are also found in the hemolymph, and several members are infection-responsive and important for defense, for example, FREP57/FBN8, FREP13/FBN9, and FREP40/FBN39 (Dong et al. 2006; Dong and Dimopoulos 2009; Simões et al. 2017). FREPs themselves might dimerize or oligomerize, but whether they interact directly with TEPs and/or LRIMs in mosquitoes remains unknown, although evidence from snails indicates that FREPs and TEPs do interact (Li et al. 2020), and the observed expression similarities support at least some functional, if not physical, interaction. CLIPA serine protease homologs are positive and negative regulators of immune responses mediated by TEP1, for example, CLIPA8 (Schnitger et al. 2007), SPCLIP1/CLIPA30 (Povelones et al. 2013), CLIPA2 (Yassine et al. 2014), CLIPA14 (Nakhleh, Christophides, et al. 2017), and CLIPA28 (El Moussawi et al. 2019). These regulatory modules also involve the catalytically active CLIPBs, for example, CLIPB14 and CLIPB15 (Volz et al. 2005), CLIPB8 (Zhang et al. 2016), and CLIPB10 (Zhang et al. 2021), and together CLIPAs and CLIPBs are also key modulators of melanization responses (Volz et al. 2006). The available evidence therefore supports the family-level expression analyses that demonstrate highly and significantly overlapping expression patterns (fig. 3) of members of this quintet of families that function in concert.

Of this quintet, expression of CLIPA protease homologs is additionally strongly and significantly similar to that of CLIPCs, CLIPDs, and SRPNs (serpins, or serine protease inhibitors). The CLIPC9 protease has recently been shown to regulate melanization downstream of SPCLIP1/CLIPA30, CLIPA8, and CLIPA28, and to be inhibited by SRPN2 (Sousa et al. 2020). CLIPC2 may function together with SRPN7 controlling the activation of effector mechanisms (Blumberg et al. 2013). Specific roles for CLIPDs, which show an evolutionary profile distinct from the other CLIPs (fig. 2), remain largely unknown. Serpins themselves are most similar in expression to PPOs, both of which would need to be replenished after being depleted during melanization responses (Gulley et al. 2013; Nakhleh, El Moussawi, et al. 2017). The PPOs in turn appear significantly similar to the CTL, which are generally considered glycan-binding recognition proteins, but at least two members—CTL4 and CTLMA2—are key regulators of melanization downstream of immune recognition (Schnitger et al. 2009; Bishnoi et al. 2019). The family-level expression similarities (fig. 3) therefore derive from the functional links amongst the CLIP, CTL, and SRPN family members that modulate the activation of melanization, and the PPO enzyme effectors of melanization activity.

Amongst classical recognition proteins, PGRPs and GNBPs are most similar, and their expression patterns both closely match those of AMPs and MD-2-like lipid recognition proteins. These similarities are driven by the upregulation of members of these gene families upon infection or following a blood meal, which promotes growth of the gut microbiota, for example, in response to blood-feeding (Dana et al. 2005), microbes (Aguilar et al. 2005), *Plasmodium* (Dong et al. 2006), or fungi (Ramirez et al. 2020). They are nevertheless not as tightly interconnected as components of the complement and melanization responses, possibly reflecting the contrast between broad-scope protection of these systems versus the generally much more pathogen-specific activities of different families of recognition proteins and antimicrobial effectors. Indeed feeding into and/or being transcriptionally activated by different immune signaling pathways means that these families may be thought of as performing analogous roles rather than functioning in concert per se. However, learning more about signaling crosstalk and response overlap has shifted thinking from traditional functional distinctions amongst immune pathways (Kounatidis and Ligoxygakis 2012). Thus, these similarities might reflect somewhat overlapping responses, but also a common readiness or priming to face newly perceived threats.

Notably, expression patterns of pathway signaling and modulation components remain distinct from the recognition and response families: Imd and JAK/STAT pathway modulators are significantly similar, whereas Toll pathway modulators group together with Toll and JAK/STAT pathway signaling members. Genes involved in RNAi (SRRP) and autophagy (APHAG) responses do not show significant similarities in expression patterns to other families; however, SRRP and APHAG genes have highly and significantly overlapping expression patterns at broad-scale resolution, and are most similar to modulators of all three pathways (supplementary fig. S14, Supplementary Material online). At broad-scale resolution, the distinction between pathway signaling/modulation and recognition/response families is accentuated, whereas the melanization and complement responses become more closely interlinked. Many of the most similar families also show substantially overlapping expression patterns when quantifying similarities across coexpression modules built from a subset of immune-related experimental conditions (see Materials and Methods, supplementary figs. S15 and S16, table S2, additional file 3, Supplementary Material online). For example, families implicated in complement system responses again show similar expression patterns (supplementary fig. S17, Supplementary Material online), and at a broader-scale resolution become more closely associated with melanization responses (supplementary fig. S18, Supplementary Material online). At broad-scale resolution pairs of similar recognition families include GNBPs and PGRPs, GNBPs and MLs, as well as galectins and B-type scavenger receptors, whereas at both resolutions Imd and JAK/STAT pathway signaling members are highly and significantly similar. Multicondition coexpression analysis therefore identifies gene expression similarities amongst sets of immune-related families with members that are known or inferred to function in concert.

### Complement-Related Families Exhibit Elevated Evolutionary-Functional Similarities

**Fig. 4.**
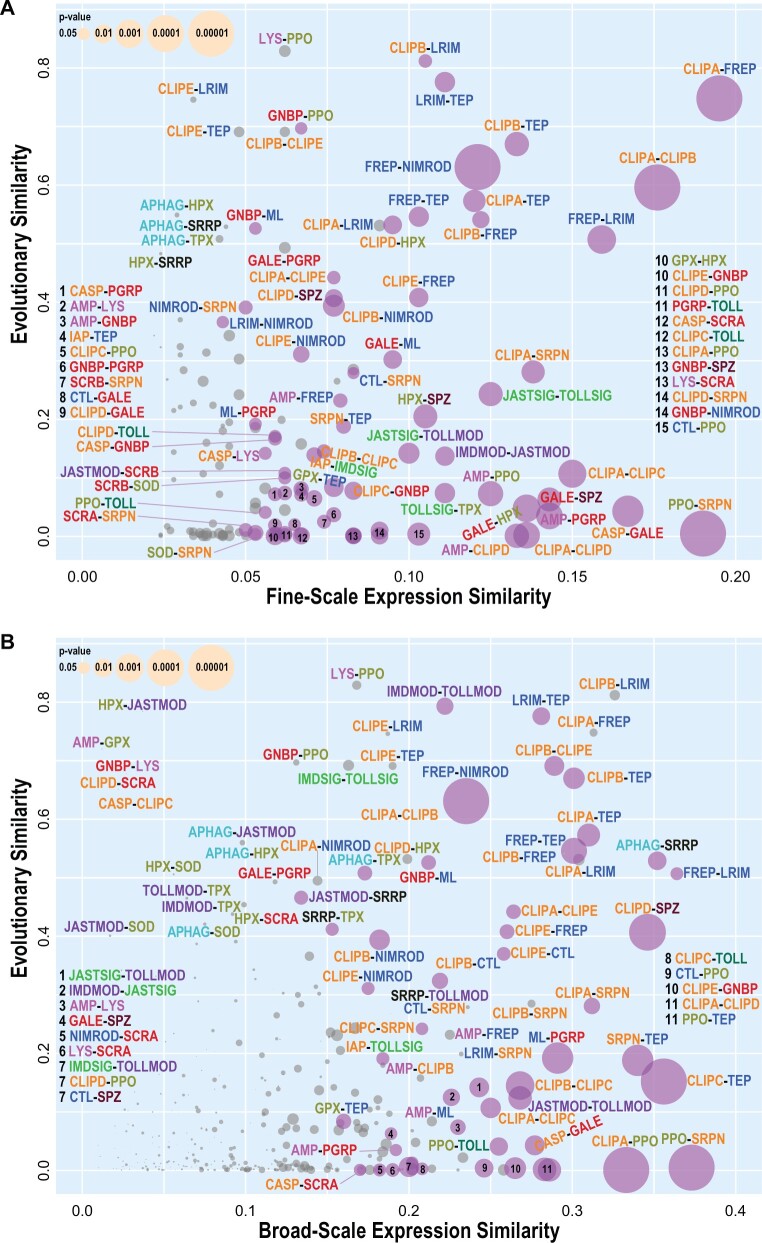
Pairwise comparisons of immune family expression similarity and evolutionary similarity. Evolutionary similarities (based on feature metric medians) of pairs of gene families are compared with their expression similarities at (*A*) fine-scale resolution and (*B*) broad-scale resolution. Pairs of families with significant (*P*<0.05) gene expression co-occurrence scores are shown with purple circles, with nonsignificant pairs shown in gray, and with circle sizes scaled by the *P* value. Family acronyms are defined in table 2 and are colored according to categories defined based on their putative roles in the principal immune phases: classical recognition (red), other recognition (blue), pathway signaling (bright green), pathway modulation (purple), cascade modulation (orange), antimicrobial effectors (pink), effector enzymes (olive green), autophagy (dark cyan), RNAi (black), cytokines (brown), and toll receptors (dark green).

Immune gene family evolutionary-functional correspondences are revealed by employing quantifications of evolutionary similarities based on gene family feature profiling and of functional similarities based on gene family expression patterns (fig. 4; supplementary additional file 4, Supplementary Material online). Most prominently, families involved in mosquito complement system responses show both high evolutionary similarities and high fine-scale and broad-scale expression similarities: recognition family pairs of LRIMs–TEPs, FREPs–TEPs, and FREPs–LRIMs, as well as modulator-recognition family pairs of CLIPAs with FREPs and TEPs, and CLIPBs with FREPs, TEPs, and LRIMs. Members of these principal complement–response gene families exhibit common expression and evolutionary profiles suggestive of common constraints. Both TEPs and LRIMs are also highly evolutionarily similar to CLIPEs, for which specific roles in complement responses remain largely unknown, but with which their expression similarity increases at broad-scale resolution, albeit remaining nonsignificant. The CLIPA protease homologs and CLIPB proteases form a highly similar pair, but their strong and significant expression similarity is not maintained at broad-scale resolution, suggesting tight functional coupling of these key modulators. Conversely, CLIPB and CLIPE modulators also form a highly similar pair, but with strong and significant expression similarity only at broad-scale resolution. In contrast, FREP-NIMROD expression similarity is maintained at both resolutions and it is amongst the most significant of all family pairs that also show high evolutionary similarities. Although a much smaller gene family than the FREPs, NIMRODs including *draper*, *nimrod*, and *eater*, are also infection-responsive and important for defense (Midega et al. 2013; Estévez-Lao and Hillyer 2014). Combining results from evolutionary profiling and knowledge-blind functional clustering therefore identifies families that appear both evolutionarily and functionally similar. These similarities are notably pronounced for families with members known to function in concert to coordinate and execute mosquito complement system responses (Povelones et al. 2016; Reyes Ruiz et al. 2019).

Additional families with above average evolutionary and expression similarities at both resolutions include another pair of modulators (CLIPA-SRPN), and another modulator-recognition pair (CLIPE-FREP). Although CLIPAs and SRPNs are known to function together in cascades regulating melanization (El Moussawi et al. 2019), potential functional interactions between CLIPEs and FREPs remain to be explored. The melanization modulator-effector pair of SRPNs and PPOs shows the highest expression similarity at both resolutions, but with negligible evolutionary similarity, suggesting that regulating these responses and executing them are subject to different constraints. Amongst other recognition proteins, MLs show above average evolutionary and expression similarities to the classical recognition families of galectins (GALEs) at fine-scale resolution, and PGRPs at broad-scale resolution with lower but still significant expression similarity at fine-scale resolution. Compared with galectins or PGRPs, the MLs are evolutionarily more similar to GNBPs, with which they show lower, but still significant, expression similarity. These patterns suggest analogous functionalities—recognition of foreign—with different specificities for lipopolysaccharides, β-galactosides, peptidoglycans, or β-1,3-glucans, that arise depending on the pathogen/microbe community composition. Common constraints faced by classical recognition phase families appear to produce similarities amongst their evolutionary trajectories, with functional similarities quantified through gene expression patterns possibly arising through immune pathway signaling crosstalk and priming (Kounatidis and Ligoxygakis 2012).

Evolutionarily similar families that only show high expression similarities at broad-scale resolution include modulators of the Imd and Toll pathways (IMDMOD-TOLLMOD) and genes involved in autophagy and RNAi responses (APHAG-SRRP). At fine-scale resolution, pathway components from JAK/STAT and Toll signaling (JASTSIG-TOLLSIG), Imd and JAK/STAT modulation (IMDMOD-JASTMOD), and JAK/STAT signaling and Toll modulation (JASTSIG-TOLLMOD) also show above average evolutionary and expression similarities. These pathways and responses play key roles in processes other than immunity, including in development and morphogenesis, so their gene expression-based functional similarities will vary depending on the conditions examined. This also means that the functional constraints they experience are not solely derived from their roles in immune processes. Their functional similarities are more stably evident when the modules are abstracted to analogous phases of signal input, signal processing, and signal output. Whether functionally similar or analogous, these immune-related pathways and responses exhibit common conservative evolutionary profiles that distinguish them from other more dynamically evolving components of the immune system (fig. 2). These constrained evolutionary features could result from the effects of pleiotropy, and possibly the modular architectures, on the trade-offs during adaptive evolution producing a limited range of available trajectories (Mauro and Ghalambor 2020).

## Conclusions

Through quantitative evolutionary feature profiling of genes and gene families, integrated with knowledge- and expression-based functional categorizations, our multispecies comparative immunogenomic analyses identified evolutionary-functional correspondences suggesting that constraints on genes with similar or analogous functions govern their evolutionary trajectories. The profiles delineate whether and how each family deviates from the feature value distributions of other families, and provide the substrate for clustering to define similarities amongst families and features. We employed insect innate immunity as our test case study system because the key implicated pathways and component gene families have been well characterized. While acknowledging that responses to infections involve diverse processes beyond the canonical immune system (Sackton 2019) and that immune-related genes may also function in other biological processes, this prior knowledge provided specific examples and strong expectations of types of genes with similar functions and distinguishing patterns of evolution, enabling the interpretation of observed correspondences within an established framework. Feature analysis within the limits of our study system identified three main axes of evolutionary trajectories characterized by gene duplication and SYN, gene maintenance/stability and sequence conservation, and gene loss and sequence divergence. Clustering highlighted similar and contrasting patterns across these axes amongst subsets of immune gene families. For example, classical recognition families, including the herein reclassified MLs, showed patterns that can be explained by the limited structural diversity of the principal microbial ligands with which they interact. Pathway signaling genes on the other hand exhibited trajectories that could relate to physical interactions of protein complexes and constraints from the effects of pleiotropy and disruptive effects of gene duplicates on signal transduction. Functional similarities defined by coexpression analyses recovered sets of immune-related families with members that are known or inferred to function in concert. Most prominently, these included families involved in the complement system and melanization responses, both of which occur mainly in the hemolymph. Comparing these with feature-based clustering results identified evolutionary-functional correspondences that were particularly striking amongst families with members known to function together in the coordination and execution of complement system responses. Our results suggest that where and how different genes participate in immune defense responses limit the range of possible evolutionary scenarios that are tolerated by natural selection. Our test case analyses of insect immunity that explored approaches to quantify gene evolutionary histories and relate these to gene functions highlight the potential for future applications to advancing understanding of functional constraints on evolution. Further developing and applying such comparative genomics approaches to explore constraints in evolutionary biology could offer opportunities to advance the understanding of how functional constraints on different components of biological systems govern their evolutionary trajectories.

## Materials and Methods

### Orthology, Variation, Alignment, and Expression Data

OGs of genes were defined using the OrthoDB (Kriventseva et al. 2015) orthology delineation procedure across 21 mosquitoes and 22 other insects (see supplementary materials, orthology data, Supplementary Material online). OrthoDB employs all-against-all protein sequence alignments to first identify all best reciprocal hits (BRHs) between all genes from each pair of species (Zdobnov et al. 2017). It then uses a graph-based clustering procedure that starts with BRH triangulation to progressively build OGs that include all genes descended from a single gene in the last common ancestor. SNPs for *An. gambiae* PEST, including all synonymous and nonsynonymous SNPs in annotated coding regions, were retrieved using the BioMart data mining tool from VectorBase (Giraldo-Calderón et al. 2015). The SNPs derive from eight variation data sets hosted at VectorBase (Neafsey et al. 2010; White et al. 2011; Weetman et al. 2012; Markianos et al. 2016; Hammond et al. 2017; Miles et al. 2017; Wiltshire et al. 2018). Multispecies whole genome alignments were generated from the assemblies of 22 mosquitoes available from VectorBase and 36 *Drosophila* available from The National Center for Biotechnology Information (supplementary table S1, Supplementary Material online). The alignment process starts with pairwise sequence comparisons that are then progressively combined following the species phylogeny using the MultiZ approach of the Threaded Blockset Aligner (Blanchette et al. 2004). Expression data for *An. gambiae* genes were retrieved from VectorBase (Expression Stats VB-2019-06) as log2 transformed expression values for 13,201 genes across 291 conditions (mean, variance, and number of replicates). Immune gene family coexpression analysis employed these expression statistics using a subset of the conditions to build coexpression modules. Coexpression analysis also employed clusters of genes defined by the VectorBase Expression Map (MacCallum et al. 2011), with gene membership of all clusters/cells retrieved from the AgamP4.11 VB-2019-02 map (comprising 12,672 genes and based on 202 conditions).

### *Anopheles gambiae* and *D. melanogaster* Immunity Gene Catalogs

The catalogs of *An. gambiae* and *D. melanogaster* immune-related genes were built by combining and updating the results of previous comparative immunogenomics studies (Christophides et al. 2002; Waterhouse et al. 2007; Bartholomay et al. 2010; Neafsey et al. 2015). *Anophelesgambiae* and *D. melanogaster* gene and OG membership for 36 immune-related gene families and subfamilies are summarized in table 2.

### Orthology-Based Evolutionary Features

Features were quantified as a suite of 13 orthology-based evolutionary metrics per OG that included: the evolutionary age (AGE) of the last common ancestor in terms of millions of years since divergence from the ultrametric species phylogeny; the universality (UNI) computed as the proportion of the total species present; the duplicability (DUP) computed as the proportion of species present with multicopy orthologs; the average ortholog copy number (ACN); the copy number variation (CNV) computed as the standard deviation of ortholog counts per species present divided by the ACN. PAML (Yang 2007) was employed using the M0 model on the alignments of OG member sequences to compute the number of synonymous substitutions per synonymous site (PDS); the number of nonsynonymous substitutions per nonsynonymous site (PDN); and the nonsynonymous to synonymous ratio (SEL). Gene turnover was estimated using the CAFE (Han et al. 2013) tool in order to quantify proportions of gene gains (expansions, EXP), gene losses (contractions, CON), or no copy-number changes (stable, STA). Orthology data combined with genomic location data were used to quantify SYN conservation as the proportion of orthologs that maintain their orthologous neighbors in the genomes of the other species. Finally, the EVR of each OG corresponds to the average rate of protein sequence divergence normalized by the distance between each pair of species as computed by OrthoDB (Waterhouse et al. 2013).

### Variation-Based and Alignment-Based Evolutionary Features

Five additional evolutionary feature metrics were computed from polymorphism data and whole genome alignments. The population genomics data for *An. gambiae* retrieved from VectorBase were used to compute per-gene metrics of the proportion of all coding-sequence SNPs that were nonsynonymous (NSP), as well as the nonsynonymous (NSD) and synonymous (SSD) SNP densities as the number of SNPs divided by the total coding-sequence length. Multispecies whole genome alignments were used to compute per-nucleotide metrics of conservation and constraint. WGA measures the proportion of the full set of 22 mosquitoes or 36 *Drosophila* that were aligned to the *An. gambiae* or *D. melanogaster* reference genomes, respectively, for each nucleotide. PhastCons (Siepel et al. 2005) was used to estimate PHC from the whole genome alignments. Per-nucleotide values were averaged over the full coding-sequence lengths of all genes to obtain per-gene metrics. The variation-based and alignment-based per-gene metrics were averaged over all genes in each OG to obtain the per-OG values for each of the metrics.

### Gene Family Metrics and Comparisons

The canonical immunity gene catalogs define immunity gene membership of subfamilies (e.g. cecropins, defensins, attacins), families (e.g. AMPs), and broader categories (e.g. antimicrobial effectors), and the orthology data sets define gene membership of OGs. Thus, the gene family evolutionary metrics were computed by averaging values over all OGs containing genes belonging to each cataloged immune gene family. These family-level means for each metric were compared with the means of all other OGs that contain at least one *An. gambiae* immune gene to quantify the extent to which the metrics of the OGs of a given immune gene family differ from all other immune gene containing OGs, that is, delta-mean (Δ$\bar{\text{x}}$). For graphical visualization, Δ$\bar{\text{x}}$ values were scaled by dividing by the absolute maximum Δ$\bar{\text{x}}$ per evolutionary feature and plotted with the color-blind safe RdYlBu palette from the *RColorBrewer* package from R (R Core Team 2021). The Wilcoxon rank-sum (Mann–Whitney *U*) test implemented in the *wilcox.test* function in R (default two-sided test) was used to test the significance of the difference of the distribution of each family’s OGs metric values (no scaling) compared with all other immune-related OGs for all metrics and each family. As several families contain few OGs, a permutation test implemented in R was also used to test the significance of the difference of the metric distributions. Observed Δ$\bar{\text{x}}$ was compared with Δ$\bar{\text{x}}$ from permutations of all OG metric values randomly assigned to size-matched sets. The number of permutation differences that were greater than the observed difference, divided by the total number of permutations provides an empirical estimate of the probability of obtaining a Δ$\bar{\text{x}}$ greater than the observed Δ$\bar{\text{x}}$ by chance.

### Clustering of Gene Family Metrics

To assess and quantify the similarities of the evolutionary feature profiles, hierarchical clustering of the evolutionary features and families was performed with the *hclust* function in R. For the *An. gambiae* analyses these comprised 18 features and 36 families, whereas for the mosquito-fly comparisons these comprised a common subset of 12 features and 35 families. For all evolutionary feature metrics, both the means and the medians of all OGs per family were assessed. Prior to clustering, the *scale* function in R was used to normalize all metric values by subtracting the means and then dividing the (centered) values by their standard deviations. Dissimilarity matrices were computed with the normalized metric values using three correlation-based distance methods and the Euclidean distance method in R. Clustering with *hclust* was performed with all dissimilarity matrices using single, complete, average, and median linkage agglomeration methods. To estimate statistical support for the clustering of families and features, 10,000 bootstrap replicates were performed with the *pvclust* R package. In *pvclust*, the approximately unbiased (AU) *P* values are computed using multiscale bootstrap resampling (Suzuki and Shimodaira 2006), and provide a confidence measure for each node of the cluster dendrograms of families and evolutionary features. The robustness of gene family clustering across all 16 tested distance–method combinations was further assessed by quantifying the co-occurrence of all pairs of families within subtrees of all 160,000 *pvclust* bootstrap replicates. This evolutionary profile similarity score (family subtree co-occurrence score) was computed and normalized as follows: (2 × co-occurrence of Family 1 and Family 2)/(co-occurrence of Family 1 with any Family + co-occurrence of Family 2 with any Family). Normalized scores of zero indicate that these pairs of families never appear in the same subtree and scores of one would indicate that they occur as sister lineages in all bootstrap samples from all distance–method combinations. Based on these assessments of clustering stability, the dissimilarity matrix from Pearson’s correlation method with the average linkage agglomeration method was selected. Specifically, the bootstrap replication analysis showed that the Pearson’s correlation distances with the average linkage method produced the fewest poorly supported nodes (based on AU *P* values) across immune families and evolutionary features (see supplementary methods, figs. S3 and S4, Supplementary Material online). The hierarchical clustering results were visualized as heatmaps with corresponding family and evolutionary feature dendrograms showing AU support, plotted with the *gplots* and *dendextend* (Galili 2015) R packages. PCA of the family by feature matrices of both median and mean metrics were performed with the *prcomp* function from the *stats* package in R. As well as producing well-supported nodes, the Pearson. Average distance–method approach on the scaled metrics produces similar family groupings to using the top ten principal components with the standard Euclidean-Ward.D2 distance–method approach (supplementary fig. S7, Supplementary Material online), that is, when applying standard clustering techniques after transforming the correlated metrics into principal components.

### Gene and Family Coexpression Analyses

Gene expression similarities amongst all pairs of *An. gambiae* immune-related families were quantified using the gene expression data and Expression Map (MacCallum et al. 2011) retrieved from VectorBase (Giraldo-Calderón et al. 2015). The map was analyzed to quantify co-occurrences of gene family members in the same cell on the map (fine-scale resolution of gene coexpression), and in the same supercell, the cell and its immediate eight neighboring cells on the map including toroidal neighbors (broader-scale resolution of gene coexpression). Pairwise family cell/supercell co-occurrence scores (expression similarity scores) were computed as the intersection, Family 1 n Family 2, divided by the union, Family 1 U Family 2 (i.e. number of cells with at least one gene from both Family 1 and Family 2/number of cells with at least one gene from either Family 1 or Family 2). A score of zero: the pair of families have no member genes that cluster in the same cell/supercell. A score of one: all member genes from both families always cluster in cells/supercells with at least one member of the other family. Statistical significance of the family cell/supercell co-occurrence scores was assessed with a permutation test: scores were recomputed after gene to cell assignments were randomly shuffled (10,000 permutations) preserving the total number of cells and families, and the number of genes in each cell and each family. These were used to calculate an empirical estimate of the probability (*P* value) of obtaining a co-occurrence score greater than the observed co-occurrence score by chance: the number of permutation scores that were greater than the observed score, divided by the total number of permutations. Complementary assessments of gene expression clustering were performed using the weighted correlation network analysis approach (Langfelder and Horvath 2008) on a subset of 24 conditions selected from the VectorBase gene expression data set including blood feeding experiments and tissues from Marinotti et al. (2006), Neira Oviedo et al. (2008), and Baker et al. (2011). Expression similarities of pairs of immune gene families and the significance of their co-occurrences were computed as for the Expression Map but using module membership rather than cell/supercell membership.

## Supplementary Material

Supplementary data are available at *Molecular Biology and Evolution* online.

## Supplementary Material

msab352_Supplementary_Data
