# Supplementary material for: Functional Constraints on Insect Immune System Components Govern Their Evolutionary Trajectories
Source: Mol Biol Evol. 2021 Dec 10;39(1):msab352. doi: 10.1093/molbev/msab352 (PMC8788225; doi:10.1093/molbev/msab352)
Supplement: msab352_Supplementary_Data [file msab352_supplementary_data.zip › Additional_File_1_FamilyDistributions_by_Metric.pdf]

Additional File 1

Distributions of computed OG metrics for all of the immune gene families for each evolutionary feature together with statistical assessments of the significance of deviations from the typical metric values. Per family data, coloured by superfamily/class, for each of the 18 evolutionary feature metrics: OGs, number of orthologous groups; Genes, number of genes; MW p-val, Mann Whitney U test p-value; PRM p-val, permutation test p-value. P-values less than 0.1 are highlighted. See Table 1 (main text) for descriptions of the evolutionary features and Table 2 (main text) for descriptions of the immune gene families.

Acronym	Evolutionary Feature
ACN	Average Copy Number
AGE	Taxonomic Age
CNV	Copy Number Variation
CON	Contractions
DUP	Duplicability
EVR	Evolutionary Rate
EXP	Expansions
NSD	Non-synonymous SNP Density
NSP	Non-synonymous SNP Proportion
PDN	PAML's dN
PDS	PAML's dS
PHC	PhastCons Constraint
SEL	PAML's dN/dS
SSD	Synonymous SNP Density
STA	Stability
SYN	Synteny
UNI	Universality
WGA	Whole Genome Alignability

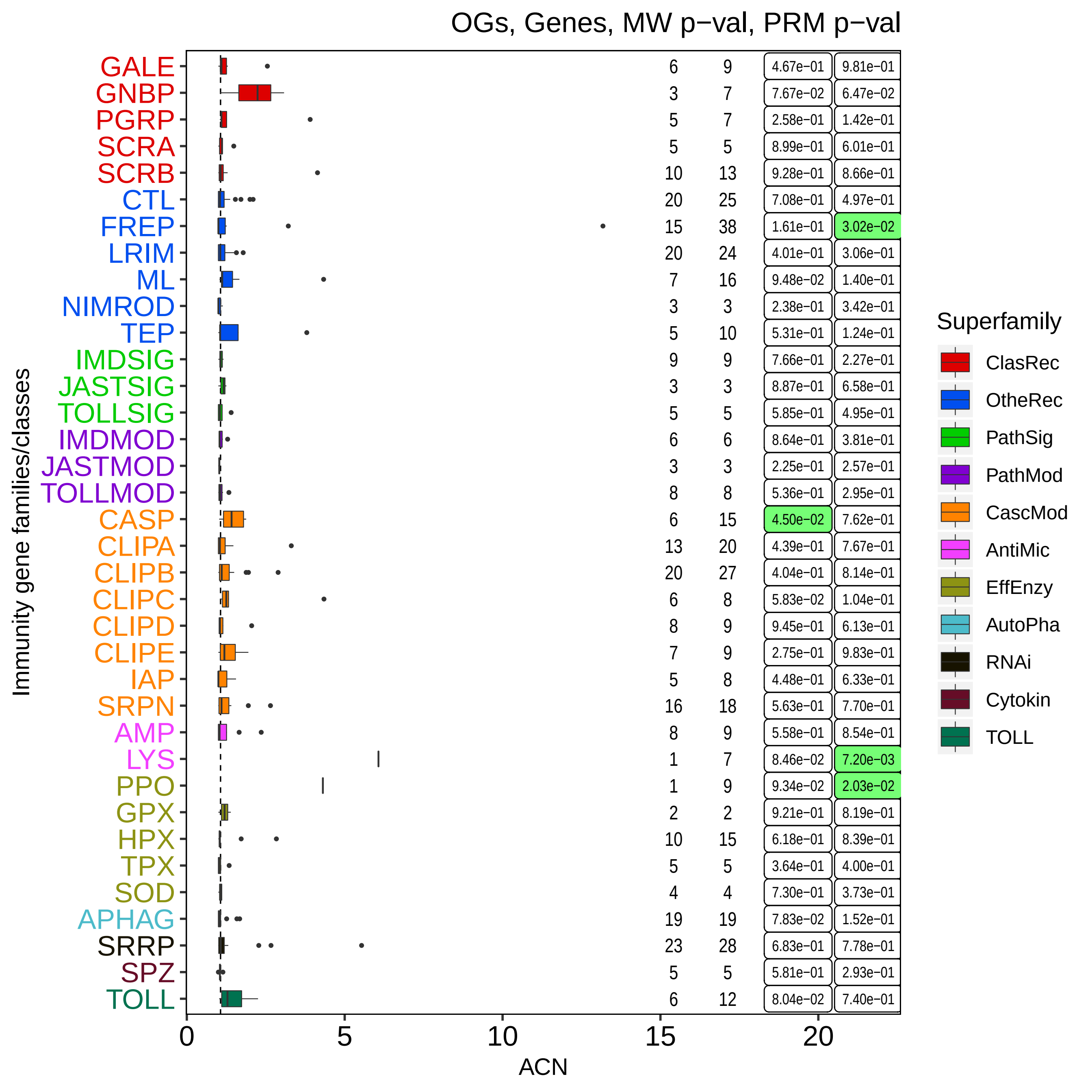

OGs, Genes, MW p−val, PRM p−val
Immunity gene families/classes
ACN
0
5
10
15
20
GALE 6 9 4.67e−01 9.81e−01
GNBP 3 7 7.67e−02 6.47e−02
PGRP 5 7 2.58e−01 1.42e−01
SCRA 5 5 8.99e−01 6.01e−01
SCRB 10 13 9.28e−01 8.66e−01
CTL 20 25 7.08e−01 4.97e−01
FREP 15 38 1.61e−01 3.02e−02
LRIM 20 24 4.01e−01 3.06e−01
ML 7 16 9.48e−02 1.40e−01
NIMROD 3 3 2.38e−01 3.42e−01
TEP 5 10 5.31e−01 1.24e−01
IMDSIG 9 9 7.66e−01 2.27e−01
JASTSIG 3 3 8.87e−01 6.58e−01
TOLLSIG 5 5 5.85e−01 4.95e−01
IMDMOD 6 6 8.64e−01 3.81e−01
JASTMOD 3 3 2.25e−01 2.57e−01
TOLLMOD 8 8 5.36e−01 2.95e−01
CASP 6 15 4.50e−02 7.62e−01
CLIPA 13 20 4.39e−01 7.67e−01
CLIPB 20 27 4.04e−01 8.14e−01
CLIPC 6 8 5.83e−02 1.04e−01
CLIPD 8 9 9.45e−01 6.13e−01
CLIPE 7 9 2.75e−01 9.83e−01
IAP 5 8 4.48e−01 6.33e−01
SRPN 16 18 5.63e−01 7.70e−01
AMP 8 9 5.58e−01 8.54e−01
LYS 1 7 8.46e−02 7.20e−03
PPO 1 9 9.34e−02 2.03e−02
GPX 2 2 9.21e−01 8.19e−01
HPX 10 15 6.18e−01 8.39e−01
TPX 5 5 3.64e−01 4.00e−01
SOD 4 4 7.30e−01 3.73e−01
APHAG 19 19 7.83e−02 1.52e−01
SRRP 23 28 6.83e−01 7.78e−01
SPZ 5 5 5.81e−01 2.93e−01
TOLL 6 12 8.04e−02 7.40e−01
Superfamily
ClasRec
OtheRec
PathSig
PathMod
CascMod
AntiMic
EffEnzy
AutoPha
RNAi
Cytokin
TOLL

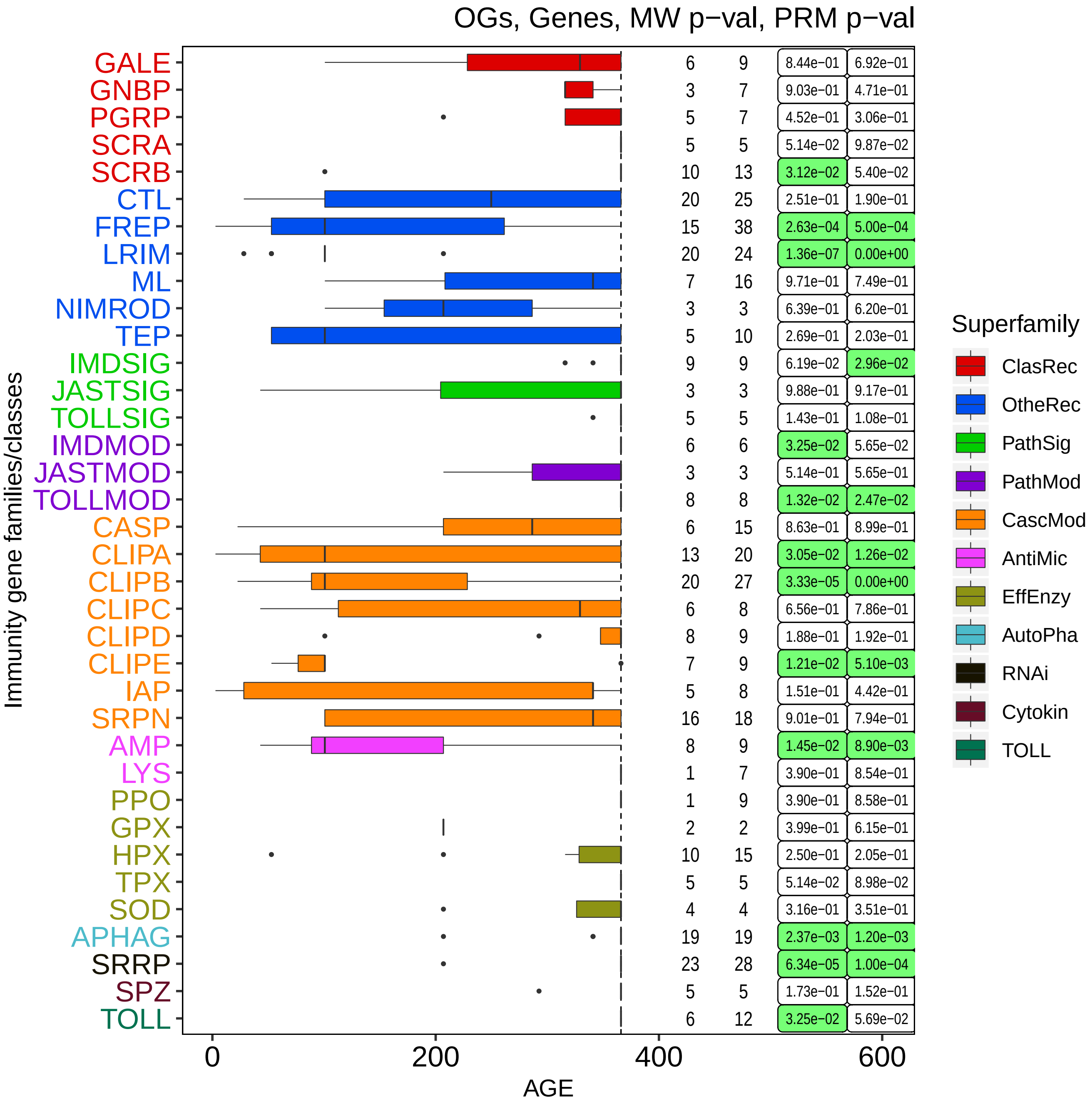

OGs, Genes, MW p−val, PRM p−val
Immunity gene families/classes
GALE 6 9 8.44e-01 6.92e-01
GNBP 3 7 9.03e-01 4.71e-01
PGRP 5 7 4.52e-01 3.06e-01
SCRA 5 5 5.14e-02 9.87e-02
SCRB 10 13 3.12e-02 5.40e-02
CTL 20 25 2.51e-01 1.90e-01
FREP 15 38 2.63e-04 5.00e-04
LRIM 20 24 1.36e-07 0.00e+00
ML 7 16 9.71e-01 7.49e-01
NIMROD 3 3 6.39e-01 6.20e-01
TEP 5 10 2.69e-01 2.03e-01
IMDSIG 9 9 6.19e-02 2.96e-02
JASTSIG 3 3 9.88e-01 9.17e-01
TOLLSIG 5 5 1.43e-01 1.08e-01
IMDMOD 6 6 3.25e-02 5.65e-02
JASTMOD 3 3 5.14e-01 5.65e-01
TOLLMOD 8 8 1.32e-02 2.47e-02
CASP 6 15 8.63e-01 8.99e-01
CLIPA 13 20 3.05e-02 1.26e-02
CLIPB 20 27 3.33e-05 0.00e+00
CLIPC 6 8 6.56e-01 7.86e-01
CLIPD 8 9 1.88e-01 1.92e-01
CLIPE 7 9 1.21e-02 5.10e-03
IAP 5 8 1.51e-01 4.42e-01
SRPN 16 18 9.01e-01 7.94e-01
AMP 8 9 1.45e-02 8.90e-03
LYS 1 7 3.90e-01 8.54e-01
PPO 1 9 3.90e-01 8.58e-01
GPX 2 2 3.99e-01 6.15e-01
HPX 10 15 2.50e-01 2.05e-01
TPX 5 5 5.14e-02 8.98e-02
SOD 4 4 3.16e-01 3.51e-01
APHAG 19 19 2.37e-03 1.20e-03
SRRP 23 28 6.34e-05 1.00e-04
SPZ 5 5 1.73e-01 1.52e-01
TOLL 6 12 3.25e-02 5.69e-02
0
200
400
600
AGE
Superfamily
ClasRec
OtheRec
PathSig
PathMod
CascMod
AntiMic
EffEnzy
AutoPha
RNAi
Cytokin
TOLL

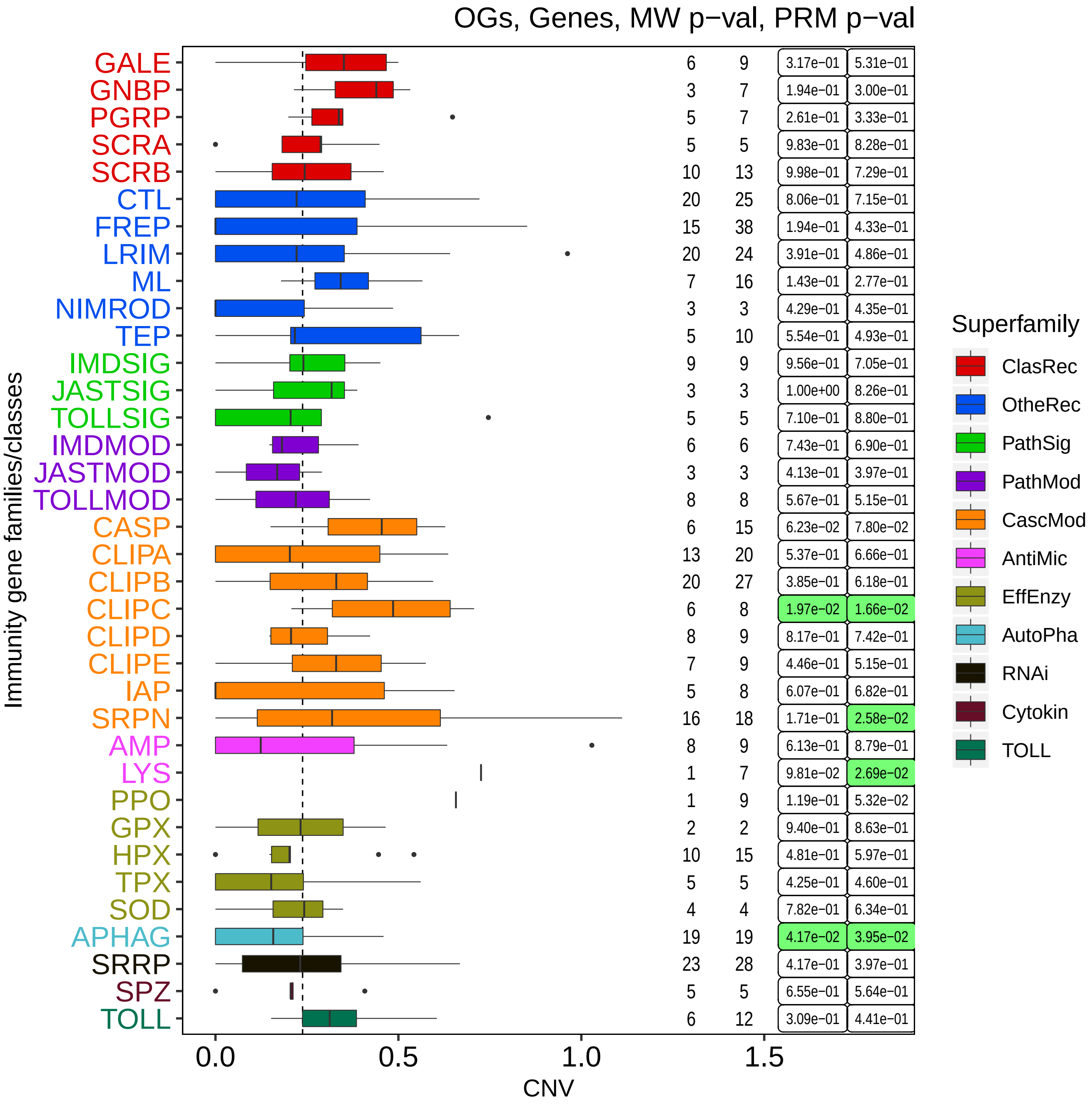

OGs, Genes, MW p−val, PRM p−val
Immunity gene families/classes
GALE 6 9 3.17e-01 5.31e-01
GNBP 3 7 1.94e-01 3.00e-01
PGRP 5 7 2.61e-01 3.33e-01
SCRA 5 5 9.83e-01 8.28e-01
SCRB 10 13 9.98e-01 7.29e-01
CTL 20 25 8.06e-01 7.15e-01
FREP 15 38 1.94e-01 4.33e-01
LRIM 20 24 3.91e-01 4.86e-01
ML 7 16 1.43e-01 2.77e-01
NIMROD 3 3 4.29e-01 4.35e-01
TEP 5 10 5.54e-01 4.93e-01
IMDSIG 9 9 9.56e-01 7.05e-01
JASTSIG 3 3 1.00e+00 8.26e-01
TOLLSIG 5 5 7.10e-01 8.80e-01
IMDMOD 6 6 7.43e-01 6.90e-01
JASTMOD 3 3 4.13e-01 3.97e-01
TOLLMOD 8 8 5.67e-01 5.15e-01
CASP 6 15 6.23e-02 7.80e-02
CLIPA 13 20 5.37e-01 6.66e-01
CLIPB 20 27 3.85e-01 6.18e-01
CLIPC 6 8 1.97e-02 1.66e-02
CLIPD 8 9 8.17e-01 7.42e-01
CLIPE 7 9 4.46e-01 5.15e-01
IAP 5 8 6.07e-01 6.82e-01
SRPN 16 18 1.71e-01 2.58e-02
AMP 8 9 6.13e-01 8.79e-01
LYS 1 7 9.81e-02 2.69e-02
PPO 1 9 1.19e-01 5.32e-02
GPX 2 2 9.40e-01 8.63e-01
HPX 10 15 4.81e-01 5.97e-01
TPX 5 5 4.25e-01 4.60e-01
SOD 4 4 7.82e-01 6.34e-01
APHAG 19 19 4.17e-02 3.95e-02
SRRP 23 28 4.17e-01 3.97e-01
SPZ 5 5 6.55e-01 5.64e-01
TOLL 6 12 3.09e-01 4.41e-01
0.0
0.5
1.0
1.5
CNV
Superfamily
ClasRec
OtheRec
PathSig
PathMod
CascMod
AntiMic
EffEnzy
AutoPha
RNAi
Cytokin
TOLL

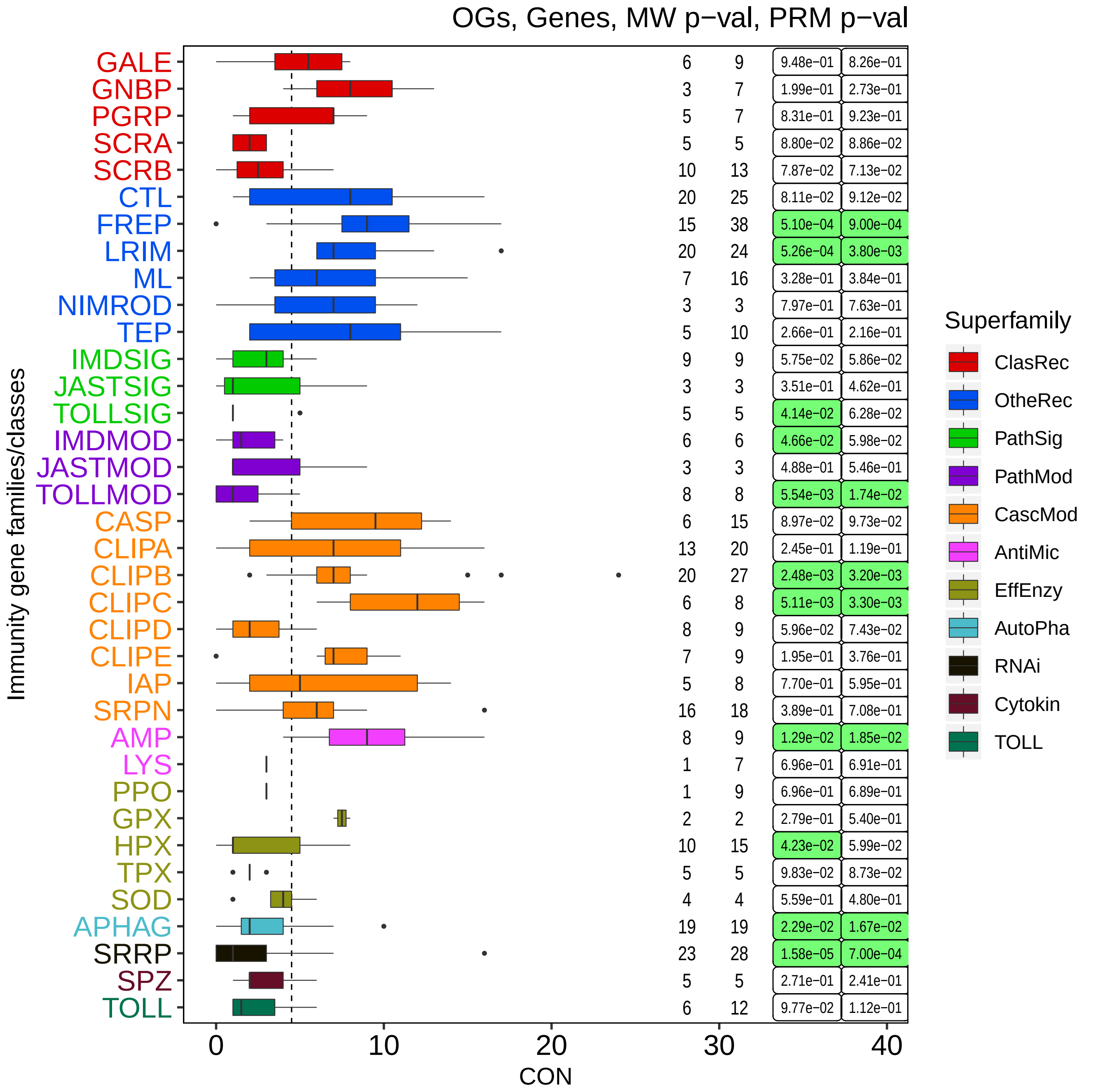

OGs, Genes, MW p−val, PRM p−val
GALE 6 9 9.48e-01 8.26e-01
GNBP 3 7 1.99e-01 2.73e-01
PGRP 5 7 8.31e-01 9.23e-01
SCRA 5 5 8.80e-02 8.86e-02
SCRB 10 13 7.87e-02 7.13e-02
CTL 20 25 8.11e-02 9.12e-02
FREP 15 38 5.10e-04 9.00e-04
LRIM 20 24 5.26e-04 3.80e-03
ML 7 16 3.28e-01 3.84e-01
NIMROD 3 3 7.97e-01 7.63e-01
TEP 5 10 2.66e-01 2.16e-01
IMDSIG 9 9 5.75e-02 5.86e-02
JASTSIG 3 3 3.51e-01 4.62e-01
TOLLSIG 5 5 4.14e-02 6.28e-02
IMDMOD 6 6 4.66e-02 5.98e-02
JASTMOD 3 3 4.88e-01 5.46e-01
TOLLMOD 8 8 5.54e-03 1.74e-02
CASP 6 15 8.97e-02 9.73e-02
CLIPA 13 20 2.45e-01 1.19e-01
CLIPB 20 27 2.48e-03 3.20e-03
CLIPC 6 8 5.11e-03 3.30e-03
CLIPD 8 9 5.96e-02 7.43e-02
CLIPE 7 9 1.95e-01 3.76e-01
IAP 5 8 7.70e-01 5.95e-01
SRPN 16 18 3.89e-01 7.08e-01
AMP 8 9 1.29e-02 1.85e-02
LYS 1 7 6.96e-01 6.91e-01
PPO 1 9 6.96e-01 6.89e-01
GPX 2 2 2.79e-01 5.40e-01
HPX 10 15 4.23e-02 5.99e-02
TPX 5 5 9.83e-02 8.73e-02
SOD 4 4 5.59e-01 4.80e-01
APHAG 19 19 2.29e-02 1.67e-02
SRRP 23 28 1.58e-05 7.00e-04
SPZ 5 5 2.71e-01 2.41e-01
TOLL 6 12 9.77e-02 1.12e-01
Immunity gene families/classes
0
10
20
30
40
CON
Superfamily
ClasRec
OtheRec
PathSig
PathMod
CascMod
AntiMic
EffEnzy
AutoPha
RNAi
Cytokin
TOLL

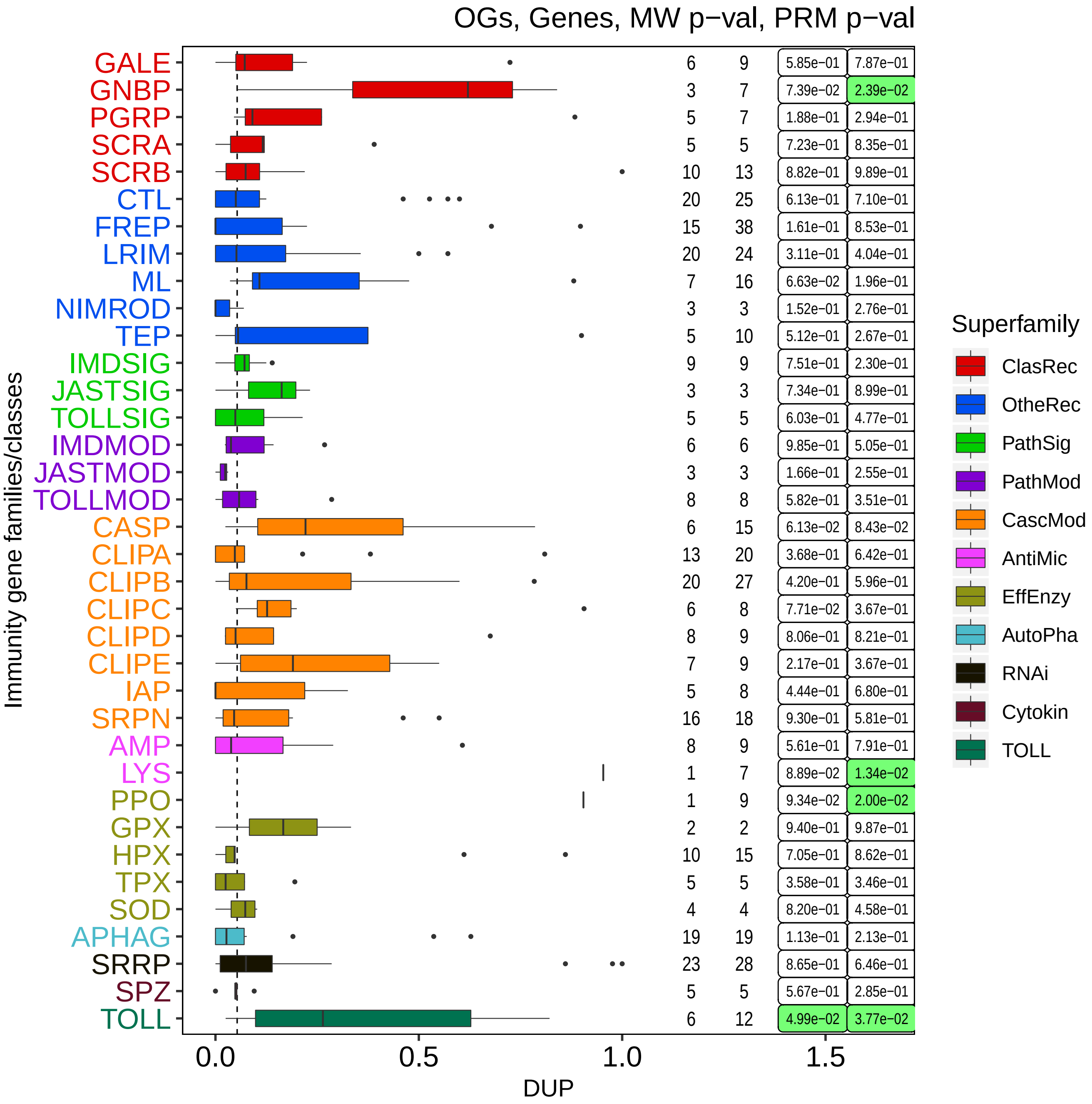
OGs, Genes, MW p−val, PRM p−val
GALE 6 9 5.85e−01 7.87e−01
GNBP 3 7 7.39e−02 2.39e−02
PGRP 5 7 1.88e−01 2.94e−01
SCRA 5 5 7.23e−01 8.35e−01
SCRB 10 13 8.82e−01 9.89e−01
CTL 20 25 6.13e−01 7.10e−01
FREP 15 38 1.61e−01 8.53e−01
LRIM 20 24 3.11e−01 4.04e−01
ML 7 16 6.63e−02 1.96e−01
NIMROD 3 3 1.52e−01 2.76e−01
TEP 5 10 5.12e−01 2.67e−01
IMDSIG 9 9 7.51e−01 2.30e−01
JASTSIG 3 3 7.34e−01 8.99e−01
TOLLSIG 5 5 6.03e−01 4.77e−01
IMDMOD 6 6 9.85e−01 5.05e−01
JASTMOD 3 3 1.66e−01 2.55e−01
TOLLMOD 8 8 5.82e−01 3.51e−01
CASP 6 15 6.13e−02 8.43e−02
CLIPA 13 20 3.68e−01 6.42e−01
CLIPB 20 27 4.20e−01 5.96e−01
CLIPC 6 8 7.71e−02 3.67e−01
CLIPD 8 9 8.06e−01 8.21e−01
CLIPE 7 9 2.17e−01 3.67e−01
IAP 5 8 4.44e−01 6.80e−01
SRPN 16 18 9.30e−01 5.81e−01
AMP 8 9 5.61e−01 7.91e−01
LYS 1 7 8.89e−02 1.34e−02
PPO 1 9 9.34e−02 2.00e−02
GPX 2 2 9.40e−01 9.87e−01
HPX 10 15 7.05e−01 8.62e−01
TPX 5 5 3.58e−01 3.46e−01
SOD 4 4 8.20e−01 4.58e−01
APHAG 19 19 1.13e−01 2.13e−01
SRRP 23 28 8.65e−01 6.46e−01
SPZ 5 5 5.67e−01 2.85e−01
TOLL 6 12 4.99e−02 3.77e−02
Immunity gene families/classes
0.0
0.5
1.0
1.5
DUP
Superfamily
ClasRec
OtheRec
PathSig
PathMod
CascMod
AntiMic
EffEnzy
AutoPha
RNAi
Cytokin
TOLL

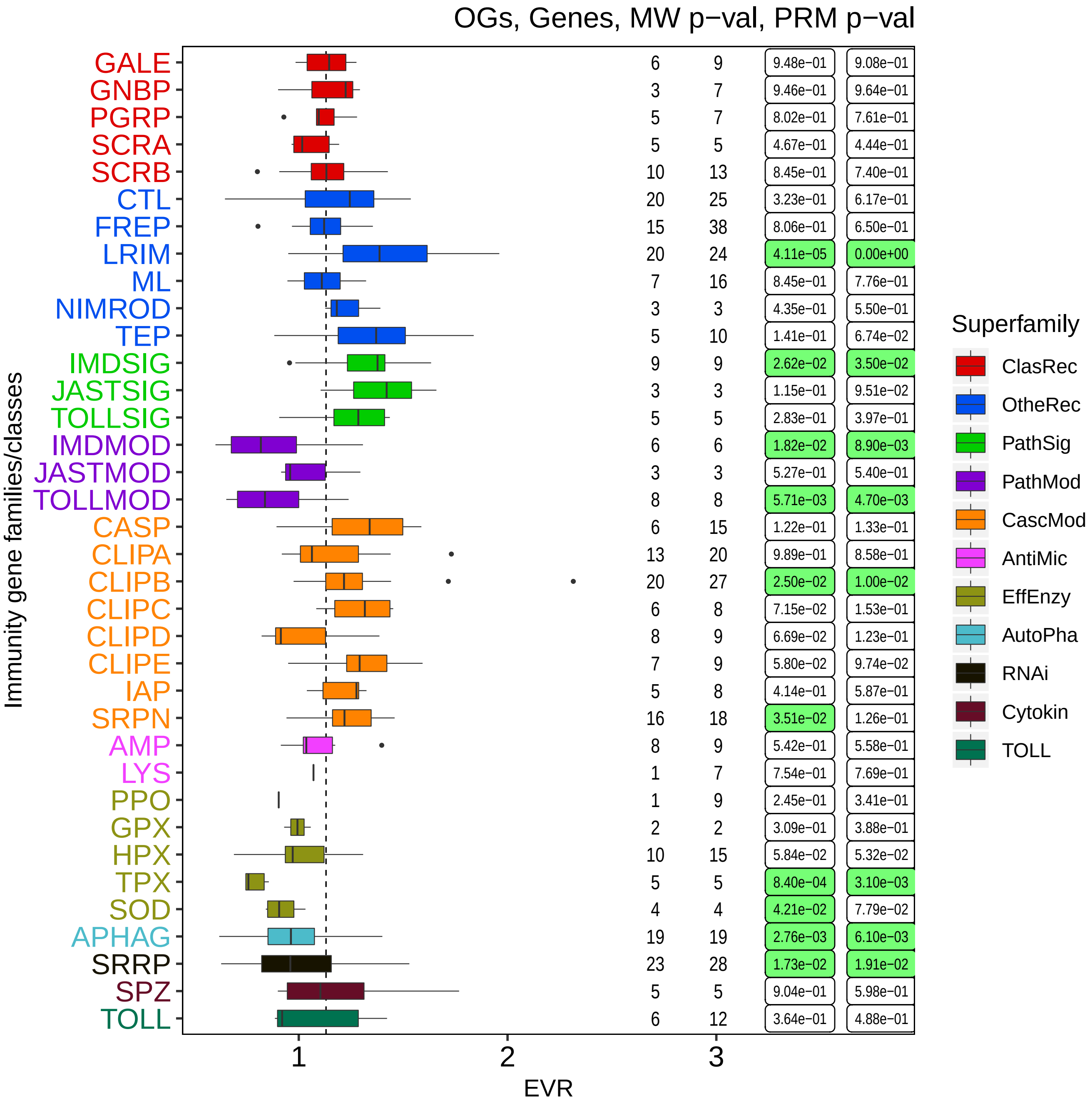
OGs, Genes, MW p−val, PRM p−val
Immunity gene families/classes
GALE 6 9 9.48e-01 9.08e-01
GNBP 3 7 9.46e-01 9.64e-01
PGRP 5 7 8.02e-01 7.61e-01
SCRA 5 5 4.67e-01 4.44e-01
SCRB 10 13 8.45e-01 7.40e-01
CTL 20 25 3.23e-01 6.17e-01
FREP 15 38 8.06e-01 6.50e-01
LRIM 20 24 4.11e-05 0.00e+00
ML 7 16 8.45e-01 7.76e-01
NIMROD 3 3 4.35e-01 5.50e-01
TEP 5 10 1.41e-01 6.74e-02
IMDSIG 9 9 2.62e-02 3.50e-02
JASTSIG 3 3 1.15e-01 9.51e-02
TOLLSIG 5 5 2.83e-01 3.97e-01
IMDMOD 6 6 1.82e-02 8.90e-03
JASTMOD 3 3 5.27e-01 5.40e-01
TOLLMOD 8 8 5.71e-03 4.70e-03
CASP 6 15 1.22e-01 1.33e-01
CLIPA 13 20 9.89e-01 8.58e-01
CLIPB 20 27 2.50e-02 1.00e-02
CLIPC 6 8 7.15e-02 1.53e-01
CLIPD 8 9 6.69e-02 1.23e-01
CLIPE 7 9 5.80e-02 9.74e-02
IAP 5 8 4.14e-01 5.87e-01
SRPN 16 18 3.51e-02 1.26e-01
AMP 8 9 5.42e-01 5.58e-01
LYS 1 7 7.54e-01 7.69e-01
PPO 1 9 2.45e-01 3.41e-01
GPX 2 2 3.09e-01 3.88e-01
HPX 10 15 5.84e-02 5.32e-02
TPX 5 5 8.40e-04 3.10e-03
SOD 4 4 4.21e-02 7.79e-02
APHAG 19 19 2.76e-03 6.10e-03
SRRP 23 28 1.73e-02 1.91e-02
SPZ 5 5 9.04e-01 5.98e-01
TOLL 6 12 3.64e-01 4.88e-01
1
2
3
EVR
Superfamily
ClasRec
OtheRec
PathSig
PathMod
CascMod
AntiMic
EffEnzy
AutoPha
RNAi
Cytokin
TOLL

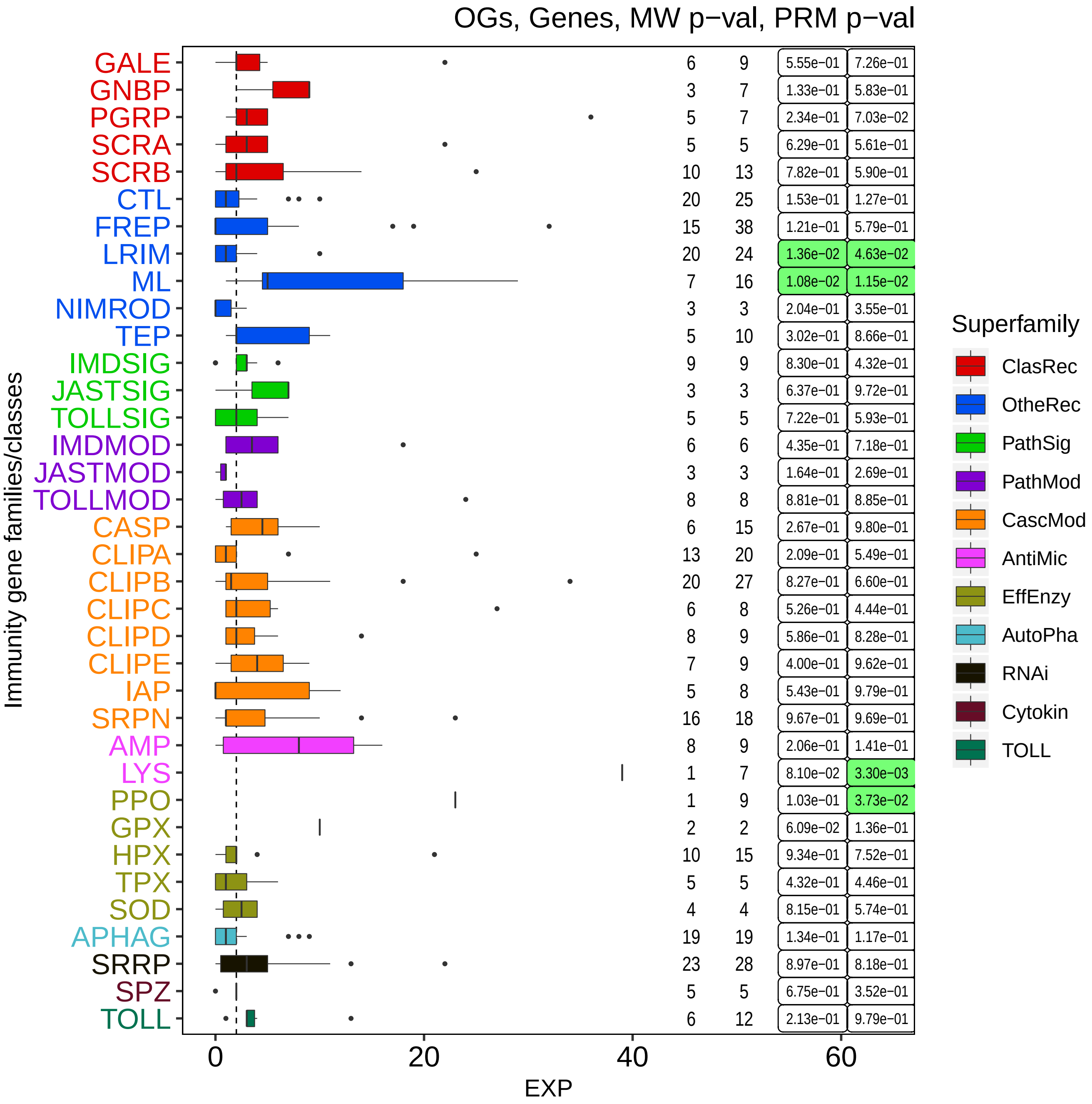
OGs, Genes, MW p−val, PRM p−val
Immunity gene families/classes
GALE 6 9 5.55e-01 7.26e-01
GNBP 3 7 1.33e-01 5.83e-01
PGRP 5 7 2.34e-01 7.03e-02
SCRA 5 5 6.29e-01 5.61e-01
SCRB 10 13 7.82e-01 5.90e-01
CTL 20 25 1.53e-01 1.27e-01
FREP 15 38 1.21e-01 5.79e-01
LRIM 20 24 1.36e-02 4.63e-02
ML 7 16 1.08e-02 1.15e-02
NIMROD 3 3 2.04e-01 3.55e-01
TEP 5 10 3.02e-01 8.66e-01
IMDSIG 9 9 8.30e-01 4.32e-01
JASTSIG 3 3 6.37e-01 9.72e-01
TOLLSIG 5 5 7.22e-01 5.93e-01
IMDMOD 6 6 4.35e-01 7.18e-01
JASTMOD 3 3 1.64e-01 2.69e-01
TOLLMOD 8 8 8.81e-01 8.85e-01
CASP 6 15 2.67e-01 9.80e-01
CLIPA 13 20 2.09e-01 5.49e-01
CLIPB 20 27 8.27e-01 6.60e-01
CLIPC 6 8 5.26e-01 4.44e-01
CLIPD 8 9 5.86e-01 8.28e-01
CLIPE 7 9 4.00e-01 9.62e-01
IAP 5 8 5.43e-01 9.79e-01
SRPN 16 18 9.67e-01 9.69e-01
AMP 8 9 2.06e-01 1.41e-01
LYS 1 7 8.10e-02 3.30e-03
PPO 1 9 1.03e-01 3.73e-02
GPX 2 2 6.09e-02 1.36e-01
HPX 10 15 9.34e-01 7.52e-01
TPX 5 5 4.32e-01 4.46e-01
SOD 4 4 8.15e-01 5.74e-01
APHAG 19 19 1.34e-01 1.17e-01
SRRP 23 28 8.97e-01 8.18e-01
SPZ 5 5 6.75e-01 3.52e-01
TOLL 6 12 2.13e-01 9.79e-01
0
20
40
60
EXP
Superfamily
ClasRec
OtheRec
PathSig
PathMod
CascMod
AntiMic
EffEnzy
AutoPha
RNAi
Cytokin
TOLL

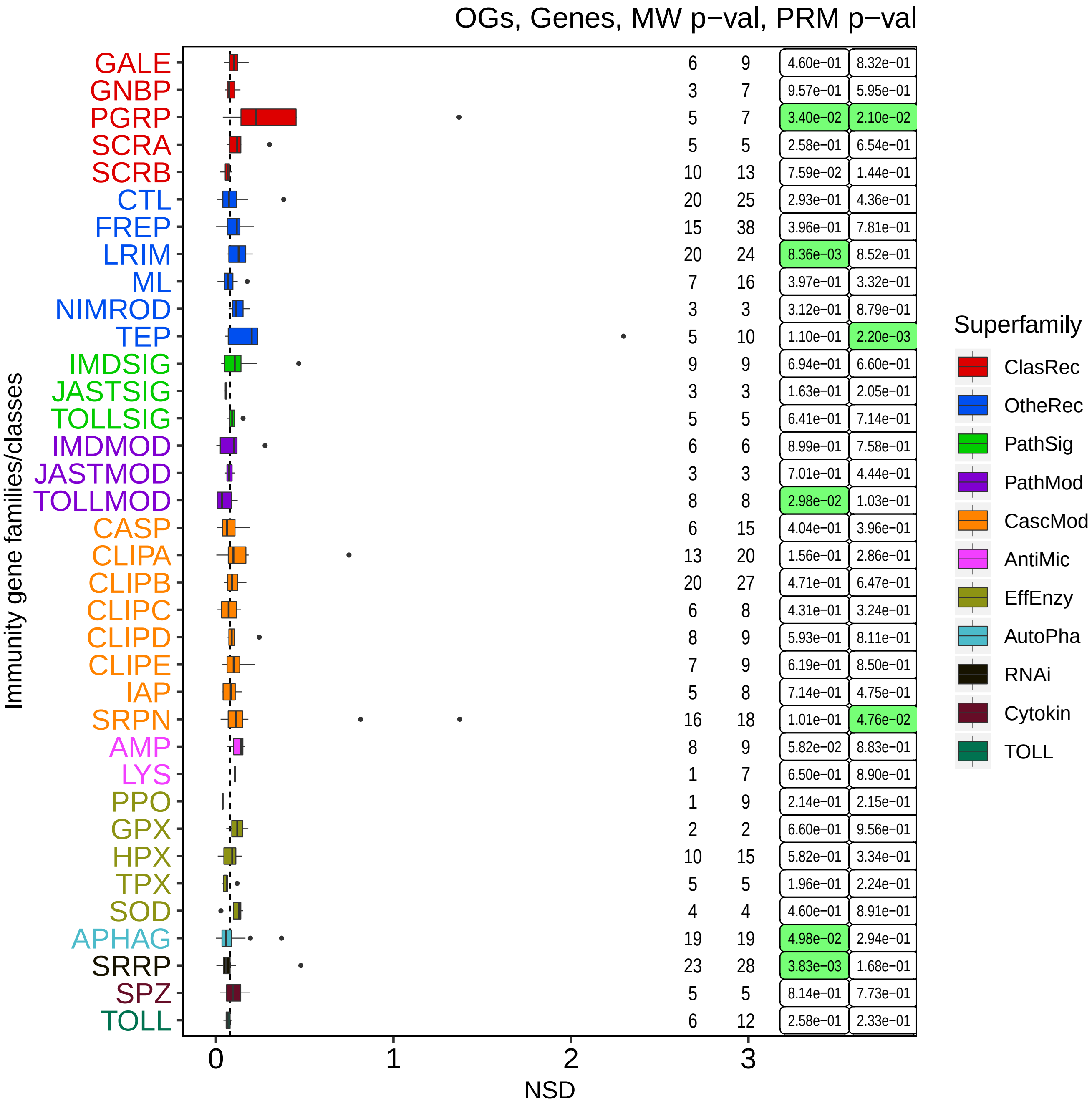

OGs, Genes, MW p−val, PRM p−val
Immunity gene families/classes
GALE 6 9 4.60e-01 8.32e-01
GNBP 3 7 9.57e-01 5.95e-01
PGRP 5 7 3.40e-02 2.10e-02
SCRA 5 5 2.58e-01 6.54e-01
SCRB 10 13 7.59e-02 1.44e-01
CTL 20 25 2.93e-01 4.36e-01
FREP 15 38 3.96e-01 7.81e-01
LRIM 20 24 8.36e-03 8.52e-01
ML 7 16 3.97e-01 3.32e-01
NIMROD 3 3 3.12e-01 8.79e-01
TEP 5 10 1.10e-01 2.20e-03
IMDSIG 9 9 6.94e-01 6.60e-01
JASTSIG 3 3 1.63e-01 2.05e-01
TOLLSIG 5 5 6.41e-01 7.14e-01
IMDMOD 6 6 8.99e-01 7.58e-01
JASTMOD 3 3 7.01e-01 4.44e-01
TOLLMOD 8 8 2.98e-02 1.03e-01
CASP 6 15 4.04e-01 3.96e-01
CLIPA 13 20 1.56e-01 2.86e-01
CLIPB 20 27 4.71e-01 6.47e-01
CLIPC 6 8 4.31e-01 3.24e-01
CLIPD 8 9 5.93e-01 8.11e-01
CLIPE 7 9 6.19e-01 8.50e-01
IAP 5 8 7.14e-01 4.75e-01
SRPN 16 18 1.01e-01 4.76e-02
AMP 8 9 5.82e-02 8.83e-01
LYS 1 7 6.50e-01 8.90e-01
PPO 1 9 2.14e-01 2.15e-01
GPX 2 2 6.60e-01 9.56e-01
HPX 10 15 5.82e-01 3.34e-01
TPX 5 5 1.96e-01 2.24e-01
SOD 4 4 4.60e-01 8.91e-01
APHAG 19 19 4.98e-02 2.94e-01
SRRP 23 28 3.83e-03 1.68e-01
SPZ 5 5 8.14e-01 7.73e-01
TOLL 6 12 2.58e-01 2.33e-01
0
1
2
3
NSD
Superfamily
ClasRec
OtheRec
PathSig
PathMod
CascMod
AntiMic
EffEnzy
AutoPha
RNAi
Cytokin
TOLL

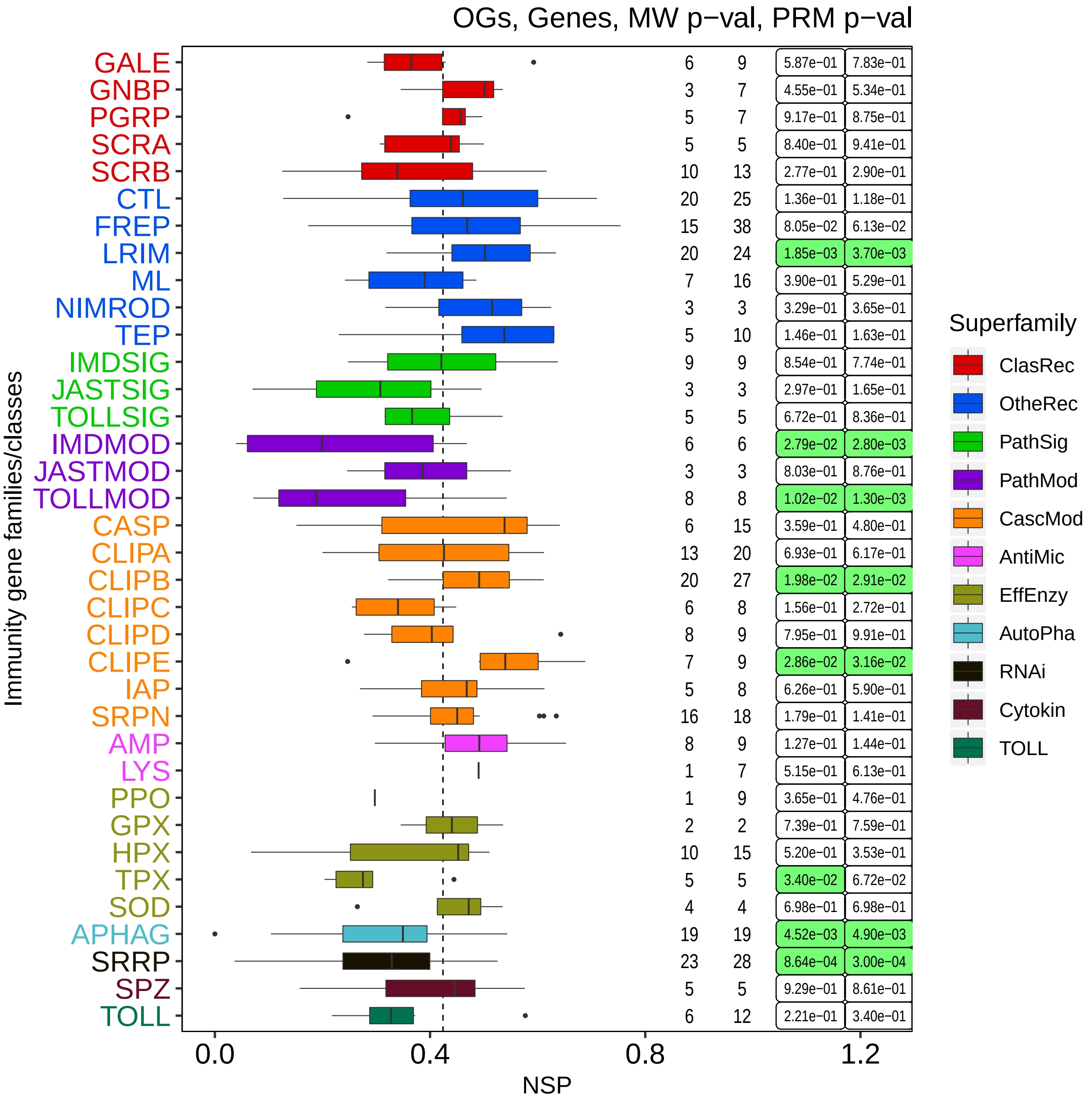

OGs, Genes, MW p−val, PRM p−val
GALE 6 9 5.87e−01 7.83e−01
GNBP 3 7 4.55e−01 5.34e−01
PGRP 5 7 9.17e−01 8.75e−01
SCRA 5 5 8.40e−01 9.41e−01
SCRB 10 13 2.77e−01 2.90e−01
CTL 20 25 1.36e−01 1.18e−01
FREP 15 38 8.05e−02 6.13e−02
LRIM 20 24 1.85e−03 3.70e−03
ML 7 16 3.90e−01 5.29e−01
NIMROD 3 3 3.29e−01 3.65e−01
TEP 5 10 1.46e−01 1.63e−01
IMDSIG 9 9 8.54e−01 7.74e−01
JASTSIG 3 3 2.97e−01 1.65e−01
TOLLSIG 5 5 6.72e−01 8.36e−01
IMDMOD 6 6 2.79e−02 2.80e−03
JASTMOD 3 3 8.03e−01 8.76e−01
TOLLMOD 8 8 1.02e−02 1.30e−03
CASP 6 15 3.59e−01 4.80e−01
CLIPA 13 20 6.93e−01 6.17e−01
CLIPB 20 27 1.98e−02 2.91e−02
CLIPC 6 8 1.56e−01 2.72e−01
CLIPD 8 9 7.95e−01 9.91e−01
CLIPE 7 9 2.86e−02 3.16e−02
IAP 5 8 6.26e−01 5.90e−01
SRPN 16 18 1.79e−01 1.41e−01
AMP 8 9 1.27e−01 1.44e−01
LYS 1 7 5.15e−01 6.13e−01
PPO 1 9 3.65e−01 4.76e−01
GPX 2 2 7.39e−01 7.59e−01
HPX 10 15 5.20e−01 3.53e−01
TPX 5 5 3.40e−02 6.72e−02
SOD 4 4 6.98e−01 6.98e−01
APHAG 19 19 4.52e−03 4.90e−03
SRRP 23 28 8.64e−04 3.00e−04
SPZ 5 5 9.29e−01 8.61e−01
TOLL 6 12 2.21e−01 3.40e−01
Immunity gene families/classes
0.0
0.4
0.8
1.2
NSP
Superfamily
ClasRec
OtheRec
PathSig
PathMod
CascMod
AntiMic
EffEnzy
AutoPha
RNAi
Cytokin
TOLL

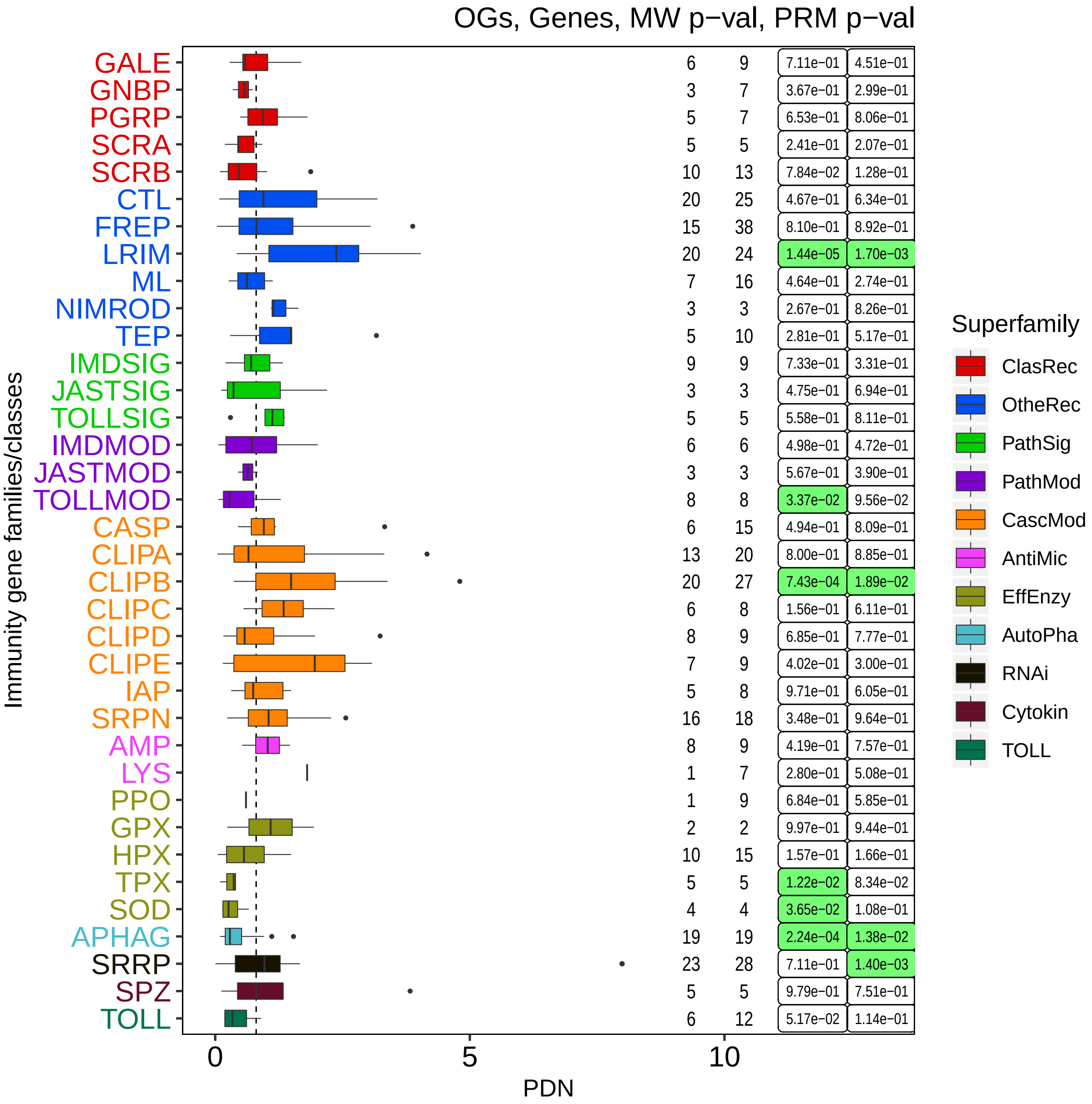
OGs, Genes, MW p−val, PRM p−val
GALE 6 9 7.11e-01 4.51e-01
GNBP 3 7 3.67e-01 2.99e-01
PGRP 5 7 6.53e-01 8.06e-01
SCRA 5 5 2.41e-01 2.07e-01
SCRB 10 13 7.84e-02 1.28e-01
CTL 20 25 4.67e-01 6.34e-01
FREP 15 38 8.10e-01 8.92e-01
LRIM 20 24 1.44e-05 1.70e-03
ML 7 16 4.64e-01 2.74e-01
NIMROD 3 3 2.67e-01 8.26e-01
TEP 5 10 2.81e-01 5.17e-01
IMDSIG 9 9 7.33e-01 3.31e-01
JASTSIG 3 3 4.75e-01 6.94e-01
TOLLSIG 5 5 5.58e-01 8.11e-01
IMDMOD 6 6 4.98e-01 4.72e-01
JASTMOD 3 3 5.67e-01 3.90e-01
TOLLMOD 8 8 3.37e-02 9.56e-02
CASP 6 15 4.94e-01 8.09e-01
CLIPA 13 20 8.00e-01 8.85e-01
CLIPB 20 27 7.43e-04 1.89e-02
CLIPC 6 8 1.56e-01 6.11e-01
CLIPD 8 9 6.85e-01 7.77e-01
CLIPE 7 9 4.02e-01 3.00e-01
IAP 5 8 9.71e-01 6.05e-01
SRPN 16 18 3.48e-01 9.64e-01
AMP 8 9 4.19e-01 7.57e-01
LYS 1 7 2.80e-01 5.08e-01
PPO 1 9 6.84e-01 5.85e-01
GPX 2 2 9.97e-01 9.44e-01
HPX 10 15 1.57e-01 1.66e-01
TPX 5 5 1.22e-02 8.34e-02
SOD 4 4 3.65e-02 1.08e-01
APHAG 19 19 2.24e-04 1.38e-02
SRRP 23 28 7.11e-01 1.40e-03
SPZ 5 5 9.79e-01 7.51e-01
TOLL 6 12 5.17e-02 1.14e-01
Immunity gene families/classes
0
5
10
PDN
Superfamily
ClasRec
OtheRec
PathSig
PathMod
CascMod
AntiMic
EffEnzy
AutoPha
RNAi
Cytokin
TOLL

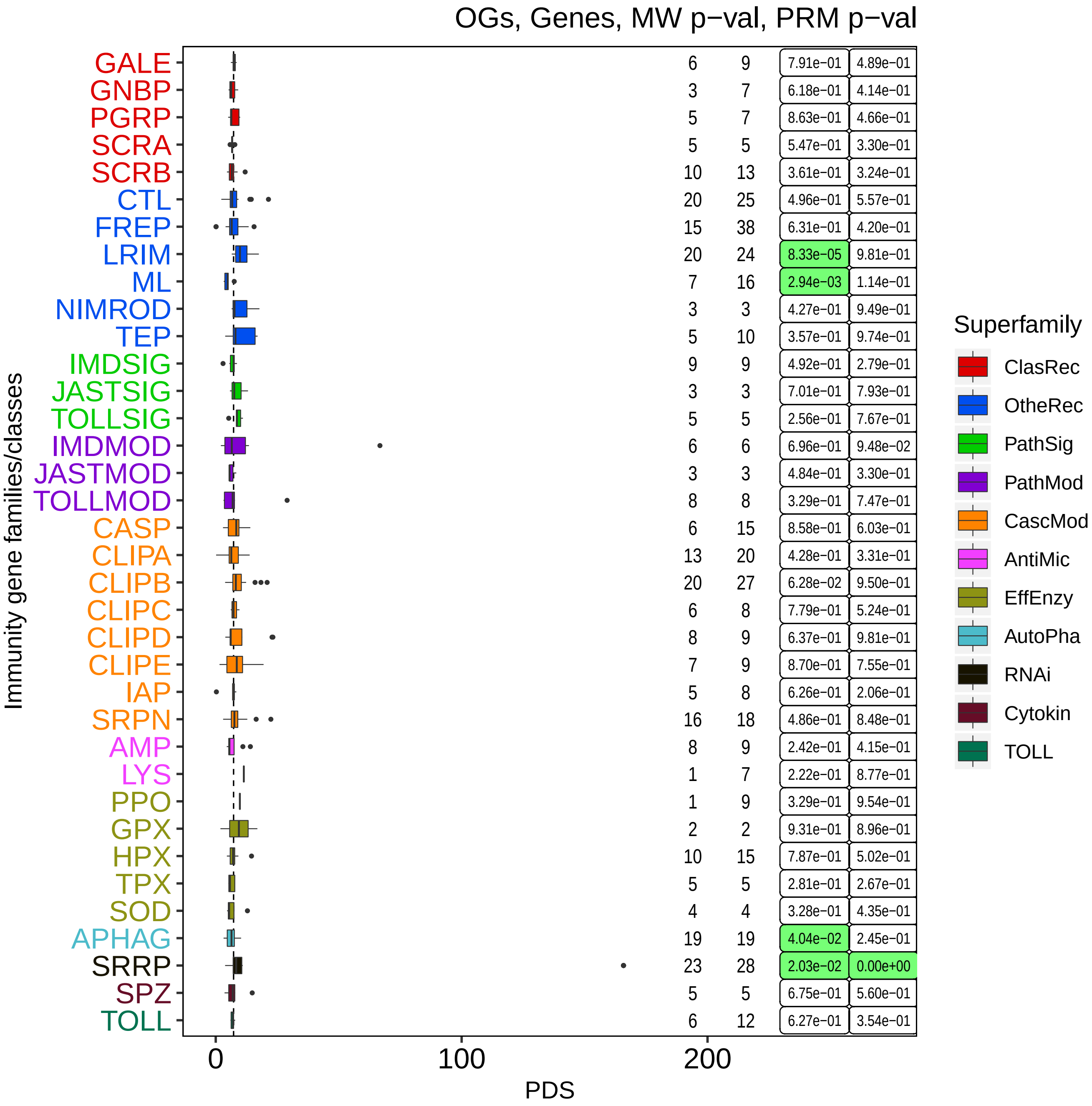

OGs, Genes, MW p−val, PRM p−val
Immunity gene families/classes
GALE 6 9 7.91e-01 4.89e-01
GNBP 3 7 6.18e-01 4.14e-01
PGRP 5 7 8.63e-01 4.66e-01
SCRA 5 5 5.47e-01 3.30e-01
SCRB 10 13 3.61e-01 3.24e-01
CTL 20 25 4.96e-01 5.57e-01
FREP 15 38 6.31e-01 4.20e-01
LRIM 20 24 8.33e-05 9.81e-01
ML 7 16 2.94e-03 1.14e-01
NIMROD 3 3 4.27e-01 9.49e-01
TEP 5 10 3.57e-01 9.74e-01
IMDSIG 9 9 4.92e-01 2.79e-01
JASTSIG 3 3 7.01e-01 7.93e-01
TOLLSIG 5 5 2.56e-01 7.67e-01
IMDMOD 6 6 6.96e-01 9.48e-02
JASTMOD 3 3 4.84e-01 3.30e-01
TOLLMOD 8 8 3.29e-01 7.47e-01
CASP 6 15 8.58e-01 6.03e-01
CLIPA 13 20 4.28e-01 3.31e-01
CLIPB 20 27 6.28e-02 9.50e-01
CLIPC 6 8 7.79e-01 5.24e-01
CLIPD 8 9 6.37e-01 9.81e-01
CLIPE 7 9 8.70e-01 7.55e-01
IAP 5 8 6.26e-01 2.06e-01
SRPN 16 18 4.86e-01 8.48e-01
AMP 8 9 2.42e-01 4.15e-01
LYS 1 7 2.22e-01 8.77e-01
PPO 1 9 3.29e-01 9.54e-01
GPX 2 2 9.31e-01 8.96e-01
HPX 10 15 7.87e-01 5.02e-01
TPX 5 5 2.81e-01 2.67e-01
SOD 4 4 3.28e-01 4.35e-01
APHAG 19 19 4.04e-02 2.45e-01
SRRP 23 28 2.03e-02 0.00e+00
SPZ 5 5 6.75e-01 5.60e-01
TOLL 6 12 6.27e-01 3.54e-01
0
100
200
PDS
Superfamily
ClasRec
OtheRec
PathSig
PathMod
CascMod
AntiMic
EffEnzy
AutoPha
RNAi
Cytokin
TOLL

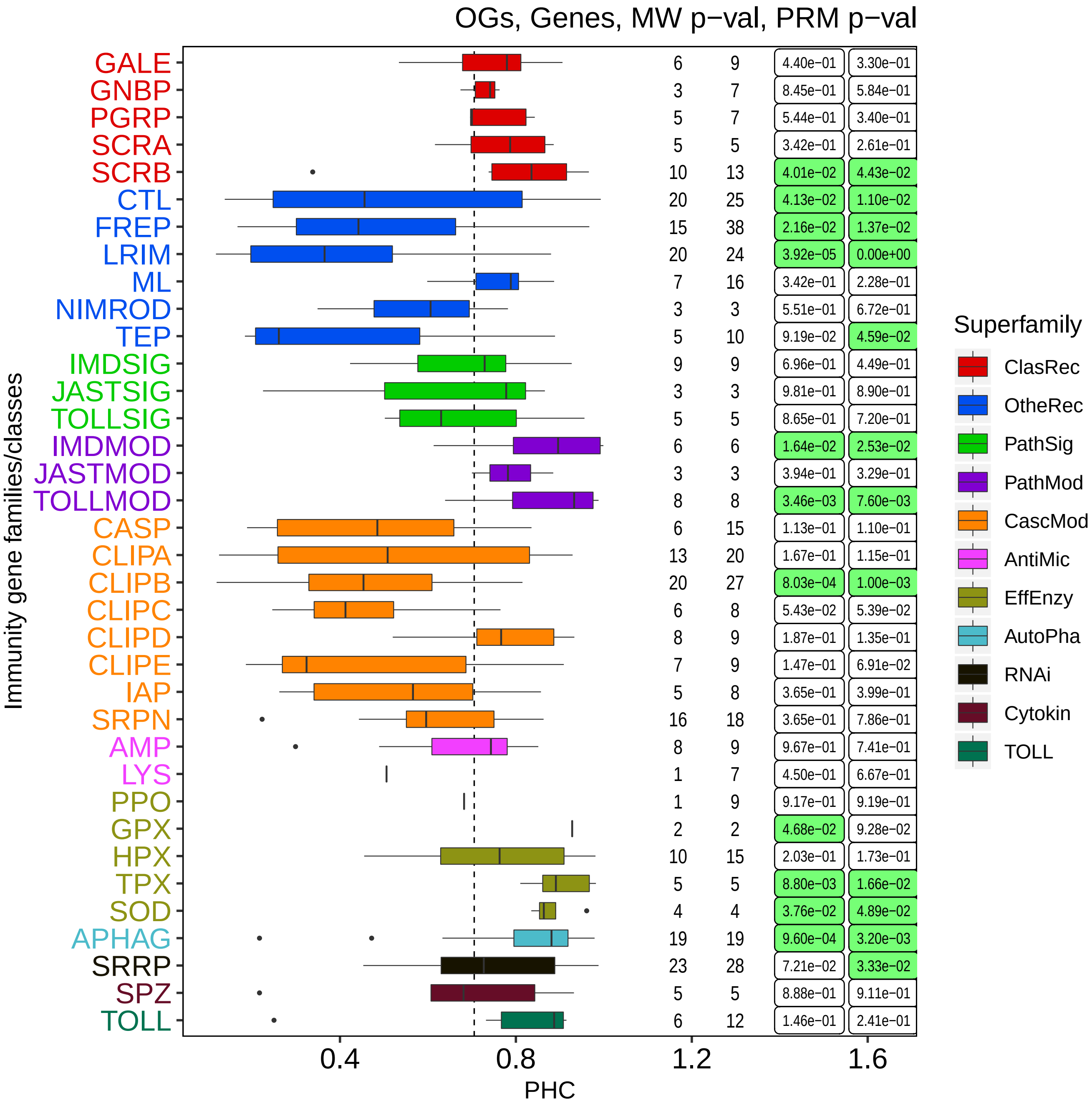
OGs, Genes, MW p−val, PRM p−val
GALE 6 9 4.40e−01 3.30e−01
GNBP 3 7 8.45e−01 5.84e−01
PGRP 5 7 5.44e−01 3.40e−01
SCRA 5 5 3.42e−01 2.61e−01
SCRB 10 13 4.01e−02 4.43e−02
CTL 20 25 4.13e−02 1.10e−02
FREP 15 38 2.16e−02 1.37e−02
LRIM 20 24 3.92e−05 0.00e+00
ML 7 16 3.42e−01 2.28e−01
NIMROD 3 3 5.51e−01 6.72e−01
TEP 5 10 9.19e−02 4.59e−02
IMDSIG 9 9 6.96e−01 4.49e−01
JASTSIG 3 3 9.81e−01 8.90e−01
TOLLSIG 5 5 8.65e−01 7.20e−01
IMDMOD 6 6 1.64e−02 2.53e−02
JASTMOD 3 3 3.94e−01 3.29e−01
TOLLMOD 8 8 3.46e−03 7.60e−03
CASP 6 15 1.13e−01 1.10e−01
CLIPA 13 20 1.67e−01 1.15e−01
CLIPB 20 27 8.03e−04 1.00e−03
CLIPC 6 8 5.43e−02 5.39e−02
CLIPD 8 9 1.87e−01 1.35e−01
CLIPE 7 9 1.47e−01 6.91e−02
IAP 5 8 3.65e−01 3.99e−01
SRPN 16 18 3.65e−01 7.86e−01
AMP 8 9 9.67e−01 7.41e−01
LYS 1 7 4.50e−01 6.67e−01
PPO 1 9 9.17e−01 9.19e−01
GPX 2 2 4.68e−02 9.28e−02
HPX 10 15 2.03e−01 1.73e−01
TPX 5 5 8.80e−03 1.66e−02
SOD 4 4 3.76e−02 4.89e−02
APHAG 19 19 9.60e−04 3.20e−03
SRRP 23 28 7.21e−02 3.33e−02
SPZ 5 5 8.88e−01 9.11e−01
TOLL 6 12 1.46e−01 2.41e−01
Immunity gene families/classes
0.4
0.8
1.2
1.6
PHC
Superfamily
ClasRec
OtheRec
PathSig
PathMod
CascMod
AntiMic
EffEnzy
AutoPha
RNAi
Cytokin
TOLL

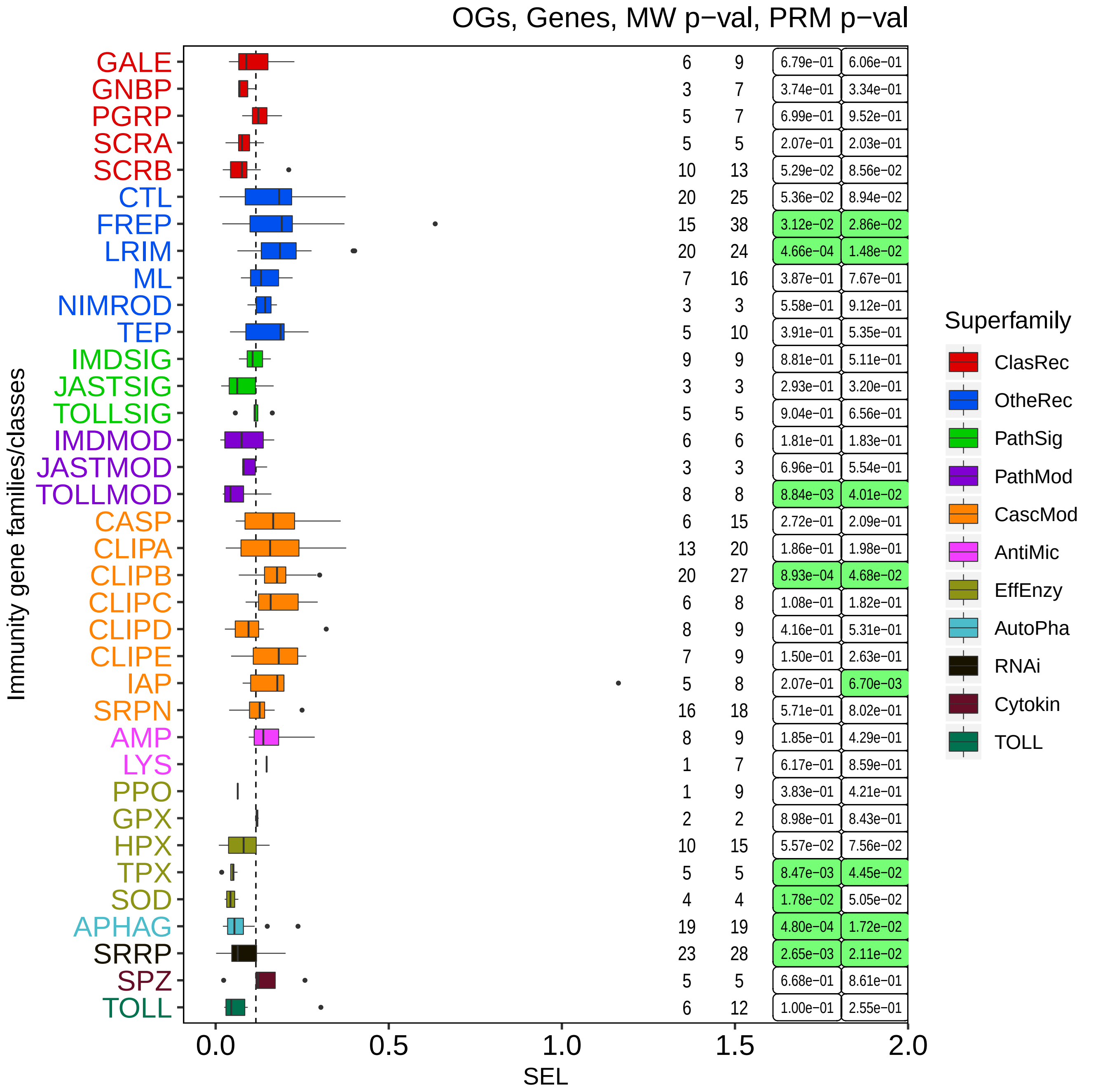
OGs, Genes, MW p−val, PRM p−val
Immunity gene families/classes
SEL
0.0
0.5
1.0
1.5
2.0
GALE 6 9 6.79e-01 6.06e-01
GNBP 3 7 3.74e-01 3.34e-01
PGRP 5 7 6.99e-01 9.52e-01
SCRA 5 5 2.07e-01 2.03e-01
SCRB 10 13 5.29e-02 8.56e-02
CTL 20 25 5.36e-02 8.94e-02
FREP 15 38 3.12e-02 2.86e-02
LRIM 20 24 4.66e-04 1.48e-02
ML 7 16 3.87e-01 7.67e-01
NIMROD 3 3 5.58e-01 9.12e-01
TEP 5 10 3.91e-01 5.35e-01
IMDSIG 9 9 8.81e-01 5.11e-01
JASTSIG 3 3 2.93e-01 3.20e-01
TOLLSIG 5 5 9.04e-01 6.56e-01
IMDMOD 6 6 1.81e-01 1.83e-01
JASTMOD 3 3 6.96e-01 5.54e-01
TOLLMOD 8 8 8.84e-03 4.01e-02
CASP 6 15 2.72e-01 2.09e-01
CLIPA 13 20 1.86e-01 1.98e-01
CLIPB 20 27 8.93e-04 4.68e-02
CLIPC 6 8 1.08e-01 1.82e-01
CLIPD 8 9 4.16e-01 5.31e-01
CLIPE 7 9 1.50e-01 2.63e-01
IAP 5 8 2.07e-01 6.70e-03
SRPN 16 18 5.71e-01 8.02e-01
AMP 8 9 1.85e-01 4.29e-01
LYS 1 7 6.17e-01 8.59e-01
PPO 1 9 3.83e-01 4.21e-01
GPX 2 2 8.98e-01 8.43e-01
HPX 10 15 5.57e-02 7.56e-02
TPX 5 5 8.47e-03 4.45e-02
SOD 4 4 1.78e-02 5.05e-02
APHAG 19 19 4.80e-04 1.72e-02
SRRP 23 28 2.65e-03 2.11e-02
SPZ 5 5 6.68e-01 8.61e-01
TOLL 6 12 1.00e-01 2.55e-01
Superfamily
ClasRec
OtheRec
PathSig
PathMod
CascMod
AntiMic
EffEnzy
AutoPha
RNAi
Cytokin
TOLL

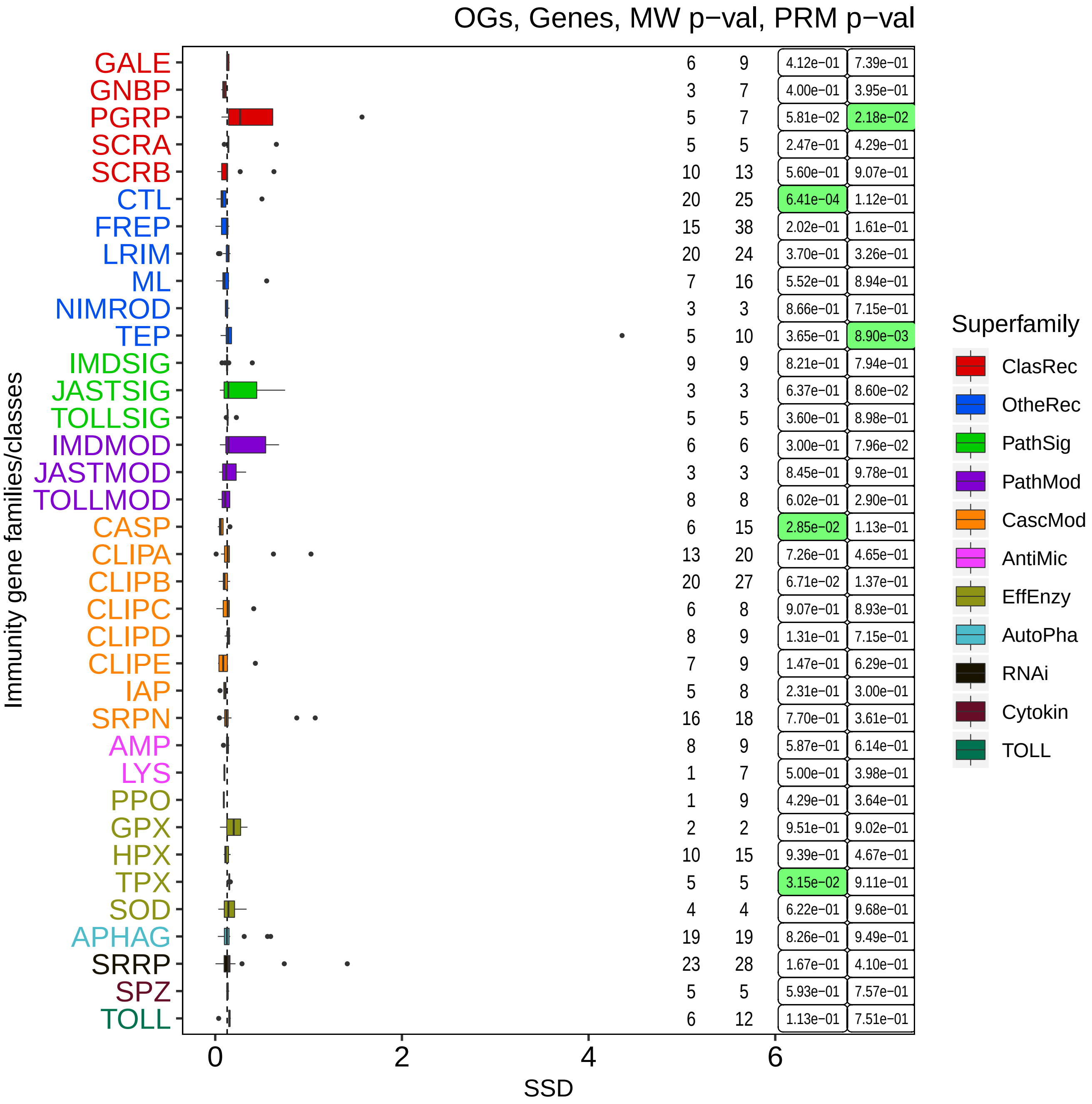

OGs, Genes, MW p−val, PRM p−val
GALE 6 9 4.12e-01 7.39e-01
GNBP 3 7 4.00e-01 3.95e-01
PGRP 5 7 5.81e-02 2.18e-02
SCRA 5 5 2.47e-01 4.29e-01
SCRB 10 13 5.60e-01 9.07e-01
CTL 20 25 6.41e-04 1.12e-01
FREP 15 38 2.02e-01 1.61e-01
LRIM 20 24 3.70e-01 3.26e-01
ML 7 16 5.52e-01 8.94e-01
NIMROD 3 3 8.66e-01 7.15e-01
TEP 5 10 3.65e-01 8.90e-03
IMDSIG 9 9 8.21e-01 7.94e-01
JASTSIG 3 3 6.37e-01 8.60e-02
TOLLSIG 5 5 3.60e-01 8.98e-01
IMDMOD 6 6 3.00e-01 7.96e-02
JASTMOD 3 3 8.45e-01 9.78e-01
TOLLMOD 8 8 6.02e-01 2.90e-01
CASP 6 15 2.85e-02 1.13e-01
CLIPA 13 20 7.26e-01 4.65e-01
CLIPB 20 27 6.71e-02 1.37e-01
CLIPC 6 8 9.07e-01 8.93e-01
CLIPD 8 9 1.31e-01 7.15e-01
CLIPE 7 9 1.47e-01 6.29e-01
IAP 5 8 2.31e-01 3.00e-01
SRPN 16 18 7.70e-01 3.61e-01
AMP 8 9 5.87e-01 6.14e-01
LYS 1 7 5.00e-01 3.98e-01
PPO 1 9 4.29e-01 3.64e-01
GPX 2 2 9.51e-01 9.02e-01
HPX 10 15 9.39e-01 4.67e-01
TPX 5 5 3.15e-02 9.11e-01
SOD 4 4 6.22e-01 9.68e-01
APHAG 19 19 8.26e-01 9.49e-01
SRRP 23 28 1.67e-01 4.10e-01
SPZ 5 5 5.93e-01 7.57e-01
TOLL 6 12 1.13e-01 7.51e-01
Immunity gene families/classes
0
2
4
6
SSD
Superfamily
ClasRec
OtheRec
PathSig
PathMod
CascMod
AntiMic
EffEnzy
AutoPha
RNAi
Cytokin
TOLL

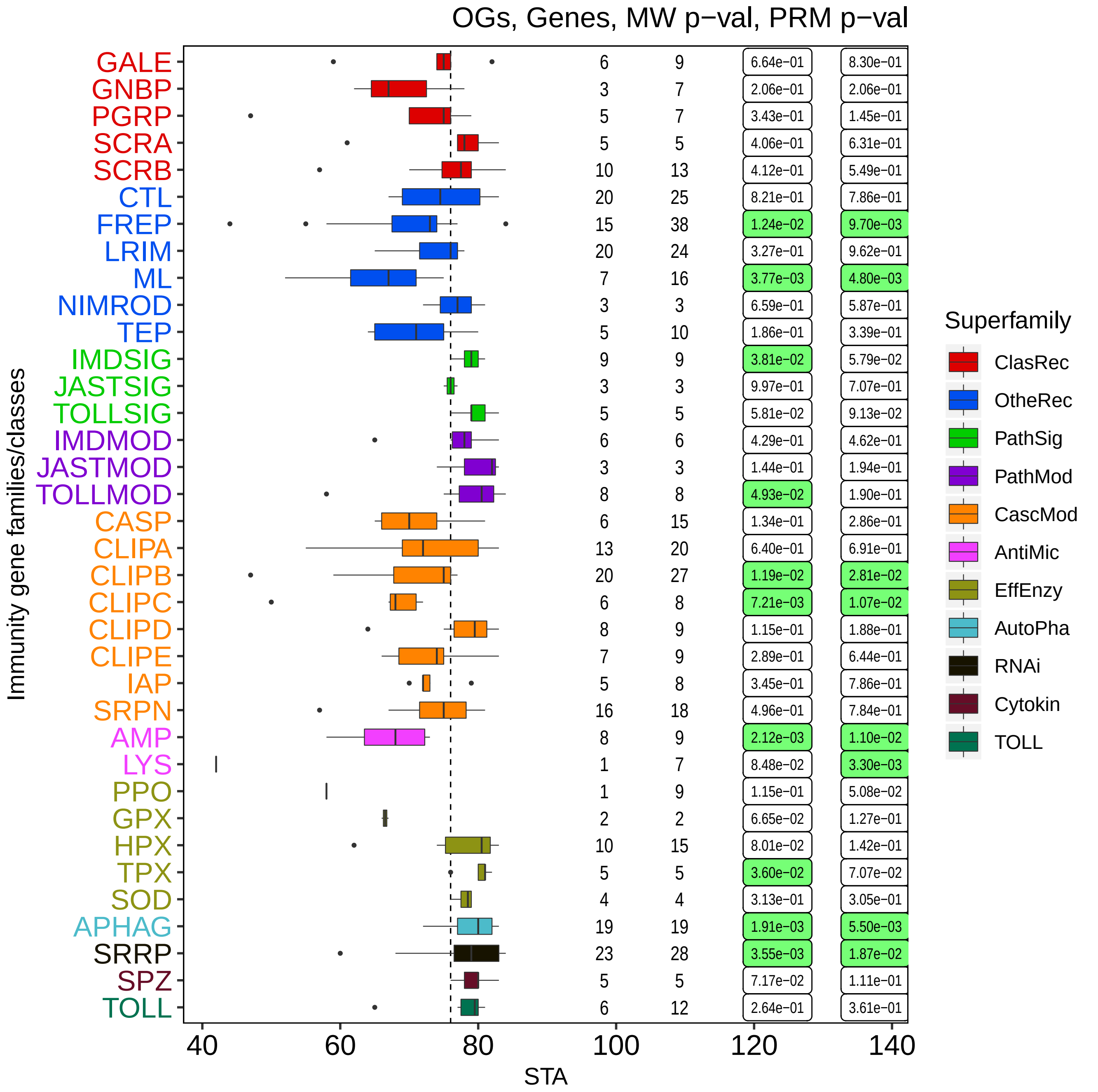
OGs, Genes, MW p−val, PRM p−val
Immunity gene families/classes
GALE 6 9 6.64e-01 8.30e-01
GNBP 3 7 2.06e-01 2.06e-01
PGRP 5 7 3.43e-01 1.45e-01
SCRA 5 5 4.06e-01 6.31e-01
SCRB 10 13 4.12e-01 5.49e-01
CTL 20 25 8.21e-01 7.86e-01
FREP 15 38 1.24e-02 9.70e-03
LRIM 20 24 3.27e-01 9.62e-01
ML 7 16 3.77e-03 4.80e-03
NIMROD 3 3 6.59e-01 5.87e-01
TEP 5 10 1.86e-01 3.39e-01
IMDSIG 9 9 3.81e-02 5.79e-02
JASTSIG 3 3 9.97e-01 7.07e-01
TOLLSIG 5 5 5.81e-02 9.13e-02
IMDMOD 6 6 4.29e-01 4.62e-01
JASTMOD 3 3 1.44e-01 1.94e-01
TOLLMOD 8 8 4.93e-02 1.90e-01
CASP 6 15 1.34e-01 2.86e-01
CLIPA 13 20 6.40e-01 6.91e-01
CLIPB 20 27 1.19e-02 2.81e-02
CLIPC 6 8 7.21e-03 1.07e-02
CLIPD 8 9 1.15e-01 1.88e-01
CLIPE 7 9 2.89e-01 6.44e-01
IAP 5 8 3.45e-01 7.86e-01
SRPN 16 18 4.96e-01 7.84e-01
AMP 8 9 2.12e-03 1.10e-02
LYS 1 7 8.48e-02 3.30e-03
PPO 1 9 1.15e-01 5.08e-02
GPX 2 2 6.65e-02 1.27e-01
HPX 10 15 8.01e-02 1.42e-01
TPX 5 5 3.60e-02 7.07e-02
SOD 4 4 3.13e-01 3.05e-01
APHAG 19 19 1.91e-03 5.50e-03
SRRP 23 28 3.55e-03 1.87e-02
SPZ 5 5 7.17e-02 1.11e-01
TOLL 6 12 2.64e-01 3.61e-01
40
60
80
100
120
140
STA
Superfamily
ClasRec
OtheRec
PathSig
PathMod
CascMod
AntiMic
EffEnzy
AutoPha
RNAi
Cytokin
TOLL

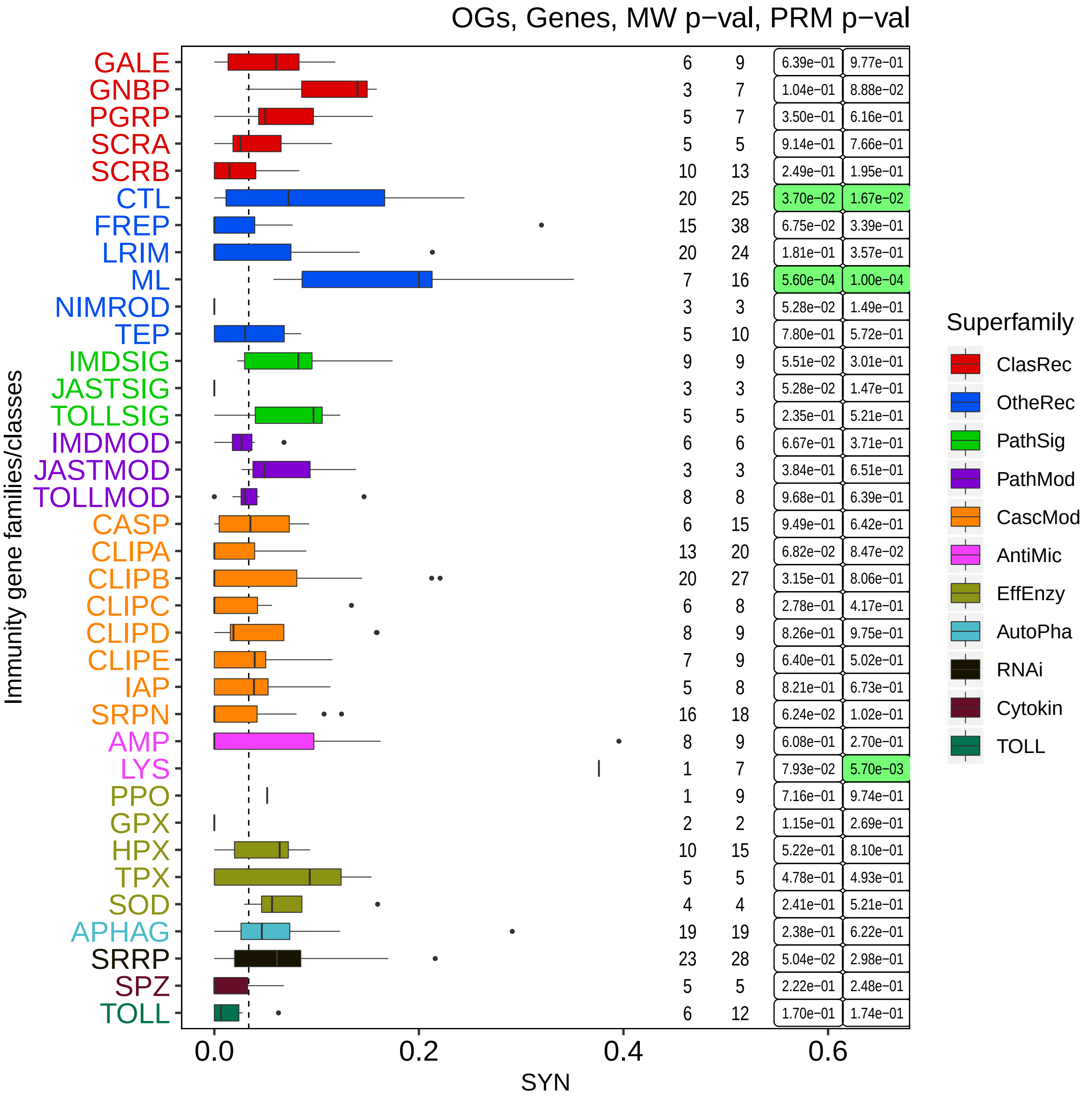

OGs, Genes, MW p−val, PRM p−val
Immunity gene families/classes
GALE 6 9 6.39e-01 9.77e-01
GNBP 3 7 1.04e-01 8.88e-02
PGRP 5 7 3.50e-01 6.16e-01
SCRA 5 5 9.14e-01 7.66e-01
SCRB 10 13 2.49e-01 1.95e-01
CTL 20 25 3.70e-02 1.67e-02
FREP 15 38 6.75e-02 3.39e-01
LRIM 20 24 1.81e-01 3.57e-01
ML 7 16 5.60e-04 1.00e-04
NIMROD 3 3 5.28e-02 1.49e-01
TEP 5 10 7.80e-01 5.72e-01
IMDSIG 9 9 5.51e-02 3.01e-01
JASTSIG 3 3 5.28e-02 1.47e-01
TOLLSIG 5 5 2.35e-01 5.21e-01
IMDMOD 6 6 6.67e-01 3.71e-01
JASTMOD 3 3 3.84e-01 6.51e-01
TOLLMOD 8 8 9.68e-01 6.39e-01
CASP 6 15 9.49e-01 6.42e-01
CLIPA 13 20 6.82e-02 8.47e-02
CLIPB 20 27 3.15e-01 8.06e-01
CLIPC 6 8 2.78e-01 4.17e-01
CLIPD 8 9 8.26e-01 9.75e-01
CLIPE 7 9 6.40e-01 5.02e-01
IAP 5 8 8.21e-01 6.73e-01
SRPN 16 18 6.24e-02 1.02e-01
AMP 8 9 6.08e-01 2.70e-01
LYS 1 7 7.93e-02 5.70e-03
PPO 1 9 7.16e-01 9.74e-01
GPX 2 2 1.15e-01 2.69e-01
HPX 10 15 5.22e-01 8.10e-01
TPX 5 5 4.78e-01 4.93e-01
SOD 4 4 2.41e-01 5.21e-01
APHAG 19 19 2.38e-01 6.22e-01
SRRP 23 28 5.04e-02 2.98e-01
SPZ 5 5 2.22e-01 2.48e-01
TOLL 6 12 1.70e-01 1.74e-01
0.0
0.2
0.4
0.6
SYN
Superfamily
ClasRec
OtheRec
PathSig
PathMod
CascMod
AntiMic
EffEnzy
AutoPha
RNAi
Cytokin
TOLL

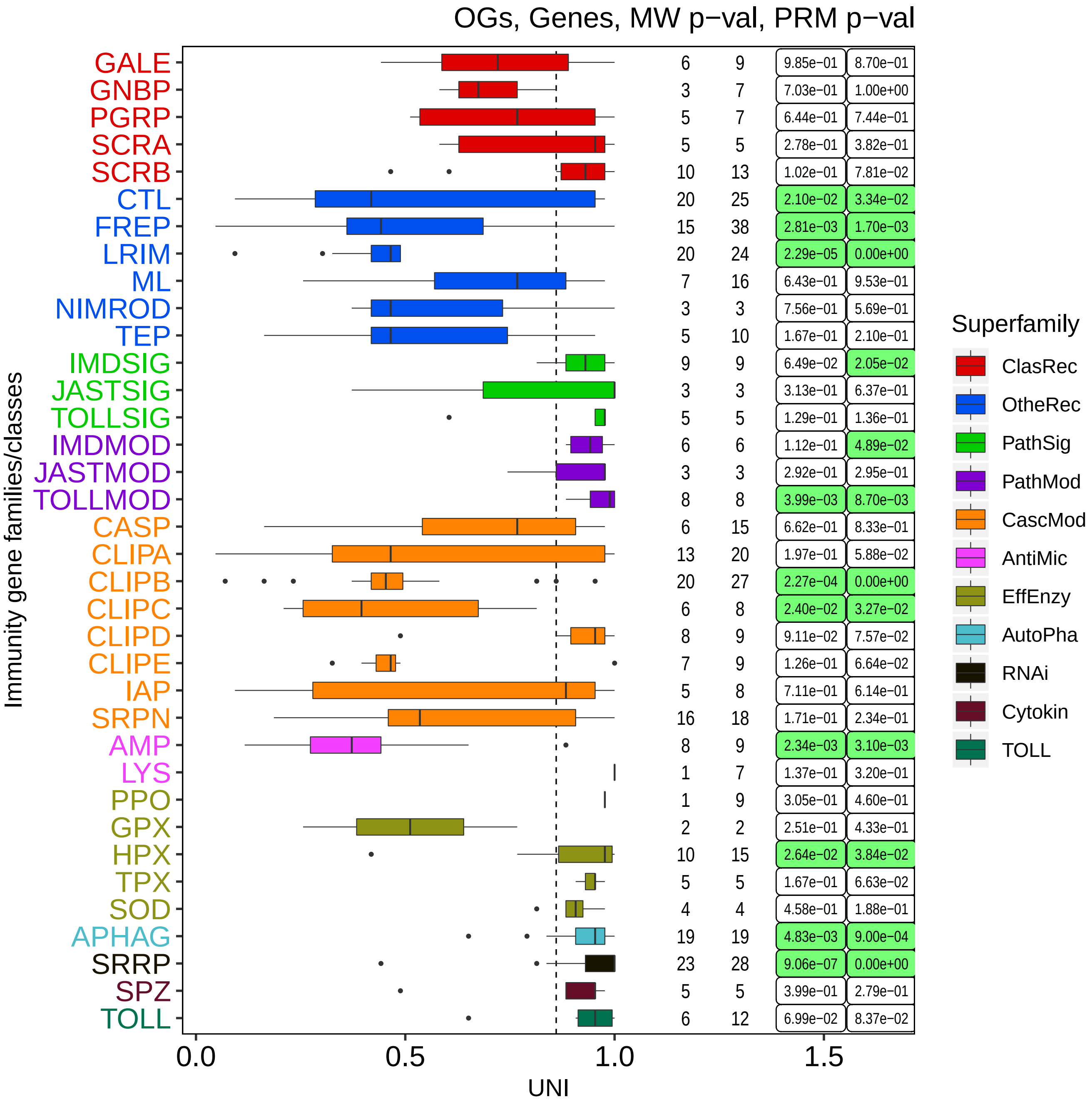
OGs, Genes, MW p−val, PRM p−val
Immunity gene families/classes
GALE 6 9 9.85e−01 8.70e−01
GNBP 3 7 7.03e−01 1.00e+00
PGRP 5 7 6.44e−01 7.44e−01
SCRA 5 5 2.78e−01 3.82e−01
SCRB 10 13 1.02e−01 7.81e−02
CTL 20 25 2.10e−02 3.34e−02
FREP 15 38 2.81e−03 1.70e−03
LRIM 20 24 2.29e−05 0.00e+00
ML 7 16 6.43e−01 9.53e−01
NIMROD 3 3 7.56e−01 5.69e−01
TEP 5 10 1.67e−01 2.10e−01
IMDSIG 9 9 6.49e−02 2.05e−02
JASTSIG 3 3 3.13e−01 6.37e−01
TOLLSIG 5 5 1.29e−01 1.36e−01
IMDMOD 6 6 1.12e−01 4.89e−02
JASTMOD 3 3 2.92e−01 2.95e−01
TOLLMOD 8 8 3.99e−03 8.70e−03
CASP 6 15 6.62e−01 8.33e−01
CLIPA 13 20 1.97e−01 5.88e−02
CLIPB 20 27 2.27e−04 0.00e+00
CLIPC 6 8 2.40e−02 3.27e−02
CLIPD 8 9 9.11e−02 7.57e−02
CLIPE 7 9 1.26e−01 6.64e−02
IAP 5 8 7.11e−01 6.14e−01
SRPN 16 18 1.71e−01 2.34e−01
AMP 8 9 2.34e−03 3.10e−03
LYS 1 7 1.37e−01 3.20e−01
PPO 1 9 3.05e−01 4.60e−01
GPX 2 2 2.51e−01 4.33e−01
HPX 10 15 2.64e−02 3.84e−02
TPX 5 5 1.67e−01 6.63e−02
SOD 4 4 4.58e−01 1.88e−01
APHAG 19 19 4.83e−03 9.00e−04
SRRP 23 28 9.06e−07 0.00e+00
SPZ 5 5 3.99e−01 2.79e−01
TOLL 6 12 6.99e−02 8.37e−02
0.0
0.5
1.0
1.5
UNI
Superfamily
ClasRec
OtheRec
PathSig
PathMod
CascMod
AntiMic
EffEnzy
AutoPha
RNAi
Cytokin
TOLL

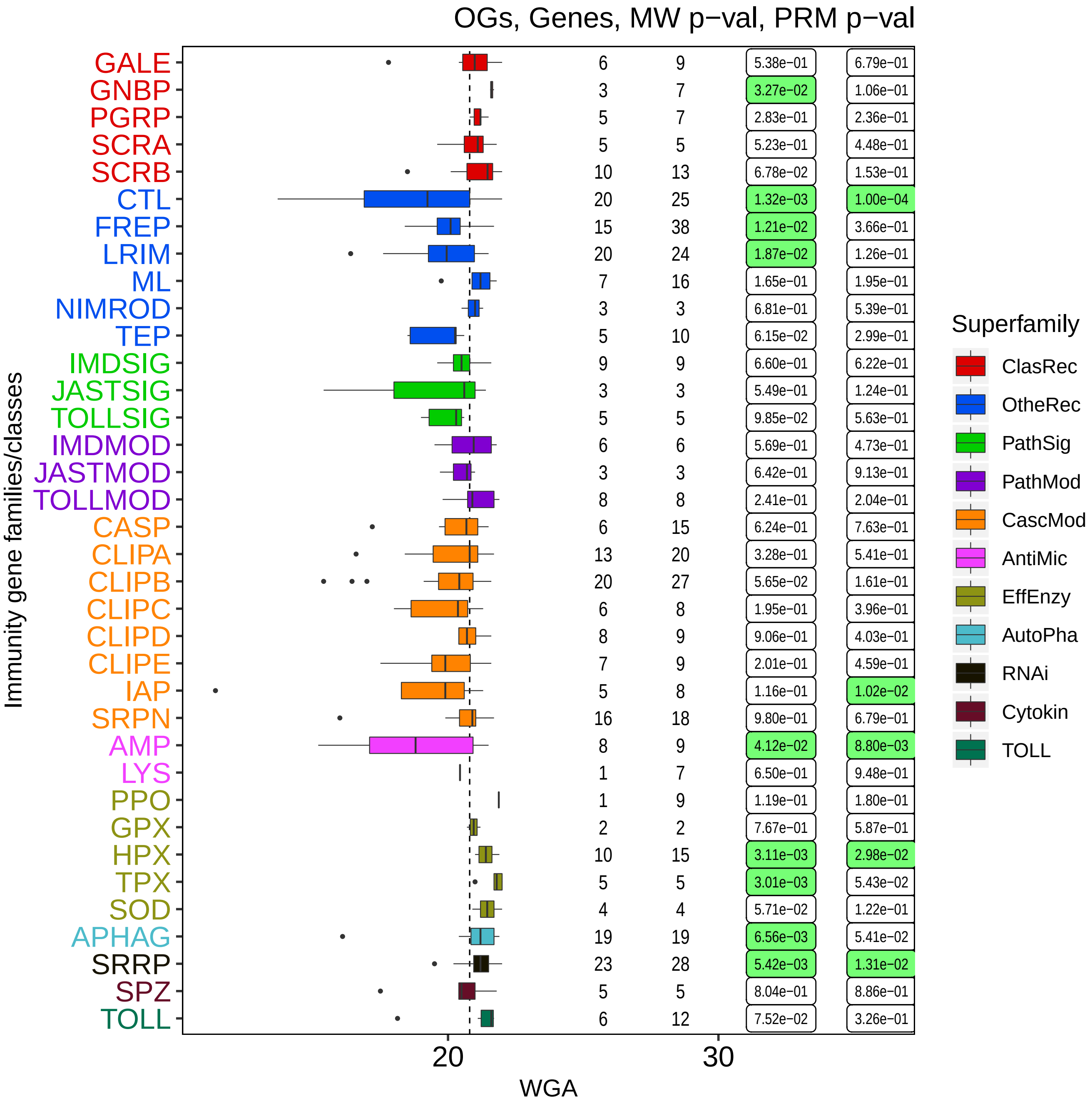
OGs, Genes, MW p−val, PRM p−val
Immunity gene families/classes
GALE 6 9 5.38e-01 6.79e-01
GNBP 3 7 3.27e-02 1.06e-01
PGRP 5 7 2.83e-01 2.36e-01
SCRA 5 5 5.23e-01 4.48e-01
SCRB 10 13 6.78e-02 1.53e-01
CTL 20 25 1.32e-03 1.00e-04
FREP 15 38 1.21e-02 3.66e-01
LRIM 20 24 1.87e-02 1.26e-01
ML 7 16 1.65e-01 1.95e-01
NIMROD 3 3 6.81e-01 5.39e-01
TEP 5 10 6.15e-02 2.99e-01
IMDSIG 9 9 6.60e-01 6.22e-01
JASTSIG 3 3 5.49e-01 1.24e-01
TOLLSIG 5 5 9.85e-02 5.63e-01
IMDMOD 6 6 5.69e-01 4.73e-01
JASTMOD 3 3 6.42e-01 9.13e-01
TOLLMOD 8 8 2.41e-01 2.04e-01
CASP 6 15 6.24e-01 7.63e-01
CLIPA 13 20 3.28e-01 5.41e-01
CLIPB 20 27 5.65e-02 1.61e-01
CLIPC 6 8 1.95e-01 3.96e-01
CLIPD 8 9 9.06e-01 4.03e-01
CLIPE 7 9 2.01e-01 4.59e-01
IAP 5 8 1.16e-01 1.02e-02
SRPN 16 18 9.80e-01 6.79e-01
AMP 8 9 4.12e-02 8.80e-03
LYS 1 7 6.50e-01 9.48e-01
PPO 1 9 1.19e-01 1.80e-01
GPX 2 2 7.67e-01 5.87e-01
HPX 10 15 3.11e-03 2.98e-02
TPX 5 5 3.01e-03 5.43e-02
SOD 4 4 5.71e-02 1.22e-01
APHAG 19 19 6.56e-03 5.41e-02
SRRP 23 28 5.42e-03 1.31e-02
SPZ 5 5 8.04e-01 8.86e-01
TOLL 6 12 7.52e-02 3.26e-01
20
30
WGA
Superfamily
ClasRec
OtheRec
PathSig
PathMod
CascMod
AntiMic
EffEnzy
AutoPha
RNAi
Cytokin
TOLL
