# Supplementary material for: Functional Constraints on Insect Immune System Components Govern Their Evolutionary Trajectories
Source: Mol Biol Evol. 2021 Dec 10;39(1):msab352. doi: 10.1093/molbev/msab352 (PMC8788225; doi:10.1093/molbev/msab352)
Supplement: msab352_Supplementary_Data [file msab352_supplementary_data.zip › Additional_File_3_expression-distributions-percluster-BFG-4.pdf]

**Additional File 3**

Visualisations of the 129 expression modules (deepSplit=4) built using the weighted correlation network analysis (WGCNA) approach with 24 selected conditions (see supplementary materials for details). Expression patterns per cluster are visualised as boxplots (showing medians, 25th and 75th percentiles, lower and upper whiskers [1.5 x interquartile range], and outliers) and coreplots (showing the medians as a dotted line with coloured areas bounded by the ranges of the 25th and 75th percentiles), with the order of the conditions determined by hierarchical clustering with hclust. Tables show Biological Process and Molecular Function Gene Ontology terms for terms with at least two occurrences in the module and with p-values according to the classic Fisher or the weight01 Fisher tests of p<0.01 (see supplementary materials for details).

| **Short Name** | **Condition** |
|---|---|
| Blood15d | Blood meal time series (Marinotti et al., 2006) blood-fed 15d |
| Blood24h | Blood meal time series (Marinotti et al., 2006) blood-fed 24h |
| Blood3h | Blood meal time series (Marinotti et al., 2006) blood-fed 3h |
| Blood48h | Blood meal time series (Marinotti et al., 2006) blood_fed 48h |
| Blood72h | Blood meal time series (Marinotti et al., 2006) blood_fed 72h |
| Blood96h | Blood meal time series (Marinotti et al., 2006) blood-fed 96h |
| BloodNBF | Blood meal time series (Marinotti et al., 2006) Non-blood-fed |
| BloodNBF18d | Blood meal after 15 days (Marinotti et al., 2006) Non-blood-fed 18d |
| BloodTwo24h | Two consecutive blood meals (Marinotti et al., 2006) blood-fed 24h |
| BloodTwoNBF | Two consecutive blood meals (Marinotti et al., 2006) Non-blood-fed |
| FatBodyBF | Blood-fed adult female tissues (Marinotti et al., 2006) fat body |
| GutAnt | Alimentary canal compartments (Neira Oviedo et al., 2008) anterior midgut |
| GutCaeca | Alimentary canal compartments (Neira Oviedo et al., 2008) gastric caeca |
| GutHind | Alimentary canal compartments (Neira Oviedo et al., 2008) hindgut |
| GutMidBF | Blood-fed adult female tissues (Marinotti et al., 2006) midgut |
| GutMidF | Adult tissues (Baker et al., 2011) midgut:female |
| GutMidM | Adult tissues (Baker et al., 2011) midgut:male |
| GutPost | Alimentary canal compartments (Neira Oviedo et al., 2008) posterior midgut |
| SalivF | Adult tissues (Baker et al., 2011) salivary gland:female |
| SalivM | Adult tissues (Baker et al., 2011) salivary gland:male |
| WholeF | Adult tissues (Baker et al., 2011) whole body:female |
| WholeM | Adult tissues (Baker et al., 2011) whole body:male |
| CarcassF | Adult tissues (Baker et al., 2011) carcass:female |
| CarcassM | Adult tissues (Baker et al., 2011) carcass:male |

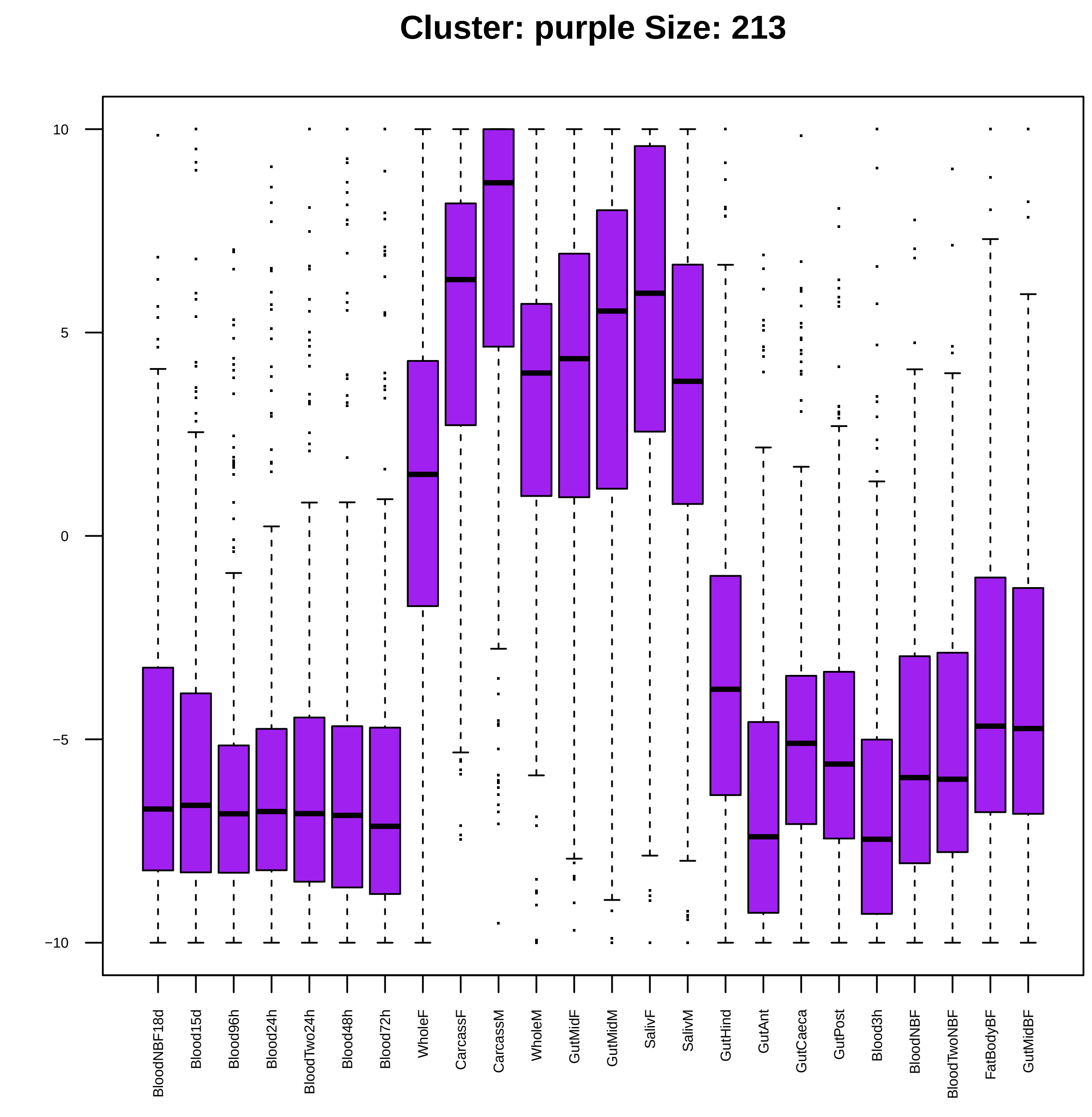

Cluster: purple Size: 213
10
5
0
−5
−10
BloodNBF18d
Blood15d
Blood96h
Blood24h
BloodTwo24h
Blood48h
Blood72h
WholeF
CarcassF
CarcassM
WholeM
GutMidF
GutMidM
SalivF
SalivM
GutHind
GutAnt
GutCaeca
GutPost
Blood3h
BloodNBF
BloodTwoNBF
FatBodyBF
GutMidBF

| | GO.ID | BPCluster: purple Size: 213 | Annotated | Significant | Expected | Rank in ClassicF | Weight01F | ClassicF |
|---|---|---|---|---|---|---|---|---|
| *1* | GO:0048871 | multicellular organismal homeostasis | 19 | 4 | 0.40 | 8 | 0.00043 | 0.00058 |
| *2* | GO:0030203 | glycosaminoglycan metabolic process | 18 | 4 | 0.38 | 7 | 0.00047 | 0.00047 |
| *3* | GO:0006906 | vesicle fusion | 22 | 5 | 0.47 | 4 | 0.00130 | 7.8e−05 |
| *4* | GO:0008333 | endosome to lysosome transport | 11 | 3 | 0.23 | 13 | 0.00135 | 0.00135 |
| *5* | GO:0006026 | aminoglycan catabolic process | 33 | 4 | 0.70 | 23 | 0.00488 | 0.00488 |
| *6* | GO:0051606 | detection of stimulus | 121 | 3 | 2.56 | 758 | 0.00644 | 0.47472 |
| *7* | GO:0006886 | intracellular protein transport | 167 | 9 | 3.54 | 33 | 0.00752 | 0.00870 |
| *8* | GO:0016236 | macroautophagy | 21 | 3 | 0.44 | 35 | 0.00934 | 0.00934 |
| *20* | GO:0006955 | immune response | 95 | 7 | 2.01 | 22 | 0.02440 | 0.00383 |
| *29* | GO:0031347 | regulation of defense response | 40 | 6 | 0.85 | 5 | 0.04060 | 0.00017 |

| | GO.ID | MFCluster: purple Size: 213 | Annotated | Significant | Expected | Rank in ClassicF | Weight01F | ClassicF |
|---|---|---|---|---|---|---|---|---|
| *2* | GO:0016811 | hydrolase activity, acting on carbon−nit... | 32 | 4 | 0.63 | 2 | 0.0034 | 0.0034 |
| *3* | GO:0003924 | GTPase activity | 116 | 7 | 2.28 | 3 | 0.0078 | 0.0078 |

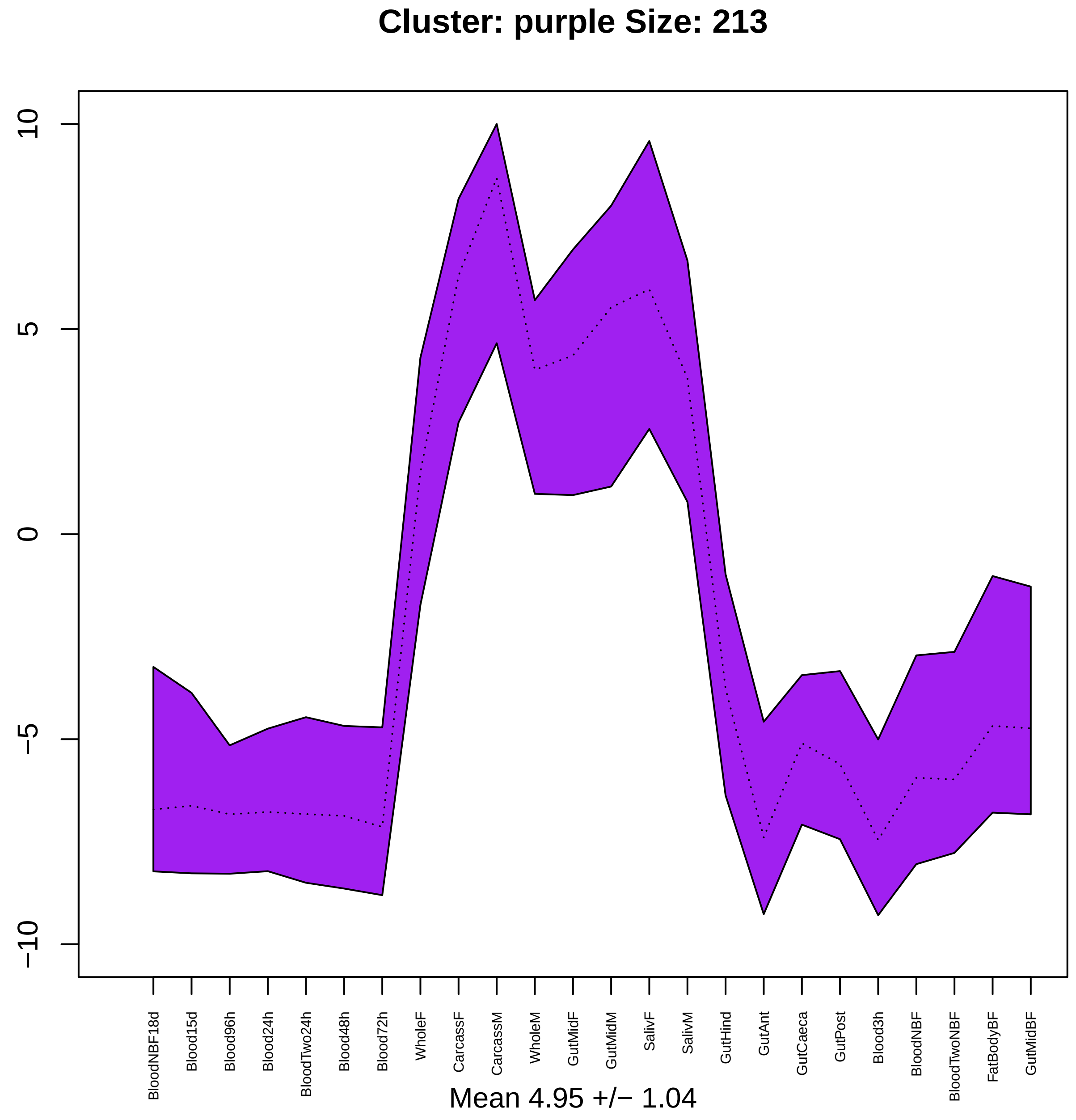

Cluster: purple Size: 213
10
5
0
−5
−10
BloodNBF18d
Blood15d
Blood96h
Blood24h
BloodTwo24h
Blood48h
Blood72h
WholeF
CarcassF
CarcassM
WholeM
GutMidF
GutMidM
SalivF
SalivM
GutHind
GutAnt
GutCaeca
GutPost
Blood3h
BloodNBF
BloodTwoNBF
FatBodyBF
GutMidBF
Mean 4.95 +/− 1.04

# Cluster: salmon4 Size: 39

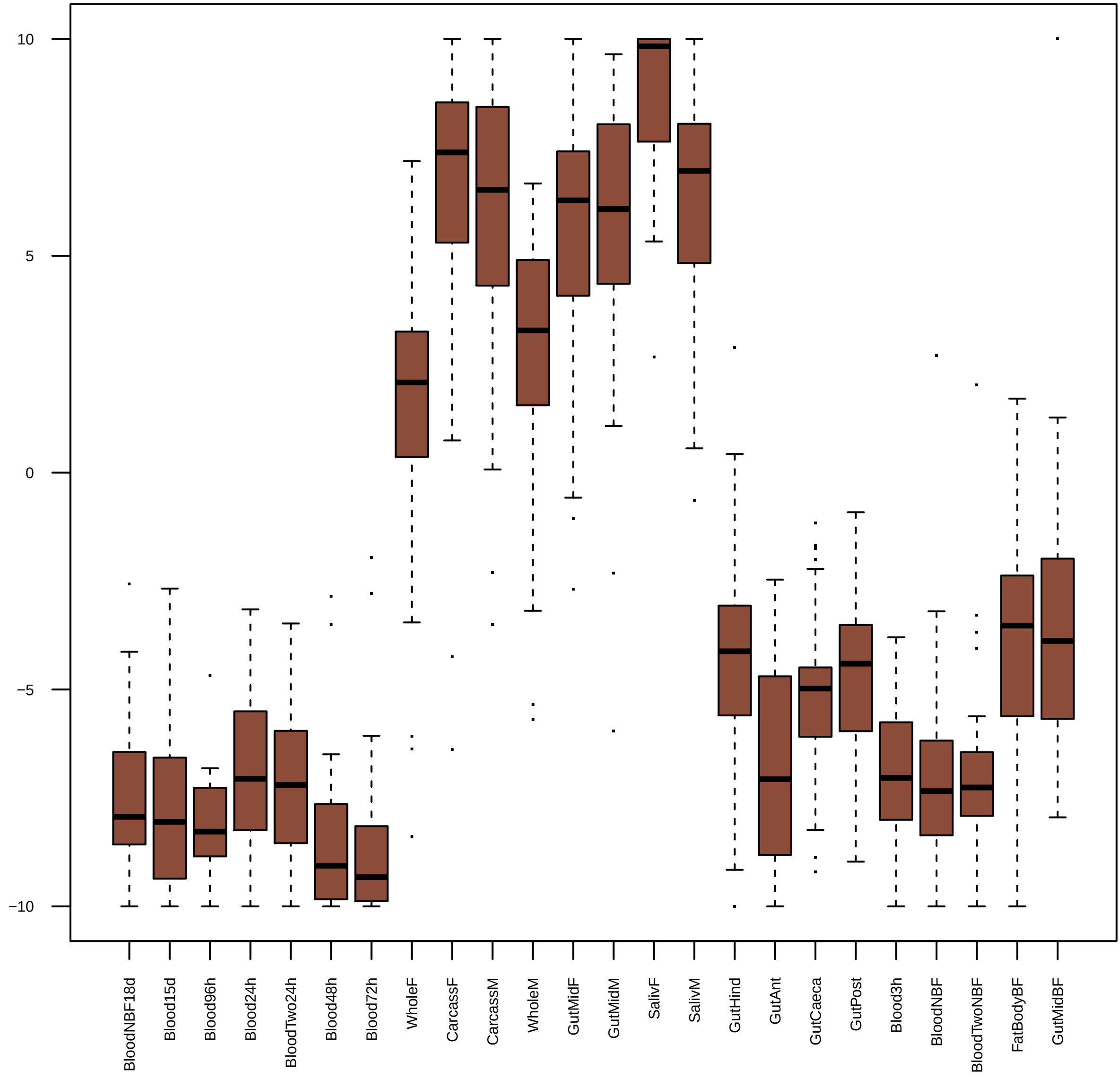

| | GO.ID | BPCluster: salmon4 Size: 39 | Annotated | Significant | Expected | Rank in ClassicF | Weight01F | ClassicF |
|---|---|---|---|---|---|---|---|---|
| *1* | GO:0006904 | vesicle docking involved in exocytosis | 15 | 2 | 0.05 | 1 | 0.0011 | 0.0011 |
| *3* | GO:0007033 | vacuole organization | 20 | 2 | 0.07 | 5 | 0.0249 | 0.0019 |
| *7* | GO:0006906 | vesicle fusion | 22 | 2 | 0.07 | 6 | 0.0340 | 0.0023 |

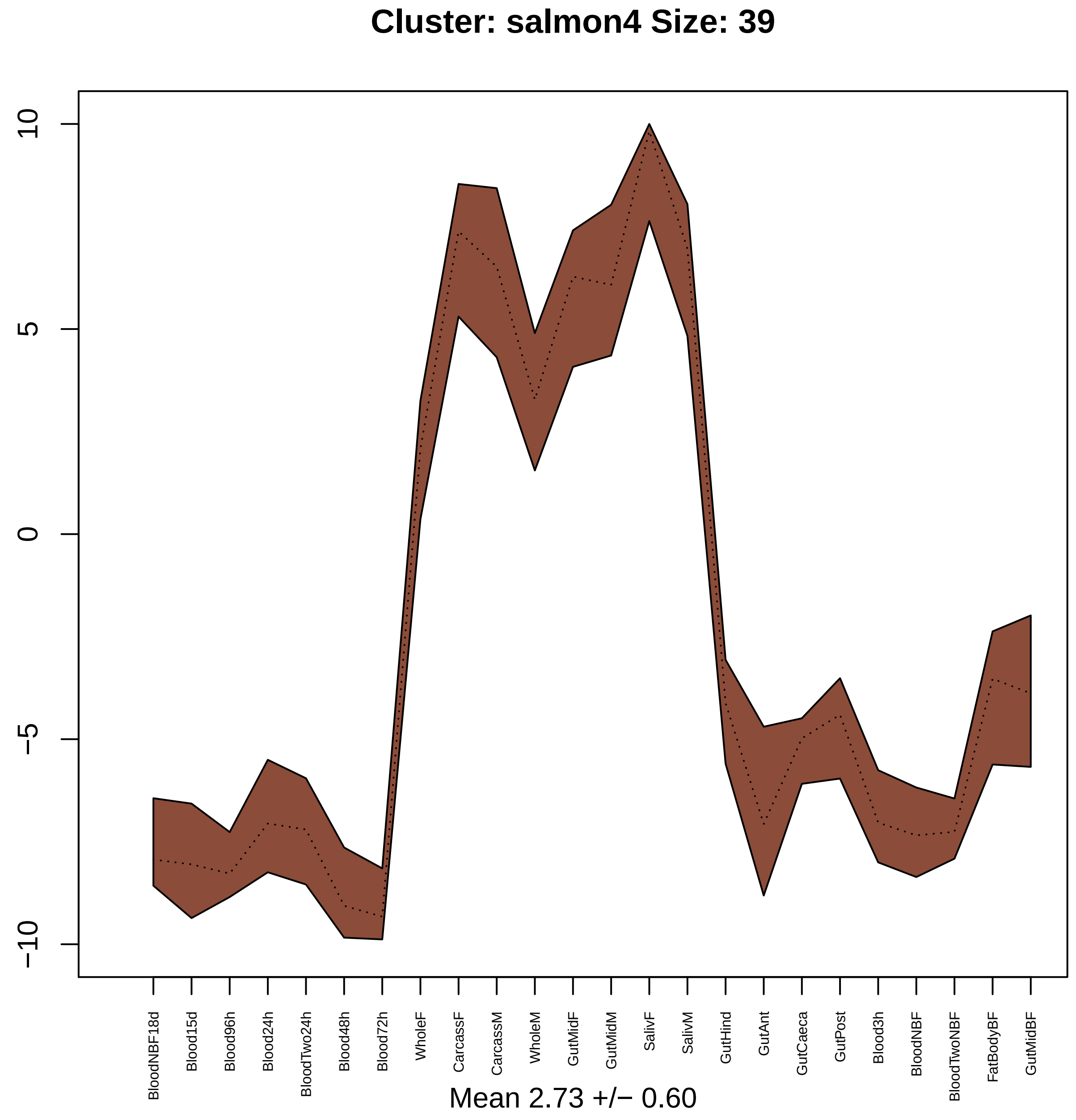
Cluster: salmon4 Size: 39
10
5
0
−5
−10
BloodNBF18d
Blood15d
Blood96h
Blood24h
BloodTwo24h
Blood48h
Blood72h
WholeF
CarcassF
CarcassM
WholeM
GutMidF
GutMidM
SalivF
SalivM
GutHind
GutAnt
GutCaeca
GutPost
Blood3h
BloodNBF
BloodTwoNBF
FatBodyBF
GutMidBF
Mean 2.73 +/− 0.60

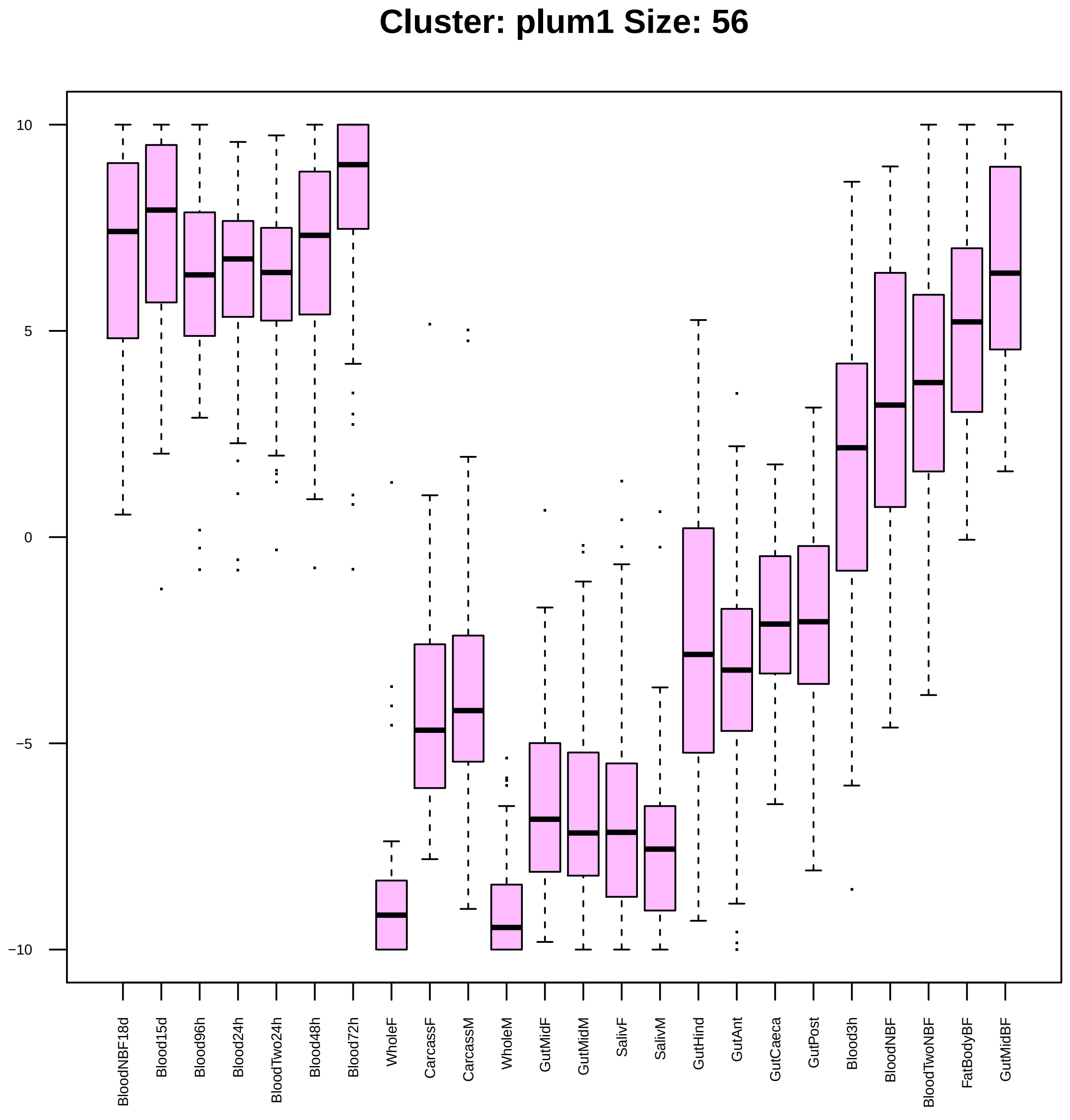

Cluster: plum1 Size: 56
10
5
0
−5
−10
BloodNBF18d
Blood15d
Blood96h
Blood24h
BloodTwo24h
Blood48h
Blood72h
WholeF
CarcassF
CarcassM
WholeM
GutMidF
GutMidM
SalivF
SalivM
GutHind
GutAnt
GutCaeca
GutPost
Blood3h
BloodNBF
BloodTwoNBF
FatBodyBF
GutMidBF

| | GO.ID | BPCluster: plum1 Size: 56 | Annotated | Significant | Expected | Rank in ClassicF | Weight01F | ClassicF |
|---|---|---|---|---|---|---|---|---|
| 1 | GO:0007277 | pole cell development | 10 | 2 | 0.05 | 2 | 0.0011 | 0.00107 |
| 2 | GO:0070507 | regulation of microtubule cytoskeleton o... | 19 | 2 | 0.10 | 8 | 0.0040 | 0.00396 |
| 3 | GO:0016325 | oocyte microtubule cytoskeleton organiza... | 22 | 2 | 0.11 | 10 | 0.0053 | 0.00529 |
| 4 | GO:0060811 | intracellular mRNA localization involved... | 30 | 2 | 0.15 | 19 | 0.0146 | 0.00972 |
| 9 | GO:0008298 | intracellular mRNA localization | 39 | 3 | 0.20 | 1 | 0.0372 | 0.00093 |
| 16 | GO:0007143 | female meiotic nuclear division | 26 | 2 | 0.13 | 17 | 0.0616 | 0.00736 |

| | GO.ID | MFCluster: plum1 Size: 56 | Annotated | Significant | Expected | Rank in ClassicF | Weight01F | ClassicF |
|---|---|---|---|---|---|---|---|---|
| *1* | GO:0008270 | zinc ion binding | 522 | 8 | 3.04 | 2 | 0.0094 | 0.0094 |
| *2* | GO:0005515 | protein binding | 2143 | 21 | 12.49 | 1 | 0.0112 | 0.0045 |

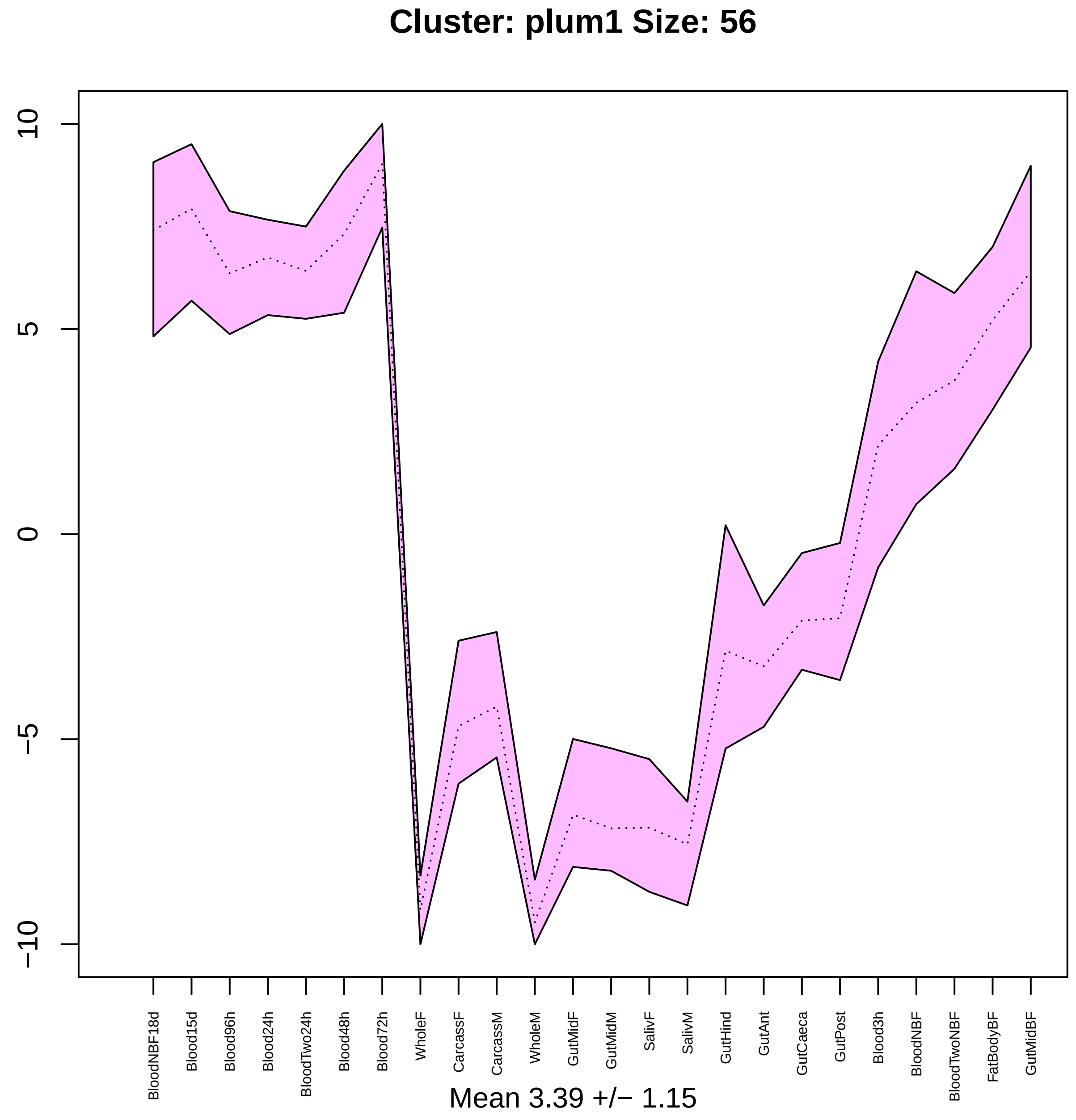
Cluster: plum1 Size: 56
10
5
0
−5
−10
BloodNBF18d
Blood15d
Blood96h
Blood24h
BloodTwo24h
Blood48h
Blood72h
WholeF
CarcassF
CarcassM
WholeM
GutMidF
GutMidM
SalivF
SalivM
GutHind
GutAnt
GutCaeca
GutPost
Blood3h
BloodNBF
BloodTwoNBF
FatBodyBF
GutMidBF
Mean 3.39 +/− 1.15

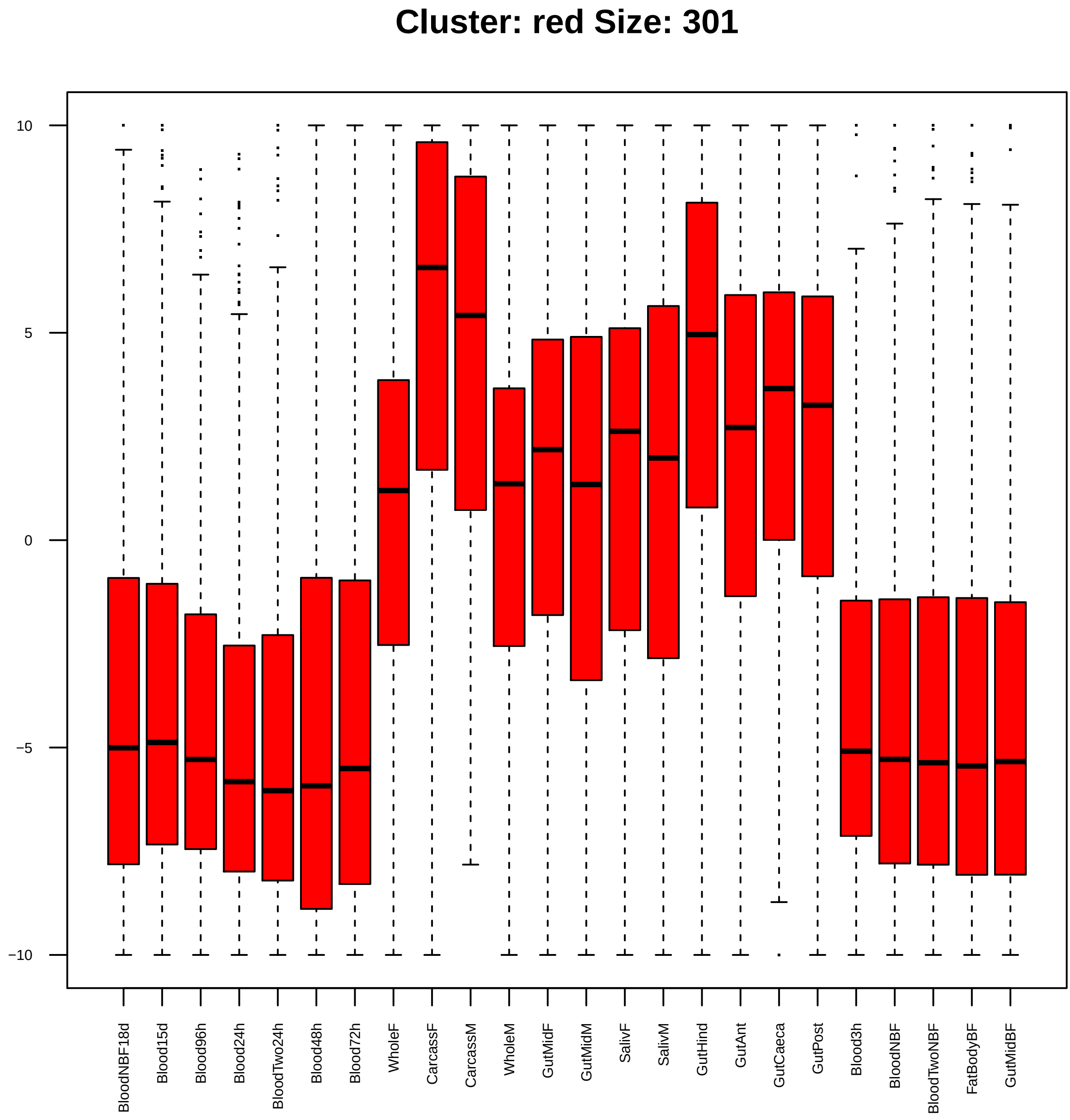
Cluster: red Size: 301
10
5
0
−5
−10
BloodNBF18d
Blood15d
Blood96h
Blood24h
BloodTwo24h
Blood48h
Blood72h
WholeF
CarcassF
CarcassM
WholeM
GutMidF
GutMidM
SalivF
SalivM
GutHind
GutAnt
GutCaeca
GutPost
Blood3h
BloodNBF
BloodTwoNBF
FatBodyBF
GutMidBF

| | GO.ID | BPCluster: red Size: 301 | Annotated | Significant | Expected | Rank in ClassicF | Weight01F | ClassicF |
|---|---|---|---|---|---|---|---|---|
| *1* | GO:0006412 | translation | 289 | 21 | 8.70 | 2 | 0.00017 | 0.00014 |
| *2* | GO:0015893 | drug transport | 11 | 3 | 0.33 | 7 | 0.00371 | 0.00371 |
| *10* | GO:0007098 | centrosome cycle | 60 | 6 | 1.81 | 8 | 0.02960 | 0.00894 |

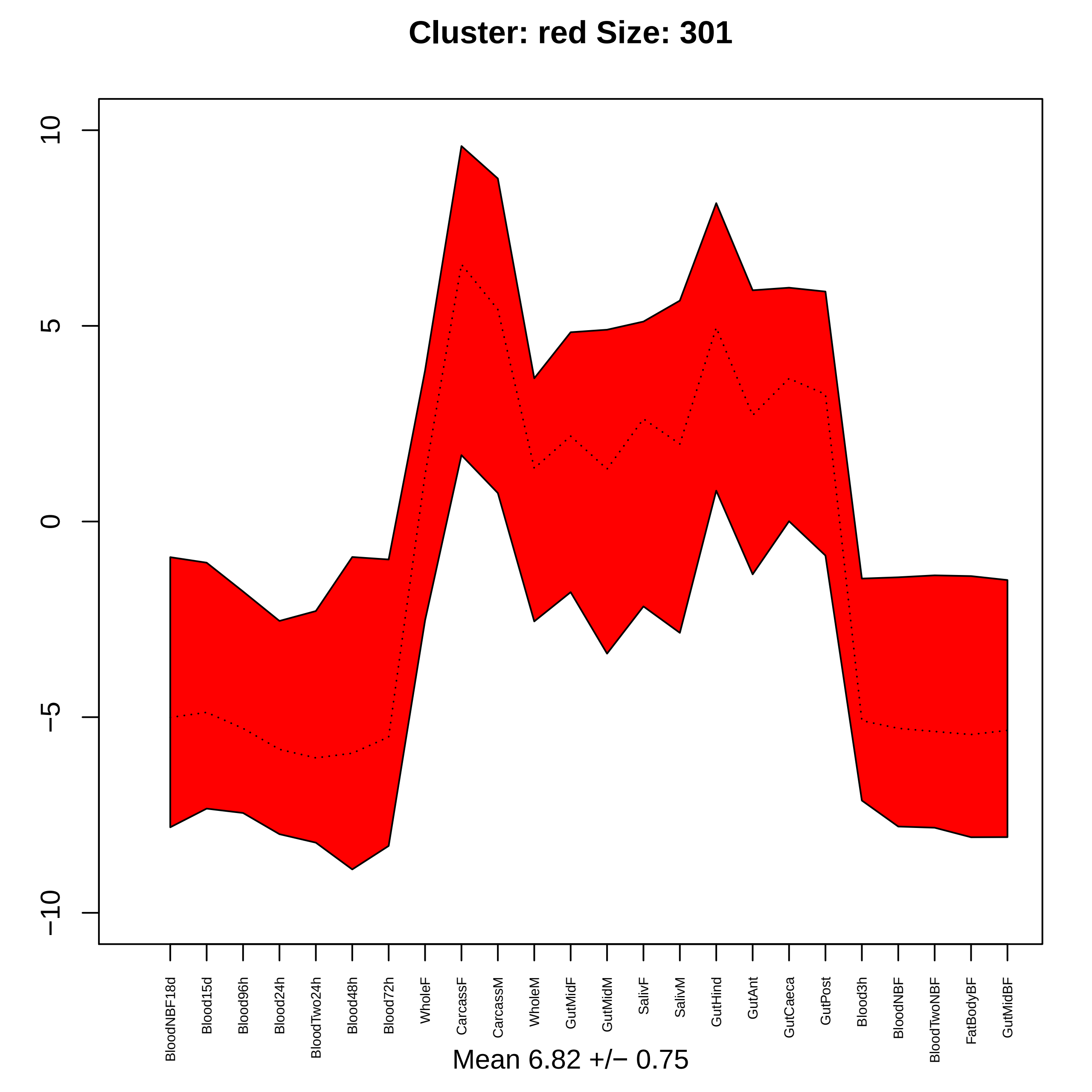

Cluster: red Size: 301
10
5
0
−5
−10
BloodNBF18d
Blood15d
Blood96h
Blood24h
BloodTwo24h
Blood48h
Blood72h
WholeF
CarcassF
CarcassM
WholeM
GutMidF
GutMidM
SalivF
SalivM
GutHind
GutAnt
GutCaeca
GutPost
Blood3h
BloodNBF
BloodTwoNBF
FatBodyBF
GutMidBF
Mean 6.82 +/− 0.75

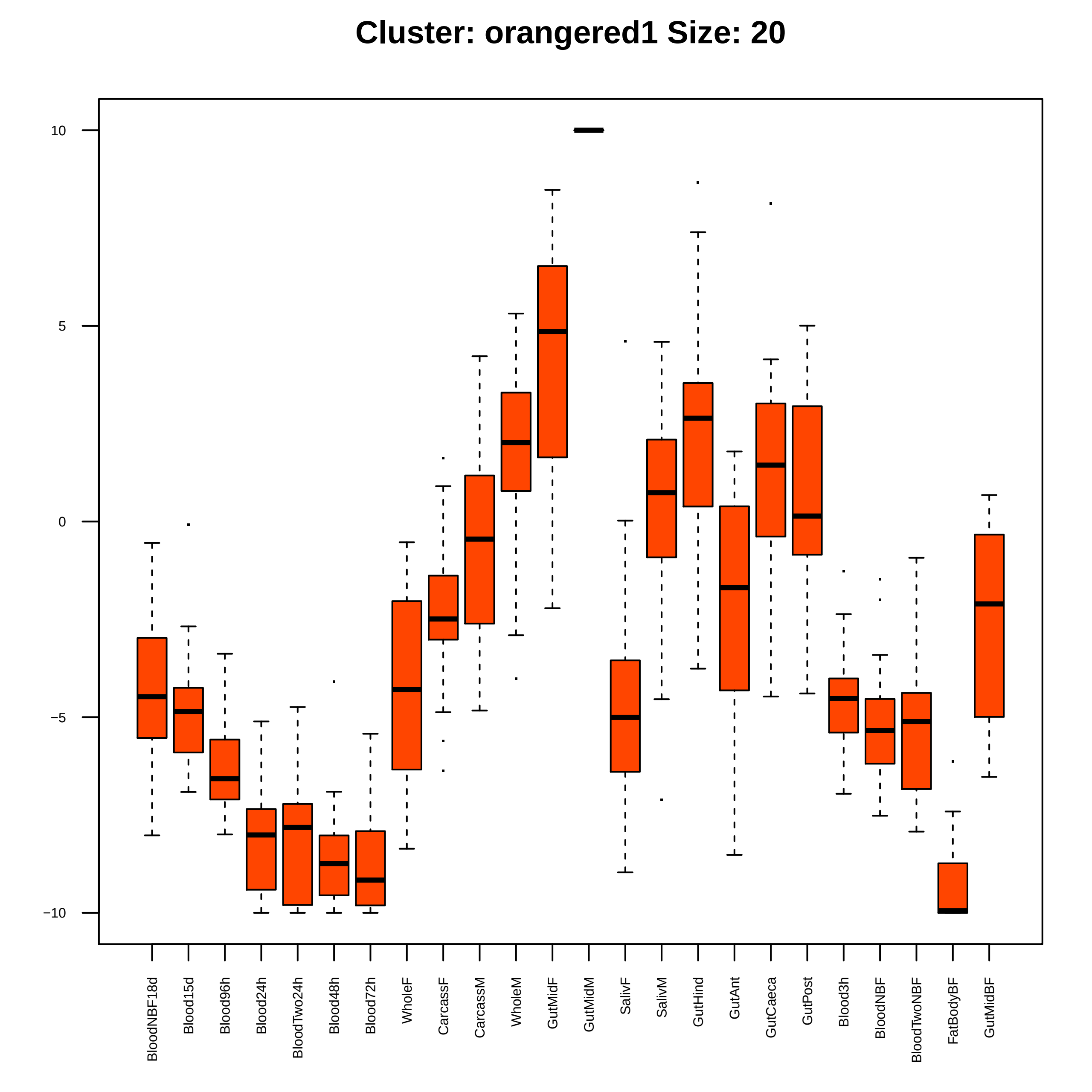
Cluster: orangered1 Size: 20
10
5
0
−5
−10
BloodNBF18d
Blood15d
Blood96h
Blood24h
BloodTwo24h
Blood48h
Blood72h
WholeF
CarcassF
CarcassM
WholeM
GutMidF
GutMidM
SalivF
SalivM
GutHind
GutAnt
GutCaeca
GutPost
Blood3h
BloodNBF
BloodTwoNBF
FatBodyBF
GutMidBF

| | GO.ID | BPCluster: orangered1 Size: 20 | Annotated | Significant | Expected | Rank in ClassicF | Weight01F | ClassicF |
|---|---|---|---|---|---|---|---|---|
| *1* | GO:0008152 | metabolic process | 4285 | 14 | 12.10 | 58 | 0.0053 | 0.2454 |
| *28* | GO:0030534 | adult behavior | 49 | 2 | 0.14 | 1 | 1.0000 | 0.0082 |

| | GO.ID | MFCluster: orangered1 Size: 20 | Annotated | Significant | Expected | Rank in ClassicF | Weight01F | ClassicF |
|---|---|---|---|---|---|---|---|---|
| *3* | GO:0016705 | oxidoreductase activity, acting on paire... | 133 | 4 | 0.30 | 4 | 0.00017 | 0.00017 |
| *4* | GO:0020037 | heme binding | 133 | 4 | 0.30 | 5 | 0.00017 | 0.00017 |
| *16* | GO:0003824 | catalytic activity | 3160 | 13 | 7.01 | 7 | 0.22045 | 0.00257 |
| *23* | GO:0046906 | tetrapyrrole binding | 134 | 4 | 0.30 | 6 | 1.00000 | 0.00017 |

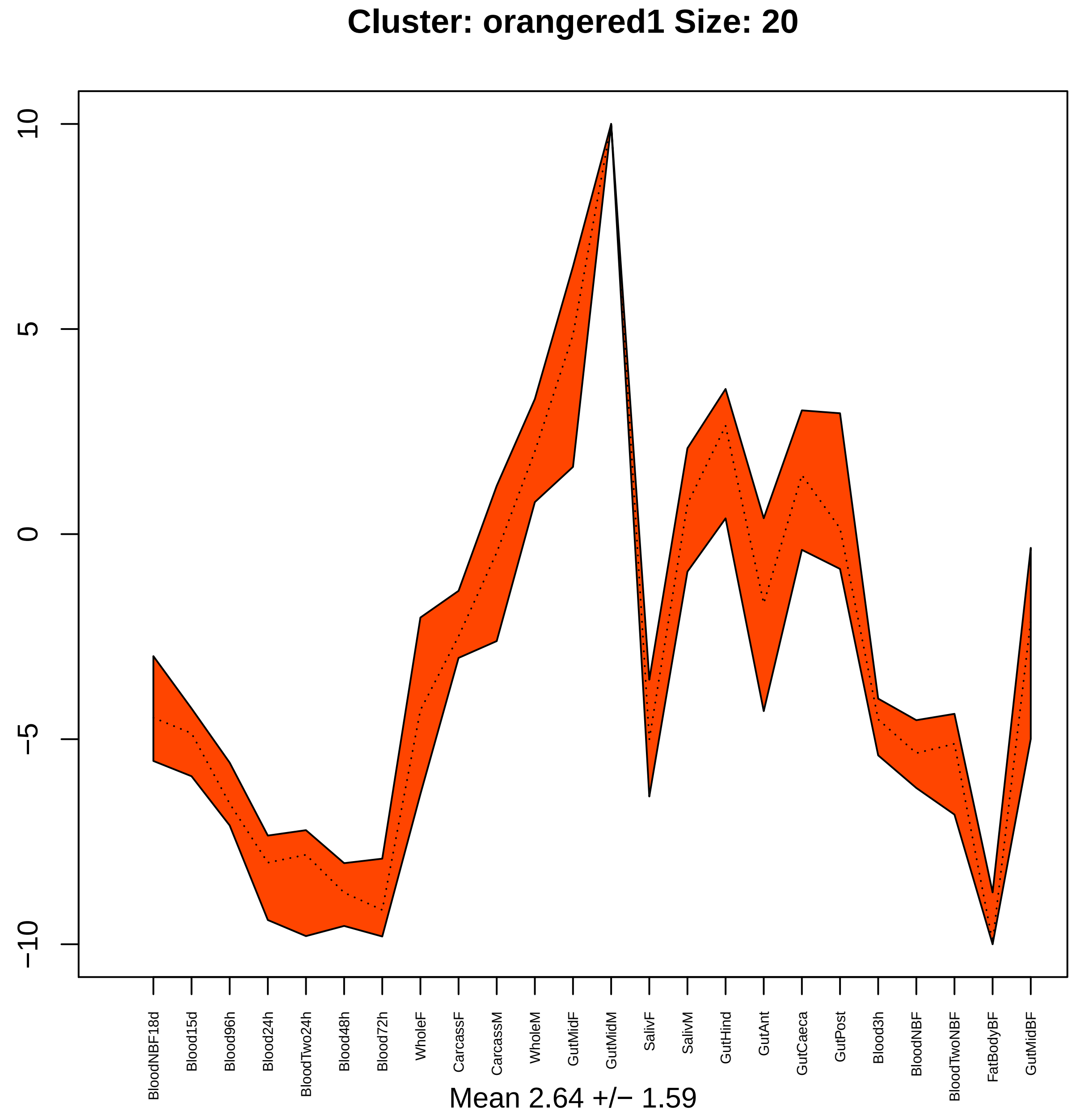

Cluster: orangered1 Size: 20
10
5
0
−5
−10
BloodNBF18d
Blood15d
Blood96h
Blood24h
BloodTwo24h
Blood48h
Blood72h
WholeF
CarcassF
CarcassM
WholeM
GutMidF
GutMidM
SalivF
SalivM
GutHind
GutAnt
GutCaeca
GutPost
Blood3h
BloodNBF
BloodTwoNBF
FatBodyBF
GutMidBF
Mean 2.64 +/− 1.59

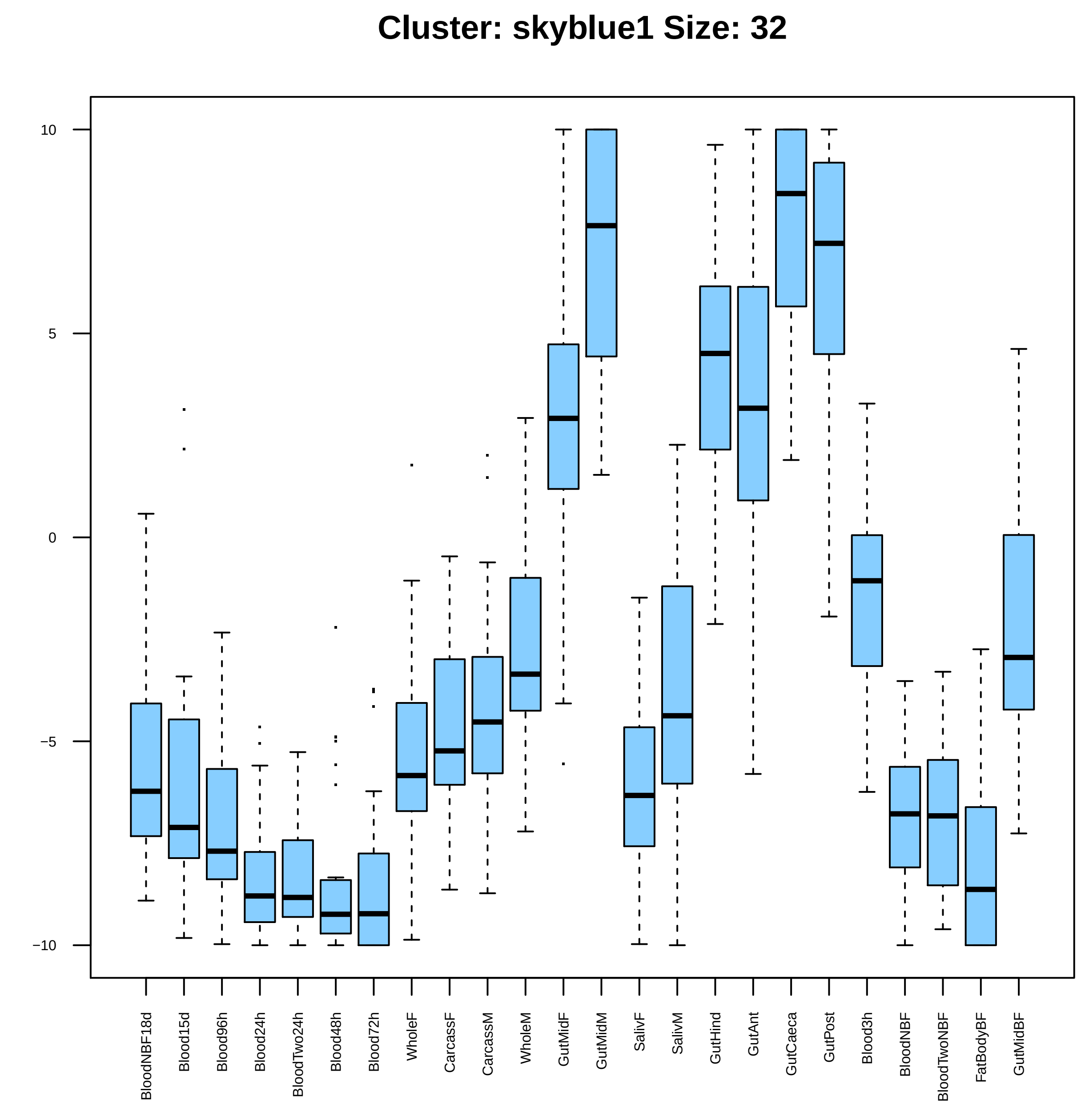
Cluster: skyblue1 Size: 32
10
5
0
−5
−10
BloodNBF18d
Blood15d
Blood96h
Blood24h
BloodTwo24h
Blood48h
Blood72h
WholeF
CarcassF
CarcassM
WholeM
GutMidF
GutMidM
SalivF
SalivM
GutHind
GutAnt
GutCaeca
GutPost
Blood3h
BloodNBF
BloodTwoNBF
FatBodyBF
GutMidBF

| | GO.ID | BPCluster: skyblue1 Size: 32 | Annotated | Significant | Expected | Rank in ClassicF | Weight01F | ClassicF |
|---|---|---|---|---|---|---|---|---|
| *2* | GO:0008152 | metabolic process | 4285 | 22 | 16.8 | 2 | 0.0022 | 0.017 |

| | GO.ID | MFCluster: skyblue1 Size: 32 | Annotated | Significant | Expected | Rank in ClassicF | Weight01F | ClassicF |
|---|---|---|---|---|---|---|---|---|
| *2* | GO:0004497 | monooxygenase activity | 110 | 3 | 0.4 | 5 | 0.007 | 0.007 |

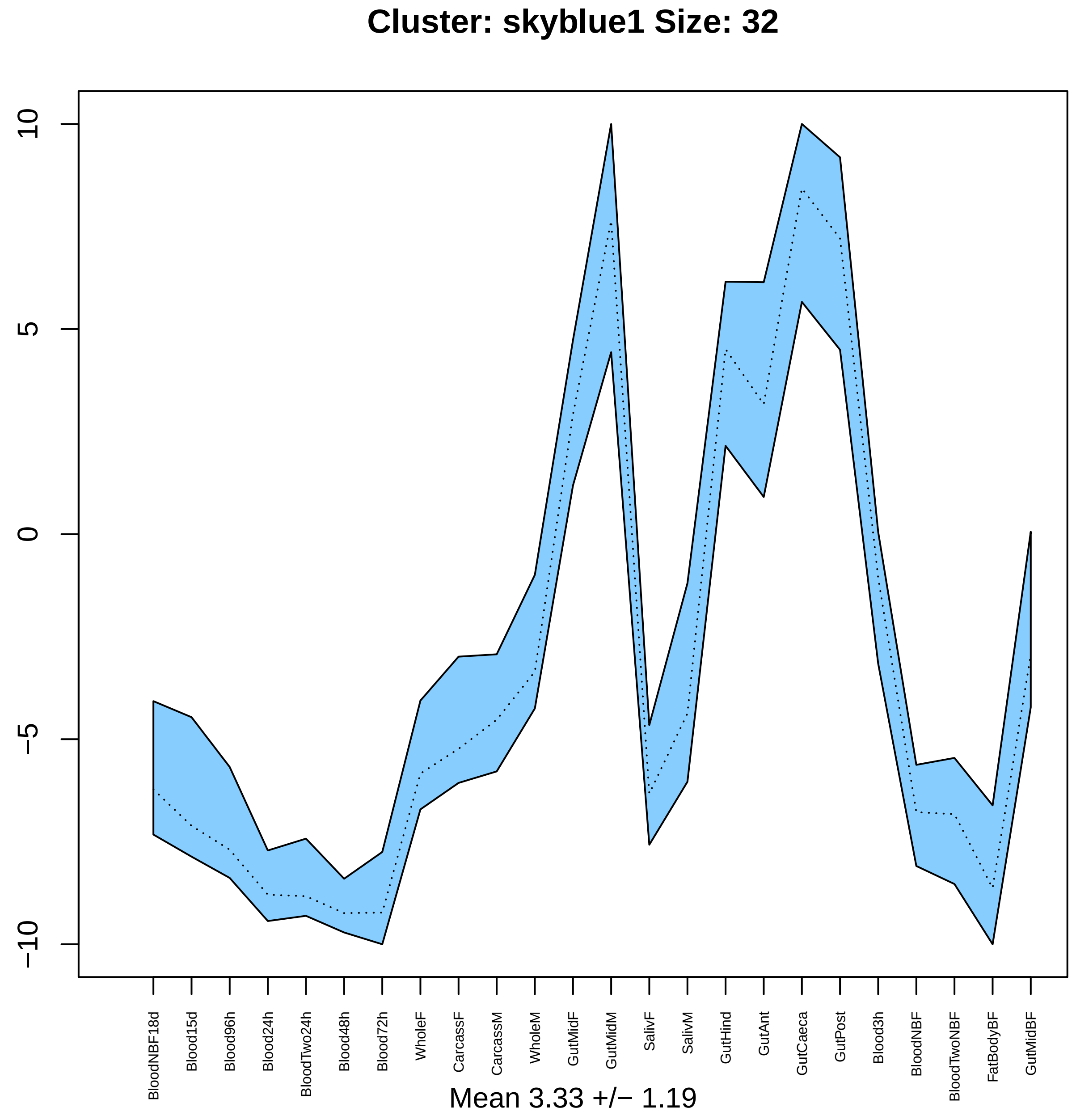

Cluster: skyblue1 Size: 32
10
5
0
−5
−10
BloodNBF18d
Blood15d
Blood96h
Blood24h
BloodTwo24h
Blood48h
Blood72h
WholeF
CarcassF
CarcassM
WholeM
GutMidF
GutMidM
SalivF
SalivM
GutHind
GutAnt
GutCaeca
GutPost
Blood3h
BloodNBF
BloodTwoNBF
FatBodyBF
GutMidBF
Mean 3.33 +/− 1.19

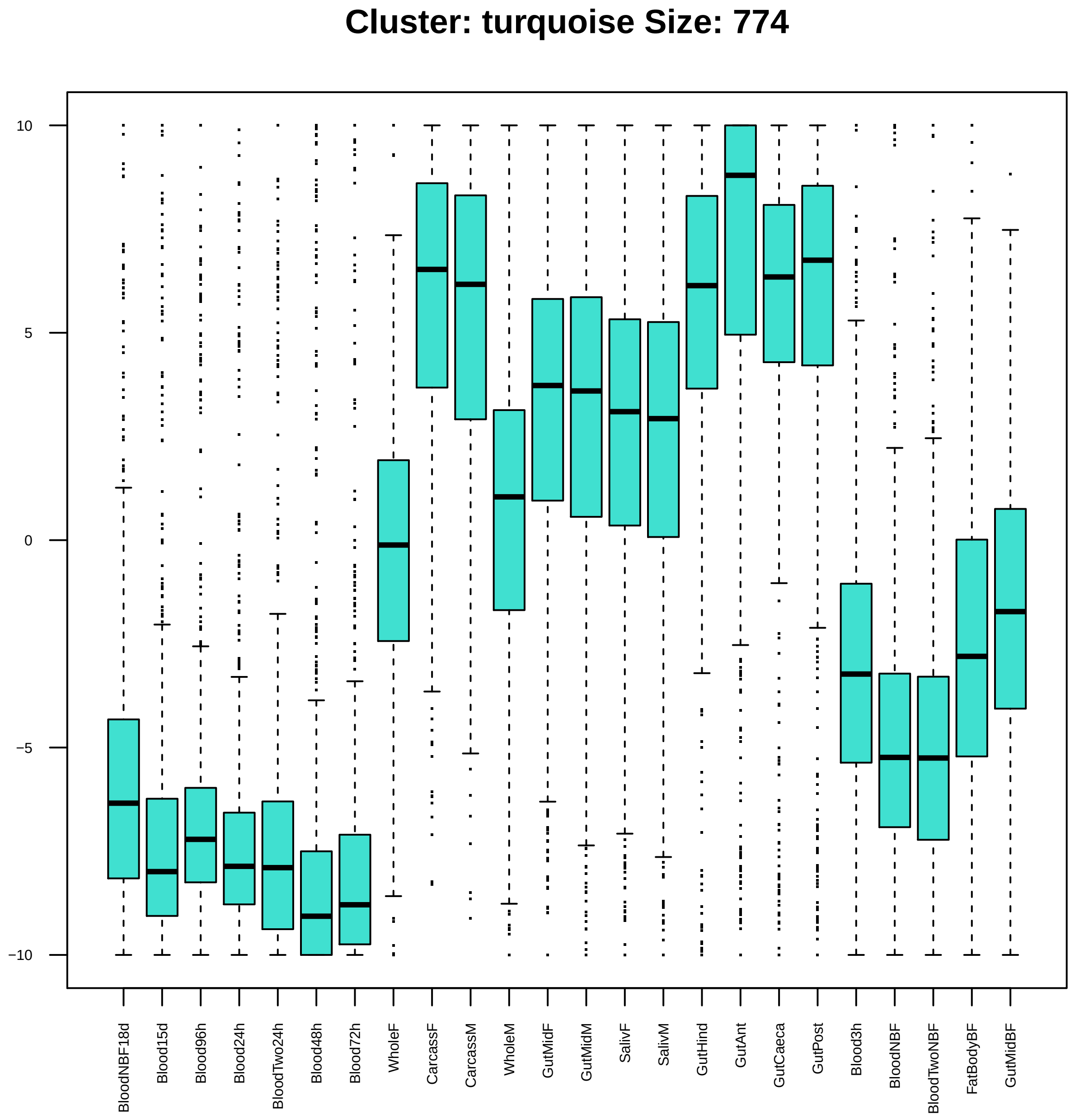

Cluster: turquoise Size: 774
10
5
0
−5
−10
BloodNBF18d
Blood15d
Blood96h
Blood24h
BloodTwo24h
Blood48h
Blood72h
WholeF
CarcassF
CarcassM
WholeM
GutMidF
GutMidM
SalivF
SalivM
GutHind
GutAnt
GutCaeca
GutPost
Blood3h
BloodNBF
BloodTwoNBF
FatBodyBF
GutMidBF

| | GO.ID | BPCluster: turquoise Size: 774 | Annotated | Significant | Expected | Rank in ClassicF | Weight01F | ClassicF |
|---|---|---|---|---|---|---|---|---|
| *4* | GO:0034220 | ion transmembrane transport | 275 | 32 | 19.92 | 18 | 0.0030 | 0.00478 |
| *5* | GO:0050909 | sensory perception of taste | 56 | 13 | 4.06 | 14 | 0.0035 | 0.00013 |
| *6* | GO:0006066 | alcohol metabolic process | 37 | 9 | 2.68 | 15 | 0.0038 | 0.00101 |
| *7* | GO:0071805 | potassium ion transmembrane transport | 23 | 6 | 1.67 | 19 | 0.0049 | 0.00488 |
| *8* | GO:0050912 | detection of chemical stimulus involved ... | 12 | 4 | 0.87 | 22 | 0.0084 | 0.00843 |

| | GO.ID | MFCluster: turquoise Size: 774 | Annotated | Significant | Expected | Rank in ClassicF | Weight01F | ClassicF |
|---|---|---|---|---|---|---|---|---|
| *6* | GO:0008527 | taste receptor activity | 12 | 5 | 0.90 | 25 | 0.0012 | 0.0012 |
| *7* | GO:0003995 | acyl−CoA dehydrogenase activity | 14 | 5 | 1.05 | 27 | 0.0026 | 0.0026 |
| *8* | GO:0050660 | flavin adenine dinucleotide binding | 58 | 11 | 4.35 | 28 | 0.0034 | 0.0034 |
| *9* | GO:0030594 | neurotransmitter receptor activity | 45 | 16 | 3.38 | 6 | 0.0035 | 6.8e−08 |
| *10* | GO:0005249 | voltage−gated potassium channel activity | 15 | 5 | 1.13 | 29 | 0.0037 | 0.0037 |
| *11* | GO:0003705 | transcription factor activity, RNA polym... | 32 | 7 | 2.40 | 33 | 0.0083 | 0.0083 |
| *12* | GO:0008812 | choline dehydrogenase activity | 18 | 5 | 1.35 | 35 | 0.0088 | 0.0088 |
| *14* | GO:0043565 | sequence−specific DNA binding | 212 | 27 | 15.91 | 30 | 0.0194 | 0.0045 |
| *16* | GO:0042302 | structural constituent of cuticle | 99 | 15 | 7.43 | 32 | 0.0270 | 0.0066 |

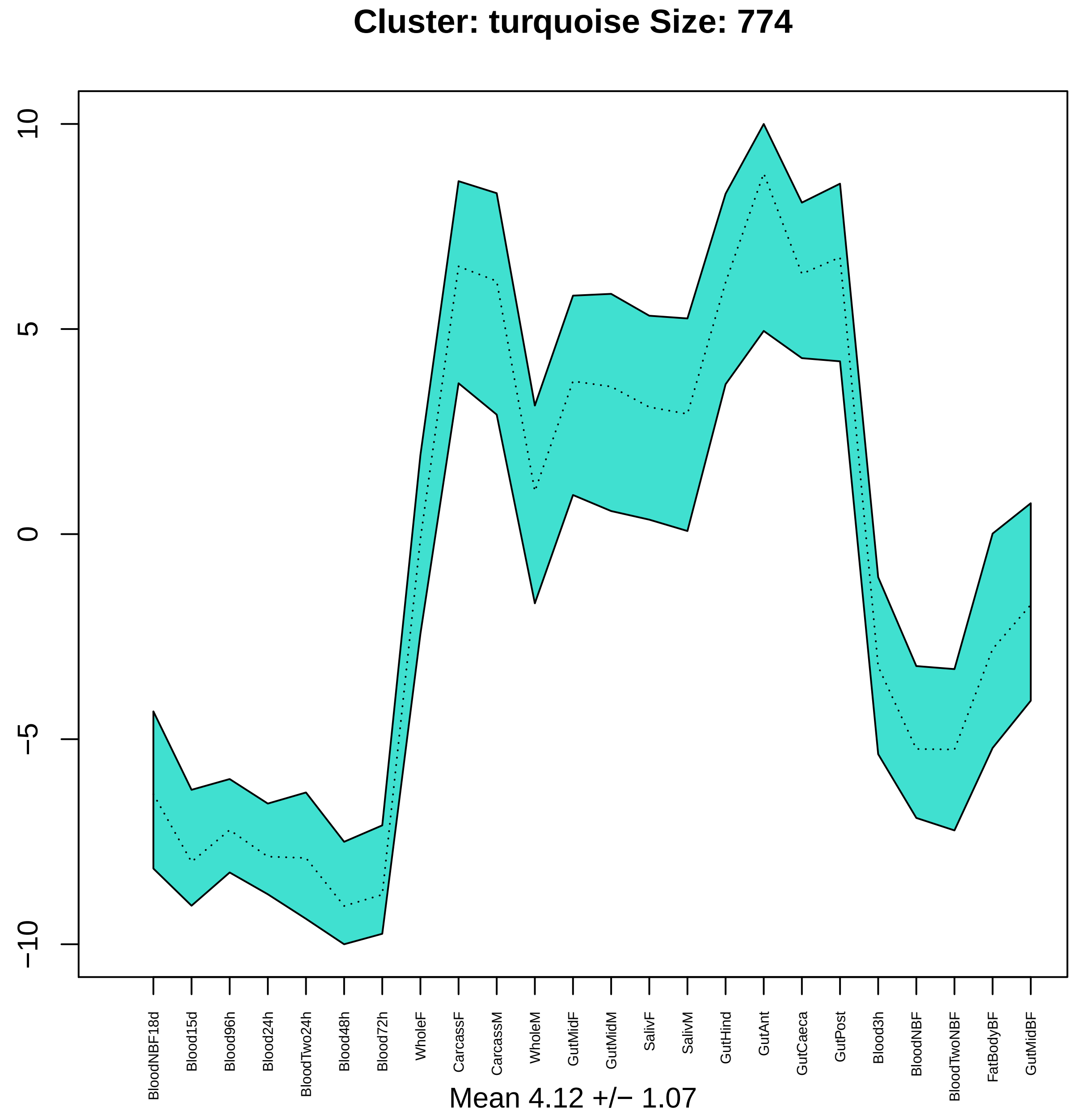

Cluster: turquoise Size: 774
10
5
0
−5
−10
BloodNBF18d
Blood15d
Blood96h
Blood24h
BloodTwo24h
Blood48h
Blood72h
WholeF
CarcassF
CarcassM
WholeM
GutMidF
GutMidM
SalivF
SalivM
GutHind
GutAnt
GutCaeca
GutPost
Blood3h
BloodNBF
BloodTwoNBF
FatBodyBF
GutMidBF
Mean 4.12 +/− 1.07

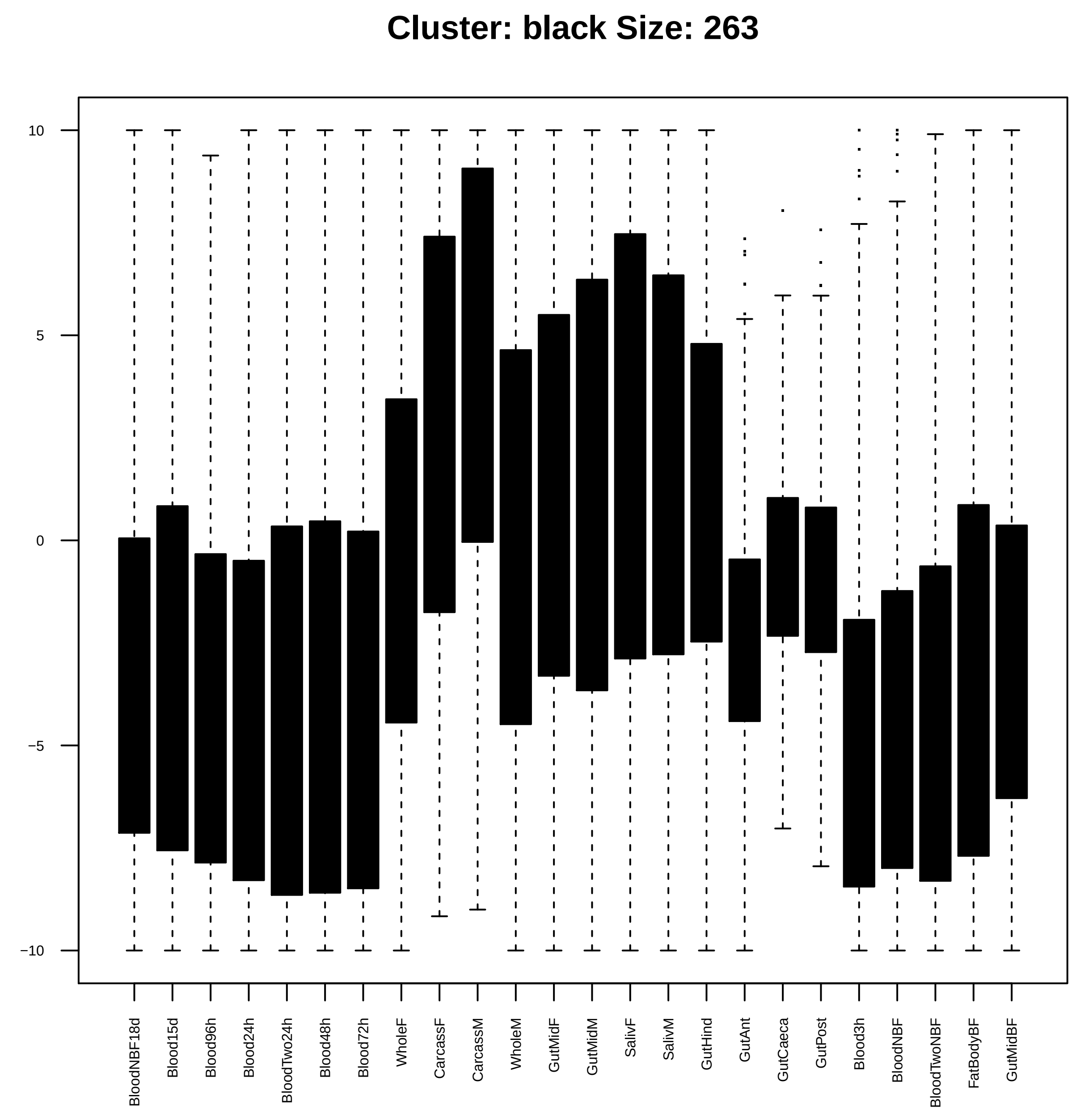
Cluster: black Size: 263
10
5
0
−5
−10
BloodNBF18d
Blood15d
Blood96h
Blood24h
BloodTwo24h
Blood48h
Blood72h
WholeF
CarcassF
CarcassM
WholeM
GutMidF
GutMidM
SalivF
SalivM
GutHind
GutAnt
GutCaeca
GutPost
Blood3h
BloodNBF
BloodTwoNBF
FatBodyBF
GutMidBF

| | GO.ID | BPCluster: black Size: 263 | Annotated | Significant | Expected | Rank in ClassicF | Weight01F | ClassicF |
|---|---|---|---|---|---|---|---|---|
| *1* | GO:0046580 | negative regulation of Ras protein signa... | 12 | 3 | 0.32 | 1 | 0.0035 | 0.0035 |
| *2* | GO:0045475 | locomotor rhythm | 24 | 4 | 0.65 | 3 | 0.0036 | 0.0036 |
| *3* | GO:0006904 | vesicle docking involved in exocytosis | 15 | 3 | 0.40 | 4 | 0.0069 | 0.0069 |
| *4* | GO:0030163 | protein catabolic process | 111 | 6 | 2.99 | 88 | 0.0098 | 0.0788 |

|  | GO.ID | MFCluster: black Size: 263 | Annotated | Significant | Expected | Rank in ClassicF | Weight01F | ClassicF |
|---|---|---|---|---|---|---|---|---|
| *1* | GO:0004520 | endodeoxyribonuclease activity | 12 | 3 | 0.30 | 1 | 0.0029 | 0.0029 |
| *2* | GO:0016893 | endonuclease activity, active with eithe... | 16 | 3 | 0.40 | 3 | 0.0059 | 0.0069 |
| *18* | GO:0019887 | protein kinase regulator activity | 29 | 4 | 0.73 | 2 | 0.0725 | 0.0056 |

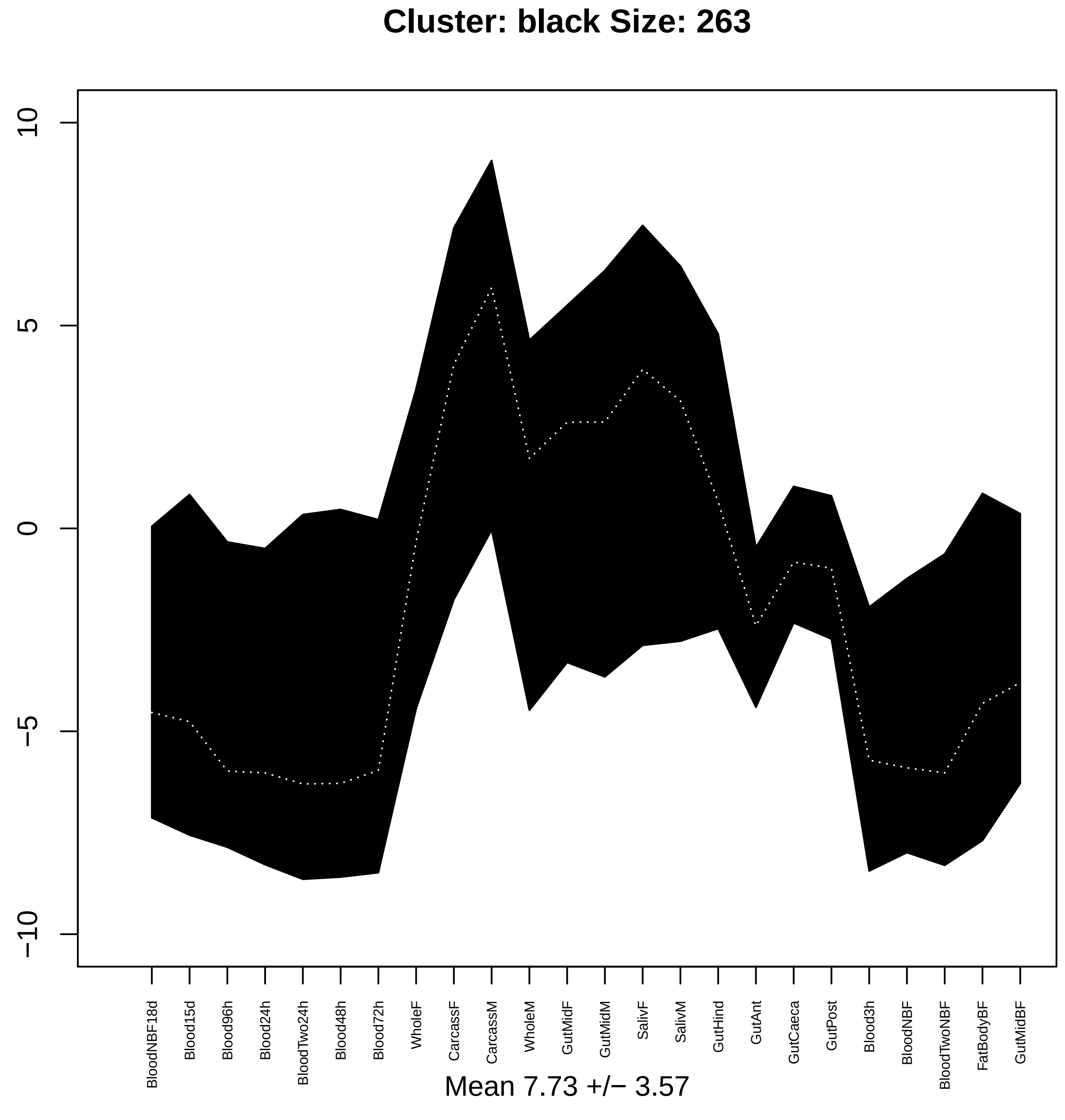

Cluster: black Size: 263
10
5
0
−5
−10
BloodNBF18d
Blood15d
Blood96h
Blood24h
BloodTwo24h
Blood48h
Blood72h
WholeF
CarcassF
CarcassM
WholeM
GutMidF
GutMidM
SalivF
SalivM
GutHind
GutAnt
GutCaeca
GutPost
Blood3h
BloodNBF
BloodTwoNBF
FatBodyBF
GutMidBF
Mean 7.73 +/− 3.57

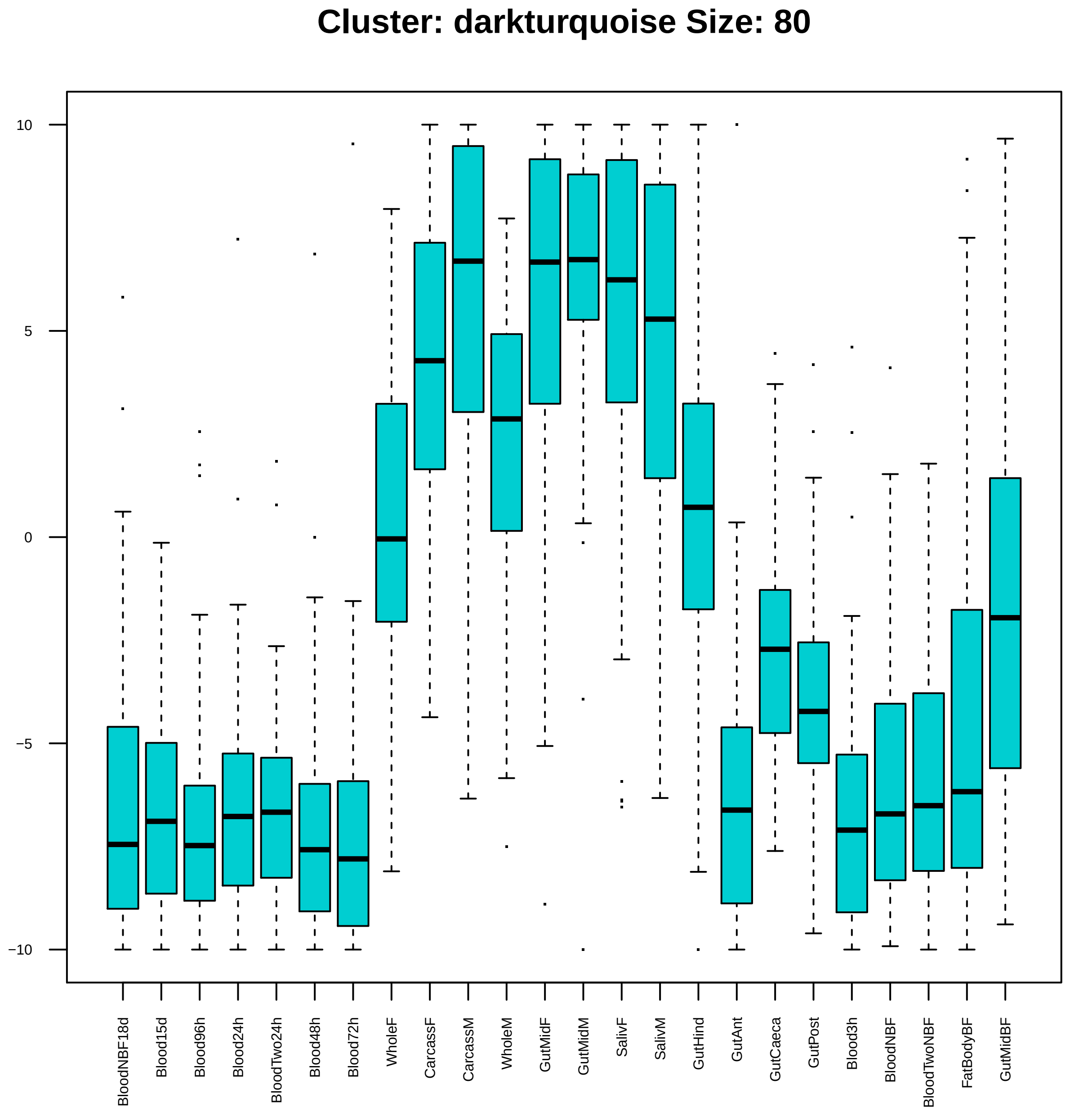
Cluster: darkturquoise Size: 80
10
5
0
−5
−10
BloodNBF18d
Blood15d
Blood96h
Blood24h
BloodTwo24h
Blood48h
Blood72h
WholeF
CarcassF
CarcassM
WholeM
GutMidF
GutMidM
SalivF
SalivM
GutHind
GutAnt
GutCaeca
GutPost
Blood3h
BloodNBF
BloodTwoNBF
FatBodyBF
GutMidBF

| | GO.ID | BPCluster: darkturquoise Size: 80 | Annotated | Significant | Expected | Rank in ClassicF | Weight01F | ClassicF |
|---|---|---|---|---|---|---|---|---|
| *1* | GO:0009792 | embryo development ending in birth or eg... | 96 | 3 | 0.89 | 62 | 0.00082 | 0.05871 |
| *2* | GO:0043901 | negative regulation of multi−organism pr... | 10 | 2 | 0.09 | 5 | 0.00361 | 0.00361 |
| *3* | GO:0034976 | response to endoplasmic reticulum stress | 11 | 2 | 0.10 | 6 | 0.00439 | 0.00439 |
| *4* | GO:0008593 | regulation of Notch signaling pathway | 52 | 4 | 0.48 | 2 | 0.00495 | 0.00129 |
| *28* | GO:0007219 | Notch signaling pathway | 63 | 5 | 0.58 | 1 | 0.09167 | 0.00027 |

| | GO.ID | MFCluster: darkturquoise Size: 80 | Annotated | Significant | Expected | Rank in ClassicF | Weight01F | ClassicF |
|---|---|---|---|---|---|---|---|---|
| *1* | GO:0003730 | mRNA 3'−UTR binding | 16 | 2 | 0.12 | 1 | 0.0064 | 0.0064 |
| *2* | GO:0046982 | protein heterodimerization activity | 55 | 3 | 0.42 | 2 | 0.0083 | 0.0083 |

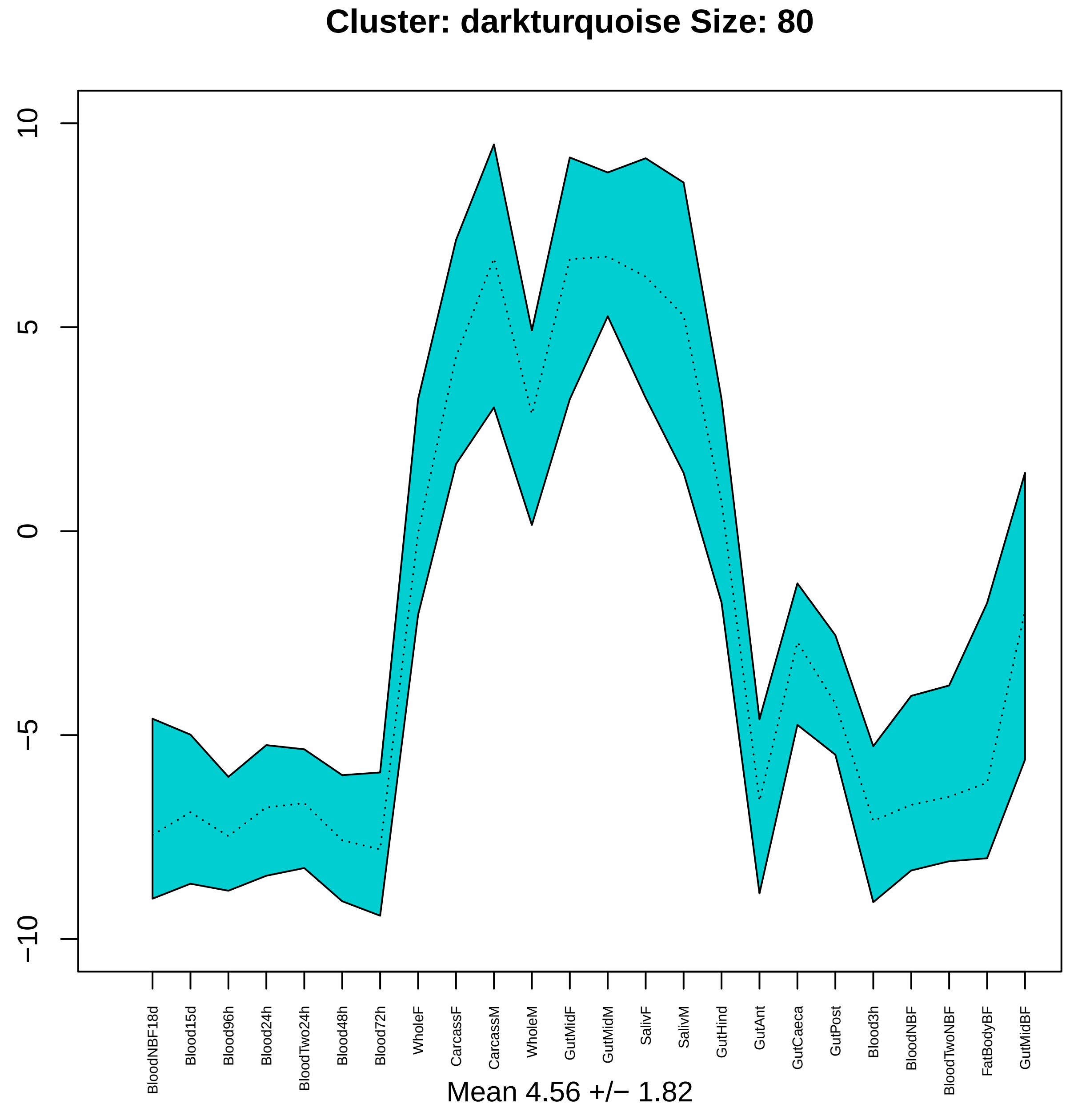
Cluster: darkturquoise Size: 80
10
5
0
−5
−10
BloodNBF18d
Blood15d
Blood96h
Blood24h
BloodTwo24h
Blood48h
Blood72h
WholeF
CarcassF
CarcassM
WholeM
GutMidF
GutMidM
SalivF
SalivM
GutHind
GutAnt
GutCaeca
GutPost
Blood3h
BloodNBF
BloodTwoNBF
FatBodyBF
GutMidBF
Mean 4.56 +/− 1.82

# Cluster: brown Size: 411

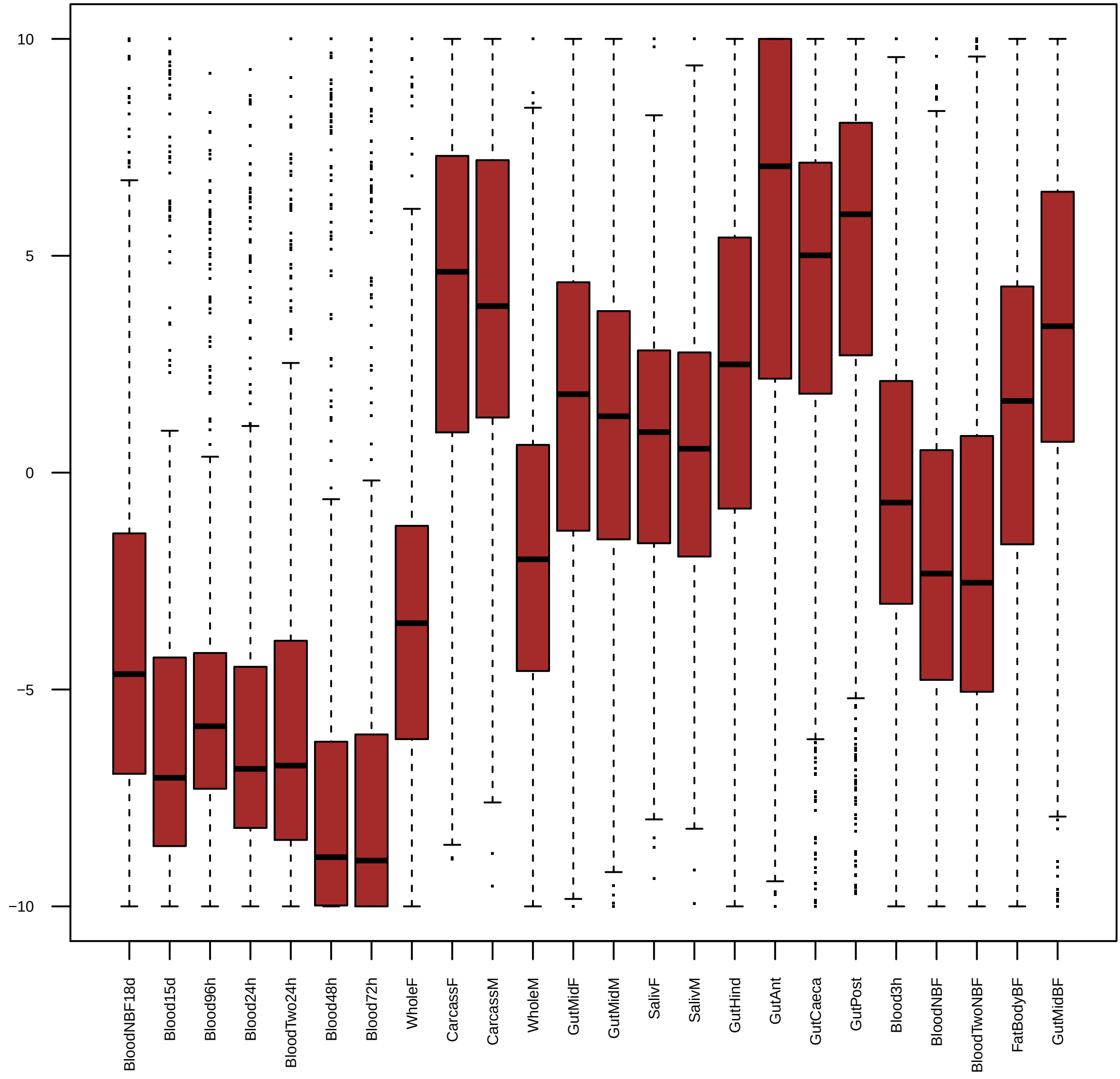

| | GO.ID | BPCluster: brown Size: 411 | Annotated | Significant | Expected | Rank in ClassicF | Weight01F | ClassicF |
|---|---|---|---|---|---|---|---|---|
| *1* | GO:0006355 | regulation of transcription, DNA−templat... | 501 | 32 | 17.60 | 7 | 0.00017 | 0.00061 |
| *2* | GO:0007186 | G protein−coupled receptor signaling pat... | 228 | 20 | 8.01 | 3 | 0.00211 | 0.00013 |
| *3* | GO:0050911 | detection of chemical stimulus involved ... | 71 | 8 | 2.49 | 23 | 0.00323 | 0.00323 |
| *4* | GO:0003333 | amino acid transmembrane transport | 23 | 4 | 0.81 | 35 | 0.00776 | 0.00776 |
| *5* | GO:0034765 | regulation of ion transmembrane transpor... | 23 | 4 | 0.81 | 36 | 0.00776 | 0.00776 |

| | GO.ID | MFCluster: brown Size: 411 | Annotated | Significant | Expected | Rank in ClassicF | Weight01F | ClassicF |
|---|---|---|---|---|---|---|---|---|
| 1 | GO:0022843 | voltage−gated cation channel activity | 23 | 5 | 0.87 | 8 | 0.0026 | 0.00144 |
| 2 | GO:0005262 | calcium channel activity | 17 | 4 | 0.64 | 11 | 0.0032 | 0.00324 |
| 3 | GO:0000976 | transcription regulatory region sequence... | 45 | 8 | 1.70 | 1 | 0.0039 | 0.00024 |
| 4 | GO:0004984 | olfactory receptor activity | 69 | 8 | 2.61 | 14 | 0.0043 | 0.00430 |
| 5 | GO:0005549 | odorant binding | 118 | 11 | 4.47 | 15 | 0.0049 | 0.00494 |
| 6 | GO:0003700 | DNA−binding transcription factor activit... | 282 | 20 | 10.68 | 16 | 0.0049 | 0.00497 |
| 9 | GO:0004930 | G protein−coupled receptor activity | 171 | 14 | 6.48 | 17 | 0.0222 | 0.00526 |
| 18 | GO:0043565 | sequence−specific DNA binding | 212 | 18 | 8.03 | 7 | 0.0824 | 0.00108 |

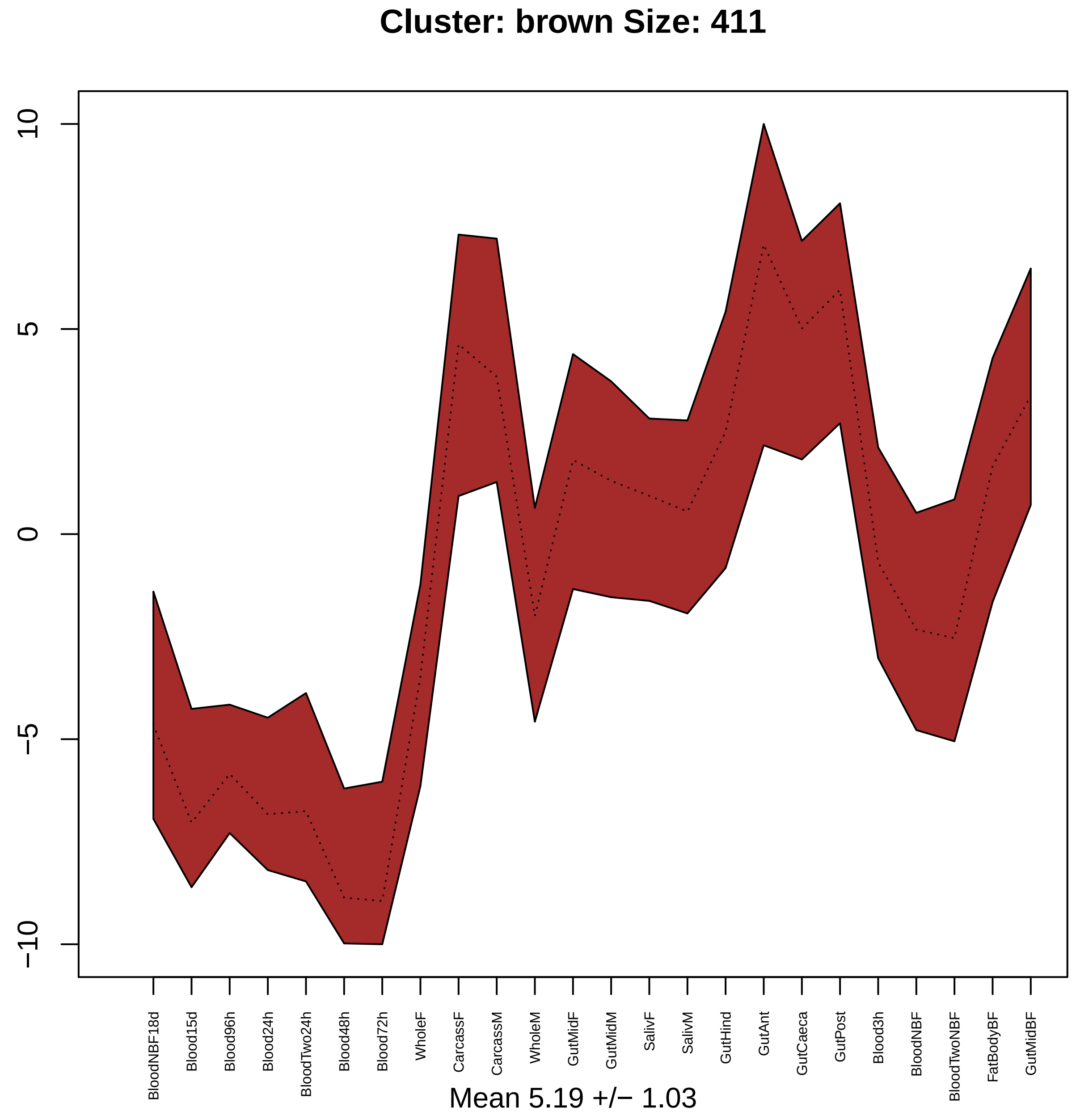
Cluster: brown Size: 411
10
5
0
−5
−10
BloodNBF18d
Blood15d
Blood96h
Blood24h
BloodTwo24h
Blood48h
Blood72h
WholeF
CarcassF
CarcassM
WholeM
GutMidF
GutMidM
SalivF
SalivM
GutHind
GutAnt
GutCaeca
GutPost
Blood3h
BloodNBF
BloodTwoNBF
FatBodyBF
GutMidBF
Mean 5.19 +/− 1.03

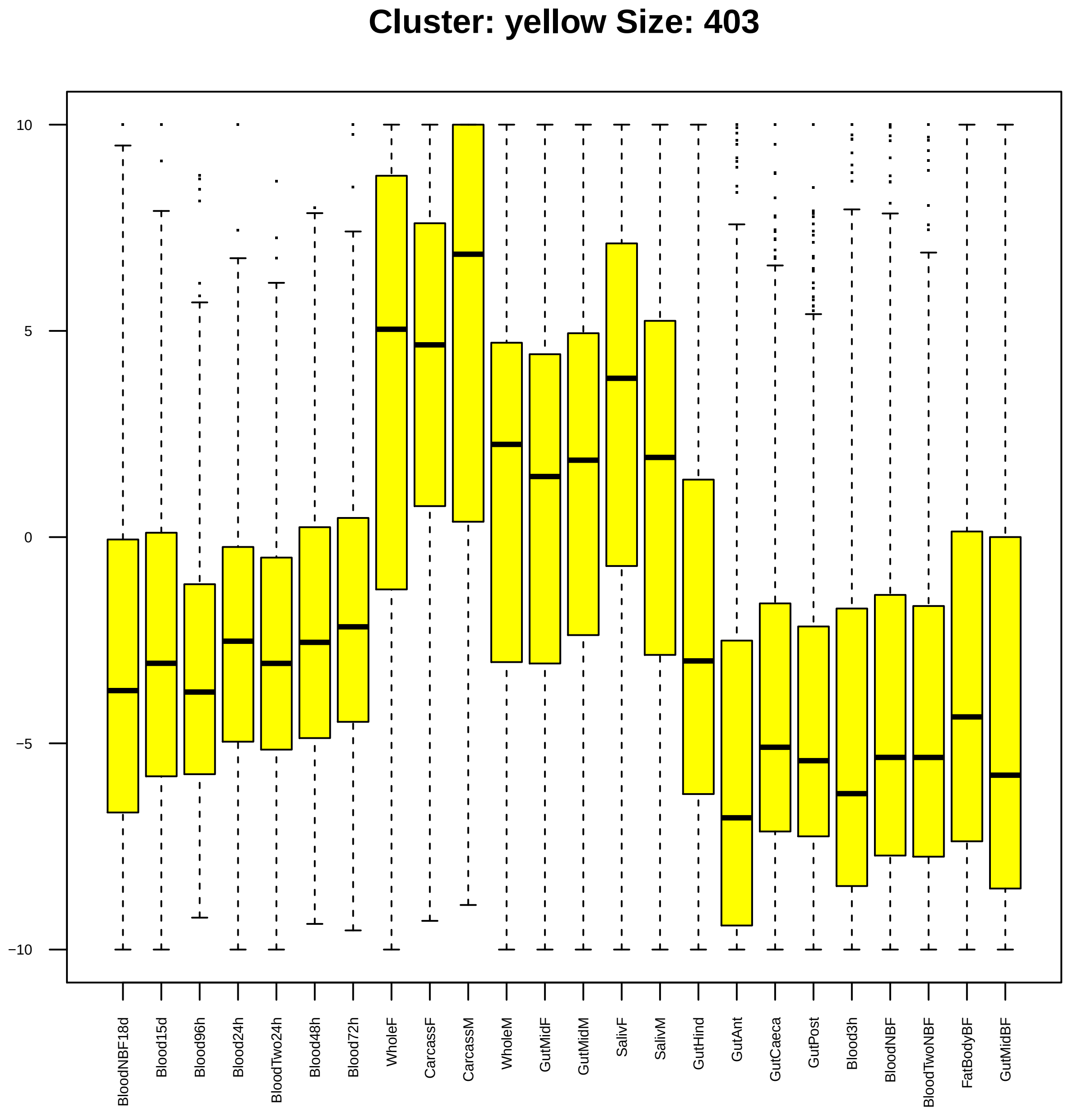
Cluster: yellow Size: 403
10
5
0
−5
−10
BloodNBF18d
Blood15d
Blood96h
Blood24h
BloodTwo24h
Blood48h
Blood72h
WholeF
CarcassF
CarcassM
WholeM
GutMidF
GutMidM
SalivF
SalivM
GutHind
GutAnt
GutCaeca
GutPost
Blood3h
BloodNBF
BloodTwoNBF
FatBodyBF
GutMidBF

| | GO.ID | BPCluster: yellow Size: 403 | Annotated | Significant | Expected | Rank in ClassicF | Weight01F | ClassicF |
|---|---|---|---|---|---|---|---|---|
| 1 | GO:0090502 | RNA phosphodiester bond hydrolysis, endo... | 14 | 5 | 0.59 | 16 | 0.00019 | 0.00019 |
| 2 | GO:0007095 | mitotic G2 DNA damage checkpoint | 43 | 8 | 1.81 | 20 | 0.00036 | 0.00036 |
| 3 | GO:0006511 | ubiquitin−dependent protein catabolic pr... | 88 | 9 | 3.71 | 80 | 0.00120 | 0.01144 |
| 4 | GO:0016236 | macroautophagy | 21 | 5 | 0.89 | 38 | 0.00150 | 0.00150 |
| 5 | GO:0016567 | protein ubiquitination | 55 | 8 | 2.32 | 46 | 0.00196 | 0.00196 |
| 6 | GO:0007033 | vacuole organization | 20 | 6 | 0.84 | 13 | 0.00346 | 0.00013 |
| 7 | GO:0006396 | RNA processing | 267 | 31 | 11.26 | 1 | 0.00697 | 2.0e−07 |
| 8 | GO:0042044 | fluid transport | 10 | 3 | 0.42 | 66 | 0.00714 | 0.00714 |
| 9 | GO:0033227 | dsRNA transport | 19 | 4 | 0.80 | 69 | 0.00727 | 0.00727 |
| 10 | GO:0048640 | negative regulation of developmental gro... | 28 | 5 | 1.18 | 59 | 0.00939 | 0.00569 |
| 11 | GO:0010883 | regulation of lipid storage | 11 | 3 | 0.46 | 77 | 0.00952 | 0.00952 |
| 15 | GO:0000398 | mRNA splicing, via spliceosome | 134 | 14 | 5.65 | 36 | 0.01398 | 0.00146 |

|  | GO.ID | MFCluster: yellow Size: 403 | Annotated | Significant | Expected | Rank in ClassicF | Weight01F | ClassicF |
|---|---|---|---|---|---|---|---|---|
| *1* | GO:0016891 | endoribonuclease activity, producing 5'−... | 11 | 4 | 0.48 | 4 | 0.0009 | 0.00090 |
| *2* | GO:0005548 | phospholipid transporter activity | 10 | 3 | 0.43 | 16 | 0.0077 | 0.00775 |
| *6* | GO:0003723 | RNA binding | 307 | 26 | 13.33 | 2 | 0.0164 | 0.00078 |
| *7* | GO:0005488 | binding | 4627 | 226 | 200.89 | 5 | 0.0191 | 0.00129 |
| *10* | GO:0016874 | ligase activity | 161 | 15 | 6.99 | 11 | 0.0267 | 0.00416 |

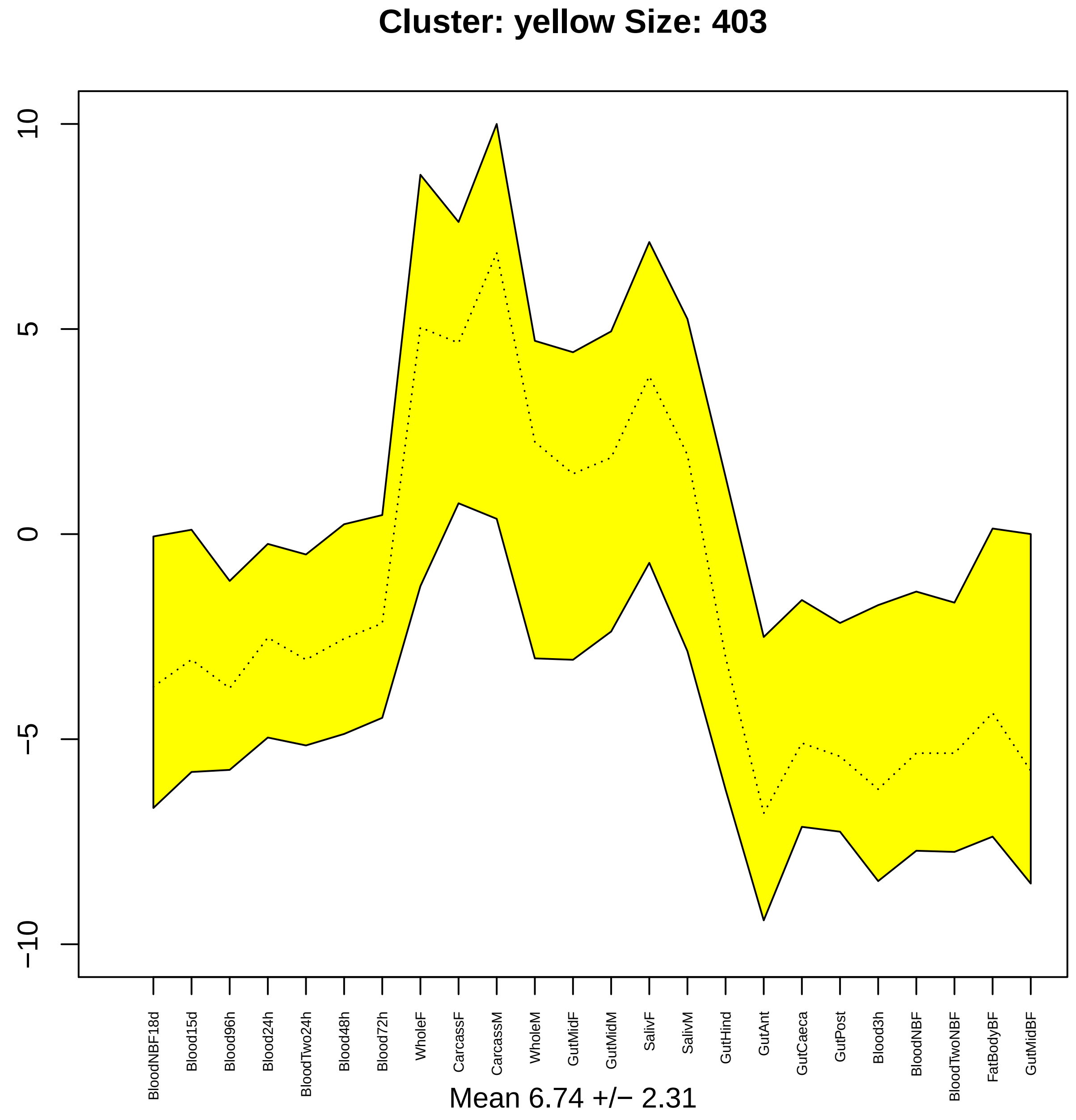

Cluster: yellow Size: 403
10
5
0
−5
−10
BloodNBF18d
Blood15d
Blood96h
Blood24h
BloodTwo24h
Blood48h
Blood72h
WholeF
CarcassF
CarcassM
WholeM
GutMidF
GutMidM
SalivF
SalivM
GutHind
GutAnt
GutCaeca
GutPost
Blood3h
BloodNBF
BloodTwoNBF
FatBodyBF
GutMidBF
Mean 6.74 +/− 2.31

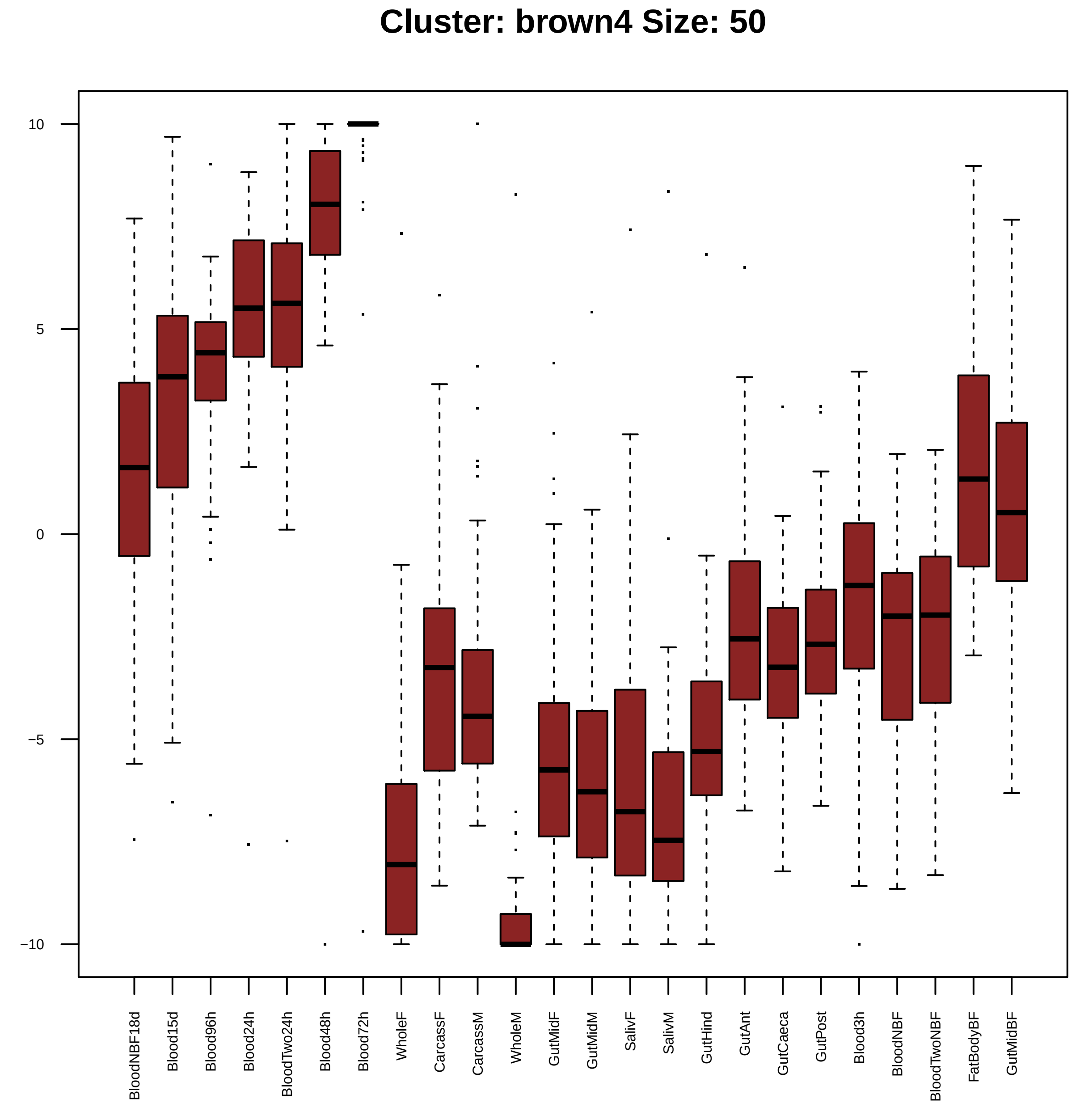
Cluster: brown4 Size: 50
10
5
0
−5
−10
BloodNBF18d
Blood15d
Blood96h
Blood24h
BloodTwo24h
Blood48h
Blood72h
WholeF
CarcassF
CarcassM
WholeM
GutMidF
GutMidM
SalivF
SalivM
GutHind
GutAnt
GutCaeca
GutPost
Blood3h
BloodNBF
BloodTwoNBF
FatBodyBF
GutMidBF

| | GO.ID | BPCluster: brown4 Size: 50 | Annotated | Significant | Expected | Rank in ClassicF | Weight01F | ClassicF |
|---|---|---|---|---|---|---|---|---|
| *1* | GO:0007062 | sister chromatid cohesion | 13 | 2 | 0.06 | 38 | 0.0013 | 0.0013 |
| *2* | GO:0006355 | regulation of transcription, DNA−templat... | 501 | 10 | 2.12 | 8 | 0.0014 | 2e−05 |
| *3* | GO:0000122 | negative regulation of transcription by ... | 57 | 3 | 0.24 | 46 | 0.0017 | 0.0017 |
| *4* | GO:0043065 | positive regulation of apoptotic process | 21 | 2 | 0.09 | 56 | 0.0035 | 0.0035 |
| *5* | GO:0045944 | positive regulation of transcription by ... | 88 | 3 | 0.37 | 69 | 0.0058 | 0.0058 |
| *7* | GO:0000082 | G1/S transition of mitotic cell cycle | 23 | 2 | 0.10 | 59 | 0.0283 | 0.0041 |

| | GO.ID | MFCluster: brown4 Size: 50 | Annotated | Significant | Expected | Rank in ClassicF | Weight01F | ClassicF |
|---|---|---|---|---|---|---|---|---|
| 2 | GO:0016896 | exoribonuclease activity, producing 5'−p... | 11 | 2 | 0.06 | 6 | 0.0015 | 0.00152 |
| 3 | GO:0001077 | proximal promoter DNA−binding transcript... | 17 | 2 | 0.09 | 9 | 0.0037 | 0.00368 |
| 4 | GO:0003676 | nucleic acid binding | 1152 | 15 | 6.23 | 4 | 0.0040 | 0.00058 |
| 5 | GO:0016817 | hydrolase activity, acting on acid anhyd... | 442 | 3 | 2.39 | 67 | 0.0055 | 0.43144 |
| 6 | GO:0003682 | chromatin binding | 69 | 3 | 0.37 | 15 | 0.0060 | 0.00600 |
| 7 | GO:0001012 | RNA polymerase II regulatory region DNA ... | 26 | 2 | 0.14 | 18 | 0.0106 | 0.00854 |
| 8 | GO:0043565 | sequence−specific DNA binding | 212 | 5 | 1.15 | 13 | 0.0107 | 0.00535 |
| 9 | GO:0003700 | DNA−binding transcription factor activit... | 282 | 6 | 1.53 | 10 | 0.0215 | 0.00373 |
| 10 | GO:0005488 | binding | 4627 | 35 | 25.03 | 3 | 0.0271 | 0.00029 |
| 21 | GO:0003677 | DNA binding | 523 | 8 | 2.83 | 16 | 0.1767 | 0.00602 |

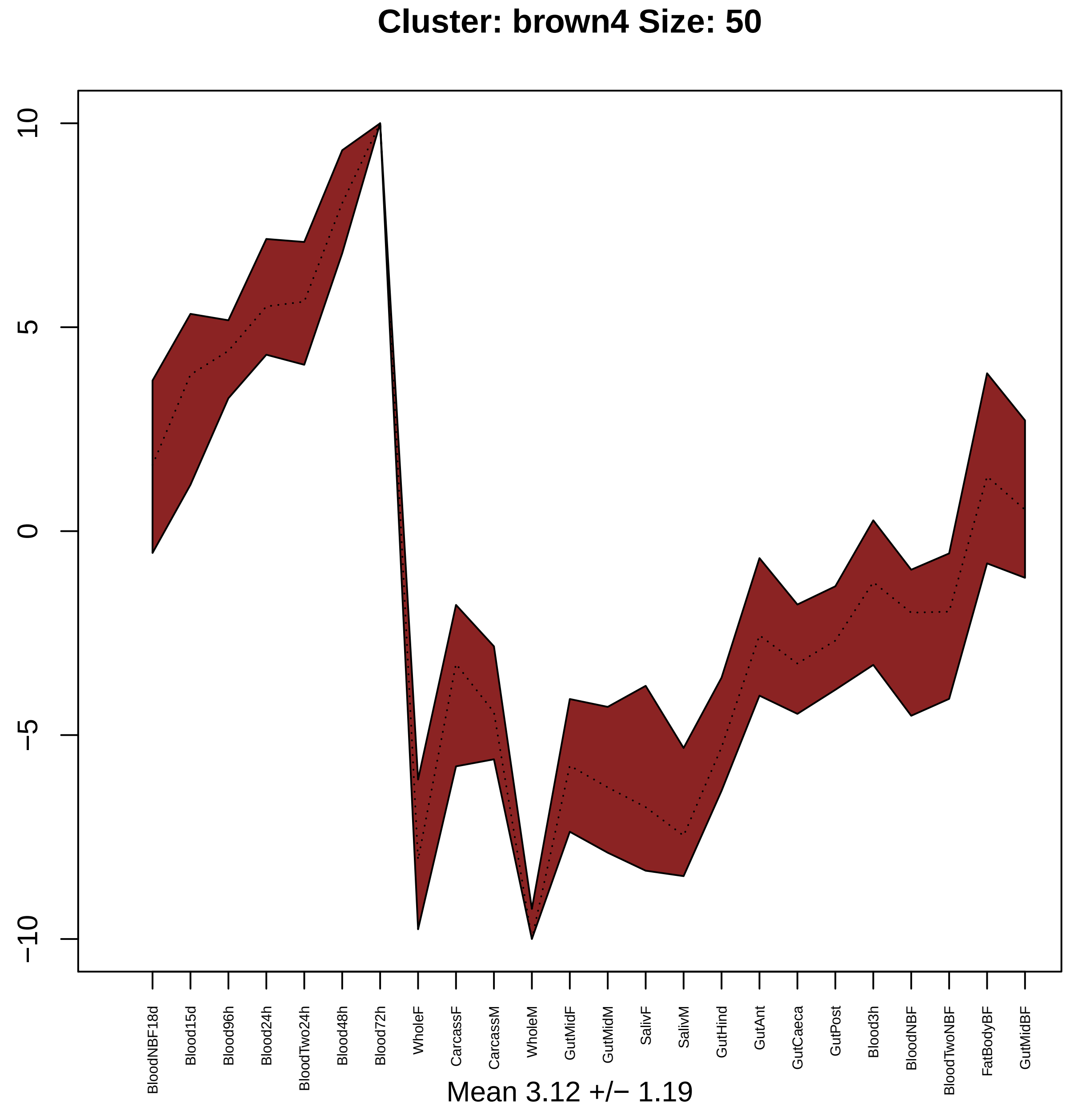

Cluster: brown4 Size: 50
10
5
0
−5
−10
BloodNBF18d
Blood15d
Blood96h
Blood24h
BloodTwo24h
Blood48h
Blood72h
WholeF
CarcassF
CarcassM
WholeM
GutMidF
GutMidM
SalivF
SalivM
GutHind
GutAnt
GutCaeca
GutPost
Blood3h
BloodNBF
BloodTwoNBF
FatBodyBF
GutMidBF
Mean 3.12 +/− 1.19

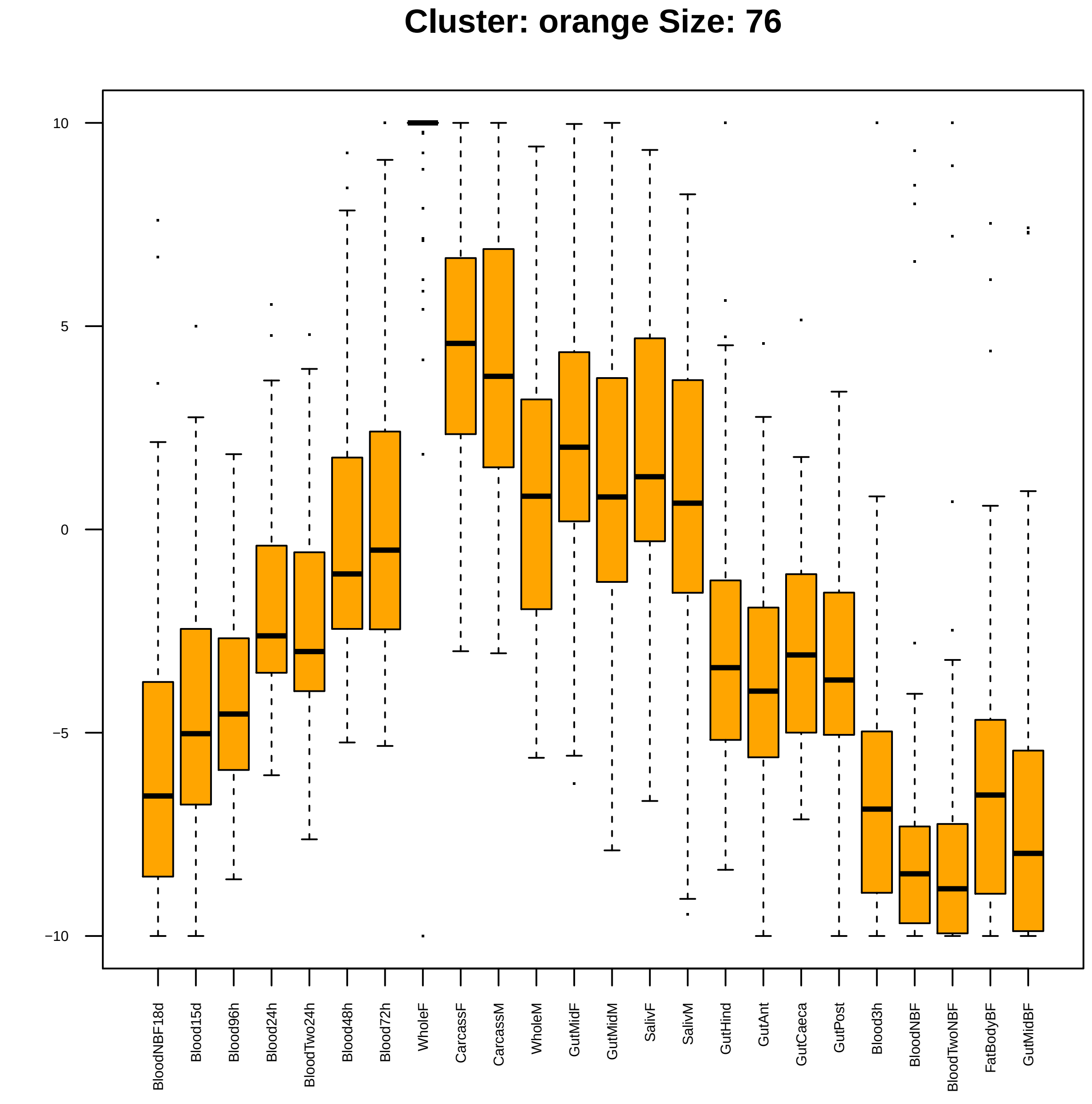
Cluster: orange Size: 76
10
5
0
−5
−10
BloodNBF18d
Blood15d
Blood96h
Blood24h
BloodTwo24h
Blood48h
Blood72h
WholeF
CarcassF
CarcassM
WholeM
GutMidF
GutMidM
SalivF
SalivM
GutHind
GutAnt
GutCaeca
GutPost
Blood3h
BloodNBF
BloodTwoNBF
FatBodyBF
GutMidBF

| | GO.ID | BPCluster: orange Size: 76 | Annotated | Significant | Expected | Rank in ClassicF | Weight01F | ClassicF |
|---|---|---|---|---|---|---|---|---|
| *1* | GO:0006406 | mRNA export from nucleus | 13 | 3 | 0.10 | 2 | 0.0001 | 0.00010 |
| *2* | GO:0065004 | protein−DNA complex assembly | 29 | 3 | 0.21 | 19 | 0.0032 | 0.00120 |
| *3* | GO:0051726 | regulation of cell cycle | 180 | 5 | 1.33 | 47 | 0.0045 | 0.00996 |
| *4* | GO:0045814 | negative regulation of gene expression, ... | 27 | 3 | 0.20 | 16 | 0.0071 | 0.00097 |
| *5* | GO:0006260 | DNA replication | 77 | 4 | 0.57 | 24 | 0.0134 | 0.00237 |
| *8* | GO:0071824 | protein−DNA complex subunit organization | 40 | 4 | 0.29 | 4 | 0.0275 | 0.00019 |
| *9* | GO:0060968 | regulation of gene silencing | 19 | 2 | 0.14 | 43 | 0.0356 | 0.00839 |
| *10* | GO:0044786 | cell cycle DNA replication | 18 | 2 | 0.13 | 41 | 0.0426 | 0.00755 |

| | GO.ID | MFCluster: orange Size: 76 | Annotated | Significant | Expected | Rank in ClassicF | Weight01F | ClassicF |
|---|---|---|---|---|---|---|---|---|
| *1* | GO:0003676 | nucleic acid binding | 1152 | 19 | 9.27 | 1 | 0.00073 | 0.0012 |
| *2* | GO:0008270 | zinc ion binding | 522 | 11 | 4.20 | 2 | 0.00259 | 0.0026 |

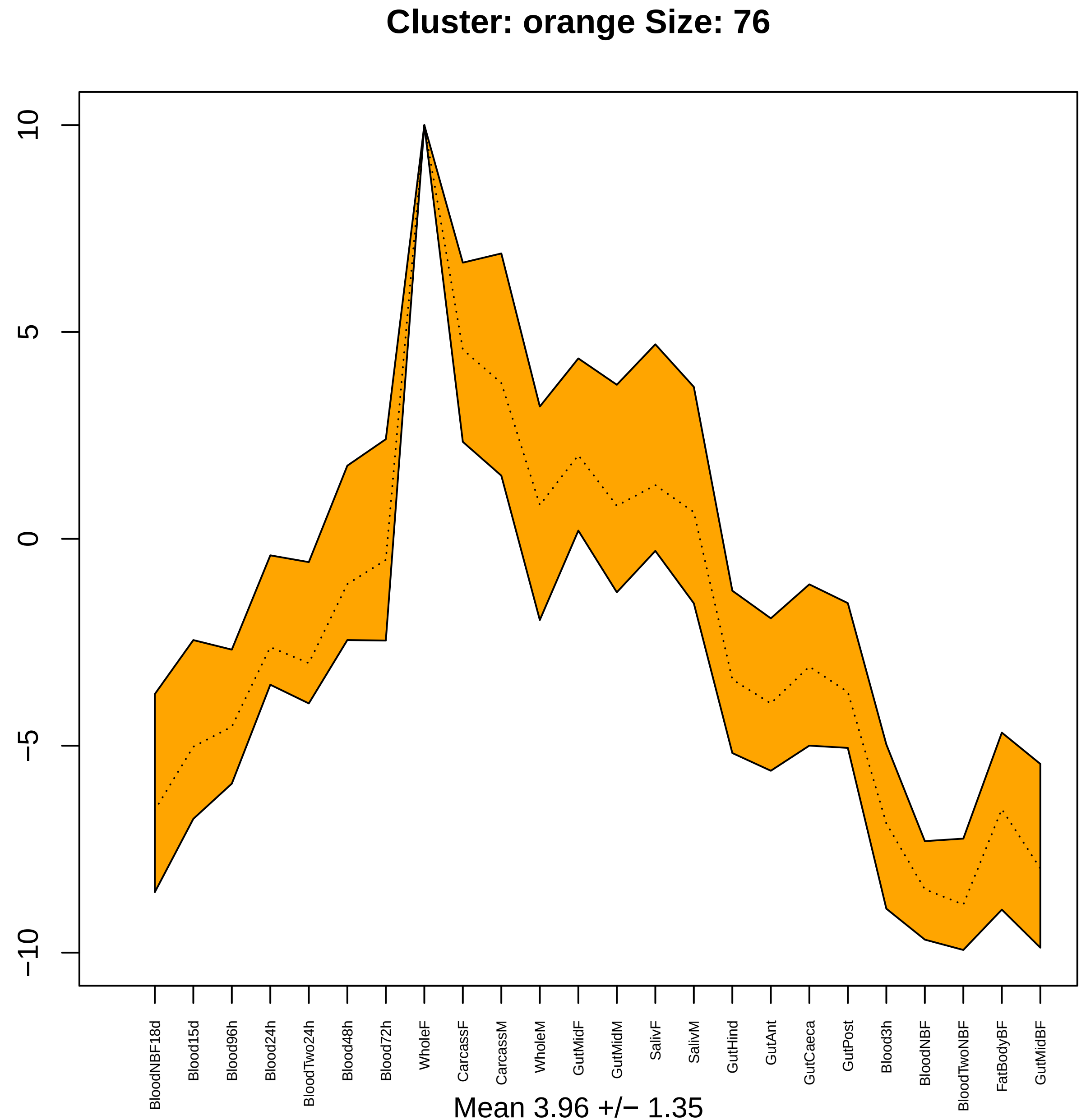

Cluster: orange Size: 76
10
5
0
−5
−10
BloodNBF18d
Blood15d
Blood96h
Blood24h
BloodTwo24h
Blood48h
Blood72h
WholeF
CarcassF
CarcassM
WholeM
GutMidF
GutMidM
SalivF
SalivM
GutHind
GutAnt
GutCaeca
GutPost
Blood3h
BloodNBF
BloodTwoNBF
FatBodyBF
GutMidBF
Mean 3.96 +/− 1.35

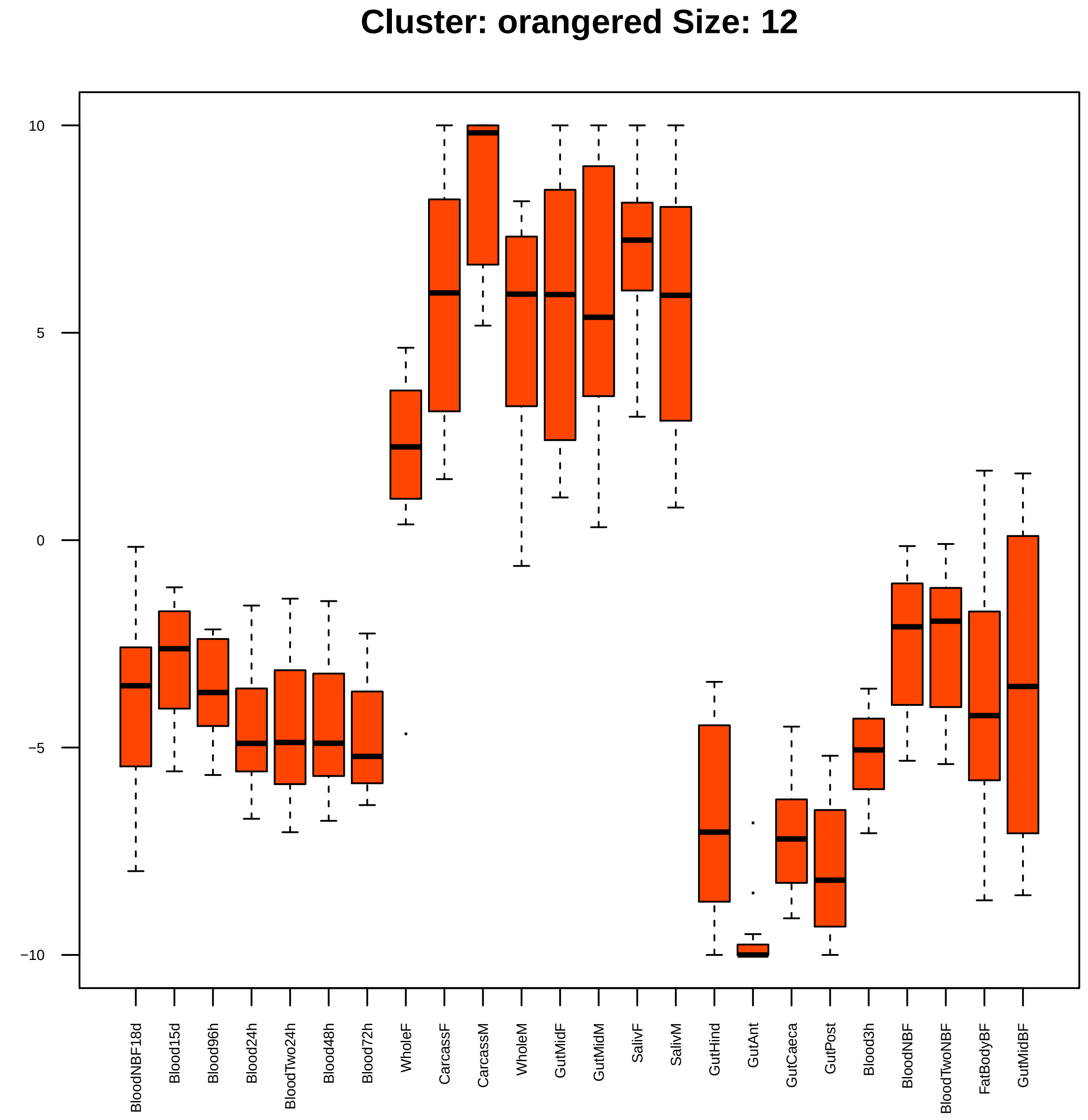

Cluster: orangered Size: 12
10
5
0
−5
−10
BloodNBF18d
Blood15d
Blood96h
Blood24h
BloodTwo24h
Blood48h
Blood72h
WholeF
CarcassF
CarcassM
WholeM
GutMidF
GutMidM
SalivF
SalivM
GutHind
GutAnt
GutCaeca
GutPost
Blood3h
BloodNBF
BloodTwoNBF
FatBodyBF
GutMidBF

| | GO.ID | BPCluster: orangered Size: 12 | Annotated | Significant | Expected | Rank in ClassicF | Weight01F | ClassicF |
|---|---|---|---|---|---|---|---|---|
| *13* | GO:0044262 | cellular carbohydrate metabolic process | 39 | 2 | 0.05 | 1 | 0.023 | 0.001 |

|  | GO.ID | MFCluster: orangered Size: 12 | Annotated | Significant | Expected | Rank in ClassicF | Weight01F | ClassicF |
|---|---|---|---|---|---|---|---|---|
| 1 | GO:0019887 | protein kinase regulator activity | 29 | 2 | 0.04 | 1 | 0.00055 | 0.00055 |

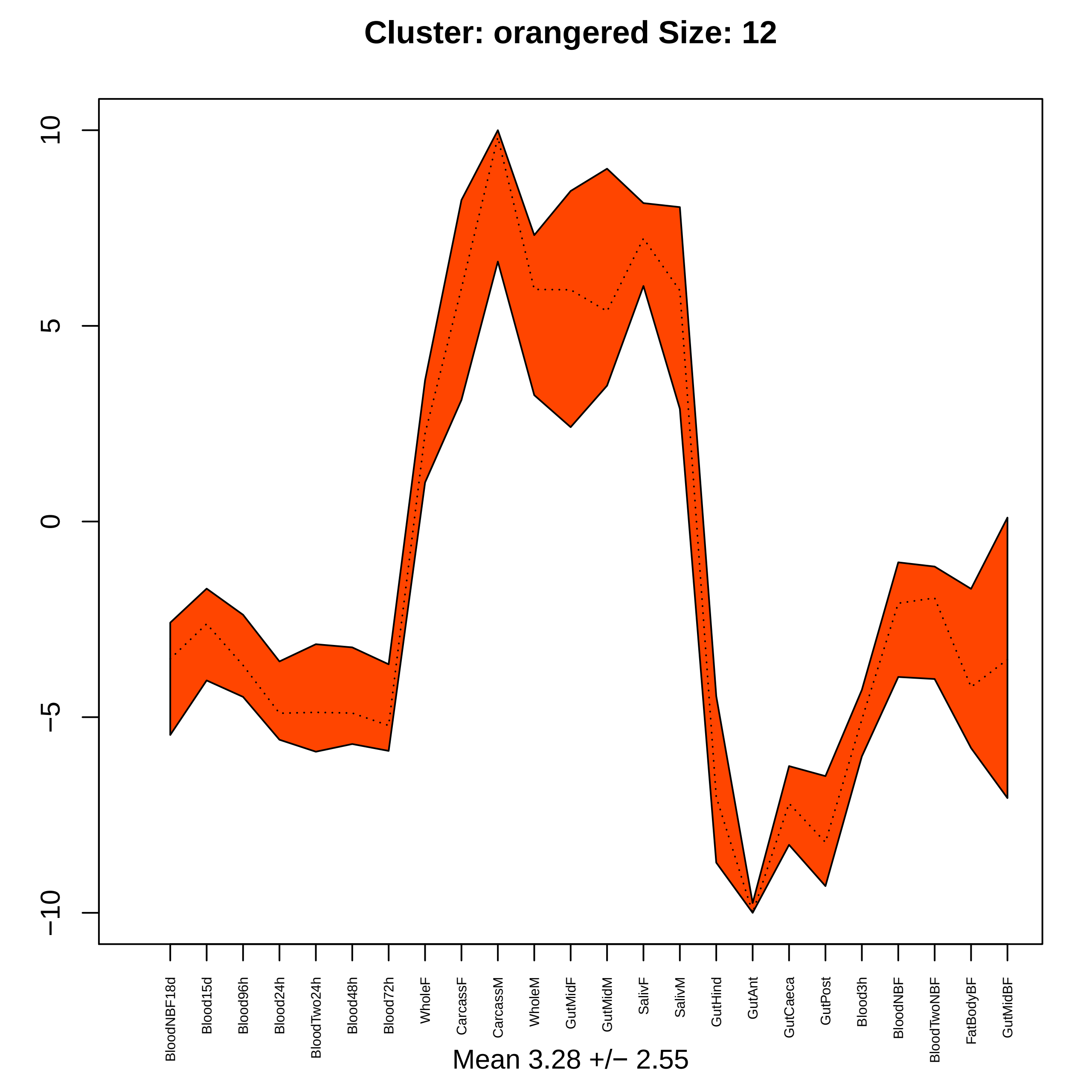
Cluster: orangered Size: 12
10
5
0
−5
−10
BloodNBF18d
Blood15d
Blood96h
Blood24h
BloodTwo24h
Blood48h
Blood72h
WholeF
CarcassF
CarcassM
WholeM
GutMidF
GutMidM
SalivF
SalivM
GutHind
GutAnt
GutCaeca
GutPost
Blood3h
BloodNBF
BloodTwoNBF
FatBodyBF
GutMidBF
Mean 3.28 +/− 2.55

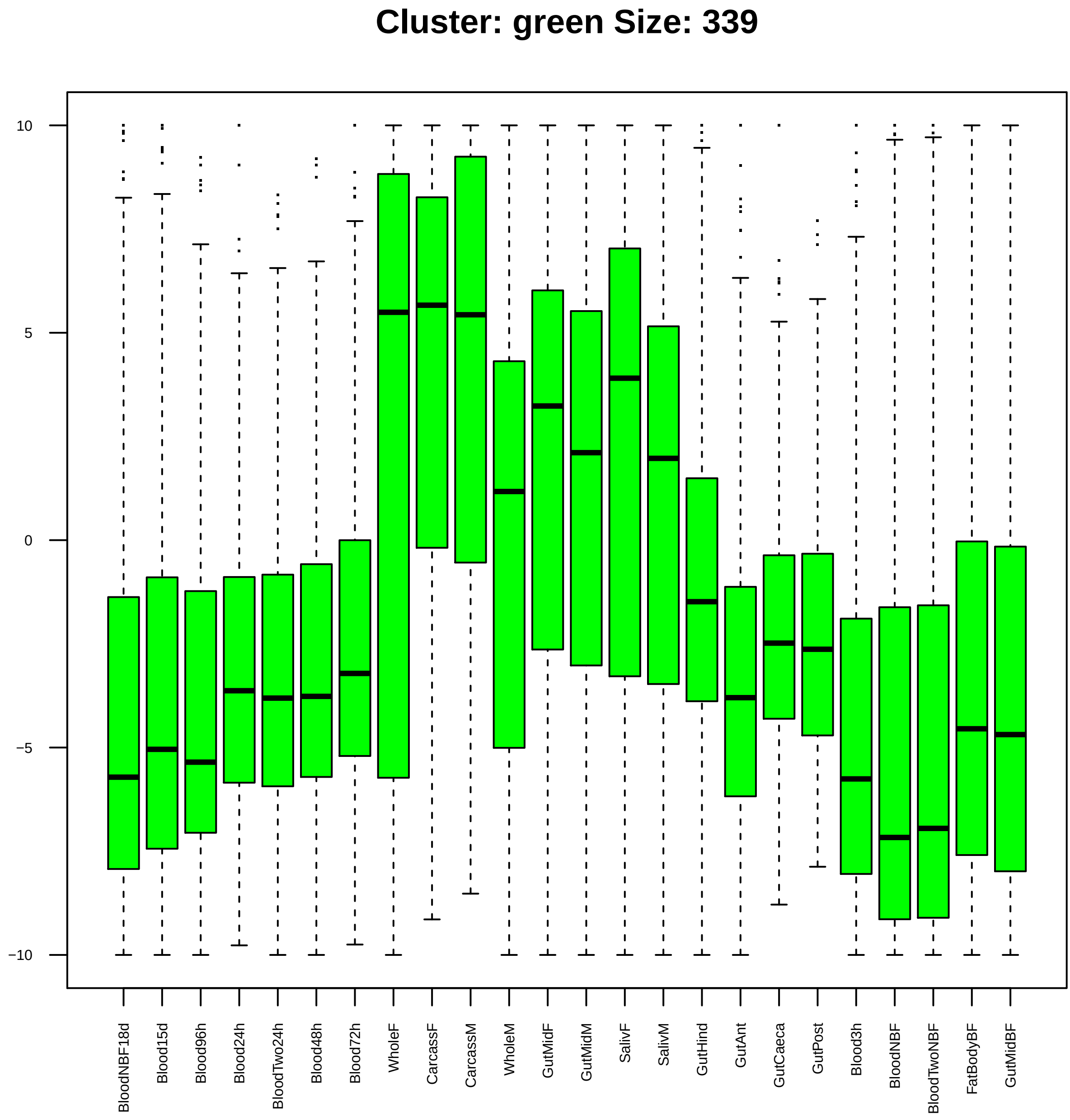

Cluster: green Size: 339
10
5
0
−5
−10
BloodNBF18d
Blood15d
Blood96h
Blood24h
BloodTwo24h
Blood48h
Blood72h
WholeF
CarcassF
CarcassM
WholeM
GutMidF
GutMidM
SalivF
SalivM
GutHind
GutAnt
GutCaeca
GutPost
Blood3h
BloodNBF
BloodTwoNBF
FatBodyBF
GutMidBF

| | GO.ID | BPCluster: green Size: 339 | Annotated | Significant | Expected | Rank in ClassicF | Weight01F | ClassicF |
|---|---|---|---|---|---|---|---|---|
| *3* | GO:0015031 | protein transport | 242 | 18 | 8.88 | 36 | 0.0022 | 0.00325 |
| *4* | GO:0001510 | RNA methylation | 17 | 4 | 0.62 | 34 | 0.0029 | 0.00288 |
| *5* | GO:0016319 | mushroom body development | 28 | 5 | 1.03 | 35 | 0.0031 | 0.00314 |
| *6* | GO:0042254 | ribosome biogenesis | 64 | 7 | 2.35 | 55 | 0.0042 | 0.00857 |
| *7* | GO:0006891 | intra−Golgi vesicle−mediated transport | 10 | 3 | 0.37 | 44 | 0.0048 | 0.00483 |
| *8* | GO:0000723 | telomere maintenance | 10 | 3 | 0.37 | 45 | 0.0048 | 0.00483 |
| *9* | GO:0000381 | regulation of alternative mRNA splicing,... | 32 | 5 | 1.17 | 50 | 0.0057 | 0.00570 |
| *10* | GO:0000398 | mRNA splicing, via spliceosome | 134 | 14 | 4.92 | 27 | 0.0058 | 0.00037 |
| *11* | GO:0006281 | DNA repair | 89 | 9 | 3.27 | 49 | 0.0098 | 0.00510 |
| *19* | GO:0043484 | regulation of RNA splicing | 38 | 6 | 1.39 | 33 | 0.0361 | 0.00236 |

|  | GO.ID | MFCluster: green Size: 339 | Annotated | Significant | Expected | Rank in ClassicF | Weight01F | ClassicF |
|---|---|---|---|---|---|---|---|---|
| 3 | GO:0008173 | RNA methyltransferase activity | 19 | 5 | 0.67 | 18 | 0.0004 | 0.00040 |
| 4 | GO:0005524 | ATP binding | 592 | 35 | 20.86 | 20 | 0.0015 | 0.00153 |
| 5 | GO:0003676 | nucleic acid binding | 1152 | 62 | 40.59 | 17 | 0.0018 | 0.00027 |
| 6 | GO:0008408 | 3'−5' exonuclease activity | 18 | 4 | 0.63 | 26 | 0.0031 | 0.00312 |
| 7 | GO:0015297 | antiporter activity | 10 | 3 | 0.35 | 29 | 0.0043 | 0.00432 |
| 8 | GO:0000049 | tRNA binding | 10 | 3 | 0.35 | 30 | 0.0043 | 0.00432 |
| 9 | GO:0016706 | oxidoreductase activity, acting on paire... | 12 | 3 | 0.42 | 32 | 0.0075 | 0.00751 |
| 13 | GO:0004518 | nuclease activity | 66 | 8 | 2.33 | 23 | 0.0224 | 0.00208 |
| 14 | GO:0016796 | exonuclease activity, active with either... | 18 | 4 | 0.63 | 27 | 0.0228 | 0.00312 |
| 18 | GO:0019001 | guanyl nucleotide binding | 137 | 11 | 4.83 | 33 | 0.0345 | 0.00880 |

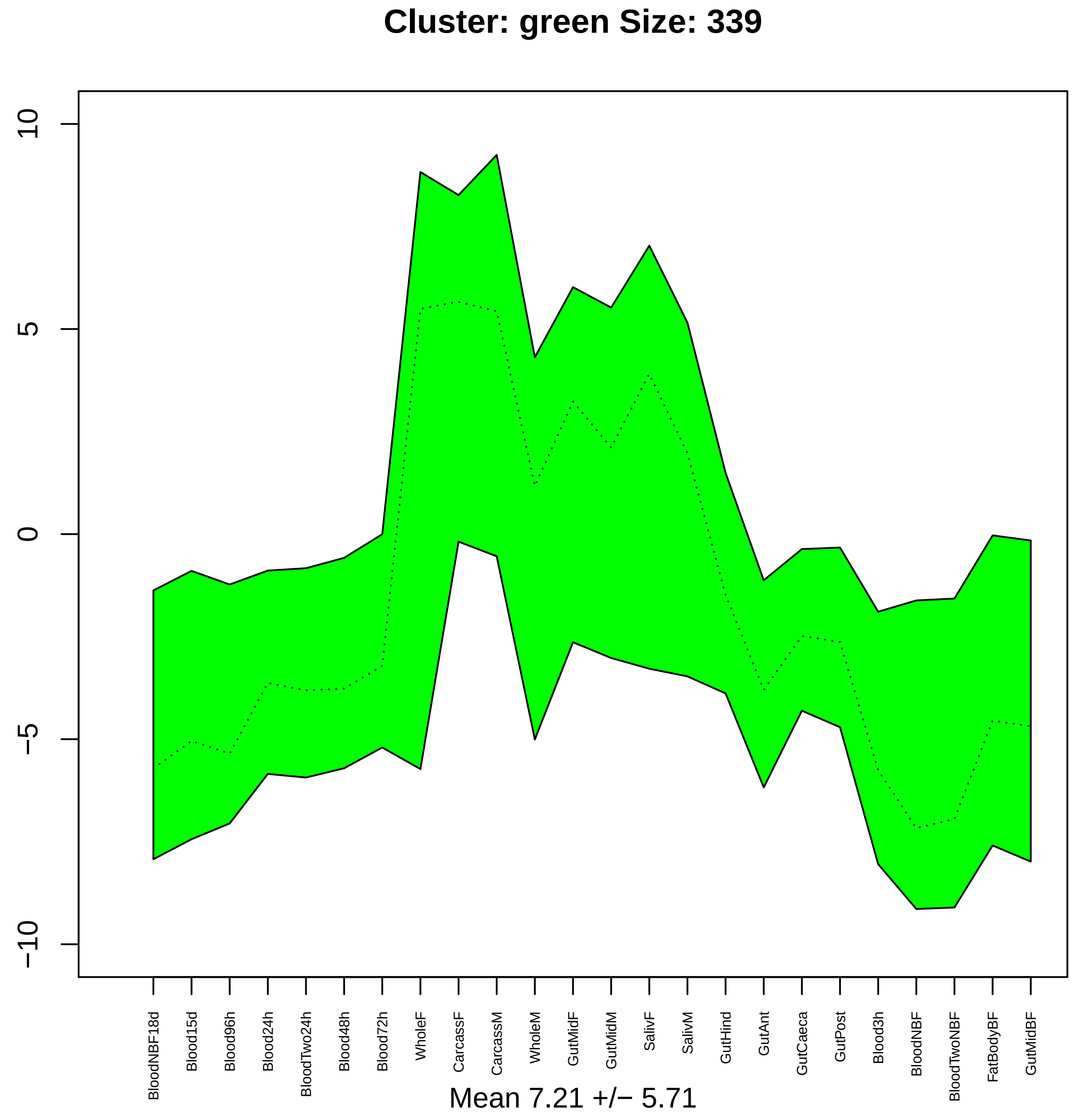
Cluster: green Size: 339
10
5
0
−5
−10
BloodNBF18d
Blood15d
Blood96h
Blood24h
BloodTwo24h
Blood48h
Blood72h
WholeF
CarcassF
CarcassM
WholeM
GutMidF
GutMidM
SalivF
SalivM
GutHind
GutAnt
GutCaeca
GutPost
Blood3h
BloodNBF
BloodTwoNBF
FatBodyBF
GutMidBF
Mean 7.21 +/− 5.71

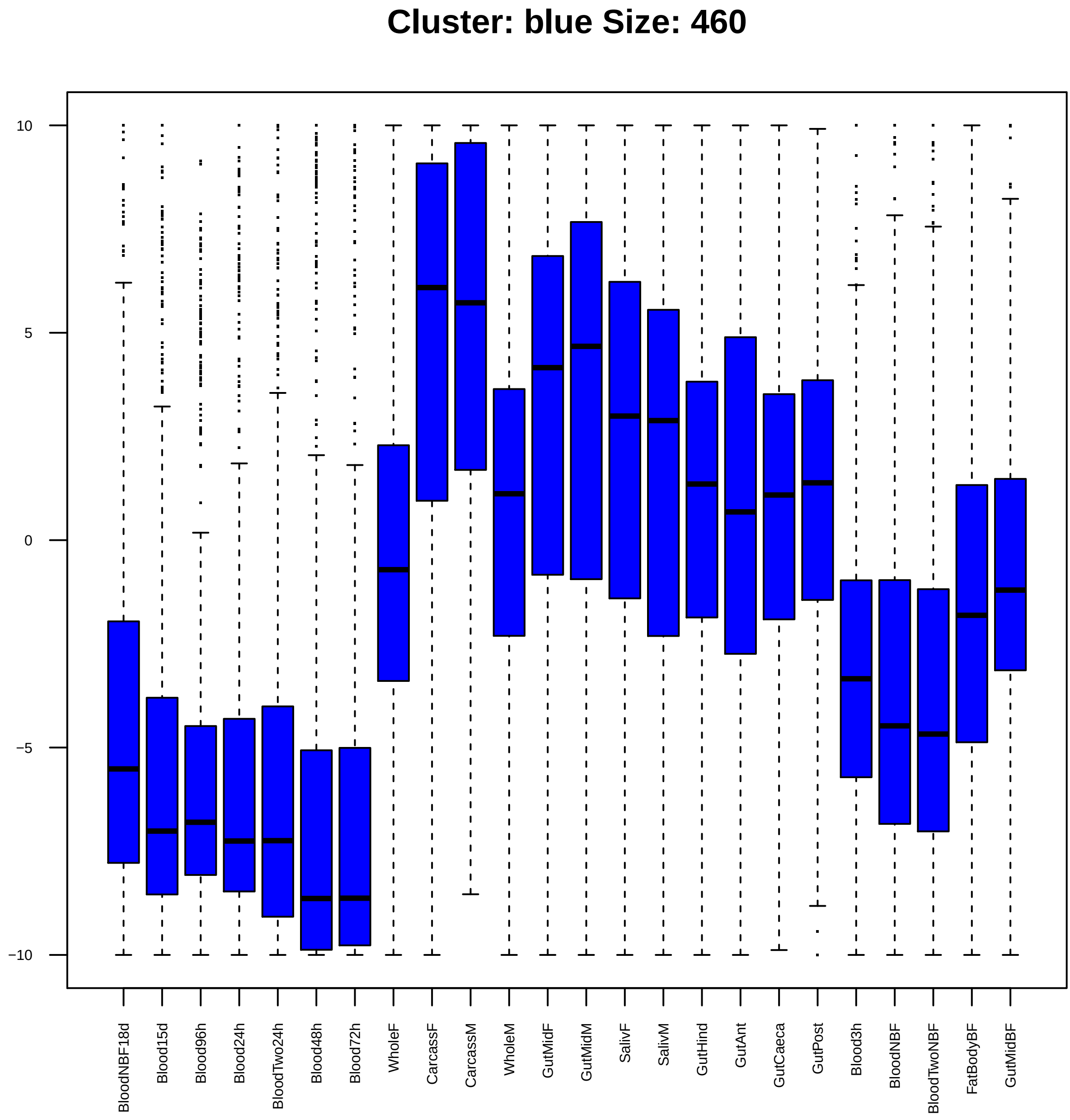
Cluster: blue Size: 460
10
5
0
−5
−10
BloodNBF18d
Blood15d
Blood96h
Blood24h
BloodTwo24h
Blood48h
Blood72h
WholeF
CarcassF
CarcassM
WholeM
GutMidF
GutMidM
SalivF
SalivM
GutHind
GutAnt
GutCaeca
GutPost
Blood3h
BloodNBF
BloodTwoNBF
FatBodyBF
GutMidBF

| | GO.ID | BPCluster: blue Size: 460 | Annotated | Significant | Expected | Rank in ClassicF | Weight01F | ClassicF |
|---|---|---|---|---|---|---|---|---|
| *1* | GO:0008045 | motor neuron axon guidance | 27 | 6 | 1.16 | 1 | 0.00082 | 0.00082 |
| *2* | GO:1901565 | organonitrogen compound catabolic proces... | 194 | 12 | 8.34 | 146 | 0.00205 | 0.12949 |
| *3* | GO:0060070 | canonical Wnt signaling pathway | 18 | 4 | 0.77 | 5 | 0.00633 | 0.00633 |
| *4* | GO:0042749 | regulation of circadian sleep/wake cycle | 10 | 3 | 0.43 | 6 | 0.00752 | 0.00752 |
| *29* | GO:0000902 | cell morphogenesis | 335 | 24 | 14.39 | 9 | 0.06867 | 0.00906 |

| | GO.ID | MFCluster: blue Size: 460 | Annotated | Significant | Expected | Rank in ClassicF | Weight01F | ClassicF |
|---|---|---|---|---|---|---|---|---|
| *1* | GO:0016746 | transferase activity, transferring acyl ... | 141 | 10 | 6.02 | 24 | 0.0012 | 0.0790 |
| *2* | GO:0001664 | G protein−coupled receptor binding | 16 | 4 | 0.68 | 1 | 0.0040 | 0.0040 |
| *3* | GO:0005506 | iron ion binding | 137 | 13 | 5.85 | 2 | 0.0056 | 0.0056 |
| *4* | GO:0004497 | monooxygenase activity | 110 | 11 | 4.70 | 3 | 0.0071 | 0.0071 |
| *5* | GO:0046983 | protein dimerization activity | 136 | 9 | 5.81 | 37 | 0.0075 | 0.1271 |

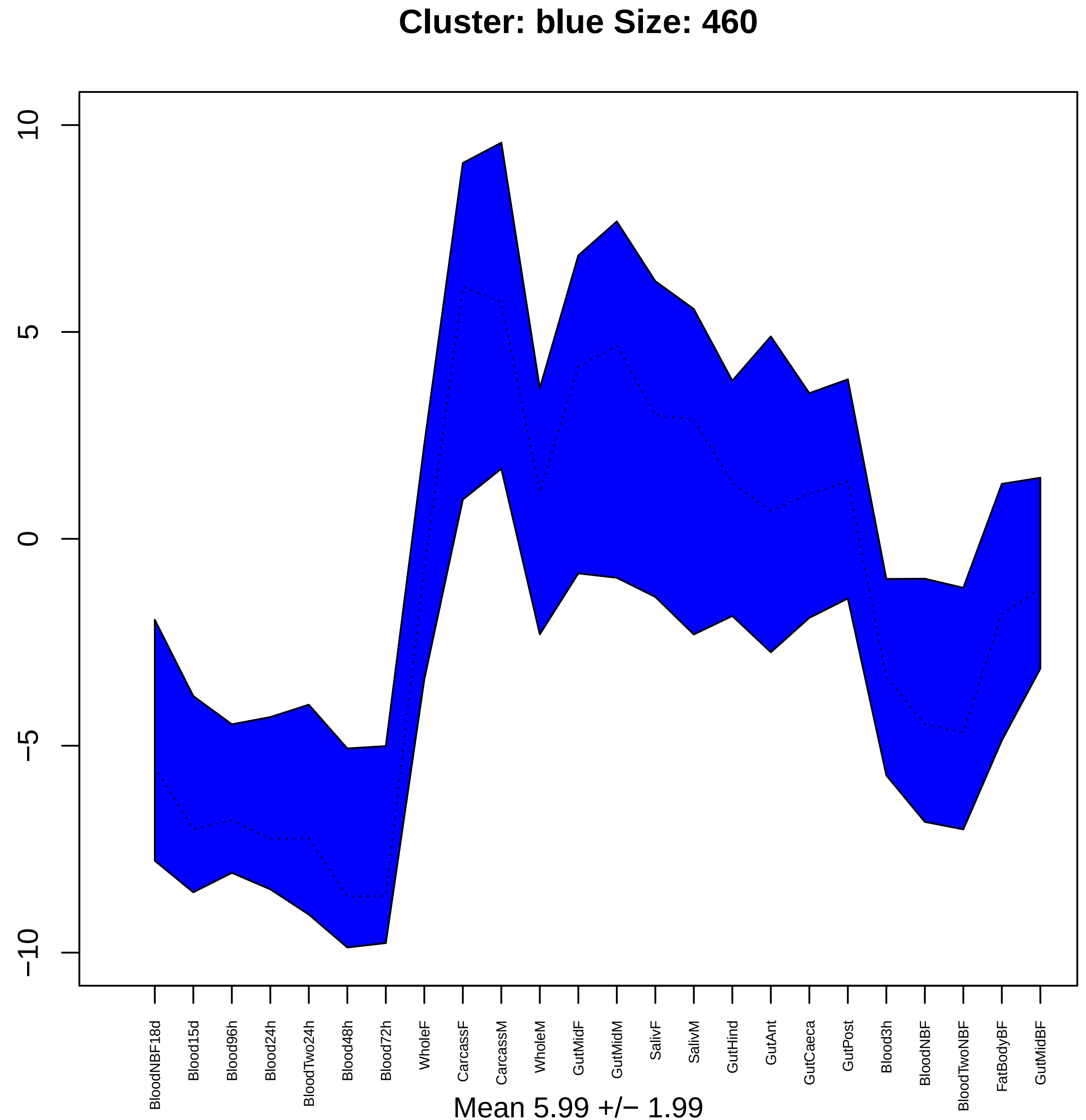

Cluster: blue Size: 460
10
5
0
−5
−10
BloodNBF18d
Blood15d
Blood96h
Blood24h
BloodTwo24h
Blood48h
Blood72h
WholeF
CarcassF
CarcassM
WholeM
GutMidF
GutMidM
SalivF
SalivM
GutHind
GutAnt
GutCaeca
GutPost
Blood3h
BloodNBF
BloodTwoNBF
FatBodyBF
GutMidBF
Mean 5.99 +/− 1.99

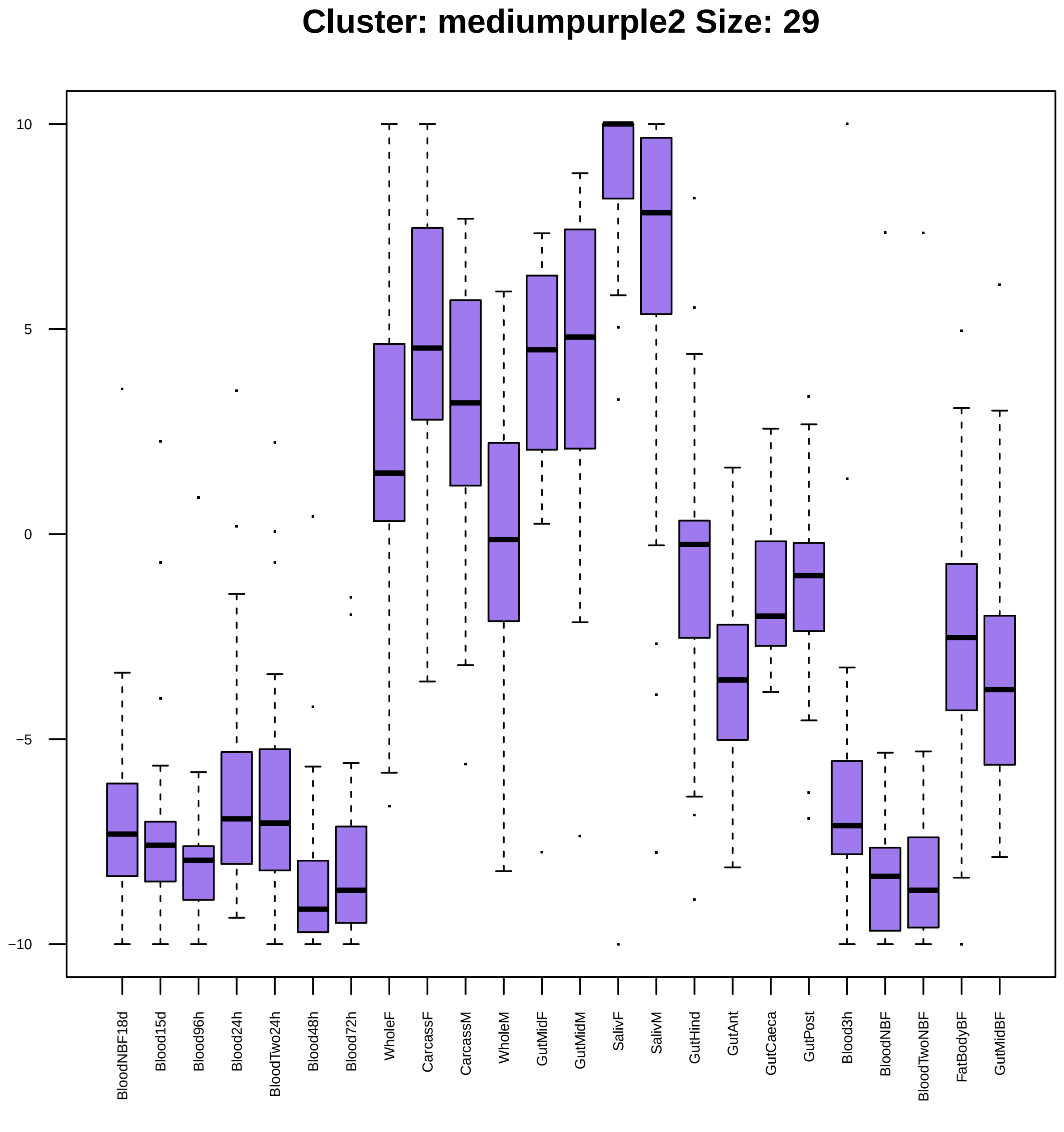
Cluster: mediumpurple2 Size: 29
10
5
0
−5
−10
BloodNBF18d
Blood15d
Blood96h
Blood24h
BloodTwo24h
Blood48h
Blood72h
WholeF
CarcassF
CarcassM
WholeM
GutMidF
GutMidM
SalivF
SalivM
GutHind
GutAnt
GutCaeca
GutPost
Blood3h
BloodNBF
BloodTwoNBF
FatBodyBF
GutMidBF

| | GO.ID | BPCluster: mediumpurple2 Size: 29 | Annotated | Significant | Expected | Rank in ClassicF | Weight01F | ClassicF |
|---|---|---|---|---|---|---|---|---|
| *3* | GO:0000022 | mitotic spindle elongation | 46 | 3 | 0.17 | 9 | 0.00056 | 0.00056 |
| *4* | GO:0006412 | translation | 289 | 7 | 1.04 | 4 | 0.00071 | 4.8e−05 |
| *5* | GO:0007030 | Golgi organization | 37 | 2 | 0.13 | 29 | 0.00768 | 0.00768 |
| *20* | GO:0010243 | response to organonitrogen compound | 37 | 2 | 0.13 | 30 | 0.05730 | 0.00768 |

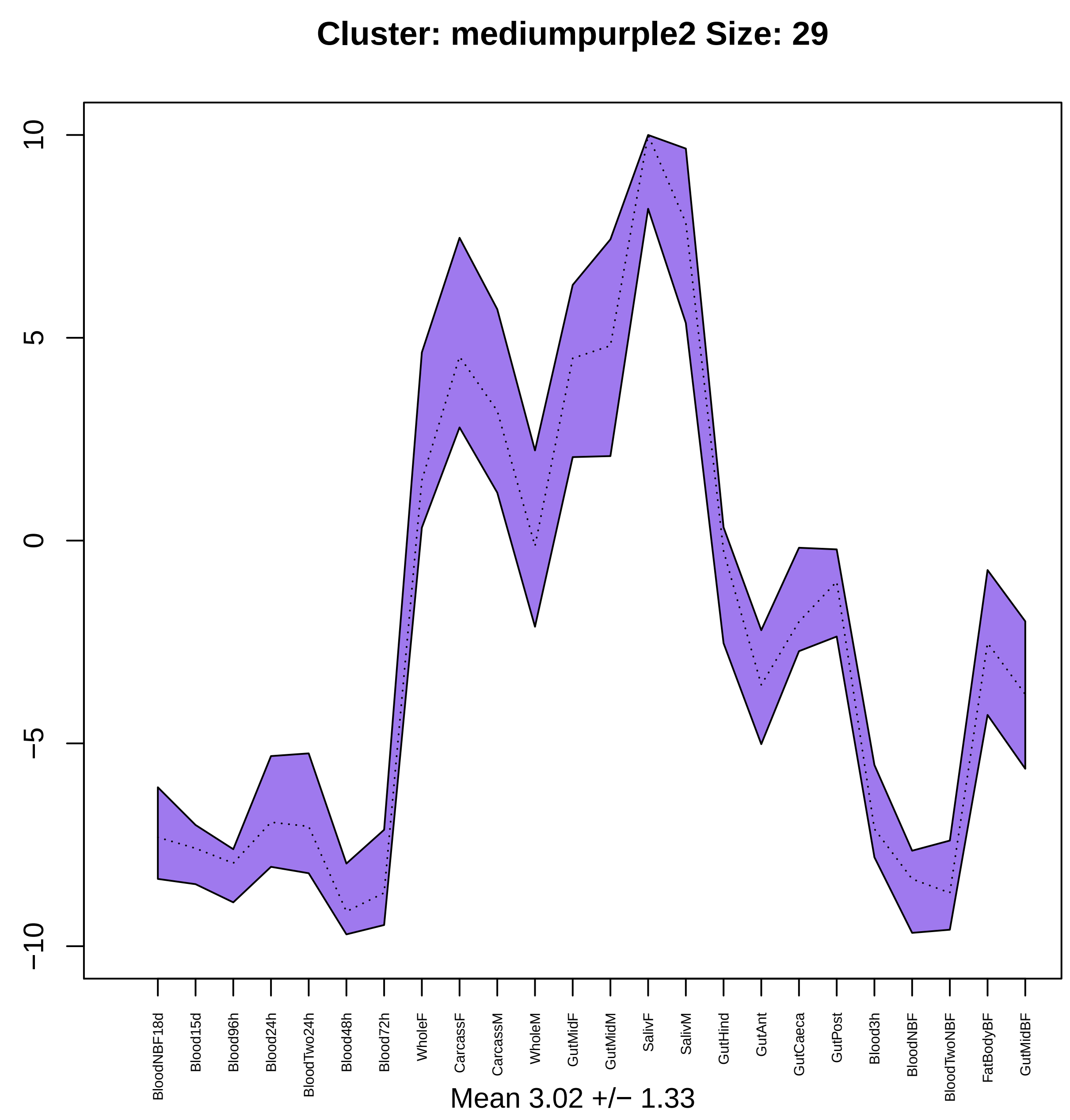

Cluster: mediumpurple2 Size: 29
10
5
0
−5
−10
BloodNBF18d
Blood15d
Blood96h
Blood24h
BloodTwo24h
Blood48h
Blood72h
WholeF
CarcassF
CarcassM
WholeM
GutMidF
GutMidM
SalivF
SalivM
GutHind
GutAnt
GutCaeca
GutPost
Blood3h
BloodNBF
BloodTwoNBF
FatBodyBF
GutMidBF
Mean 3.02 +/− 1.33

# Cluster: skyblue3 Size: 57

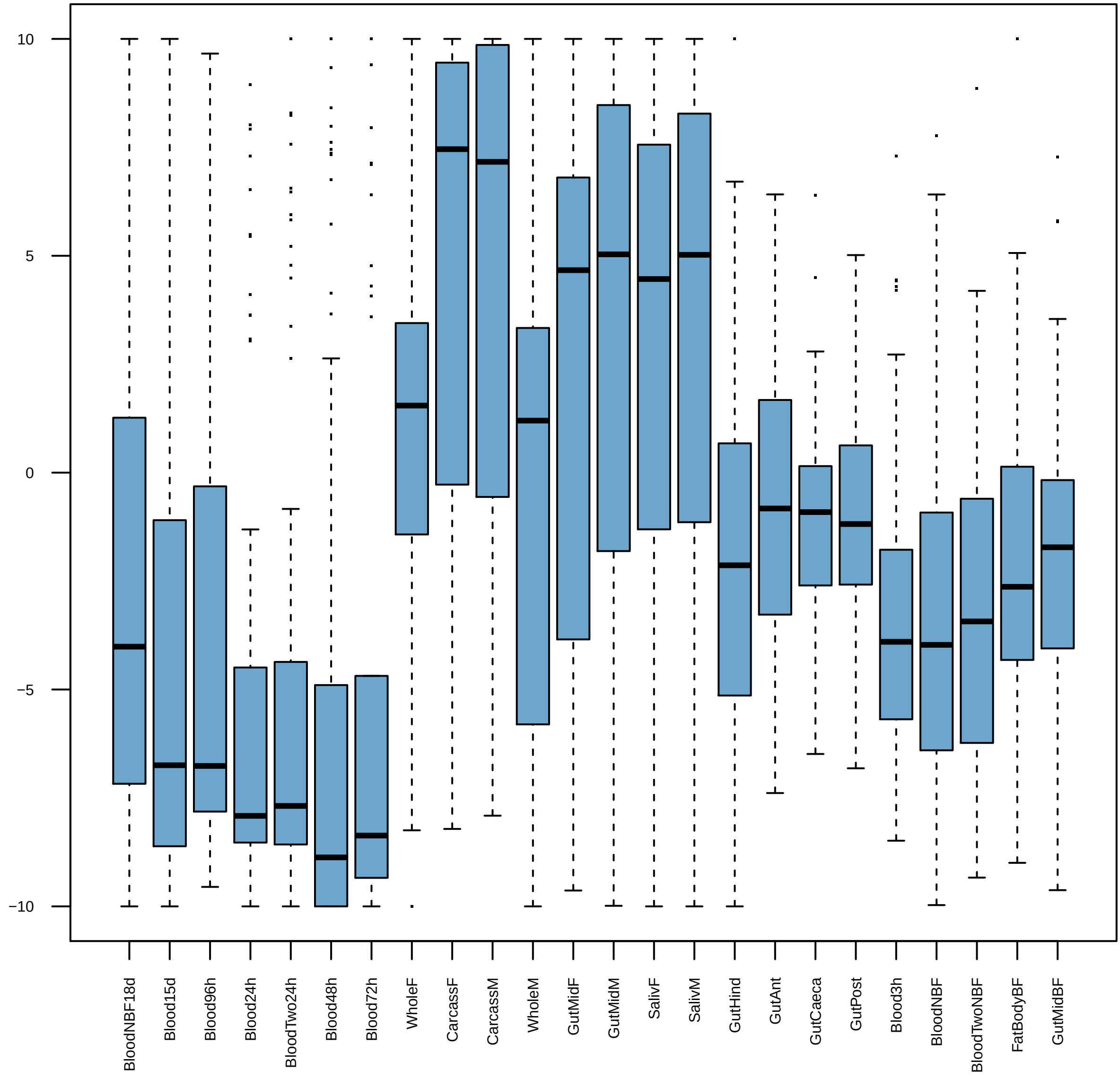

| | GO.ID | BPCluster: skyblue3 Size: 57 | Annotated | Significant | Expected | Rank in ClassicF | Weight01F | ClassicF |
|---|---|---|---|---|---|---|---|---|
| *1* | GO:0055088 | lipid homeostasis | 12 | 2 | 0.08 | 2 | 0.0024 | 0.0024 |

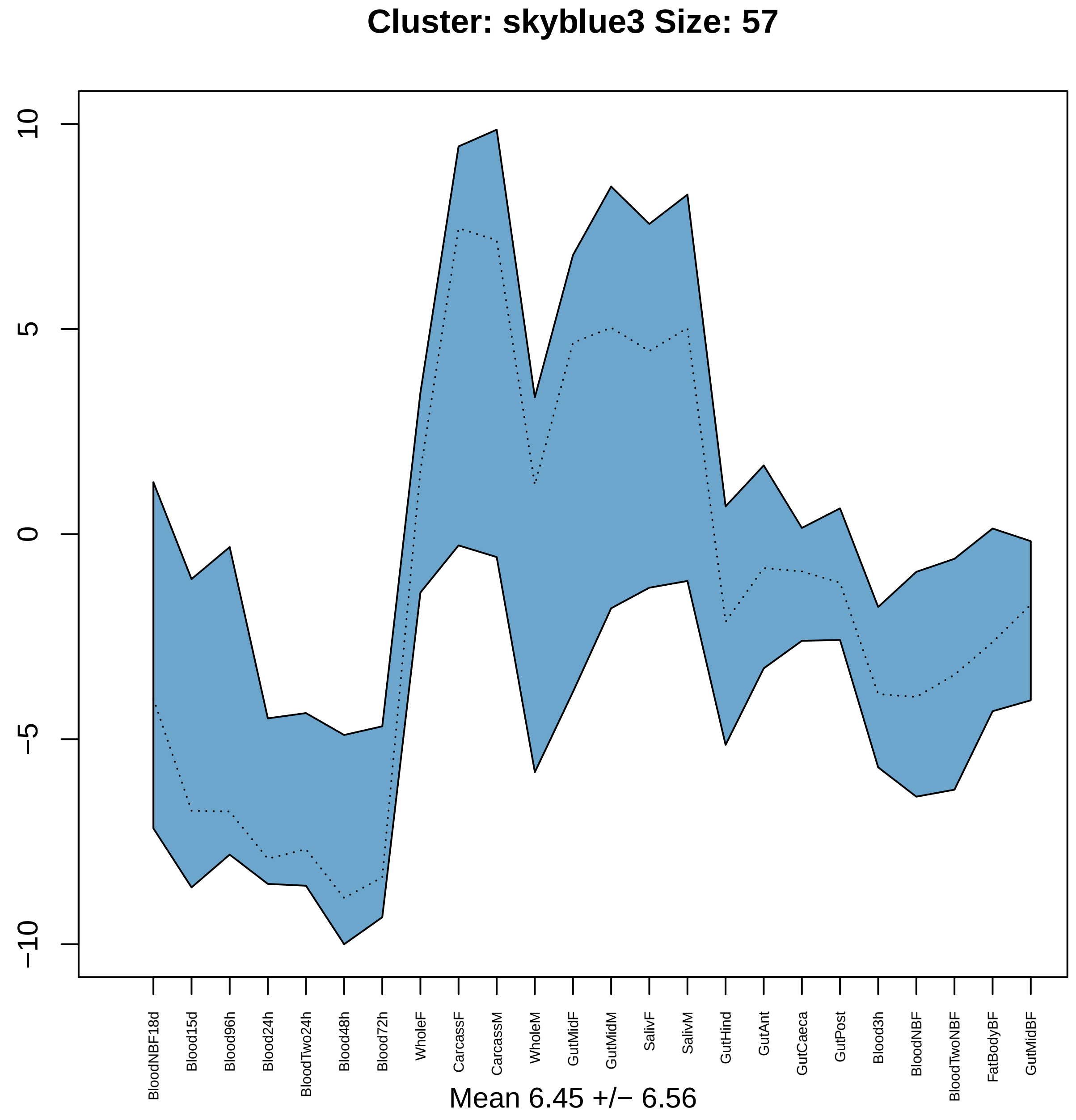
Cluster: skyblue3 Size: 57
10
5
0
−5
−10
BloodNBF18d
Blood15d
Blood96h
Blood24h
BloodTwo24h
Blood48h
Blood72h
WholeF
CarcassF
CarcassM
WholeM
GutMidF
GutMidM
SalivF
SalivM
GutHind
GutAnt
GutCaeca
GutPost
Blood3h
BloodNBF
BloodTwoNBF
FatBodyBF
GutMidBF
Mean 6.45 +/− 6.56

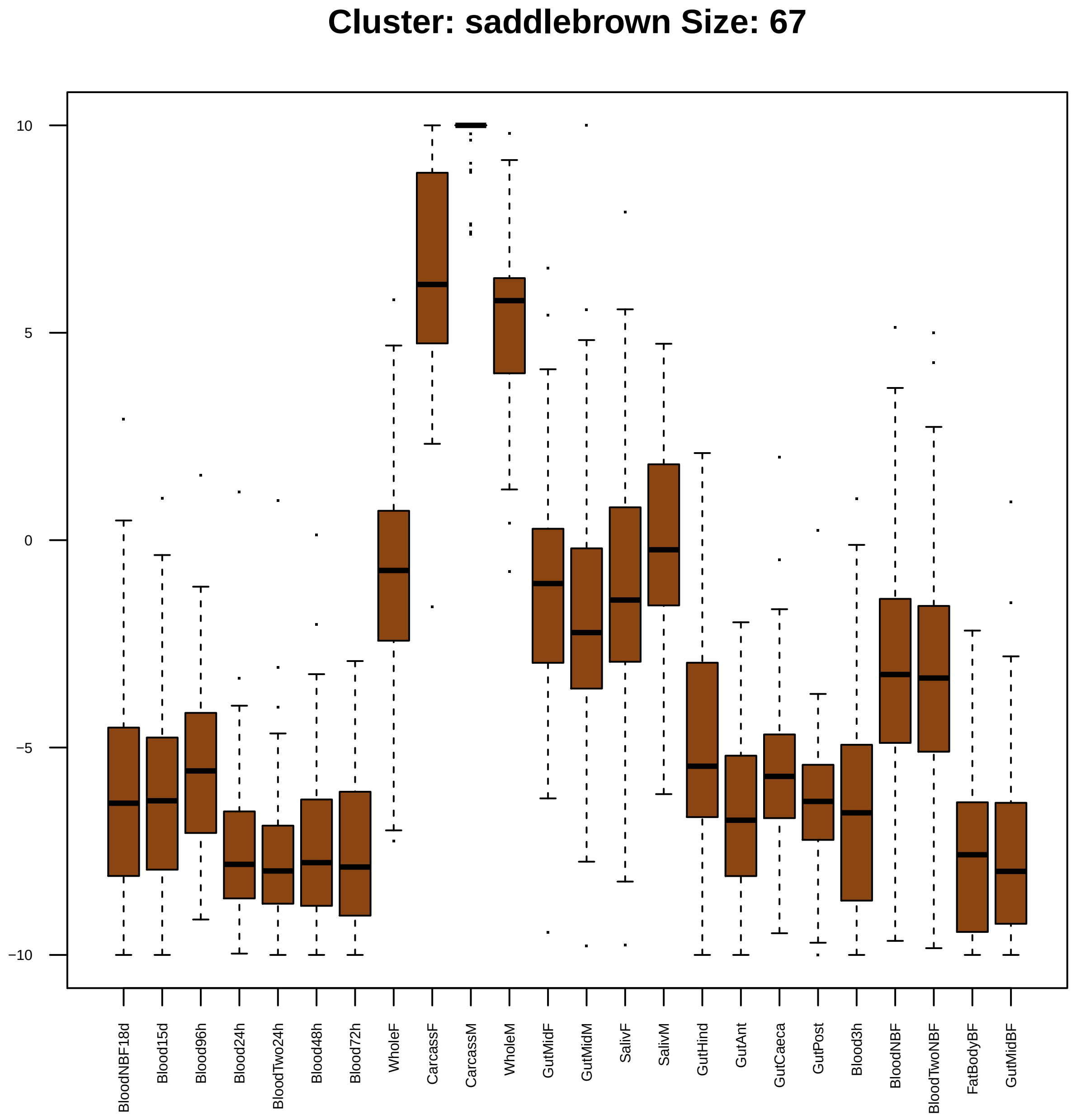
Cluster: saddlebrown Size: 67
10
5
0
−5
−10
BloodNBF18d
Blood15d
Blood96h
Blood24h
BloodTwo24h
Blood48h
Blood72h
WholeF
CarcassF
CarcassM
WholeM
GutMidF
GutMidM
SalivF
SalivM
GutHind
GutAnt
GutCaeca
GutPost
Blood3h
BloodNBF
BloodTwoNBF
FatBodyBF
GutMidBF

| | GO.ID | BPCluster: saddlebrown Size: 67 | Annotated | Significant | Expected | Rank in ClassicF | Weight01F | ClassicF |
|---|---|---|---|---|---|---|---|---|
| 2 | GO:0055114 | oxidation−reduction process | 545 | 16 | 3.67 | 2 | 0.0010 | 2.0e−07 |
| 3 | GO:0006544 | glycine metabolic process | 10 | 2 | 0.07 | 19 | 0.0019 | 0.00193 |
| 4 | GO:0016226 | iron−sulfur cluster assembly | 11 | 2 | 0.07 | 20 | 0.0024 | 0.00235 |
| 5 | GO:0005977 | glycogen metabolic process | 11 | 2 | 0.07 | 21 | 0.0024 | 0.00235 |
| 6 | GO:0042135 | neurotransmitter catabolic process | 12 | 2 | 0.08 | 29 | 0.0028 | 0.00281 |
| 7 | GO:0006096 | glycolytic process | 12 | 2 | 0.08 | 30 | 0.0028 | 0.00281 |
| 11 | GO:0009117 | nucleotide metabolic process | 165 | 5 | 1.11 | 39 | 0.0310 | 0.00475 |
| 12 | GO:0016052 | carbohydrate catabolic process | 17 | 3 | 0.11 | 14 | 0.0318 | 0.00018 |
| 15 | GO:0006090 | pyruvate metabolic process | 20 | 3 | 0.13 | 16 | 0.0504 | 0.00030 |

| | GO.ID | MFCluster: saddlebrown Size: 67 | Annotated | Significant | Expected | Rank in ClassicF | Weight01F | ClassicF |
|---|---|---|---|---|---|---|---|---|
| 1 | GO:0016616 | oxidoreductase activity, acting on the C... | 41 | 4 | 0.28 | 2 | 0.00017 | 0.00017 |
| 2 | GO:0051287 | NAD binding | 26 | 3 | 0.18 | 4 | 0.00073 | 0.00073 |
| 3 | GO:0000287 | magnesium ion binding | 34 | 3 | 0.24 | 7 | 0.00161 | 0.00161 |
| 5 | GO:0016903 | oxidoreductase activity, acting on the a... | 33 | 3 | 0.23 | 5 | 0.03945 | 0.00148 |
| 17 | GO:0043169 | cation binding | 1275 | 17 | 8.84 | 9 | 0.10838 | 0.00403 |

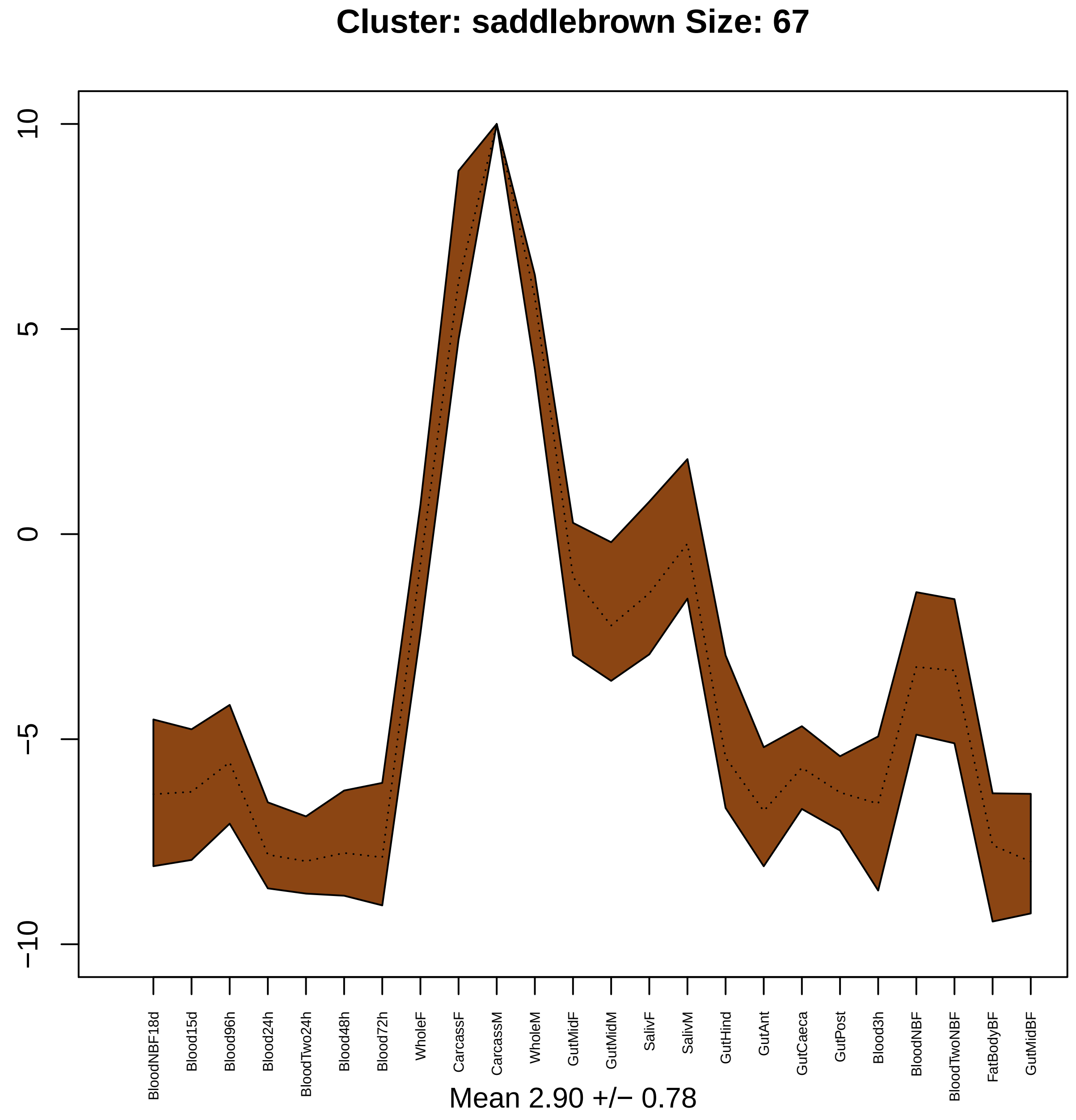
Cluster: saddlebrown Size: 67
10
5
0
−5
−10
BloodNBF18d
Blood15d
Blood96h
Blood24h
BloodTwo24h
Blood48h
Blood72h
WholeF
CarcassF
CarcassM
WholeM
GutMidF
GutMidM
SalivF
SalivM
GutHind
GutAnt
GutCaeca
GutPost
Blood3h
BloodNBF
BloodTwoNBF
FatBodyBF
GutMidBF
Mean 2.90 +/− 0.78

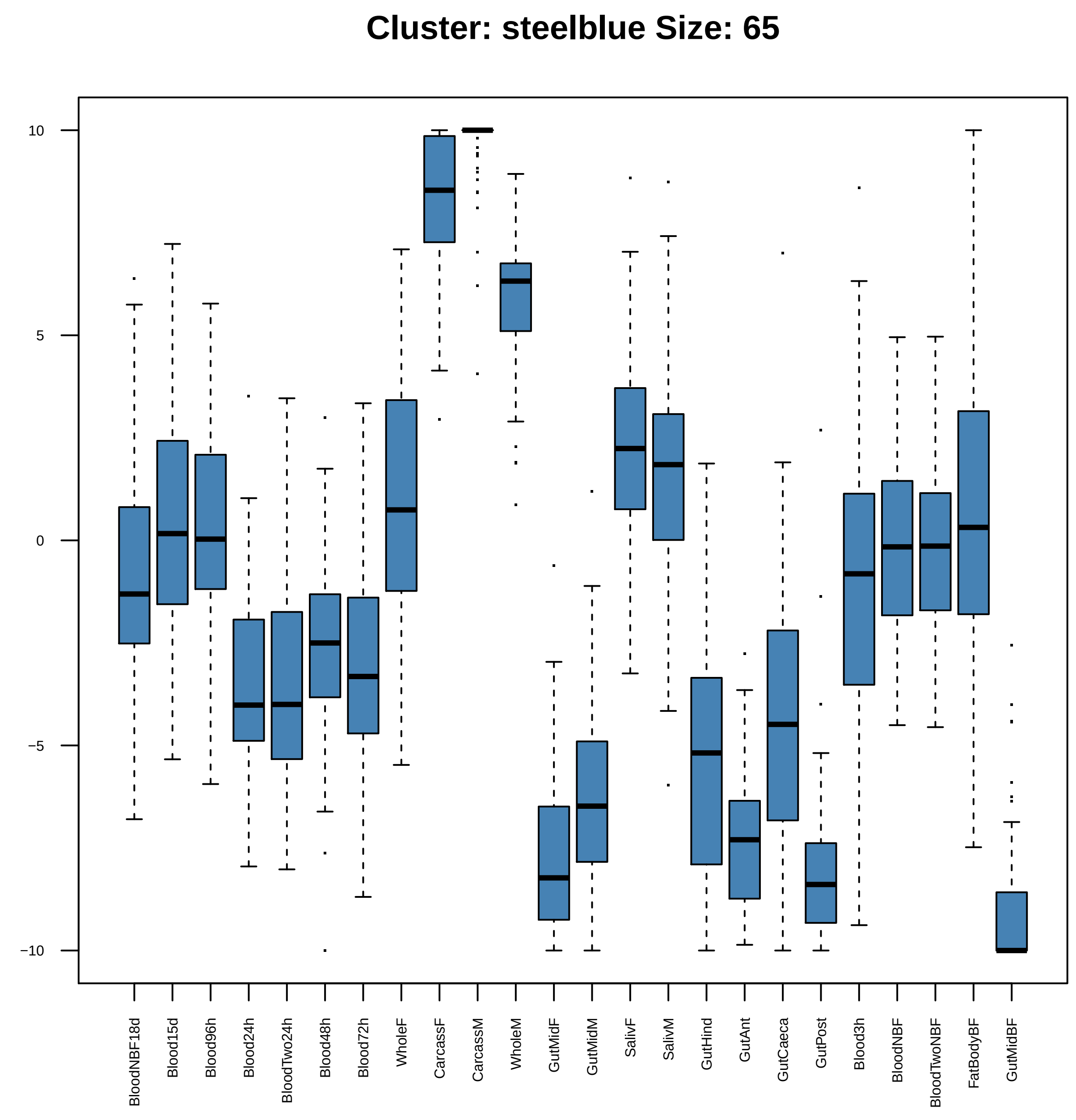

Cluster: steelblue Size: 65
10
5
0
−5
−10
BloodNBF18d
Blood15d
Blood96h
Blood24h
BloodTwo24h
Blood48h
Blood72h
WholeF
CarcassF
CarcassM
WholeM
GutMidF
GutMidM
SalivF
SalivM
GutHind
GutAnt
GutCaeca
GutPost
Blood3h
BloodNBF
BloodTwoNBF
FatBodyBF
GutMidBF

| | GO.ID | BPCluster: steelblue Size: 65 | Annotated | Significant | Expected | Rank in ClassicF | Weight01F | ClassicF |
|---|---|---|---|---|---|---|---|---|
| *3* | GO:0009084 | glutamine family amino acid biosynthetic... | 12 | 2 | 0.07 | 18 | 0.0021 | 0.00208 |
| *10* | GO:0008152 | metabolic process | 4285 | 34 | 24.86 | 11 | 0.0858 | 0.00043 |

| | GO.ID | MFCluster: steelblue Size: 65 | Annotated | Significant | Expected | Rank in ClassicF | Weight01F | ClassicF |
|---|---|---|---|---|---|---|---|---|
| 2 | GO:0004866 | endopeptidase inhibitor activity | 40 | 4 | 0.23 | 9 | 0.0039 | 7.8e−05 |
| 3 | GO:0008233 | peptidase activity | 500 | 22 | 2.91 | 4 | 0.0042 | 2.8e−15 |
| 4 | GO:0004867 | serine−type endopeptidase inhibitor acti... | 23 | 2 | 0.13 | 15 | 0.0078 | 0.0078 |

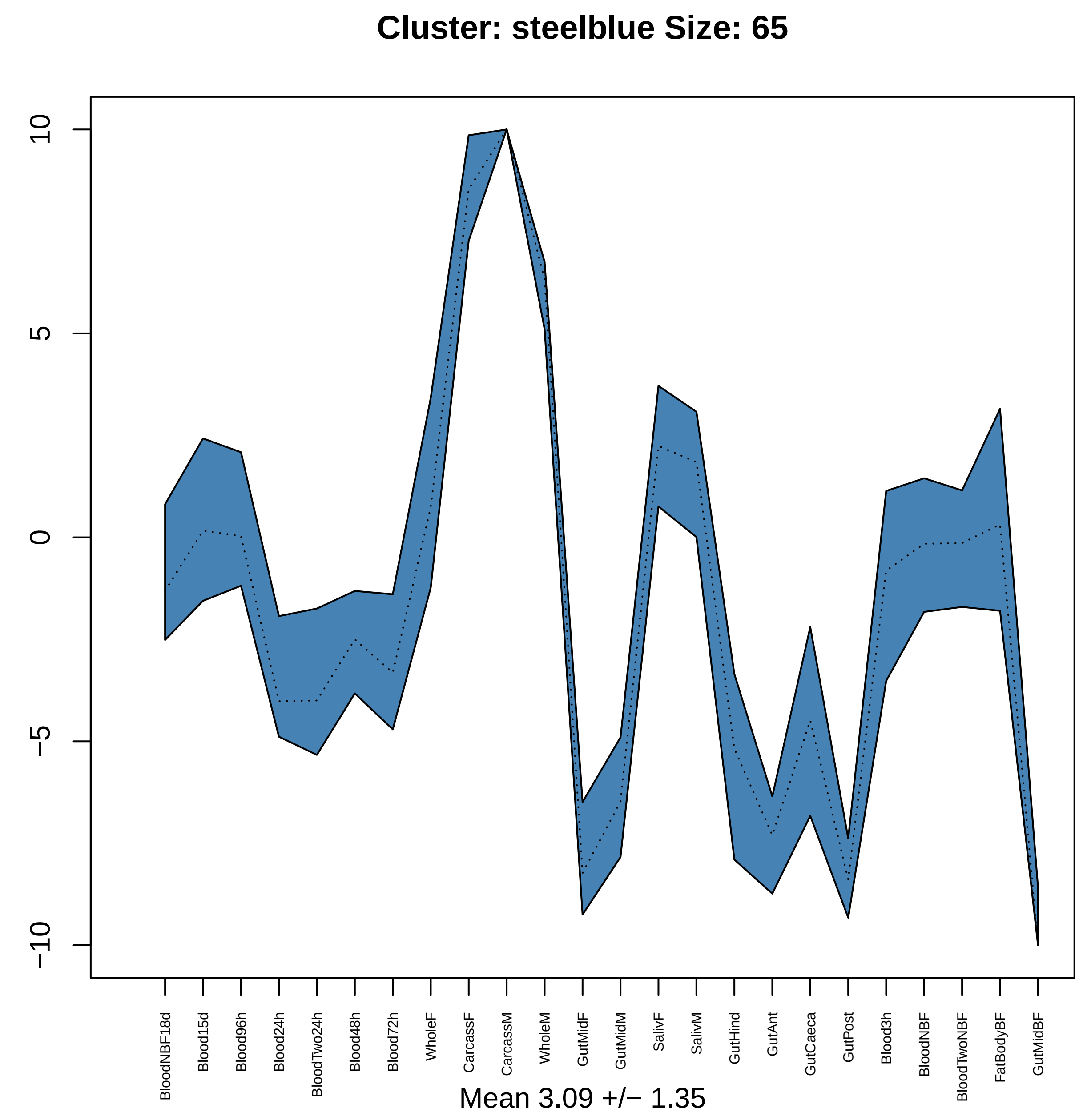

Cluster: steelblue Size: 65
10
5
0
−5
−10
BloodNBF18d
Blood15d
Blood96h
Blood24h
BloodTwo24h
Blood48h
Blood72h
WholeF
CarcassF
CarcassM
WholeM
GutMidF
GutMidM
SalivF
SalivM
GutHind
GutAnt
GutCaeca
GutPost
Blood3h
BloodNBF
BloodTwoNBF
FatBodyBF
GutMidBF
Mean 3.09 +/− 1.35

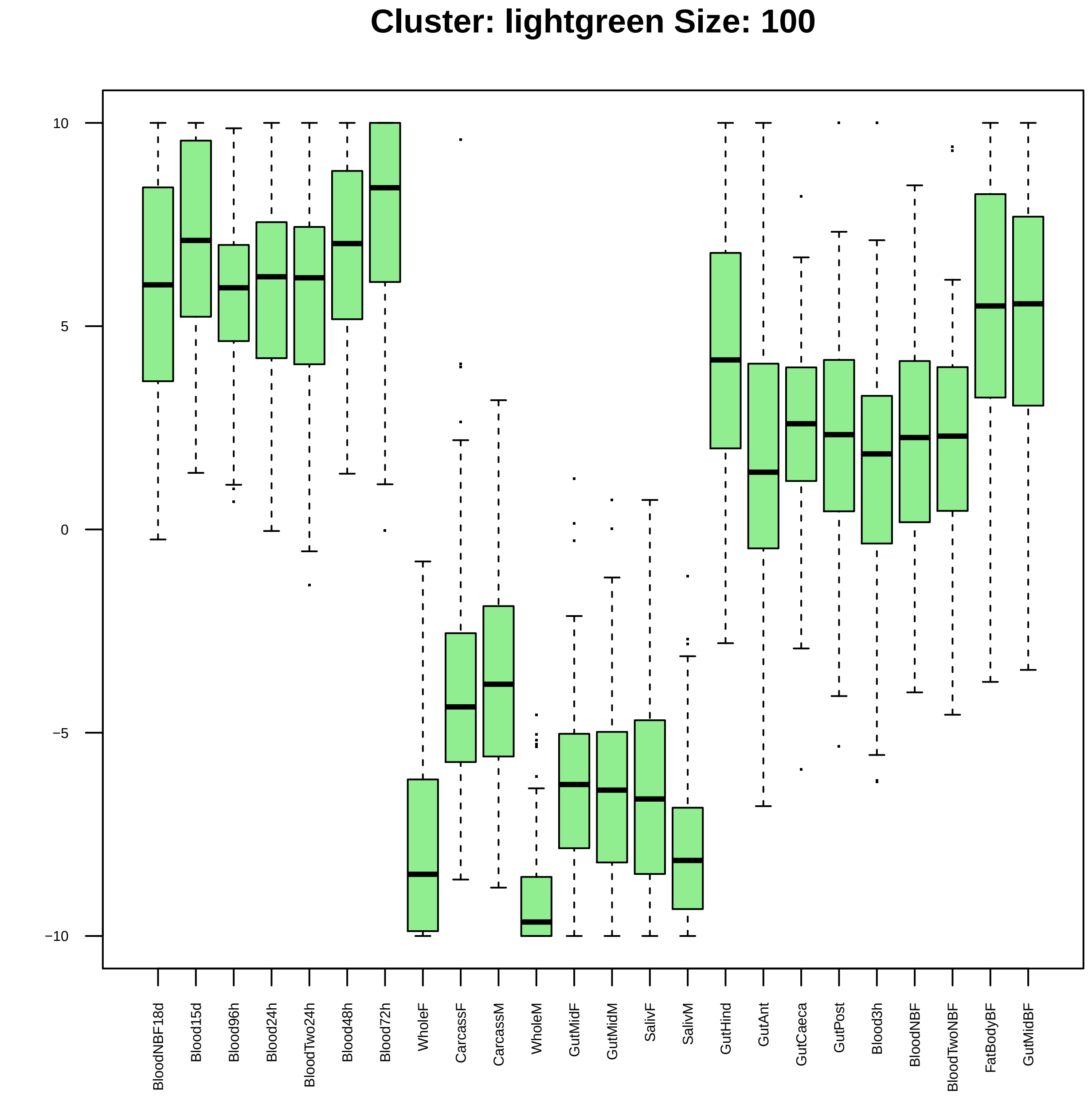

Cluster: lightgreen Size: 100
10
5
0
−5
−10
BloodNBF18d
Blood15d
Blood96h
Blood24h
BloodTwo24h
Blood48h
Blood72h
WholeF
CarcassF
CarcassM
WholeM
GutMidF
GutMidM
SalivF
SalivM
GutHind
GutAnt
GutCaeca
GutPost
Blood3h
BloodNBF
BloodTwoNBF
FatBodyBF
GutMidBF

| | GO.ID | BPCluster: lightgreen Size: 100 | Annotated | Significant | Expected | Rank in ClassicF | Weight01F | ClassicF |
|---|---|---|---|---|---|---|---|---|
| 1 | GO:0016482 | cytosolic transport | 15 | 3 | 0.16 | 30 | 0.00052 | 0.00052 |
| 2 | GO:0042078 | germ−line stem cell division | 18 | 3 | 0.20 | 37 | 0.00092 | 0.00092 |
| 3 | GO:0050829 | defense response to Gram−negative bacter... | 43 | 4 | 0.47 | 43 | 0.00119 | 0.00119 |
| 4 | GO:0022416 | chaeta development | 44 | 4 | 0.48 | 46 | 0.00130 | 0.00130 |
| 5 | GO:0030718 | germ−line stem cell population maintenan... | 26 | 3 | 0.29 | 62 | 0.00275 | 0.00275 |
| 6 | GO:0007391 | dorsal closure | 58 | 4 | 0.64 | 75 | 0.00362 | 0.00362 |
| 7 | GO:0022411 | cellular component disassembly | 36 | 3 | 0.40 | 113 | 0.00399 | 0.00698 |
| 8 | GO:0042044 | fluid transport | 10 | 2 | 0.11 | 89 | 0.00505 | 0.00505 |
| 9 | GO:0045887 | positive regulation of synaptic growth a... | 10 | 2 | 0.11 | 90 | 0.00505 | 0.00505 |
| 10 | GO:0098930 | axonal transport | 10 | 2 | 0.11 | 91 | 0.00505 | 0.00505 |
| 11 | GO:0046330 | positive regulation of JNK cascade | 11 | 2 | 0.12 | 102 | 0.00613 | 0.00613 |
| 12 | GO:0046529 | imaginal disc fusion, thorax closure | 12 | 2 | 0.13 | 117 | 0.00730 | 0.00730 |
| 13 | GO:0007432 | salivary gland boundary specification | 12 | 2 | 0.13 | 118 | 0.00730 | 0.00730 |
| 14 | GO:0007517 | muscle organ development | 66 | 4 | 0.72 | 99 | 0.00790 | 0.00575 |
| 15 | GO:0110111 | negative regulation of animal organ morp... | 14 | 2 | 0.15 | 142 | 0.00993 | 0.00993 |
| 16 | GO:0048542 | lymph gland development | 14 | 2 | 0.15 | 143 | 0.00993 | 0.00993 |
| 17 | GO:0007400 | neuroblast fate determination | 14 | 2 | 0.15 | 144 | 0.00993 | 0.00993 |
| 26 | GO:0001763 | morphogenesis of a branching structure | 38 | 3 | 0.42 | 126 | 0.02133 | 0.00812 |
| 27 | GO:0072001 | renal system development | 40 | 3 | 0.44 | 138 | 0.02134 | 0.00936 |
| 28 | GO:0035051 | cardiocyte differentiation | 14 | 2 | 0.15 | 145 | 0.02157 | 0.00993 |
| 30 | GO:0006468 | protein phosphorylation | 271 | 9 | 2.97 | 61 | 0.02210 | 0.00264 |

| | GO.ID | MFCluster: lightgreen Size: 100 | Annotated | Significant | Expected | Rank in ClassicF | Weight01F | ClassicF |
|---|---|---|---|---|---|---|---|---|
| 1 | GO:0004674 | protein serine/threonine kinase activity | 150 | 6 | 1.56 | 4 | 0.0045 | 0.00454 |
| 2 | GO:0004879 | nuclear receptor activity | 10 | 2 | 0.10 | 5 | 0.0046 | 0.00455 |
| 3 | GO:0008092 | cytoskeletal protein binding | 170 | 8 | 1.77 | 1 | 0.0057 | 0.00036 |
| 7 | GO:0005515 | protein binding | 2143 | 36 | 22.30 | 2 | 0.0341 | 0.00062 |

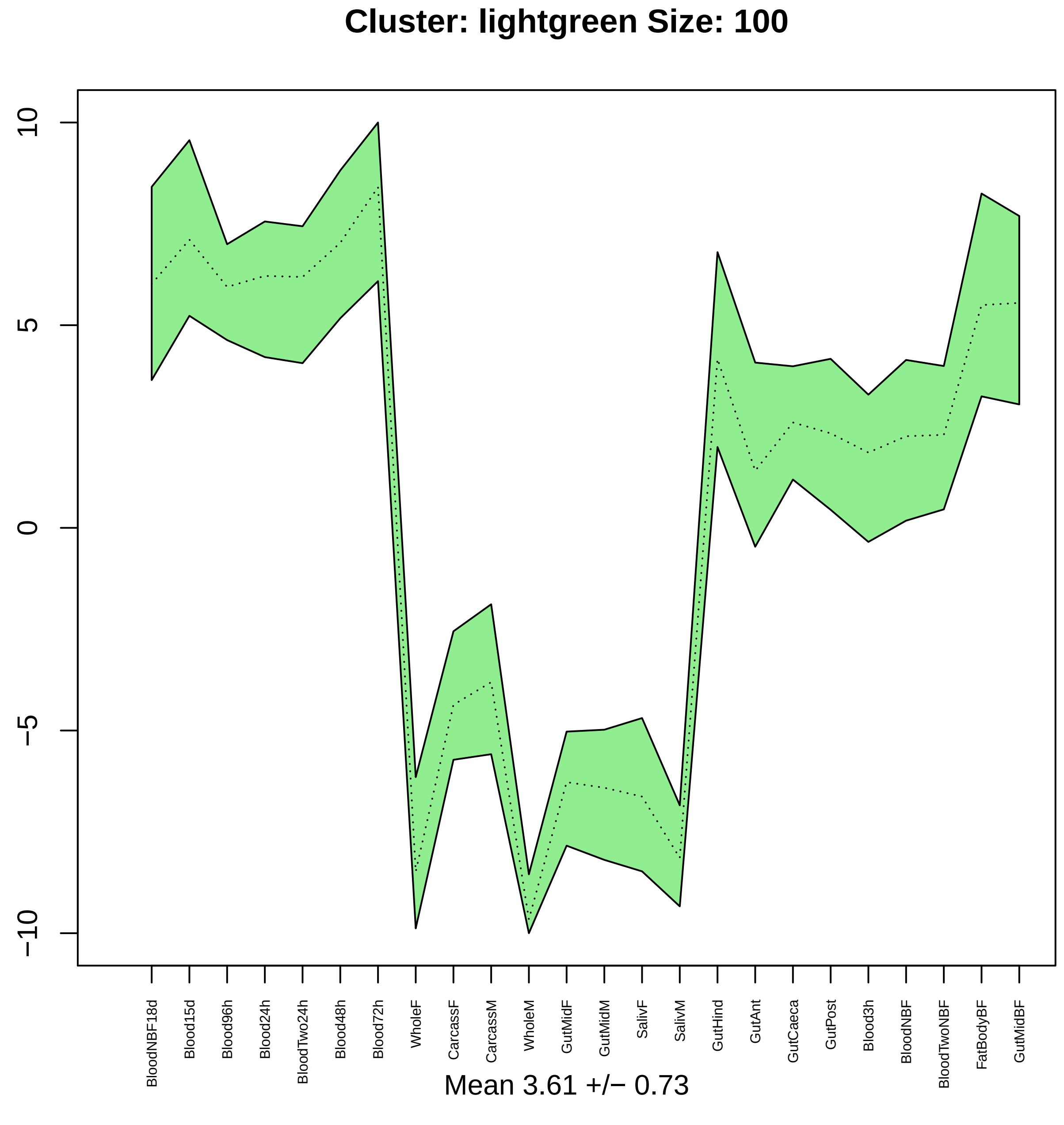

Cluster: lightgreen Size: 100
10
5
0
−5
−10
BloodNBF18d
Blood15d
Blood96h
Blood24h
BloodTwo24h
Blood48h
Blood72h
WholeF
CarcassF
CarcassM
WholeM
GutMidF
GutMidM
SalivF
SalivM
GutHind
GutAnt
GutCaeca
GutPost
Blood3h
BloodNBF
BloodTwoNBF
FatBodyBF
GutMidBF
Mean 3.61 +/− 0.73

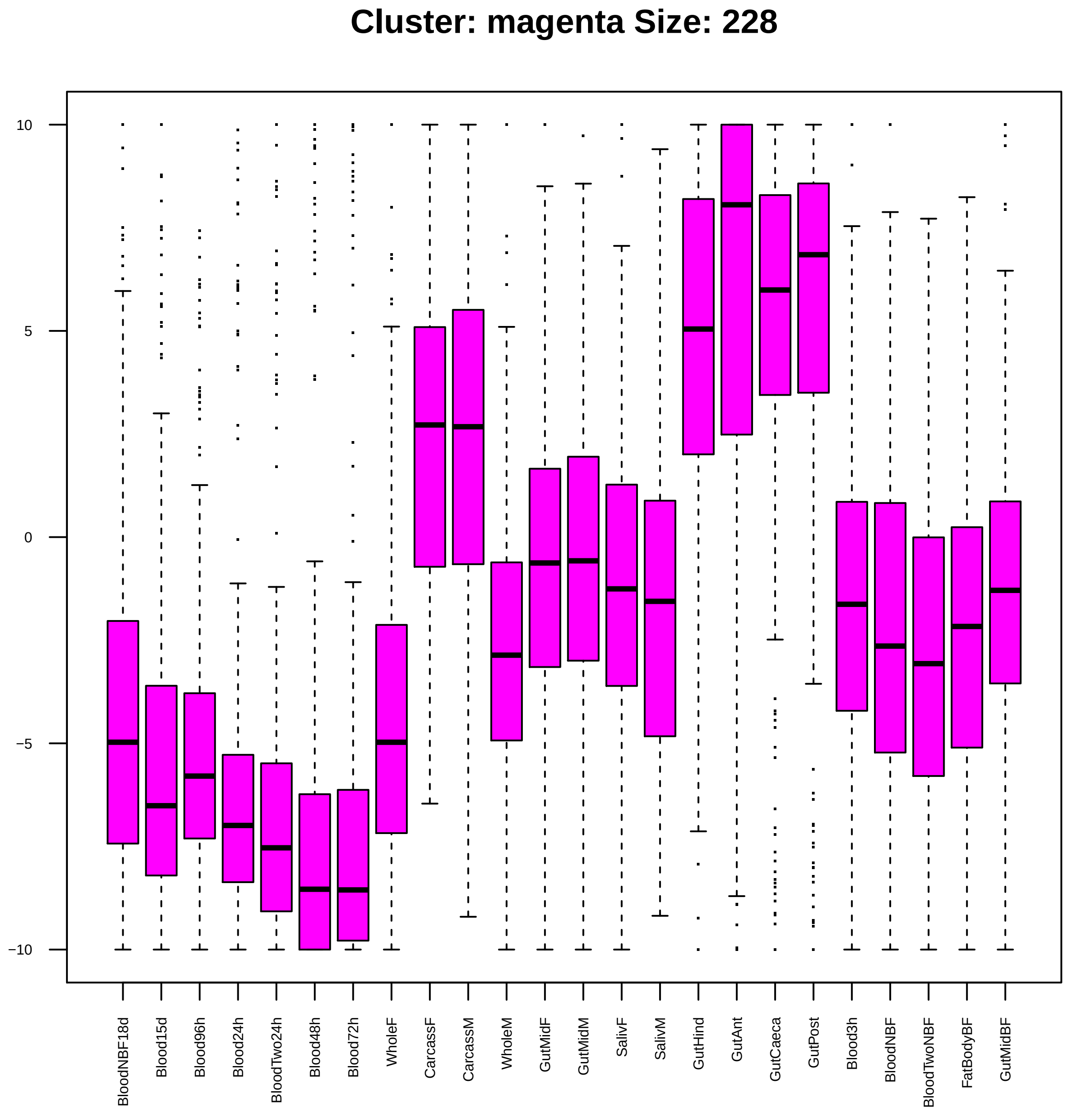
Cluster: magenta Size: 228
10
5
0
−5
−10
BloodNBF18d
Blood15d
Blood96h
Blood24h
BloodTwo24h
Blood48h
Blood72h
WholeF
CarcassF
CarcassM
WholeM
GutMidF
GutMidM
SalivF
SalivM
GutHind
GutAnt
GutCaeca
GutPost
Blood3h
BloodNBF
BloodTwoNBF
FatBodyBF
GutMidBF

| | GO.ID | BPCluster: magenta Size: 228 | Annotated | Significant | Expected | Rank in ClassicF | Weight01F | ClassicF |
|---|---|---|---|---|---|---|---|---|
| *1* | GO:0050907 | detection of chemical stimulus involved ... | 83 | 7 | 1.65 | 3 | 0.0011 | 0.0012 |
| *2* | GO:0008152 | metabolic process | 4285 | 87 | 85.34 | 403 | 0.0031 | 0.4161 |
| *3* | GO:0006836 | neurotransmitter transport | 81 | 4 | 1.61 | 61 | 0.0069 | 0.0774 |

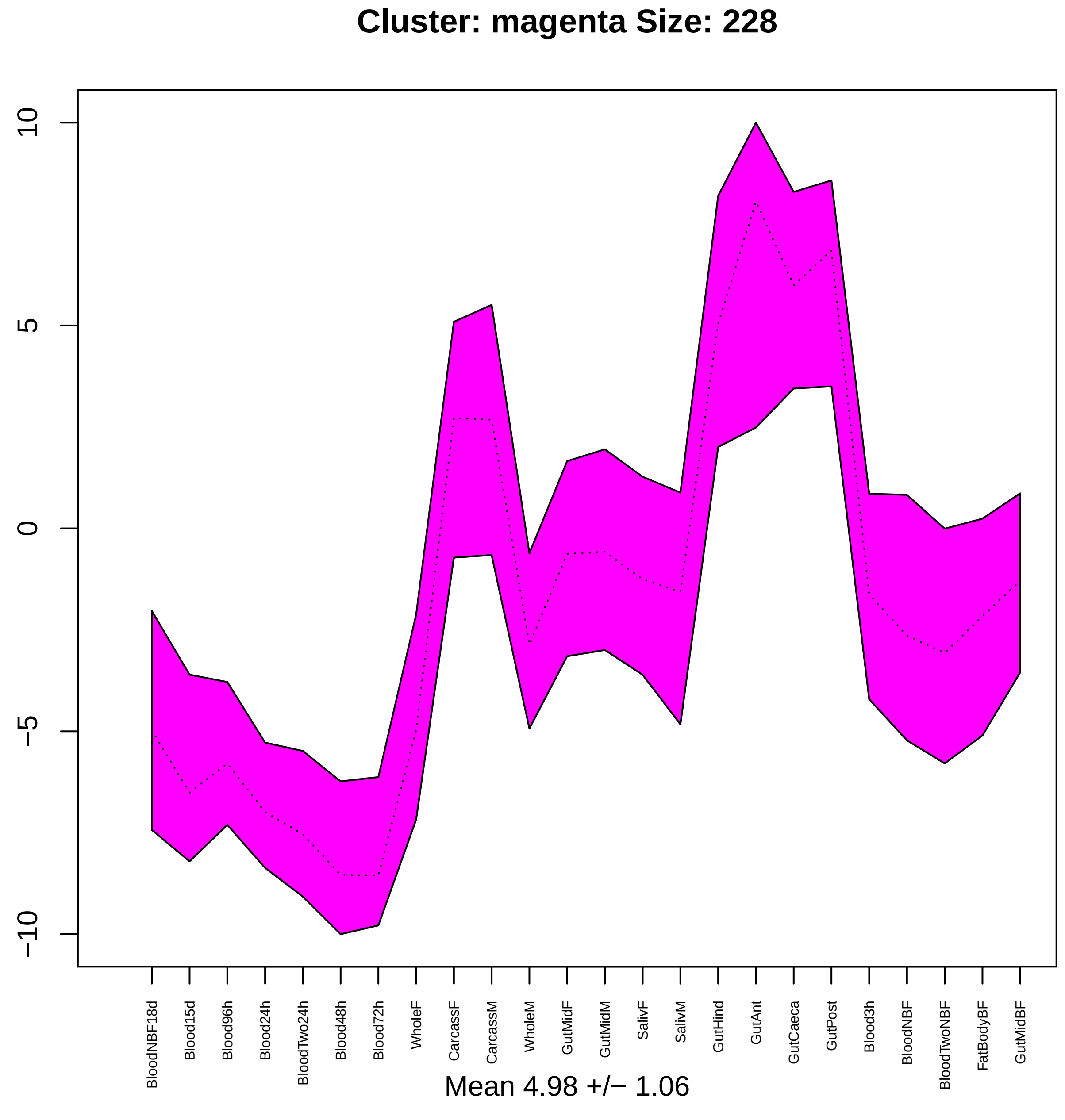

Cluster: magenta Size: 228
10
5
0
−5
−10
BloodNBF18d
Blood15d
Blood96h
Blood24h
BloodTwo24h
Blood48h
Blood72h
WholeF
CarcassF
CarcassM
WholeM
GutMidF
GutMidM
SalivF
SalivM
GutHind
GutAnt
GutCaeca
GutPost
Blood3h
BloodNBF
BloodTwoNBF
FatBodyBF
GutMidBF
Mean 4.98 +/− 1.06

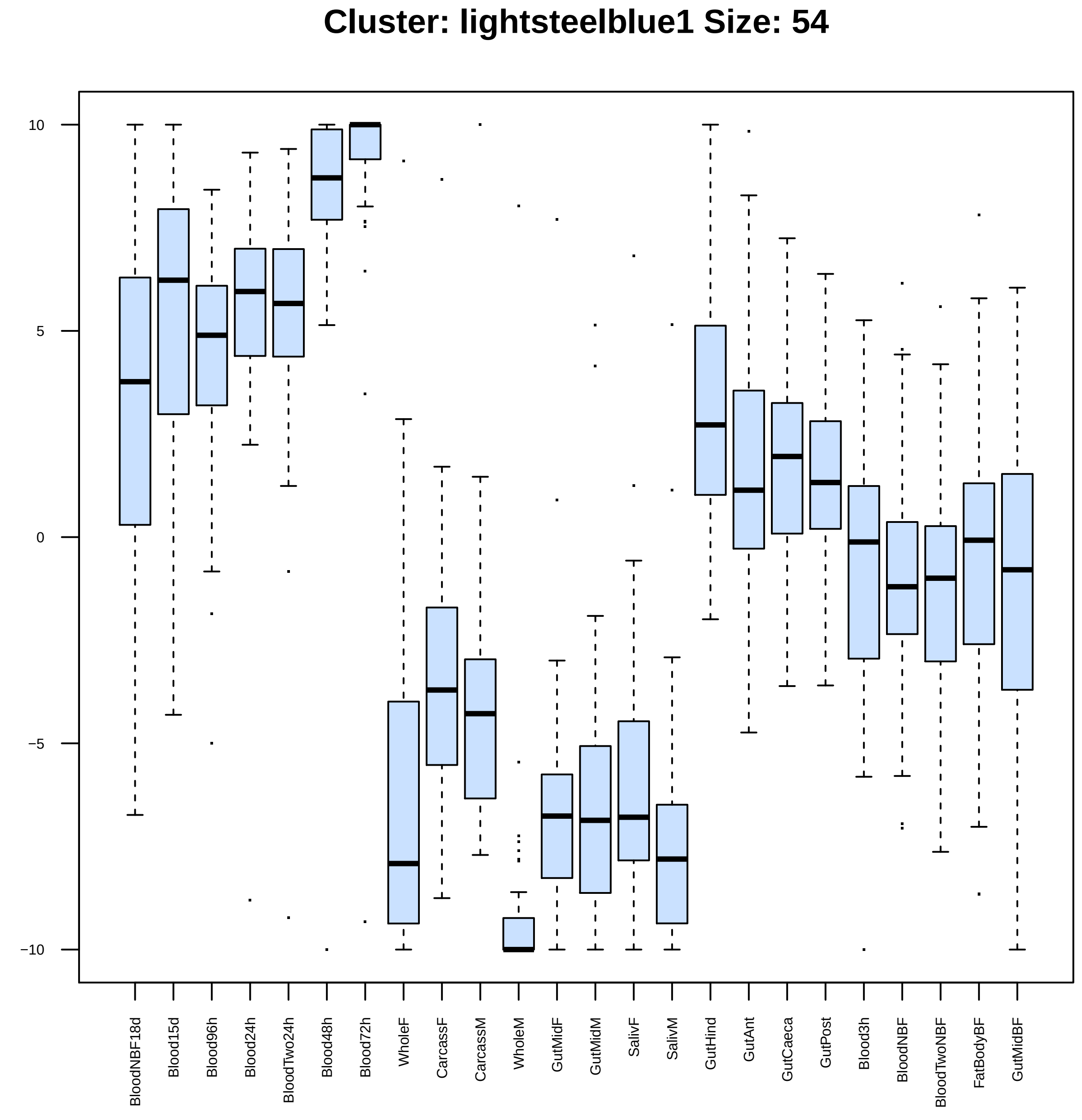

Cluster: lightsteelblue1 Size: 54
10
5
0
−5
−10
BloodNBF18d
Blood15d
Blood96h
Blood24h
BloodTwo24h
Blood48h
Blood72h
WholeF
CarcassF
CarcassM
WholeM
GutMidF
GutMidM
SalivF
SalivM
GutHind
GutAnt
GutCaeca
GutPost
Blood3h
BloodNBF
BloodTwoNBF
FatBodyBF
GutMidBF

| | GO.ID | BPCluster: lightsteelblue1 Size: 54 | Annotated | Significant | Expected | Rank in ClassicF | Weight01F | ClassicF |
|---|---|---|---|---|---|---|---|---|
| 1 | GO:2000045 | regulation of G1/S transition of mitotic... | 16 | 2 | 0.09 | 20 | 0.0033 | 0.0033 |
| 2 | GO:0120032 | regulation of plasma membrane bounded ce... | 13 | 2 | 0.07 | 16 | 0.0053 | 0.0022 |

|  | GO.ID | MFCluster: lightsteelblue1 Size: 54 | Annotated | Significant | Expected | Rank in ClassicF | Weight01F | ClassicF |
|---|---|---|---|---|---|---|---|---|
| 1 | GO:0003729 | mRNA binding | 99 | 4 | 0.56 | 6 | 0.0023 | 0.00230 |
| 2 | GO:0000166 | nucleotide binding | 998 | 10 | 5.68 | 16 | 0.0025 | 0.04892 |
| 3 | GO:0019901 | protein kinase binding | 24 | 2 | 0.14 | 7 | 0.0080 | 0.00805 |
| 5 | GO:0003723 | RNA binding | 307 | 8 | 1.75 | 3 | 0.0198 | 0.00028 |

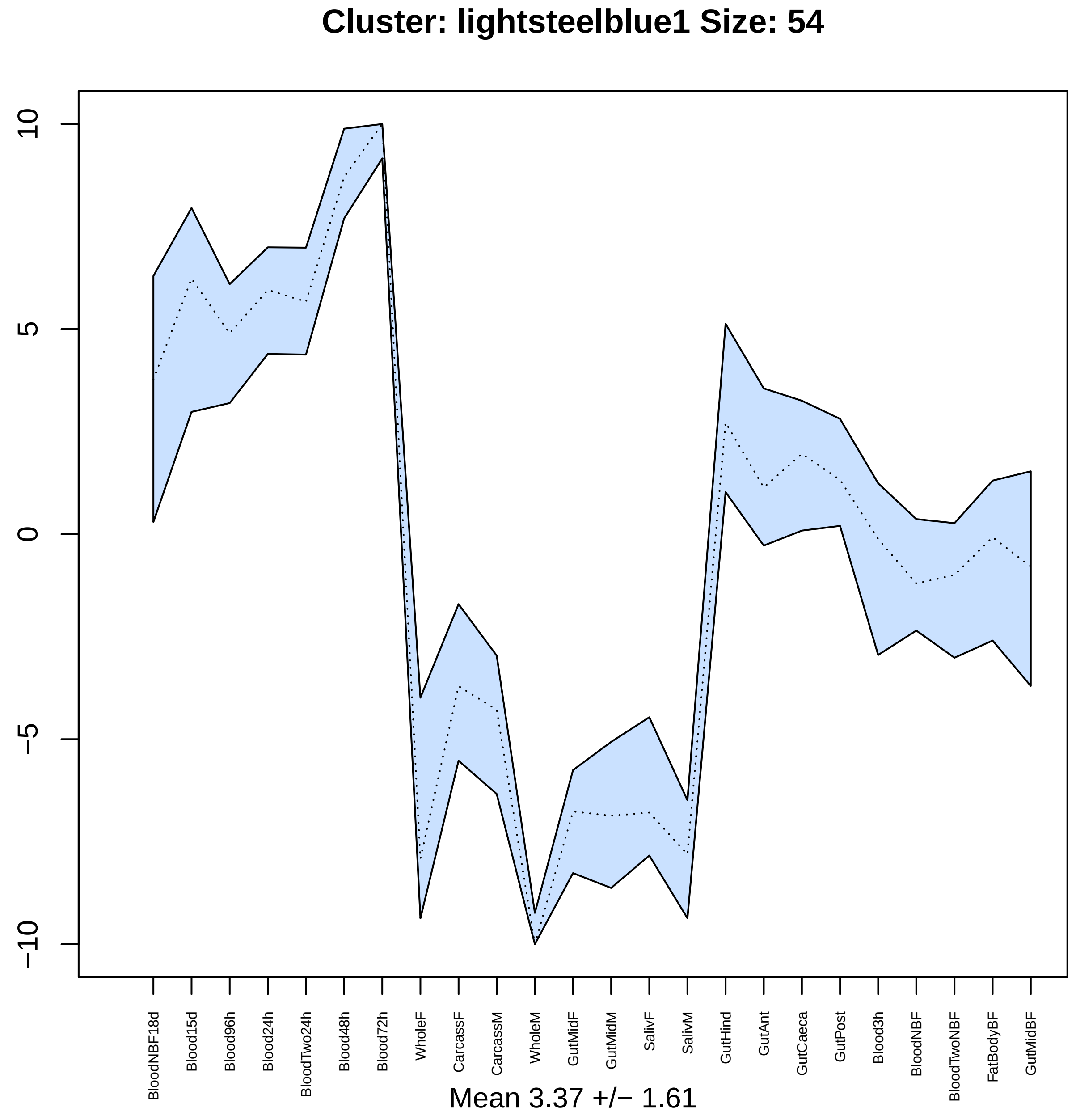

Cluster: lightsteelblue1 Size: 54
10
5
0
−5
−10
BloodNBF18d
Blood15d
Blood96h
Blood24h
BloodTwo24h
Blood48h
Blood72h
WholeF
CarcassF
CarcassM
WholeM
GutMidF
GutMidM
SalivF
SalivM
GutHind
GutAnt
GutCaeca
GutPost
Blood3h
BloodNBF
BloodTwoNBF
FatBodyBF
GutMidBF
Mean 3.37 +/− 1.61

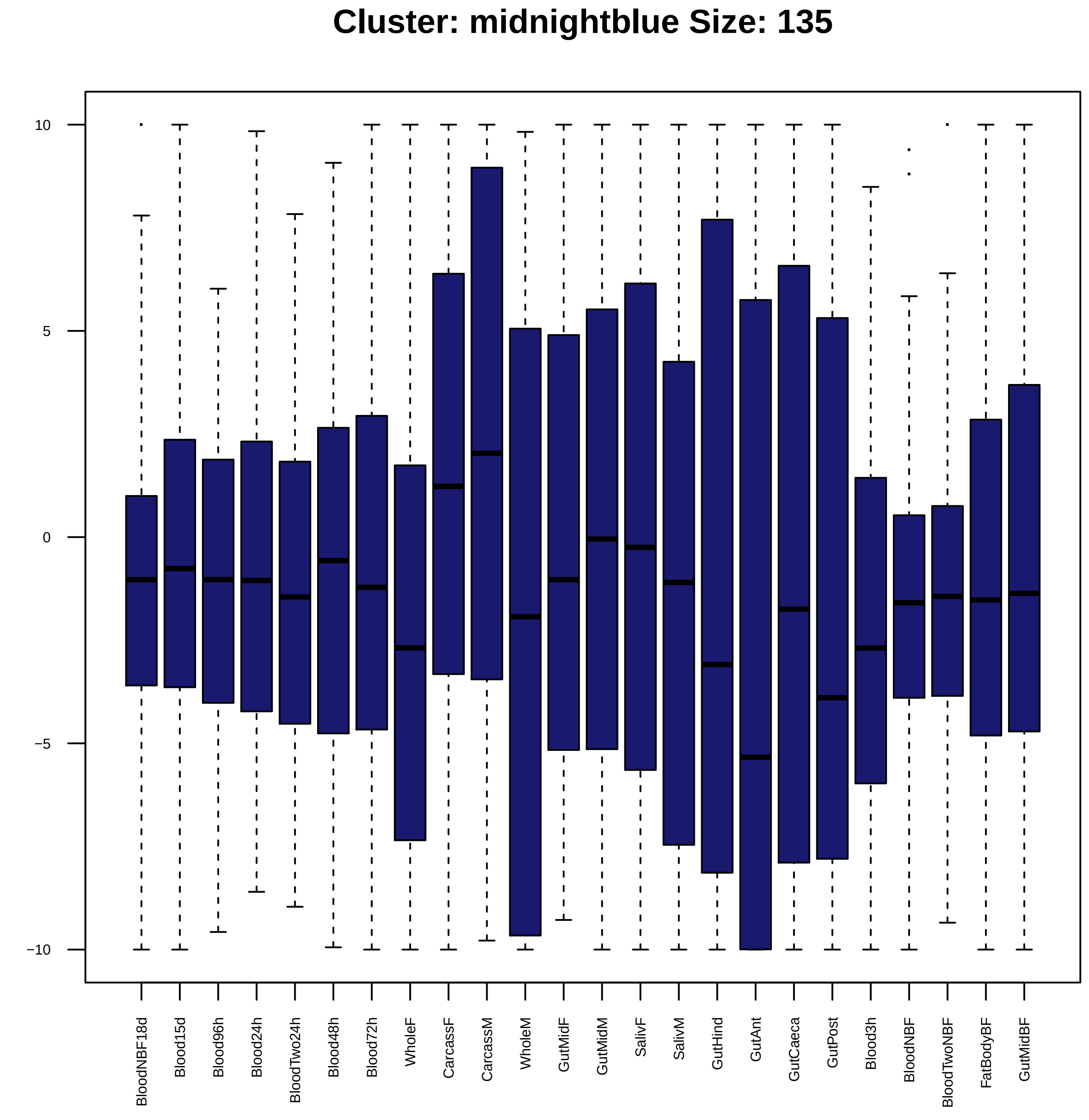

Cluster: midnightblue Size: 135
10
5
0
−5
−10
BloodNBF18d
Blood15d
Blood96h
Blood24h
BloodTwo24h
Blood48h
Blood72h
WholeF
CarcassF
CarcassM
WholeM
GutMidF
GutMidM
SalivF
SalivM
GutHind
GutAnt
GutCaeca
GutPost
Blood3h
BloodNBF
BloodTwoNBF
FatBodyBF
GutMidBF

| | GO.ID | BPCluster: midnightblue Size: 135 | Annotated | Significant | Expected | Rank in ClassicF | Weight01F | ClassicF |
|---|---|---|---|---|---|---|---|---|
| *1* | GO:0009100 | glycoprotein metabolic process | 60 | 5 | 0.88 | 9 | 0.0012 | 0.0018 |
| *2* | GO:0000281 | mitotic cytokinesis | 26 | 3 | 0.38 | 18 | 0.0063 | 0.0063 |
| *3* | GO:0007469 | antennal development | 10 | 2 | 0.15 | 21 | 0.0090 | 0.0090 |
| *4* | GO:0007314 | oocyte anterior/posterior axis specifica... | 44 | 4 | 0.65 | 13 | 0.0146 | 0.0038 |
| *12* | GO:0030707 | ovarian follicle cell development | 121 | 6 | 1.78 | 20 | 0.0268 | 0.0086 |
| *20* | GO:0009798 | axis specification | 108 | 7 | 1.59 | 5 | 0.0415 | 0.0010 |

| | GO.ID | MFCluster: midnightblue Size: 135 | Annotated | Significant | Expected | Rank in ClassicF | Weight01F | ClassicF |
|---|---|---|---|---|---|---|---|---|
| *1* | GO:0008324 | cation transmembrane transporter activit... | 210 | 4 | 2.80 | 61 | 0.0014 | 0.3062 |
| *2* | GO:0030554 | adenyl nucleotide binding | 606 | 11 | 8.07 | 33 | 0.0034 | 0.1810 |
| *6* | GO:0016791 | phosphatase activity | 97 | 5 | 1.29 | 2 | 0.0870 | 0.0093 |

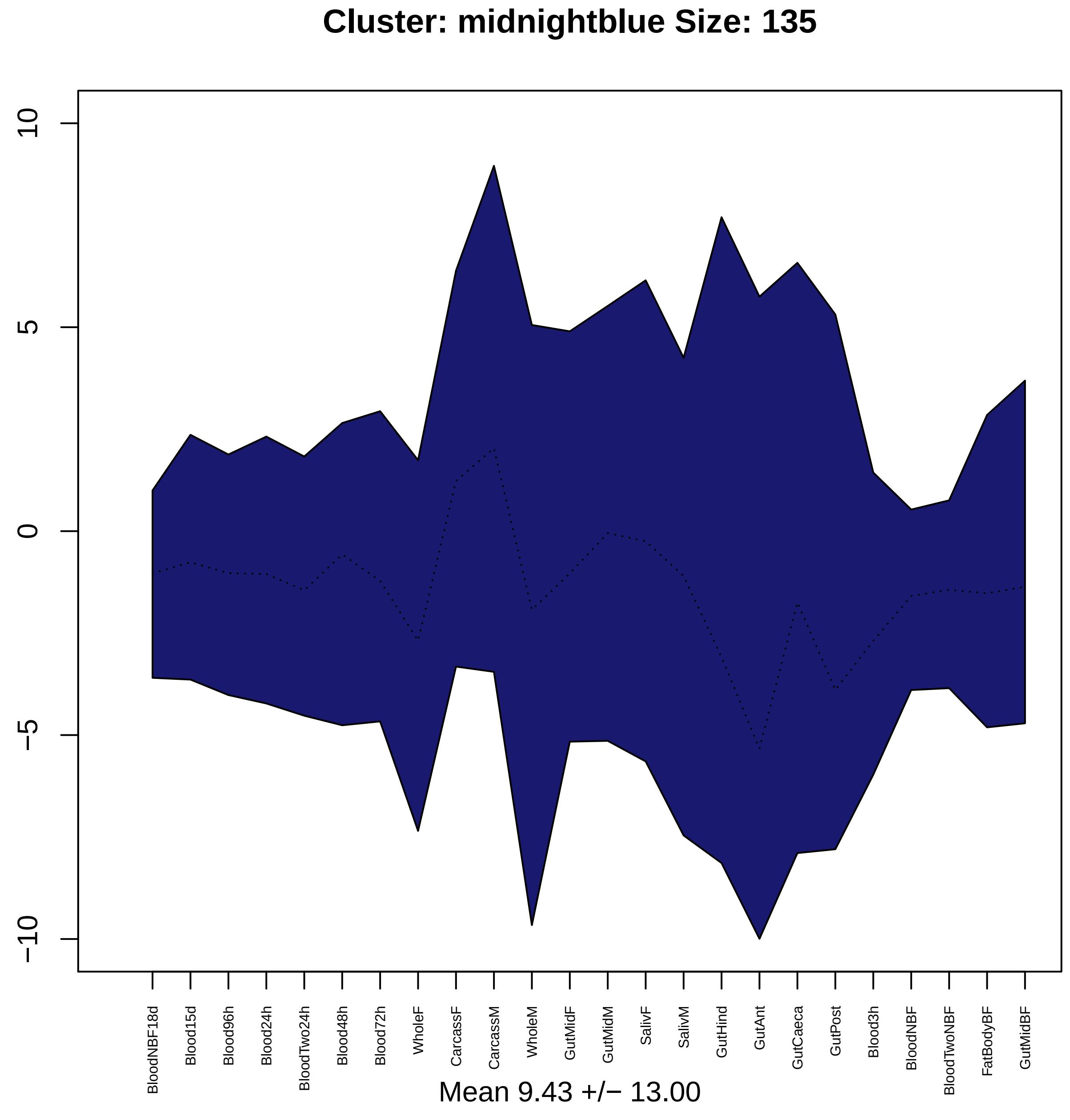

Cluster: midnightblue Size: 135
10
5
0
−5
−10
BloodNBF18d
Blood15d
Blood96h
Blood24h
BloodTwo24h
Blood48h
Blood72h
WholeF
CarcassF
CarcassM
WholeM
GutMidF
GutMidM
SalivF
SalivM
GutHind
GutAnt
GutCaeca
GutPost
Blood3h
BloodNBF
BloodTwoNBF
FatBodyBF
GutMidBF
Mean 9.43 +/− 13.00

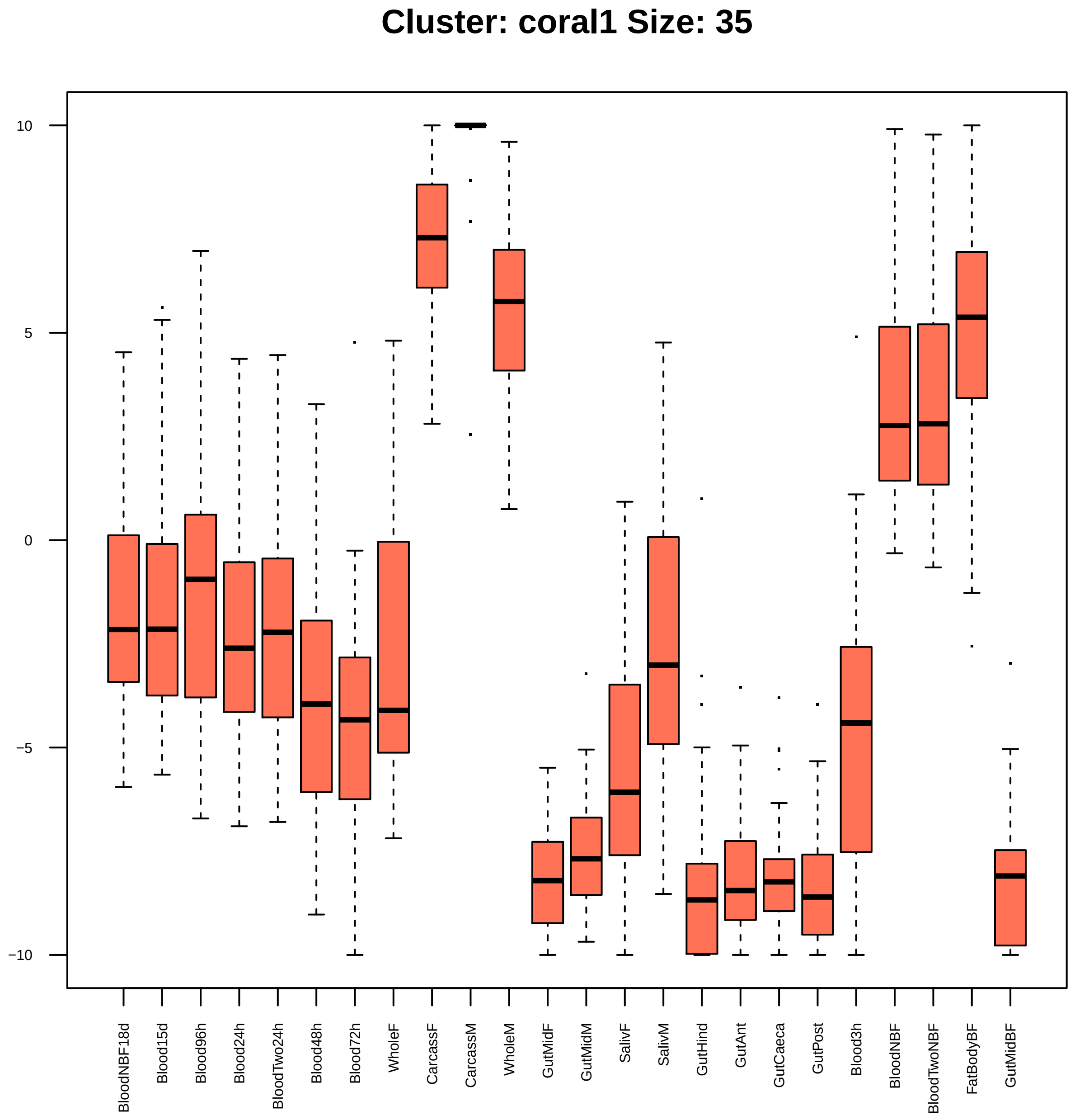
Cluster: coral1 Size: 35
10
5
0
−5
−10
BloodNBF18d
Blood15d
Blood96h
Blood24h
BloodTwo24h
Blood48h
Blood72h
WholeF
CarcassF
CarcassM
WholeM
GutMidF
GutMidM
SalivF
SalivM
GutHind
GutAnt
GutCaeca
GutPost
Blood3h
BloodNBF
BloodTwoNBF
FatBodyBF
GutMidBF

| | GO.ID | BPCluster: coral1 Size: 35 | Annotated | Significant | Expected | Rank in ClassicF | Weight01F | ClassicF |
|---|---|---|---|---|---|---|---|---|
| *1* | GO:0055114 | oxidation−reduction process | 545 | 6 | 1.54 | 1 | 0.0029 | 0.0029 |
| *2* | GO:0009058 | biosynthetic process | 1357 | 4 | 3.83 | 41 | 0.0053 | 0.5533 |

| | GO.ID | MFCluster: coral1 Size: 35 | Annotated | Significant | Expected | Rank in ClassicF | Weight01F | ClassicF |
|---|---|---|---|---|---|---|---|---|
| *1* | GO:0042302 | structural constituent of cuticle | 99 | 3 | 0.29 | 1 | 0.019 | 0.0028 |
| *3* | GO:0016747 | transferase activity, transferring acyl ... | 112 | 3 | 0.33 | 3 | 0.032 | 0.0040 |
| *12* | GO:0016491 | oxidoreductase activity | 523 | 6 | 1.52 | 2 | 0.059 | 0.0030 |

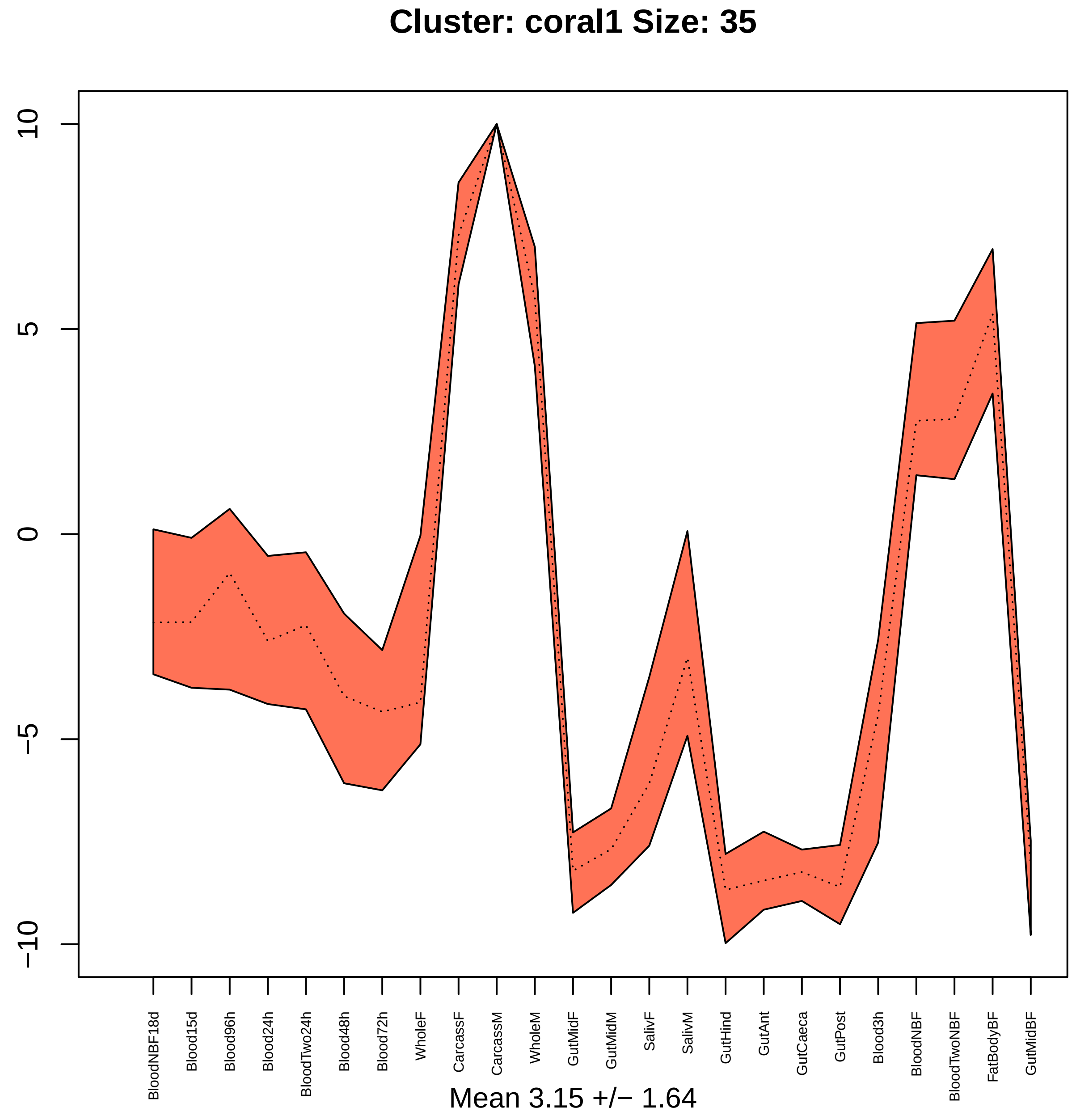
Cluster: coral1 Size: 35
10
5
0
−5
−10
BloodNBF18d
Blood15d
Blood96h
Blood24h
BloodTwo24h
Blood48h
Blood72h
WholeF
CarcassF
CarcassM
WholeM
GutMidF
GutMidM
SalivF
SalivM
GutHind
GutAnt
GutCaeca
GutPost
Blood3h
BloodNBF
BloodTwoNBF
FatBodyBF
GutMidBF
Mean 3.15 +/− 1.64

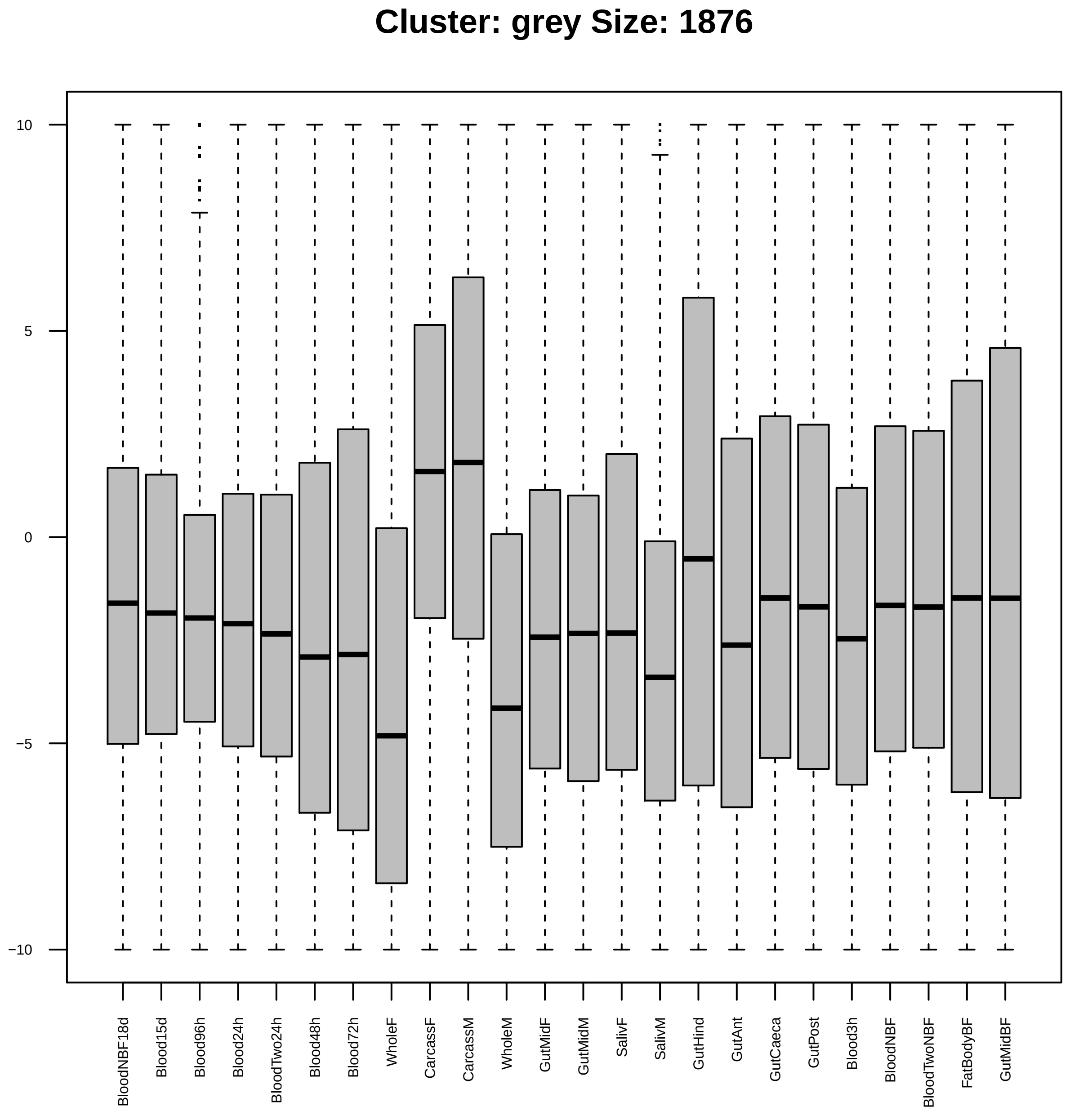

Cluster: grey Size: 1876
10
5
0
−5
−10
BloodNBF18d
Blood15d
Blood96h
Blood24h
BloodTwo24h
Blood48h
Blood72h
WholeF
CarcassF
CarcassM
WholeM
GutMidF
GutMidM
SalivF
SalivM
GutHind
GutAnt
GutCaeca
GutPost
Blood3h
BloodNBF
BloodTwoNBF
FatBodyBF
GutMidBF

| | GO.ID | BPCluster: grey Size: 1876 | Annotated | Significant | Expected | Rank in ClassicF | Weight01F | ClassicF |
|---|---|---|---|---|---|---|---|---|
| 1 | GO:0055085 | transmembrane transport | 469 | 105 | 84.95 | 36 | 6.7e−05 | 0.00852 |
| 2 | GO:1901606 | alpha−amino acid catabolic process | 27 | 12 | 4.89 | 7 | 0.0014 | 0.00140 |
| 3 | GO:0055059 | asymmetric neuroblast division | 21 | 10 | 3.80 | 10 | 0.0019 | 0.00185 |
| 4 | GO:0007298 | border follicle cell migration | 58 | 20 | 10.50 | 13 | 0.0021 | 0.00208 |
| 5 | GO:0050803 | regulation of synapse structure or activ... | 56 | 15 | 10.14 | 247 | 0.0029 | 0.06925 |
| 6 | GO:0006576 | cellular biogenic amine metabolic proces... | 16 | 8 | 2.90 | 17 | 0.0037 | 0.00367 |
| 7 | GO:0019318 | hexose metabolic process | 23 | 10 | 4.17 | 18 | 0.0042 | 0.00424 |
| 8 | GO:0017144 | drug metabolic process | 246 | 48 | 44.56 | 705 | 0.0057 | 0.30520 |
| 9 | GO:0007309 | oocyte axis specification | 60 | 16 | 10.87 | 235 | 0.0059 | 0.06418 |
| 10 | GO:0010721 | negative regulation of cell development | 23 | 9 | 4.17 | 59 | 0.0059 | 0.01460 |
| 11 | GO:0007391 | dorsal closure | 58 | 17 | 10.50 | 90 | 0.0065 | 0.02471 |
| 12 | GO:0035099 | hemocyte migration | 11 | 6 | 1.99 | 25 | 0.0070 | 0.00702 |
| 13 | GO:0006637 | acyl−CoA metabolic process | 11 | 6 | 1.99 | 26 | 0.0070 | 0.00702 |
| 14 | GO:0008340 | determination of adult lifespan | 68 | 21 | 12.32 | 28 | 0.0072 | 0.00716 |
| 15 | GO:0005975 | carbohydrate metabolic process | 210 | 63 | 38.04 | 1 | 0.0083 | 1.4e−05 |
| 21 | GO:0035088 | establishment or maintenance of apical/b... | 27 | 11 | 4.89 | 19 | 0.0126 | 0.00501 |
| 28 | GO:0006810 | transport | 1142 | 251 | 206.84 | 3 | 0.0193 | 0.00014 |

| | GO.ID | MFCluster: grey Size: 1876 | Annotated | Significant | Expected | Rank in ClassicF | Weight01F | ClassicF |
|---|---|---|---|---|---|---|---|---|
| *1* | GO:0030246 | carbohydrate binding | 60 | 22 | 10.69 | 1 | 0.00039 | 0.00039 |
| *2* | GO:0016903 | oxidoreductase activity, acting on the a... | 33 | 13 | 5.88 | 2 | 0.00090 | 0.00286 |
| *3* | GO:0005215 | transporter activity | 548 | 117 | 97.60 | 6 | 0.00721 | 0.01546 |
| *25* | GO:0038024 | cargo receptor activity | 17 | 8 | 3.03 | 3 | 0.07333 | 0.00525 |

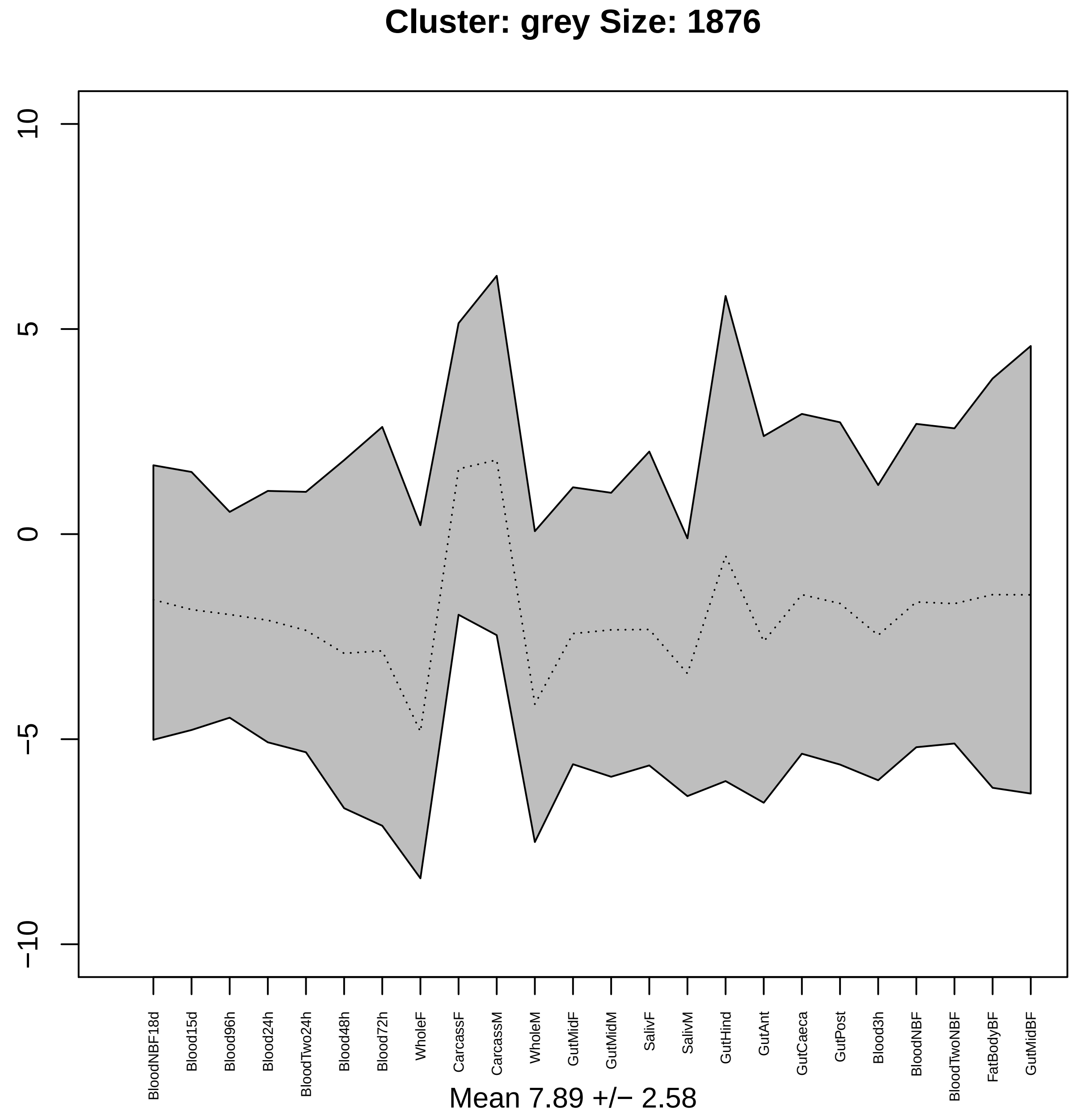

Cluster: grey Size: 1876
10
5
0
−5
−10
BloodNBF18d
Blood15d
Blood96h
Blood24h
BloodTwo24h
Blood48h
Blood72h
WholeF
CarcassF
CarcassM
WholeM
GutMidF
GutMidM
SalivF
SalivM
GutHind
GutAnt
GutCaeca
GutPost
Blood3h
BloodNBF
BloodTwoNBF
FatBodyBF
GutMidBF
Mean 7.89 +/− 2.58

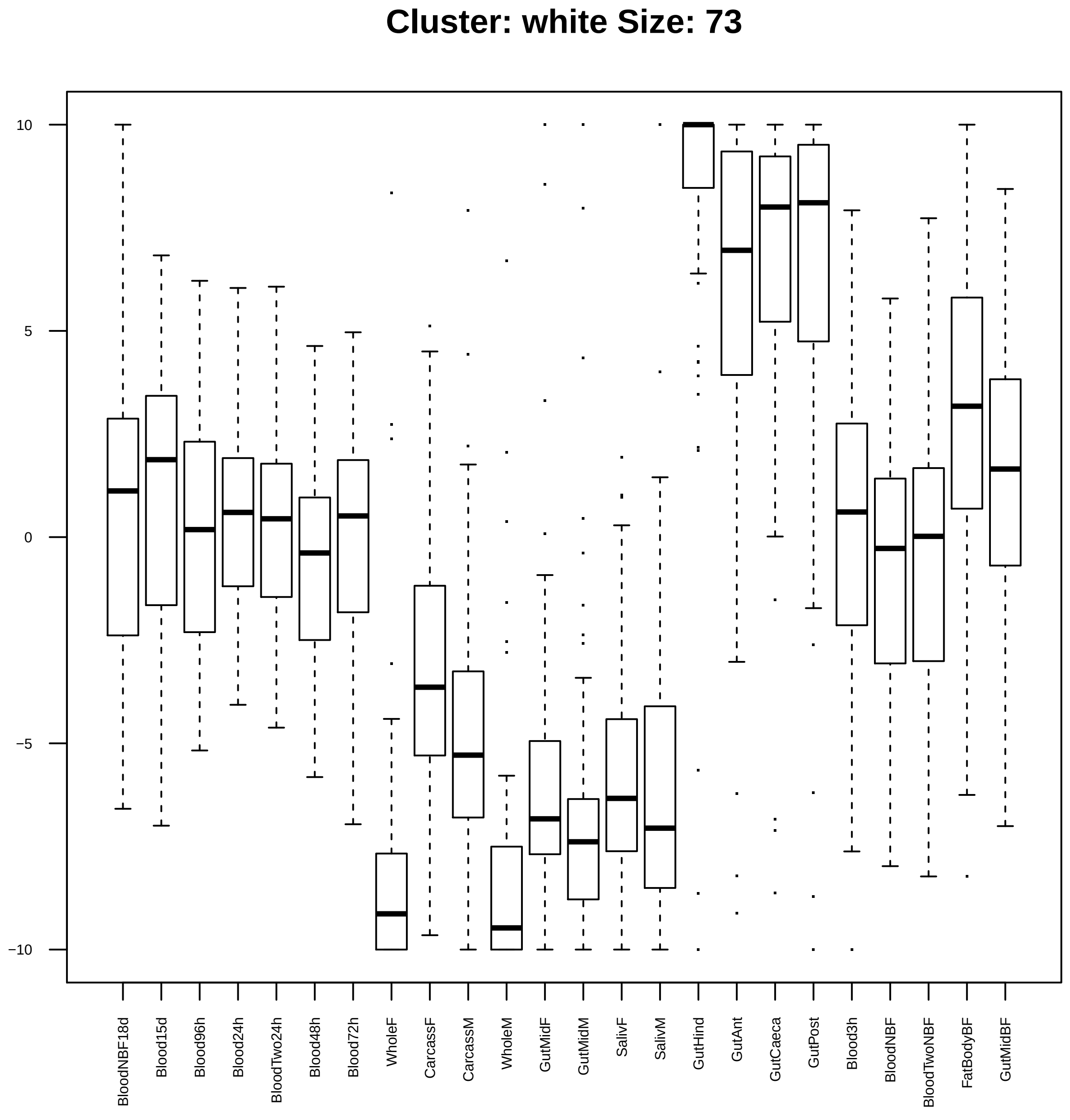

Cluster: white Size: 73
10
5
0
−5
−10
BloodNBF18d
Blood15d
Blood96h
Blood24h
BloodTwo24h
Blood48h
Blood72h
WholeF
CarcassF
CarcassM
WholeM
GutMidF
GutMidM
SalivF
SalivM
GutHind
GutAnt
GutCaeca
GutPost
Blood3h
BloodNBF
BloodTwoNBF
FatBodyBF
GutMidBF

| | GO.ID | BPCluster: white Size: 73 | Annotated | Significant | Expected | Rank in ClassicF | Weight01F | ClassicF |
|---|---|---|---|---|---|---|---|---|
| 3 | GO:0051298 | centrosome duplication | 51 | 4 | 0.42 | 33 | 0.0008 | 0.0008 |
| 4 | GO:0043161 | proteasome−mediated ubiquitin−dependent ... | 37 | 3 | 0.31 | 40 | 0.0035 | 0.0035 |
| 5 | GO:0009893 | positive regulation of metabolic process | 218 | 3 | 1.81 | 217 | 0.0083 | 0.2714 |

|  | GO.ID | MFCluster: white Size: 73 | Annotated | Significant | Expected | Rank in ClassicF | Weight01F | ClassicF |
|---|---|---|---|---|---|---|---|---|
| *2* | GO:0004298 | threonine−type endopeptidase activity | 14 | 2 | 0.1 | 3 | 0.0041 | 0.0041 |

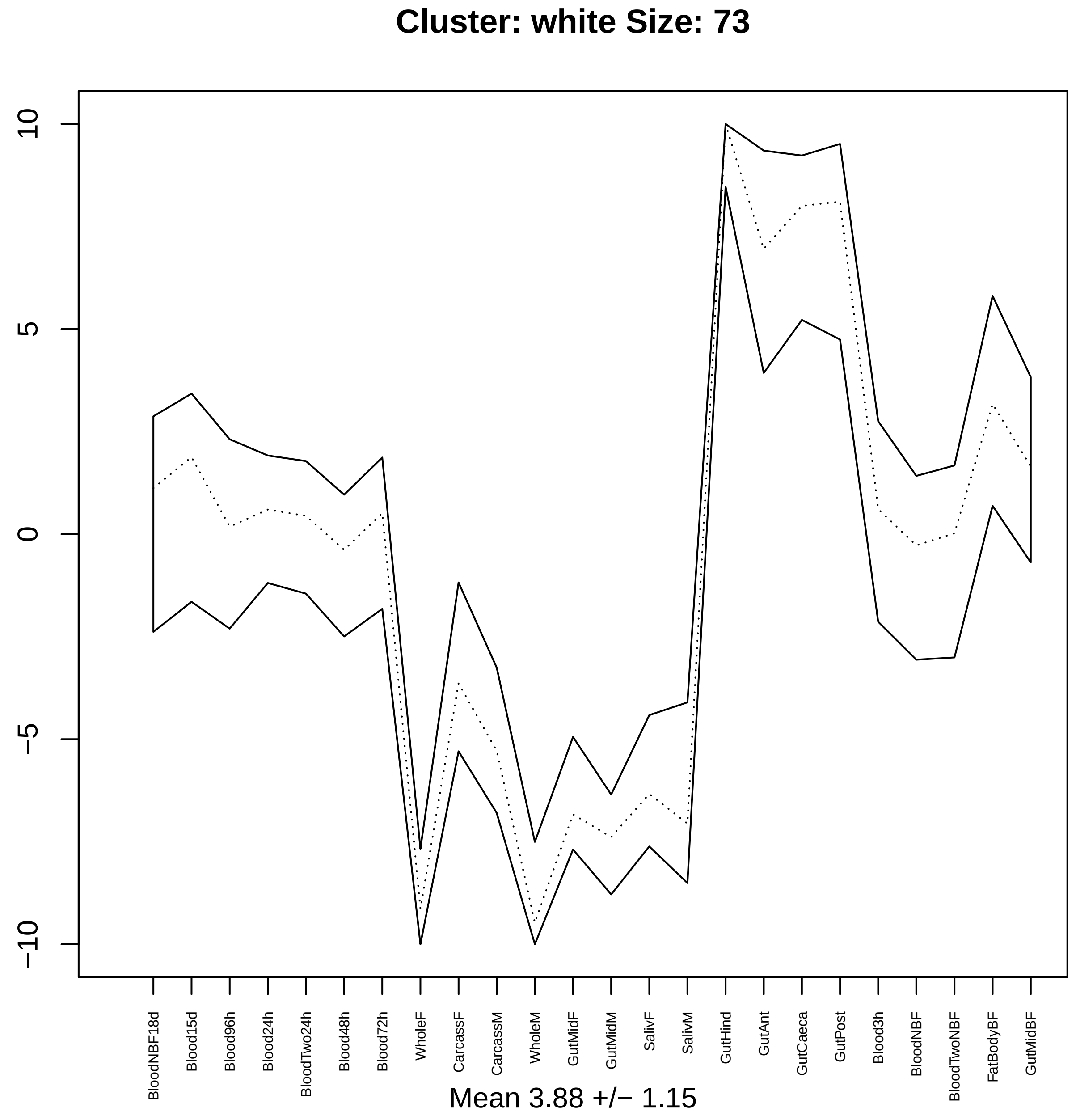

Cluster: white Size: 73
10
5
0
−5
−10
BloodNBF18d
Blood15d
Blood96h
Blood24h
BloodTwo24h
Blood48h
Blood72h
WholeF
CarcassF
CarcassM
WholeM
GutMidF
GutMidM
SalivF
SalivM
GutHind
GutAnt
GutCaeca
GutPost
Blood3h
BloodNBF
BloodTwoNBF
FatBodyBF
GutMidBF
Mean 3.88 +/− 1.15

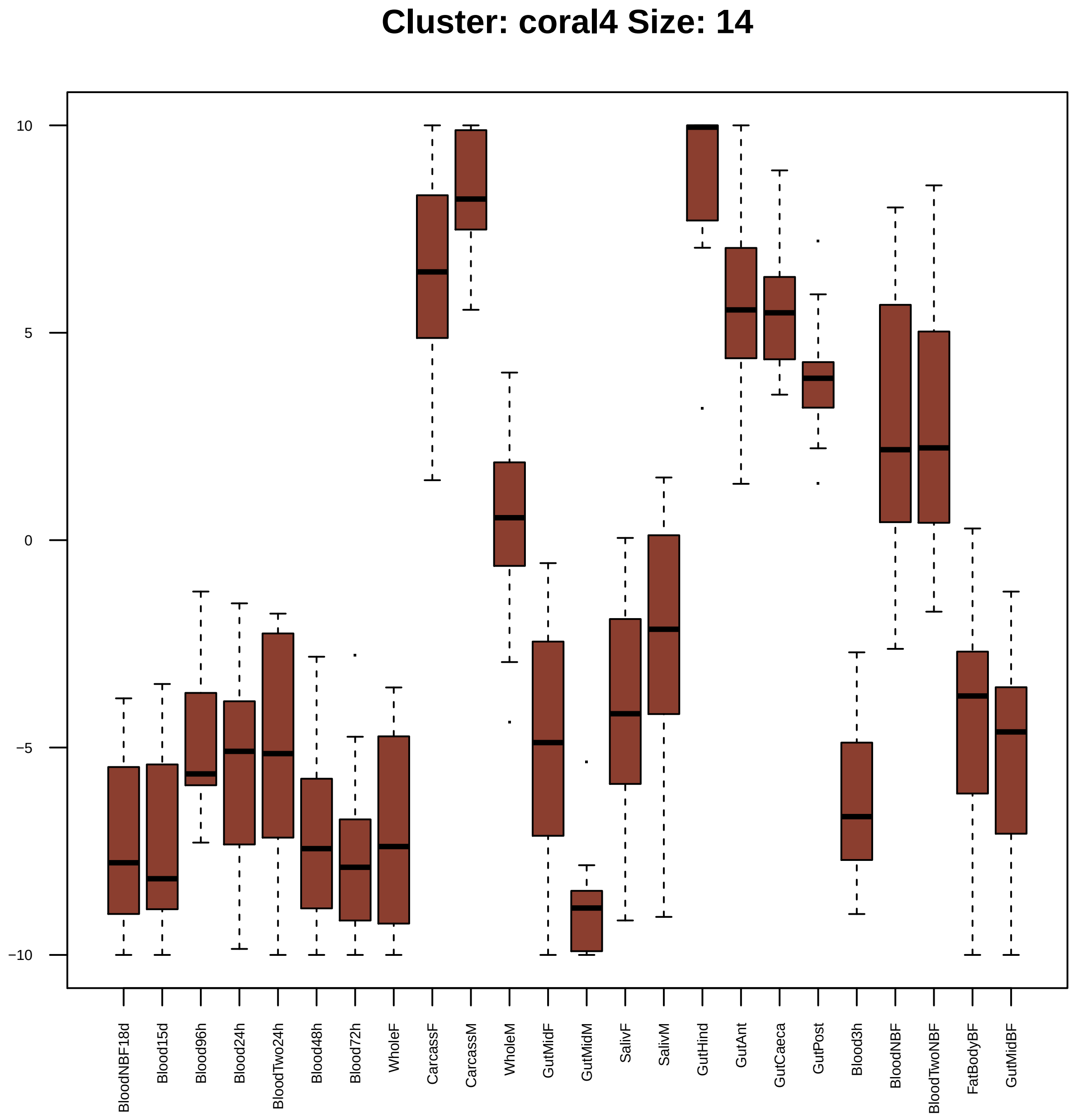

Cluster: coral4 Size: 14
10
5
0
−5
−10
BloodNBF18d
Blood15d
Blood96h
Blood24h
BloodTwo24h
Blood48h
Blood72h
WholeF
CarcassF
CarcassM
WholeM
GutMidF
GutMidM
SalivF
SalivM
GutHind
GutAnt
GutCaeca
GutPost
Blood3h
BloodNBF
BloodTwoNBF
FatBodyBF
GutMidBF

| | GO.ID | BPCluster: coral4 Size: 14 | Annotated | Significant | Expected | Rank in ClassicF | Weight01F | ClassicF |
|---|---|---|---|---|---|---|---|---|
| *3* | GO:0022900 | electron transport chain | 37 | 5 | 0.06 | 17 | 0.0033 | 2.2e−09 |
| *11* | GO:0006164 | purine nucleotide biosynthetic process | 68 | 3 | 0.12 | 47 | 1.0000 | 0.00018 |
| *12* | GO:0006139 | nucleobase−containing compound metabolic... | 1429 | 7 | 2.46 | 62 | 1.0000 | 0.00390 |
| *30* | GO:1901137 | carbohydrate derivative biosynthetic pro... | 161 | 3 | 0.28 | 59 | 1.0000 | 0.00225 |

| | GO.ID | MFCluster: coral4 Size: 14 | Annotated | Significant | Expected | Rank in ClassicF | Weight01F | ClassicF |
|---|---|---|---|---|---|---|---|---|
| *5* | GO:0003954 | NADH dehydrogenase activity | 25 | 6 | 0.04 | 1 | 0.0042 | 4.2e−13 |
| *19* | GO:0022804 | active transmembrane transporter activit... | 136 | 3 | 0.21 | 17 | 1.0000 | 0.00097 |
| *26* | GO:0015077 | monovalent inorganic cation transmembran... | 138 | 3 | 0.21 | 18 | 1.0000 | 0.00101 |
| *28* | GO:0015399 | primary active transmembrane transporter... | 76 | 3 | 0.12 | 15 | 1.0000 | 0.00017 |
| *30* | GO:0043492 | ATPase activity, coupled to movement of ... | 73 | 3 | 0.11 | 13 | 1.0000 | 0.00016 |

# Cluster: coral4 Size: 14

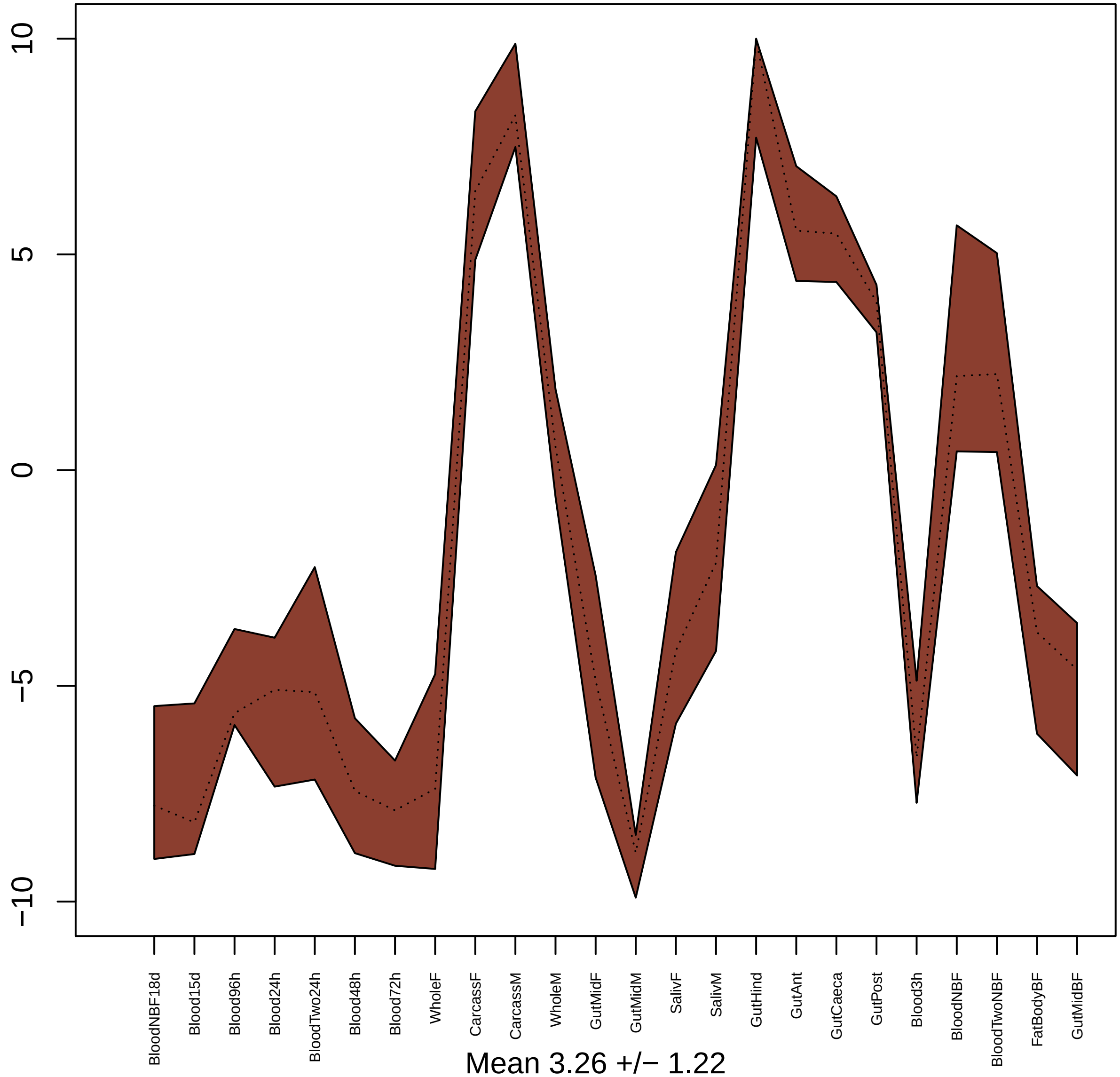


Mean 3.26 +/− 1.22

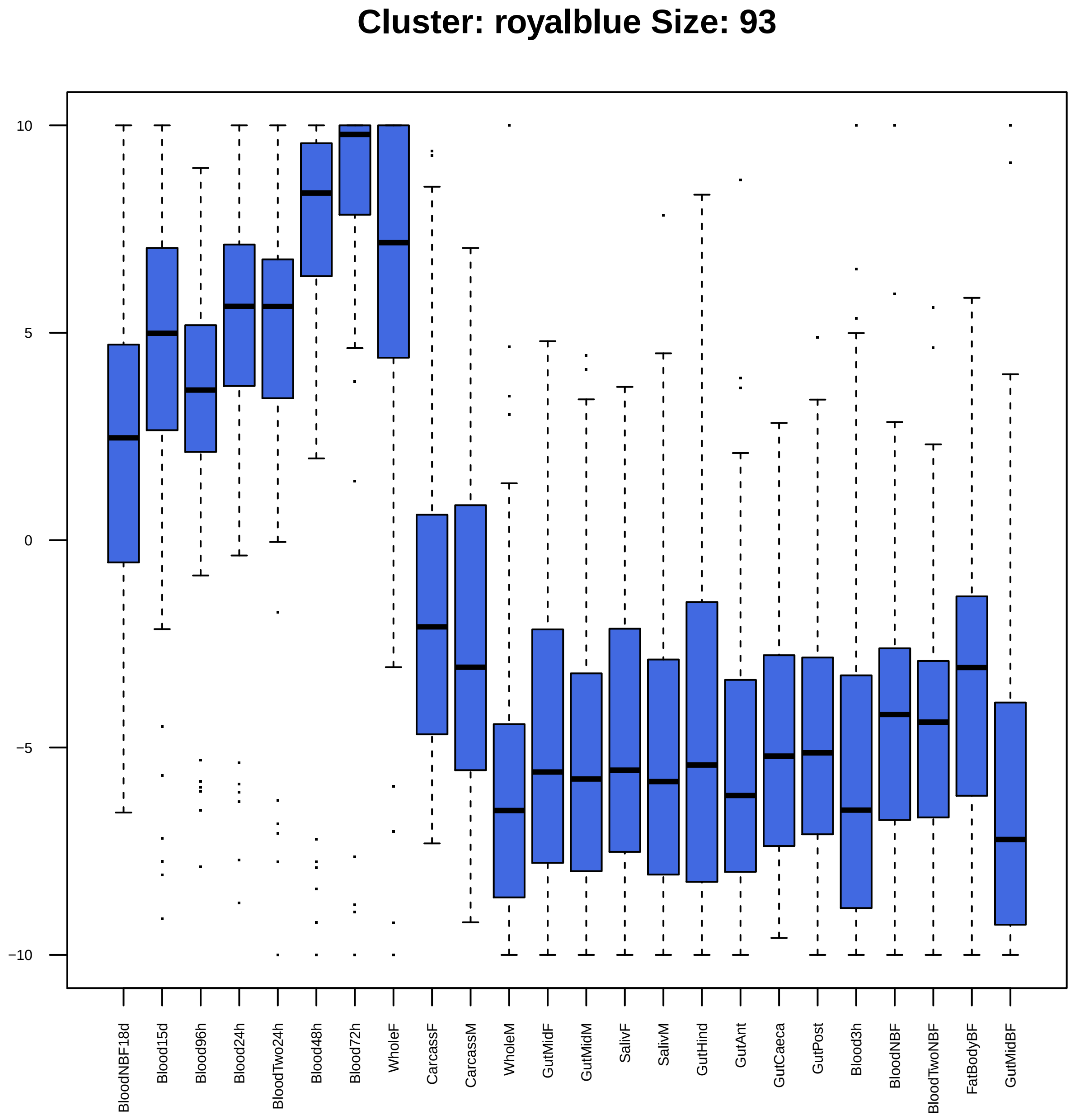
Cluster: royalblue Size: 93
10
5
0
−5
−10
BloodNBF18d
Blood15d
Blood96h
Blood24h
BloodTwo24h
Blood48h
Blood72h
WholeF
CarcassF
CarcassM
WholeM
GutMidF
GutMidM
SalivF
SalivM
GutHind
GutAnt
GutCaeca
GutPost
Blood3h
BloodNBF
BloodTwoNBF
FatBodyBF
GutMidBF

| | GO.ID | BPCluster: royalblue Size: 93 | Annotated | Significant | Expected | Rank in ClassicF | Weight01F | ClassicF |
|---|---|---|---|---|---|---|---|---|
| *1* | GO:0007447 | imaginal disc pattern formation | 47 | 3 | 0.36 | 17 | 0.00083 | 0.00545 |
| *2* | GO:0006606 | protein import into nucleus | 26 | 3 | 0.20 | 4 | 0.00098 | 0.00098 |
| *3* | GO:0033301 | cell cycle comprising mitosis without cy... | 11 | 2 | 0.08 | 12 | 0.00304 | 0.00304 |
| *4* | GO:0007099 | centriole replication | 15 | 2 | 0.12 | 18 | 0.00570 | 0.00570 |
| *5* | GO:0016318 | ommatidial rotation | 16 | 2 | 0.12 | 20 | 0.00648 | 0.00648 |
| *6* | GO:0006334 | nucleosome assembly | 17 | 2 | 0.13 | 22 | 0.00731 | 0.00731 |
| *7* | GO:0045448 | mitotic cell cycle, embryonic | 17 | 2 | 0.13 | 23 | 0.00731 | 0.00731 |
| *8* | GO:0007611 | learning or memory | 57 | 3 | 0.44 | 28 | 0.00744 | 0.00933 |
| *9* | GO:2000113 | negative regulation of cellular macromol... | 129 | 3 | 0.99 | 158 | 0.00752 | 0.07608 |
| *10* | GO:0008283 | cell proliferation | 153 | 4 | 1.18 | 70 | 0.00792 | 0.02928 |
| *11* | GO:0042059 | negative regulation of epidermal growth ... | 19 | 2 | 0.15 | 26 | 0.00910 | 0.00910 |

| | GO.ID | MFCluster: royalblue Size: 93 | Annotated | Significant | Expected | Rank in ClassicF | Weight01F | ClassicF |
|---|---|---|---|---|---|---|---|---|
| *2* | GO:0008536 | Ran GTPase binding | 16 | 2 | 0.15 | 11 | 0.0097 | 0.00966 |
| *3* | GO:0005515 | protein binding | 2143 | 33 | 20.21 | 7 | 0.0130 | 0.00080 |
| *5* | GO:0003676 | nucleic acid binding | 1152 | 21 | 10.87 | 8 | 0.0216 | 0.00154 |
| *19* | GO:0008565 | protein transporter activity | 28 | 4 | 0.26 | 4 | 0.1508 | 0.00013 |

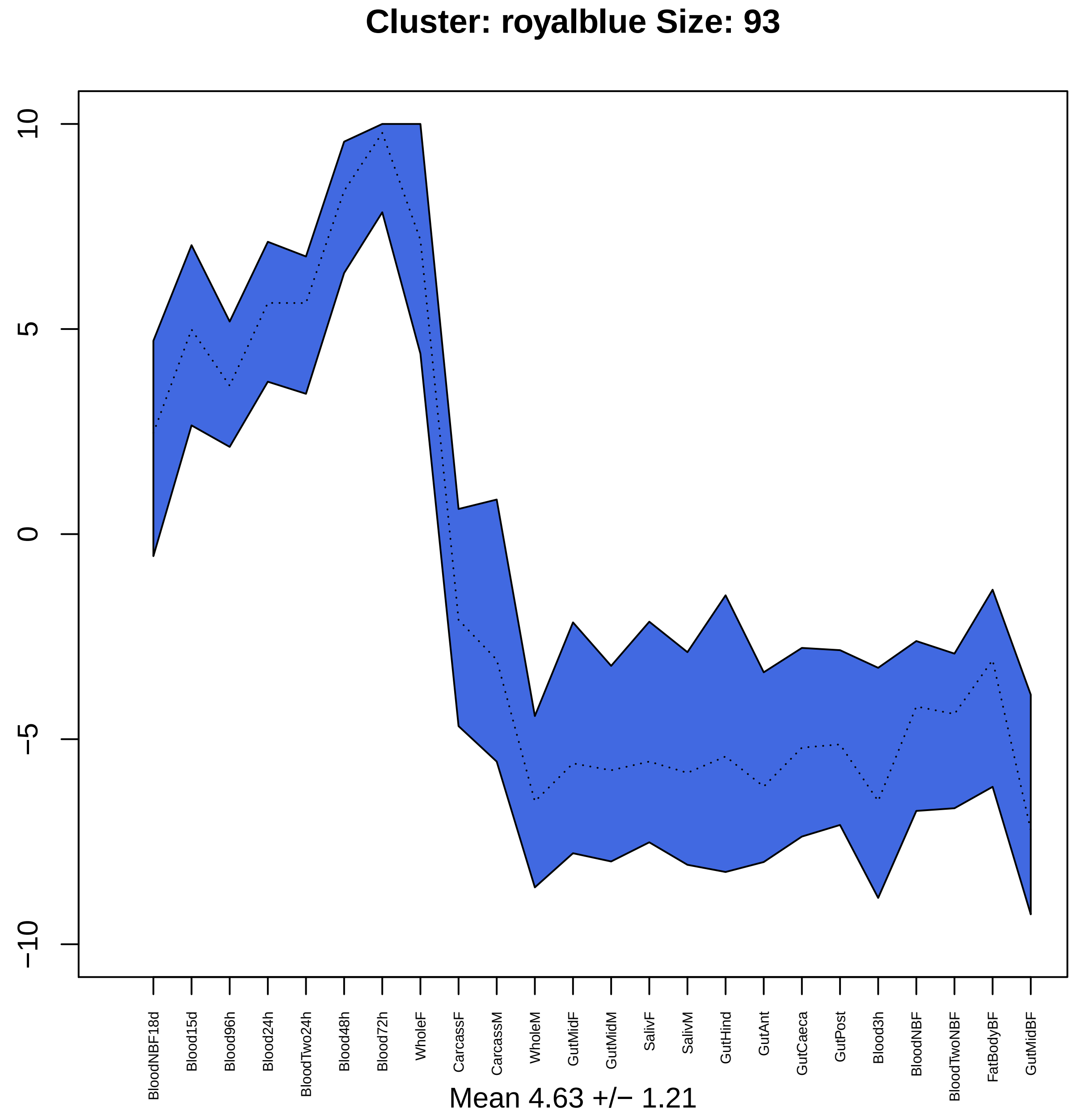

Cluster: royalblue Size: 93
10
5
0
−5
−10
BloodNBF18d
Blood15d
Blood96h
Blood24h
BloodTwo24h
Blood48h
Blood72h
WholeF
CarcassF
CarcassM
WholeM
GutMidF
GutMidM
SalivF
SalivM
GutHind
GutAnt
GutCaeca
GutPost
Blood3h
BloodNBF
BloodTwoNBF
FatBodyBF
GutMidBF
Mean 4.63 +/− 1.21

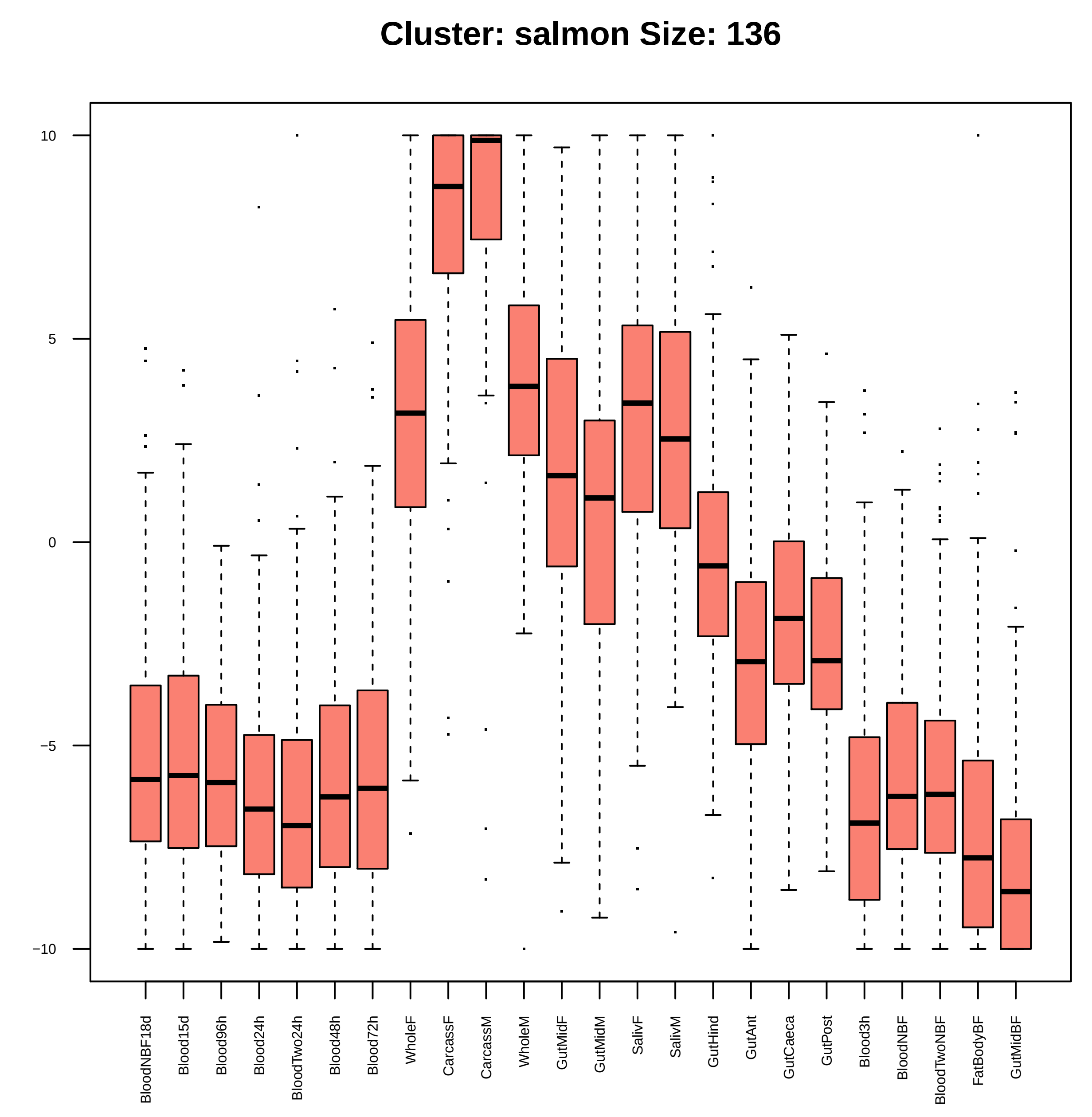

Cluster: salmon Size: 136
10
5
0
−5
−10
BloodNBF18d
Blood15d
Blood96h
Blood24h
BloodTwo24h
Blood48h
Blood72h
WholeF
CarcassF
CarcassM
WholeM
GutMidF
GutMidM
SalivF
SalivM
GutHind
GutAnt
GutCaeca
GutPost
Blood3h
BloodNBF
BloodTwoNBF
FatBodyBF
GutMidBF

|  | GO.ID | BPCluster: salmon Size: 136 | Annotated | Significant | Expected | Rank in ClassicF | Weight01F | ClassicF |
|---|---|---|---|---|---|---|---|---|
| 3 | GO:0009108 | coenzyme biosynthetic process | 60 | 6 | 0.88 | 11 | 0.00033 | 0.00023 |
| 4 | GO:0006412 | translation | 289 | 19 | 4.26 | 1 | 0.00066 | 2.7e−08 |
| 5 | GO:0006414 | translational elongation | 22 | 3 | 0.32 | 23 | 0.00390 | 0.00390 |
| 6 | GO:0006733 | oxidoreduction coenzyme metabolic proces... | 34 | 3 | 0.50 | 32 | 0.00425 | 0.01334 |
| 14 | GO:0032787 | monocarboxylic acid metabolic process | 88 | 5 | 1.30 | 27 | 0.05600 | 0.00936 |
| 16 | GO:0006732 | coenzyme metabolic process | 77 | 7 | 1.14 | 10 | 0.06789 | 0.00013 |
| 17 | GO:0009058 | biosynthetic process | 1357 | 34 | 20.00 | 16 | 0.06801 | 0.00060 |

| | GO.ID | MFCluster: salmon Size: 136 | Annotated | Significant | Expected | Rank in ClassicF | Weight01F | ClassicF |
|---|---|---|---|---|---|---|---|---|
| 2 | GO:0003746 | translation elongation factor activity | 14 | 3 | 0.18 | 3 | 0.00066 | 0.00066 |
| 3 | GO:0000287 | magnesium ion binding | 34 | 4 | 0.43 | 5 | 0.00086 | 0.00086 |
| 4 | GO:0016860 | intramolecular oxidoreductase activity | 23 | 3 | 0.29 | 7 | 0.00296 | 0.00296 |
| 5 | GO:0000049 | tRNA binding | 10 | 2 | 0.13 | 9 | 0.00678 | 0.00678 |
| 6 | GO:0016878 | acid−thiol ligase activity | 11 | 2 | 0.14 | 11 | 0.00822 | 0.00822 |
| 7 | GO:0016779 | nucleotidyltransferase activity | 61 | 4 | 0.78 | 10 | 0.00887 | 0.00745 |
| 8 | GO:0016645 | oxidoreductase activity, acting on the C... | 12 | 2 | 0.15 | 12 | 0.00978 | 0.00978 |
| 17 | GO:0016877 | ligase activity, forming carbon−sulfur b... | 17 | 3 | 0.22 | 6 | 0.07274 | 0.00120 |

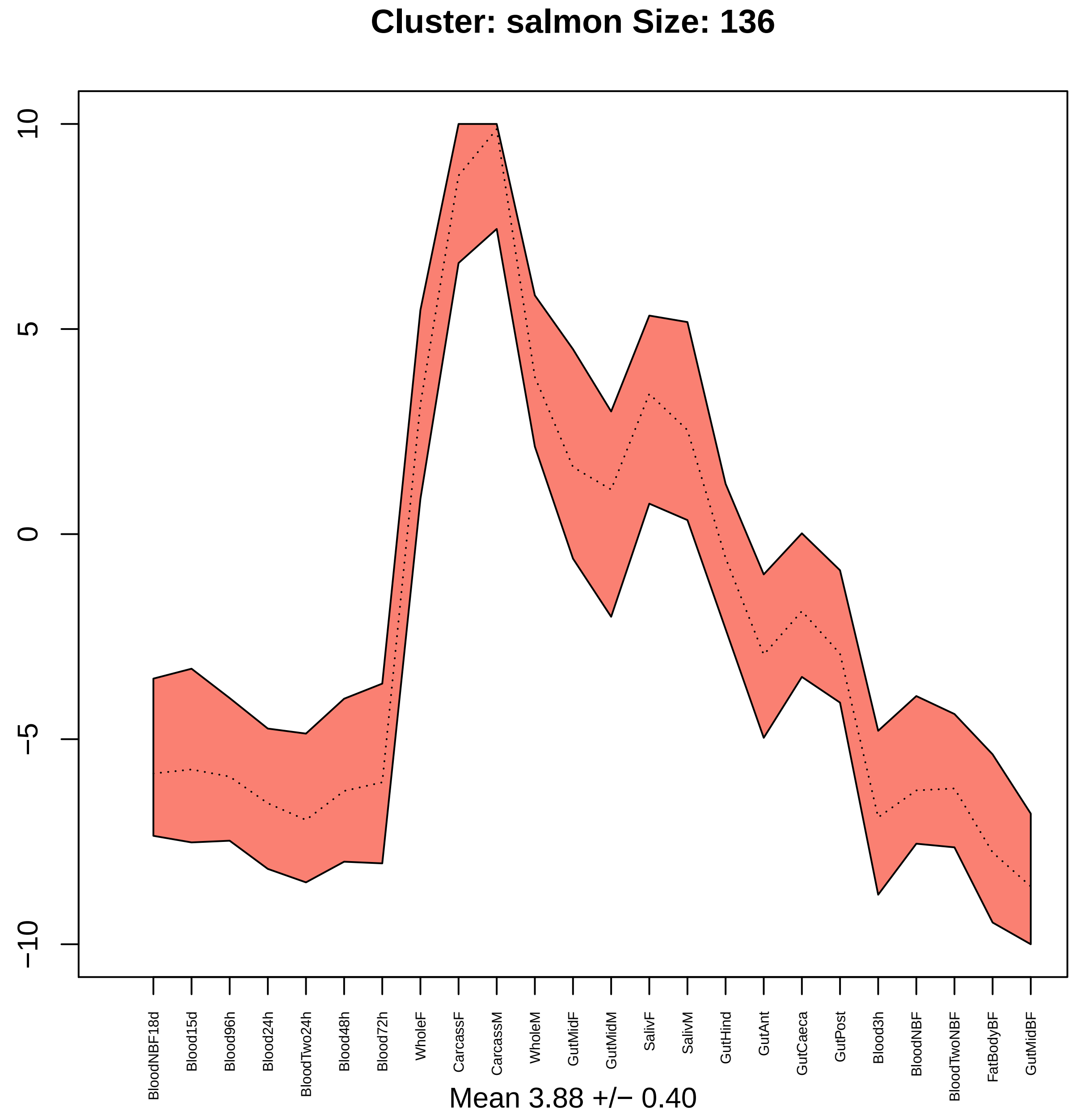
Cluster: salmon Size: 136
10
5
0
−5
−10
BloodNBF18d
Blood15d
Blood96h
Blood24h
BloodTwo24h
Blood48h
Blood72h
WholeF
CarcassF
CarcassM
WholeM
GutMidF
GutMidM
SalivF
SalivM
GutHind
GutAnt
GutCaeca
GutPost
Blood3h
BloodNBF
BloodTwoNBF
FatBodyBF
GutMidBF
Mean 3.88 +/− 0.40

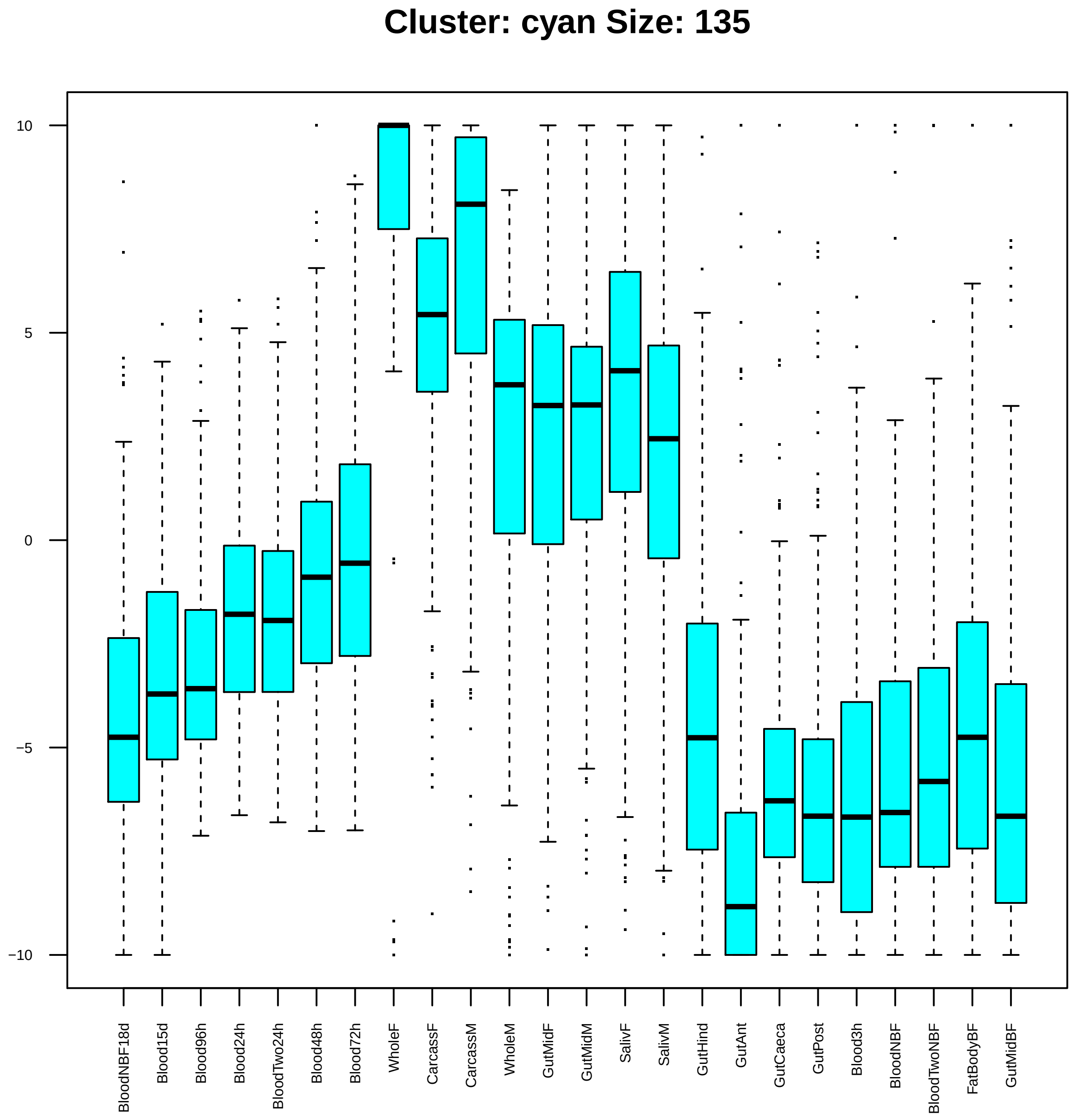
Cluster: cyan Size: 135
10
5
0
−5
−10
BloodNBF18d
Blood15d
Blood96h
Blood24h
BloodTwo24h
Blood48h
Blood72h
WholeF
CarcassF
CarcassM
WholeM
GutMidF
GutMidM
SalivF
SalivM
GutHind
GutAnt
GutCaeca
GutPost
Blood3h
BloodNBF
BloodTwoNBF
FatBodyBF
GutMidBF

| | GO.ID | BPCluster: cyan Size: 135 | Annotated | Significant | Expected | Rank in ClassicF | Weight01F | ClassicF |
|---|---|---|---|---|---|---|---|---|
| 1 | GO:0022008 | neurogenesis | 706 | 23 | 10.07 | 19 | 6.7e−06 | 0.00010 |
| 2 | GO:0000398 | mRNA splicing, via spliceosome | 134 | 11 | 1.91 | 9 | 0.00034 | 2.6e−06 |
| 3 | GO:0034472 | snRNA 3'−end processing | 11 | 3 | 0.16 | 32 | 0.00043 | 0.00043 |
| 4 | GO:0000381 | regulation of alternative mRNA splicing,... | 32 | 4 | 0.46 | 42 | 0.00103 | 0.00103 |
| 5 | GO:0016246 | RNA interference | 19 | 3 | 0.27 | 57 | 0.00231 | 0.00231 |
| 6 | GO:0007517 | muscle organ development | 66 | 5 | 0.94 | 58 | 0.00239 | 0.00239 |
| 7 | GO:0007095 | mitotic G2 DNA damage checkpoint | 43 | 4 | 0.61 | 62 | 0.00313 | 0.00313 |
| 8 | GO:0000956 | nuclear−transcribed mRNA catabolic proce... | 20 | 4 | 0.29 | 20 | 0.00808 | 0.00016 |
| 9 | GO:0019751 | polyol metabolic process | 10 | 2 | 0.14 | 91 | 0.00841 | 0.00841 |
| 10 | GO:0045815 | positive regulation of gene expression, ... | 10 | 2 | 0.14 | 92 | 0.00841 | 0.00841 |
| 11 | GO:0000288 | nuclear−transcribed mRNA catabolic proce... | 10 | 2 | 0.14 | 93 | 0.00841 | 0.00841 |
| 13 | GO:0000075 | cell cycle checkpoint | 64 | 6 | 0.91 | 27 | 0.01362 | 0.00028 |
| 22 | GO:1901991 | negative regulation of mitotic cell cycl... | 60 | 6 | 0.86 | 23 | 0.01976 | 0.00019 |

| | GO.ID | MFCluster: cyan Size: 135 | Annotated | Significant | Expected | Rank in ClassicF | Weight01F | ClassicF |
|---|---|---|---|---|---|---|---|---|
| *1* | GO:0004004 | ATP−dependent RNA helicase activity | 28 | 3 | 0.40 | 4 | 0.0074 | 0.0074 |
| *12* | GO:0003676 | nucleic acid binding | 1152 | 28 | 16.62 | 1 | 0.0720 | 0.0029 |
| *13* | GO:0000166 | nucleotide binding | 998 | 24 | 14.40 | 2 | 0.0758 | 0.0071 |

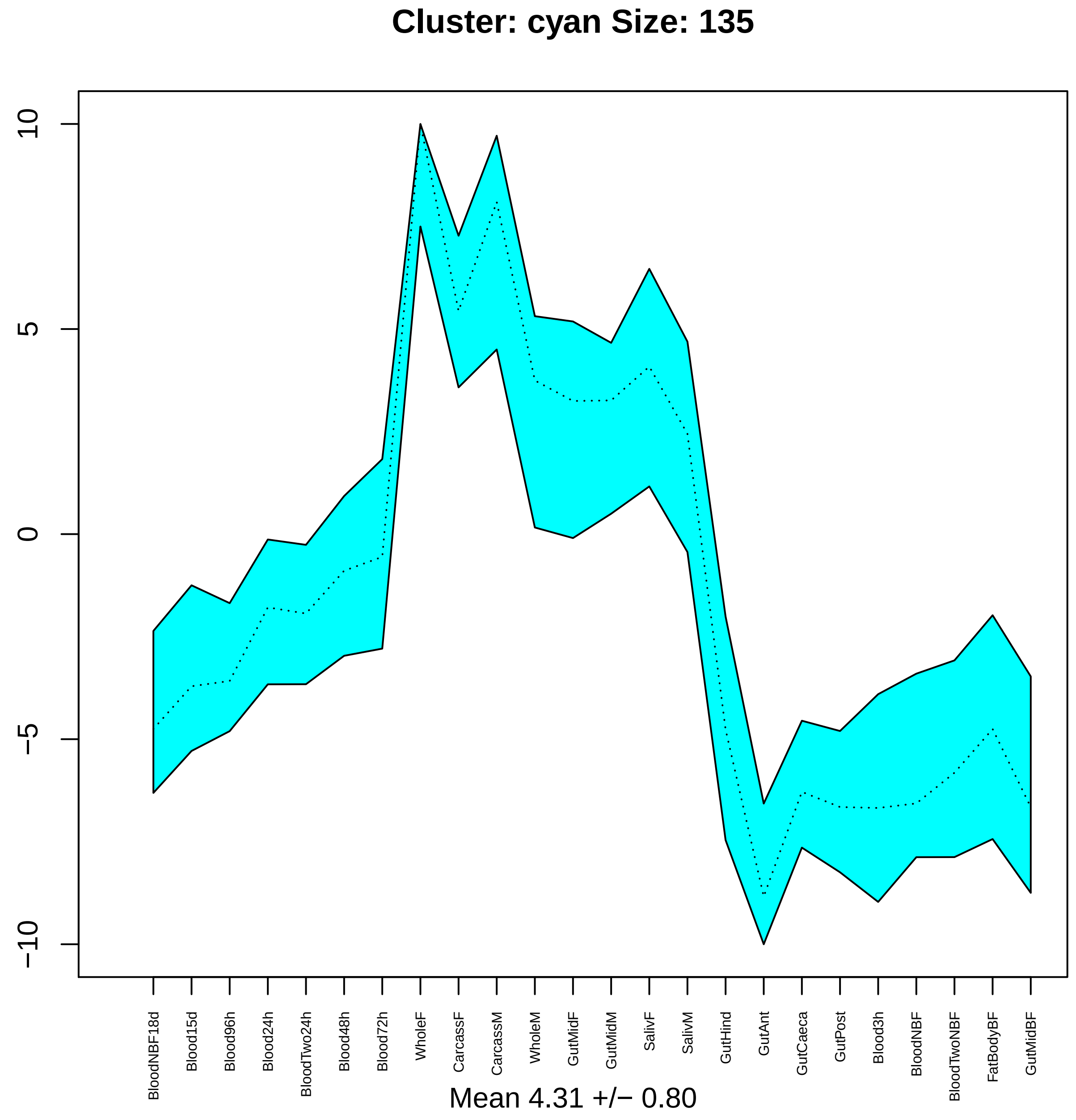
Cluster: cyan Size: 135
10
5
0
−5
−10
BloodNBF18d
Blood15d
Blood96h
Blood24h
BloodTwo24h
Blood48h
Blood72h
WholeF
CarcassF
CarcassM
WholeM
GutMidF
GutMidM
SalivF
SalivM
GutHind
GutAnt
GutCaeca
GutPost
Blood3h
BloodNBF
BloodTwoNBF
FatBodyBF
GutMidBF
Mean 4.31 +/− 0.80

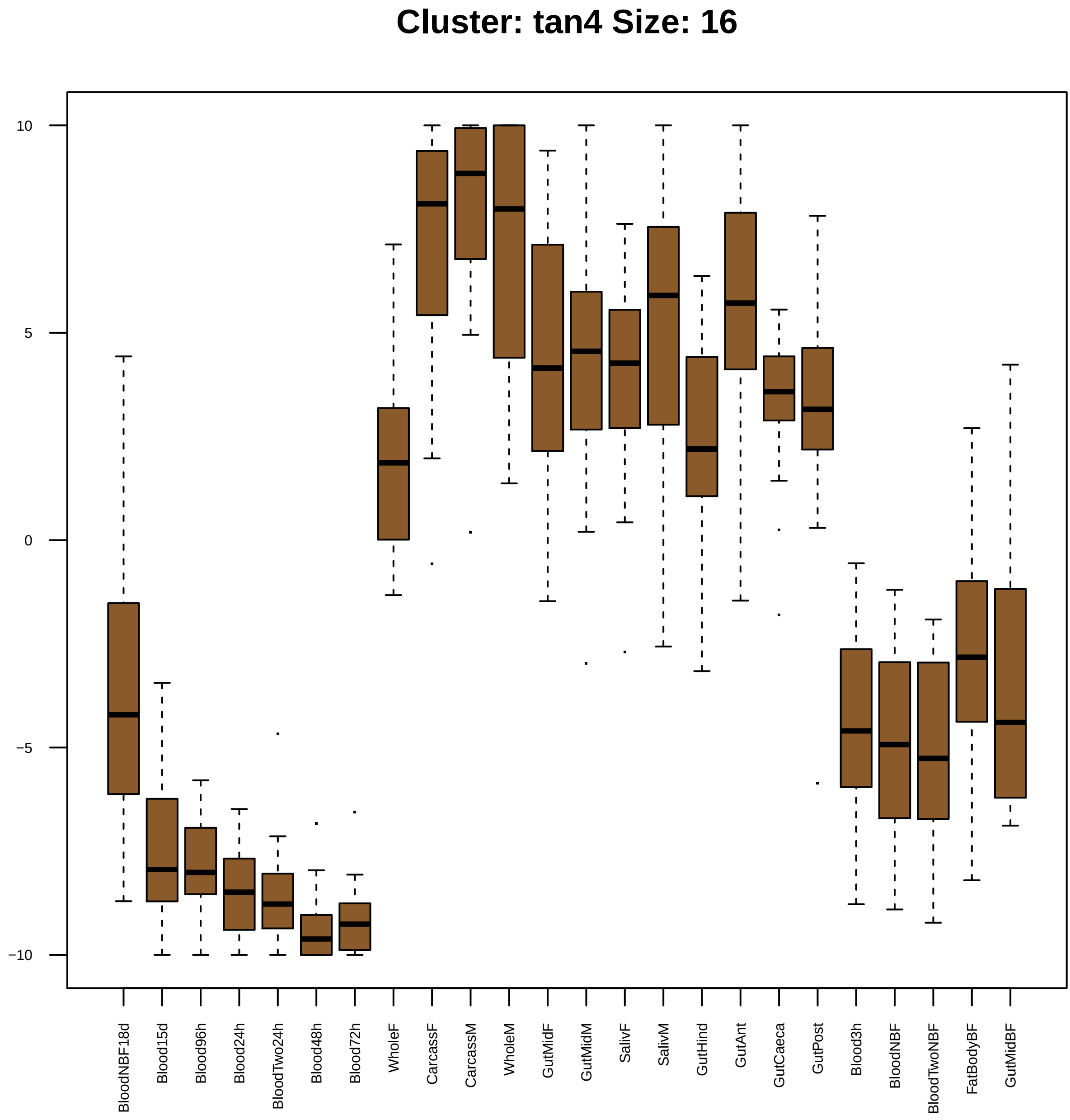

Cluster: tan4 Size: 16
10
5
0
−5
−10
BloodNBF18d
Blood15d
Blood96h
Blood24h
BloodTwo24h
Blood48h
Blood72h
WholeF
CarcassF
CarcassM
WholeM
GutMidF
GutMidM
SalivF
SalivM
GutHind
GutAnt
GutCaeca
GutPost
Blood3h
BloodNBF
BloodTwoNBF
FatBodyBF
GutMidBF

| | GO.ID | BPCluster: tan4 Size: 16 | Annotated | Significant | Expected | Rank in ClassicF | Weight01F | ClassicF |
|---|---|---|---|---|---|---|---|---|
| *10* | GO:0009416 | response to light stimulus | 65 | 2 | 0.08 | 2 | 0.057 | 0.0028 |

|  | GO.ID | MFCluster: tan4 Size: 16 | Annotated | Significant | Expected | Rank in ClassicF | Weight01F | ClassicF |
|---|---|---|---|---|---|---|---|---|
| *1* | GO:0046961 | proton−transporting ATPase activity, rot... | 21 | 2 | 0.03 | 1 | 0.00029 | 0.00029 |
| *23* | GO:0043492 | ATPase activity, coupled to movement of ... | 73 | 2 | 0.09 | 8 | 1.00000 | 0.00348 |

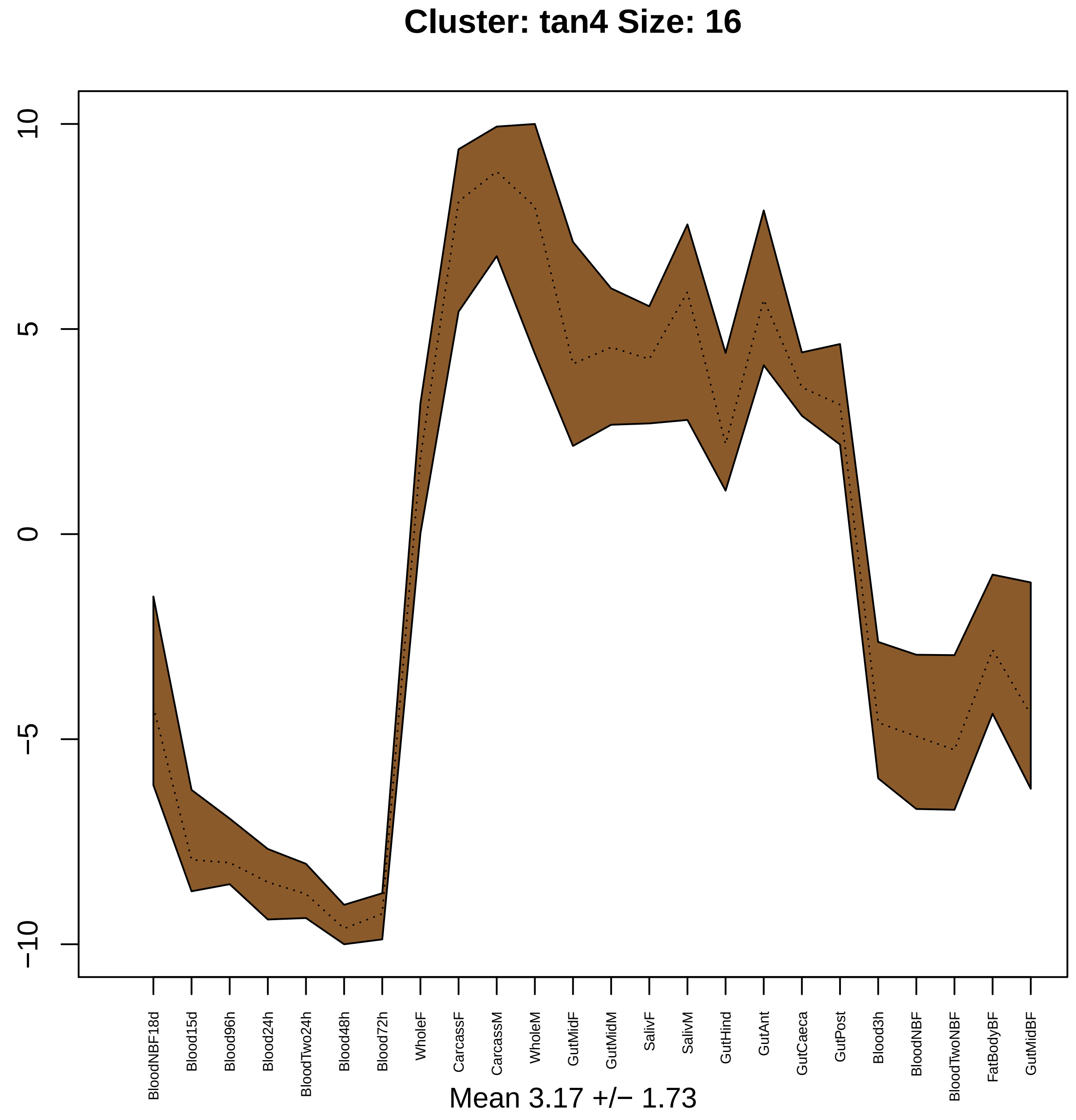
Cluster: tan4 Size: 16
10
5
0
−5
−10
BloodNBF18d
Blood15d
Blood96h
Blood24h
BloodTwo24h
Blood48h
Blood72h
WholeF
CarcassF
CarcassM
WholeM
GutMidF
GutMidM
SalivF
SalivM
GutHind
GutAnt
GutCaeca
GutPost
Blood3h
BloodNBF
BloodTwoNBF
FatBodyBF
GutMidBF
Mean 3.17 +/− 1.73

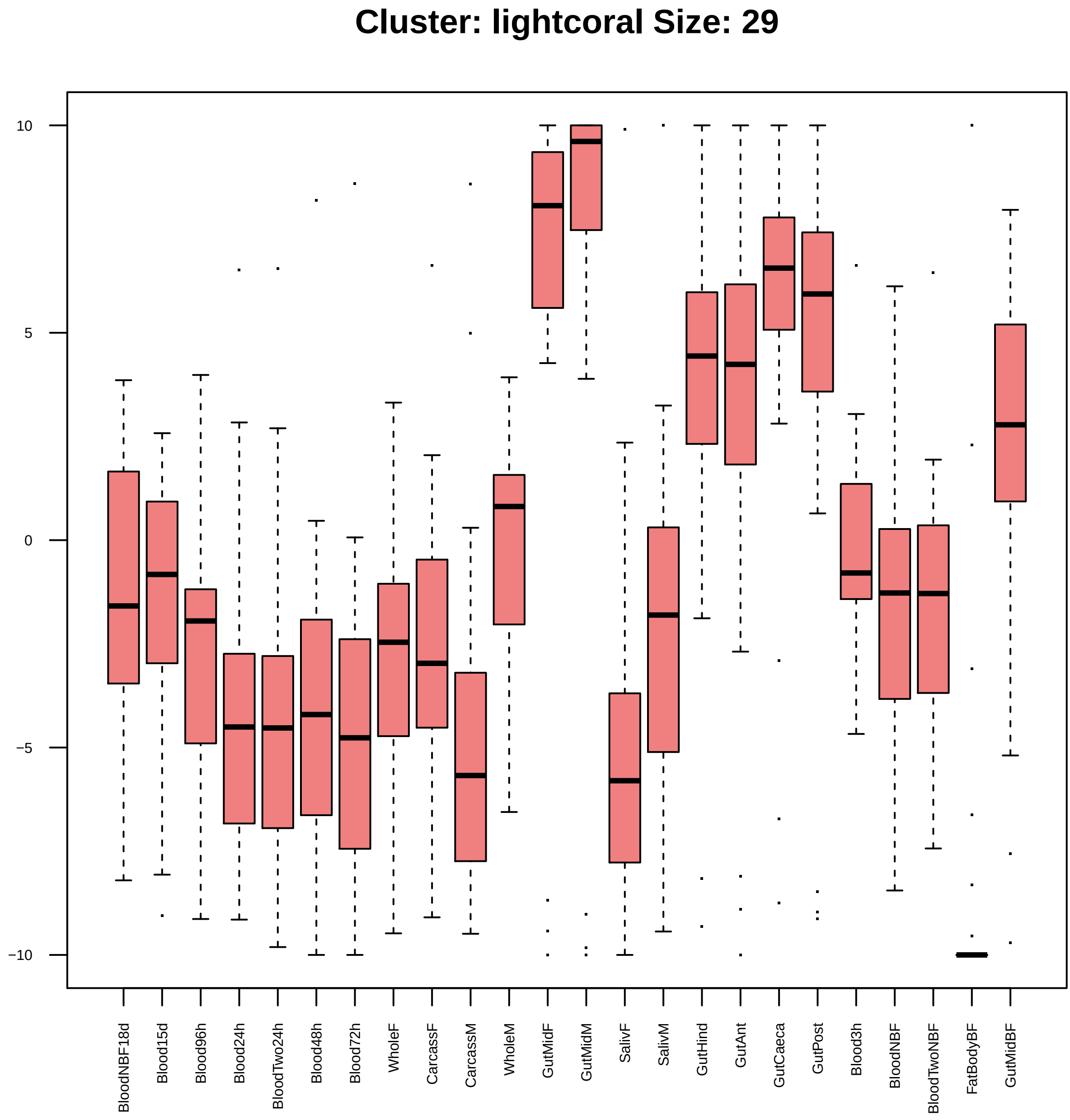

Cluster: lightcoral Size: 29
10
5
0
−5
−10
BloodNBF18d
Blood15d
Blood96h
Blood24h
BloodTwo24h
Blood48h
Blood72h
WholeF
CarcassF
CarcassM
WholeM
GutMidF
GutMidM
SalivF
SalivM
GutHind
GutAnt
GutCaeca
GutPost
Blood3h
BloodNBF
BloodTwoNBF
FatBodyBF
GutMidBF

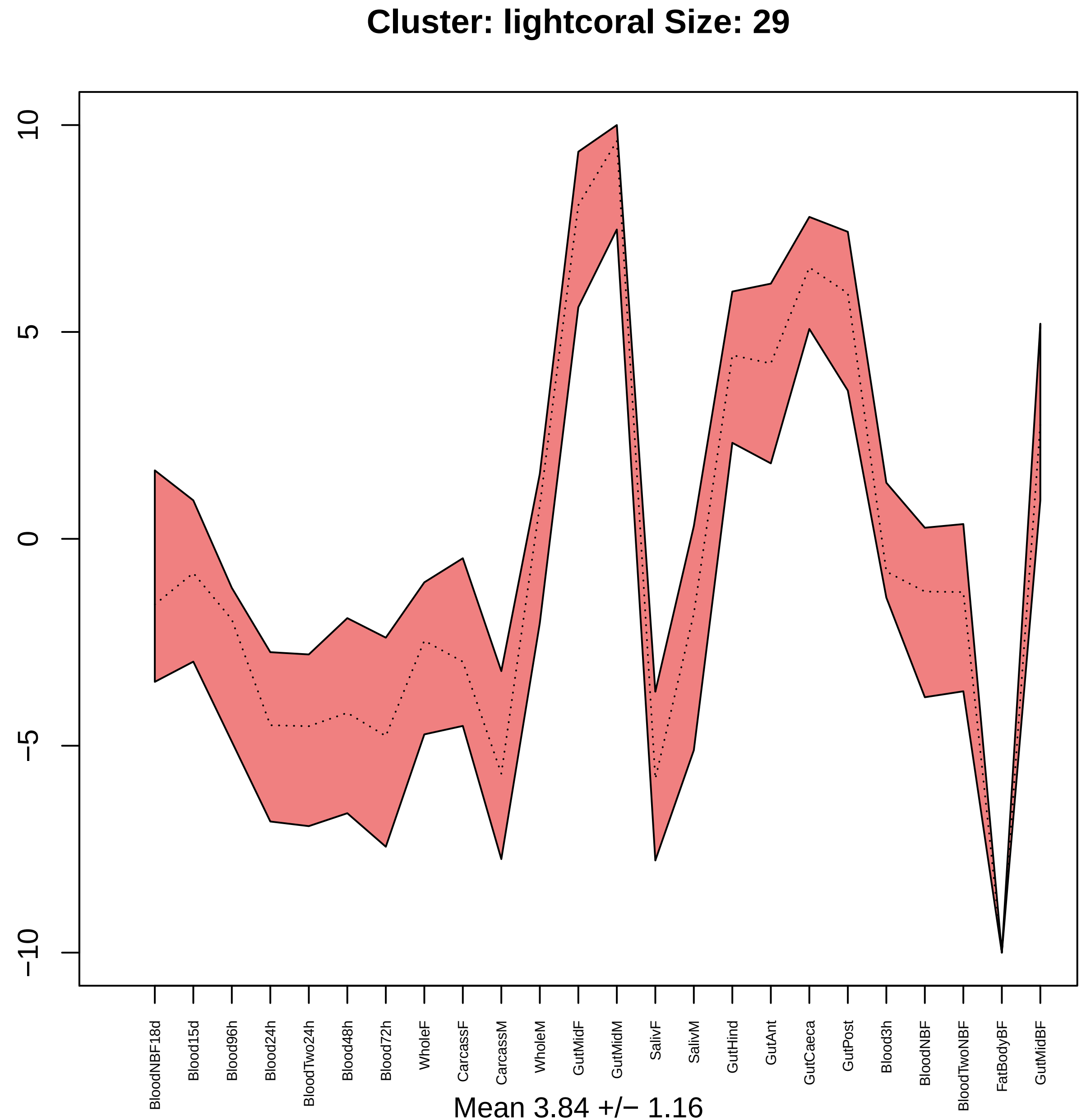
Cluster: lightcoral Size: 29
10
5
0
−5
−10
BloodNBF18d
Blood15d
Blood96h
Blood24h
BloodTwo24h
Blood48h
Blood72h
WholeF
CarcassF
CarcassM
WholeM
GutMidF
GutMidM
SalivF
SalivM
GutHind
GutAnt
GutCaeca
GutPost
Blood3h
BloodNBF
BloodTwoNBF
FatBodyBF
GutMidBF
Mean 3.84 +/− 1.16

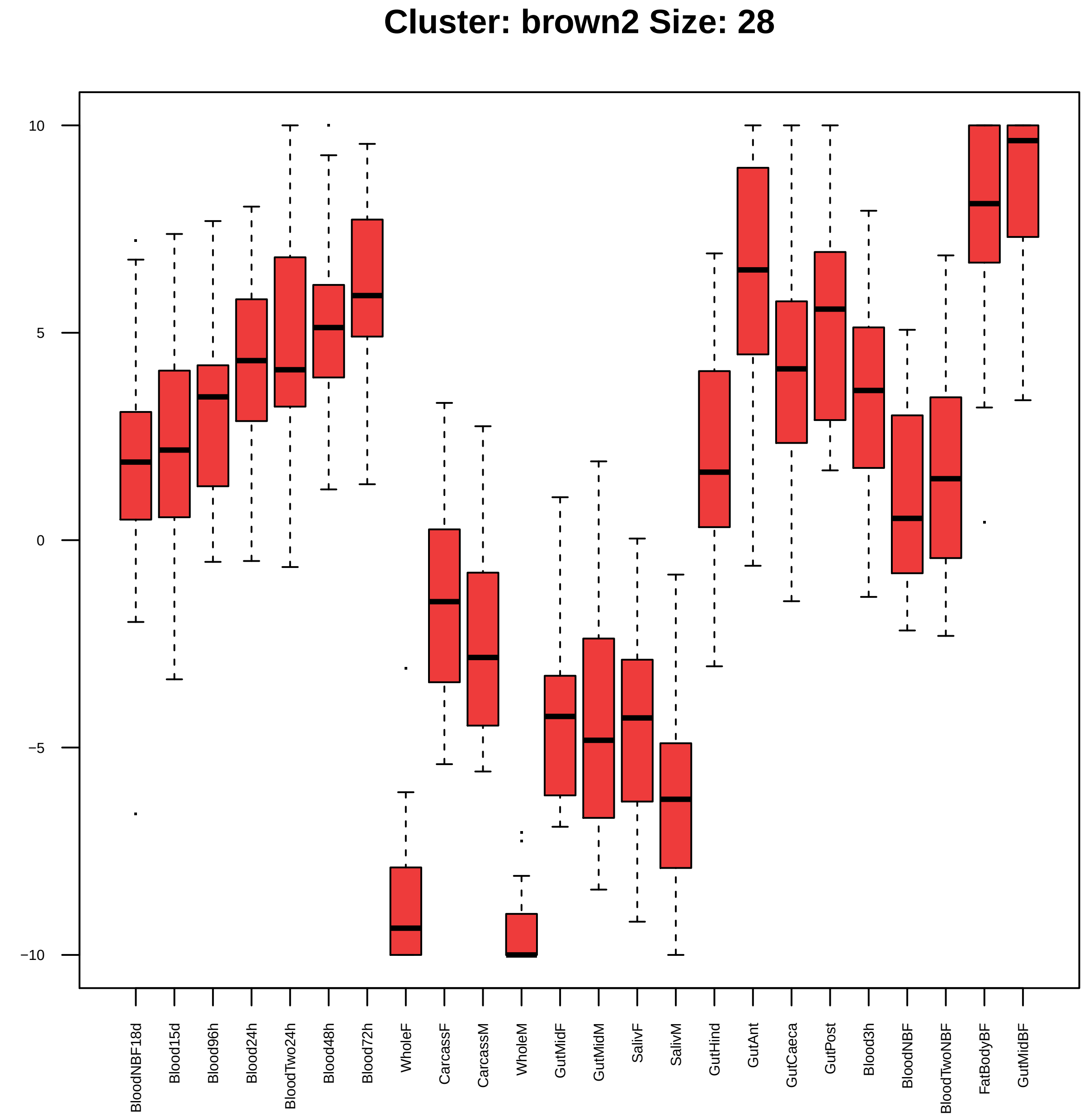

Cluster: brown2 Size: 28
10
5
0
−5
−10
BloodNBF18d
Blood15d
Blood96h
Blood24h
BloodTwo24h
Blood48h
Blood72h
WholeF
CarcassF
CarcassM
WholeM
GutMidF
GutMidM
SalivF
SalivM
GutHind
GutAnt
GutCaeca
GutPost
Blood3h
BloodNBF
BloodTwoNBF
FatBodyBF
GutMidBF

| | GO.ID | BPCluster: brown2 Size: 28 | Annotated | Significant | Expected | Rank in ClassicF | Weight01F | ClassicF |
|---|---|---|---|---|---|---|---|---|
| *1* | GO:0030177 | positive regulation of Wnt signaling pat... | 11 | 2 | 0.02 | 1 | 0.00018 | 0.00018 |
| *2* | GO:0006367 | transcription initiation from RNA polyme... | 39 | 2 | 0.07 | 3 | 0.00231 | 0.00231 |
| *3* | GO:0048569 | post−embryonic animal organ development | 222 | 3 | 0.42 | 9 | 0.01131 | 0.00725 |

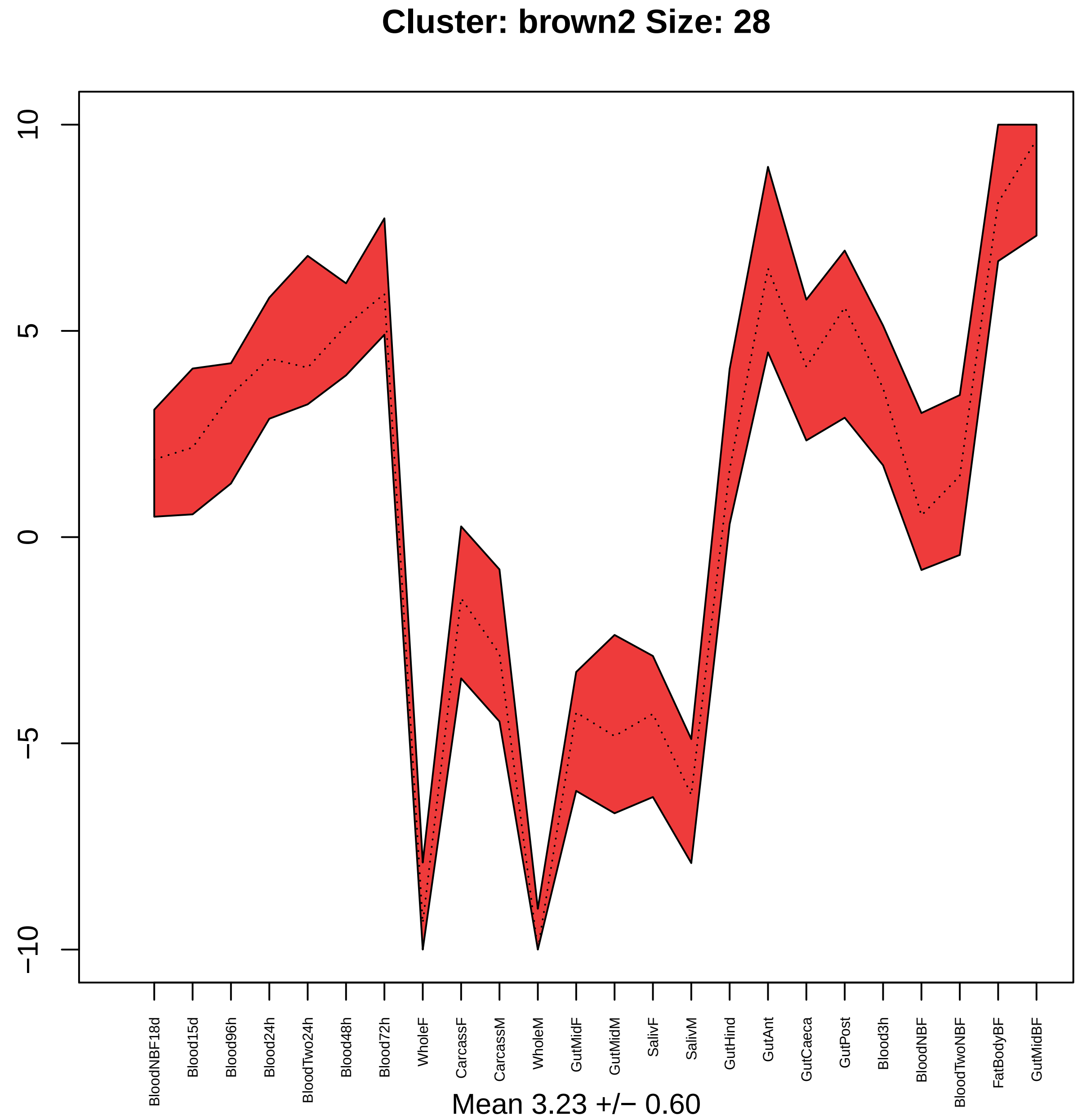
Cluster: brown2 Size: 28
10
5
0
−5
−10
BloodNBF18d
Blood15d
Blood96h
Blood24h
BloodTwo24h
Blood48h
Blood72h
WholeF
CarcassF
CarcassM
WholeM
GutMidF
GutMidM
SalivF
SalivM
GutHind
GutAnt
GutCaeca
GutPost
Blood3h
BloodNBF
BloodTwoNBF
FatBodyBF
GutMidBF
Mean 3.23 +/− 0.60

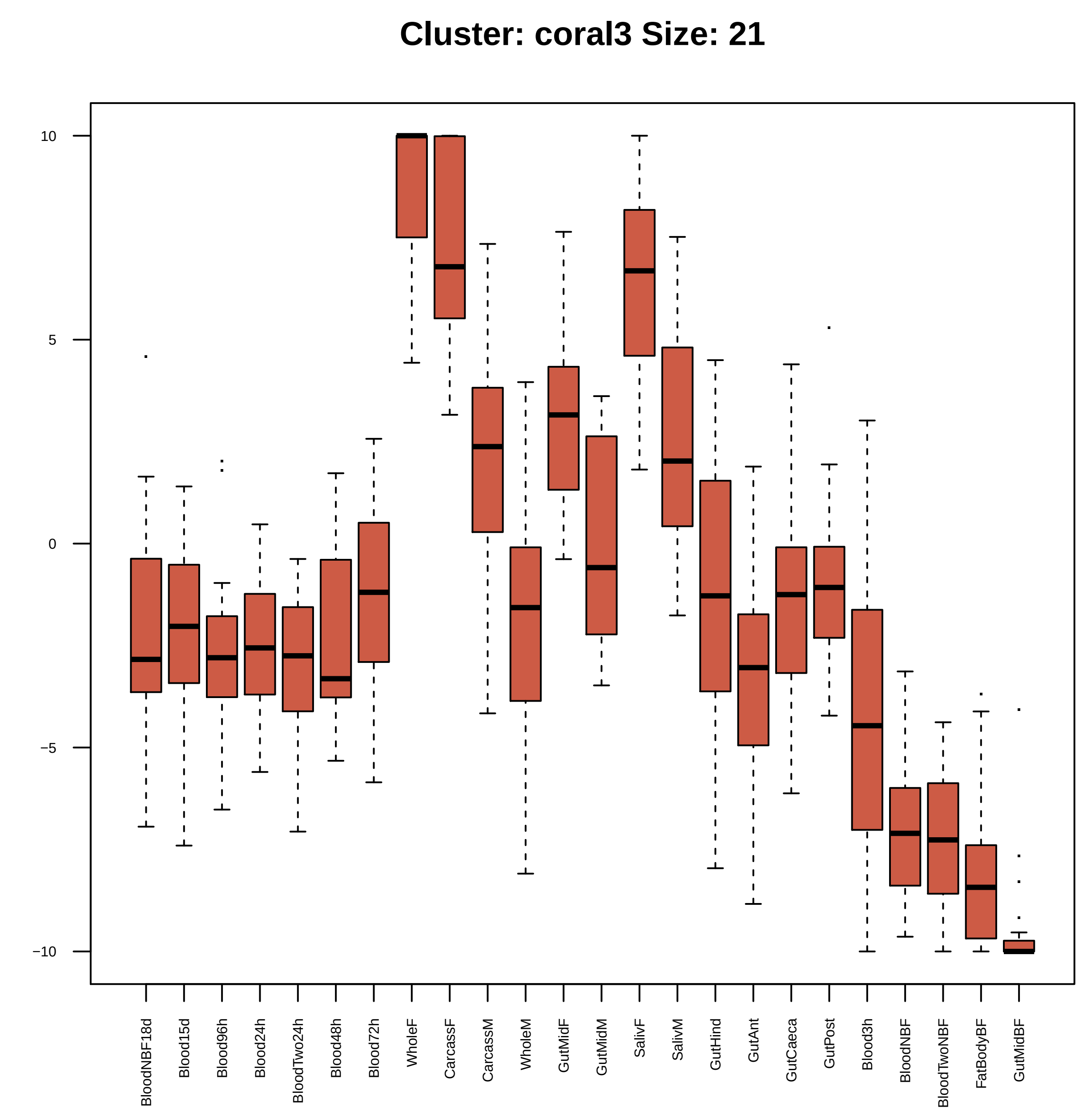

Cluster: coral3 Size: 21
10
5
0
−5
−10
BloodNBF18d
Blood15d
Blood96h
Blood24h
BloodTwo24h
Blood48h
Blood72h
WholeF
CarcassF
CarcassM
WholeM
GutMidF
GutMidM
SalivF
SalivM
GutHind
GutAnt
GutCaeca
GutPost
Blood3h
BloodNBF
BloodTwoNBF
FatBodyBF
GutMidBF

| | GO.ID | BPCluster: coral3 Size: 21 | Annotated | Significant | Expected | Rank in ClassicF | Weight01F | ClassicF |
|---|---|---|---|---|---|---|---|---|
| 1 | GO:0022008 | neurogenesis | 706 | 8 | 1.88 | 2 | 5.5e−06 | 0.00021 |
| 2 | GO:0001731 | formation of translation preinitiation c... | 13 | 2 | 0.03 | 4 | 0.00051 | 0.00051 |
| 3 | GO:0031122 | cytoplasmic microtubule organization | 16 | 2 | 0.04 | 8 | 0.00079 | 0.00079 |
| 4 | GO:0006457 | protein folding | 72 | 3 | 0.19 | 9 | 0.00084 | 0.00084 |
| 5 | GO:0006446 | regulation of translational initiation | 19 | 2 | 0.05 | 11 | 0.00111 | 0.00111 |
| 6 | GO:0044267 | cellular protein metabolic process | 1027 | 6 | 2.74 | 43 | 0.00266 | 0.04328 |
| 7 | GO:0007052 | mitotic spindle organization | 114 | 3 | 0.30 | 14 | 0.00315 | 0.00315 |
| 8 | GO:0006364 | rRNA processing | 34 | 2 | 0.09 | 16 | 0.00357 | 0.00357 |
| 12 | GO:0042254 | ribosome biogenesis | 64 | 3 | 0.17 | 6 | 0.06872 | 0.00059 |

| | GO.ID | MFCluster: coral3 Size: 21 | Annotated | Significant | Expected | Rank in ClassicF | Weight01F | ClassicF |
|---|---|---|---|---|---|---|---|---|
| *1* | GO:0051082 | unfolded protein binding | 41 | 3 | 0.10 | 1 | 0.00013 | 0.00013 |
| *2* | GO:0003743 | translation initiation factor activity | 35 | 2 | 0.09 | 2 | 0.00334 | 0.00334 |
| *3* | GO:0000166 | nucleotide binding | 998 | 7 | 2.49 | 4 | 0.01471 | 0.00755 |
| *6* | GO:0003723 | RNA binding | 307 | 4 | 0.77 | 3 | 0.04756 | 0.00614 |
| *17* | GO:0008135 | translation factor activity, RNA binding | 54 | 2 | 0.13 | 6 | 1.00000 | 0.00780 |
| *22* | GO:1901265 | nucleoside phosphate binding | 998 | 7 | 2.49 | 5 | 1.00000 | 0.00755 |

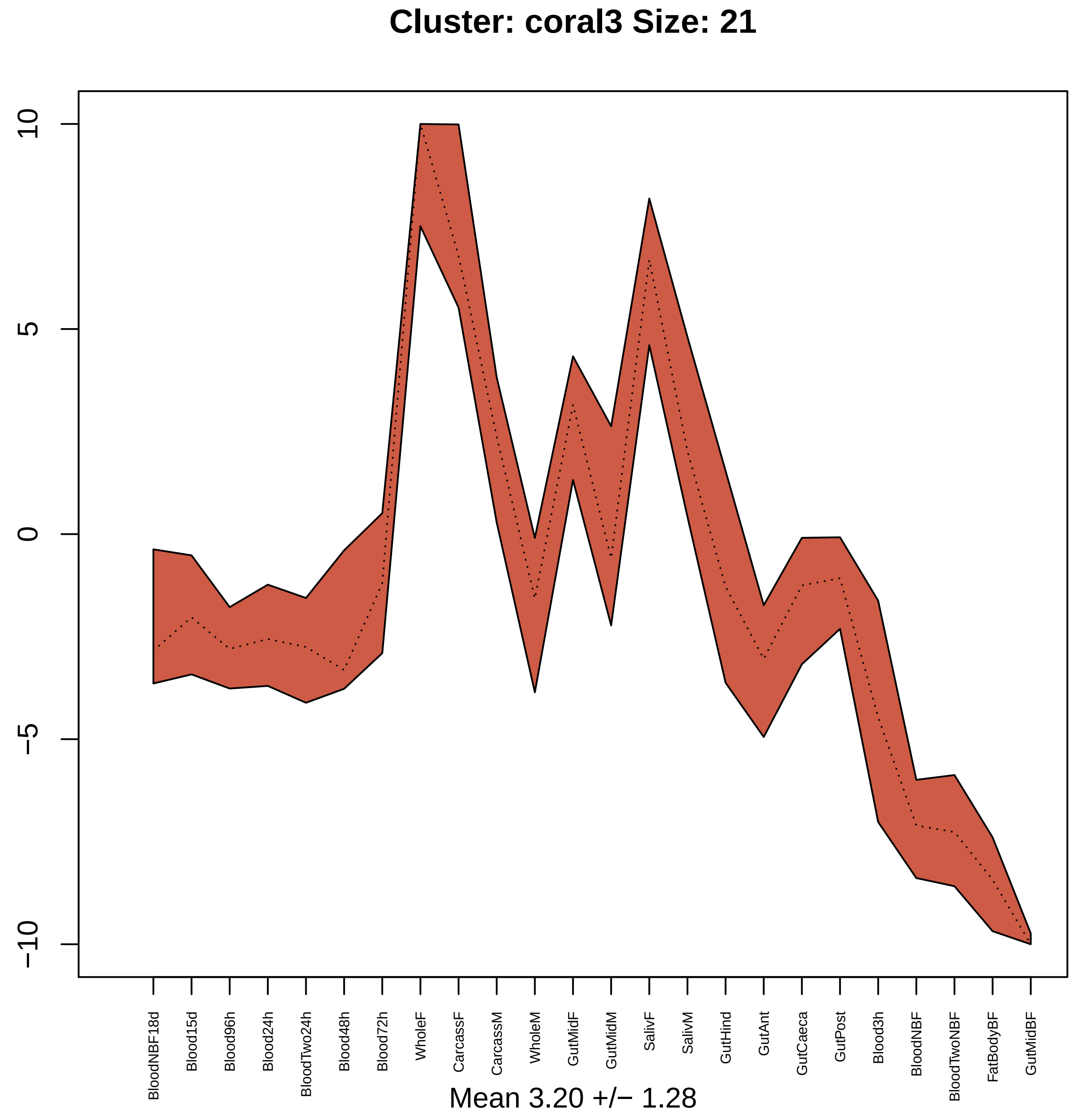
Cluster: coral3 Size: 21
10
5
0
−5
−10
BloodNBF18d
Blood15d
Blood96h
Blood24h
BloodTwo24h
Blood48h
Blood72h
WholeF
CarcassF
CarcassM
WholeM
GutMidF
GutMidM
SalivF
SalivM
GutHind
GutAnt
GutCaeca
GutPost
Blood3h
BloodNBF
BloodTwoNBF
FatBodyBF
GutMidBF
Mean 3.20 +/− 1.28

# Cluster: pink Size: 232

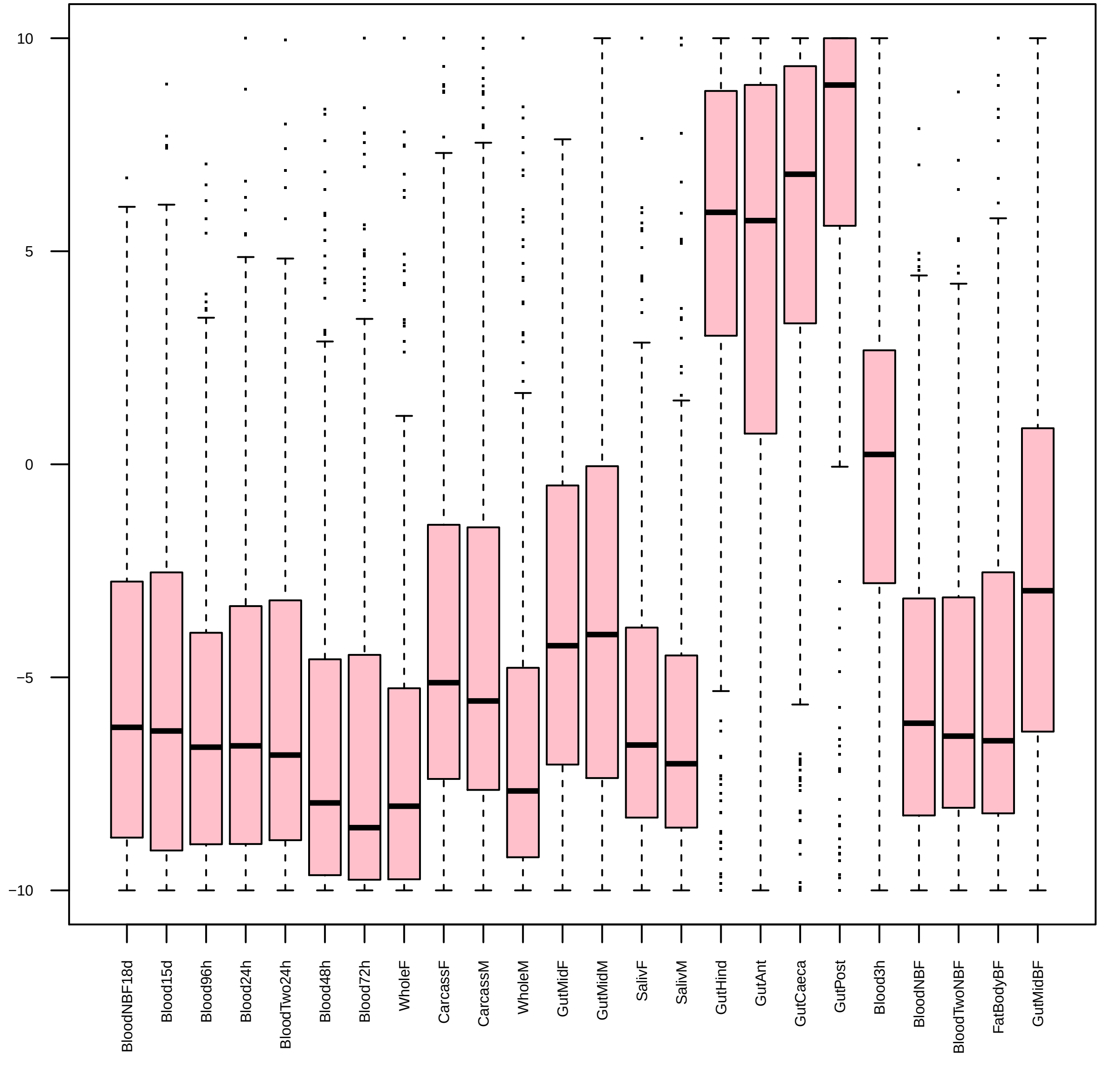

| | GO.ID | BPCluster: pink Size: 232 | Annotated | Significant | Expected | Rank in ClassicF | Weight01F | ClassicF |
|---|---|---|---|---|---|---|---|---|
| *3* | GO:0015991 | ATP hydrolysis coupled proton transport | 19 | 4 | 0.46 | 3 | 0.00096 | 0.00096 |
| *4* | GO:0044270 | cellular nitrogen compound catabolic pro... | 304 | 9 | 7.34 | 195 | 0.00202 | 0.31293 |
| *5* | GO:0046700 | heterocycle catabolic process | 305 | 9 | 7.37 | 197 | 0.00264 | 0.31632 |
| *6* | GO:1901361 | organic cyclic compound catabolic proces... | 308 | 9 | 7.44 | 207 | 0.00518 | 0.32652 |
| *22* | GO:0032787 | monocarboxylic acid metabolic process | 88 | 7 | 2.13 | 10 | 0.09083 | 0.00517 |

| | GO.ID | MFCluster: pink Size: 232 | Annotated | Significant | Expected | Rank in ClassicF | Weight01F | ClassicF |
|---|---|---|---|---|---|---|---|---|
| *3* | GO:0008236 | serine−type peptidase activity | 278 | 24 | 6.40 | 2 | 0.0019 | 1.7e−08 |
| *4* | GO:0032934 | sterol binding | 12 | 3 | 0.28 | 10 | 0.0023 | 0.0023 |
| *5* | GO:0003824 | catalytic activity | 3160 | 118 | 72.76 | 1 | 0.0025 | 6.4e−13 |
| *6* | GO:0016787 | hydrolase activity | 1445 | 56 | 33.27 | 8 | 0.0033 | 2.0e−05 |
| *13* | GO:0016627 | oxidoreductase activity, acting on the C... | 30 | 4 | 0.69 | 13 | 0.0503 | 0.0047 |

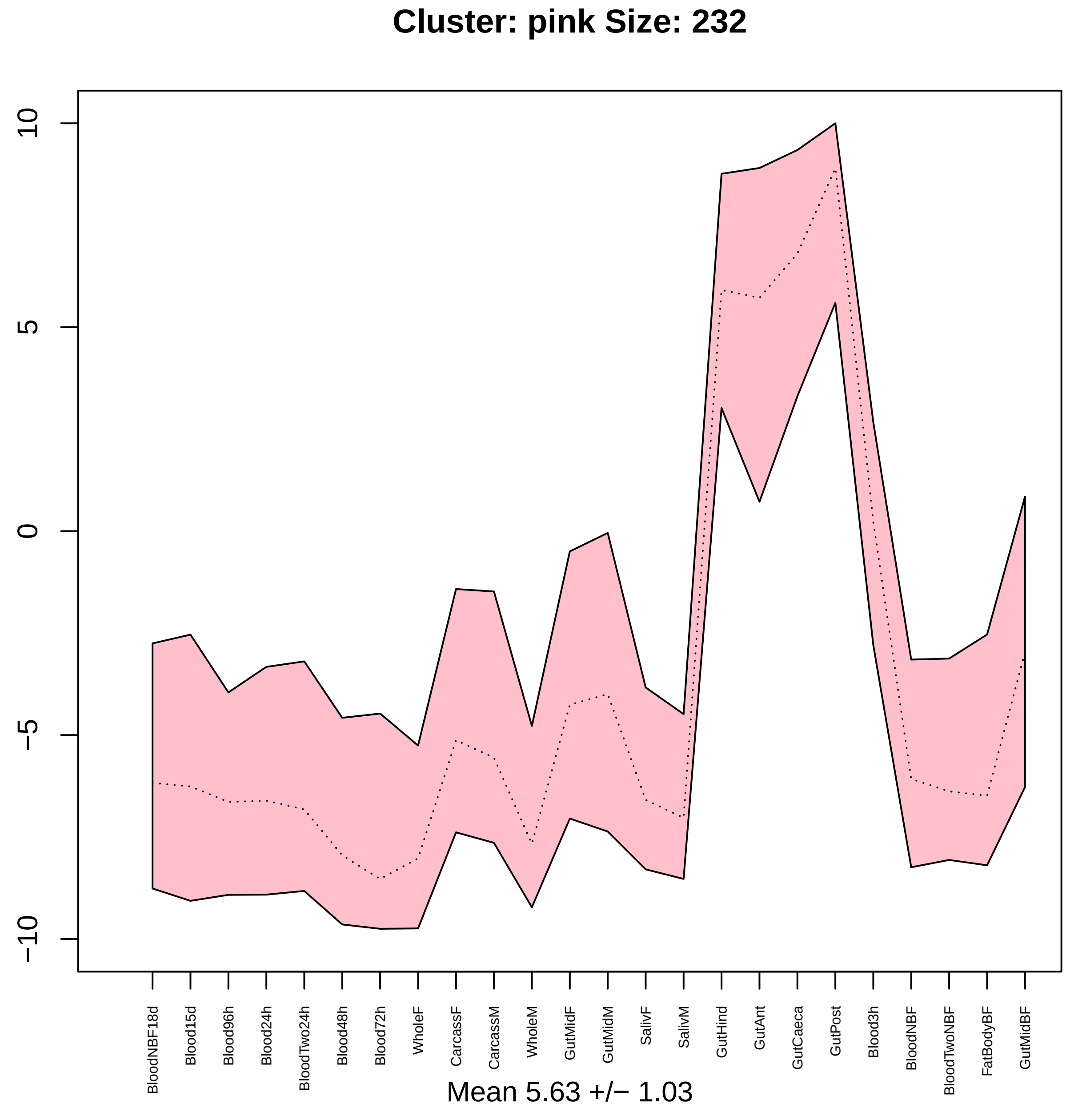

Cluster: pink Size: 232
10
5
0
−5
−10
BloodNBF18d
Blood15d
Blood96h
Blood24h
BloodTwo24h
Blood48h
Blood72h
WholeF
CarcassF
CarcassM
WholeM
GutMidF
GutMidM
SalivF
SalivM
GutHind
GutAnt
GutCaeca
GutPost
Blood3h
BloodNBF
BloodTwoNBF
FatBodyBF
GutMidBF
Mean 5.63 +/− 1.03

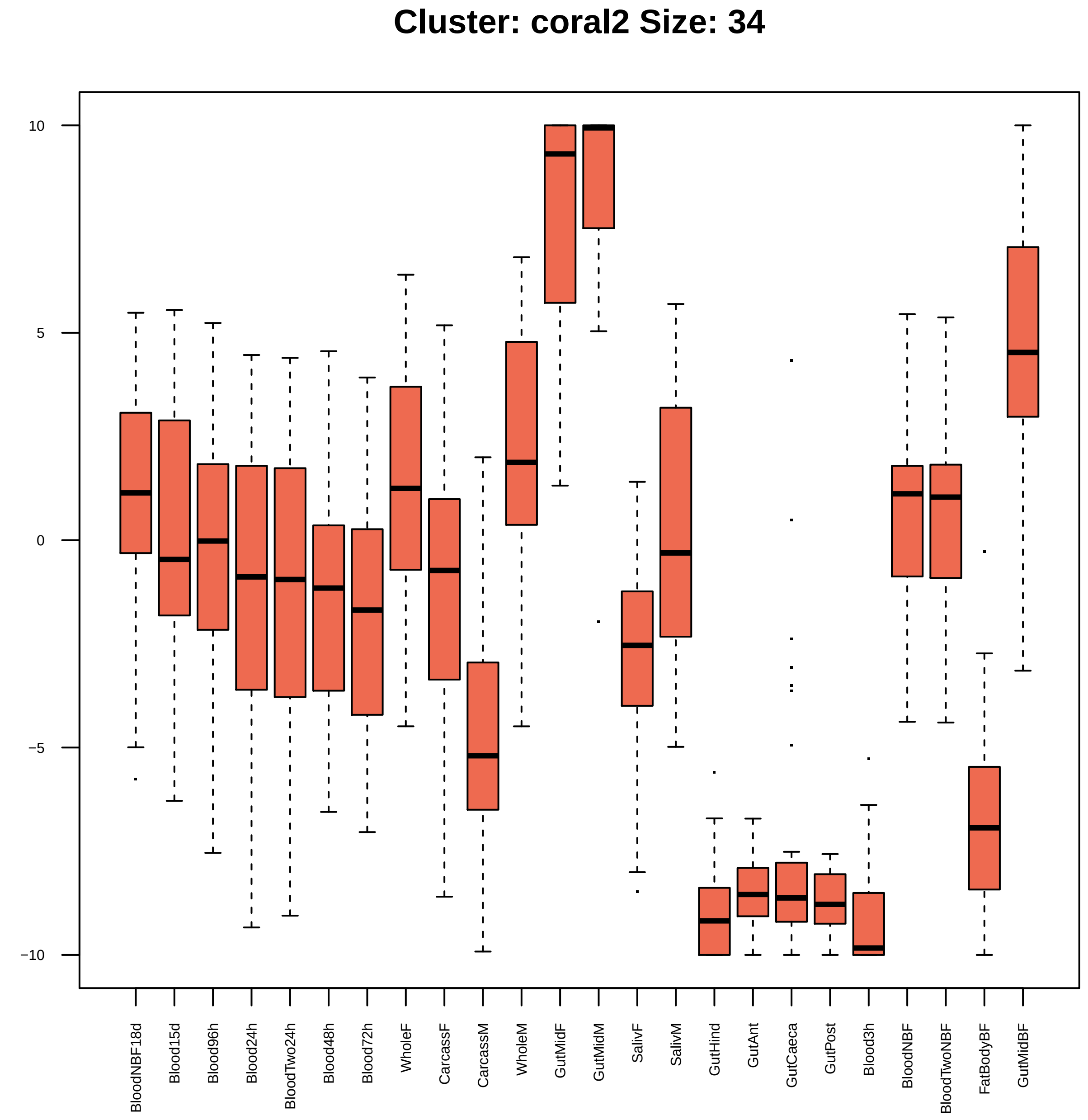

Cluster: coral2 Size: 34
10
5
0
−5
−10
BloodNBF18d
Blood15d
Blood96h
Blood24h
BloodTwo24h
Blood48h
Blood72h
WholeF
CarcassF
CarcassM
WholeM
GutMidF
GutMidM
SalivF
SalivM
GutHind
GutAnt
GutCaeca
GutPost
Blood3h
BloodNBF
BloodTwoNBF
FatBodyBF
GutMidBF

| | GO.ID | BPCluster: coral2 Size: 34 | Annotated | Significant | Expected | Rank in ClassicF | Weight01F | ClassicF |
|---|---|---|---|---|---|---|---|---|
| *3* | GO:0006508 | proteolysis | 594 | 8 | 2.05 | 6 | 0.00053 | 0.00053 |
| *4* | GO:0005975 | carbohydrate metabolic process | 210 | 5 | 0.72 | 7 | 0.00061 | 0.00061 |
| *5* | GO:0045087 | innate immune response | 60 | 3 | 0.21 | 8 | 0.00107 | 0.00107 |
| *6* | GO:0042742 | defense response to bacterium | 61 | 3 | 0.21 | 9 | 0.00113 | 0.00113 |
| *15* | GO:1901564 | organonitrogen compound metabolic proces... | 1889 | 13 | 6.52 | 15 | 1.00000 | 0.00377 |
| *16* | GO:1901135 | carbohydrate derivative metabolic proces... | 344 | 5 | 1.19 | 18 | 1.00000 | 0.00546 |
| *22* | GO:0051707 | response to other organism | 92 | 3 | 0.32 | 13 | 1.00000 | 0.00367 |
| *23* | GO:0009617 | response to bacterium | 64 | 3 | 0.22 | 11 | 1.00000 | 0.00130 |
| *29* | GO:0006952 | defense response | 108 | 3 | 0.37 | 19 | 1.00000 | 0.00576 |

| | GO.ID | MFCluster: coral2 Size: 34 | Annotated | Significant | Expected | Rank in ClassicF | Weight01F | ClassicF |
|---|---|---|---|---|---|---|---|---|
| *8* | GO:0003824 | catalytic activity | 3160 | 14 | 8.33 | 10 | 0.145 | 0.0082 |
| *22* | GO:0140096 | catalytic activity, acting on a protein | 947 | 8 | 2.50 | 9 | 1.000 | 0.0017 |
| *26* | GO:0016787 | hydrolase activity | 1445 | 10 | 3.81 | 8 | 1.000 | 0.0016 |

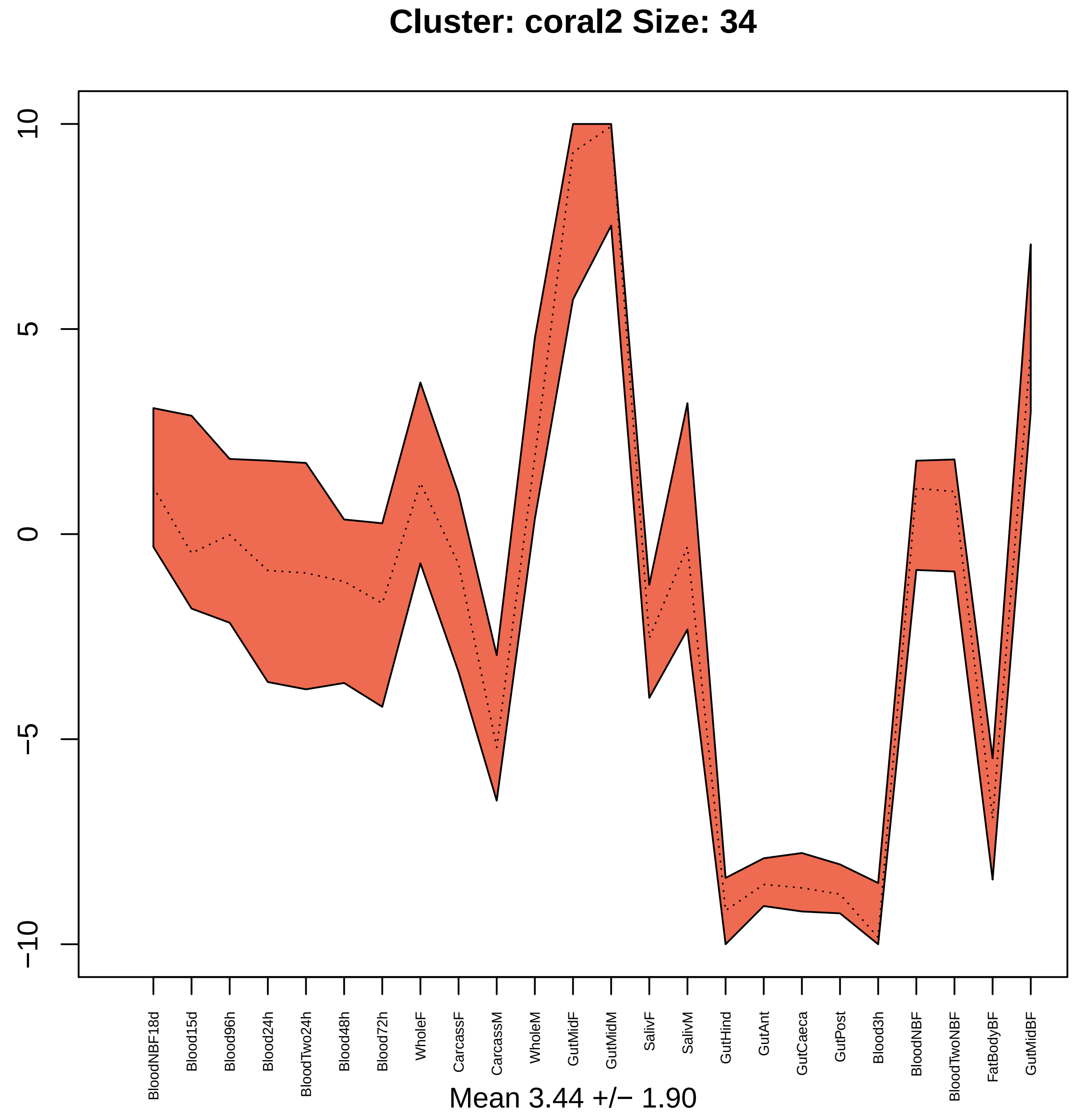

Cluster: coral2 Size: 34
10
5
0
−5
−10
BloodNBF18d
Blood15d
Blood96h
Blood24h
BloodTwo24h
Blood48h
Blood72h
WholeF
CarcassF
CarcassM
WholeM
GutMidF
GutMidM
SalivF
SalivM
GutHind
GutAnt
GutCaeca
GutPost
Blood3h
BloodNBF
BloodTwoNBF
FatBodyBF
GutMidBF
Mean 3.44 +/− 1.90

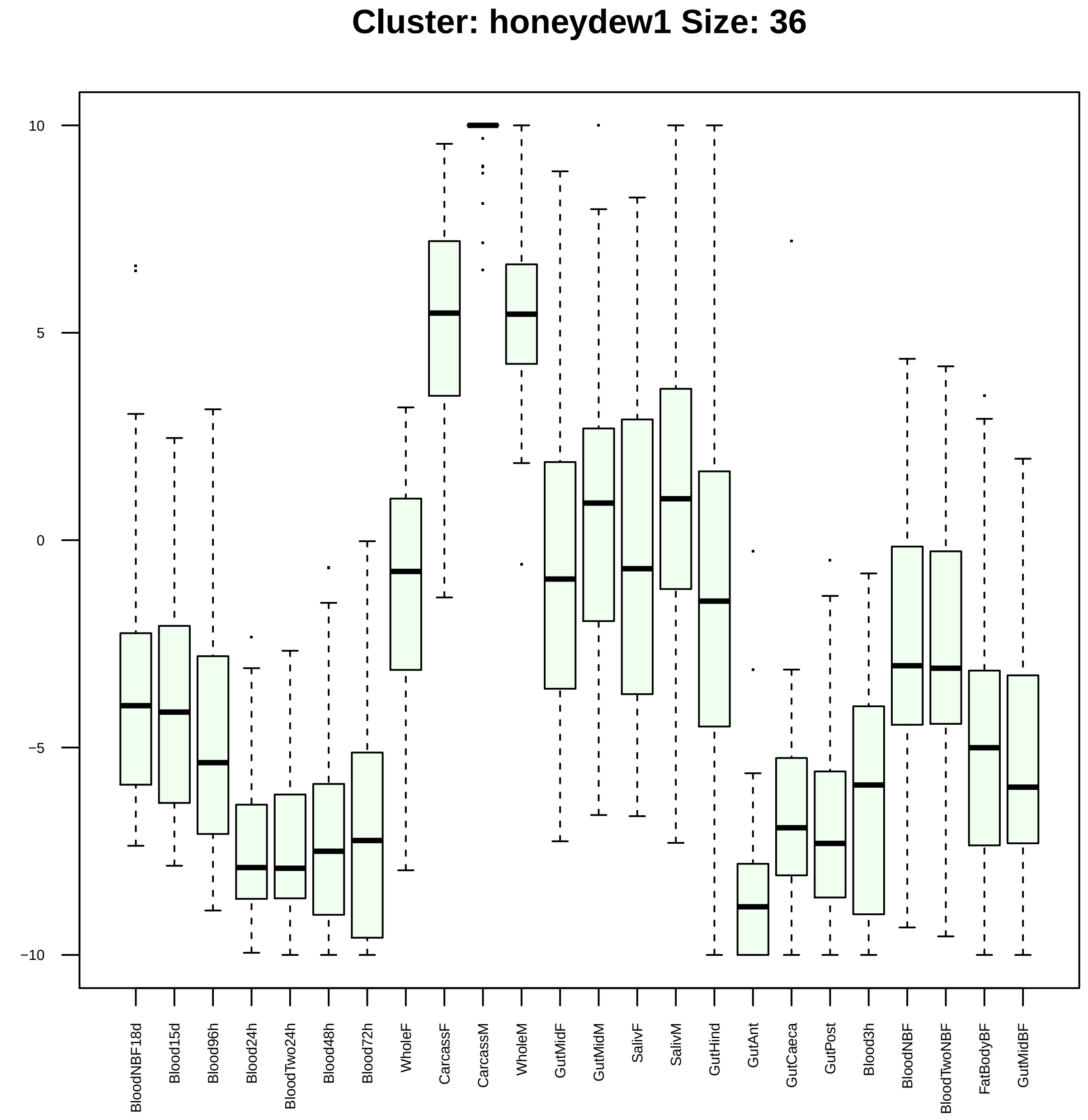

| | GO.ID | BPCluster: honeydew1 Size: 36 | Annotated | Significant | Expected | Rank in ClassicF | Weight01F | ClassicF |
|---|---|---|---|---|---|---|---|---|
| *1* | GO:0070997 | neuron death | 15 | 2 | 0.06 | 1 | 0.0036 | 0.0014 |
| *2* | GO:0048284 | organelle fusion | 33 | 2 | 0.12 | 3 | 0.0286 | 0.0067 |
| *3* | GO:0022411 | cellular component disassembly | 36 | 2 | 0.14 | 4 | 0.0322 | 0.0079 |
| *21* | GO:0010639 | negative regulation of organelle organiz... | 29 | 2 | 0.11 | 2 | 0.0632 | 0.0052 |

| | GO.ID | MFCluster: honeydew1 Size: 36 | Annotated | Significant | Expected | Rank in ClassicF | Weight01F | ClassicF |
|---|---|---|---|---|---|---|---|---|
| *1* | GO:0003779 | actin binding | 82 | 3 | 0.30 | 1 | 0.0031 | 0.0031 |
| *2* | GO:0015294 | solute:cation symporter activity | 37 | 2 | 0.13 | 2 | 0.0207 | 0.0077 |

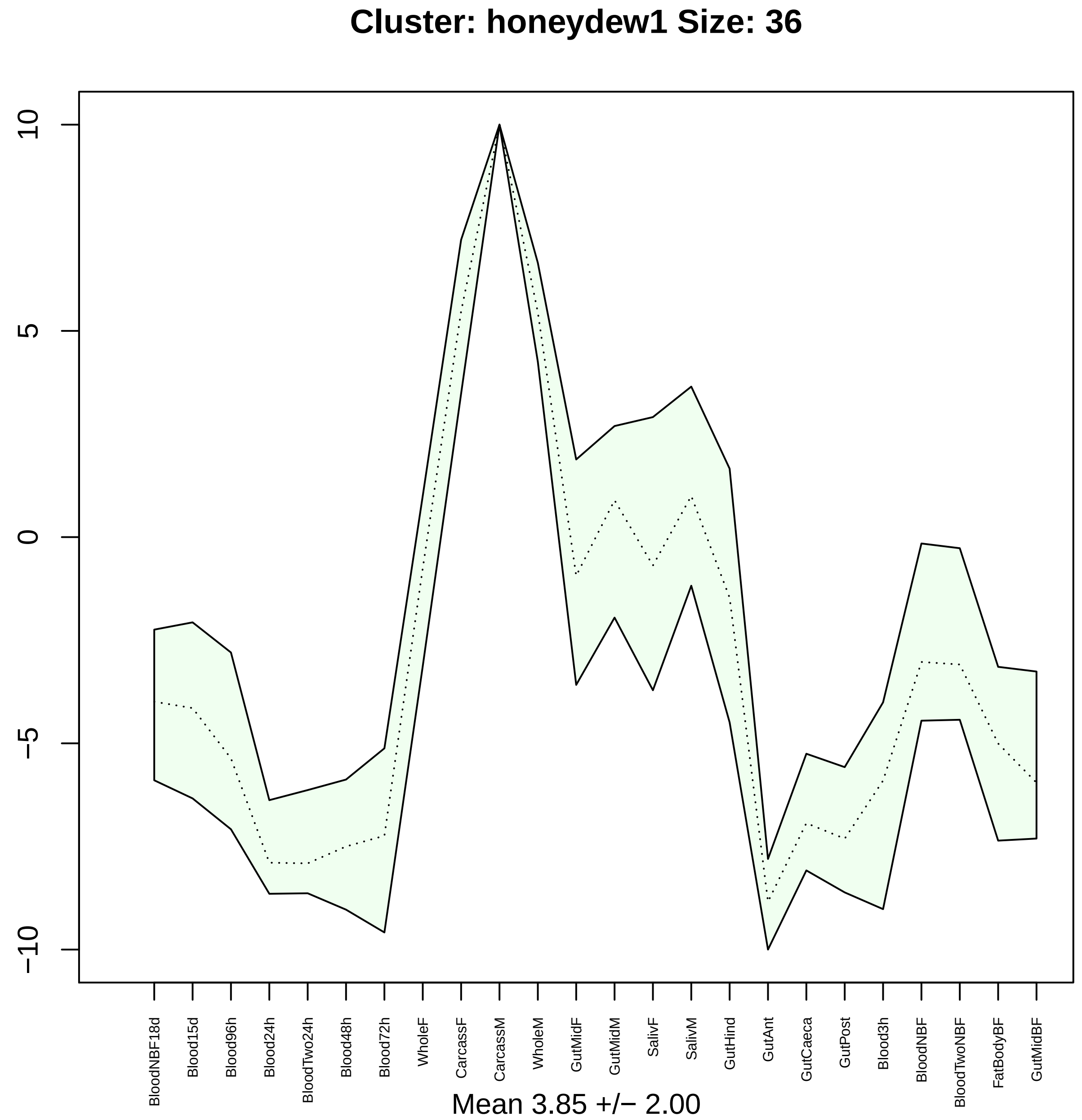
Cluster: honeydew1 Size: 36
10
5
0
−5
−10
BloodNBF18d
Blood15d
Blood96h
Blood24h
BloodTwo24h
Blood48h
Blood72h
WholeF
CarcassF
CarcassM
WholeM
GutMidF
GutMidM
SalivF
SalivM
GutHind
GutAnt
GutCaeca
GutPost
Blood3h
BloodNBF
BloodTwoNBF
FatBodyBF
GutMidBF
Mean 3.85 +/− 2.00

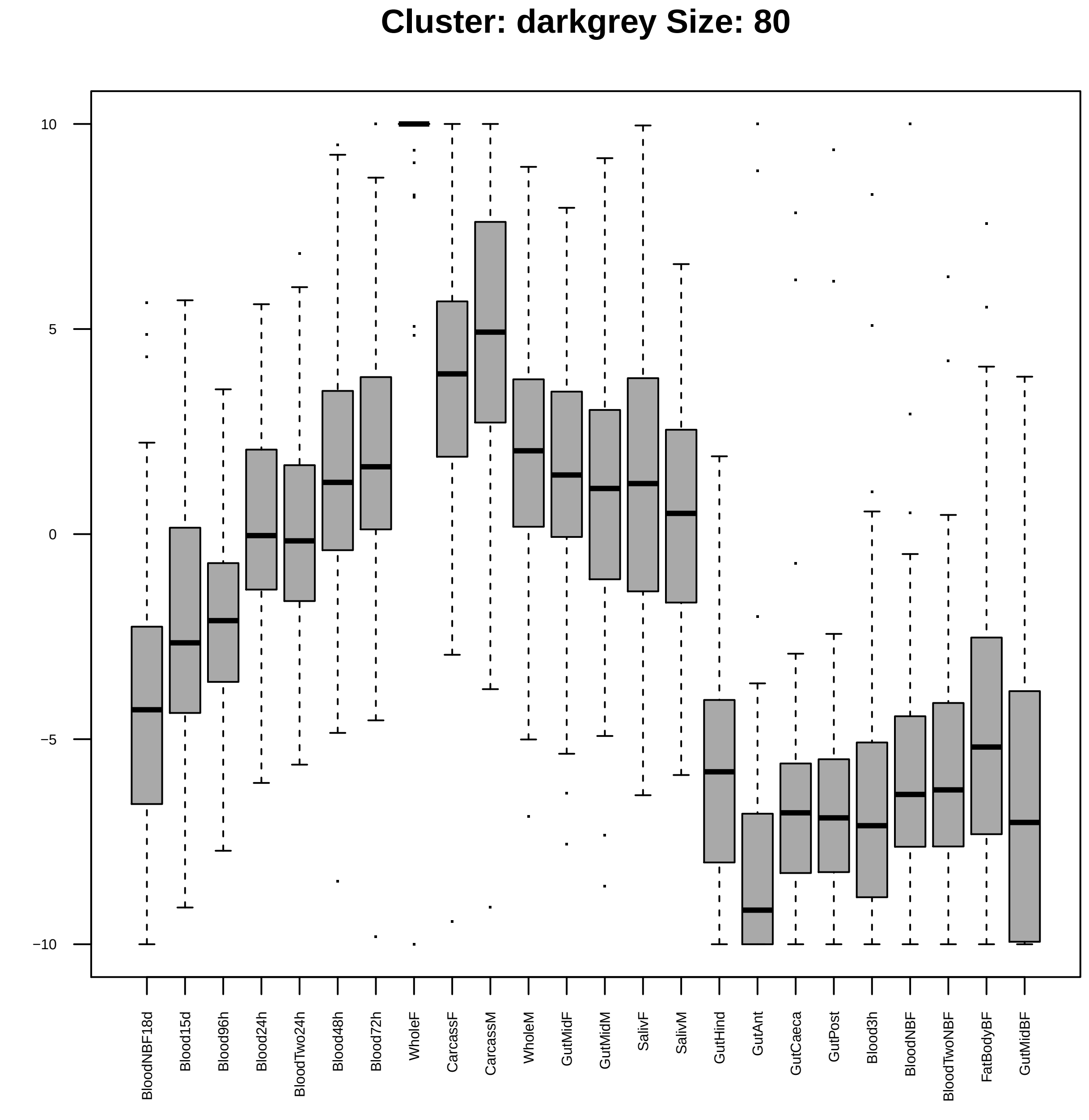

Cluster: darkgrey Size: 80
10
5
0
−5
−10
BloodNBF18d
Blood15d
Blood96h
Blood24h
BloodTwo24h
Blood48h
Blood72h
WholeF
CarcassF
CarcassM
WholeM
GutMidF
GutMidM
SalivF
SalivM
GutHind
GutAnt
GutCaeca
GutPost
Blood3h
BloodNBF
BloodTwoNBF
FatBodyBF
GutMidBF

| | GO.ID | BPCluster: darkgrey Size: 80 | Annotated | Significant | Expected | Rank in ClassicF | Weight01F | ClassicF |
|---|---|---|---|---|---|---|---|---|
| *2* | GO:2001251 | negative regulation of chromosome organi... | 14 | 3 | 0.11 | 46 | 0.00017 | 0.00017 |
| *3* | GO:0007052 | mitotic spindle organization | 114 | 6 | 0.93 | 51 | 0.00030 | 0.00030 |
| *4* | GO:0022008 | neurogenesis | 706 | 13 | 5.76 | 98 | 0.00050 | 0.00354 |
| *5* | GO:0000077 | DNA damage checkpoint | 53 | 4 | 0.43 | 66 | 0.00060 | 0.00086 |
| *6* | GO:0006261 | DNA−dependent DNA replication | 52 | 4 | 0.42 | 64 | 0.00080 | 0.00080 |
| *7* | GO:0007474 | imaginal disc−derived wing vein specific... | 26 | 3 | 0.21 | 75 | 0.00116 | 0.00116 |
| *8* | GO:0007093 | mitotic cell cycle checkpoint | 56 | 4 | 0.46 | 71 | 0.00212 | 0.00106 |
| *9* | GO:0008544 | epidermis development | 34 | 3 | 0.28 | 90 | 0.00220 | 0.00256 |
| *10* | GO:0022403 | cell cycle phase | 10 | 2 | 0.08 | 91 | 0.00281 | 0.00281 |
| *11* | GO:0030261 | chromosome condensation | 25 | 4 | 0.20 | 32 | 0.00318 | 4.4e−05 |
| *12* | GO:2000241 | regulation of reproductive process | 30 | 3 | 0.24 | 79 | 0.00331 | 0.00178 |
| *13* | GO:0007076 | mitotic chromosome condensation | 14 | 2 | 0.11 | 110 | 0.00557 | 0.00557 |
| *14* | GO:0051649 | establishment of localization in cell | 317 | 6 | 2.58 | 208 | 0.00601 | 0.04281 |
| *15* | GO:0000413 | protein peptidyl−prolyl isomerization | 15 | 2 | 0.12 | 113 | 0.00640 | 0.00640 |
| *16* | GO:1901991 | negative regulation of mitotic cell cycl... | 60 | 4 | 0.49 | 77 | 0.00683 | 0.00137 |
| *20* | GO:0006338 | chromatin remodeling | 37 | 3 | 0.30 | 96 | 0.01943 | 0.00327 |
| *23* | GO:0140013 | meiotic nuclear division | 68 | 4 | 0.55 | 88 | 0.02311 | 0.00218 |
| *28* | GO:0010389 | regulation of G2/M transition of mitotic... | 48 | 3 | 0.39 | 116 | 0.03122 | 0.00683 |

| | GO.ID | MFCluster: darkgrey Size: 80 | Annotated | Significant | Expected | Rank in ClassicF | Weight01F | ClassicF |
|---|---|---|---|---|---|---|---|---|
| *1* | GO:0003677 | DNA binding | 523 | 13 | 4.43 | 4 | 0.00053 | 0.00034 |
| *2* | GO:0008270 | zinc ion binding | 522 | 12 | 4.42 | 7 | 0.00120 | 0.00120 |
| *3* | GO:0008094 | DNA−dependent ATPase activity | 33 | 4 | 0.28 | 3 | 0.00415 | 0.00016 |
| *4* | GO:0005524 | ATP binding | 592 | 11 | 5.01 | 15 | 0.00993 | 0.00993 |
| *8* | GO:0005515 | protein binding | 2143 | 28 | 18.13 | 11 | 0.02336 | 0.00534 |
| *10* | GO:0140097 | catalytic activity, acting on DNA | 81 | 5 | 0.69 | 5 | 0.02899 | 0.00057 |
| *13* | GO:0008026 | ATP−dependent helicase activity | 76 | 4 | 0.64 | 9 | 0.07458 | 0.00378 |
| *18* | GO:0016853 | isomerase activity | 84 | 4 | 0.71 | 12 | 0.09415 | 0.00541 |

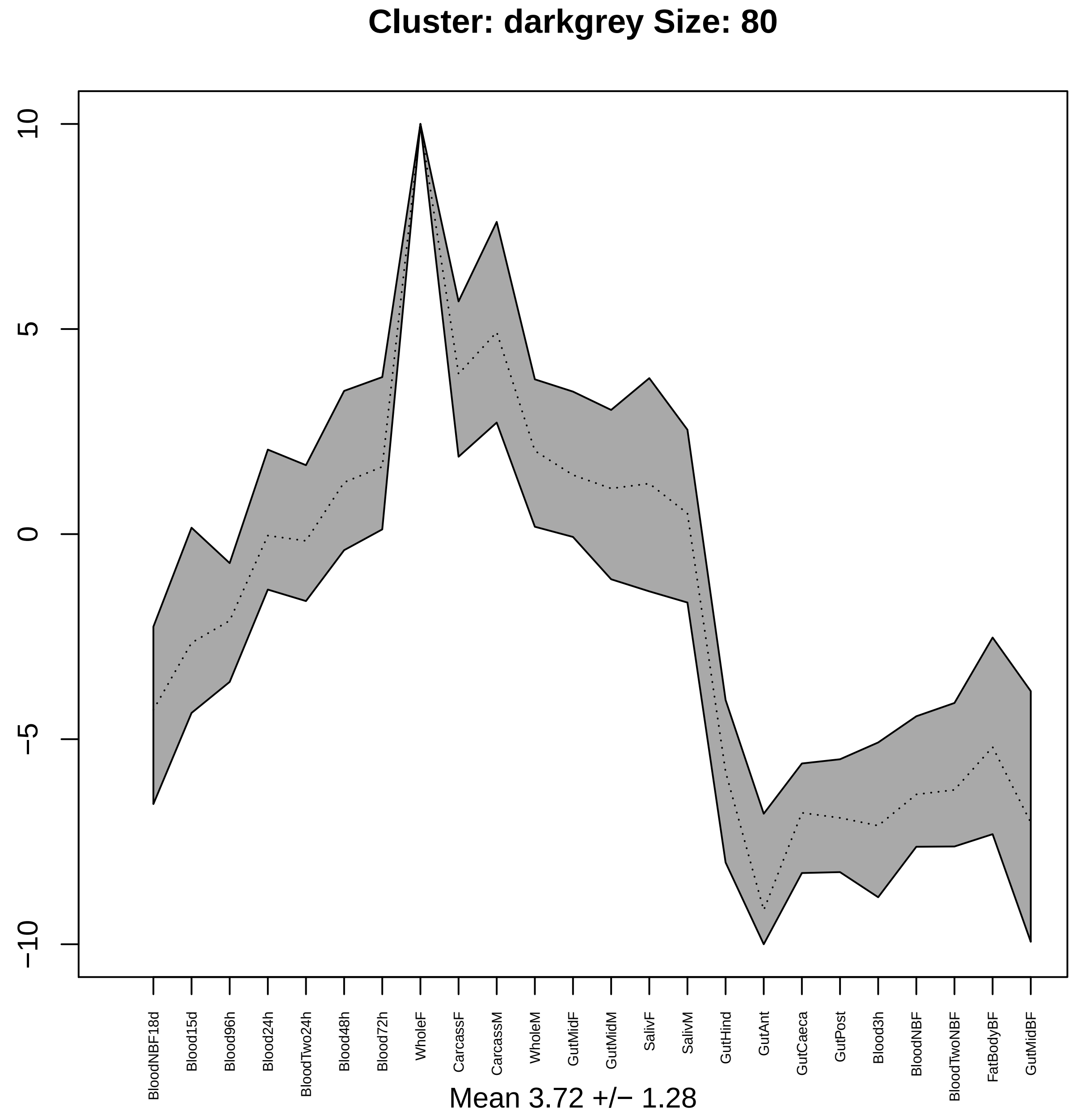

Cluster: darkgrey Size: 80
10
5
0
−5
−10
BloodNBF18d
Blood15d
Blood96h
Blood24h
BloodTwo24h
Blood48h
Blood72h
WholeF
CarcassF
CarcassM
WholeM
GutMidF
GutMidM
SalivF
SalivM
GutHind
GutAnt
GutCaeca
GutPost
Blood3h
BloodNBF
BloodTwoNBF
FatBodyBF
GutMidBF
Mean 3.72 +/− 1.28

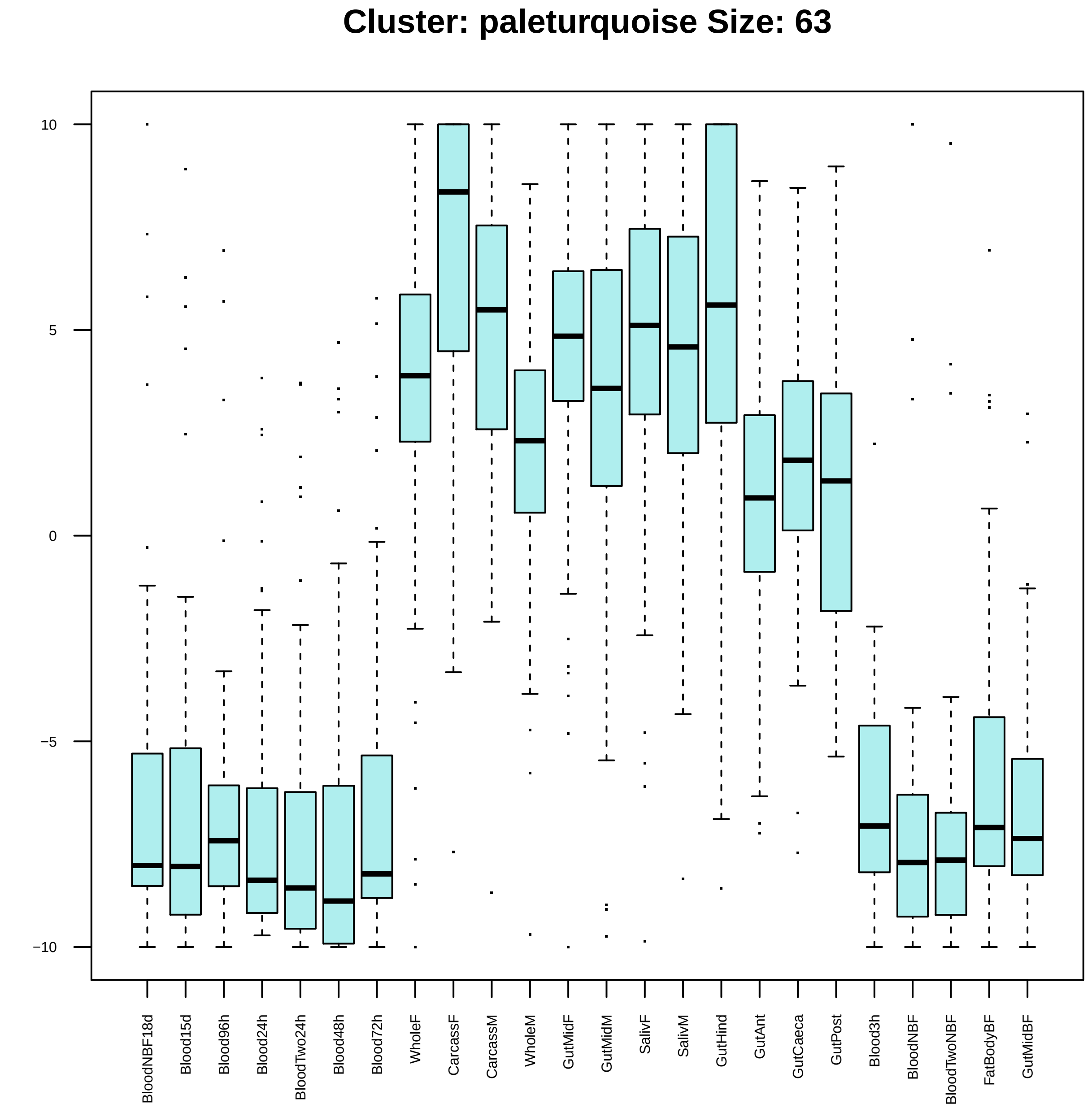

Cluster: paleturquoise Size: 63
10
5
0
−5
−10
BloodNBF18d
Blood15d
Blood96h
Blood24h
BloodTwo24h
Blood48h
Blood72h
WholeF
CarcassF
CarcassM
WholeM
GutMidF
GutMidM
SalivF
SalivM
GutHind
GutAnt
GutCaeca
GutPost
Blood3h
BloodNBF
BloodTwoNBF
FatBodyBF
GutMidBF

| | GO.ID | BPCluster: paleturquoise Size: 63 | Annotated | Significant | Expected | Rank in ClassicF | Weight01F | ClassicF |
|---|---|---|---|---|---|---|---|---|
| *3* | GO:0006413 | translational initiation | 39 | 3 | 0.18 | 16 | 0.0033 | 0.00077 |
| *4* | GO:0009058 | biosynthetic process | 1357 | 14 | 6.38 | 21 | 0.0066 | 0.00166 |
| *13* | GO:0140014 | mitotic nuclear division | 105 | 5 | 0.49 | 10 | 0.0758 | 0.00011 |

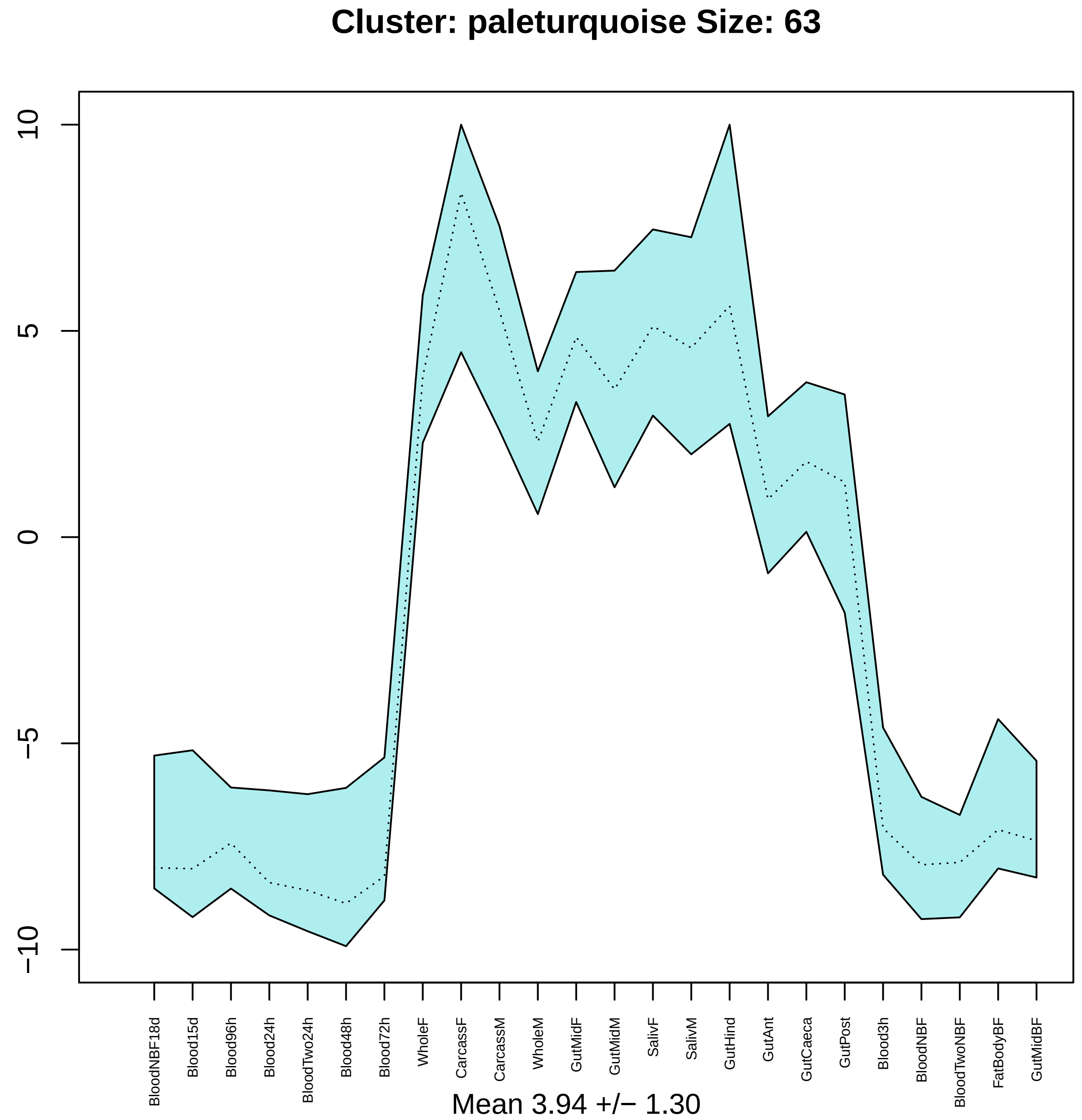

Cluster: paleturquoise Size: 63
10
5
0
−5
−10
BloodNBF18d
Blood15d
Blood96h
Blood24h
BloodTwo24h
Blood48h
Blood72h
WholeF
CarcassF
CarcassM
WholeM
GutMidF
GutMidM
SalivF
SalivM
GutHind
GutAnt
GutCaeca
GutPost
Blood3h
BloodNBF
BloodTwoNBF
FatBodyBF
GutMidBF
Mean 3.94 +/− 1.30

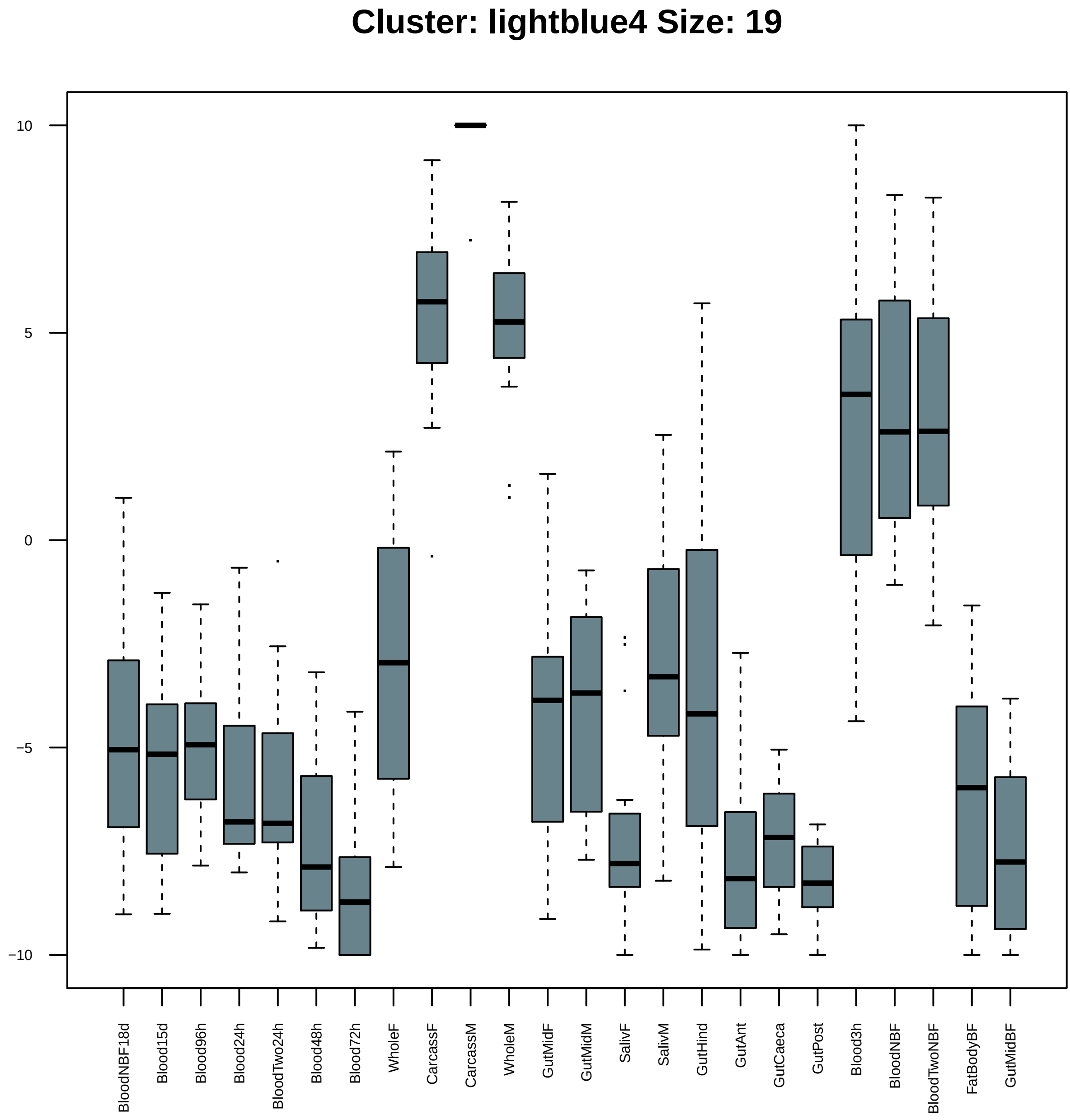

Cluster: lightblue4 Size: 19
10
5
0
−5
−10
BloodNBF18d
Blood15d
Blood96h
Blood24h
BloodTwo24h
Blood48h
Blood72h
WholeF
CarcassF
CarcassM
WholeM
GutMidF
GutMidM
SalivF
SalivM
GutHind
GutAnt
GutCaeca
GutPost
Blood3h
BloodNBF
BloodTwoNBF
FatBodyBF
GutMidBF

| | GO.ID | BPCluster: lightblue4 Size: 19 | Annotated | Significant | Expected | Rank in ClassicF | Weight01F | ClassicF |
|---|---|---|---|---|---|---|---|---|
| *1* | GO:0003012 | muscle system process | 14 | 2 | 0.02 | 1 | 0.0063 | 0.00024 |
| *29* | GO:0070925 | organelle assembly | 89 | 2 | 0.15 | 2 | 1.0000 | 0.00976 |

| | GO.ID | MFCluster: lightblue4 Size: 19 | Annotated | Significant | Expected | Rank in ClassicF | Weight01F | ClassicF |
|---|---|---|---|---|---|---|---|---|
| 1 | GO:0003774 | motor activity | 60 | 3 | 0.11 | 1 | 0.00015 | 0.00015 |
| 2 | GO:0005509 | calcium ion binding | 173 | 4 | 0.31 | 2 | 0.00019 | 0.00019 |

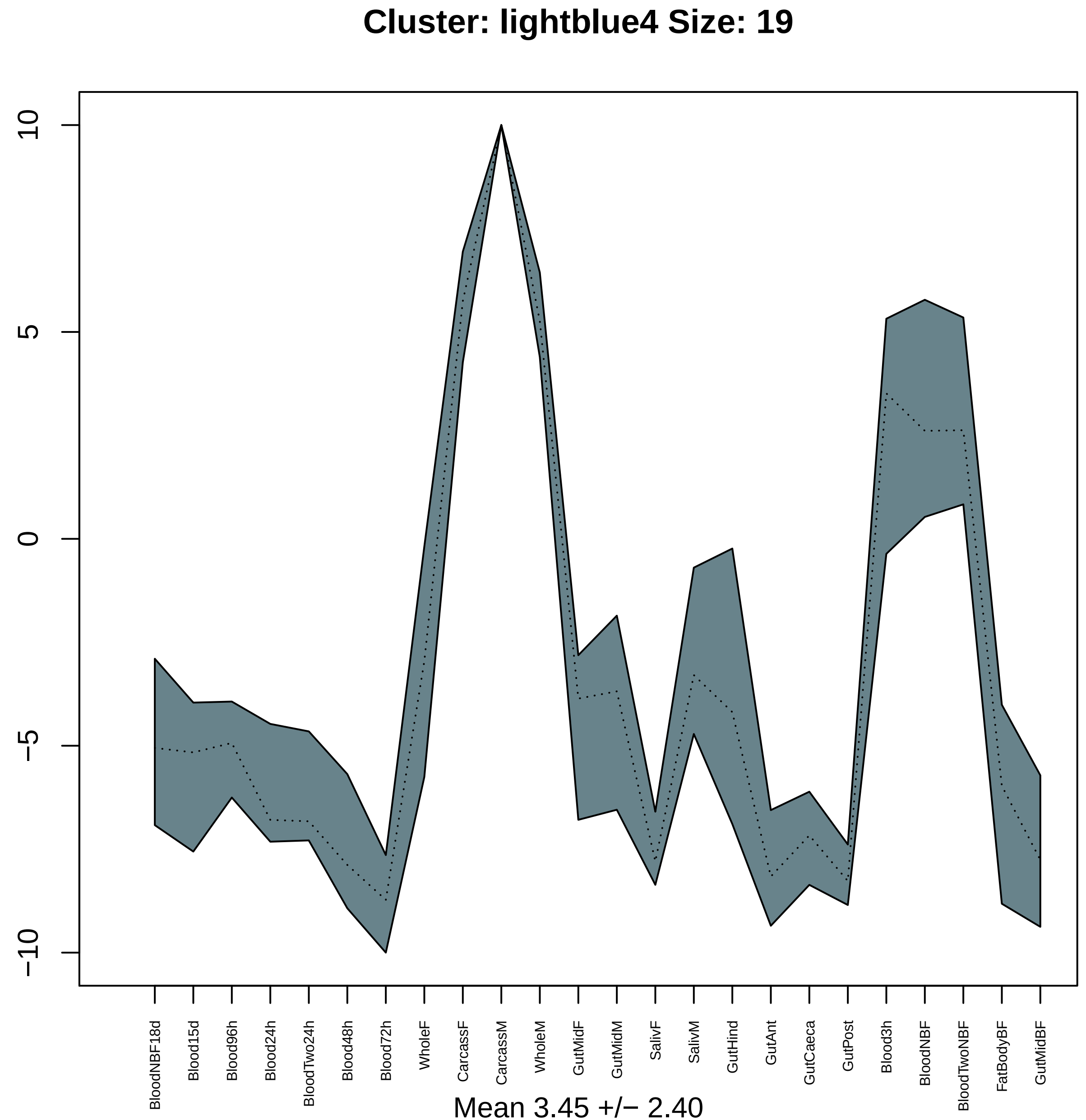
Cluster: lightblue4 Size: 19
10
5
0
−5
−10
BloodNBF18d
Blood15d
Blood96h
Blood24h
BloodTwo24h
Blood48h
Blood72h
WholeF
CarcassF
CarcassM
WholeM
GutMidF
GutMidM
SalivF
SalivM
GutHind
GutAnt
GutCaeca
GutPost
Blood3h
BloodNBF
BloodTwoNBF
FatBodyBF
GutMidBF
Mean 3.45 +/− 2.40

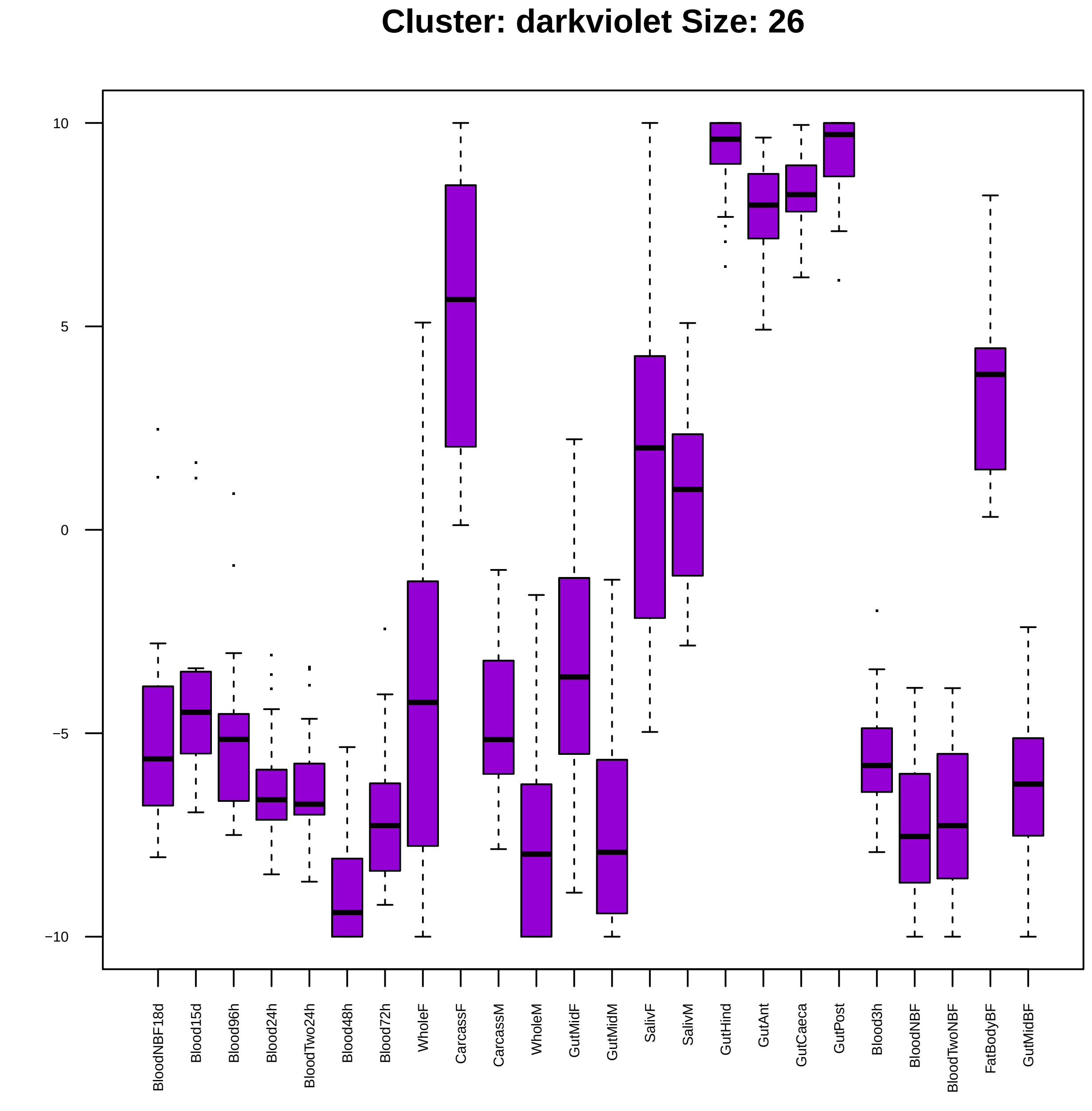

Cluster: darkviolet Size: 26
10
5
0
−5
−10
BloodNBF18d
Blood15d
Blood96h
Blood24h
BloodTwo24h
Blood48h
Blood72h
WholeF
CarcassF
CarcassM
WholeM
GutMidF
GutMidM
SalivF
SalivM
GutHind
GutAnt
GutCaeca
GutPost
Blood3h
BloodNBF
BloodTwoNBF
FatBodyBF
GutMidBF

| | GO.ID | BPCluster: darkviolet Size: 26 | Annotated | Significant | Expected | Rank in ClassicF | Weight01F | ClassicF |
|---|---|---|---|---|---|---|---|---|
| *1* | GO:0006412 | translation | 289 | 25 | 1.13 | 1 | < 1e−30 | < 1e−30 |
| *4* | GO:0006414 | translational elongation | 22 | 2 | 0.09 | 50 | 0.0032 | 0.0032 |
| *29* | GO:0071840 | cellular component organization or bioge... | 1209 | 11 | 4.74 | 51 | 1.0000 | 0.0035 |

| | GO.ID | MFCluster: darkviolet Size: 26 | Annotated | Significant | Expected | Rank in ClassicF | Weight01F | ClassicF |
|---|---|---|---|---|---|---|---|---|
| *1* | GO:0003735 | structural constituent of ribosome | 146 | 24 | 0.51 | 1 | < 1e−30 | < 1e−30 |
| *11* | GO:0003723 | RNA binding | 307 | 5 | 1.06 | 4 | 1.000 | 0.0036 |
| *14* | GO:0005198 | structural molecule activity | 318 | 24 | 1.10 | 2 | 1.000 | < 1e−30 |

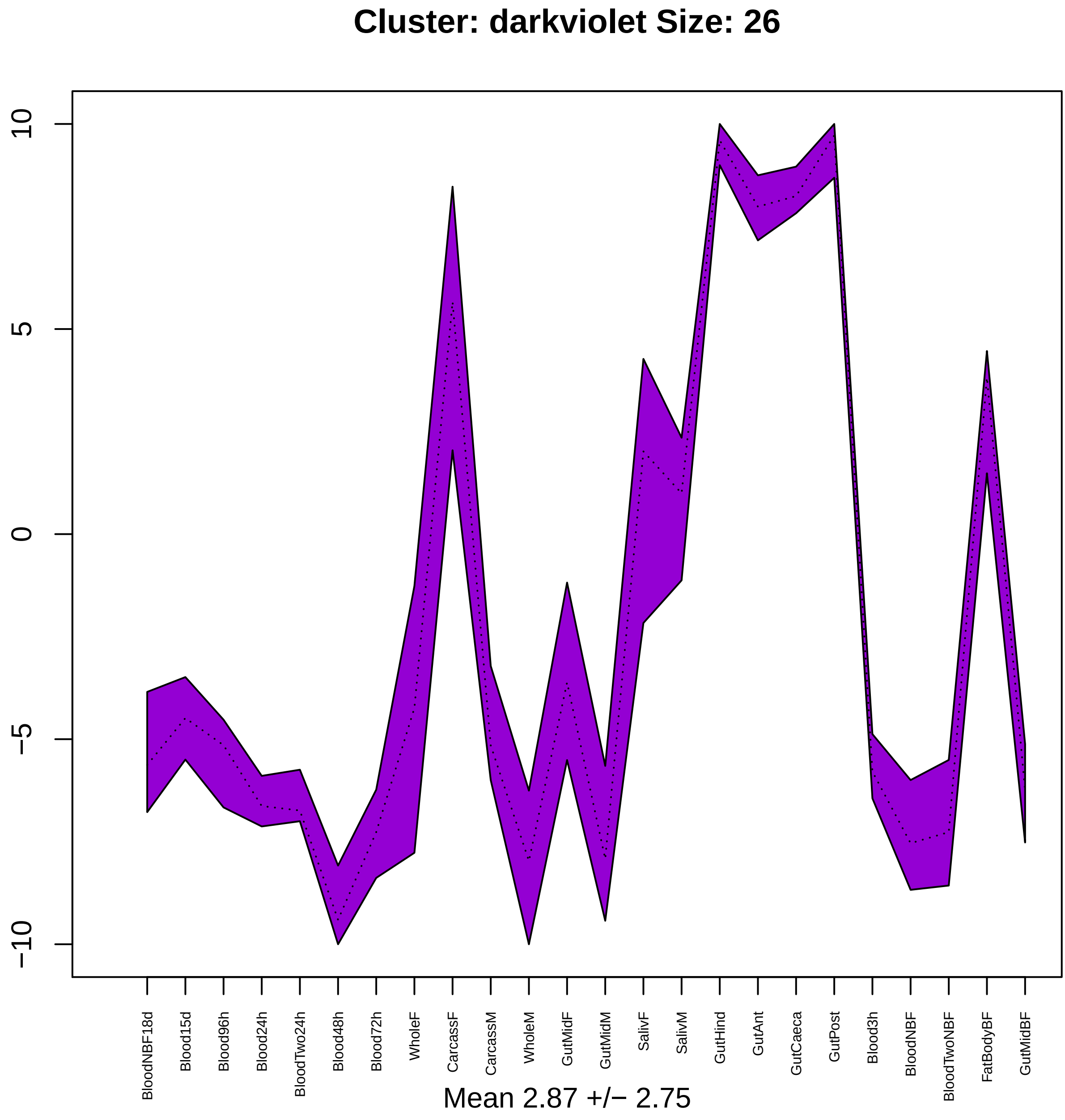
Cluster: darkviolet Size: 26
10
5
0
−5
−10
BloodNBF18d
Blood15d
Blood96h
Blood24h
BloodTwo24h
Blood48h
Blood72h
WholeF
CarcassF
CarcassM
WholeM
GutMidF
GutMidM
SalivF
SalivM
GutHind
GutAnt
GutCaeca
GutPost
Blood3h
BloodNBF
BloodTwoNBF
FatBodyBF
GutMidBF
Mean 2.87 +/− 2.75

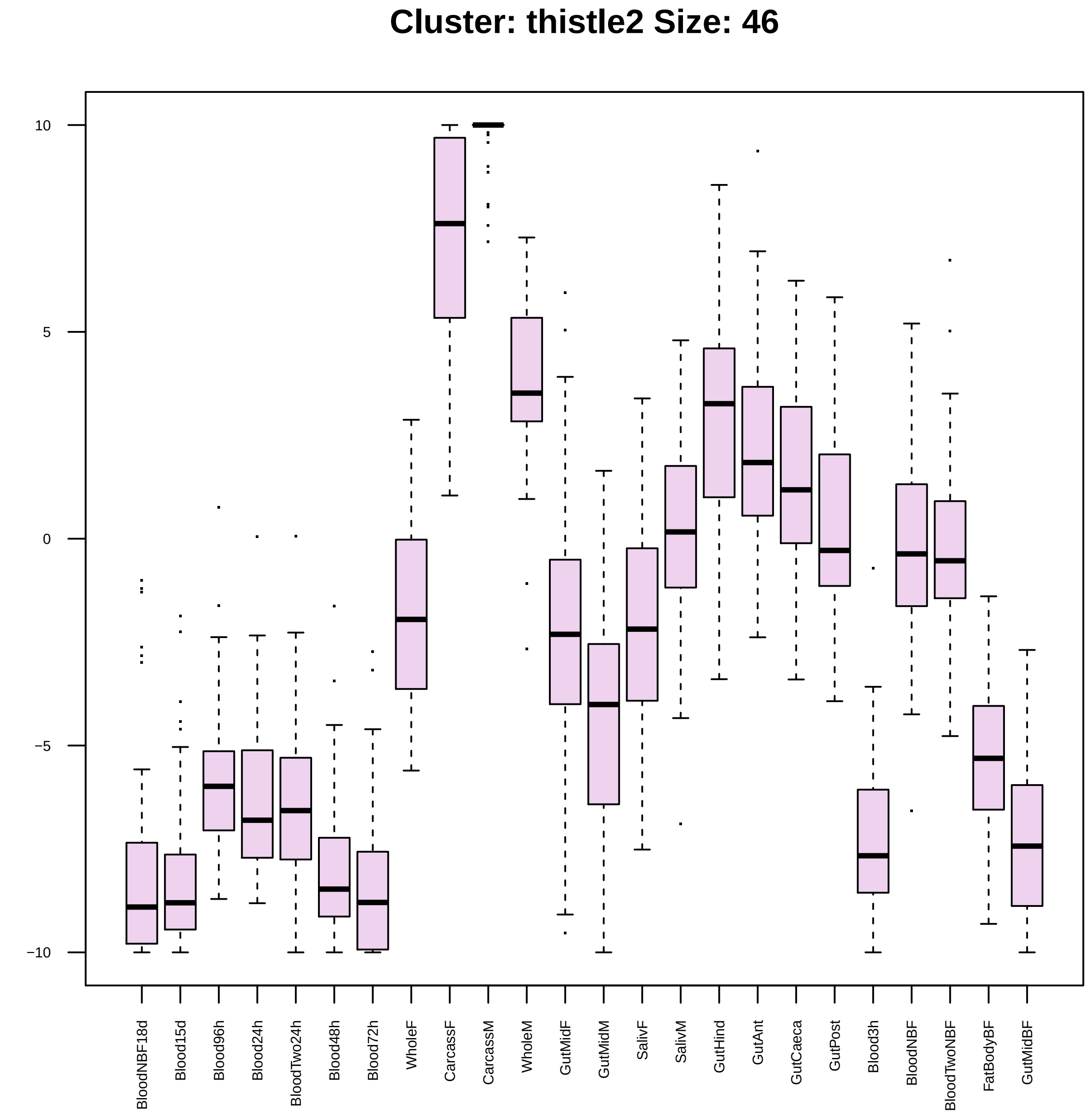
Cluster: thistle2 Size: 46
10
5
0
−5
−10
BloodNBF18d
Blood15d
Blood96h
Blood24h
BloodTwo24h
Blood48h
Blood72h
WholeF
CarcassF
CarcassM
WholeM
GutMidF
GutMidM
SalivF
SalivM
GutHind
GutAnt
GutCaeca
GutPost
Blood3h
BloodNBF
BloodTwoNBF
FatBodyBF
GutMidBF

| | GO.ID | BPCluster: thistle2 Size: 46 | Annotated | Significant | Expected | Rank in ClassicF | Weight01F | ClassicF |
|---|---|---|---|---|---|---|---|---|
| *29* | GO:0009165 | nucleotide biosynthetic process | 90 | 3 | 0.44 | 63 | 1.000 | 0.0092 |

| | GO.ID | MFCluster: thistle2 Size: 46 | Annotated | Significant | Expected | Rank in ClassicF | Weight01F | ClassicF |
|---|---|---|---|---|---|---|---|---|
| 5 | GO:0051539 | 4 iron, 4 sulfur cluster binding | 14 | 2 | 0.06 | 22 | 0.0015 | 0.00148 |
| 6 | GO:0022857 | transmembrane transporter activity | 442 | 10 | 1.84 | 9 | 0.0015 | 6.7e−06 |
| 7 | GO:0015078 | proton transmembrane transporter activit... | 52 | 6 | 0.22 | 8 | 0.0023 | 5.4e−08 |
| 8 | GO:0015662 | ATPase activity, coupled to transmembran... | 20 | 2 | 0.08 | 23 | 0.0030 | 0.00304 |
| 9 | GO:0044769 | ATPase activity, coupled to transmembran... | 22 | 2 | 0.09 | 25 | 0.0040 | 0.00367 |
| 10 | GO:0036442 | proton−exporting ATPase activity | 27 | 2 | 0.11 | 26 | 0.0240 | 0.00551 |
| 26 | GO:0008324 | cation transmembrane transporter activit... | 210 | 6 | 0.87 | 17 | 1.0000 | 0.00019 |
| 27 | GO:0003824 | catalytic activity | 3160 | 21 | 13.15 | 24 | 1.0000 | 0.00336 |
| 29 | GO:0051540 | metal cluster binding | 44 | 3 | 0.18 | 20 | 1.0000 | 0.00077 |

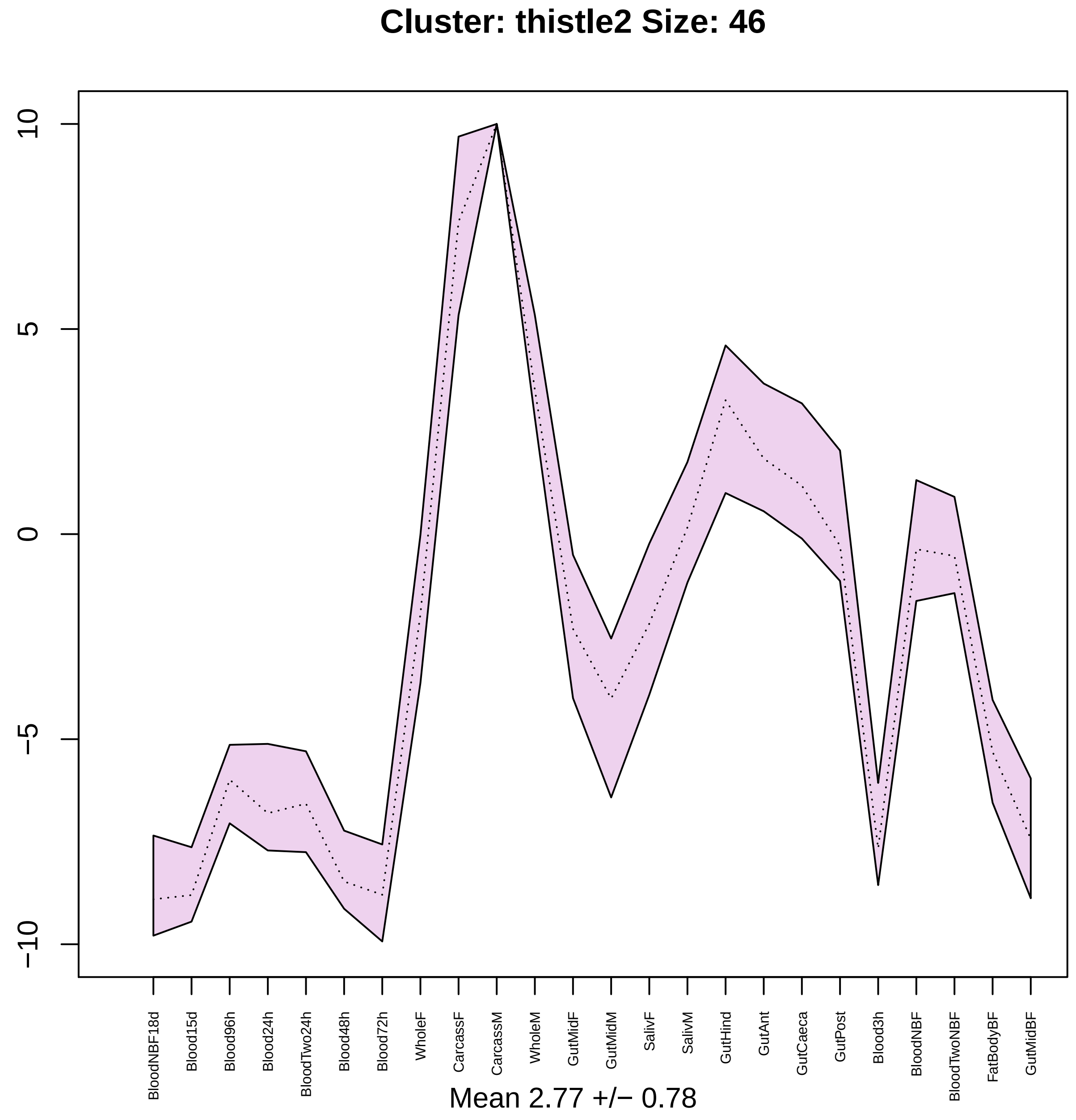

Cluster: thistle2 Size: 46
10
5
0
−5
−10
BloodNBF18d
Blood15d
Blood96h
Blood24h
BloodTwo24h
Blood48h
Blood72h
WholeF
CarcassF
CarcassM
WholeM
GutMidF
GutMidM
SalivF
SalivM
GutHind
GutAnt
GutCaeca
GutPost
Blood3h
BloodNBF
BloodTwoNBF
FatBodyBF
GutMidBF
Mean 2.77 +/− 0.78

## Cluster: palevioletred3 Size: 39

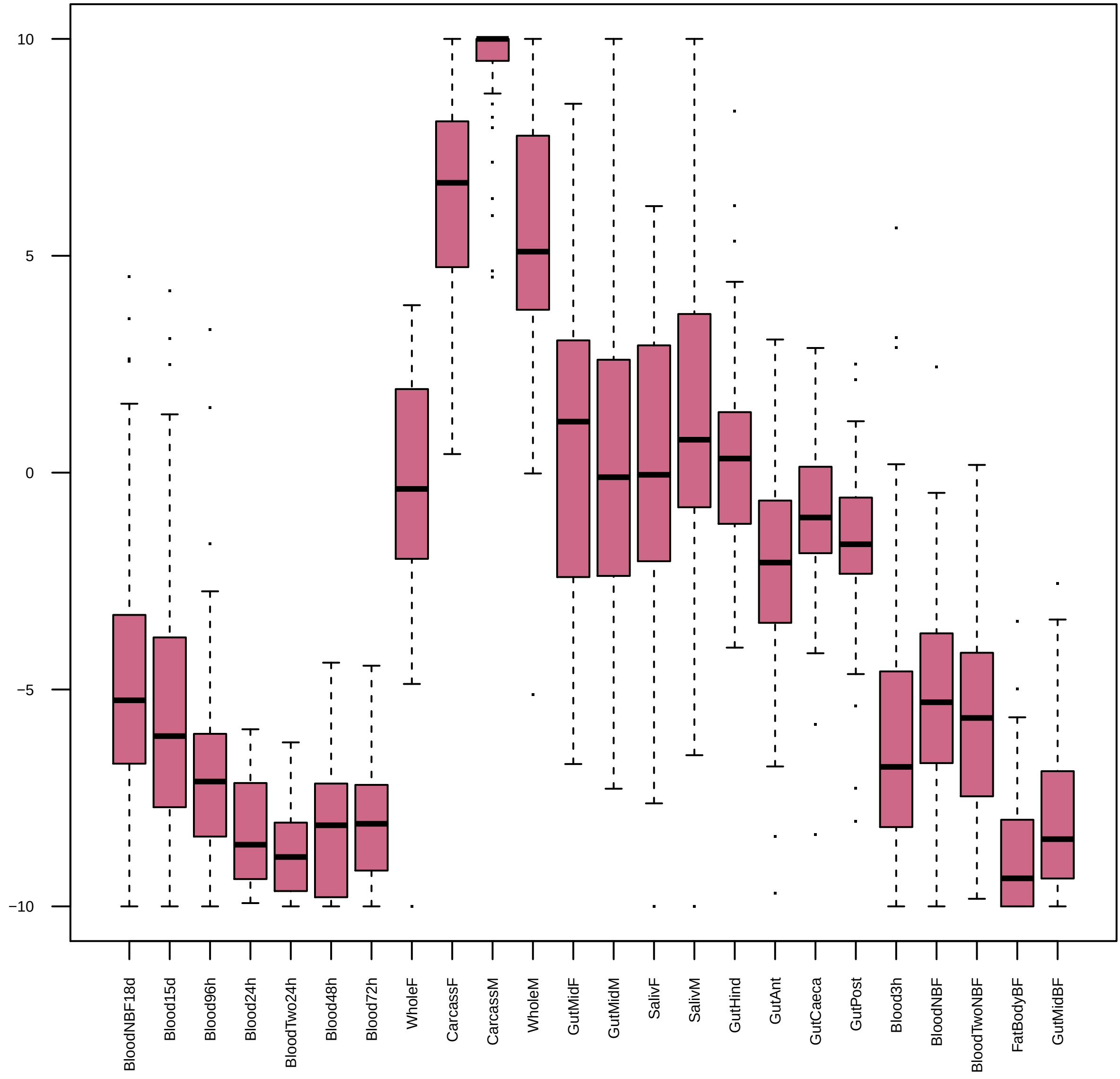

| | GO.ID | BPCluster: palevioletred3 Size: 39 | Annotated | Significant | Expected | Rank in ClassicF | Weight01F | ClassicF |
|---|---|---|---|---|---|---|---|---|
| 1 | GO:0016226 | iron−sulfur cluster assembly | 11 | 2 | 0.04 | 3 | 0.00061 | 0.00061 |
| 2 | GO:0042181 | ketone biosynthetic process | 14 | 2 | 0.05 | 5 | 0.00101 | 0.00101 |
| 3 | GO:0006733 | oxidoreduction coenzyme metabolic proces... | 34 | 2 | 0.12 | 8 | 0.00596 | 0.00596 |
| 4 | GO:0045454 | cell redox homeostasis | 36 | 2 | 0.12 | 9 | 0.00667 | 0.00667 |
| 5 | GO:0009058 | biosynthetic process | 1357 | 5 | 4.68 | 149 | 0.00740 | 0.51731 |
| 6 | GO:0055114 | oxidation−reduction process | 545 | 6 | 1.88 | 10 | 0.00863 | 0.00863 |
| 14 | GO:0019725 | cellular homeostasis | 84 | 4 | 0.29 | 1 | 0.06142 | 0.00017 |

| | GO.ID | MFCluster: palevioletred3 Size: 39 | Annotated | Significant | Expected | Rank in ClassicF | Weight01F | ClassicF |
|---|---|---|---|---|---|---|---|---|
| *1* | GO:0015035 | protein disulfide oxidoreductase activit... | 14 | 2 | 0.04 | 1 | 0.00072 | 0.00072 |
| *2* | GO:0051536 | iron−sulfur cluster binding | 44 | 2 | 0.13 | 4 | 0.00710 | 0.00710 |
| *3* | GO:0009055 | electron transfer activity | 48 | 2 | 0.14 | 6 | 0.00841 | 0.00841 |

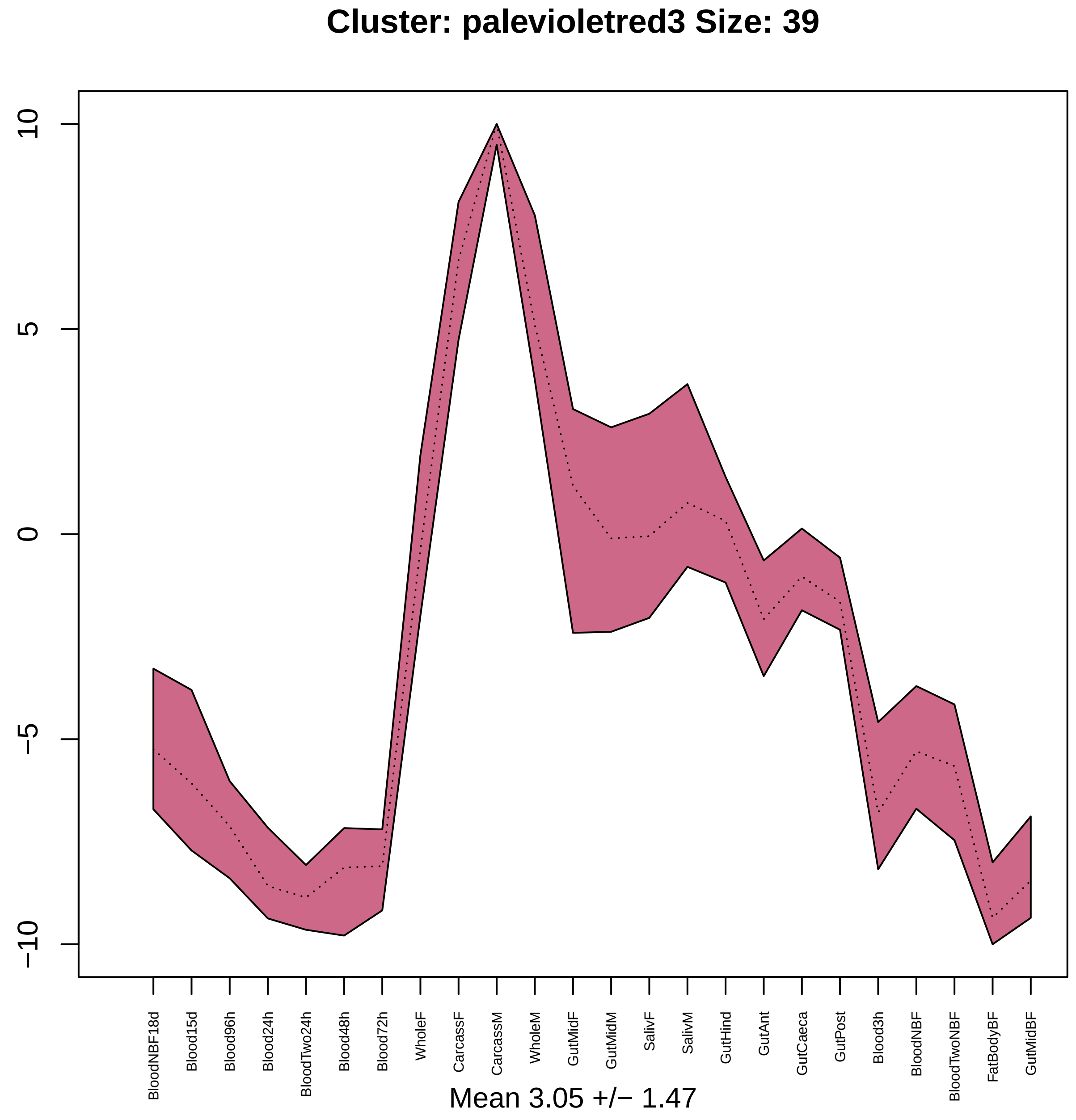

Cluster: palevioletred3 Size: 39
10
5
0
−5
−10
BloodNBF18d
Blood15d
Blood96h
Blood24h
BloodTwo24h
Blood48h
Blood72h
WholeF
CarcassF
CarcassM
WholeM
GutMidF
GutMidM
SalivF
SalivM
GutHind
GutAnt
GutCaeca
GutPost
Blood3h
BloodNBF
BloodTwoNBF
FatBodyBF
GutMidBF
Mean 3.05 +/− 1.47

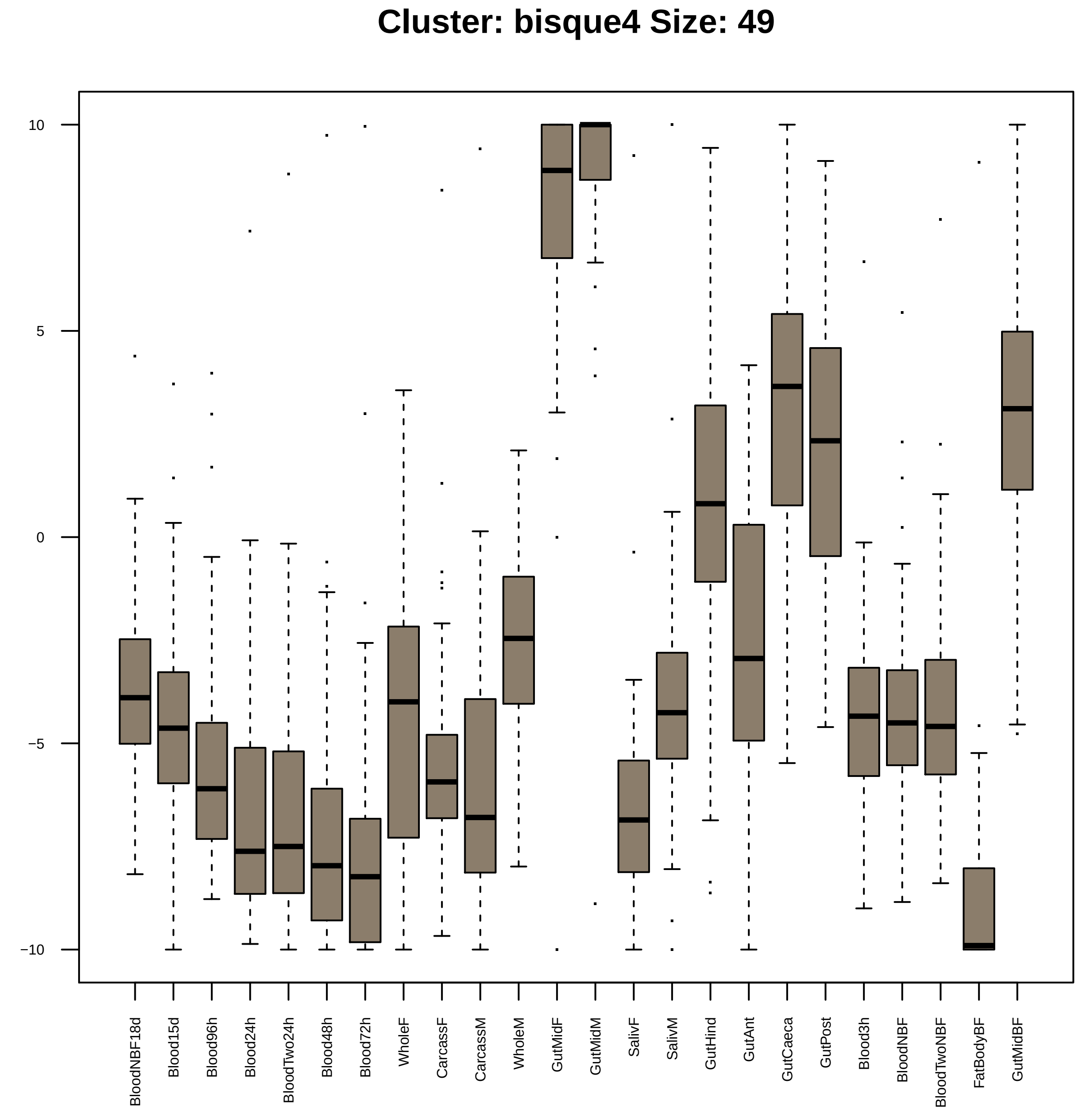

Cluster: bisque4 Size: 49
10
5
0
−5
−10
BloodNBF18d
Blood15d
Blood96h
Blood24h
BloodTwo24h
Blood48h
Blood72h
WholeF
CarcassF
CarcassM
WholeM
GutMidF
GutMidM
SalivF
SalivM
GutHind
GutAnt
GutCaeca
GutPost
Blood3h
BloodNBF
BloodTwoNBF
FatBodyBF
GutMidBF

| | GO.ID | BPCluster: bisque4 Size: 49 | Annotated | Significant | Expected | Rank in ClassicF | Weight01F | ClassicF |
|---|---|---|---|---|---|---|---|---|
| *1* | GO:0007156 | homophilic cell adhesion via plasma memb... | 23 | 2 | 0.1 | 1 | 0.0048 | 0.0048 |

| | GO.ID | MFCluster: bisque4 Size: 49 | Annotated | Significant | Expected | Rank in ClassicF | Weight01F | ClassicF |
|---|---|---|---|---|---|---|---|---|
| 1 | GO:0016705 | oxidoreductase activity, acting on paire... | 133 | 4 | 0.54 | 2 | 0.0018 | 0.00184 |
| 2 | GO:0005509 | calcium ion binding | 173 | 4 | 0.70 | 5 | 0.0048 | 0.00476 |
| 3 | GO:0008237 | metallopeptidase activity | 104 | 4 | 0.42 | 1 | 0.0056 | 0.00074 |
| 4 | GO:0001882 | nucleoside binding | 142 | 3 | 0.57 | 15 | 0.0076 | 0.01880 |
| 5 | GO:0004497 | monooxygenase activity | 110 | 3 | 0.44 | 10 | 0.0095 | 0.00947 |
| 29 | GO:0003824 | catalytic activity | 3160 | 20 | 12.71 | 6 | 0.4275 | 0.00549 |

## Cluster: bisque4 Size: 49

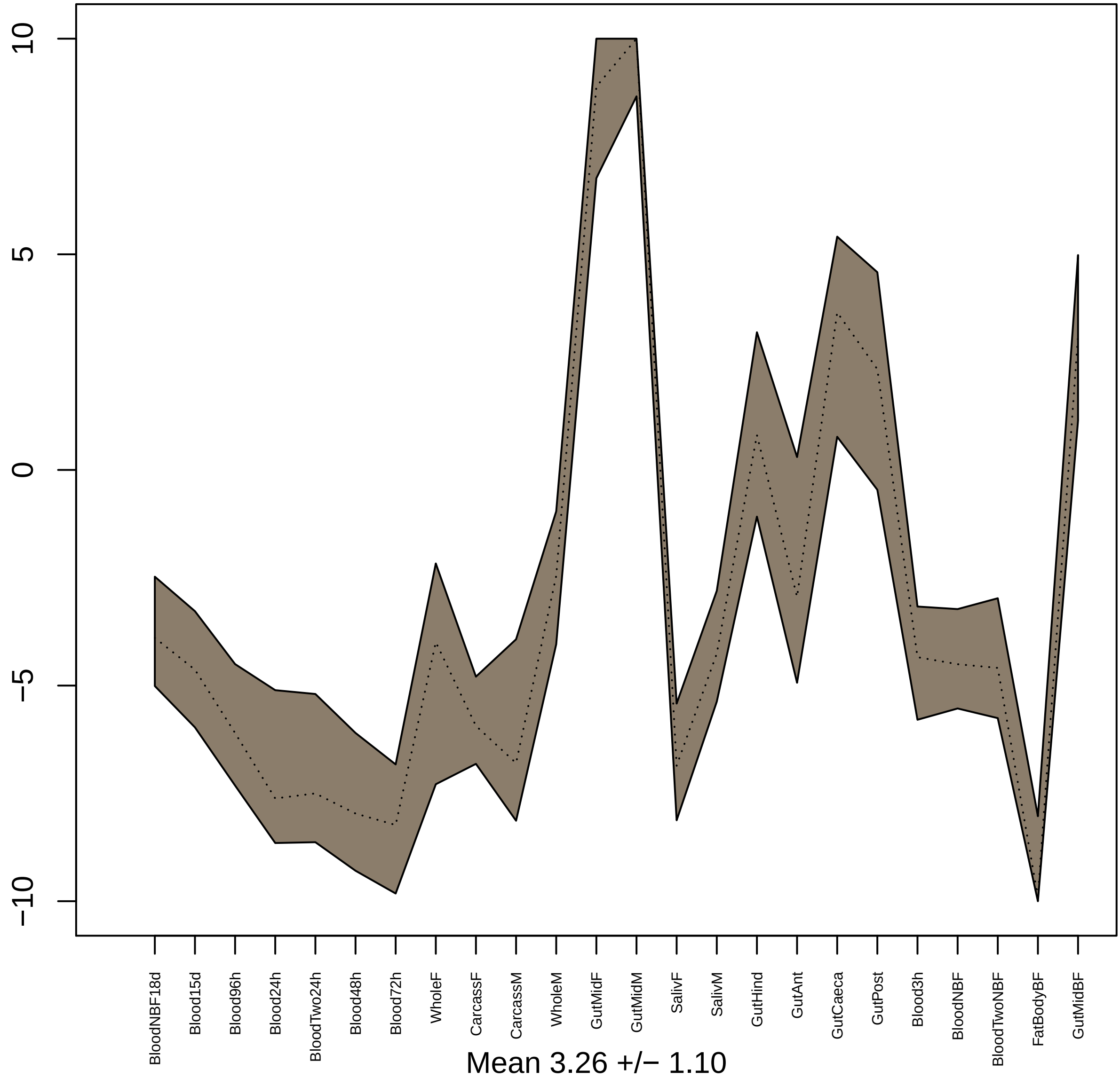

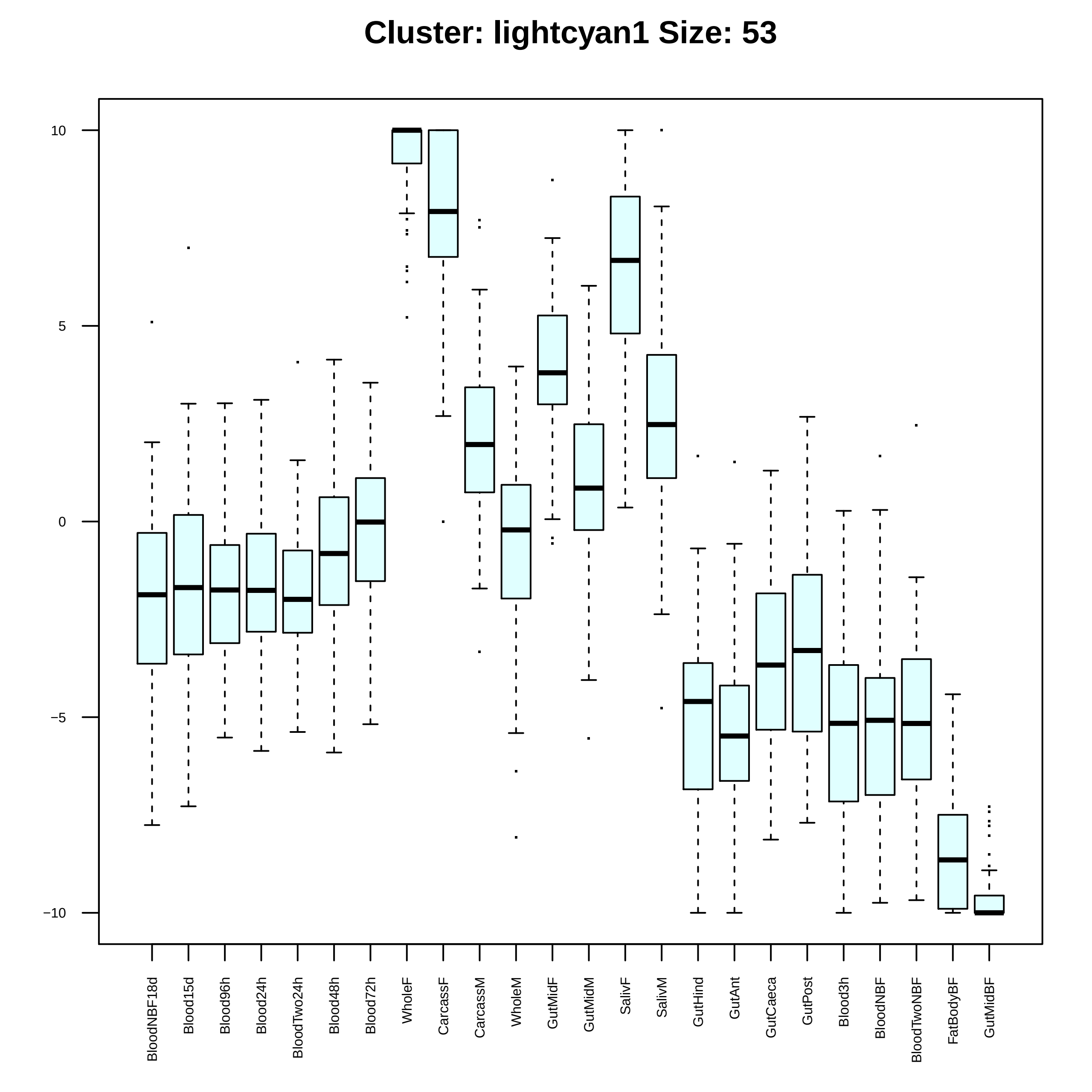

Cluster: lightcyan1 Size: 53
10
5
0
−5
−10
BloodNBF18d
Blood15d
Blood96h
Blood24h
BloodTwo24h
Blood48h
Blood72h
WholeF
CarcassF
CarcassM
WholeM
GutMidF
GutMidM
SalivF
SalivM
GutHind
GutAnt
GutCaeca
GutPost
Blood3h
BloodNBF
BloodTwoNBF
FatBodyBF
GutMidBF

| | GO.ID | BPCluster: lightcyan1 Size: 53 | Annotated | Significant | Expected | Rank in ClassicF | Weight01F | ClassicF |
|---|---|---|---|---|---|---|---|---|
| *4* | GO:0042254 | ribosome biogenesis | 64 | 10 | 0.37 | 1 | 0.00036 | 1.4e−12 |
| *5* | GO:0006378 | mRNA polyadenylation | 13 | 2 | 0.08 | 27 | 0.00245 | 0.0025 |
| *6* | GO:0006379 | mRNA cleavage | 13 | 2 | 0.08 | 28 | 0.00245 | 0.0025 |

| | GO.ID | MFCluster: lightcyan1 Size: 53 | Annotated | Significant | Expected | Rank in ClassicF | Weight01F | ClassicF |
|---|---|---|---|---|---|---|---|---|
| *2* | GO:0003729 | mRNA binding | 99 | 4 | 0.56 | 6 | 0.011 | 0.00230 |
| *4* | GO:0003723 | RNA binding | 307 | 8 | 1.75 | 2 | 0.022 | 0.00028 |
| *6* | GO:0008026 | ATP−dependent helicase activity | 76 | 4 | 0.43 | 3 | 0.028 | 0.00086 |
| *18* | GO:0016887 | ATPase activity | 233 | 5 | 1.33 | 12 | 0.191 | 0.00980 |
| *19* | GO:0005488 | binding | 4627 | 35 | 26.32 | 7 | 0.193 | 0.00234 |

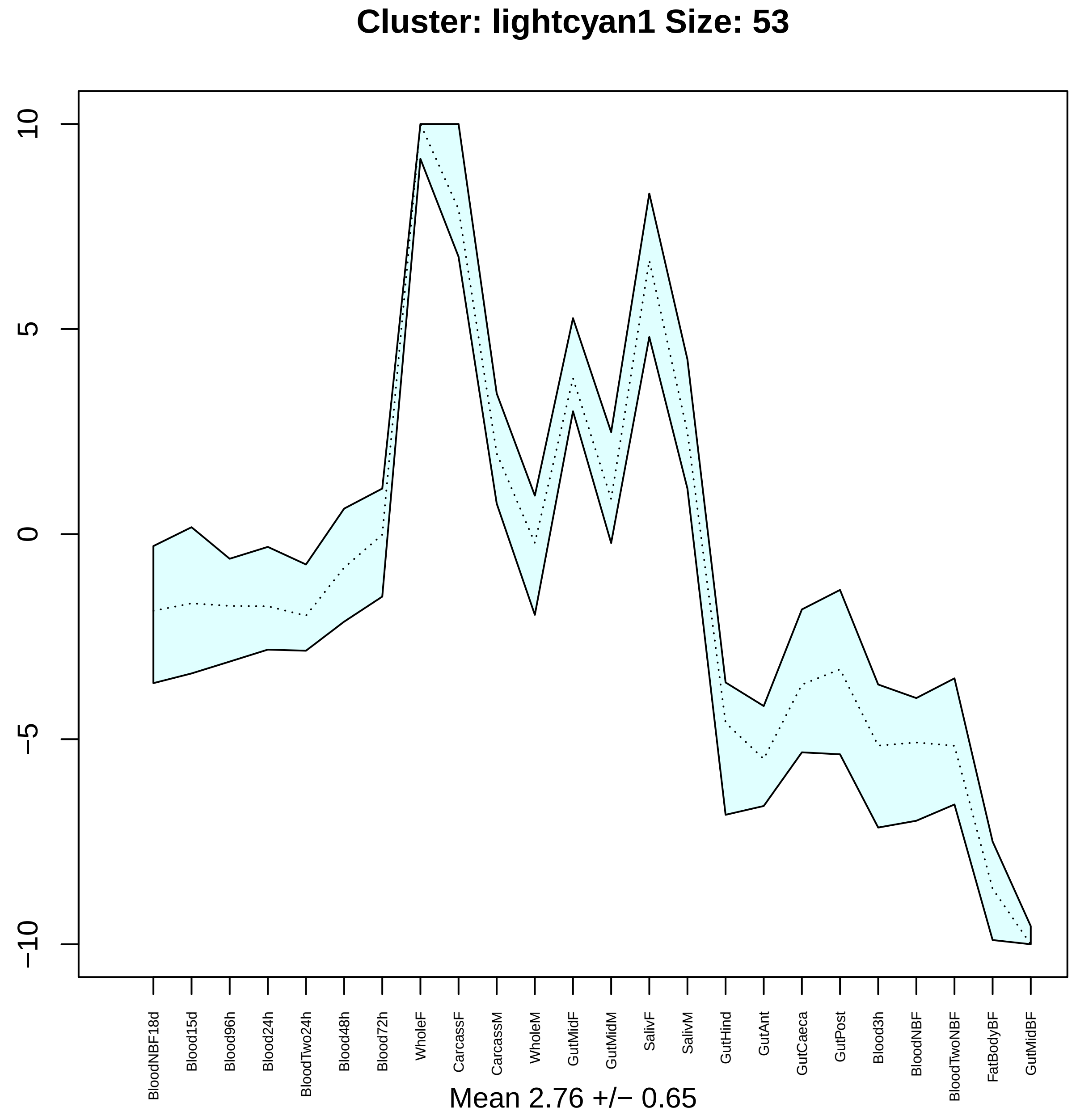
Cluster: lightcyan1 Size: 53
10
5
0
−5
−10
BloodNBF18d
Blood15d
Blood96h
Blood24h
BloodTwo24h
Blood48h
Blood72h
WholeF
CarcassF
CarcassM
WholeM
GutMidF
GutMidM
SalivF
SalivM
GutHind
GutAnt
GutCaeca
GutPost
Blood3h
BloodNBF
BloodTwoNBF
FatBodyBF
GutMidBF
Mean 2.76 +/− 0.65

## Cluster: violet Size: 63

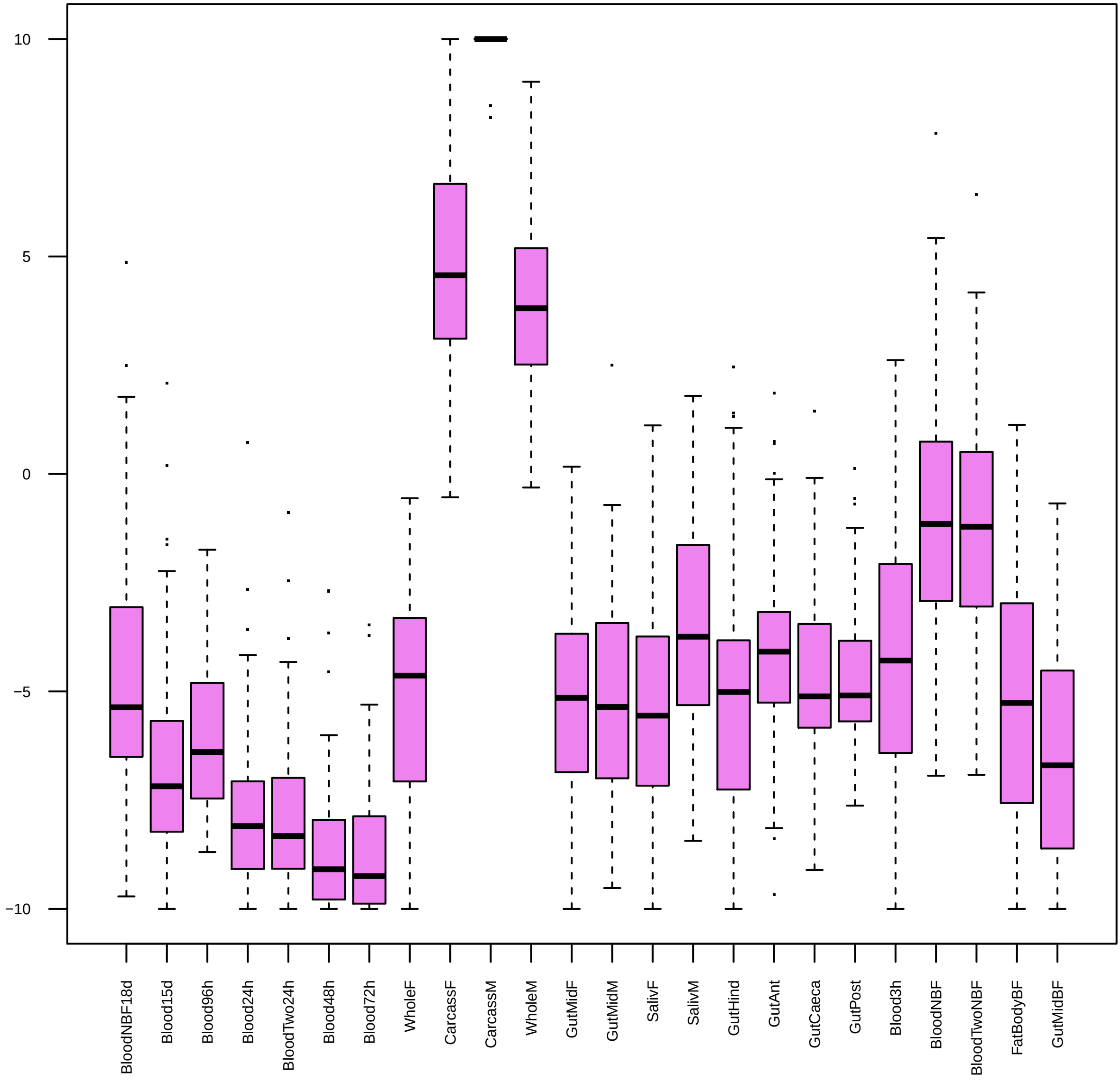

| | GO.ID | BPCluster: violet Size: 63 | Annotated | Significant | Expected | Rank in ClassicF | Weight01F | ClassicF |
|---|---|---|---|---|---|---|---|---|
| 1 | GO:0042246 | tissue regeneration | 10 | 2 | 0.06 | 1 | 0.0014 | 0.0014 |
| 2 | GO:0007155 | cell adhesion | 136 | 4 | 0.77 | 3 | 0.0069 | 0.0069 |
| 3 | GO:0071805 | potassium ion transmembrane transport | 23 | 2 | 0.13 | 5 | 0.0073 | 0.0073 |

| | GO.ID | MFCluster: violet Size: 63 | Annotated | Significant | Expected | Rank in ClassicF | Weight01F | ClassicF |
|---|---|---|---|---|---|---|---|---|
| *1* | GO:0005267 | potassium channel activity | 25 | 2 | 0.12 | 11 | 0.0068 | 0.00677 |
| *2* | GO:0005215 | transporter activity | 548 | 9 | 2.74 | 7 | 0.0112 | 0.00116 |
| *12* | GO:0022839 | ion gated channel activity | 87 | 4 | 0.43 | 5 | 0.1135 | 0.00087 |

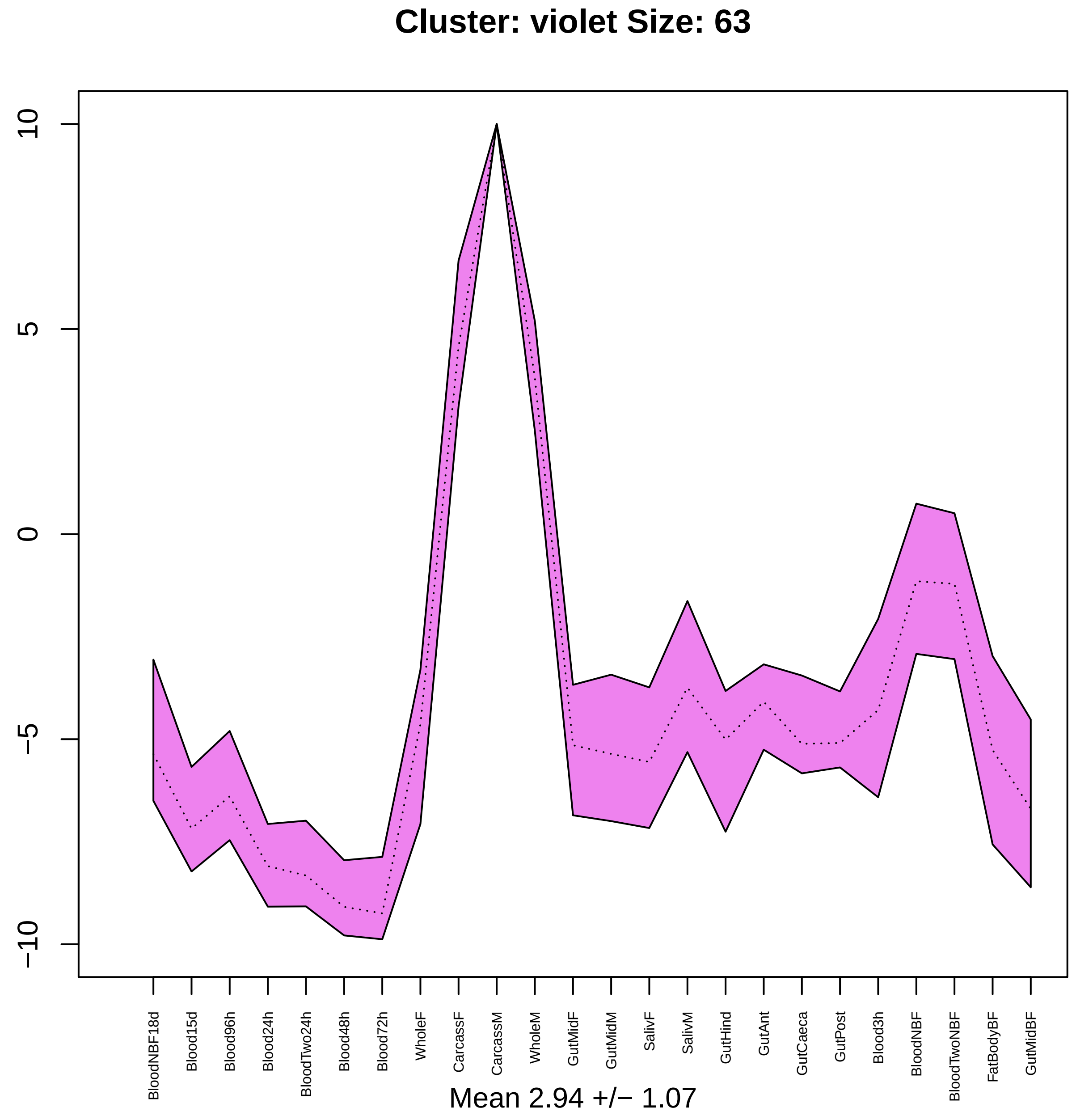

Cluster: violet Size: 63
10
5
0
−5
−10
BloodNBF18d
Blood15d
Blood96h
Blood24h
BloodTwo24h
Blood48h
Blood72h
WholeF
CarcassF
CarcassM
WholeM
GutMidF
GutMidM
SalivF
SalivM
GutHind
GutAnt
GutCaeca
GutPost
Blood3h
BloodNBF
BloodTwoNBF
FatBodyBF
GutMidBF
Mean 2.94 +/− 1.07

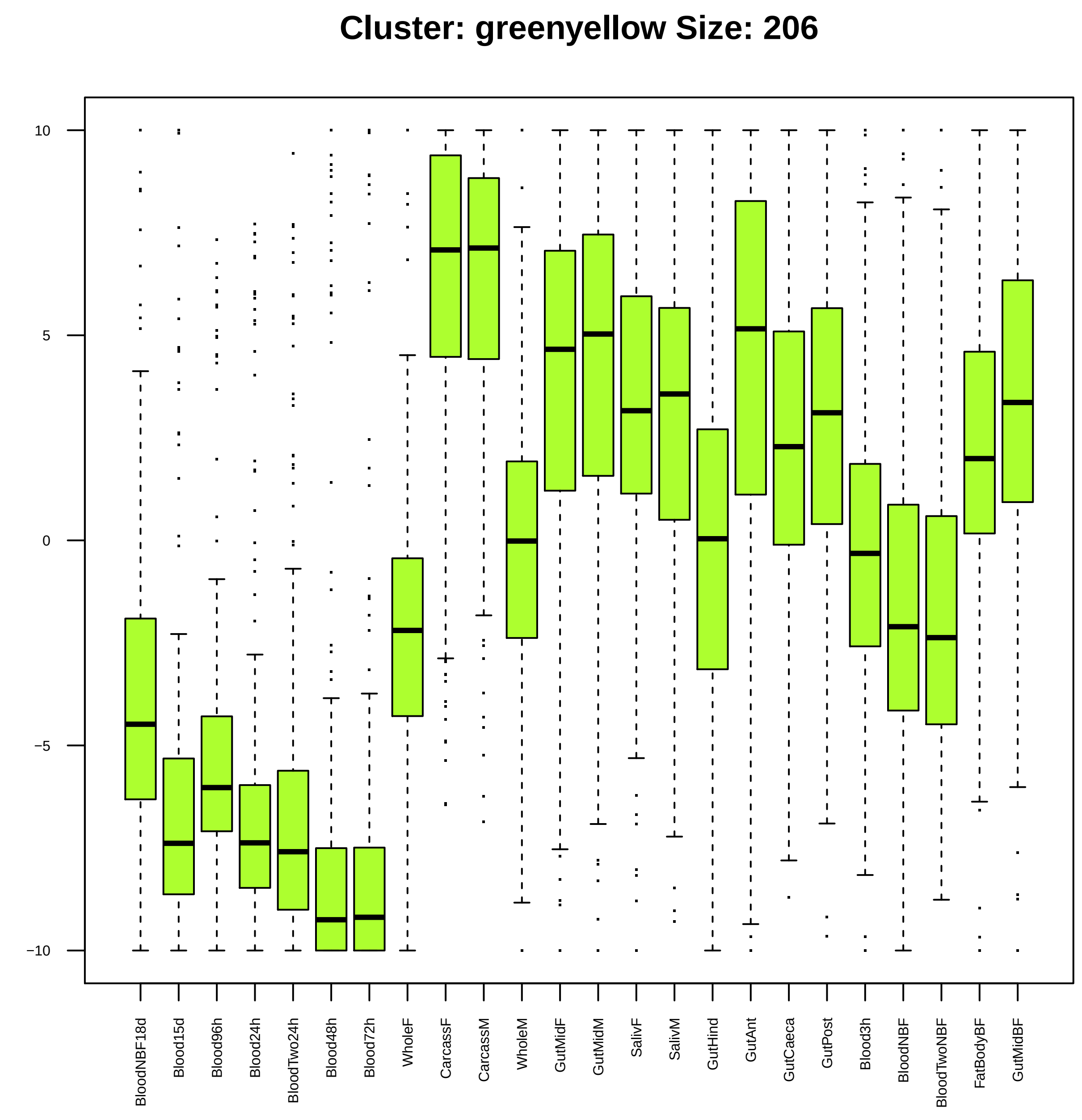
Cluster: greenyellow Size: 206
10
5
0
−5
−10
BloodNBF18d
Blood15d
Blood96h
Blood24h
BloodTwo24h
Blood48h
Blood72h
WholeF
CarcassF
CarcassM
WholeM
GutMidF
GutMidM
SalivF
SalivM
GutHind
GutAnt
GutCaeca
GutPost
Blood3h
BloodNBF
BloodTwoNBF
FatBodyBF
GutMidBF

| | GO.ID | BPCluster: greenyellow Size: 206 | Annotated | Significant | Expected | Rank in ClassicF | Weight01F | ClassicF |
|---|---|---|---|---|---|---|---|---|
| *27* | GO:0007606 | sensory perception of chemical stimulus | 149 | 8 | 2.62 | 1 | 0.097 | 0.0045 |

| | GO.ID | MFCluster: greenyellow Size: 206 | Annotated | Significant | Expected | Rank in ClassicF | Weight01F | ClassicF |
|---|---|---|---|---|---|---|---|---|
| 1 | GO:0042626 | ATPase activity, coupled to transmembran... | 73 | 5 | 1.42 | 11 | 0.0032 | 0.01343 |
| 2 | GO:0003705 | transcription factor activity, RNA polym... | 32 | 4 | 0.62 | 6 | 0.0032 | 0.00321 |
| 16 | GO:0043565 | sequence−specific DNA binding | 212 | 10 | 4.12 | 8 | 0.0916 | 0.00806 |
| 17 | GO:0019199 | transmembrane receptor protein kinase ac... | 15 | 3 | 0.29 | 4 | 0.0923 | 0.00275 |
| 18 | GO:0003700 | DNA−binding transcription factor activit... | 282 | 13 | 5.48 | 5 | 0.0943 | 0.00319 |
| 25 | GO:0004888 | transmembrane signaling receptor activit... | 255 | 14 | 4.95 | 1 | 0.1530 | 0.00041 |

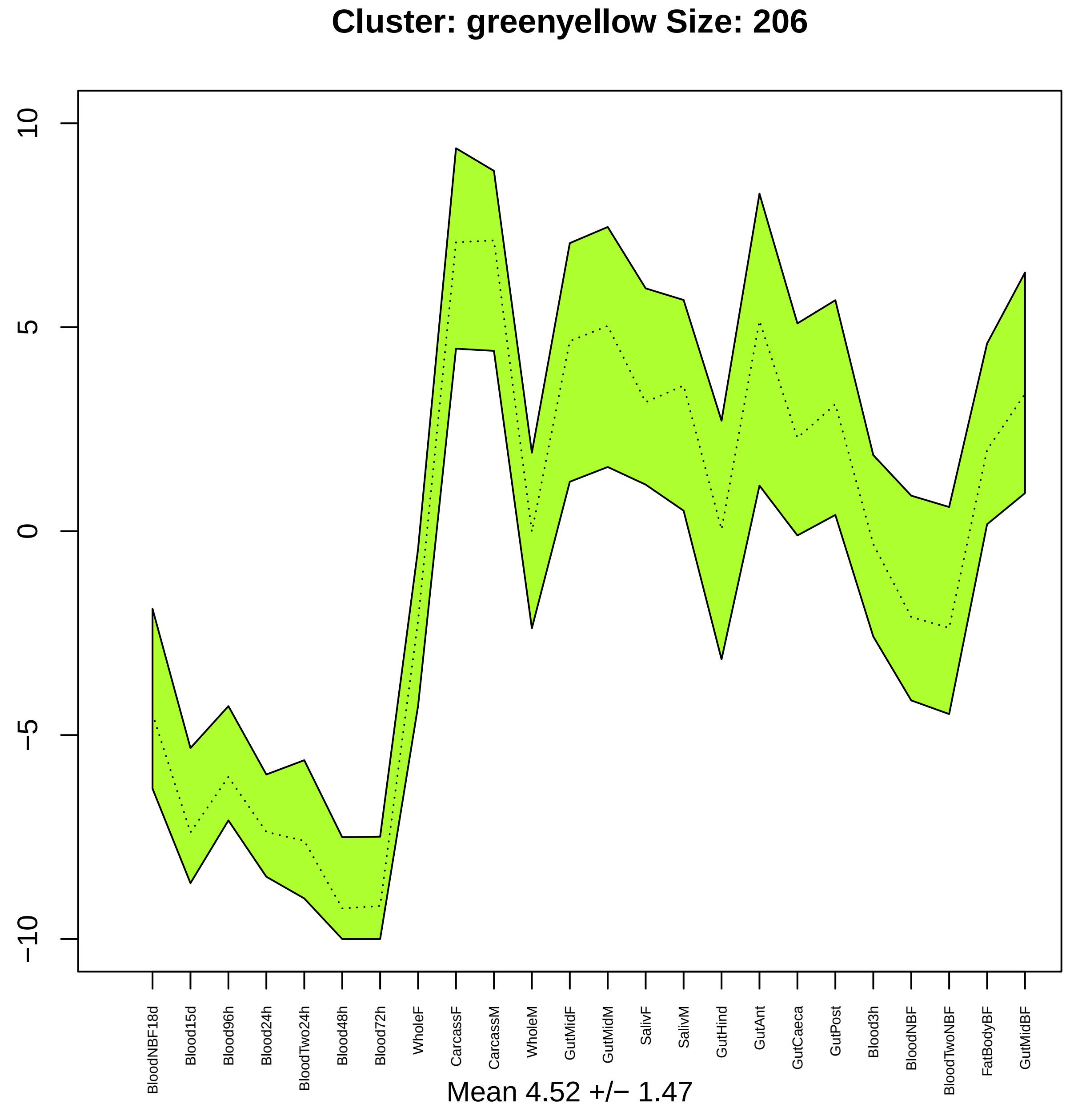

Cluster: greenyellow Size: 206
10
5
0
−5
−10
BloodNBF18d
Blood15d
Blood96h
Blood24h
BloodTwo24h
Blood48h
Blood72h
WholeF
CarcassF
CarcassM
WholeM
GutMidF
GutMidM
SalivF
SalivM
GutHind
GutAnt
GutCaeca
GutPost
Blood3h
BloodNBF
BloodTwoNBF
FatBodyBF
GutMidBF
Mean 4.52 +/− 1.47

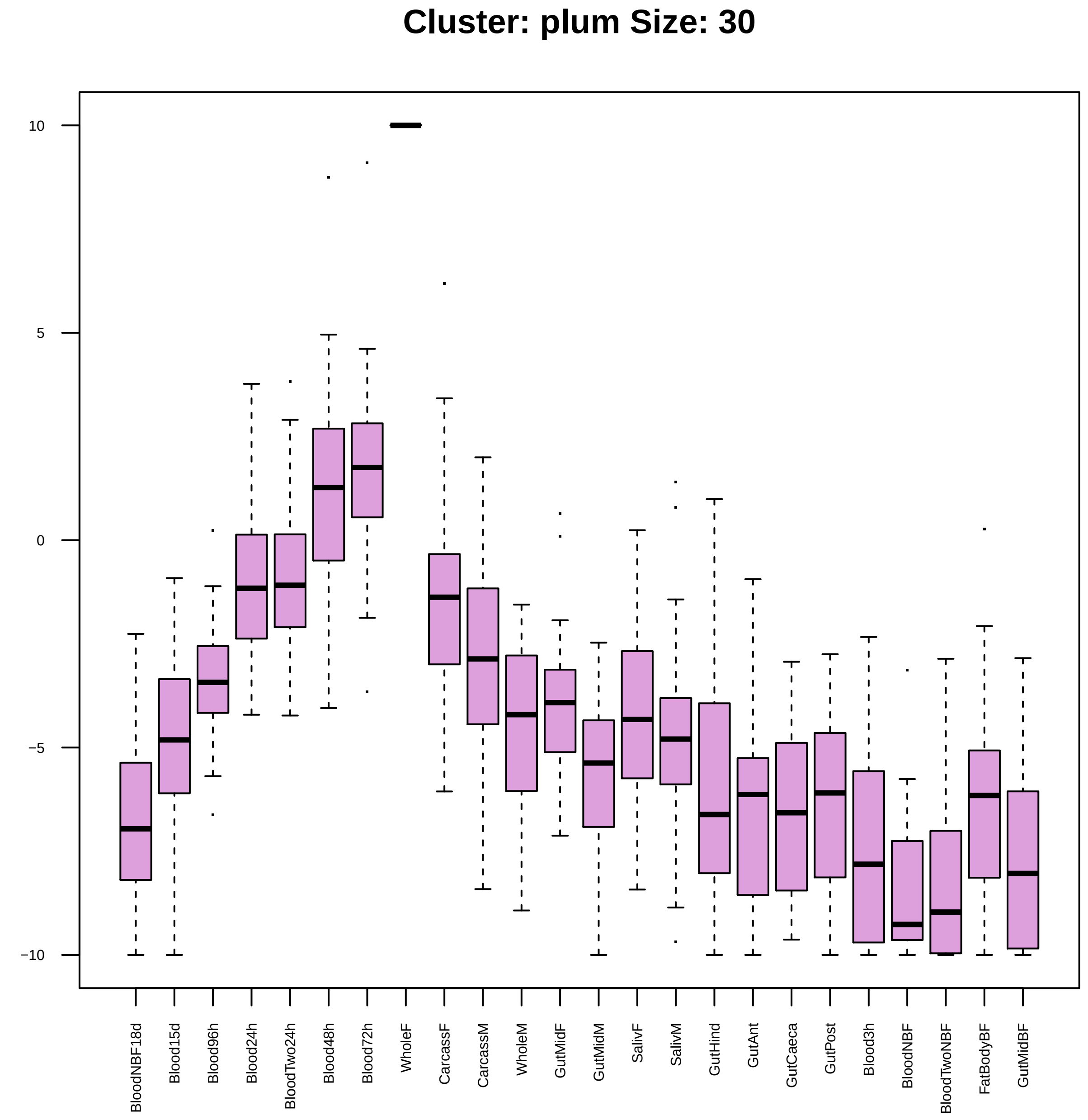
Cluster: plum Size: 30
10
5
0
−5
−10
BloodNBF18d
Blood15d
Blood96h
Blood24h
BloodTwo24h
Blood48h
Blood72h
WholeF
CarcassF
CarcassM
WholeM
GutMidF
GutMidM
SalivF
SalivM
GutHind
GutAnt
GutCaeca
GutPost
Blood3h
BloodNBF
BloodTwoNBF
FatBodyBF
GutMidBF

| | GO.ID | BPCluster: plum Size: 30 | Annotated | Significant | Expected | Rank in ClassicF | Weight01F | ClassicF |
|---|---|---|---|---|---|---|---|---|
| *1* | GO:0008152 | metabolic process | 4285 | 13 | 10.08 | 69 | 0.0030 | 0.0855 |
| *2* | GO:0007018 | microtubule−based movement | 65 | 2 | 0.15 | 4 | 0.0099 | 0.0099 |
| *3* | GO:0006913 | nucleocytoplasmic transport | 58 | 2 | 0.14 | 1 | 0.0176 | 0.0079 |

| | GO.ID | MFCluster: plum Size: 30 | Annotated | Significant | Expected | Rank in ClassicF | Weight01F | ClassicF |
|---|---|---|---|---|---|---|---|---|
| *1* | GO:0005524 | ATP binding | 592 | 7 | 1.48 | 5 | 0.00035 | 0.00035 |
| *2* | GO:0003777 | microtubule motor activity | 40 | 2 | 0.10 | 22 | 0.00434 | 0.00434 |
| *3* | GO:0008017 | microtubule binding | 55 | 2 | 0.14 | 24 | 0.00809 | 0.00809 |
| *4* | GO:0008094 | DNA−dependent ATPase activity | 33 | 2 | 0.08 | 20 | 0.02804 | 0.00297 |
| *6* | GO:0004386 | helicase activity | 103 | 3 | 0.26 | 16 | 0.03538 | 0.00198 |
| *15* | GO:0042623 | ATPase activity, coupled | 193 | 4 | 0.48 | 8 | 0.19992 | 0.00113 |
| *18* | GO:0000166 | nucleotide binding | 998 | 8 | 2.49 | 14 | 0.34904 | 0.00158 |
| *25* | GO:0016887 | ATPase activity | 233 | 4 | 0.58 | 17 | 1.00000 | 0.00228 |
| *26* | GO:1901265 | nucleoside phosphate binding | 998 | 8 | 2.49 | 15 | 1.00000 | 0.00158 |
| *27* | GO:0035639 | purine ribonucleoside triphosphate bindi... | 725 | 7 | 1.81 | 10 | 1.00000 | 0.00119 |
| *29* | GO:0032553 | ribonucleotide binding | 740 | 7 | 1.85 | 12 | 1.00000 | 0.00135 |
| *30* | GO:0032555 | purine ribonucleotide binding | 732 | 7 | 1.83 | 11 | 1.00000 | 0.00126 |

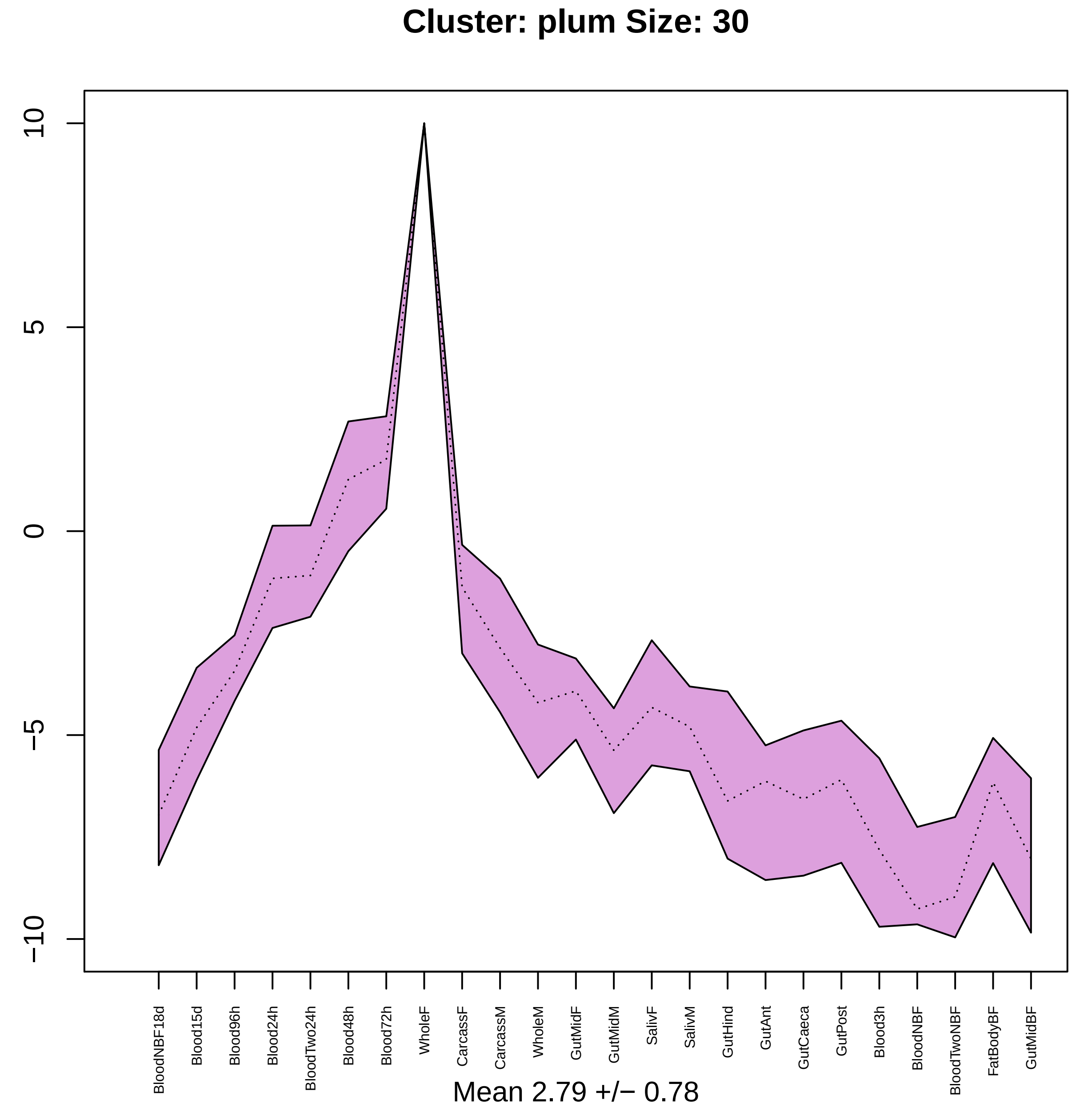

Cluster: plum Size: 30
10
5
0
−5
−10
BloodNBF18d
Blood15d
Blood96h
Blood24h
BloodTwo24h
Blood48h
Blood72h
WholeF
CarcassF
CarcassM
WholeM
GutMidF
GutMidM
SalivF
SalivM
GutHind
GutAnt
GutCaeca
GutPost
Blood3h
BloodNBF
BloodTwoNBF
FatBodyBF
GutMidBF
Mean 2.79 +/− 0.78

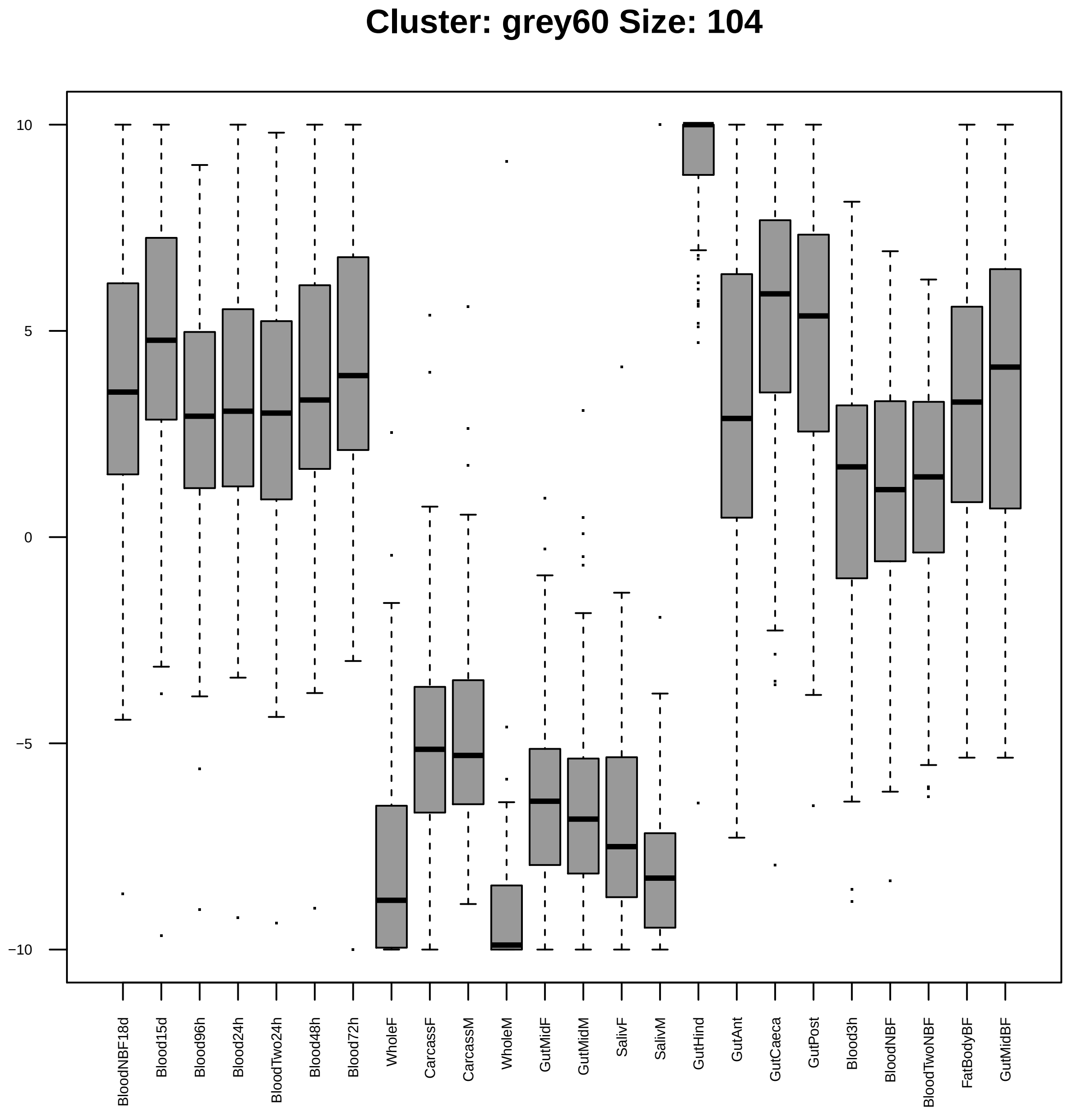

Cluster: grey60 Size: 104
10
5
0
−5
−10
BloodNBF18d
Blood15d
Blood96h
Blood24h
BloodTwo24h
Blood48h
Blood72h
WholeF
CarcassF
CarcassM
WholeM
GutMidF
GutMidM
SalivF
SalivM
GutHind
GutAnt
GutCaeca
GutPost
Blood3h
BloodNBF
BloodTwoNBF
FatBodyBF
GutMidBF

| | GO.ID | BPCluster: grey60 Size: 104 | Annotated | Significant | Expected | Rank in ClassicF | Weight01F | ClassicF |
|---|---|---|---|---|---|---|---|---|
| 2 | GO:0032482 | Rab protein signal transduction | 27 | 4 | 0.28 | 14 | 0.00014 | 0.00014 |
| 3 | GO:0007264 | small GTPase mediated signal transductio... | 153 | 9 | 1.56 | 4 | 0.00021 | 2.1e−05 |
| 4 | GO:0048488 | synaptic vesicle endocytosis | 38 | 5 | 0.39 | 9 | 0.00142 | 3.6e−05 |
| 5 | GO:0007269 | neurotransmitter secretion | 61 | 4 | 0.62 | 31 | 0.00332 | 0.00332 |
| 6 | GO:0043161 | proteasome−mediated ubiquitin−dependent ... | 37 | 3 | 0.38 | 39 | 0.00613 | 0.00613 |
| 7 | GO:0007040 | lysosome organization | 12 | 2 | 0.12 | 40 | 0.00632 | 0.00632 |
| 8 | GO:0008347 | glial cell migration | 13 | 2 | 0.13 | 44 | 0.00742 | 0.00742 |
| 9 | GO:0016183 | synaptic vesicle coating | 13 | 2 | 0.13 | 45 | 0.00742 | 0.00742 |
| 10 | GO:0006796 | phosphate−containing compound metabolic ... | 642 | 6 | 6.54 | 831 | 0.00802 | 0.65027 |
| 11 | GO:0051124 | synaptic growth at neuromuscular junctio... | 57 | 3 | 0.58 | 79 | 0.00969 | 0.02000 |
| 12 | GO:0000413 | protein peptidyl−prolyl isomerization | 15 | 2 | 0.15 | 49 | 0.00986 | 0.00986 |
| 16 | GO:0046907 | intracellular transport | 280 | 10 | 2.85 | 20 | 0.02062 | 0.00048 |
| 20 | GO:0072528 | pyrimidine−containing compound biosynthe... | 15 | 2 | 0.15 | 50 | 0.02987 | 0.00986 |

| | GO.ID | MFCluster: grey60 Size: 104 | Annotated | Significant | Expected | Rank in ClassicF | Weight01F | ClassicF |
|---|---|---|---|---|---|---|---|---|
| *3* | GO:0004298 | threonine−type endopeptidase activity | 14 | 2 | 0.14 | 13 | 0.0085 | 0.0085 |
| *8* | GO:0016776 | phosphotransferase activity, phosphate g... | 14 | 2 | 0.14 | 14 | 0.0394 | 0.0085 |

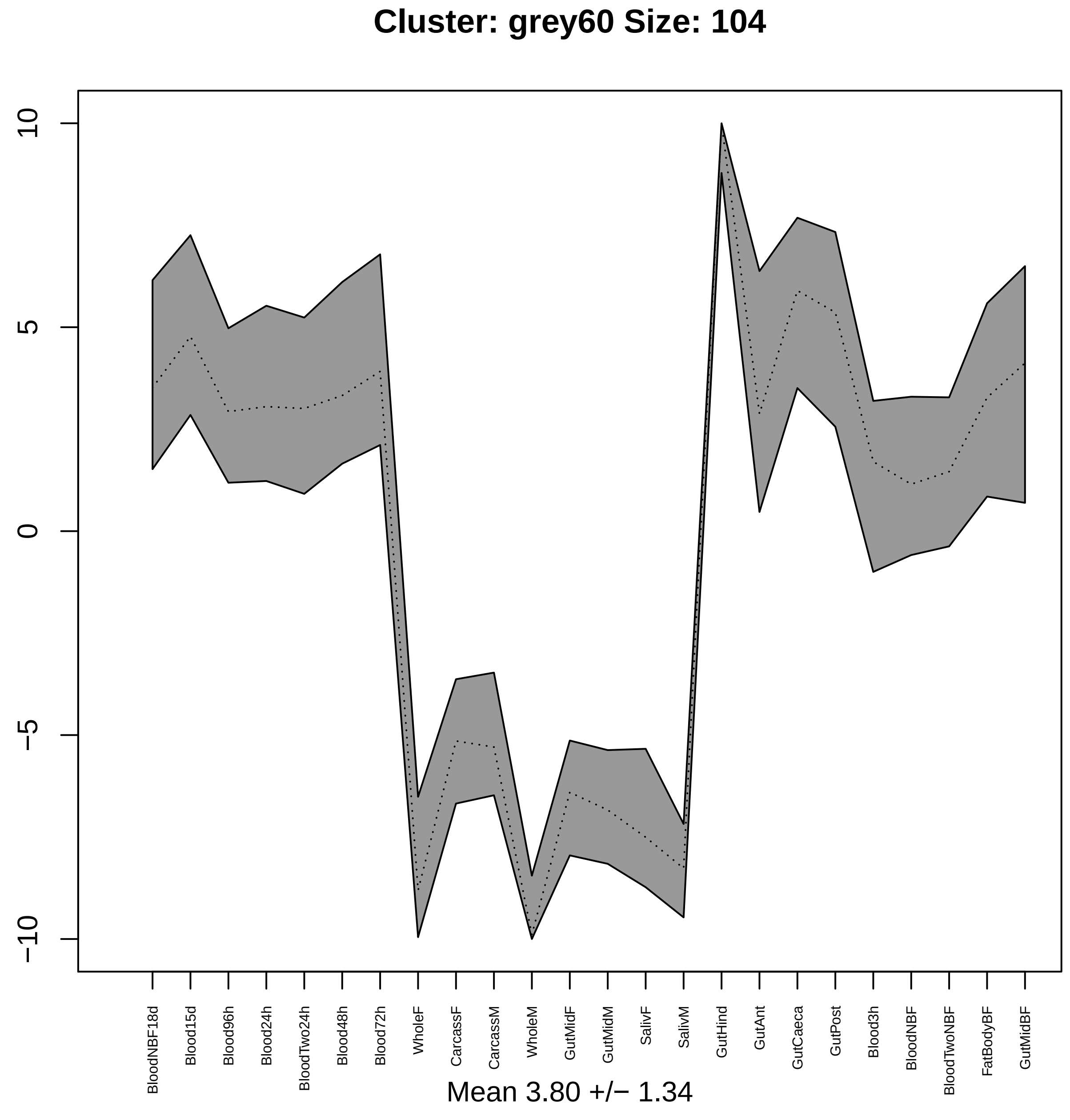

Cluster: grey60 Size: 104
10
5
0
−5
−10
BloodNBF18d
Blood15d
Blood96h
Blood24h
BloodTwo24h
Blood48h
Blood72h
WholeF
CarcassF
CarcassM
WholeM
GutMidF
GutMidM
SalivF
SalivM
GutHind
GutAnt
GutCaeca
GutPost
Blood3h
BloodNBF
BloodTwoNBF
FatBodyBF
GutMidBF
Mean 3.80 +/− 1.34

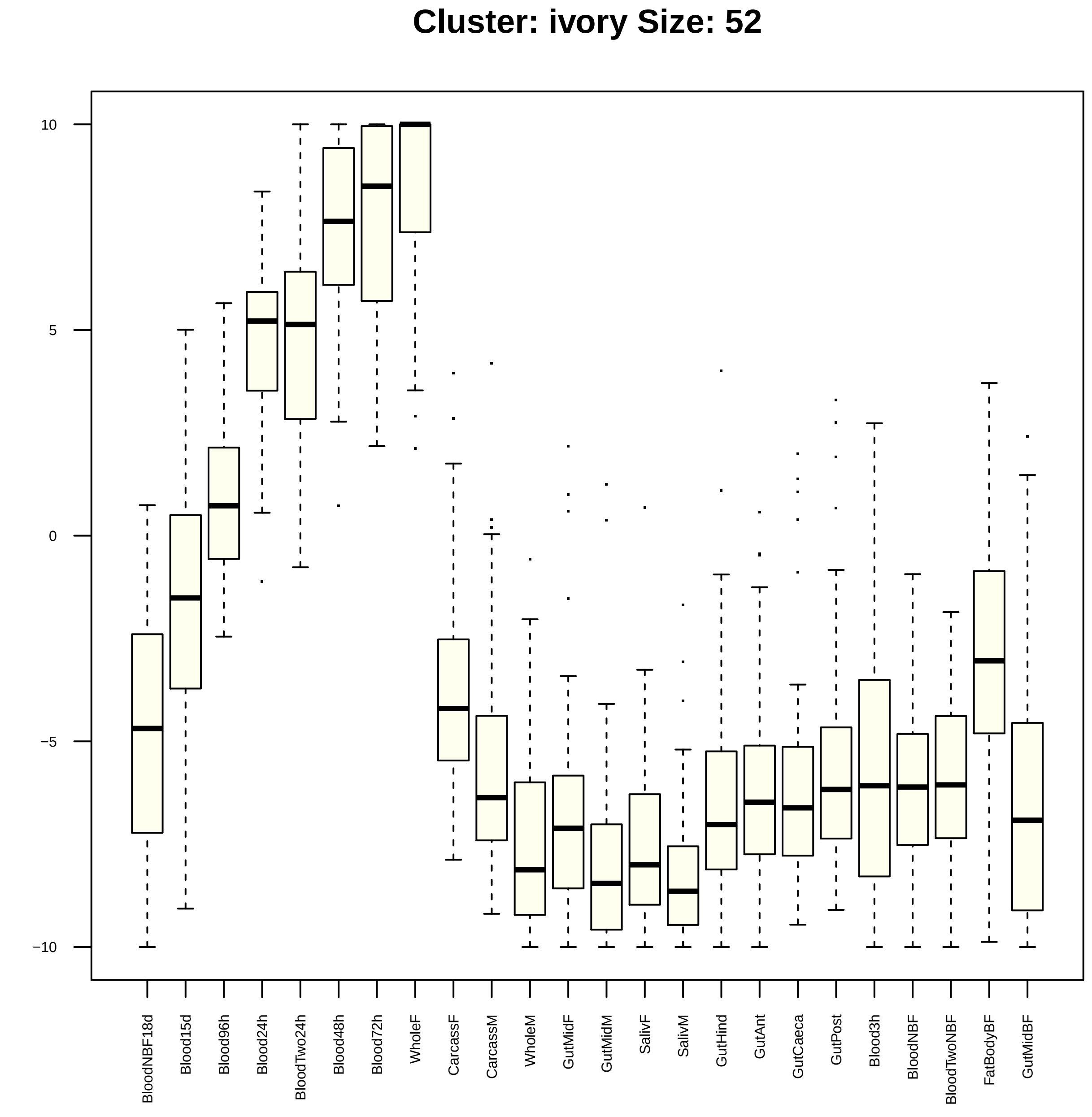

Cluster: ivory Size: 52
10
5
0
−5
−10
BloodNBF18d
Blood15d
Blood96h
Blood24h
BloodTwo24h
Blood48h
Blood72h
WholeF
CarcassF
CarcassM
WholeM
GutMidF
GutMidM
SalivF
SalivM
GutHind
GutAnt
GutCaeca
GutPost
Blood3h
BloodNBF
BloodTwoNBF
FatBodyBF
GutMidBF

| | GO.ID | BPCluster: ivory Size: 52 | Annotated | Significant | Expected | Rank in ClassicF | Weight01F | ClassicF |
|---|---|---|---|---|---|---|---|---|
| 8 | GO:2000112 | regulation of cellular macromolecule bio... | 560 | 7 | 2.81 | 156 | 0.00012 | 0.01878 |
| 9 | GO:0006261 | DNA−dependent DNA replication | 52 | 11 | 0.26 | 3 | 0.00013 | 3.9e−16 |
| 10 | GO:0007088 | regulation of mitotic nuclear division | 31 | 3 | 0.16 | 84 | 0.00047 | 0.00047 |
| 11 | GO:0042023 | DNA endoreduplication | 12 | 2 | 0.06 | 92 | 0.00156 | 0.00156 |
| 12 | GO:0007052 | mitotic spindle organization | 114 | 4 | 0.57 | 99 | 0.00237 | 0.00237 |
| 13 | GO:0007131 | reciprocal meiotic recombination | 15 | 2 | 0.08 | 101 | 0.00246 | 0.00246 |
| 14 | GO:0007099 | centriole replication | 15 | 2 | 0.08 | 102 | 0.00246 | 0.00246 |
| 15 | GO:0051276 | chromosome organization | 251 | 9 | 1.26 | 40 | 0.00260 | 2.5e−06 |
| 16 | GO:0006260 | DNA replication | 77 | 13 | 0.39 | 2 | 0.00301 | 1.2e−17 |
| 17 | GO:0045448 | mitotic cell cycle, embryonic | 17 | 2 | 0.09 | 109 | 0.00317 | 0.00317 |
| 18 | GO:0031570 | DNA integrity checkpoint | 54 | 4 | 0.27 | 73 | 0.00459 | 0.00014 |
| 19 | GO:0043065 | positive regulation of apoptotic process | 21 | 2 | 0.11 | 117 | 0.00483 | 0.00483 |
| 20 | GO:0008284 | positive regulation of cell proliferatio... | 23 | 2 | 0.12 | 122 | 0.00578 | 0.00578 |
| 21 | GO:0006342 | chromatin silencing | 26 | 2 | 0.13 | 127 | 0.00736 | 0.00736 |
| 22 | GO:0006277 | DNA amplification | 15 | 6 | 0.08 | 6 | 0.00847 | 4.7e−11 |
| 23 | GO:0051783 | regulation of nuclear division | 33 | 4 | 0.17 | 53 | 0.00912 | 1.9e−05 |
| 24 | GO:0006974 | cellular response to DNA damage stimulus | 168 | 10 | 0.84 | 10 | 0.00961 | 4.8e−09 |
| 25 | GO:0019730 | antimicrobial humoral response | 30 | 2 | 0.15 | 137 | 0.00972 | 0.00972 |
| 26 | GO:0051298 | centrosome duplication | 51 | 4 | 0.26 | 72 | 0.01226 | 0.00011 |
| 27 | GO:0045930 | negative regulation of mitotic cell cycl... | 67 | 4 | 0.34 | 79 | 0.01372 | 0.00032 |
| 28 | GO:0007259 | JAK−STAT cascade | 22 | 2 | 0.11 | 119 | 0.01456 | 0.00529 |
| 29 | GO:0044773 | mitotic DNA damage checkpoint | 47 | 3 | 0.24 | 93 | 0.01882 | 0.00160 |
| 30 | GO:0000086 | G2/M transition of mitotic cell cycle | 52 | 3 | 0.26 | 97 | 0.01883 | 0.00215 |

| | GO.ID | MFCluster: ivory Size: 52 | Annotated | Significant | Expected | Rank in ClassicF | Weight01F | ClassicF |
|---|---|---|---|---|---|---|---|---|
| *4* | GO:0005524 | ATP binding | 592 | 10 | 3.04 | 14 | 0.00059 | 0.00059 |
| *5* | GO:0003887 | DNA−directed DNA polymerase activity | 16 | 2 | 0.08 | 23 | 0.00294 | 0.00294 |
| *6* | GO:0016796 | exonuclease activity, active with either... | 18 | 2 | 0.09 | 28 | 0.00372 | 0.00372 |
| *7* | GO:0004536 | deoxyribonuclease activity | 19 | 2 | 0.10 | 29 | 0.00415 | 0.00415 |
| *9* | GO:0016462 | pyrophosphatase activity | 439 | 9 | 2.25 | 10 | 0.03787 | 0.00029 |
| *14* | GO:0000166 | nucleotide binding | 998 | 13 | 5.12 | 17 | 0.08237 | 0.00090 |
| *22* | GO:0017111 | nucleoside−triphosphatase activity | 430 | 8 | 2.21 | 19 | 0.17815 | 0.00125 |
| *26* | GO:0004386 | helicase activity | 103 | 5 | 0.53 | 9 | 0.28023 | 0.00016 |

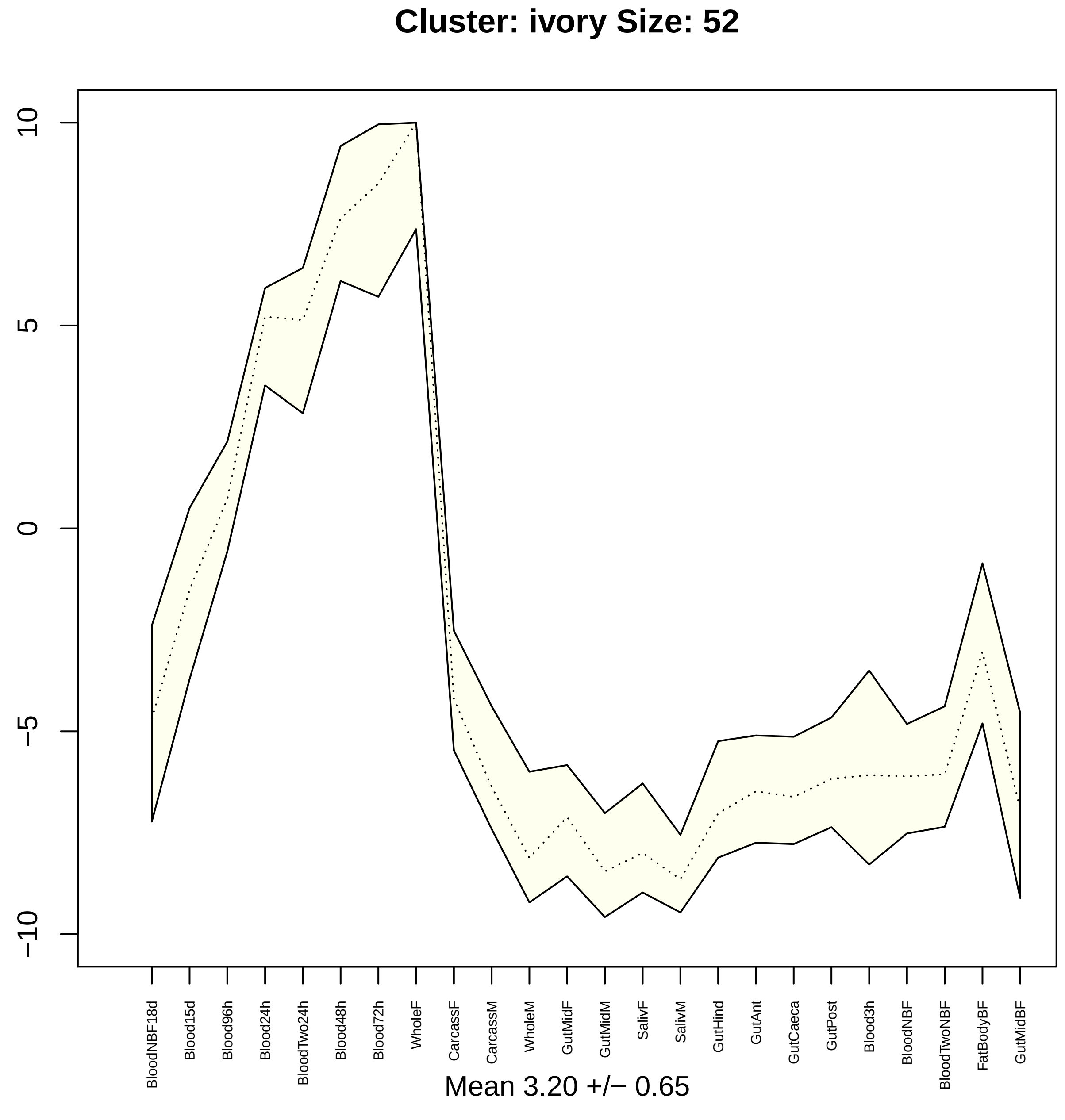
Cluster: ivory Size: 52
10
5
0
−5
−10
BloodNBF18d
Blood15d
Blood96h
Blood24h
BloodTwo24h
Blood48h
Blood72h
WholeF
CarcassF
CarcassM
WholeM
GutMidF
GutMidM
SalivF
SalivM
GutHind
GutAnt
GutCaeca
GutPost
Blood3h
BloodNBF
BloodTwoNBF
FatBodyBF
GutMidBF
Mean 3.20 +/− 0.65

# Cluster: honeydew Size: 22

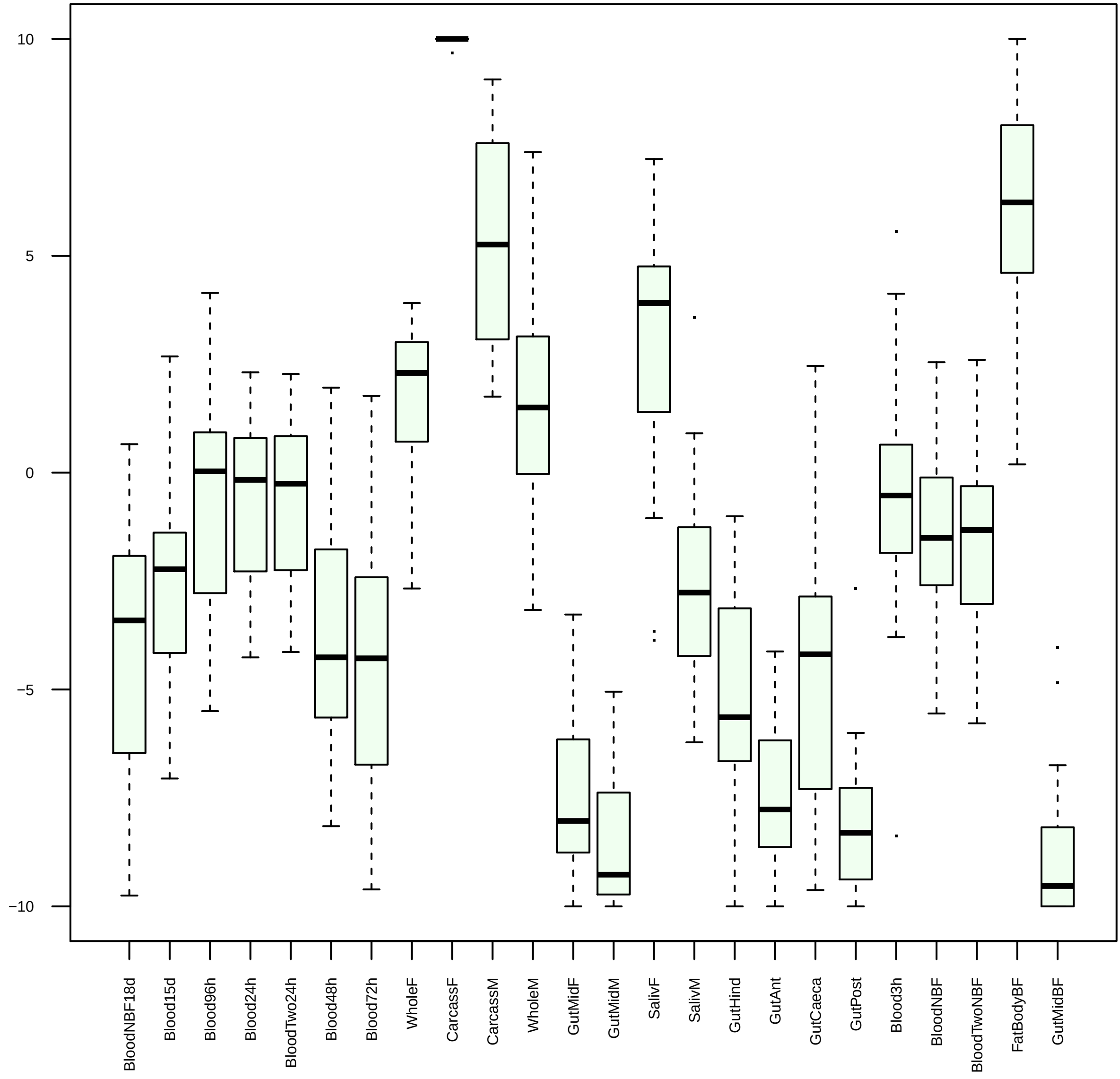

| | GO.ID | BPCluster: honeydew Size: 22 | Annotated | Significant | Expected | Rank in ClassicF | Weight01F | ClassicF |
|---|---|---|---|---|---|---|---|---|
| *2* | GO:0042135 | neurotransmitter catabolic process | 12 | 2 | 0.03 | 6 | 0.00043 | 0.00043 |
| *3* | GO:0009072 | aromatic amino acid family metabolic pro... | 14 | 2 | 0.04 | 11 | 0.00060 | 0.00060 |
| *4* | GO:0042737 | drug catabolic process | 48 | 3 | 0.13 | 5 | 0.00159 | 0.00025 |
| *5* | GO:1901606 | alpha−amino acid catabolic process | 27 | 2 | 0.07 | 15 | 0.00226 | 0.00226 |
| *6* | GO:0006807 | nitrogen compound metabolic process | 2967 | 13 | 7.91 | 23 | 0.00876 | 0.01199 |

| | GO.ID | MFCluster: honeydew Size: 22 | Annotated | Significant | Expected | Rank in ClassicF | Weight01F | ClassicF |
|---|---|---|---|---|---|---|---|---|
| 1 | GO:0016741 | transferase activity, transferring one−c... | 92 | 3 | 0.22 | 1 | 0.0084 | 0.0012 |

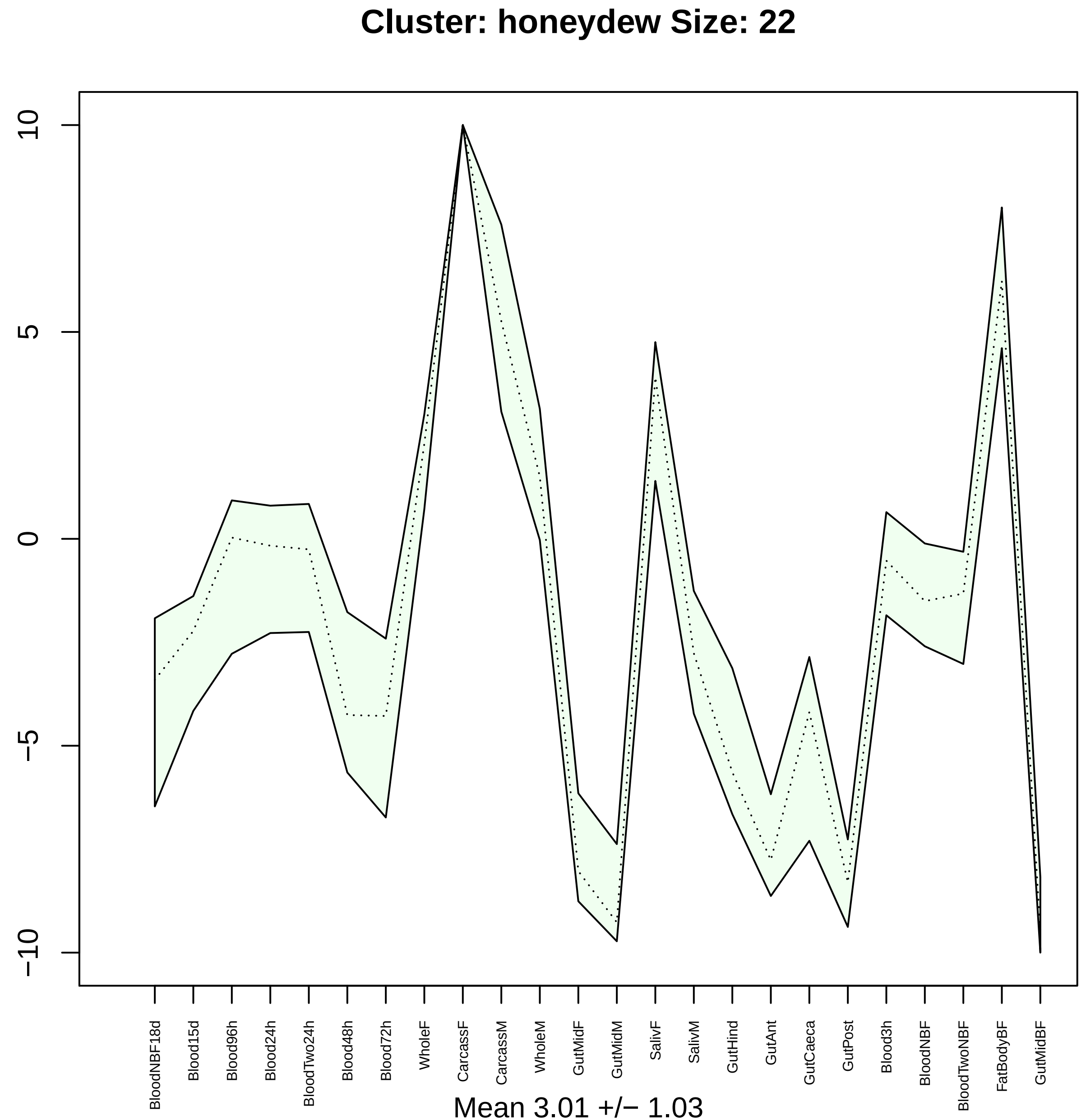

Cluster: honeydew Size: 22
10
5
0
−5
−10
BloodNBF18d
Blood15d
Blood96h
Blood24h
BloodTwo24h
Blood48h
Blood72h
WholeF
CarcassF
CarcassM
WholeM
GutMidF
GutMidM
SalivF
SalivM
GutHind
GutAnt
GutCaeca
GutPost
Blood3h
BloodNBF
BloodTwoNBF
FatBodyBF
GutMidBF
Mean 3.01 +/− 1.03

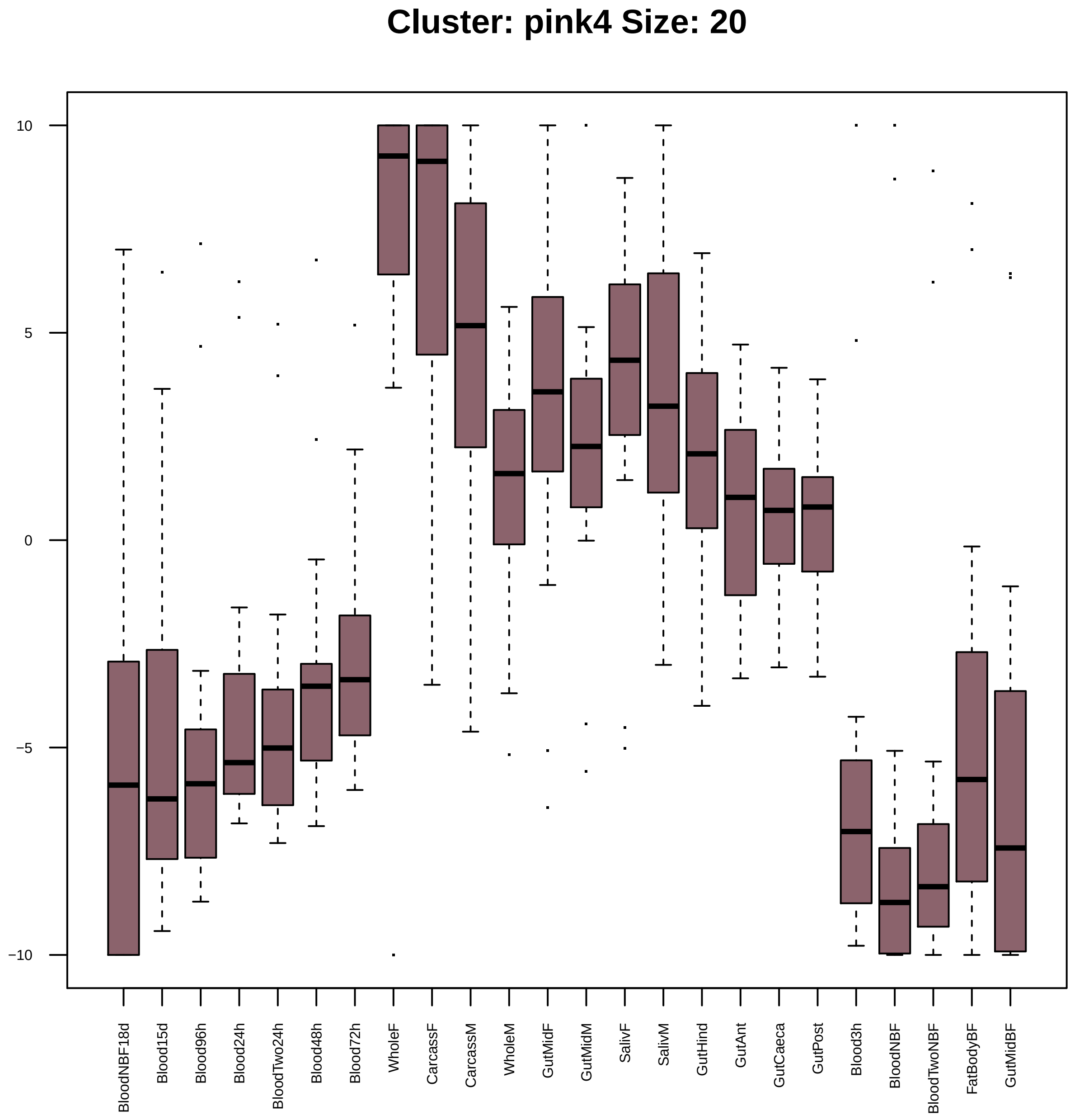
Cluster: pink4 Size: 20
10
5
0
−5
−10
BloodNBF18d
Blood15d
Blood96h
Blood24h
BloodTwo24h
Blood48h
Blood72h
WholeF
CarcassF
CarcassM
WholeM
GutMidF
GutMidM
SalivF
SalivM
GutHind
GutAnt
GutCaeca
GutPost
Blood3h
BloodNBF
BloodTwoNBF
FatBodyBF
GutMidBF

| | GO.ID | BPCluster: pink4 Size: 20 | Annotated | Significant | Expected | Rank in ClassicF | Weight01F | ClassicF |
|---|---|---|---|---|---|---|---|---|
| 13 | GO:0051298 | centrosome duplication | 51 | 2 | 0.14 | 2 | 0.087 | 0.0079 |

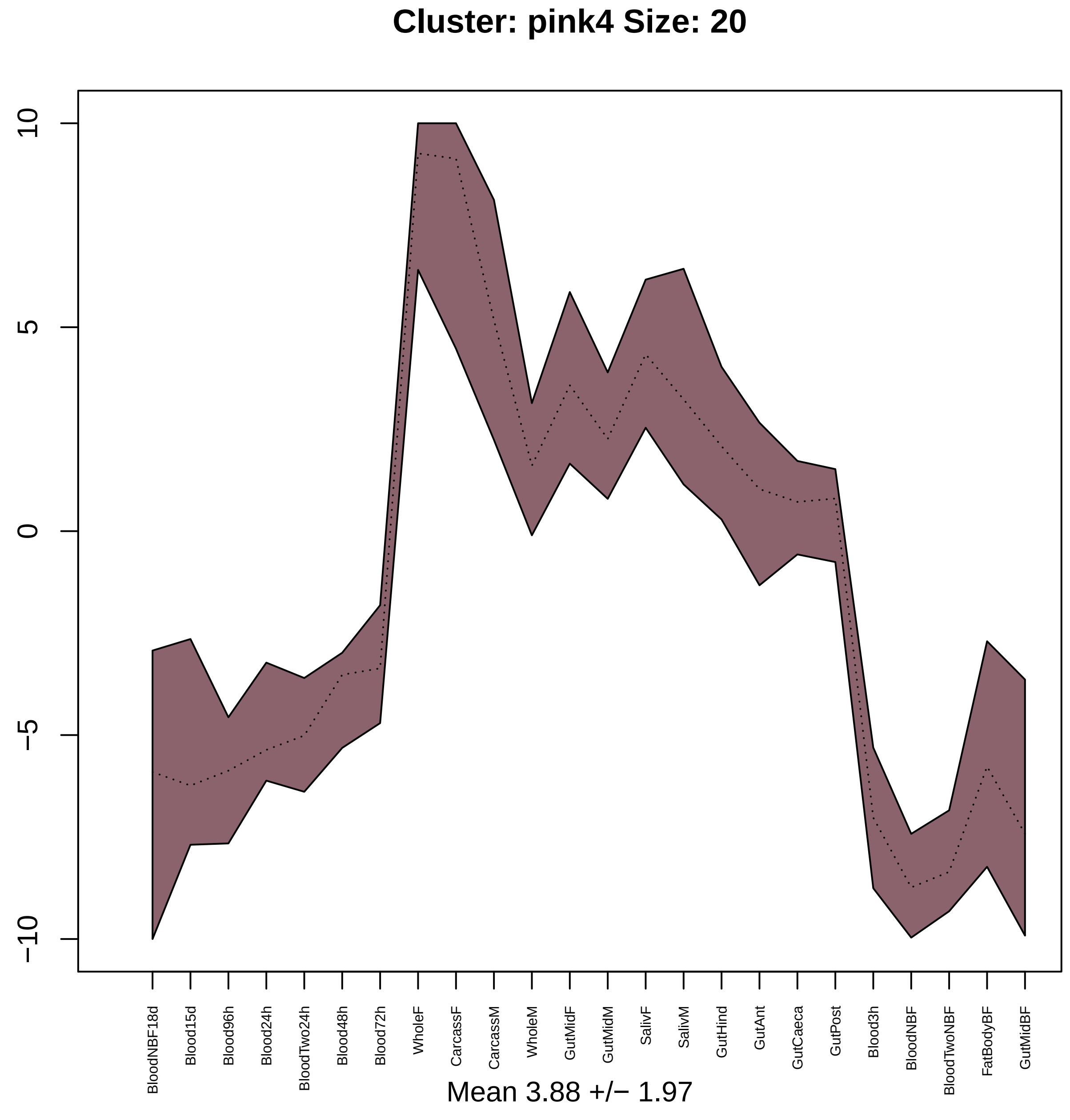

Cluster: pink4 Size: 20
10
5
0
−5
−10
BloodNBF18d
Blood15d
Blood96h
Blood24h
BloodTwo24h
Blood48h
Blood72h
WholeF
CarcassF
CarcassM
WholeM
GutMidF
GutMidM
SalivF
SalivM
GutHind
GutAnt
GutCaeca
GutPost
Blood3h
BloodNBF
BloodTwoNBF
FatBodyBF
GutMidBF
Mean 3.88 +/− 1.97

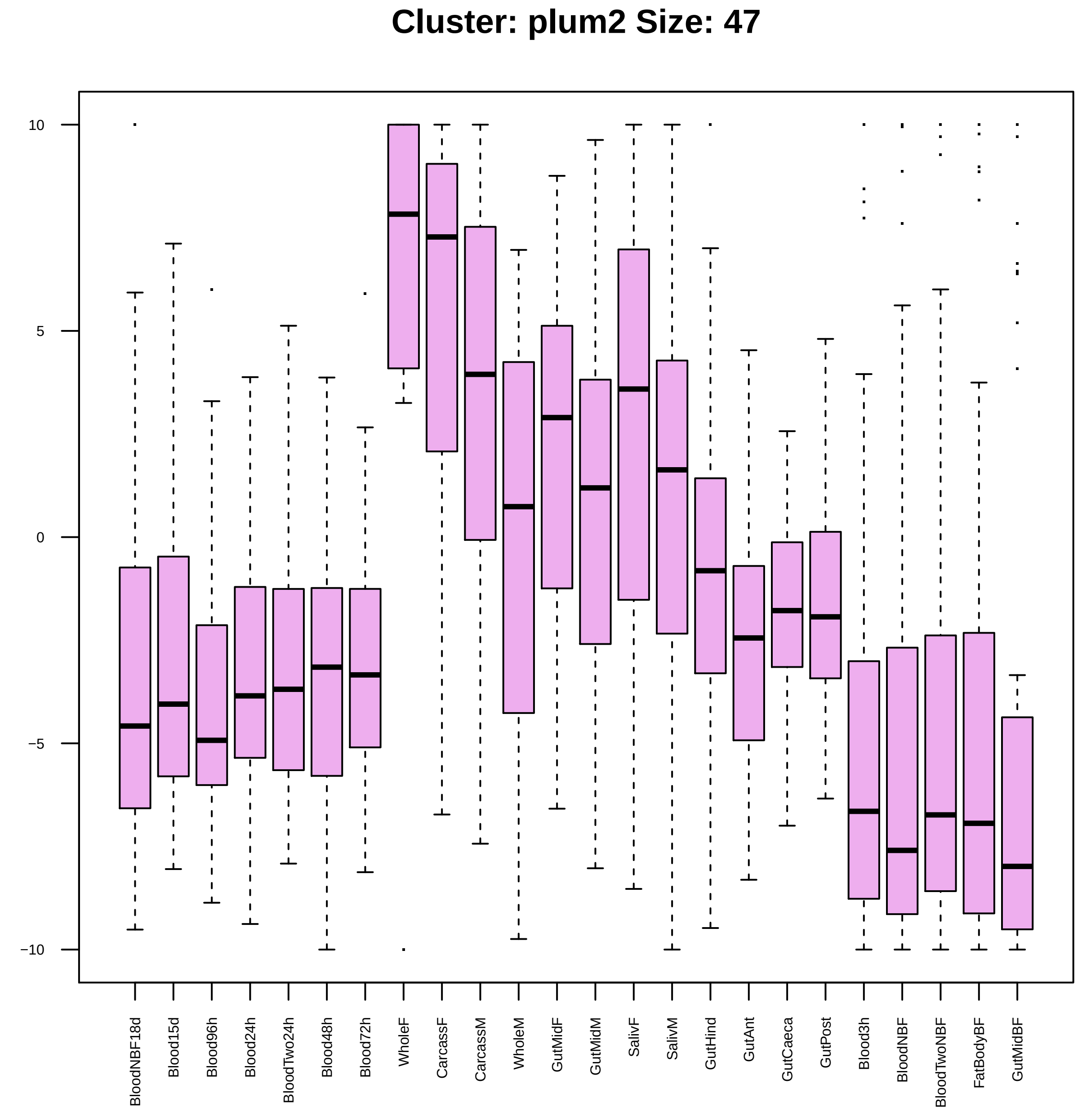

Cluster: plum2 Size: 47
10
5
0
−5
−10
BloodNBF18d
Blood15d
Blood96h
Blood24h
BloodTwo24h
Blood48h
Blood72h
WholeF
CarcassF
CarcassM
WholeM
GutMidF
GutMidM
SalivF
SalivM
GutHind
GutAnt
GutCaeca
GutPost
Blood3h
BloodNBF
BloodTwoNBF
FatBodyBF
GutMidBF

| | GO.ID | BPCluster: plum2 Size: 47 | Annotated | Significant | Expected | Rank in ClassicF | Weight01F | ClassicF |
|---|---|---|---|---|---|---|---|---|
| 2 | GO:0006479 | protein methylation | 34 | 2 | 0.13 | 3 | 0.029 | 0.0071 |

| | GO.ID | MFCluster: plum2 Size: 47 | Annotated | Significant | Expected | Rank in ClassicF | Weight01F | ClassicF |
|---|---|---|---|---|---|---|---|---|
| *1* | GO:0016417 | S−acyltransferase activity | 11 | 2 | 0.05 | 1 | 0.0012 | 0.0012 |
| *3* | GO:0008276 | protein methyltransferase activity | 25 | 2 | 0.12 | 3 | 0.0372 | 0.0064 |
| *9* | GO:0008170 | N−methyltransferase activity | 25 | 2 | 0.12 | 4 | 0.0508 | 0.0064 |

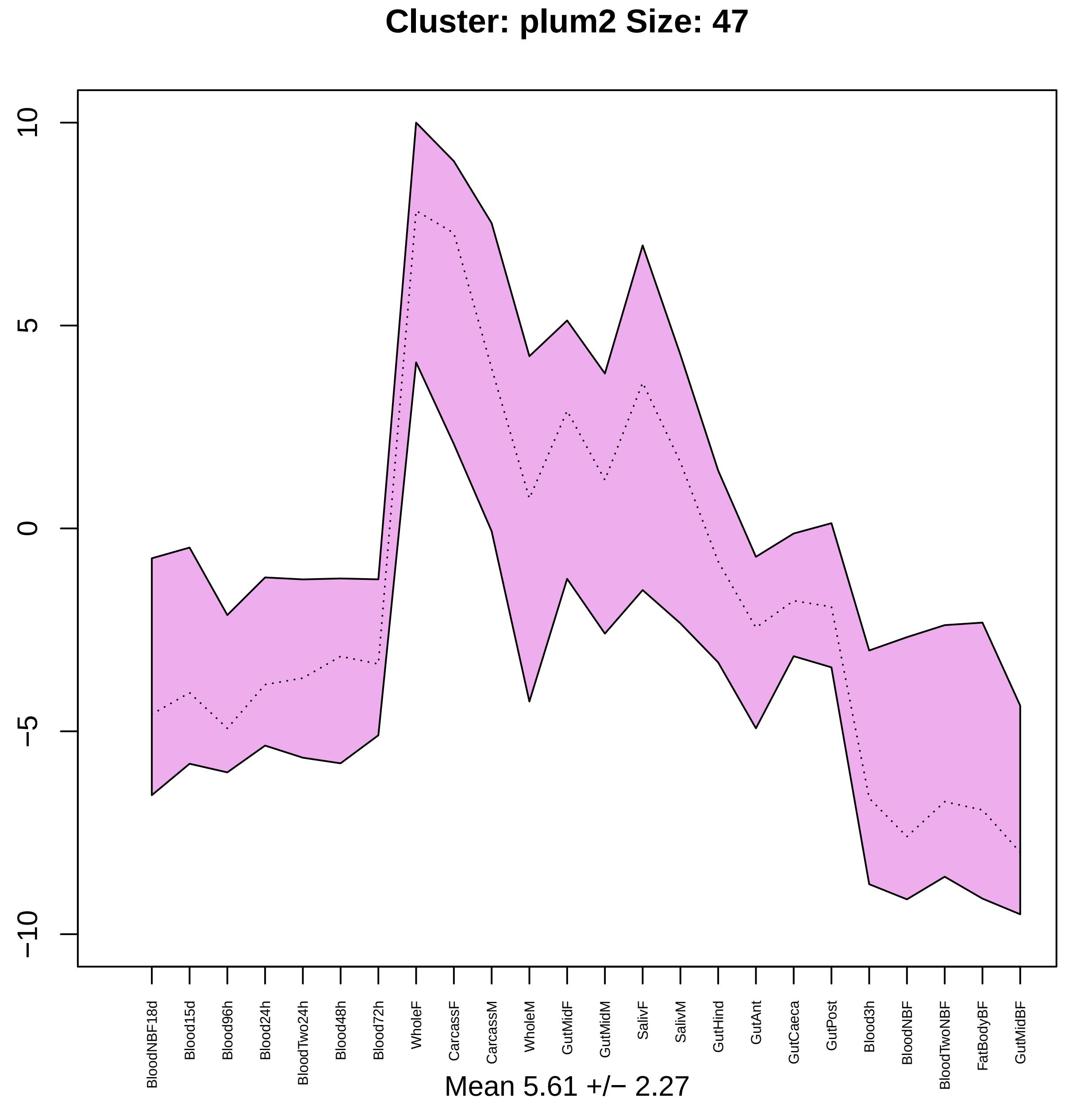

Cluster: plum2 Size: 47
10
5
0
−5
−10
BloodNBF18d
Blood15d
Blood96h
Blood24h
BloodTwo24h
Blood48h
Blood72h
WholeF
CarcassF
CarcassM
WholeM
GutMidF
GutMidM
SalivF
SalivM
GutHind
GutAnt
GutCaeca
GutPost
Blood3h
BloodNBF
BloodTwoNBF
FatBodyBF
GutMidBF
Mean 5.61 +/− 2.27

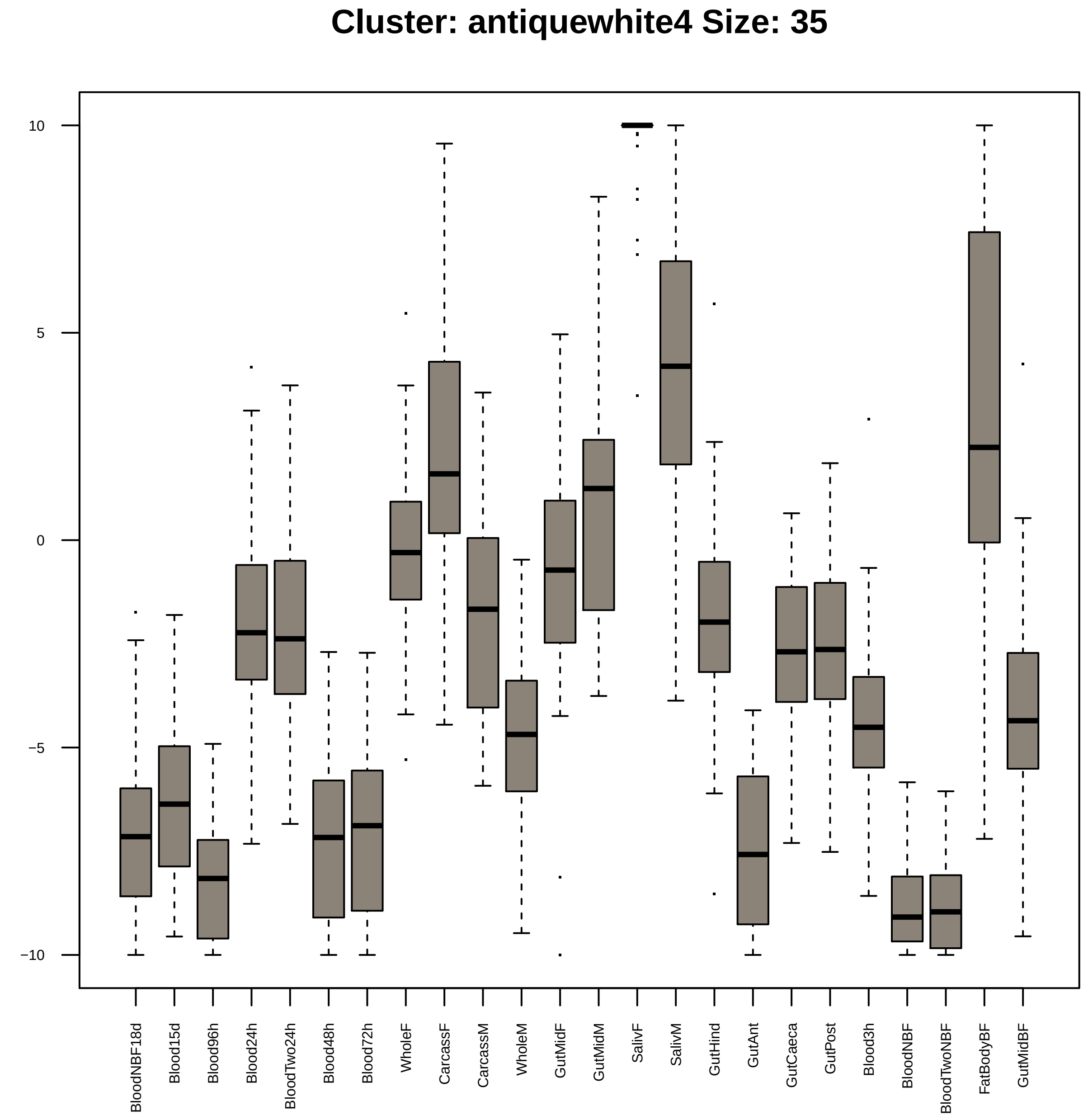
Cluster: antiquewhite4 Size: 35
10
5
0
−5
−10
BloodNBF18d
Blood15d
Blood96h
Blood24h
BloodTwo24h
Blood48h
Blood72h
WholeF
CarcassF
CarcassM
WholeM
GutMidF
GutMidM
SalivF
SalivM
GutHind
GutAnt
GutCaeca
GutPost
Blood3h
BloodNBF
BloodTwoNBF
FatBodyBF
GutMidBF

| | GO.ID | BPCluster: antiquewhite4 Size: 35 | Annotated | Significant | Expected | Rank in ClassicF | Weight01F | ClassicF |
|---|---|---|---|---|---|---|---|---|
| *3* | GO:0006662 | glycerol ether metabolic process | 11 | 2 | 0.05 | 10 | 0.0011 | 0.0011 |
| *4* | GO:0048193 | Golgi vesicle transport | 54 | 3 | 0.25 | 15 | 0.0018 | 0.0018 |
| *5* | GO:0045047 | protein targeting to ER | 11 | 2 | 0.05 | 11 | 0.0044 | 0.0011 |
| *6* | GO:0016485 | protein processing | 24 | 2 | 0.11 | 25 | 0.0052 | 0.0052 |
| *7* | GO:0070972 | protein localization to endoplasmic reti... | 13 | 3 | 0.06 | 8 | 0.0085 | 2.3e−05 |
| *10* | GO:0006612 | protein targeting to membrane | 14 | 2 | 0.06 | 14 | 0.0131 | 0.0018 |
| *16* | GO:0061024 | membrane organization | 94 | 3 | 0.43 | 29 | 0.0467 | 0.0086 |
| *28* | GO:0006886 | intracellular protein transport | 167 | 4 | 0.76 | 27 | 0.0920 | 0.0065 |

|  | GO.ID | MFCluster: antiquewhite4 Size: 35 | Annotated | Significant | Expected | Rank in ClassicF | Weight01F | ClassicF |
|---|---|---|---|---|---|---|---|---|
| 2 | GO:0008194 | UDP−glycosyltransferase activity | 30 | 3 | 0.12 | 4 | 0.00020 | 0.00020 |
| 3 | GO:0005048 | signal sequence binding | 11 | 2 | 0.04 | 5 | 0.00078 | 0.00078 |
| 4 | GO:0043021 | ribonucleoprotein complex binding | 14 | 2 | 0.05 | 6 | 0.00129 | 0.00129 |
| 5 | GO:0015035 | protein disulfide oxidoreductase activit... | 14 | 2 | 0.05 | 7 | 0.00129 | 0.00129 |
| 6 | GO:0016860 | intramolecular oxidoreductase activity | 23 | 2 | 0.09 | 10 | 0.00350 | 0.00350 |

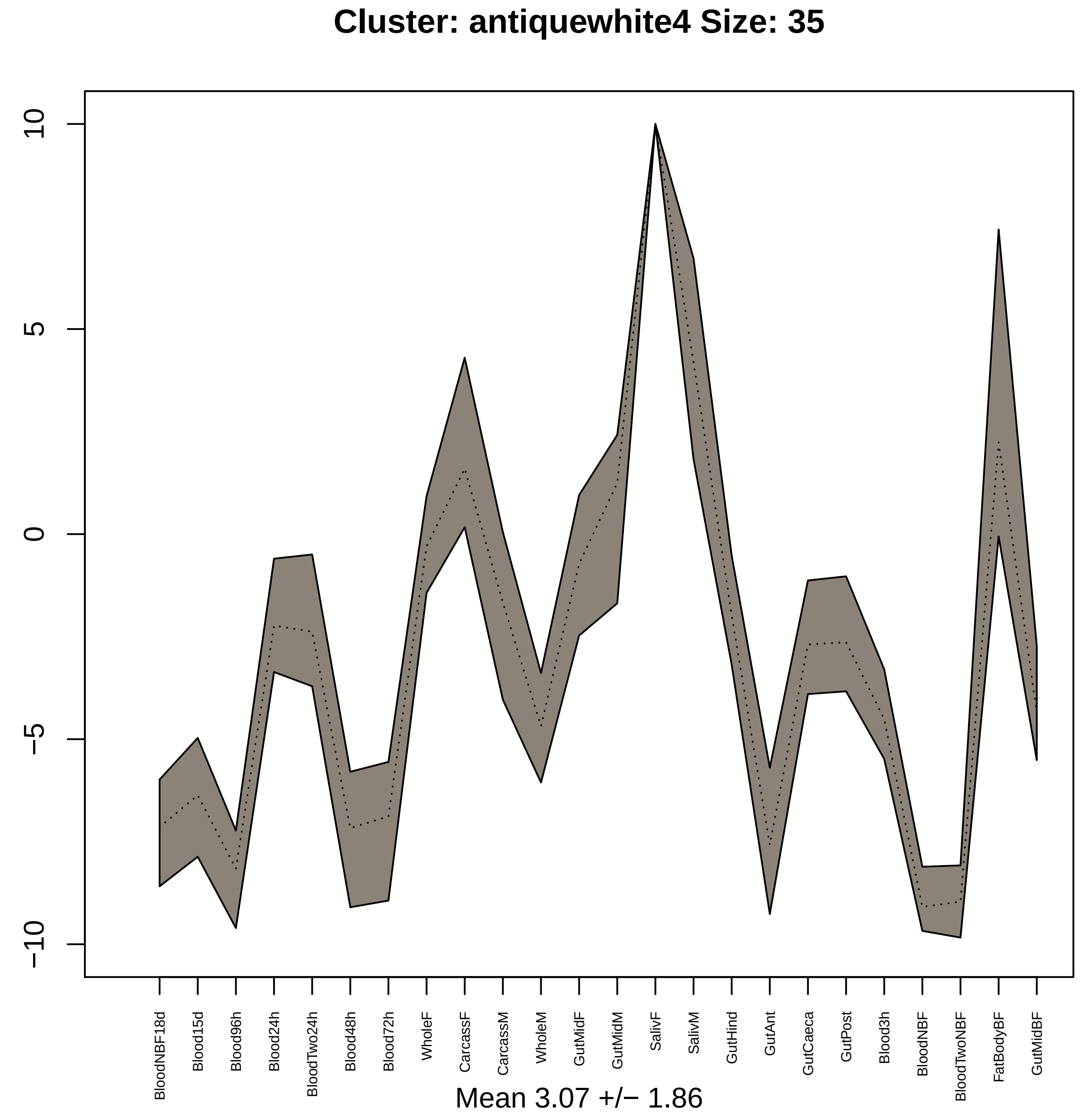

Cluster: antiquewhite4 Size: 35
10
5
0
−5
−10
BloodNBF18d
Blood15d
Blood96h
Blood24h
BloodTwo24h
Blood48h
Blood72h
WholeF
CarcassF
CarcassM
WholeM
GutMidF
GutMidM
SalivF
SalivM
GutHind
GutAnt
GutCaeca
GutPost
Blood3h
BloodNBF
BloodTwoNBF
FatBodyBF
GutMidBF
Mean 3.07 +/− 1.86

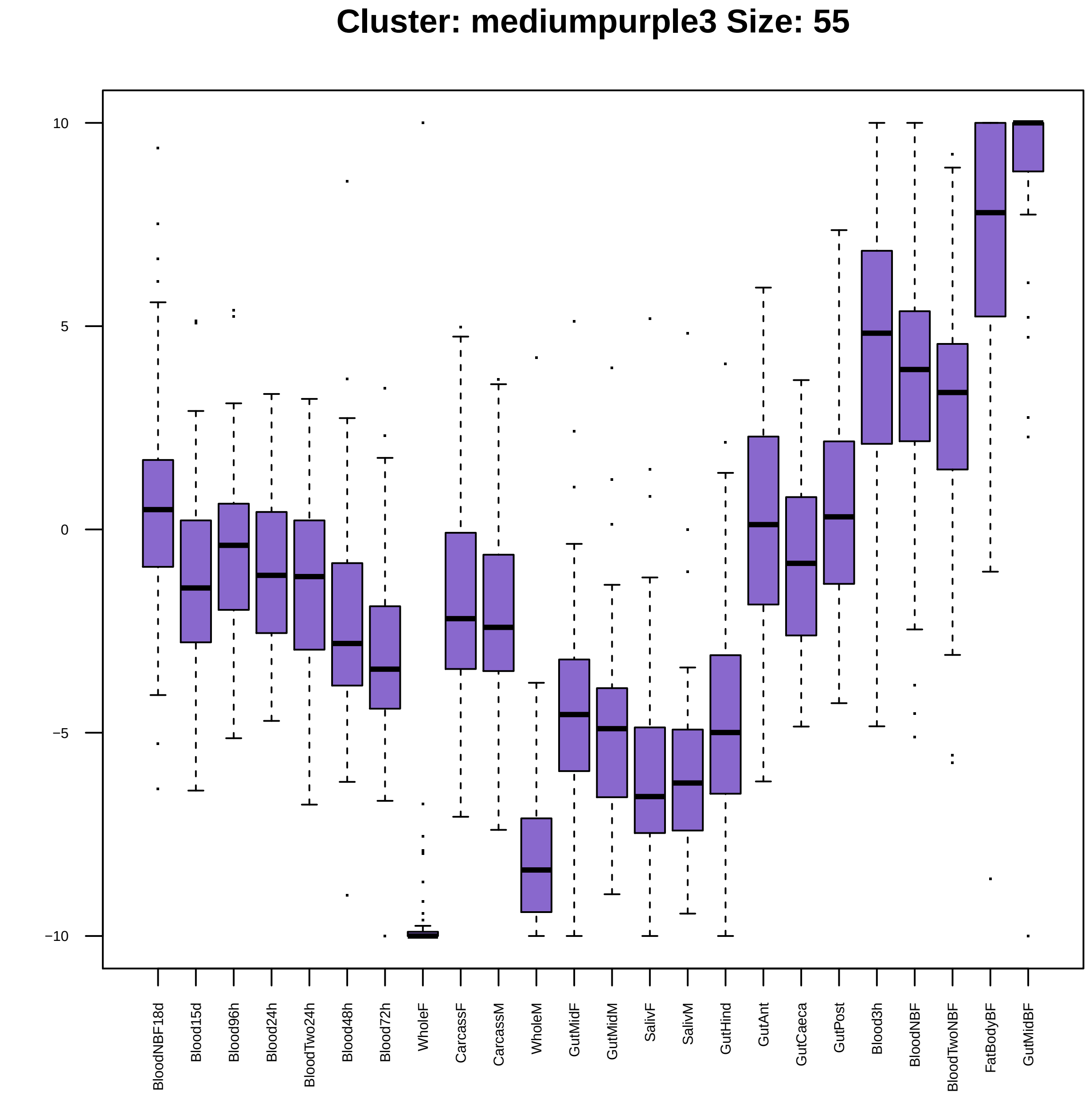
Cluster: mediumpurple3 Size: 55
10
5
0
−5
−10
BloodNBF18d
Blood15d
Blood96h
Blood24h
BloodTwo24h
Blood48h
Blood72h
WholeF
CarcassF
CarcassM
WholeM
GutMidF
GutMidM
SalivF
SalivM
GutHind
GutAnt
GutCaeca
GutPost
Blood3h
BloodNBF
BloodTwoNBF
FatBodyBF
GutMidBF

| | GO.ID | BPCluster: mediumpurple3 Size: 55 | Annotated | Significant | Expected | Rank in ClassicF | Weight01F | ClassicF |
|---|---|---|---|---|---|---|---|---|
| *4* | GO:0050909 | sensory perception of taste | 56 | 3 | 0.32 | 21 | 0.024 | 0.0037 |

| | GO.ID | MFCluster: mediumpurple3 Size: 55 | Annotated | Significant | Expected | Rank in ClassicF | Weight01F | ClassicF |
|---|---|---|---|---|---|---|---|---|
| *3* | GO:0005549 | odorant binding | 118 | 5 | 0.65 | 6 | 0.00045 | 0.00045 |
| *4* | GO:0005272 | sodium channel activity | 26 | 2 | 0.14 | 12 | 0.00897 | 0.00897 |

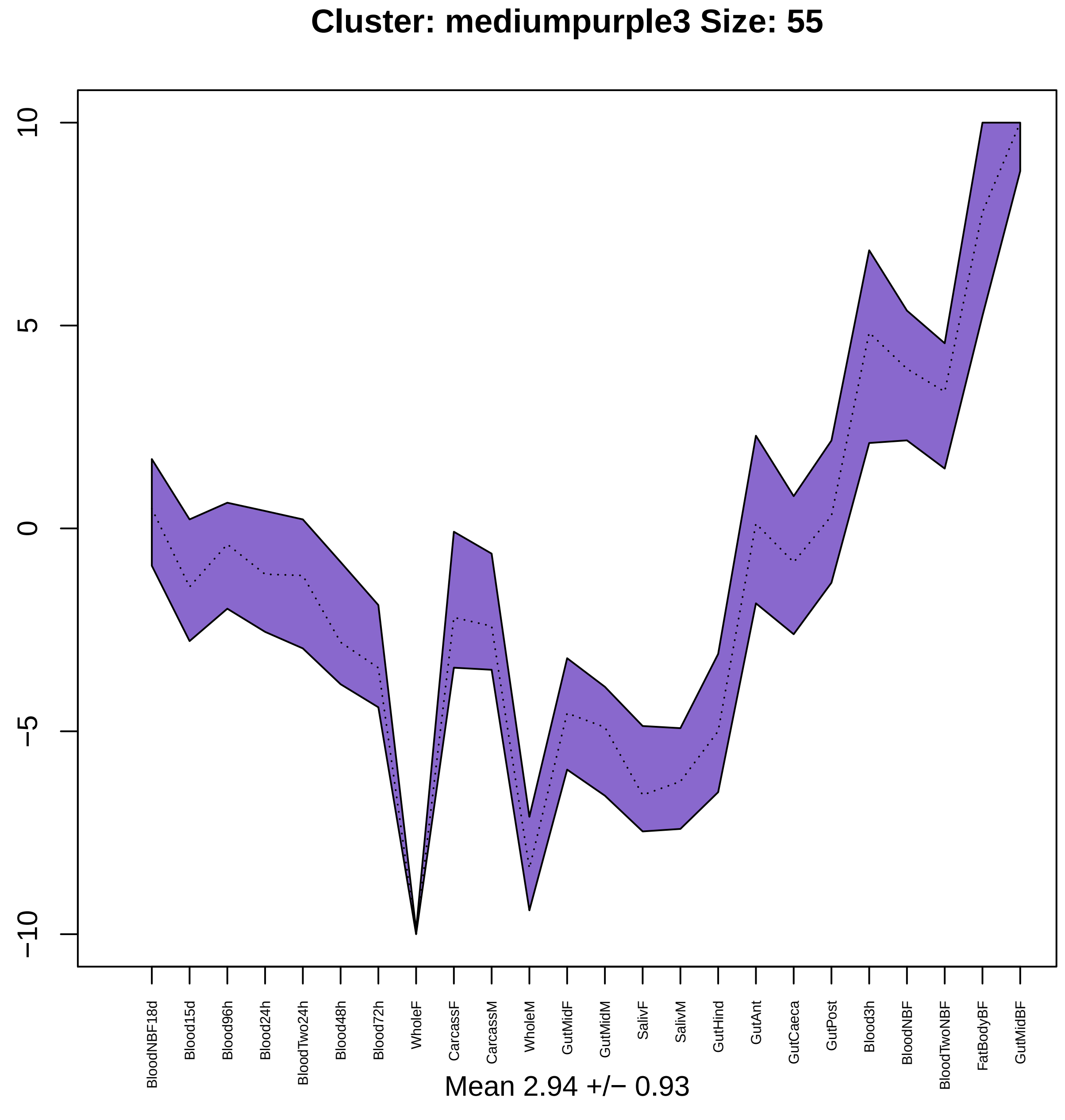

Cluster: mediumpurple3 Size: 55
10
5
0
−5
−10
BloodNBF18d
Blood15d
Blood96h
Blood24h
BloodTwo24h
Blood48h
Blood72h
WholeF
CarcassF
CarcassM
WholeM
GutMidF
GutMidM
SalivF
SalivM
GutHind
GutAnt
GutCaeca
GutPost
Blood3h
BloodNBF
BloodTwoNBF
FatBodyBF
GutMidBF
Mean 2.94 +/− 0.93

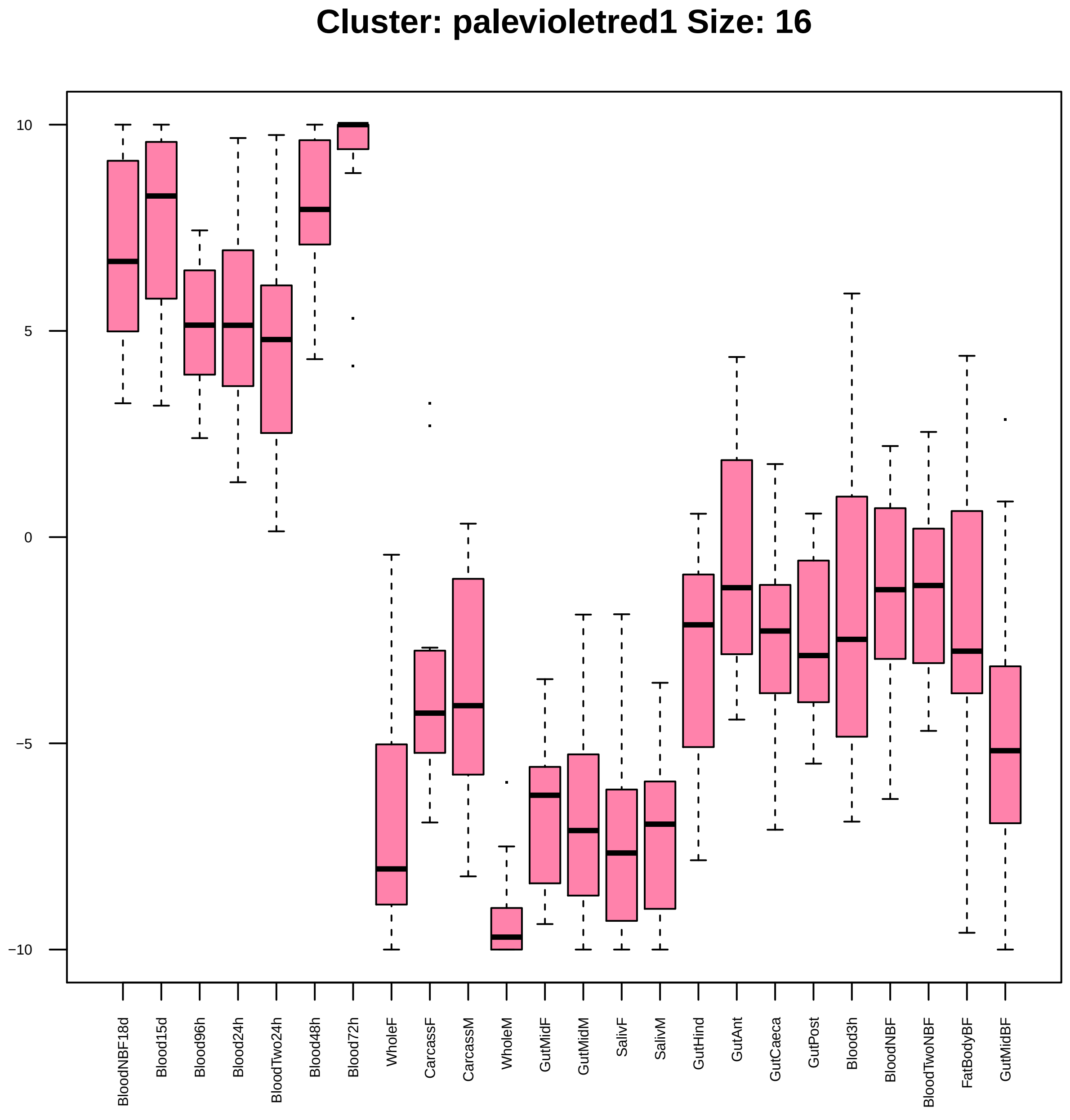
Cluster: palevioletred1 Size: 16
10
5
0
−5
−10
BloodNBF18d
Blood15d
Blood96h
Blood24h
BloodTwo24h
Blood48h
Blood72h
WholeF
CarcassF
CarcassM
WholeM
GutMidF
GutMidM
SalivF
SalivM
GutHind
GutAnt
GutCaeca
GutPost
Blood3h
BloodNBF
BloodTwoNBF
FatBodyBF
GutMidBF

| | GO.ID | BPCluster: palevioletred1 Size: 16 | Annotated | Significant | Expected | Rank in ClassicF | Weight01F | ClassicF |
|---|---|---|---|---|---|---|---|---|
| *1* | GO:0006342 | chromatin silencing | 26 | 2 | 0.04 | 6 | 0.00057 | 0.00057 |
| *2* | GO:0016570 | histone modification | 58 | 2 | 0.08 | 18 | 0.00281 | 0.00281 |

| | GO.ID | MFCluster: palevioletred1 Size: 16 | Annotated | Significant | Expected | Rank in ClassicF | Weight01F | ClassicF |
|---|---|---|---|---|---|---|---|---|
| *1* | GO:0019904 | protein domain specific binding | 37 | 2 | 0.05 | 3 | 0.0011 | 0.00112 |
| *2* | GO:0043565 | sequence−specific DNA binding | 212 | 3 | 0.29 | 5 | 0.0026 | 0.00258 |
| *3* | GO:0003677 | DNA binding | 523 | 6 | 0.73 | 1 | 0.0027 | 2.3e−05 |
| *4* | GO:0003700 | DNA−binding transcription factor activit... | 282 | 3 | 0.39 | 6 | 0.0058 | 0.00579 |
| *18* | GO:1901363 | heterocyclic compound binding | 2046 | 7 | 2.84 | 7 | 1.0000 | 0.00756 |
| *23* | GO:0140110 | transcription regulator activity | 322 | 4 | 0.45 | 2 | 1.0000 | 0.00066 |

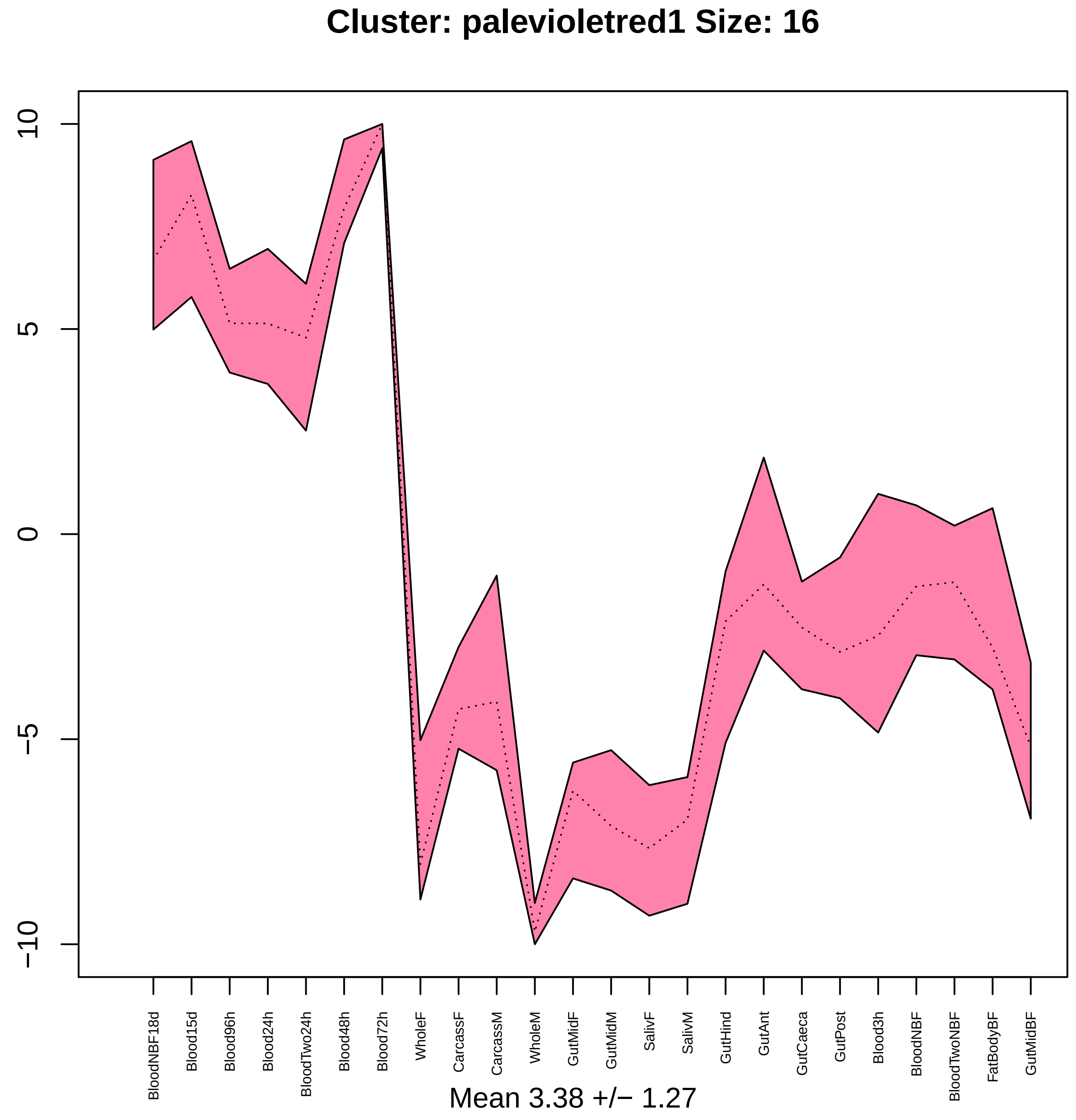

Cluster: palevioletred1 Size: 16
10
5
0
−5
−10
BloodNBF18d
Blood15d
Blood96h
Blood24h
BloodTwo24h
Blood48h
Blood72h
WholeF
CarcassF
CarcassM
WholeM
GutMidF
GutMidM
SalivF
SalivM
GutHind
GutAnt
GutCaeca
GutPost
Blood3h
BloodNBF
BloodTwoNBF
FatBodyBF
GutMidBF
Mean 3.38 +/− 1.27

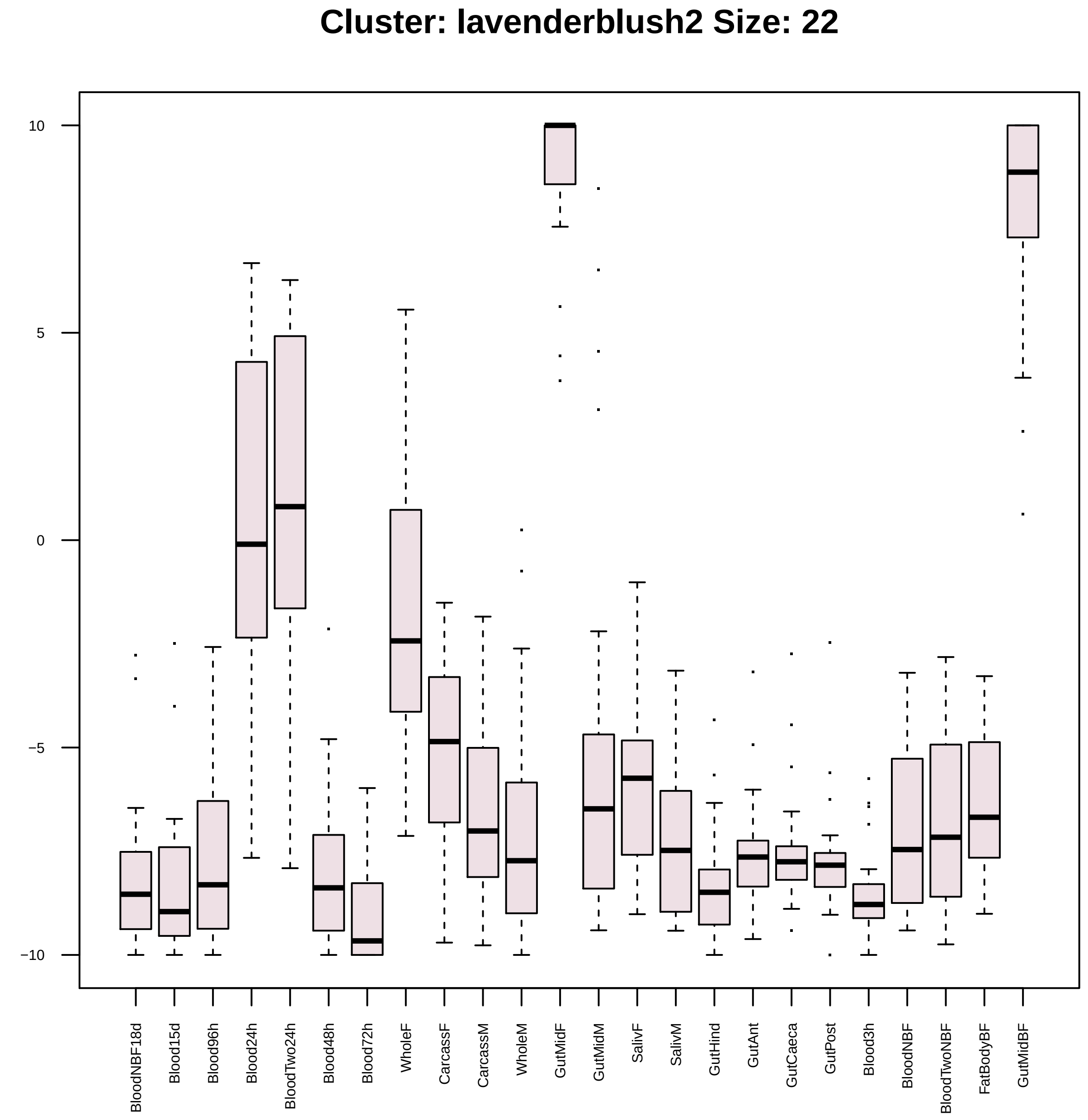

Cluster: lavenderblush2 Size: 22
10
5
0
−5
−10
BloodNBF18d
Blood15d
Blood96h
Blood24h
BloodTwo24h
Blood48h
Blood72h
WholeF
CarcassF
CarcassM
WholeM
GutMidF
GutMidM
SalivF
SalivM
GutHind
GutAnt
GutCaeca
GutPost
Blood3h
BloodNBF
BloodTwoNBF
FatBodyBF
GutMidBF

| | GO.ID | BPCluster: lavenderblush2 Size: 22 | Annotated | Significant | Expected | Rank in ClassicF | Weight01F | ClassicF |
|---|---|---|---|---|---|---|---|---|
| *7* | GO:0008152 | metabolic process | 4285 | 14 | 9.41 | 6 | 0.274 | 0.00380 |
| *19* | GO:0019538 | protein metabolic process | 1480 | 10 | 3.25 | 3 | 1.000 | 0.00017 |

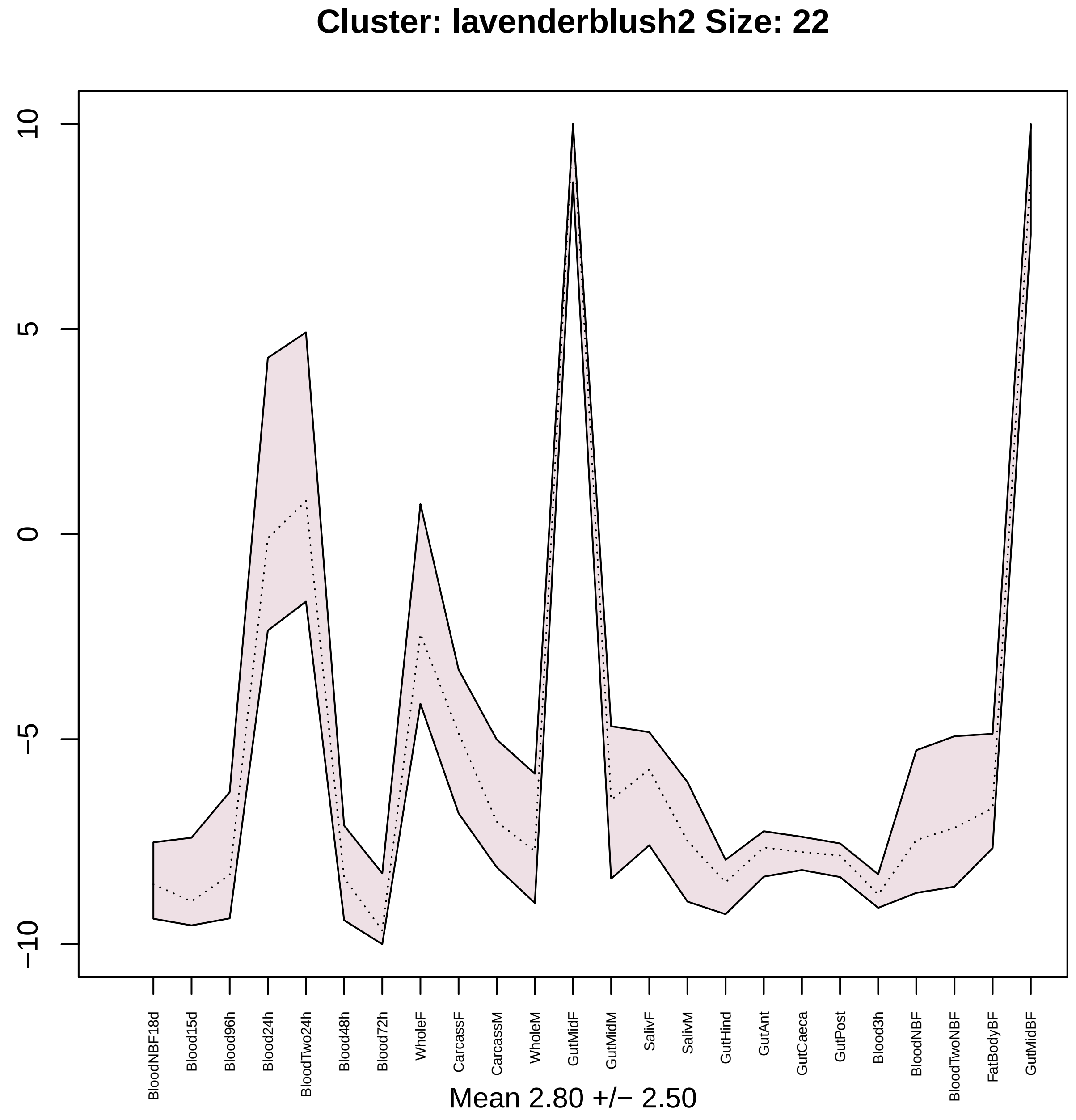

Cluster: lavenderblush2 Size: 22
10
5
0
−5
−10
BloodNBF18d
Blood15d
Blood96h
Blood24h
BloodTwo24h
Blood48h
Blood72h
WholeF
CarcassF
CarcassM
WholeM
GutMidF
GutMidM
SalivF
SalivM
GutHind
GutAnt
GutCaeca
GutPost
Blood3h
BloodNBF
BloodTwoNBF
FatBodyBF
GutMidBF
Mean 2.80 +/− 2.50

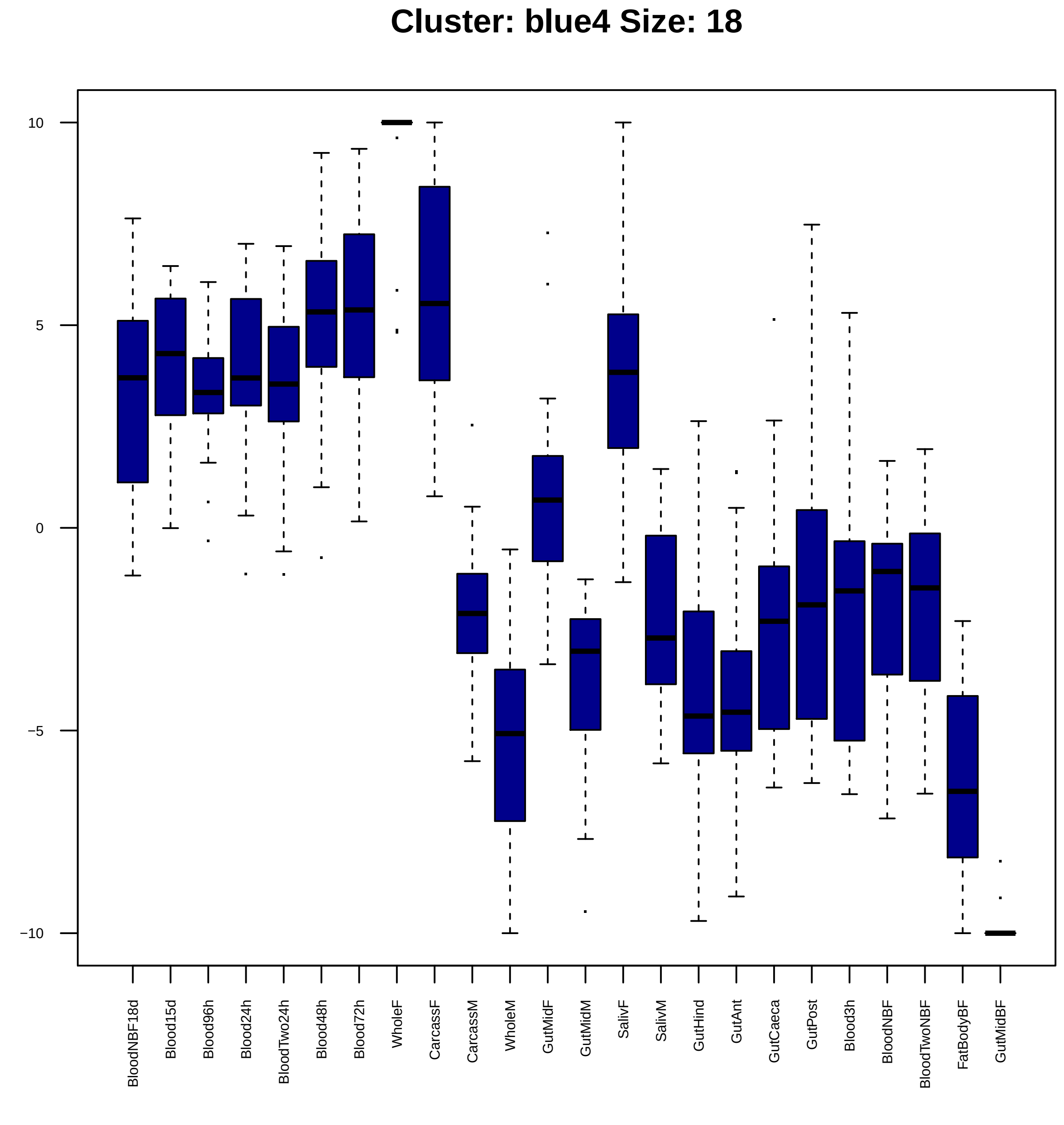
Cluster: blue4 Size: 18
10
5
0
−5
−10
BloodNBF18d
Blood15d
Blood96h
Blood24h
BloodTwo24h
Blood48h
Blood72h
WholeF
CarcassF
CarcassM
WholeM
GutMidF
GutMidM
SalivF
SalivM
GutHind
GutAnt
GutCaeca
GutPost
Blood3h
BloodNBF
BloodTwoNBF
FatBodyBF
GutMidBF

| | GO.ID | BPCluster: blue4 Size: 18 | Annotated | Significant | Expected | Rank in ClassicF | Weight01F | ClassicF |
|---|---|---|---|---|---|---|---|---|
| *1* | GO:0042254 | ribosome biogenesis | 64 | 3 | 0.09 | 1 | 0.00059 | 7.8e−05 |
| *8* | GO:0006396 | RNA processing | 267 | 3 | 0.38 | 4 | 0.03702 | 0.0051 |

| | GO.ID | MFCluster: blue4 Size: 18 | Annotated | Significant | Expected | Rank in ClassicF | Weight01F | ClassicF |
|---|---|---|---|---|---|---|---|---|
| 1 | GO:0003723 | RNA binding | 307 | 4 | 0.60 | 1 | 0.0057 | 0.0023 |
| 27 | GO:0140098 | catalytic activity, acting on RNA | 165 | 3 | 0.32 | 2 | 1.0000 | 0.0036 |

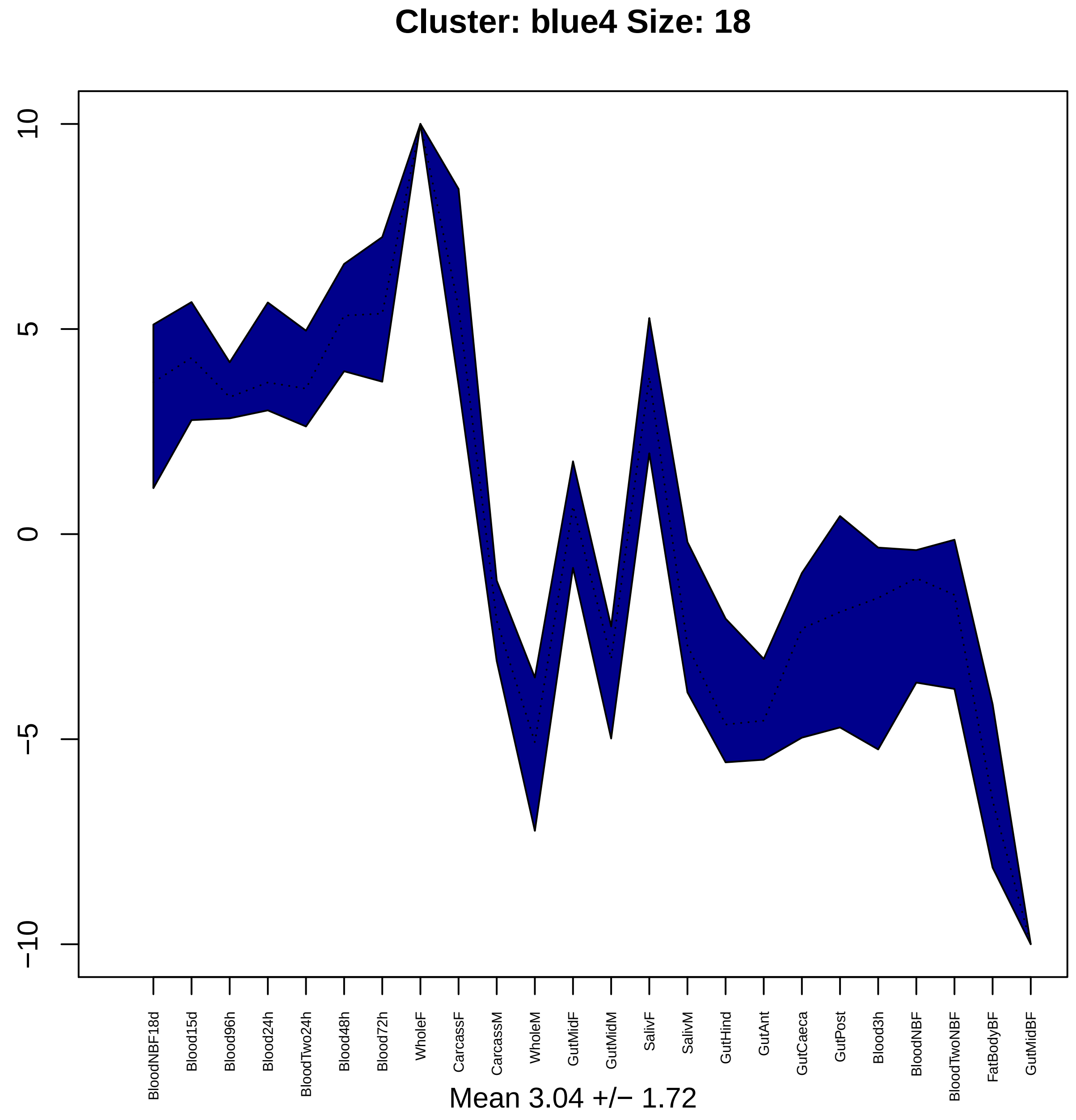

Cluster: blue4 Size: 18
10
5
0
−5
−10
BloodNBF18d
Blood15d
Blood96h
Blood24h
BloodTwo24h
Blood48h
Blood72h
WholeF
CarcassF
CarcassM
WholeM
GutMidF
GutMidM
SalivF
SalivM
GutHind
GutAnt
GutCaeca
GutPost
Blood3h
BloodNBF
BloodTwoNBF
FatBodyBF
GutMidBF
Mean 3.04 +/− 1.72

# Cluster: magenta3 Size: 16

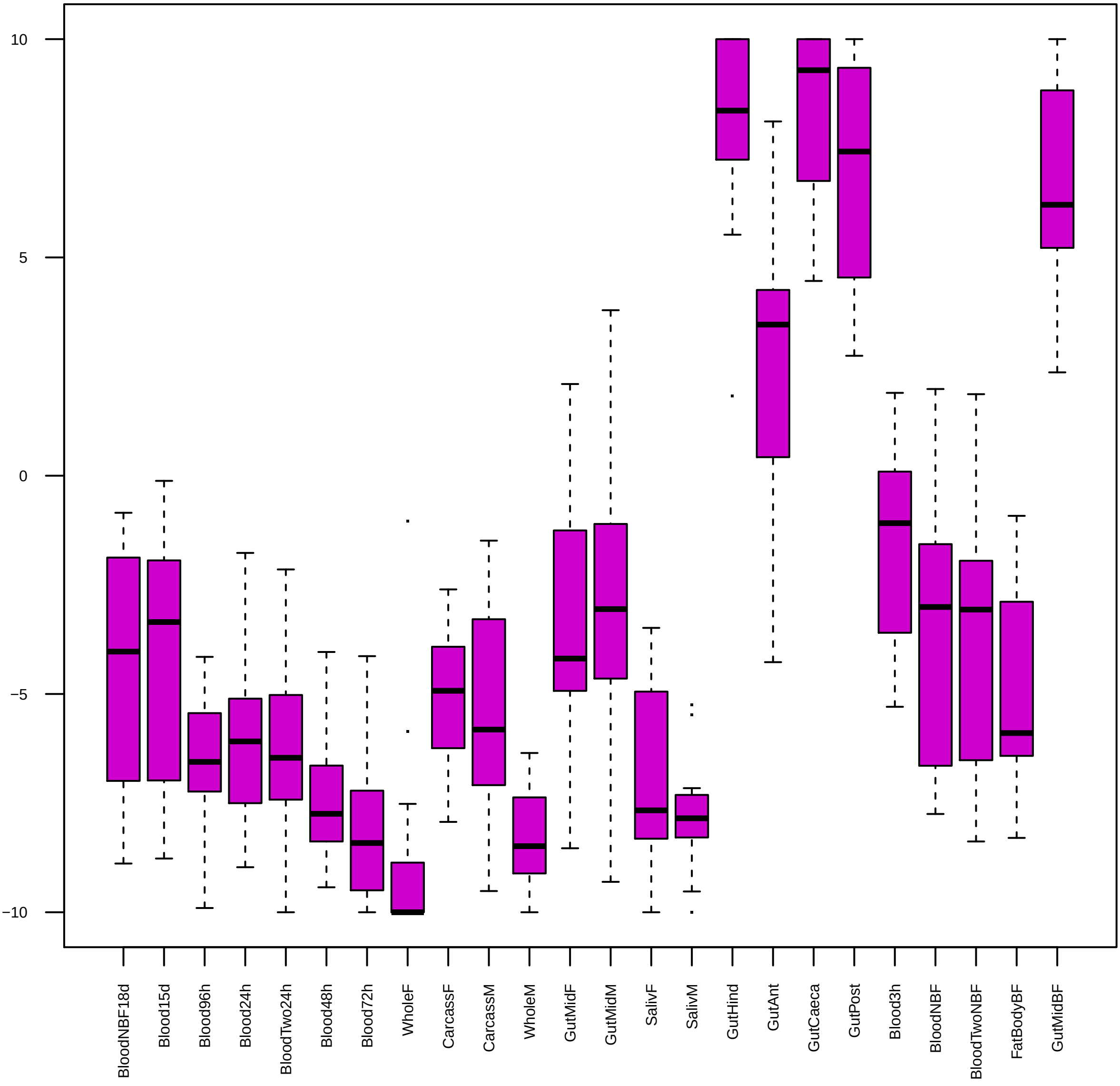

| | GO.ID | MFCluster: magenta3 Size: 16 | Annotated | Significant | Expected | Rank in ClassicF | Weight01F | ClassicF |
|---|---|---|---|---|---|---|---|---|
| *1* | GO:0004553 | hydrolase activity, hydrolyzing O−glycos... | 84 | 2 | 0.10 | 1 | 0.0046 | 0.0046 |
| *17* | GO:0016798 | hydrolase activity, acting on glycosyl b... | 95 | 2 | 0.12 | 2 | 1.0000 | 0.0058 |

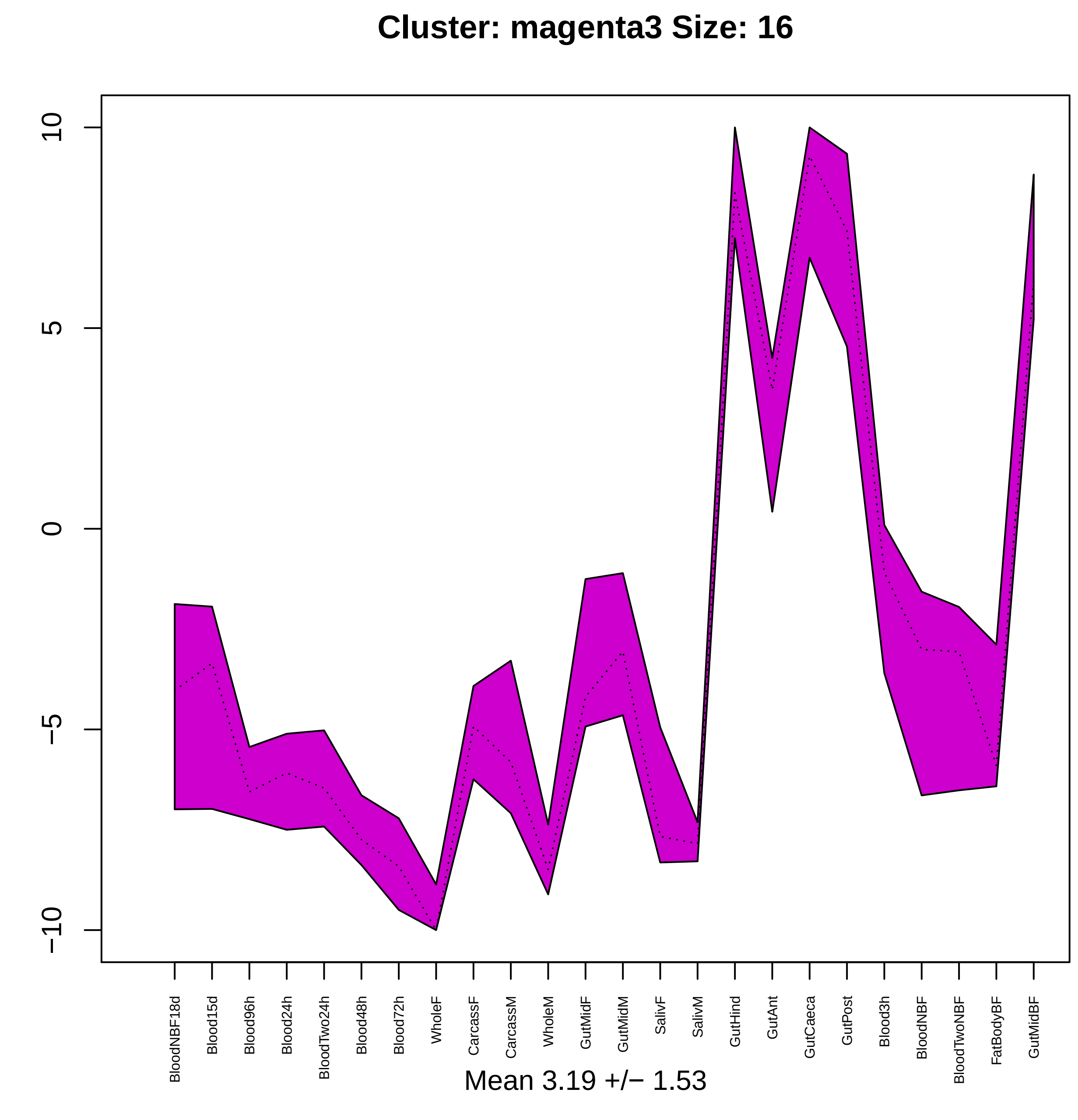
Cluster: magenta3 Size: 16
10
5
0
−5
−10
BloodNBF18d
Blood15d
Blood96h
Blood24h
BloodTwo24h
Blood48h
Blood72h
WholeF
CarcassF
CarcassM
WholeM
GutMidF
GutMidM
SalivF
SalivM
GutHind
GutAnt
GutCaeca
GutPost
Blood3h
BloodNBF
BloodTwoNBF
FatBodyBF
GutMidBF
Mean 3.19 +/− 1.53

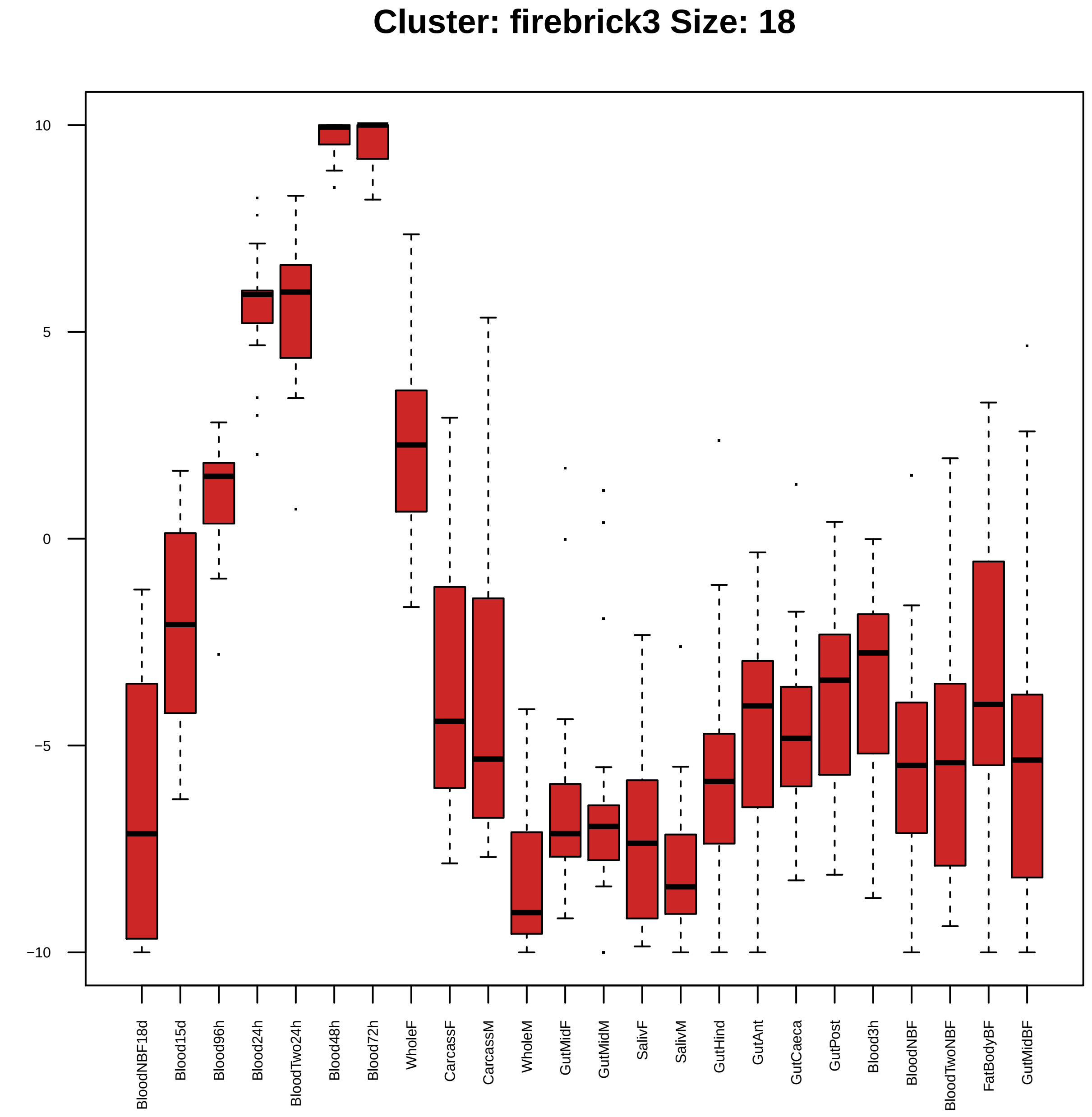
Cluster: firebrick3 Size: 18
10
5
0
−5
−10
BloodNBF18d
Blood15d
Blood96h
Blood24h
BloodTwo24h
Blood48h
Blood72h
WholeF
CarcassF
CarcassM
WholeM
GutMidF
GutMidM
SalivF
SalivM
GutHind
GutAnt
GutCaeca
GutPost
Blood3h
BloodNBF
BloodTwoNBF
FatBodyBF
GutMidBF

| | GO.ID | BPCluster: firebrick3 Size: 18 | Annotated | Significant | Expected | Rank in ClassicF | Weight01F | ClassicF |
|---|---|---|---|---|---|---|---|---|
| *1* | GO:0051298 | centrosome duplication | 51 | 2 | 0.11 | 17 | 0.0054 | 0.0054 |
| *2* | GO:0140014 | mitotic nuclear division | 105 | 3 | 0.23 | 2 | 0.0057 | 0.0014 |
| *3* | GO:0051301 | cell division | 126 | 3 | 0.28 | 7 | 0.0105 | 0.0023 |
| *4* | GO:0007049 | cell cycle | 432 | 5 | 0.95 | 3 | 0.0167 | 0.0017 |
| *22* | GO:0007088 | regulation of mitotic nuclear division | 31 | 2 | 0.07 | 5 | 0.0322 | 0.0020 |

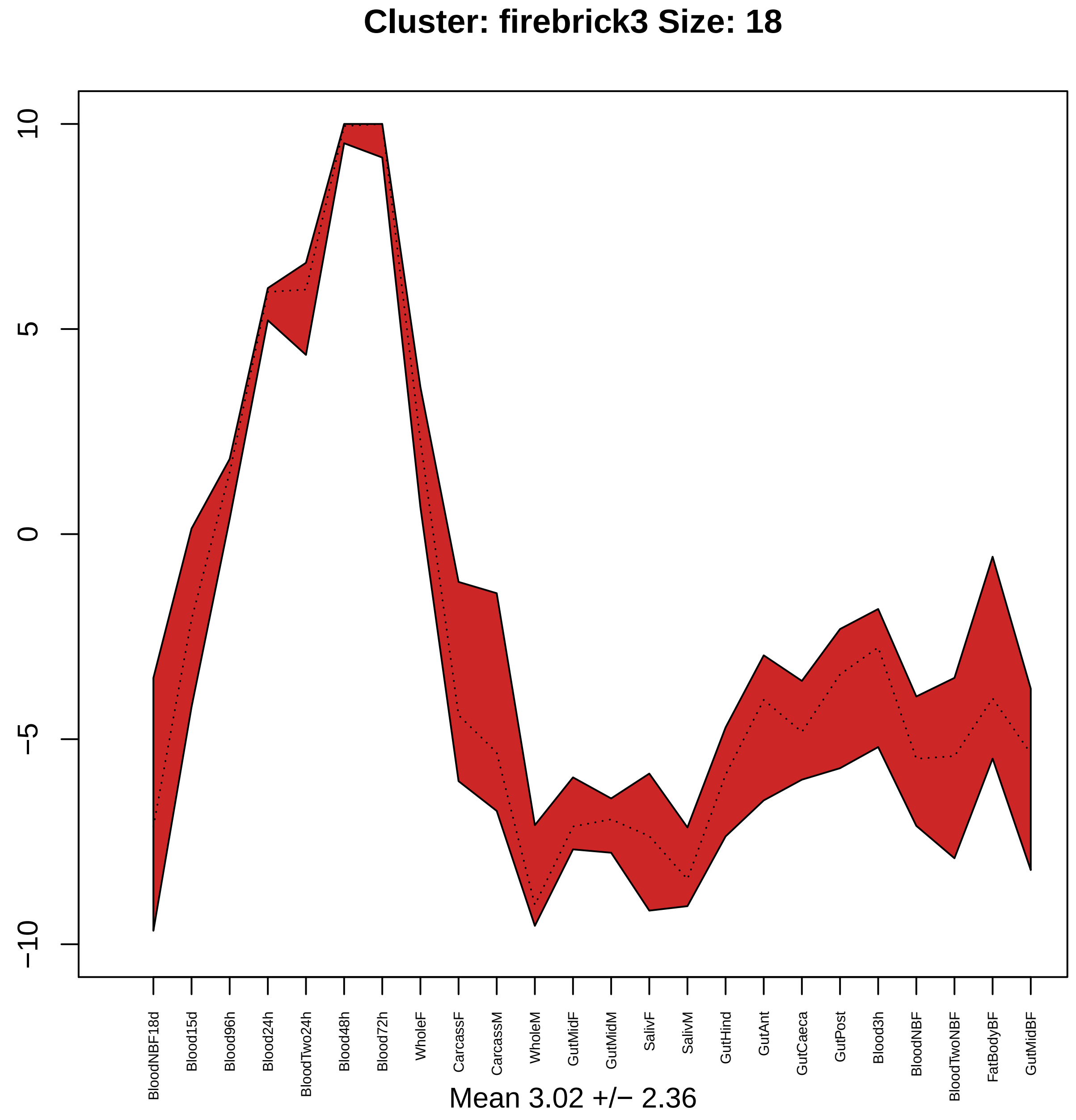

Cluster: firebrick3 Size: 18
10
5
0
−5
−10
BloodNBF18d
Blood15d
Blood96h
Blood24h
BloodTwo24h
Blood48h
Blood72h
WholeF
CarcassF
CarcassM
WholeM
GutMidF
GutMidM
SalivF
SalivM
GutHind
GutAnt
GutCaeca
GutPost
Blood3h
BloodNBF
BloodTwoNBF
FatBodyBF
GutMidBF
Mean 3.02 +/− 2.36

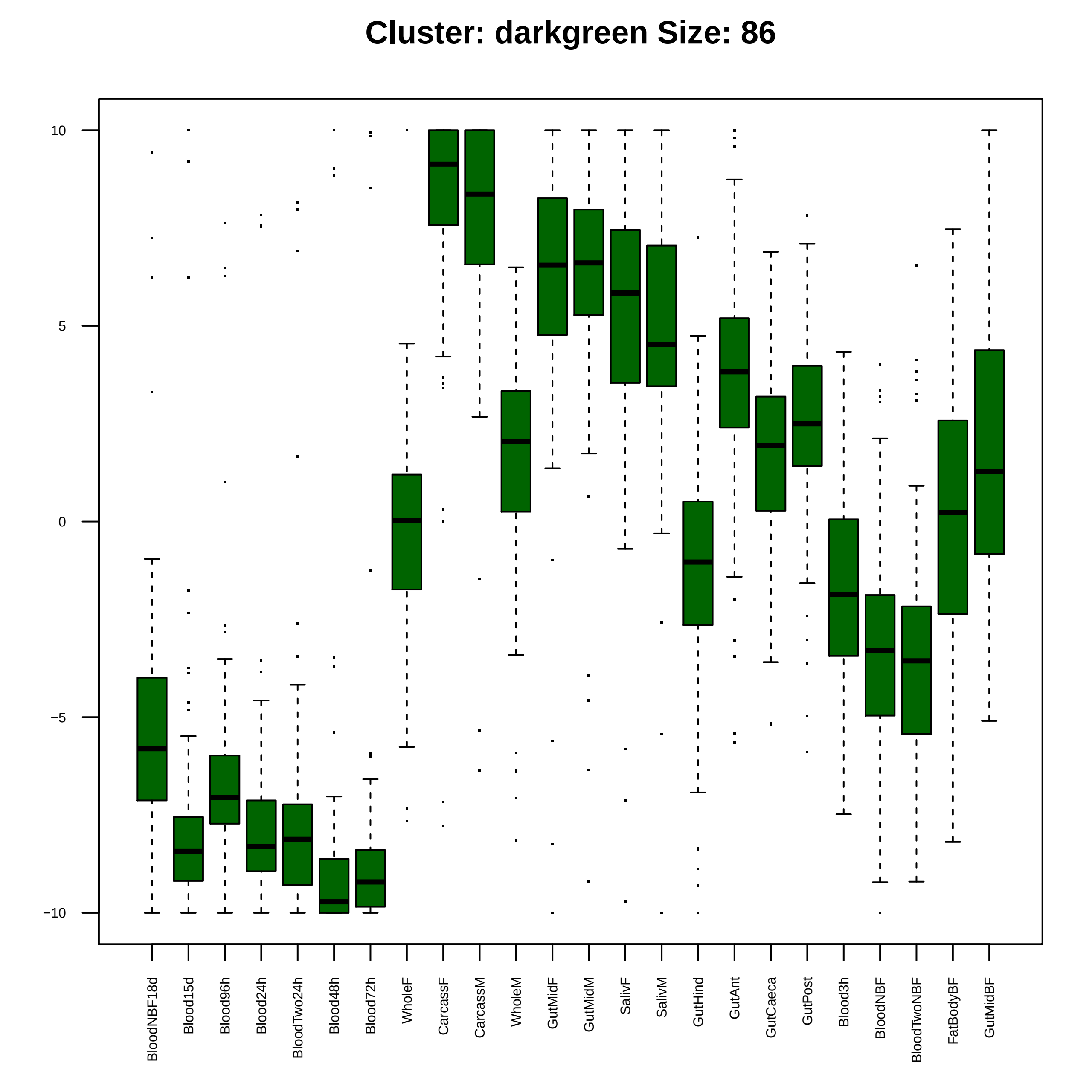
Cluster: darkgreen Size: 86
10
5
0
−5
−10
BloodNBF18d
Blood15d
Blood96h
Blood24h
BloodTwo24h
Blood48h
Blood72h
WholeF
CarcassF
CarcassM
WholeM
GutMidF
GutMidM
SalivF
SalivM
GutHind
GutAnt
GutCaeca
GutPost
Blood3h
BloodNBF
BloodTwoNBF
FatBodyBF
GutMidBF

| | GO.ID | BPCluster: darkgreen Size: 86 | Annotated | Significant | Expected | Rank in ClassicF | Weight01F | ClassicF |
|---|---|---|---|---|---|---|---|---|
| 1 | GO:0022404 | molting cycle process | 10 | 2 | 0.08 | 2 | 0.0025 | 0.0025 |
| 2 | GO:0007432 | salivary gland boundary specification | 12 | 2 | 0.09 | 3 | 0.0036 | 0.0036 |
| 3 | GO:0016042 | lipid catabolic process | 53 | 3 | 0.41 | 6 | 0.0076 | 0.0076 |

| | GO.ID | MFCluster: darkgreen Size: 86 | Annotated | Significant | Expected | Rank in ClassicF | Weight01F | ClassicF |
|---|---|---|---|---|---|---|---|---|
| *1* | GO:0016298 | lipase activity | 36 | 3 | 0.26 | 2 | 0.0038 | 0.0023 |
| *3* | GO:0052689 | carboxylic ester hydrolase activity | 35 | 3 | 0.26 | 1 | 0.0138 | 0.0021 |
| *12* | GO:0004930 | G protein−coupled receptor activity | 171 | 5 | 1.26 | 3 | 0.0859 | 0.0081 |

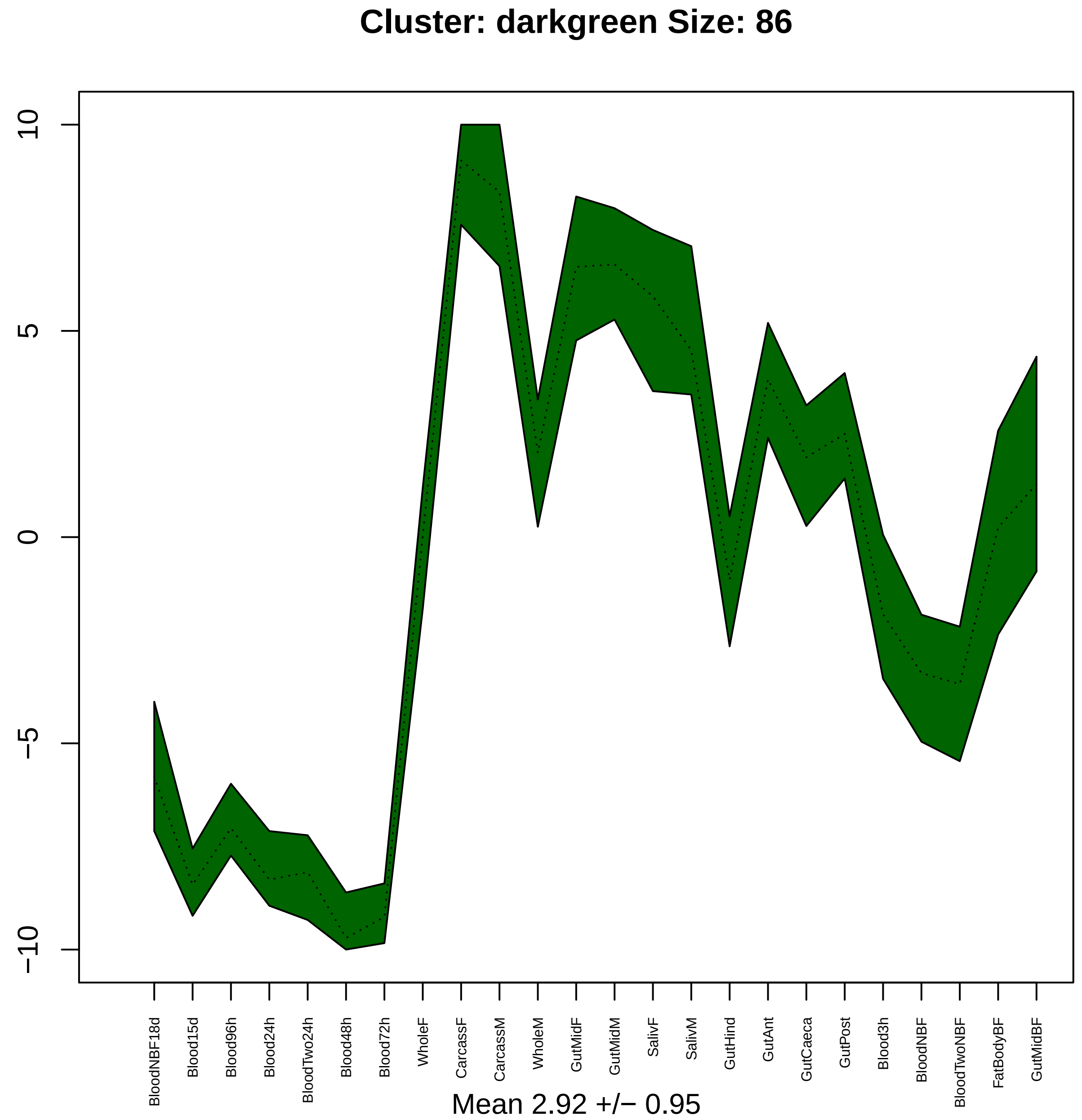

Cluster: darkgreen Size: 86
10
5
0
−5
−10
BloodNBF18d
Blood15d
Blood96h
Blood24h
BloodTwo24h
Blood48h
Blood72h
WholeF
CarcassF
CarcassM
WholeM
GutMidF
GutMidM
SalivF
SalivM
GutHind
GutAnt
GutCaeca
GutPost
Blood3h
BloodNBF
BloodTwoNBF
FatBodyBF
GutMidBF
Mean 2.92 +/− 0.95

# Cluster: indianred3 Size: 18

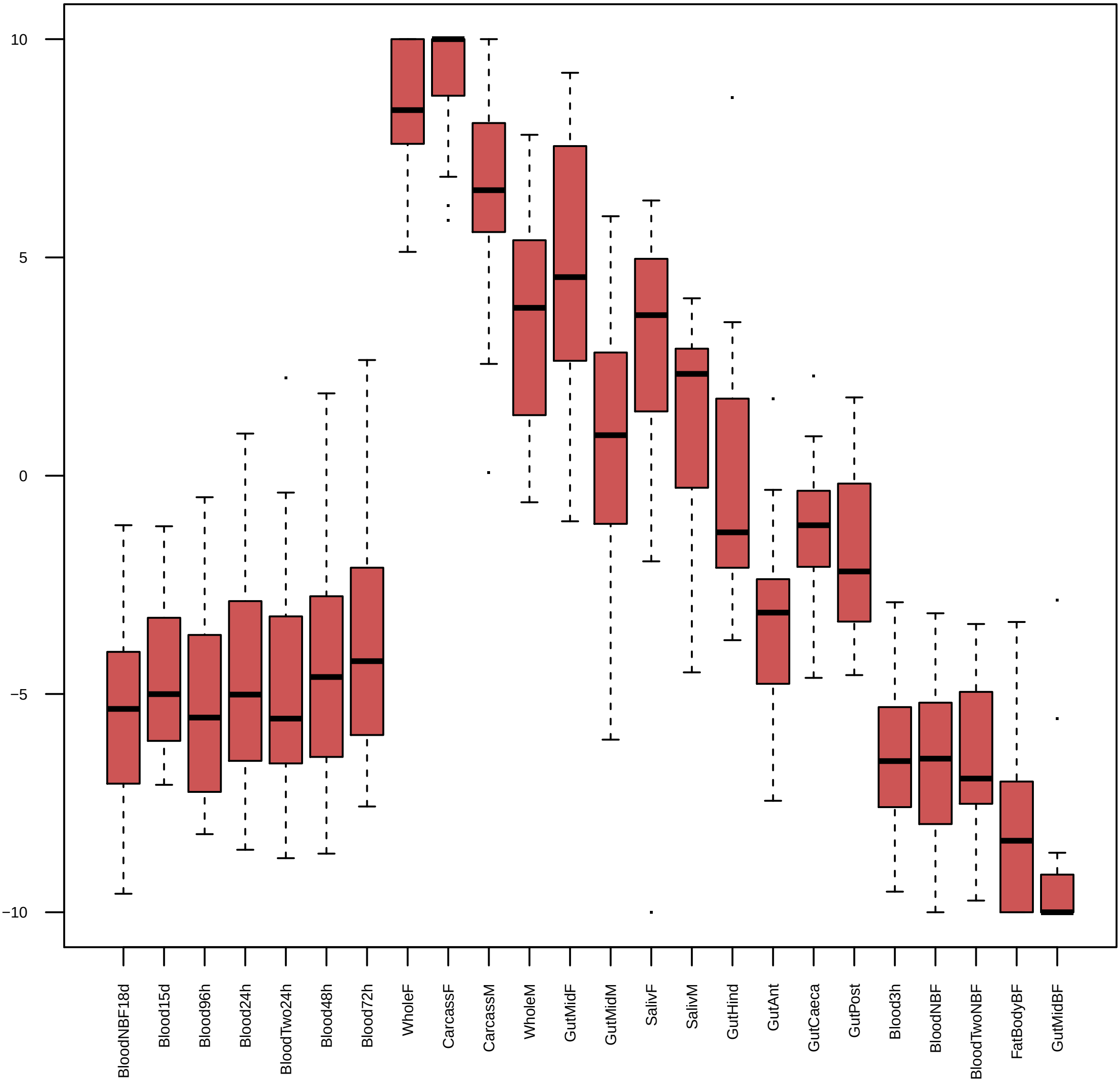

| | GO.ID | BPCluster: indianred3 Size: 18 | Annotated | Significant | Expected | Rank in ClassicF | Weight01F | ClassicF |
|---|---|---|---|---|---|---|---|---|
| *1* | GO:0006457 | protein folding | 72 | 3 | 0.19 | 1 | 0.00084 | 0.00084 |
| *2* | GO:0006412 | translation | 289 | 4 | 0.77 | 5 | 0.00615 | 0.00615 |

| | GO.ID | MFCluster: indianred3 Size: 18 | Annotated | Significant | Expected | Rank in ClassicF | Weight01F | ClassicF |
|---|---|---|---|---|---|---|---|---|
| *2* | GO:0003735 | structural constituent of ribosome | 146 | 3 | 0.3 | 2 | 0.0031 | 0.0031 |

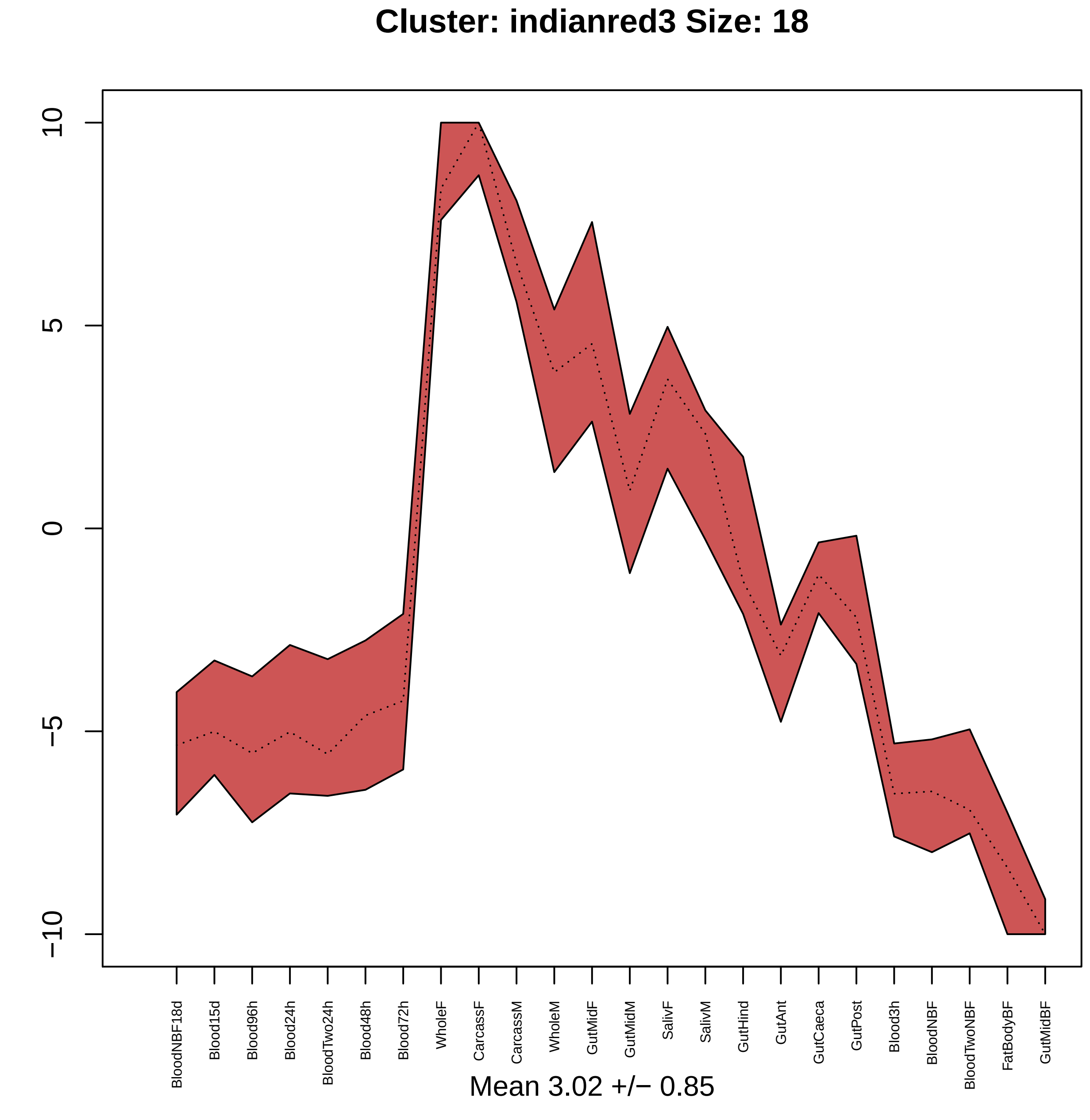

Cluster: indianred3 Size: 18
10
5
0
−5
−10
BloodNBF18d
Blood15d
Blood96h
Blood24h
BloodTwo24h
Blood48h
Blood72h
WholeF
CarcassF
CarcassM
WholeM
GutMidF
GutMidM
SalivF
SalivM
GutHind
GutAnt
GutCaeca
GutPost
Blood3h
BloodNBF
BloodTwoNBF
FatBodyBF
GutMidBF
Mean 3.02 +/− 0.85

# Cluster: yellowgreen Size: 57

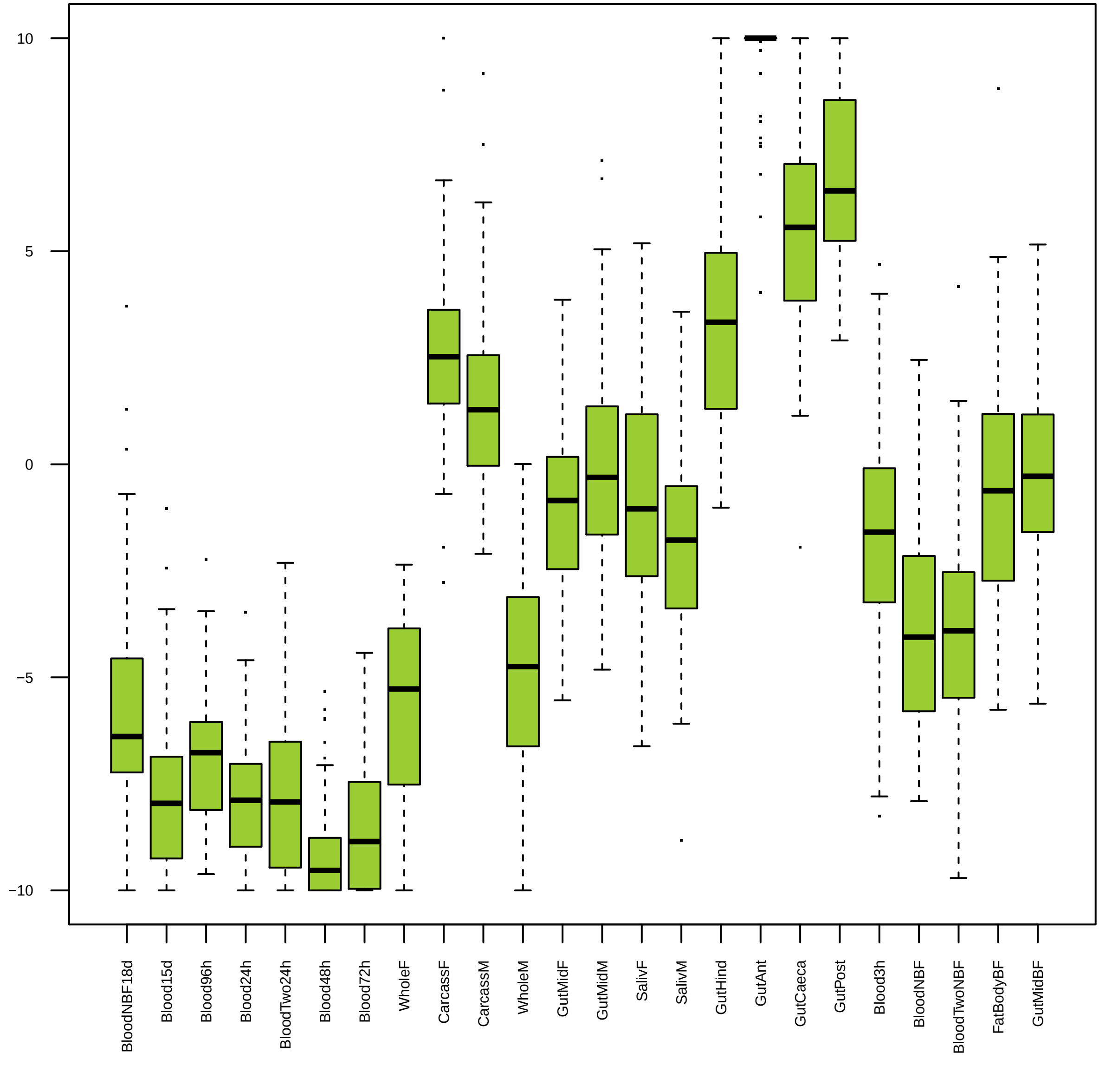

| | GO.ID | BPCluster: yellowgreen Size: 57 | Annotated | Significant | Expected | Rank in ClassicF | Weight01F | ClassicF |
|---|---|---|---|---|---|---|---|---|
| 1 | GO:0002805 | regulation of antimicrobial peptide bios... | 18 | 2 | 0.08 | 2 | 0.014 | 0.0031 |

| | GO.ID | MFCluster: yellowgreen Size: 57 | Annotated | Significant | Expected | Rank in ClassicF | Weight01F | ClassicF |
|---|---|---|---|---|---|---|---|---|
| 6 | GO:0005216 | ion channel activity | 139 | 4 | 0.69 | 1 | 0.076 | 0.0048 |

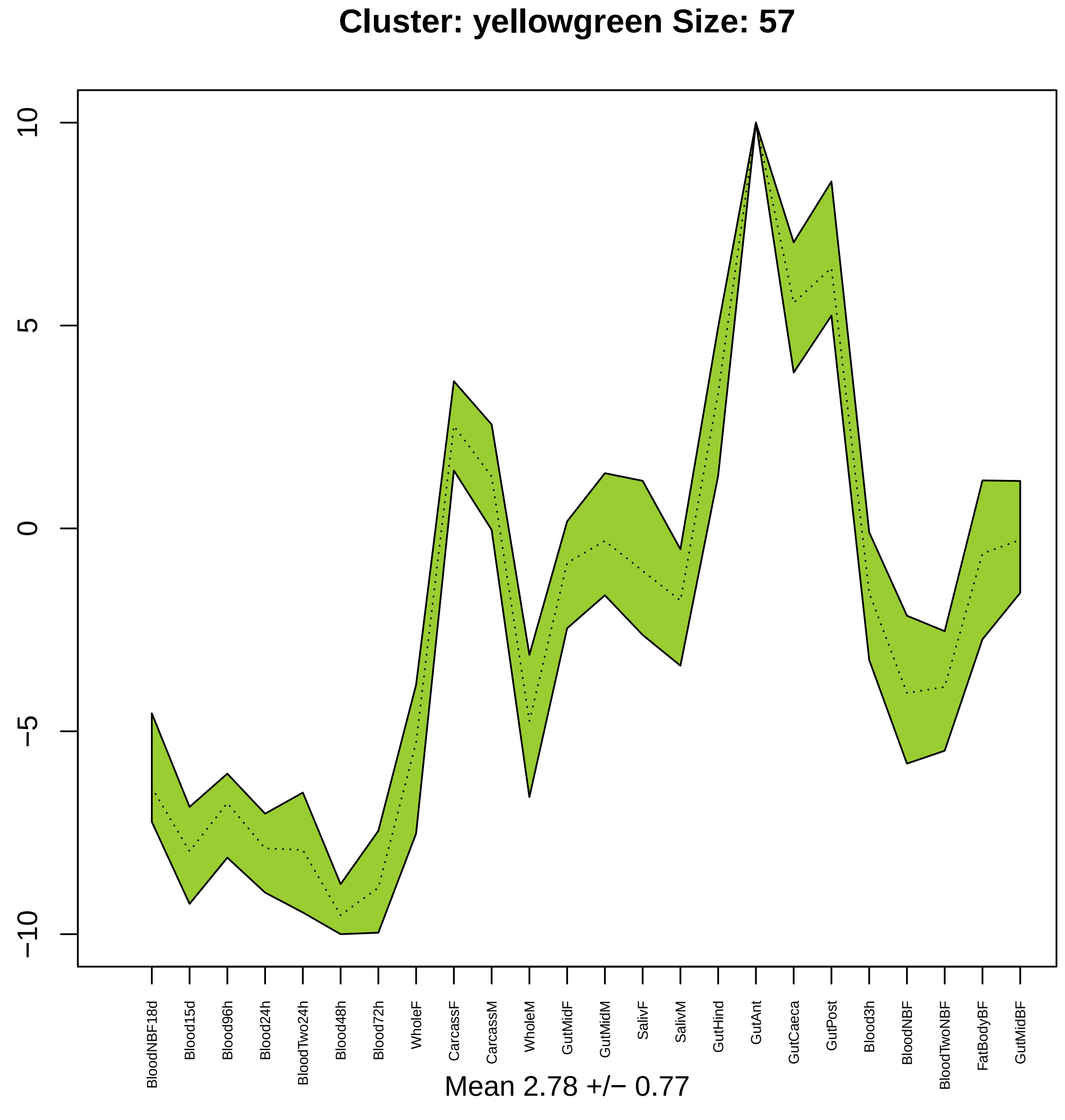

Cluster: yellowgreen Size: 57
10
5
0
−5
−10
BloodNBF18d
Blood15d
Blood96h
Blood24h
BloodTwo24h
Blood48h
Blood72h
WholeF
CarcassF
CarcassM
WholeM
GutMidF
GutMidM
SalivF
SalivM
GutHind
GutAnt
GutCaeca
GutPost
Blood3h
BloodNBF
BloodTwoNBF
FatBodyBF
GutMidBF
Mean 2.78 +/− 0.77

# Cluster: indianred4 Size: 29

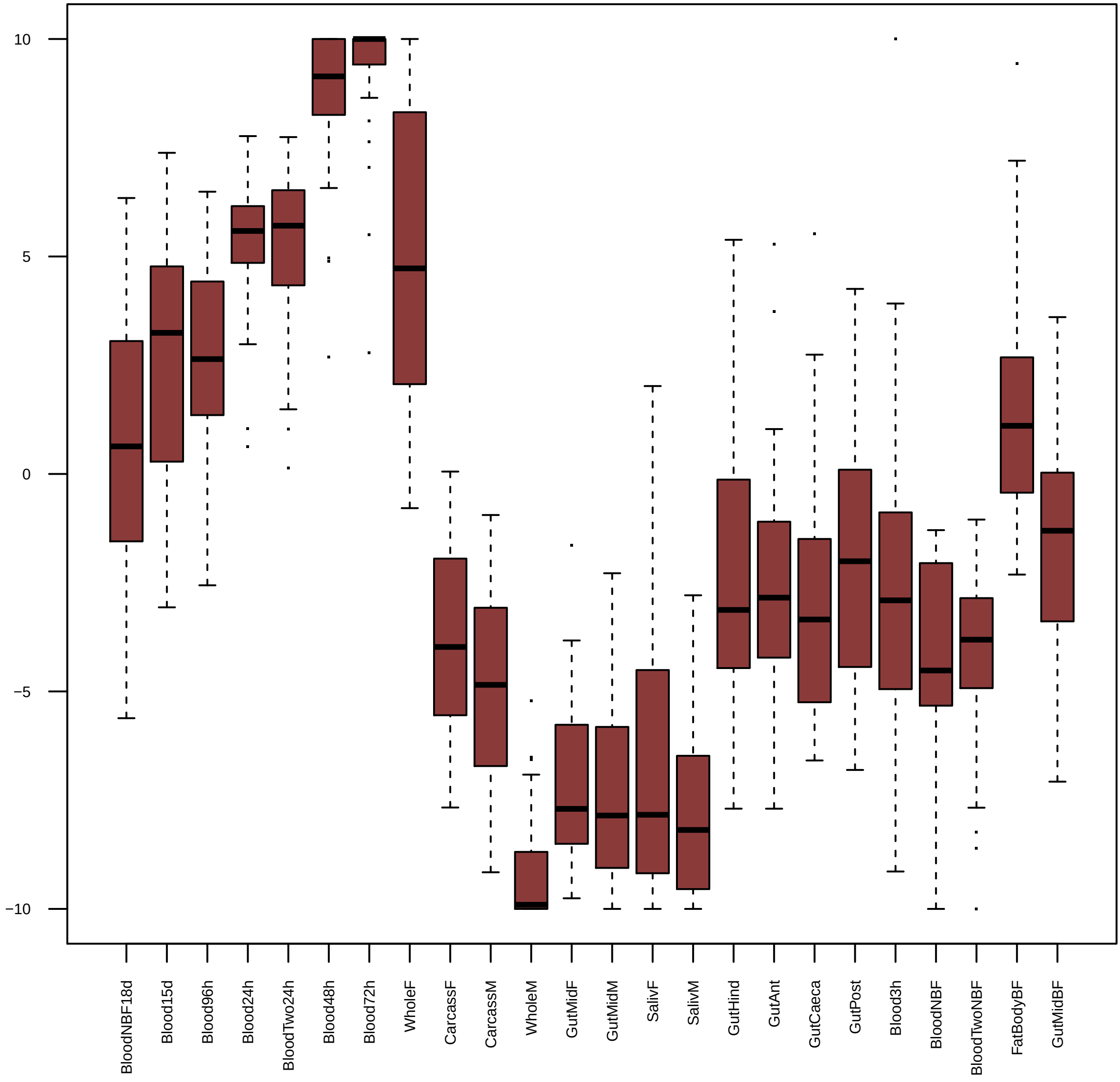

| | GO.ID | BPCluster: indianred4 Size: 29 | Annotated | Significant | Expected | Rank in ClassicF | Weight01F | ClassicF |
|---|---|---|---|---|---|---|---|---|
| 1 | GO:0046331 | lateral inhibition | 92 | 3 | 0.29 | 11 | 0.0028 | 0.0028 |
| 2 | GO:0007030 | Golgi organization | 37 | 2 | 0.12 | 18 | 0.0058 | 0.0058 |
| 3 | GO:0018108 | peptidyl−tyrosine phosphorylation | 38 | 2 | 0.12 | 21 | 0.0061 | 0.0061 |
| 4 | GO:0010389 | regulation of G2/M transition of mitotic... | 48 | 2 | 0.15 | 26 | 0.0119 | 0.0097 |

| | GO.ID | MFCluster: indianred4 Size: 29 | Annotated | Significant | Expected | Rank in ClassicF | Weight01F | ClassicF |
|---|---|---|---|---|---|---|---|---|
| *1* | GO:0043138 | 3'−5' DNA helicase activity | 11 | 2 | 0.04 | 1 | 0.00053 | 0.00053 |
| *9* | GO:0004713 | protein tyrosine kinase activity | 36 | 2 | 0.11 | 5 | 0.07662 | 0.00574 |

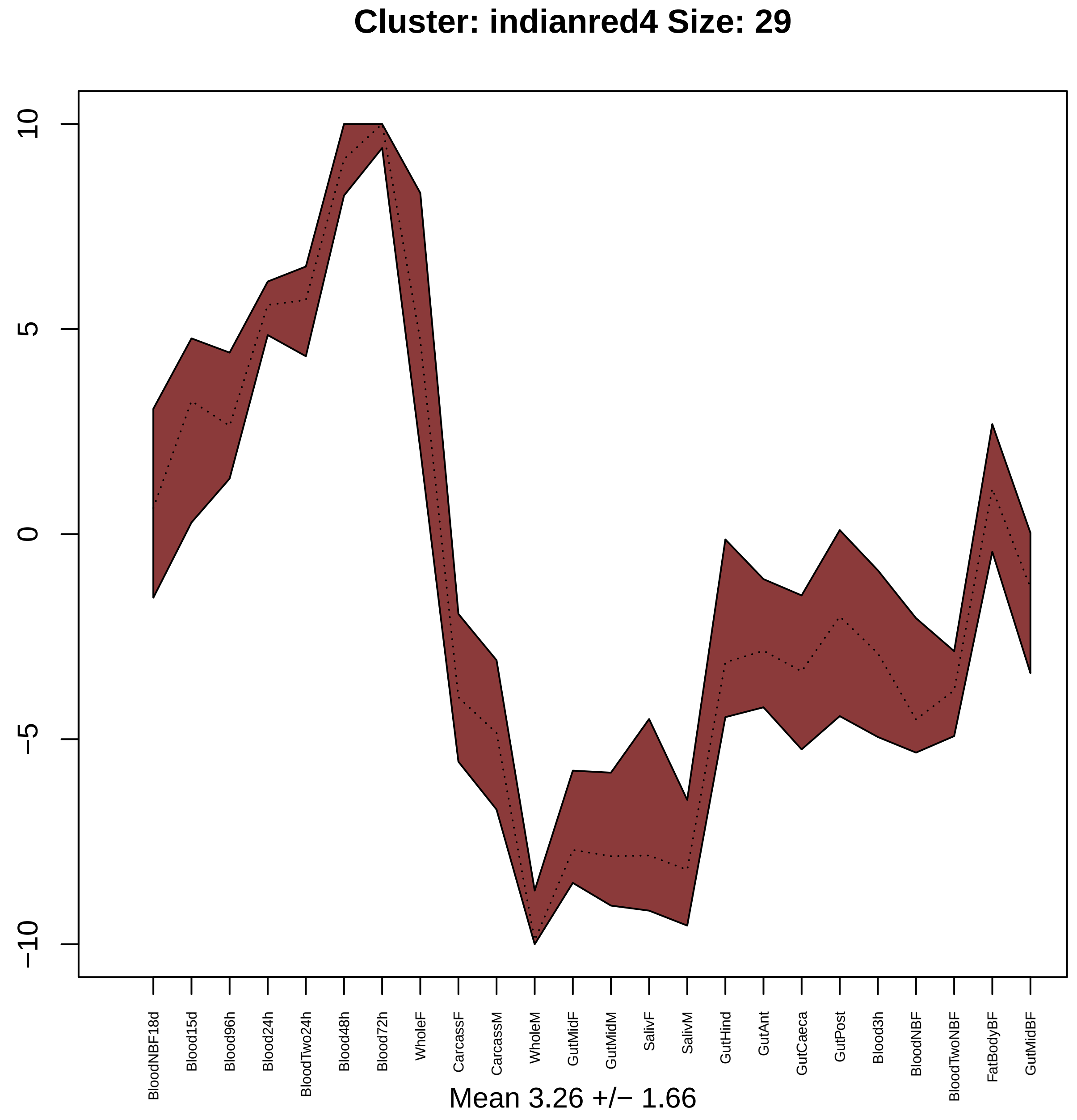

Cluster: indianred4 Size: 29
10
5
0
−5
−10
BloodNBF18d
Blood15d
Blood96h
Blood24h
BloodTwo24h
Blood48h
Blood72h
WholeF
CarcassF
CarcassM
WholeM
GutMidF
GutMidM
SalivF
SalivM
GutHind
GutAnt
GutCaeca
GutPost
Blood3h
BloodNBF
BloodTwoNBF
FatBodyBF
GutMidBF
Mean 3.26 +/− 1.66

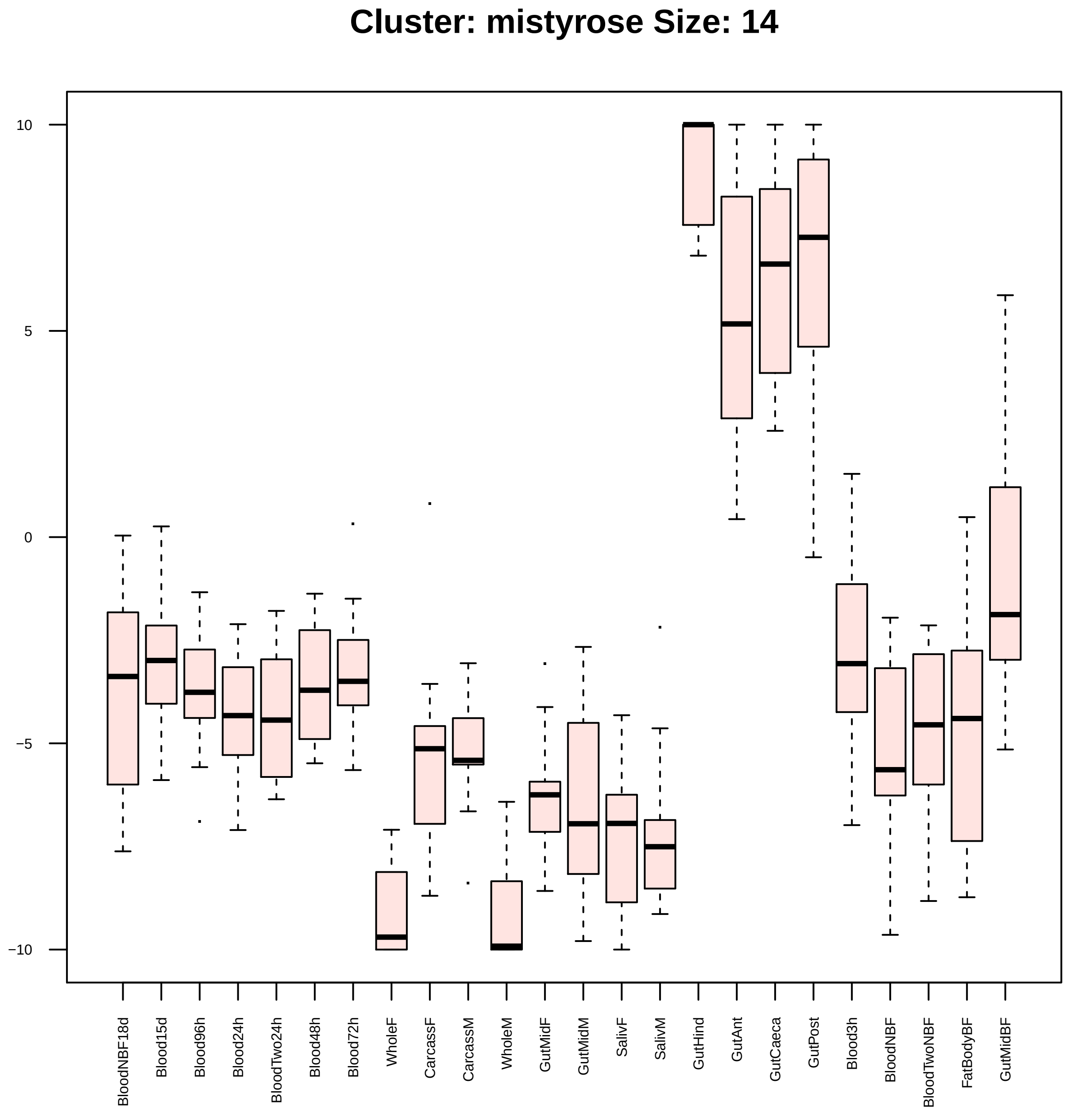

Cluster: mistyrose Size: 14
10
5
0
−5
−10
BloodNBF18d
Blood15d
Blood96h
Blood24h
BloodTwo24h
Blood48h
Blood72h
WholeF
CarcassF
CarcassM
WholeM
GutMidF
GutMidM
SalivF
SalivM
GutHind
GutAnt
GutCaeca
GutPost
Blood3h
BloodNBF
BloodTwoNBF
FatBodyBF
GutMidBF

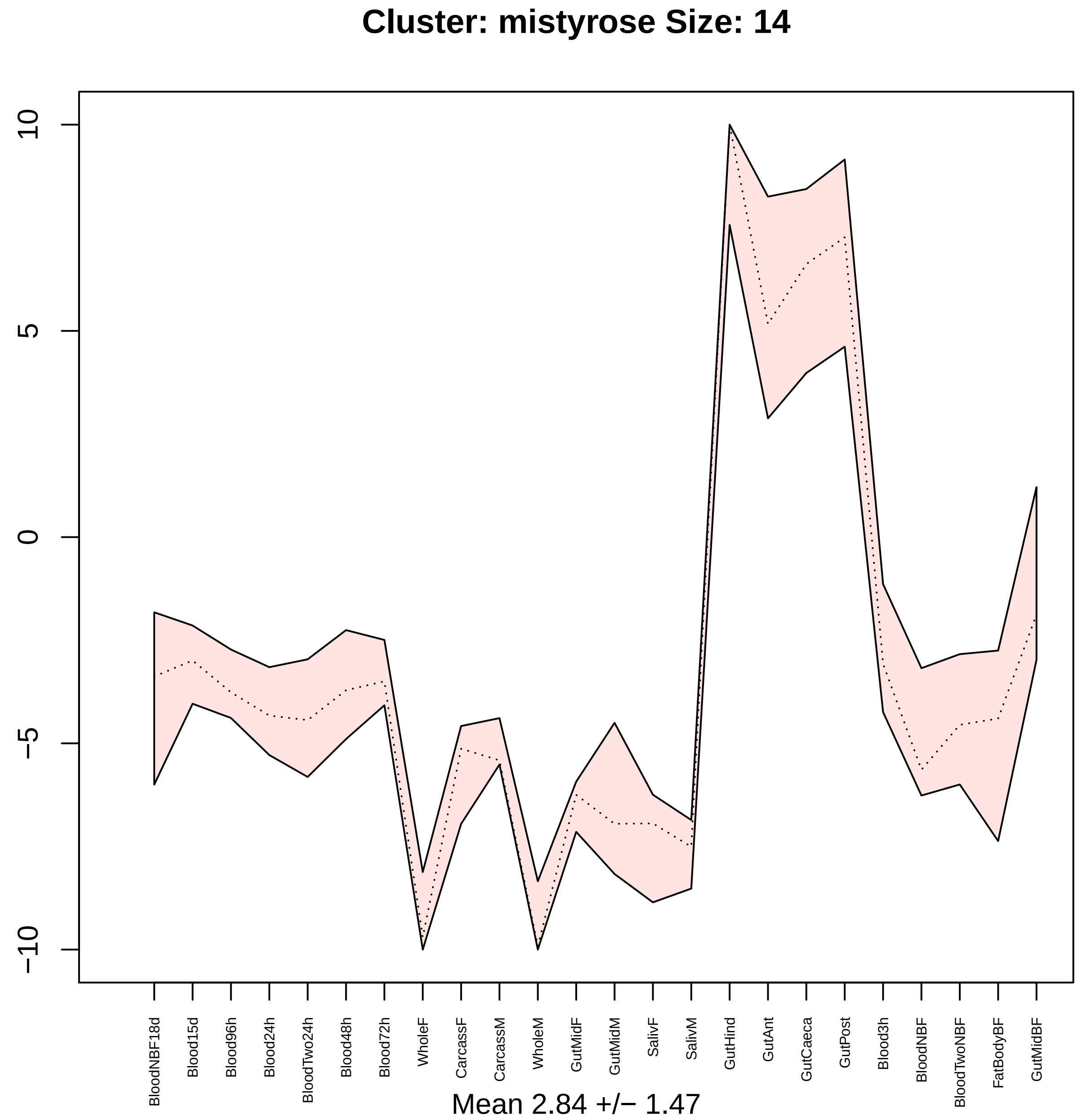

Cluster: mistyrose Size: 14
10
5
0
−5
−10
BloodNBF18d
Blood15d
Blood96h
Blood24h
BloodTwo24h
Blood48h
Blood72h
WholeF
CarcassF
CarcassM
WholeM
GutMidF
GutMidM
SalivF
SalivM
GutHind
GutAnt
GutCaeca
GutPost
Blood3h
BloodNBF
BloodTwoNBF
FatBodyBF
GutMidBF
Mean 2.84 +/− 1.47

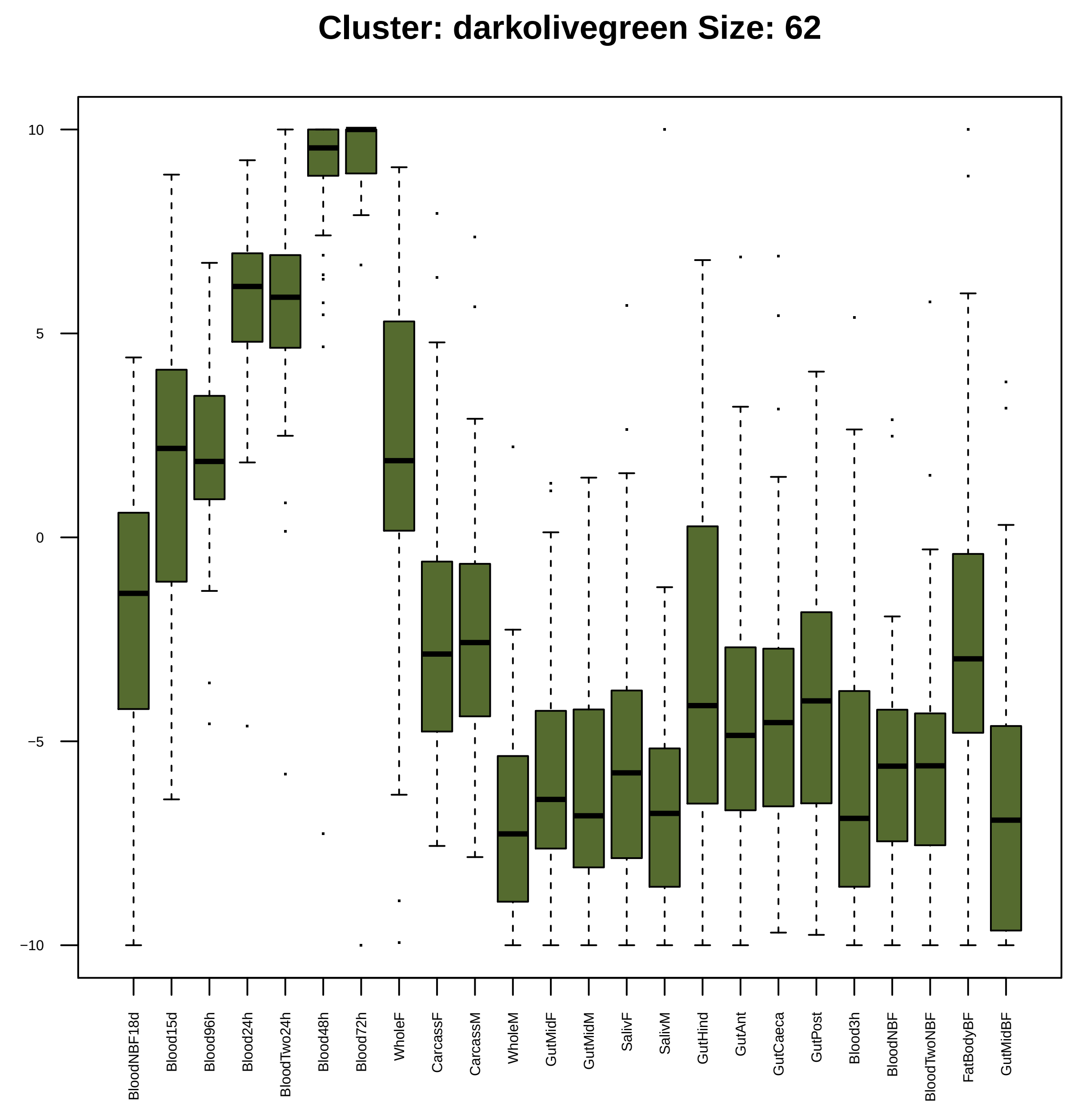

Cluster: darkolivegreen Size: 62
10
5
0
−5
−10
BloodNBF18d
Blood15d
Blood96h
Blood24h
BloodTwo24h
Blood48h
Blood72h
WholeF
CarcassF
CarcassM
WholeM
GutMidF
GutMidM
SalivF
SalivM
GutHind
GutAnt
GutCaeca
GutPost
Blood3h
BloodNBF
BloodTwoNBF
FatBodyBF
GutMidBF

| | GO.ID | BPCluster: darkolivegreen Size: 62 | Annotated | Significant | Expected | Rank in ClassicF | Weight01F | ClassicF |
|---|---|---|---|---|---|---|---|---|
| 1 | GO:0007093 | mitotic cell cycle checkpoint | 56 | 3 | 0.30 | 11 | 0.00093 | 0.00317 |
| 2 | GO:0007346 | regulation of mitotic cell cycle | 114 | 5 | 0.61 | 3 | 0.00238 | 0.00031 |
| 3 | GO:0030334 | regulation of cell migration | 14 | 2 | 0.07 | 8 | 0.00241 | 0.00241 |
| 4 | GO:0051179 | localization | 1336 | 10 | 7.12 | 247 | 0.00496 | 0.15710 |
| 5 | GO:0022008 | neurogenesis | 706 | 10 | 3.76 | 10 | 0.01309 | 0.00286 |
| 11 | GO:0040012 | regulation of locomotion | 21 | 3 | 0.11 | 2 | 0.03469 | 0.00017 |

| | GO.ID | MFCluster: darkolivegreen Size: 62 | Annotated | Significant | Expected | Rank in ClassicF | Weight01F | ClassicF |
|---|---|---|---|---|---|---|---|---|
| *1* | GO:0008017 | microtubule binding | 55 | 4 | 0.33 | 1 | 0.0003 | 0.0003 |
| *5* | GO:0004536 | deoxyribonuclease activity | 19 | 2 | 0.11 | 4 | 0.0402 | 0.0056 |

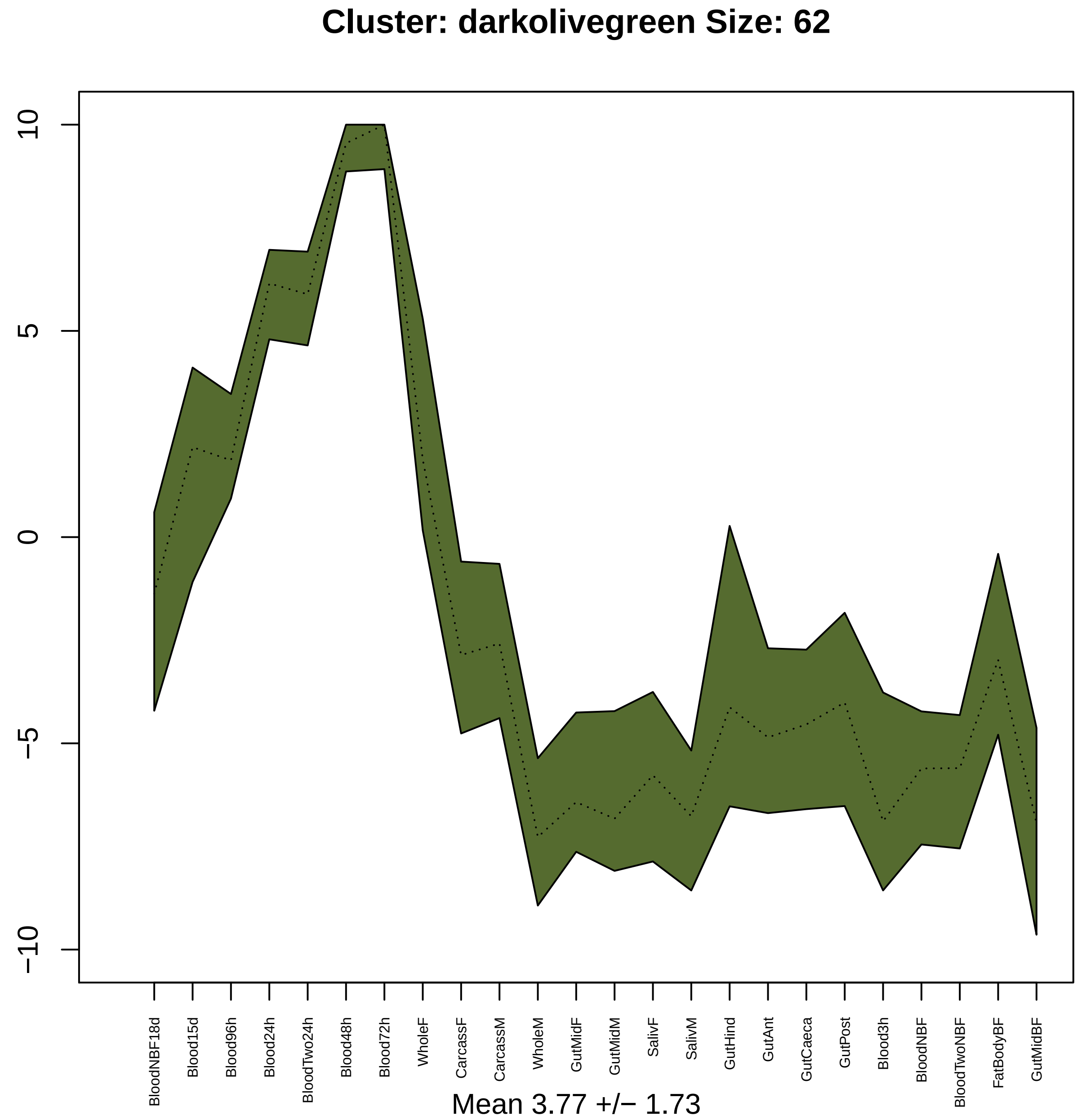

Cluster: darkolivegreen Size: 62
10
5
0
−5
−10
BloodNBF18d
Blood15d
Blood96h
Blood24h
BloodTwo24h
Blood48h
Blood72h
WholeF
CarcassF
CarcassM
WholeM
GutMidF
GutMidM
SalivF
SalivM
GutHind
GutAnt
GutCaeca
GutPost
Blood3h
BloodNBF
BloodTwoNBF
FatBodyBF
GutMidBF
Mean 3.77 +/− 1.73

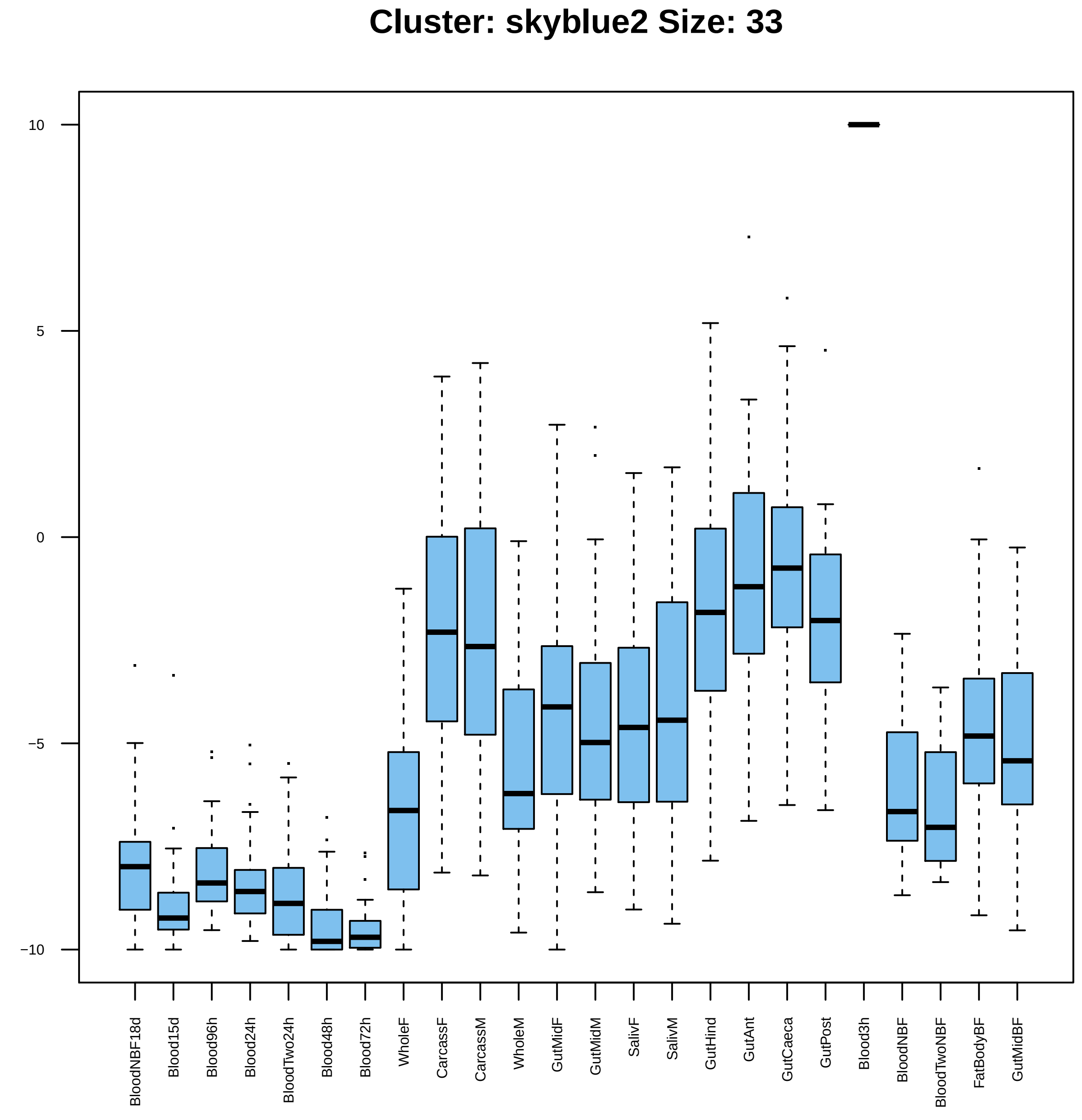
Cluster: skyblue2 Size: 33
10
5
0
−5
−10
BloodNBF18d
Blood15d
Blood96h
Blood24h
BloodTwo24h
Blood48h
Blood72h
WholeF
CarcassF
CarcassM
WholeM
GutMidF
GutMidM
SalivF
SalivM
GutHind
GutAnt
GutCaeca
GutPost
Blood3h
BloodNBF
BloodTwoNBF
FatBodyBF
GutMidBF

| | GO.ID | BPCluster: skyblue2 Size: 33 | Annotated | Significant | Expected | Rank in ClassicF | Weight01F | ClassicF |
|---|---|---|---|---|---|---|---|---|
| *3* | GO:0042737 | drug catabolic process | 48 | 2 | 0.08 | 1 | 0.034 | 0.0024 |
| *9* | GO:0006030 | chitin metabolic process | 95 | 2 | 0.15 | 3 | 0.096 | 0.0091 |
| *17* | GO:1901071 | glucosamine−containing compound metaboli... | 99 | 2 | 0.16 | 5 | 1.000 | 0.0099 |
| *22* | GO:1901564 | organonitrogen compound metabolic proces... | 1889 | 7 | 2.96 | 4 | 1.000 | 0.0098 |

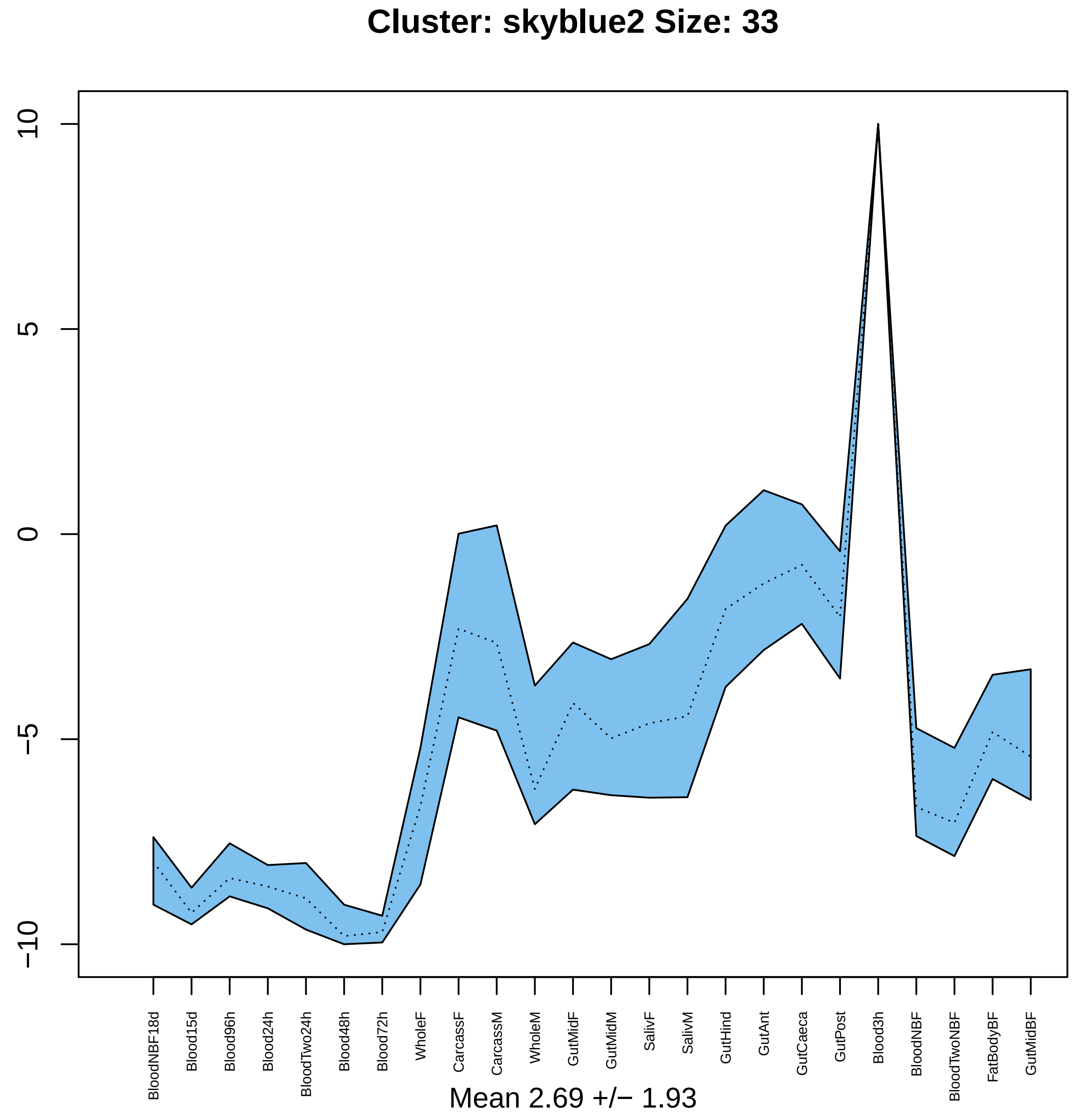

Cluster: skyblue2 Size: 33
10
5
0
−5
−10
BloodNBF18d
Blood15d
Blood96h
Blood24h
BloodTwo24h
Blood48h
Blood72h
WholeF
CarcassF
CarcassM
WholeM
GutMidF
GutMidM
SalivF
SalivM
GutHind
GutAnt
GutCaeca
GutPost
Blood3h
BloodNBF
BloodTwoNBF
FatBodyBF
GutMidBF
Mean 2.69 +/− 1.93

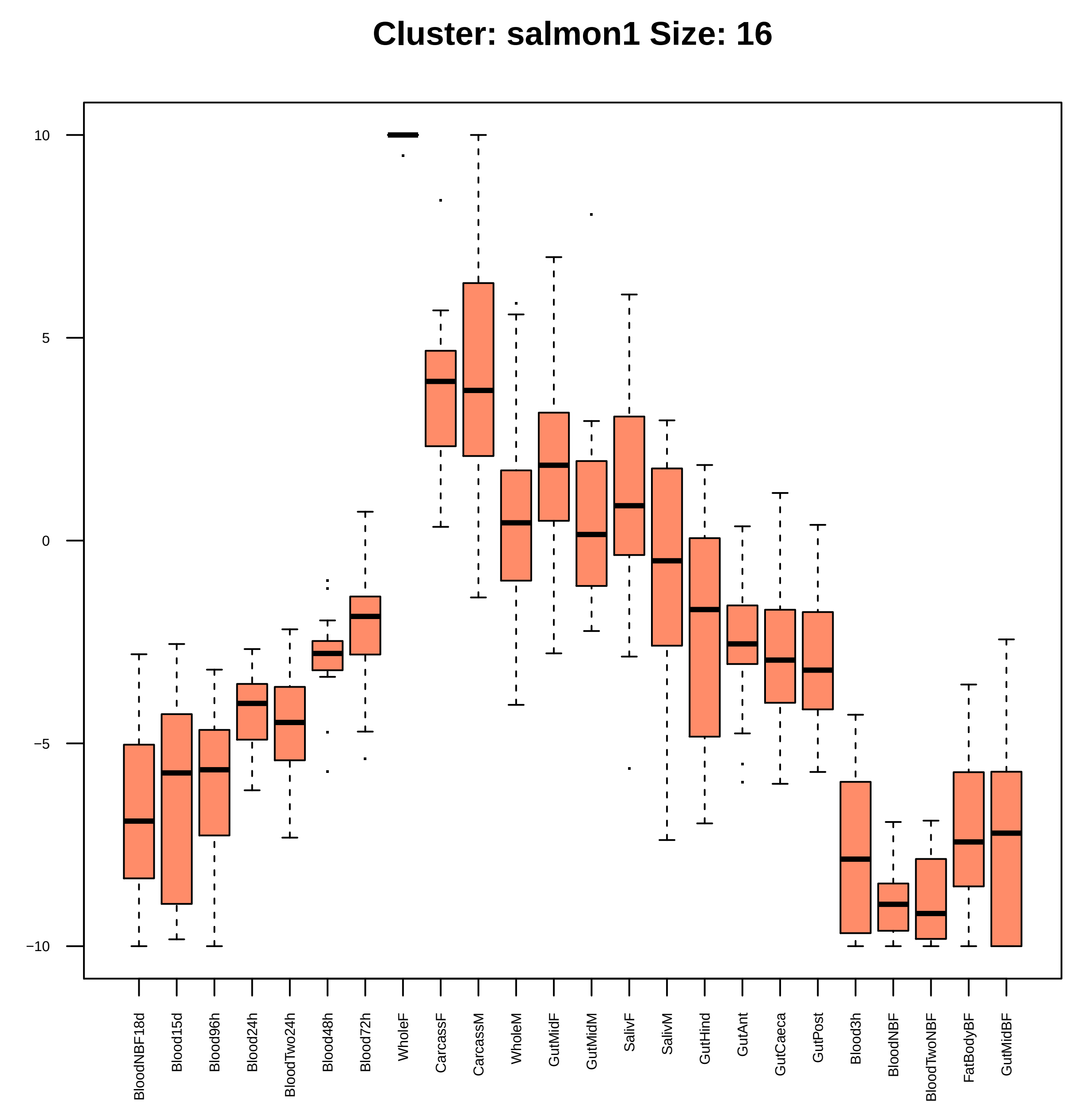

Cluster: salmon1 Size: 16
10
5
0
−5
−10
BloodNBF18d
Blood15d
Blood96h
Blood24h
BloodTwo24h
Blood48h
Blood72h
WholeF
CarcassF
CarcassM
WholeM
GutMidF
GutMidM
SalivF
SalivM
GutHind
GutAnt
GutCaeca
GutPost
Blood3h
BloodNBF
BloodTwoNBF
FatBodyBF
GutMidBF

| | GO.ID | BPCluster: salmon1 Size: 16 | Annotated | Significant | Expected | Rank in ClassicF | Weight01F | ClassicF |
|---|---|---|---|---|---|---|---|---|
| *1* | GO:0006338 | chromatin remodeling | 37 | 2 | 0.05 | 1 | 0.0011 | 0.0011 |
| *13* | GO:0006396 | RNA processing | 267 | 3 | 0.38 | 4 | 0.0932 | 0.0051 |
| *26* | GO:0090304 | nucleic acid metabolic process | 1059 | 5 | 1.49 | 5 | 1.0000 | 0.0088 |

| | GO.ID | MFCluster: salmon1 Size: 16 | Annotated | Significant | Expected | Rank in ClassicF | Weight01F | ClassicF |
|---|---|---|---|---|---|---|---|---|
| *1* | GO:0043138 | 3'−5' DNA helicase activity | 11 | 2 | 0.02 | 1 | 0.00012 | 0.00012 |
| *2* | GO:0004003 | ATP−dependent DNA helicase activity | 21 | 2 | 0.03 | 2 | 0.00044 | 0.00044 |
| *27* | GO:0140097 | catalytic activity, acting on DNA | 81 | 2 | 0.12 | 7 | 1.00000 | 0.00642 |

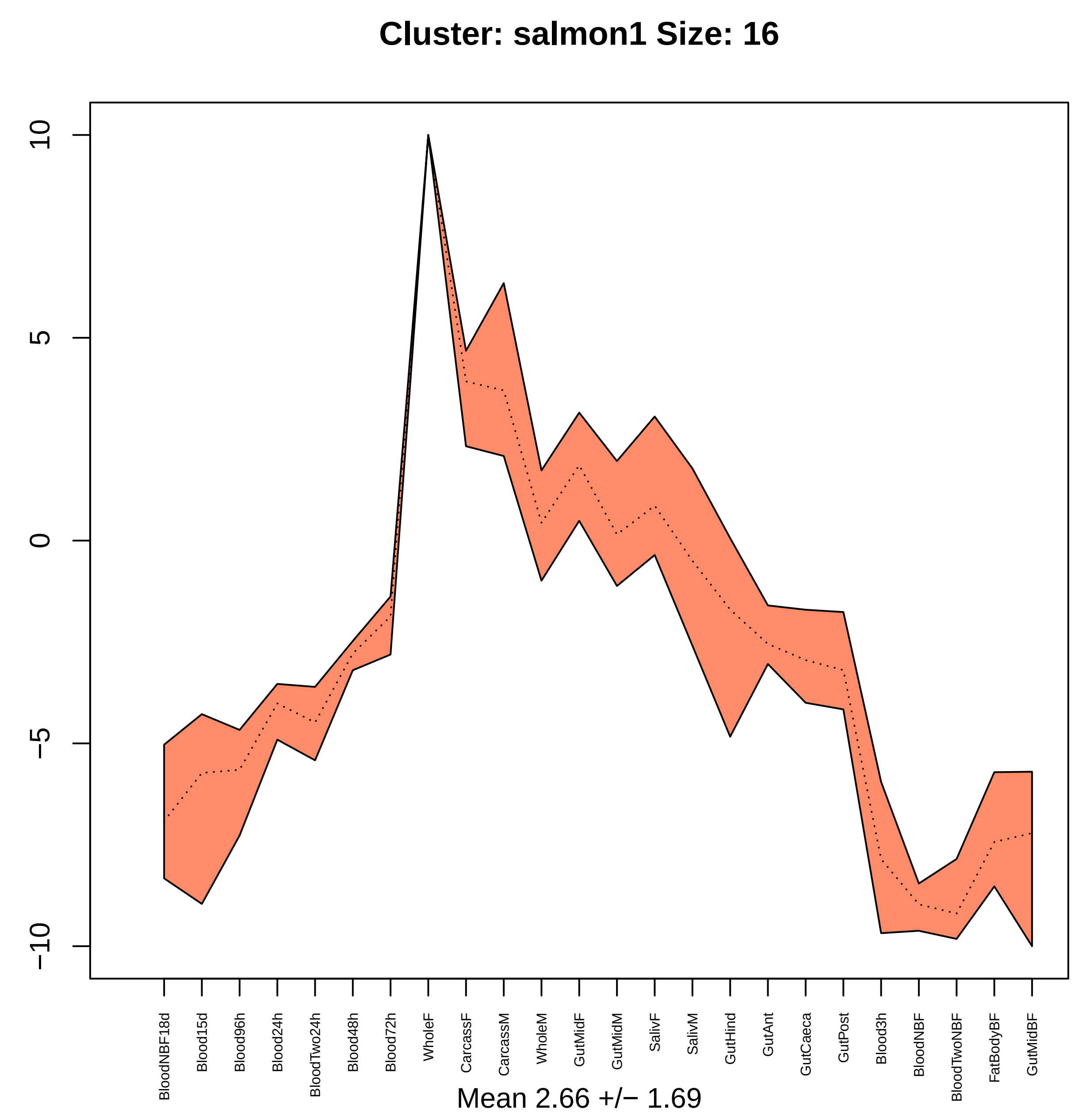
Cluster: salmon1 Size: 16
10
5
0
−5
−10
BloodNBF18d
Blood15d
Blood96h
Blood24h
BloodTwo24h
Blood48h
Blood72h
WholeF
CarcassF
CarcassM
WholeM
GutMidF
GutMidM
SalivF
SalivM
GutHind
GutAnt
GutCaeca
GutPost
Blood3h
BloodNBF
BloodTwoNBF
FatBodyBF
GutMidBF
Mean 2.66 +/− 1.69

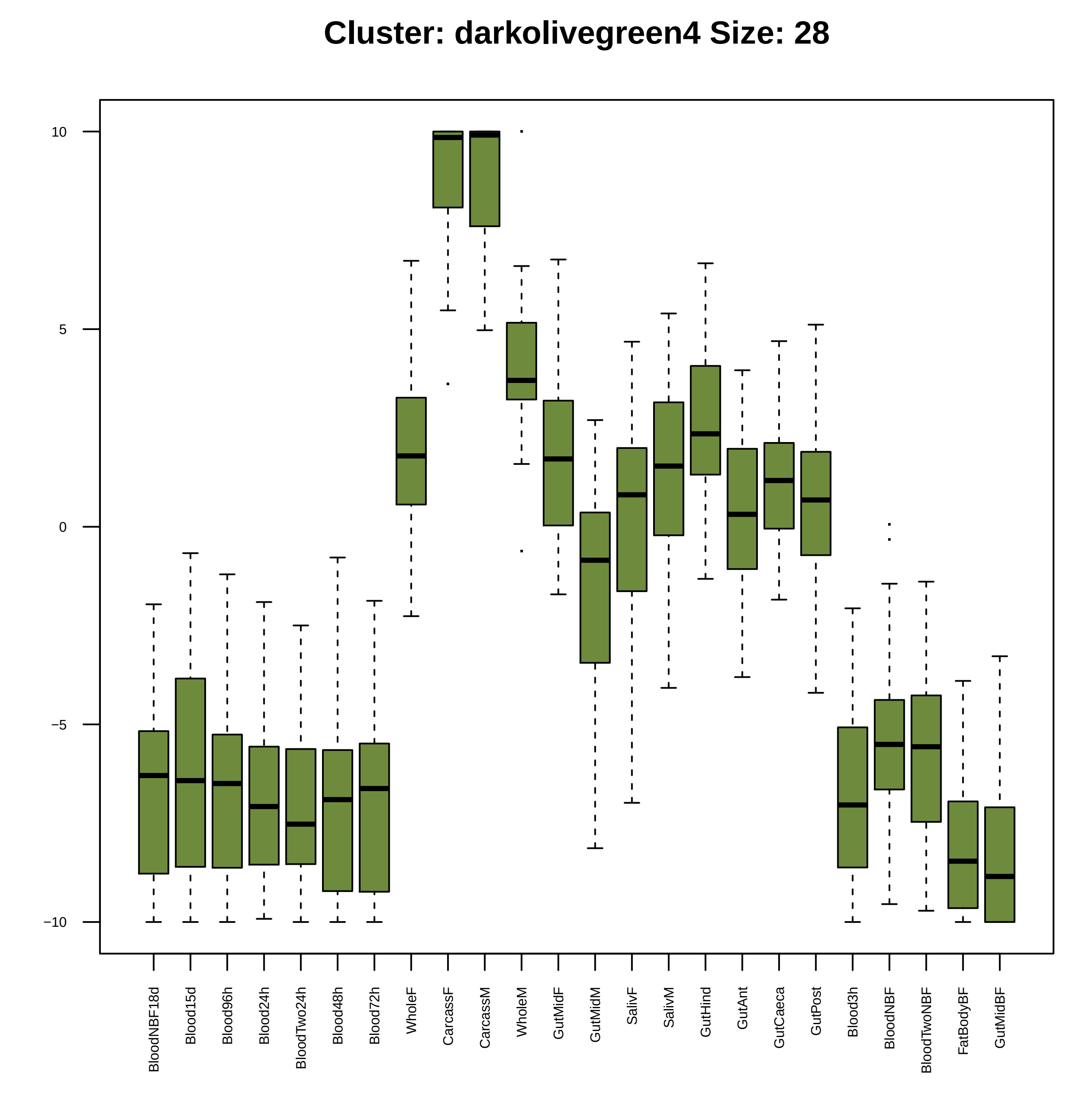
Cluster: darkolivegreen4 Size: 28
10
5
0
−5
−10
BloodNBF18d
Blood15d
Blood96h
Blood24h
BloodTwo24h
Blood48h
Blood72h
WholeF
CarcassF
CarcassM
WholeM
GutMidF
GutMidM
SalivF
SalivM
GutHind
GutAnt
GutCaeca
GutPost
Blood3h
BloodNBF
BloodTwoNBF
FatBodyBF
GutMidBF

| | GO.ID | BPCluster: darkolivegreen4 Size: 28 | Annotated | Significant | Expected | Rank in ClassicF | Weight01F | ClassicF |
|---|---|---|---|---|---|---|---|---|
| *24* | GO:0044249 | cellular biosynthetic process | 1306 | 11 | 3.89 | 12 | 1.000 | 0.00038 |

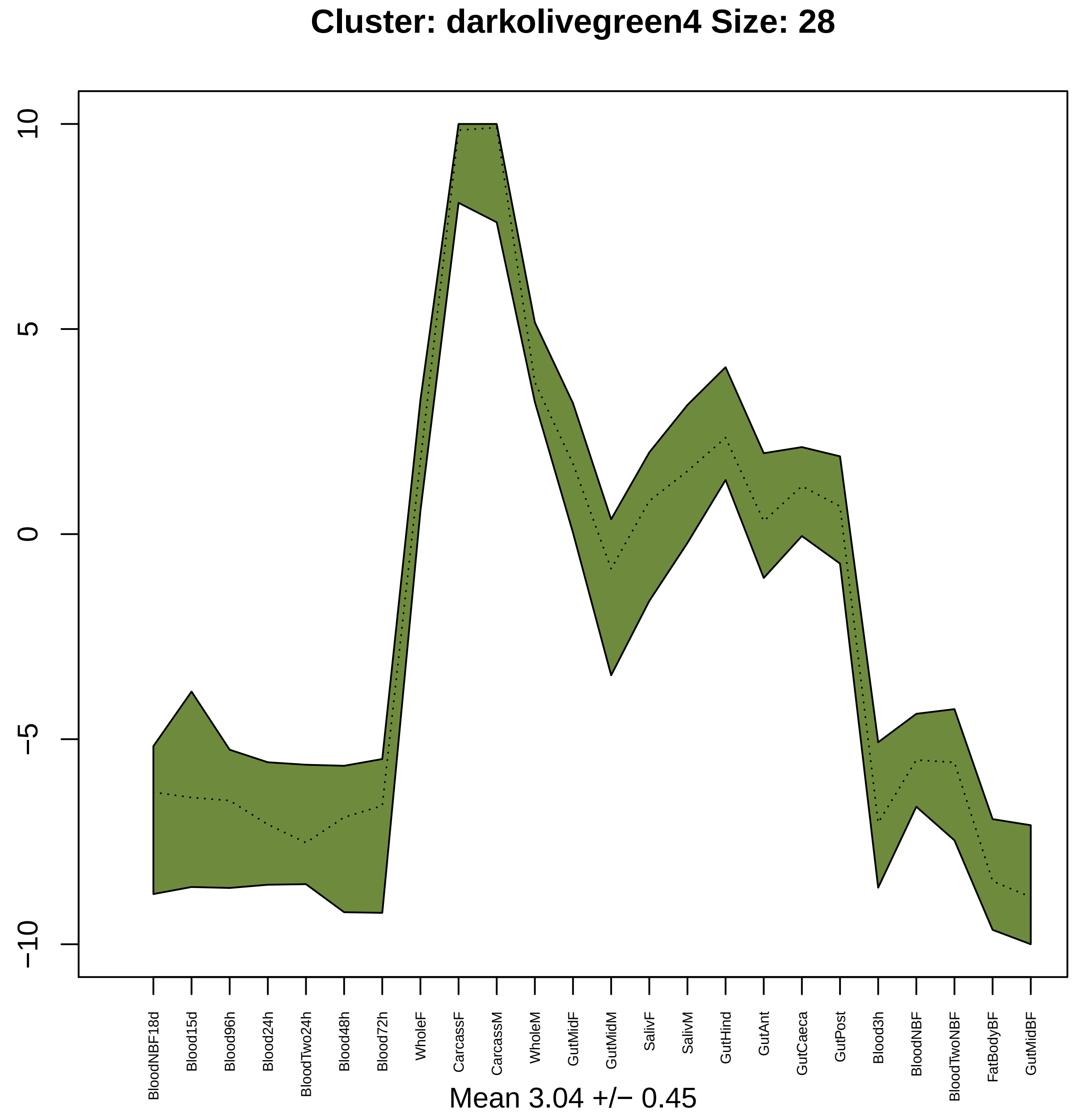

Cluster: darkolivegreen4 Size: 28
10
5
0
−5
−10
BloodNBF18d
Blood15d
Blood96h
Blood24h
BloodTwo24h
Blood48h
Blood72h
WholeF
CarcassF
CarcassM
WholeM
GutMidF
GutMidM
SalivF
SalivM
GutHind
GutAnt
GutCaeca
GutPost
Blood3h
BloodNBF
BloodTwoNBF
FatBodyBF
GutMidBF
Mean 3.04 +/− 0.45

# Cluster: mediumorchid Size: 34

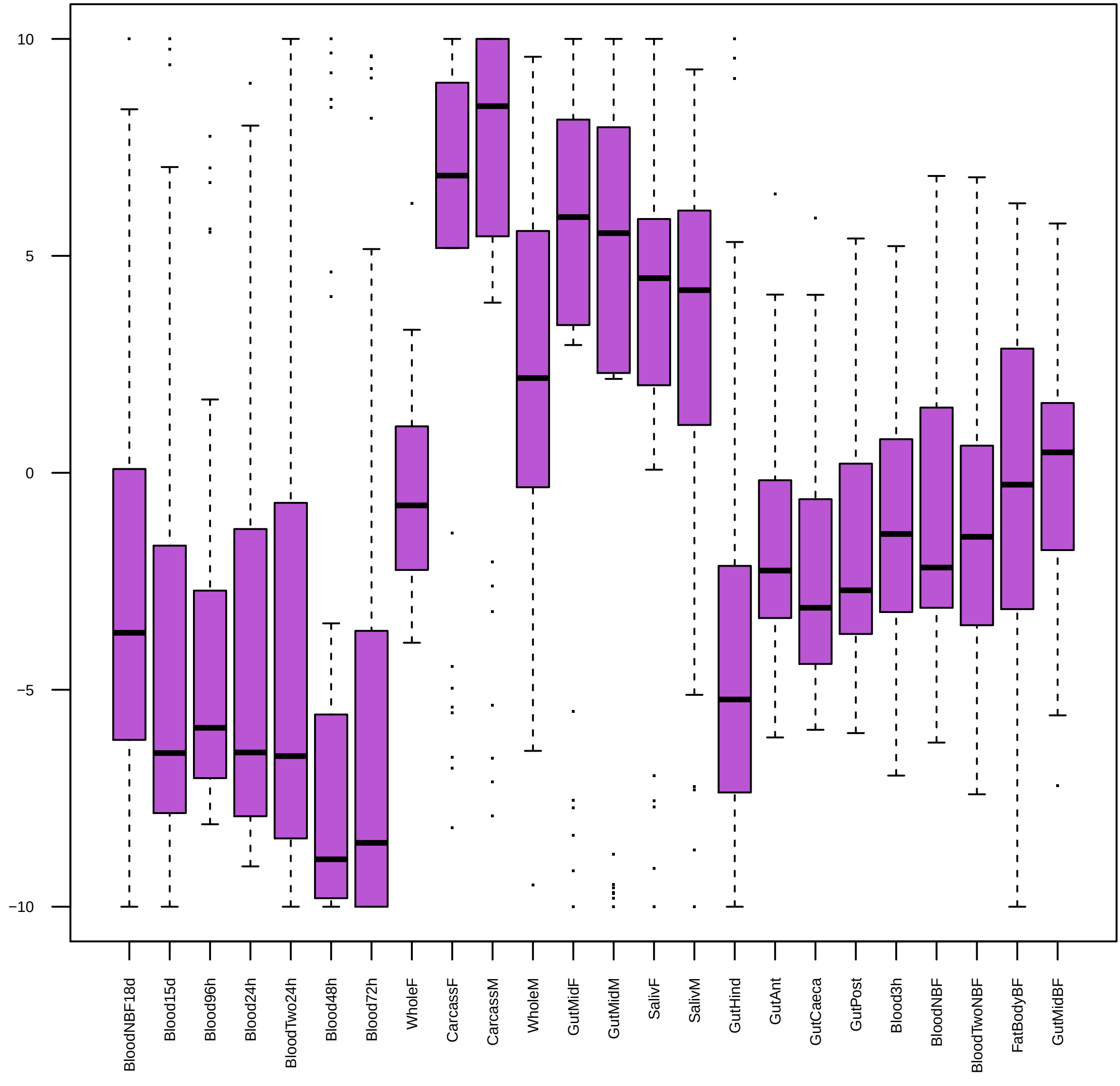

| | GO.ID | BPCluster: mediumorchid Size: 34 | Annotated | Significant | Expected | Rank in ClassicF | Weight01F | ClassicF |
|---|---|---|---|---|---|---|---|---|
| *1* | GO:0007430 | terminal branching, open tracheal system | 12 | 2 | 0.04 | 2 | 0.00067 | 0.00067 |
| *2* | GO:0042059 | negative regulation of epidermal growth ... | 19 | 2 | 0.06 | 9 | 0.00171 | 0.00171 |
| *3* | GO:0008355 | olfactory learning | 26 | 2 | 0.09 | 18 | 0.00320 | 0.00320 |
| *4* | GO:0006633 | fatty acid biosynthetic process | 32 | 2 | 0.11 | 26 | 0.00483 | 0.00483 |
| *8* | GO:0022603 | regulation of anatomical structure morph... | 133 | 3 | 0.44 | 48 | 0.02407 | 0.00896 |
| *9* | GO:0007613 | memory | 34 | 2 | 0.11 | 29 | 0.02493 | 0.00544 |
| *15* | GO:2000241 | regulation of reproductive process | 30 | 2 | 0.10 | 23 | 0.03409 | 0.00425 |
| *20* | GO:0045595 | regulation of cell differentiation | 112 | 3 | 0.37 | 32 | 0.03576 | 0.00557 |

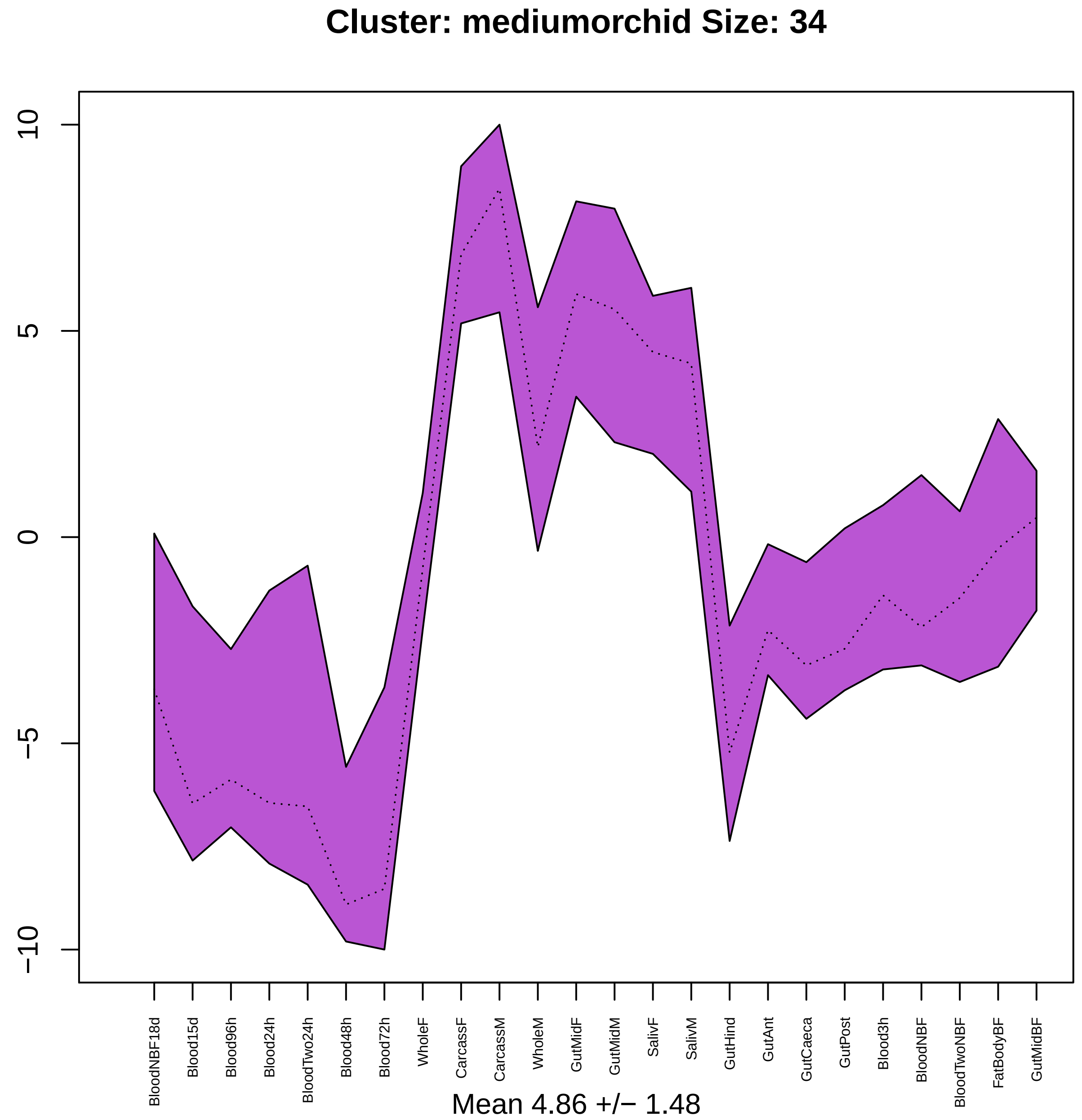

Cluster: mediumorchid Size: 34
10
5
0
−5
−10
BloodNBF18d
Blood15d
Blood96h
Blood24h
BloodTwo24h
Blood48h
Blood72h
WholeF
CarcassF
CarcassM
WholeM
GutMidF
GutMidM
SalivF
SalivM
GutHind
GutAnt
GutCaeca
GutPost
Blood3h
BloodNBF
BloodTwoNBF
FatBodyBF
GutMidBF
Mean 4.86 +/− 1.48

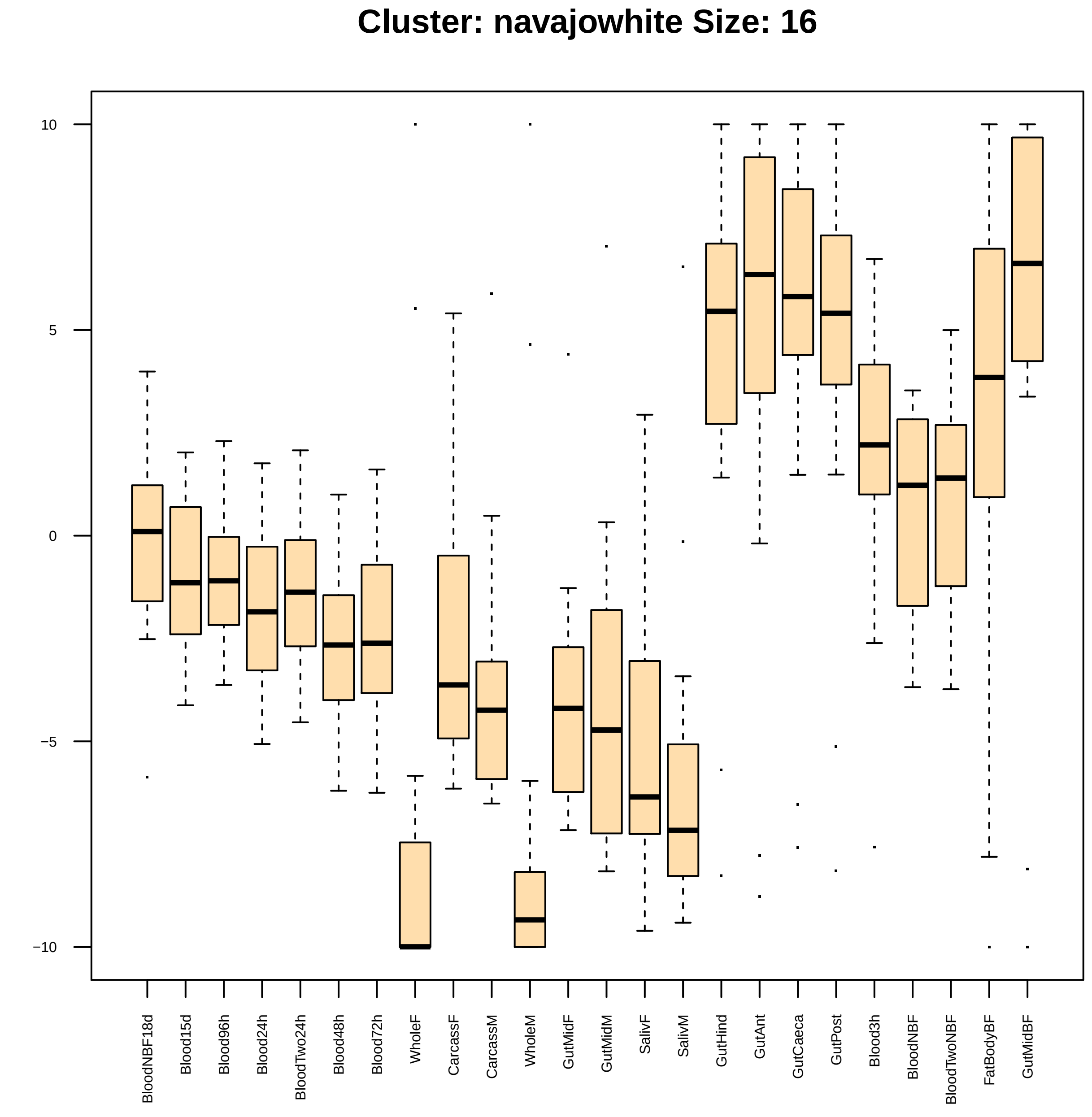
Cluster: navajowhite Size: 16
10
5
0
−5
−10
BloodNBF18d
Blood15d
Blood96h
Blood24h
BloodTwo24h
Blood48h
Blood72h
WholeF
CarcassF
CarcassM
WholeM
GutMidF
GutMidM
SalivF
SalivM
GutHind
GutAnt
GutCaeca
GutPost
Blood3h
BloodNBF
BloodTwoNBF
FatBodyBF
GutMidBF

| | GO.ID | BPCluster: navajowhite Size: 16 | Annotated | Significant | Expected | Rank in ClassicF | Weight01F | ClassicF |
|---|---|---|---|---|---|---|---|---|
| *1* | GO:0006605 | protein targeting | 47 | 2 | 0.07 | 2 | 0.0019 | 0.0019 |
| *2* | GO:0017038 | protein import | 35 | 2 | 0.05 | 1 | 0.0113 | 0.0010 |
| *11* | GO:0072594 | establishment of protein localization to... | 55 | 2 | 0.08 | 3 | 0.0360 | 0.0025 |

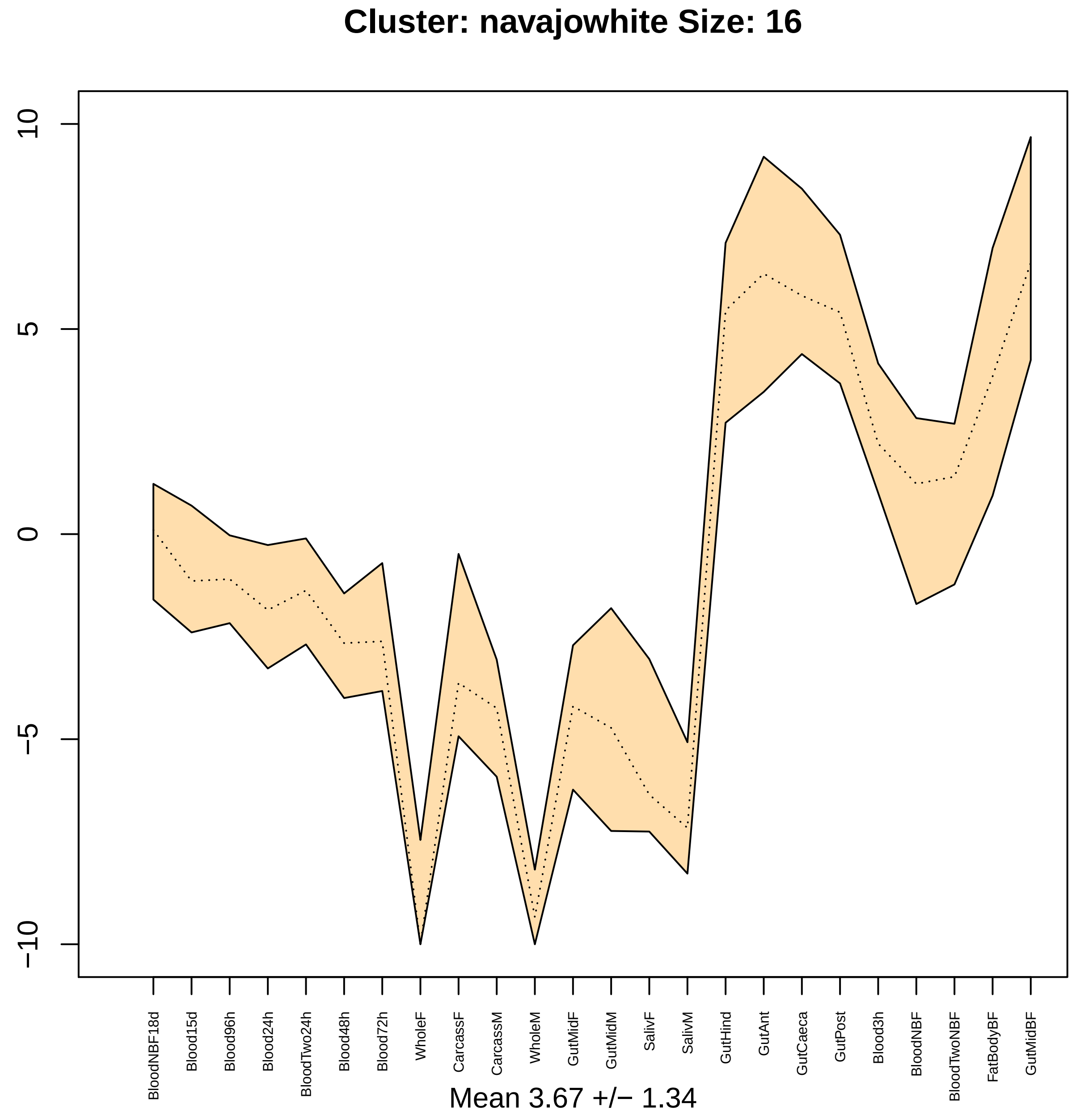
Cluster: navajowhite Size: 16
10
5
0
−5
−10
BloodNBF18d
Blood15d
Blood96h
Blood24h
BloodTwo24h
Blood48h
Blood72h
WholeF
CarcassF
CarcassM
WholeM
GutMidF
GutMidM
SalivF
SalivM
GutHind
GutAnt
GutCaeca
GutPost
Blood3h
BloodNBF
BloodTwoNBF
FatBodyBF
GutMidBF
Mean 3.67 +/− 1.34

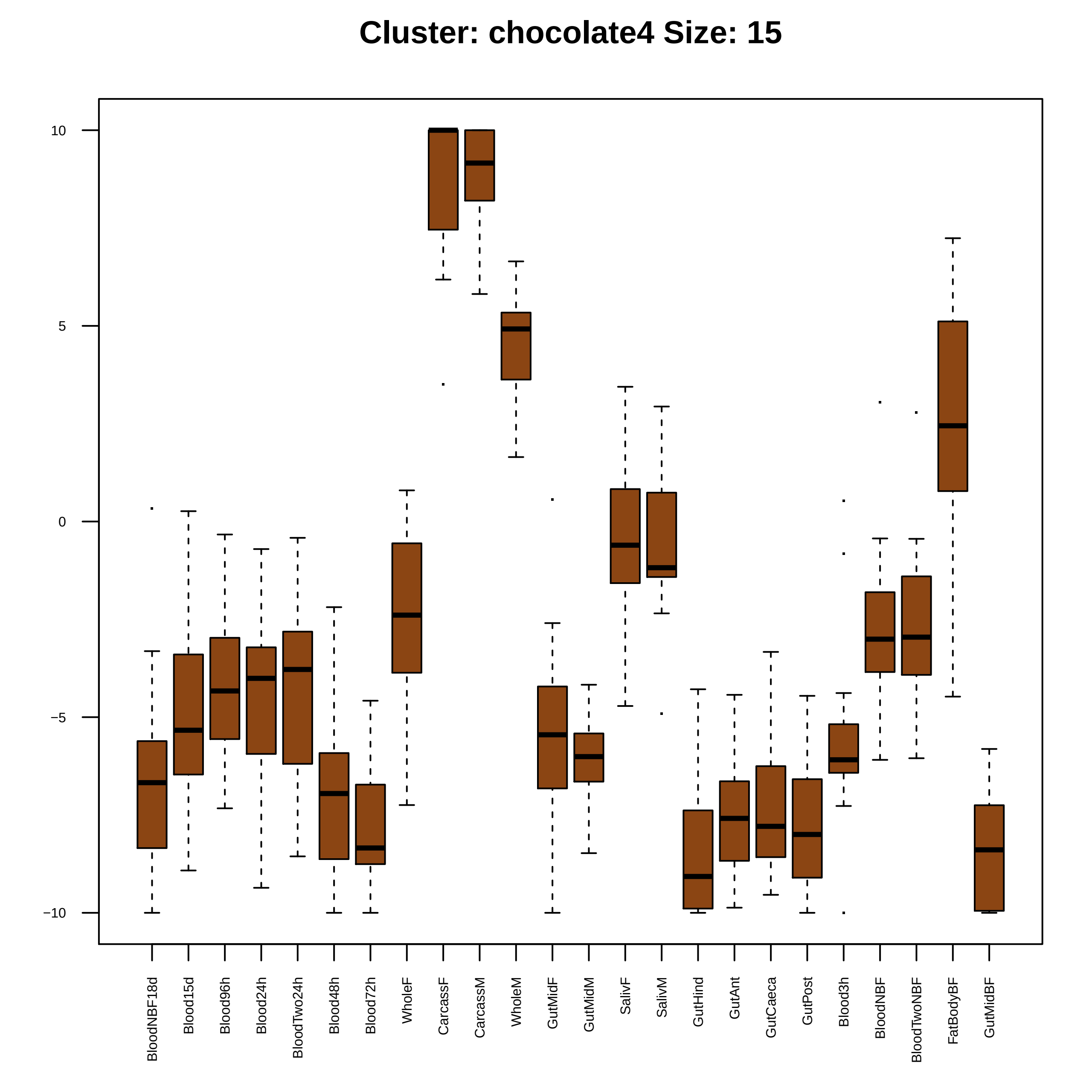

Cluster: chocolate4 Size: 15
10
5
0
−5
−10
BloodNBF18d
Blood15d
Blood96h
Blood24h
BloodTwo24h
Blood48h
Blood72h
WholeF
CarcassF
CarcassM
WholeM
GutMidF
GutMidM
SalivF
SalivM
GutHind
GutAnt
GutCaeca
GutPost
Blood3h
BloodNBF
BloodTwoNBF
FatBodyBF
GutMidBF

| | GO.ID | BPCluster: chocolate4 Size: 15 | Annotated | Significant | Expected | Rank in ClassicF | Weight01F | ClassicF |
|---|---|---|---|---|---|---|---|---|
| *1* | GO:0006508 | proteolysis | 594 | 4 | 0.56 | 1 | 0.0049 | 0.00096 |

| | GO.ID | MFCluster: chocolate4 Size: 15 | Annotated | Significant | Expected | Rank in ClassicF | Weight01F | ClassicF |
|---|---|---|---|---|---|---|---|---|
| *1* | GO:0004252 | serine−type endopeptidase activity | 252 | 3 | 0.35 | 2 | 0.0042 | 0.0042 |
| *3* | GO:0008233 | peptidase activity | 500 | 4 | 0.69 | 1 | 0.0308 | 0.0034 |
| *9* | GO:0008236 | serine−type peptidase activity | 278 | 3 | 0.39 | 3 | 1.0000 | 0.0056 |
| *20* | GO:0017171 | serine hydrolase activity | 279 | 3 | 0.39 | 4 | 1.0000 | 0.0056 |

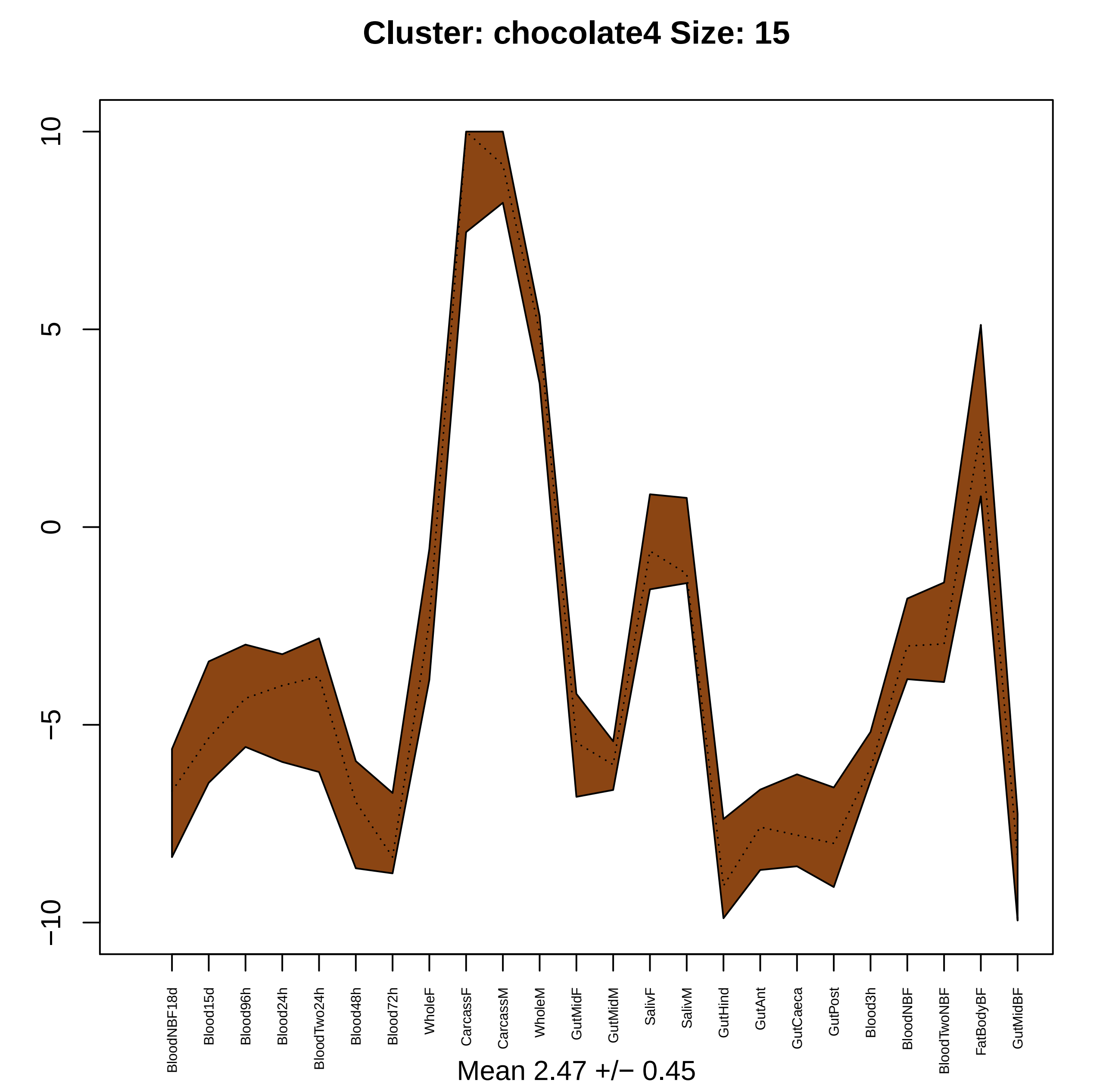

Cluster: chocolate4 Size: 15
10
5
0
−5
−10
BloodNBF18d
Blood15d
Blood96h
Blood24h
BloodTwo24h
Blood48h
Blood72h
WholeF
CarcassF
CarcassM
WholeM
GutMidF
GutMidM
SalivF
SalivM
GutHind
GutAnt
GutCaeca
GutPost
Blood3h
BloodNBF
BloodTwoNBF
FatBodyBF
GutMidBF
Mean 2.47 +/− 0.45

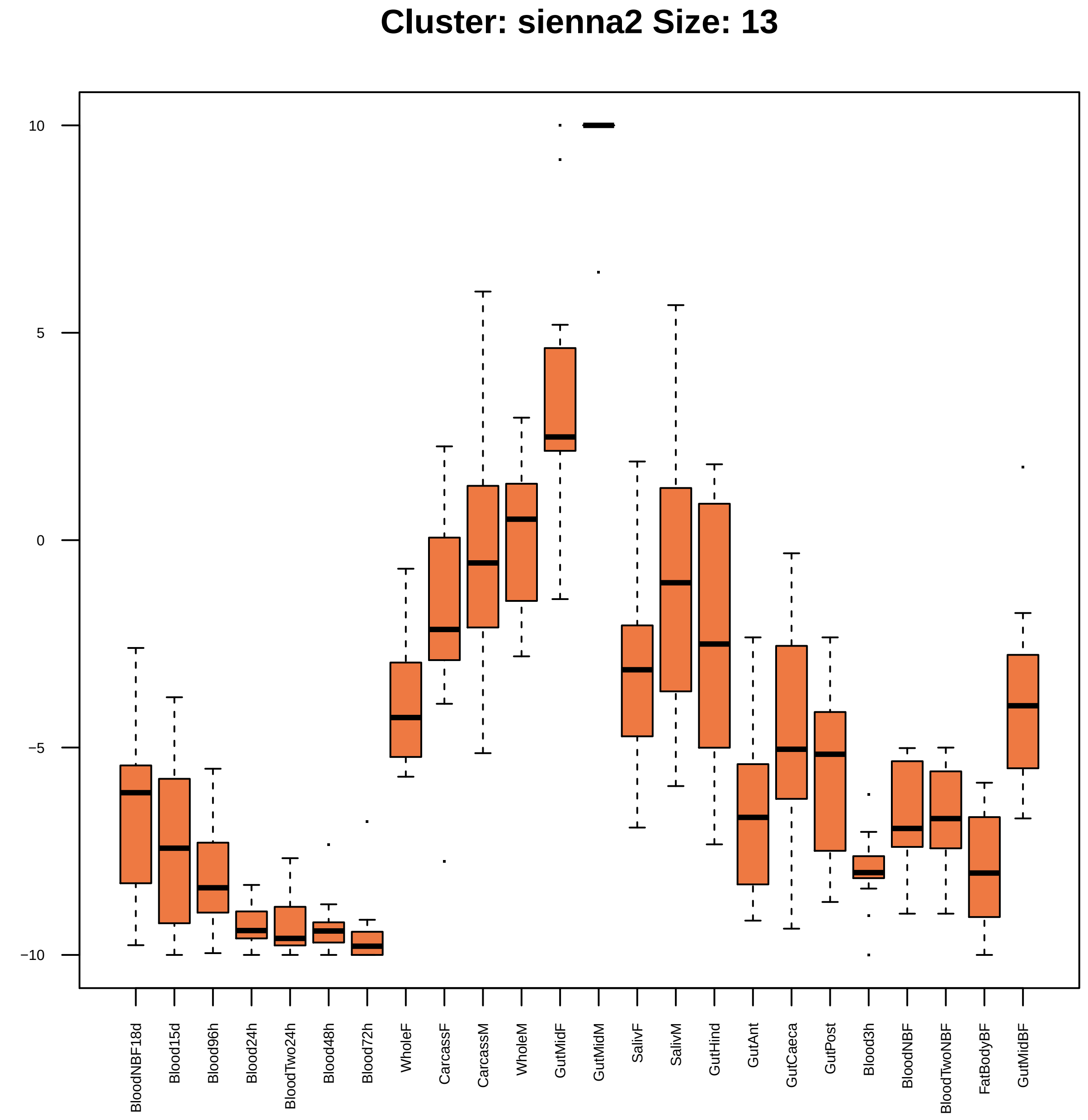

Cluster: sienna2 Size: 13
10
5
0
−5
−10
BloodNBF18d
Blood15d
Blood96h
Blood24h
BloodTwo24h
Blood48h
Blood72h
WholeF
CarcassF
CarcassM
WholeM
GutMidF
GutMidM
SalivF
SalivM
GutHind
GutAnt
GutCaeca
GutPost
Blood3h
BloodNBF
BloodTwoNBF
FatBodyBF
GutMidBF

| | GO.ID | BPCluster: sienna2 Size: 13 | Annotated | Significant | Expected | Rank in ClassicF | Weight01F | ClassicF |
|---|---|---|---|---|---|---|---|---|
| *1* | GO:0035725 | sodium ion transmembrane transport | 46 | 2 | 0.08 | 2 | 0.0027 | 0.00269 |
| *4* | GO:0055085 | transmembrane transport | 469 | 5 | 0.81 | 1 | 0.0248 | 0.00067 |

| | GO.ID | MFCluster: sienna2 Size: 13 | Annotated | Significant | Expected | Rank in ClassicF | Weight01F | ClassicF |
|---|---|---|---|---|---|---|---|---|
| *8* | GO:0015370 | solute:sodium symporter activity | 31 | 2 | 0.05 | 3 | 0.023 | 0.00096 |
| *12* | GO:0022857 | transmembrane transporter activity | 442 | 5 | 0.67 | 1 | 0.066 | 0.00029 |
| *28* | GO:0015291 | secondary active transmembrane transport... | 60 | 2 | 0.09 | 7 | 1.000 | 0.00357 |
| *30* | GO:0015293 | symporter activity | 37 | 2 | 0.06 | 5 | 1.000 | 0.00137 |

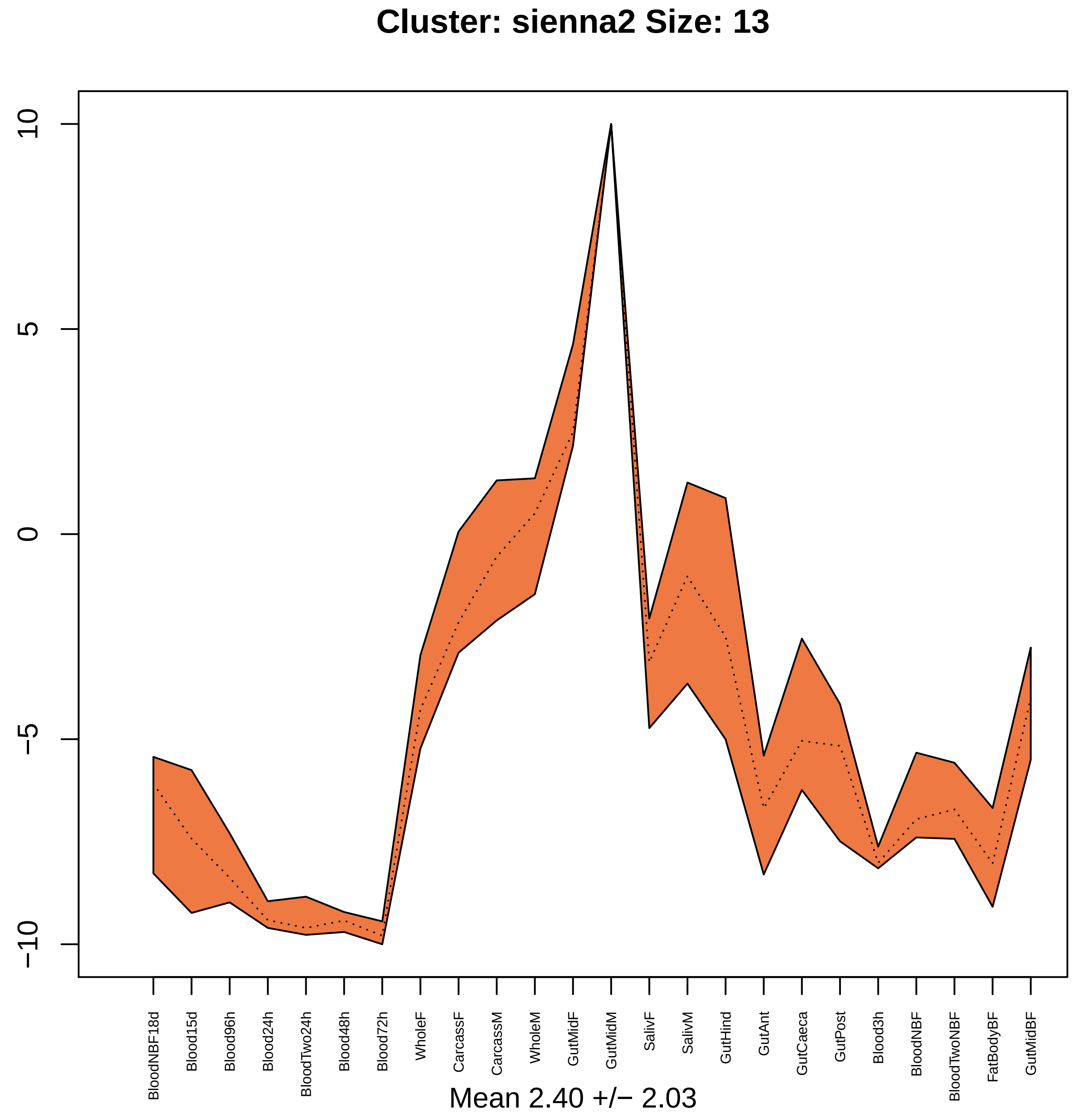

Cluster: sienna2 Size: 13
10
5
0
−5
−10
BloodNBF18d
Blood15d
Blood96h
Blood24h
BloodTwo24h
Blood48h
Blood72h
WholeF
CarcassF
CarcassM
WholeM
GutMidF
GutMidM
SalivF
SalivM
GutHind
GutAnt
GutCaeca
GutPost
Blood3h
BloodNBF
BloodTwoNBF
FatBodyBF
GutMidBF
Mean 2.40 +/− 2.03

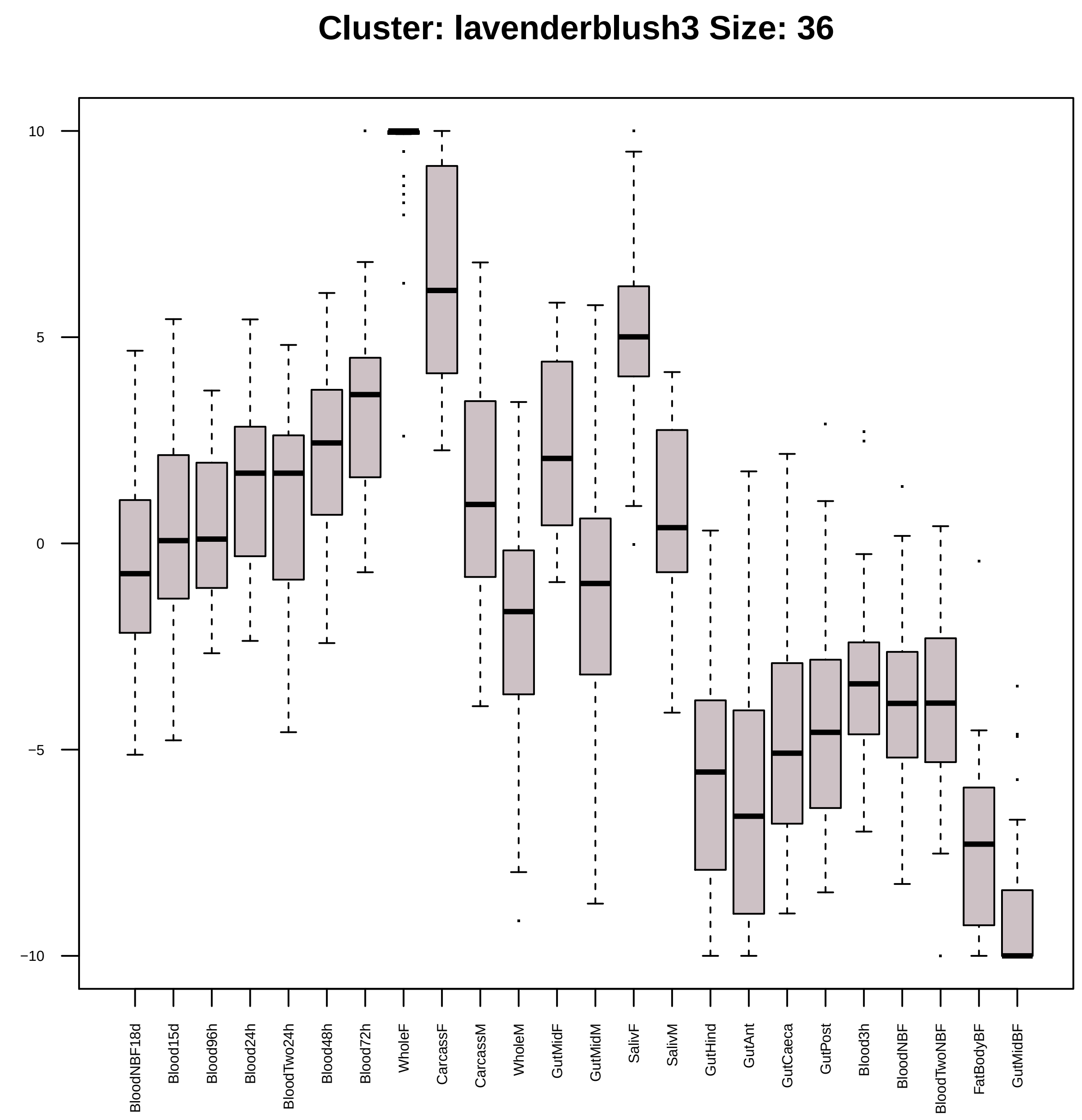
Cluster: lavenderblush3 Size: 36
10
5
0
−5
−10
BloodNBF18d
Blood15d
Blood96h
Blood24h
BloodTwo24h
Blood48h
Blood72h
WholeF
CarcassF
CarcassM
WholeM
GutMidF
GutMidM
SalivF
SalivM
GutHind
GutAnt
GutCaeca
GutPost
Blood3h
BloodNBF
BloodTwoNBF
FatBodyBF
GutMidBF

| | GO.ID | BPCluster: lavenderblush3 Size: 36 | Annotated | Significant | Expected | Rank in ClassicF | Weight01F | ClassicF |
|---|---|---|---|---|---|---|---|---|
| 3 | GO:0032508 | DNA duplex unwinding | 18 | 2 | 0.08 | 24 | 0.0031 | 0.00312 |
| 4 | GO:0010972 | negative regulation of G2/M transition o... | 44 | 2 | 0.21 | 37 | 0.0046 | 0.01791 |
| 5 | GO:0042254 | ribosome biogenesis | 64 | 4 | 0.30 | 13 | 0.0076 | 0.00021 |
| 6 | GO:0000398 | mRNA splicing, via spliceosome | 134 | 4 | 0.63 | 26 | 0.0095 | 0.00334 |
| 22 | GO:0006397 | mRNA processing | 164 | 5 | 0.77 | 20 | 0.1105 | 0.00090 |

| | GO.ID | MFCluster: lavenderblush3 Size: 36 | Annotated | Significant | Expected | Rank in ClassicF | Weight01F | ClassicF |
|---|---|---|---|---|---|---|---|---|
| *1* | GO:0003729 | mRNA binding | 99 | 4 | 0.45 | 6 | 0.0010 | 0.0010 |
| *2* | GO:0003676 | nucleic acid binding | 1152 | 17 | 5.27 | 1 | 0.0039 | 2.3e−06 |
| *3* | GO:0004003 | ATP−dependent DNA helicase activity | 21 | 2 | 0.10 | 7 | 0.0040 | 0.0040 |
| *4* | GO:0003899 | DNA−directed 5'−3' RNA polymerase activi... | 27 | 2 | 0.12 | 10 | 0.0066 | 0.0066 |
| *5* | GO:0003723 | RNA binding | 307 | 8 | 1.41 | 2 | 0.0079 | 5.4e−05 |

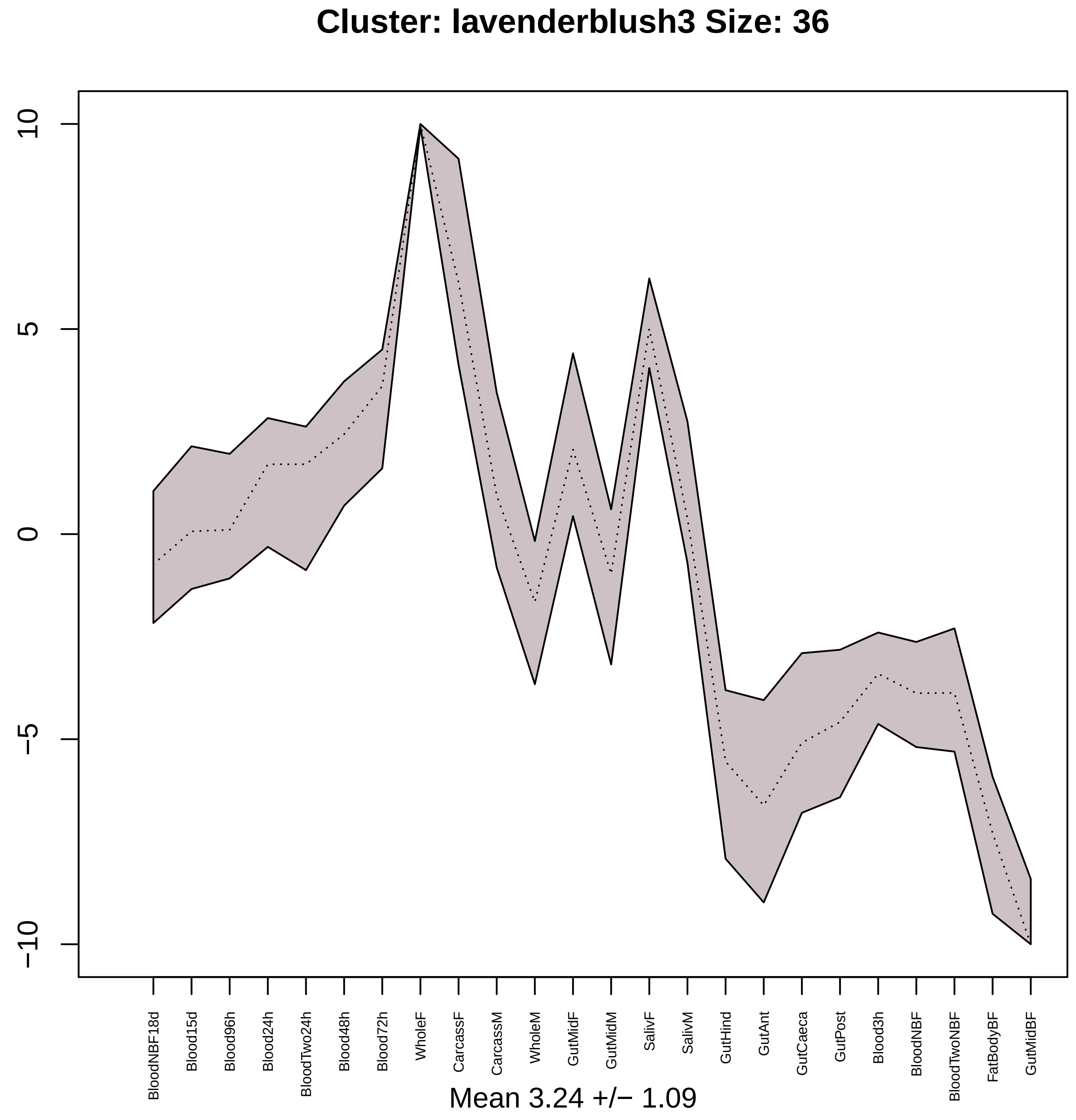

Cluster: lavenderblush3 Size: 36
10
5
0
−5
−10
BloodNBF18d
Blood15d
Blood96h
Blood24h
BloodTwo24h
Blood48h
Blood72h
WholeF
CarcassF
CarcassM
WholeM
GutMidF
GutMidM
SalivF
SalivM
GutHind
GutAnt
GutCaeca
GutPost
Blood3h
BloodNBF
BloodTwoNBF
FatBodyBF
GutMidBF
Mean 3.24 +/− 1.09

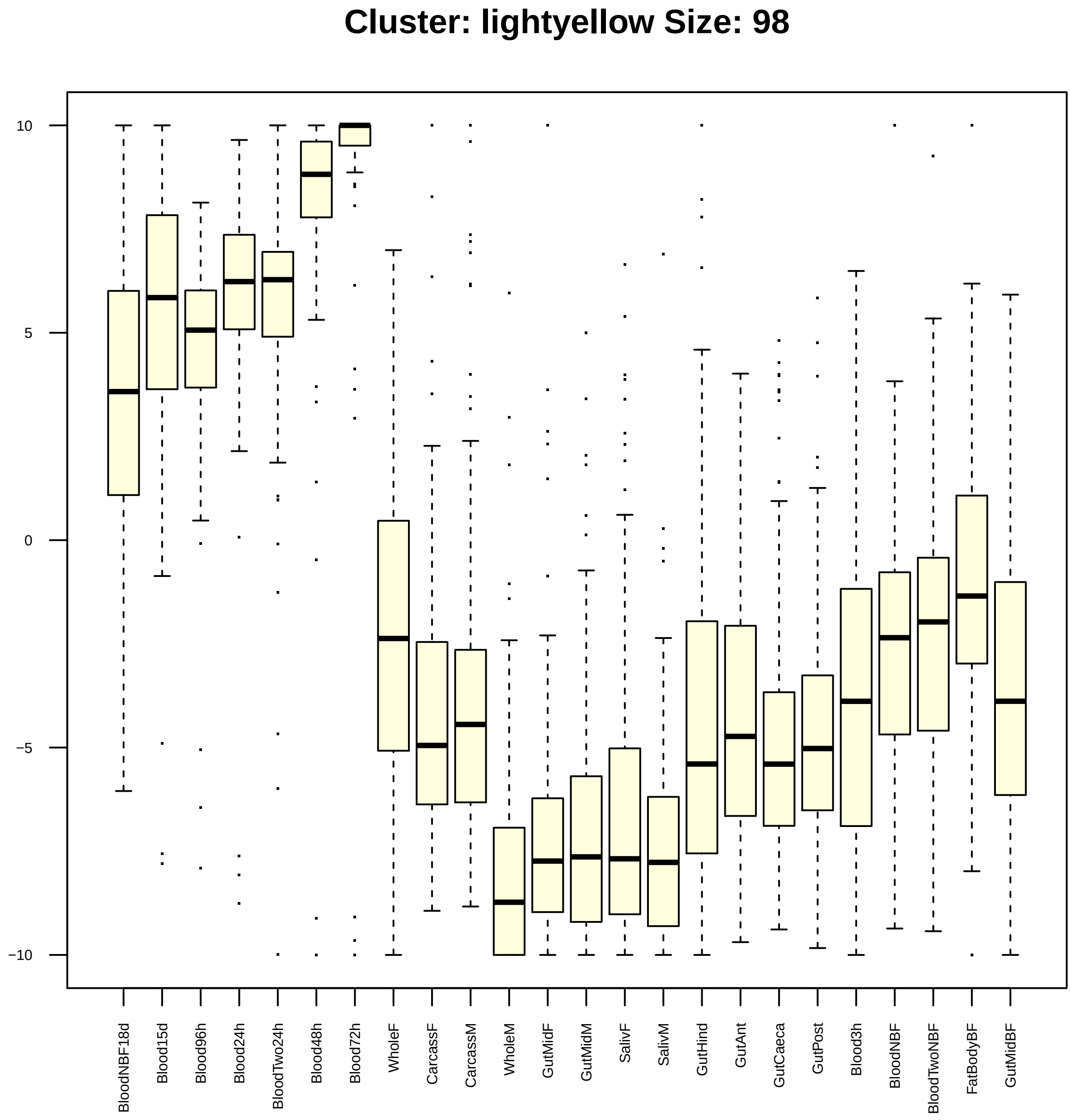
Cluster: lightyellow Size: 98
10
5
0
−5
−10
BloodNBF18d
Blood15d
Blood96h
Blood24h
BloodTwo24h
Blood48h
Blood72h
WholeF
CarcassF
CarcassM
WholeM
GutMidF
GutMidM
SalivF
SalivM
GutHind
GutAnt
GutCaeca
GutPost
Blood3h
BloodNBF
BloodTwoNBF
FatBodyBF
GutMidBF

| | GO.ID | BPCluster: lightyellow Size: 98 | Annotated | Significant | Expected | Rank in ClassicF | Weight01F | ClassicF |
|---|---|---|---|---|---|---|---|---|
| 1 | GO:0032006 | regulation of TOR signaling | 10 | 3 | 0.10 | 29 | 0.00010 | 0.00010 |
| 2 | GO:0046626 | regulation of insulin receptor signaling... | 12 | 3 | 0.12 | 36 | 0.00018 | 0.00018 |
| 3 | GO:0016476 | regulation of embryonic cell shape | 18 | 3 | 0.18 | 70 | 0.00064 | 0.00064 |
| 4 | GO:0007447 | imaginal disc pattern formation | 47 | 3 | 0.46 | 180 | 0.00133 | 0.01047 |
| 5 | GO:0017148 | negative regulation of translation | 24 | 3 | 0.23 | 90 | 0.00153 | 0.00153 |
| 6 | GO:0008285 | negative regulation of cell proliferatio... | 30 | 3 | 0.29 | 114 | 0.00295 | 0.00295 |
| 7 | GO:0007446 | imaginal disc growth | 28 | 3 | 0.27 | 104 | 0.00388 | 0.00241 |
| 8 | GO:0030713 | ovarian follicle cell stalk formation | 10 | 2 | 0.10 | 126 | 0.00398 | 0.00398 |
| 9 | GO:0022403 | cell cycle phase | 10 | 2 | 0.10 | 127 | 0.00398 | 0.00398 |
| 10 | GO:1902532 | negative regulation of intracellular sig... | 34 | 3 | 0.33 | 131 | 0.00423 | 0.00423 |
| 11 | GO:0009954 | proximal/distal pattern formation | 11 | 2 | 0.11 | 138 | 0.00483 | 0.00483 |
| 12 | GO:0035099 | hemocyte migration | 11 | 2 | 0.11 | 139 | 0.00483 | 0.00483 |
| 13 | GO:0048518 | positive regulation of biological proces... | 430 | 14 | 4.18 | 20 | 0.00507 | 4.7e−05 |
| 14 | GO:1902533 | positive regulation of intracellular sig... | 41 | 4 | 0.40 | 69 | 0.00546 | 0.00063 |
| 15 | GO:0016322 | neuron remodeling | 12 | 2 | 0.12 | 149 | 0.00577 | 0.00577 |
| 16 | GO:0030178 | negative regulation of Wnt signaling pat... | 13 | 2 | 0.13 | 160 | 0.00677 | 0.00677 |
| 20 | GO:0007423 | sensory organ development | 219 | 8 | 2.13 | 84 | 0.01517 | 0.00116 |
| 21 | GO:0000003 | reproduction | 452 | 11 | 4.39 | 125 | 0.01747 | 0.00376 |
| 24 | GO:0007420 | brain development | 54 | 4 | 0.53 | 94 | 0.02468 | 0.00178 |
| 30 | GO:0048477 | oogenesis | 263 | 8 | 2.56 | 123 | 0.02826 | 0.00368 |

| | GO.ID | MFCluster: lightyellow Size: 98 | Annotated | Significant | Expected | Rank in ClassicF | Weight01F | ClassicF |
|---|---|---|---|---|---|---|---|---|
| *1* | GO:0005515 | protein binding | 2143 | 43 | 23.48 | 2 | 0.00028 | 3.6e−06 |
| *2* | GO:0043021 | ribonucleoprotein complex binding | 14 | 2 | 0.15 | 7 | 0.00991 | 0.00991 |
| *11* | GO:0044877 | protein−containing complex binding | 50 | 4 | 0.55 | 4 | 0.05636 | 0.00210 |
| *16* | GO:0003676 | nucleic acid binding | 1152 | 25 | 12.62 | 3 | 0.09265 | 0.00038 |

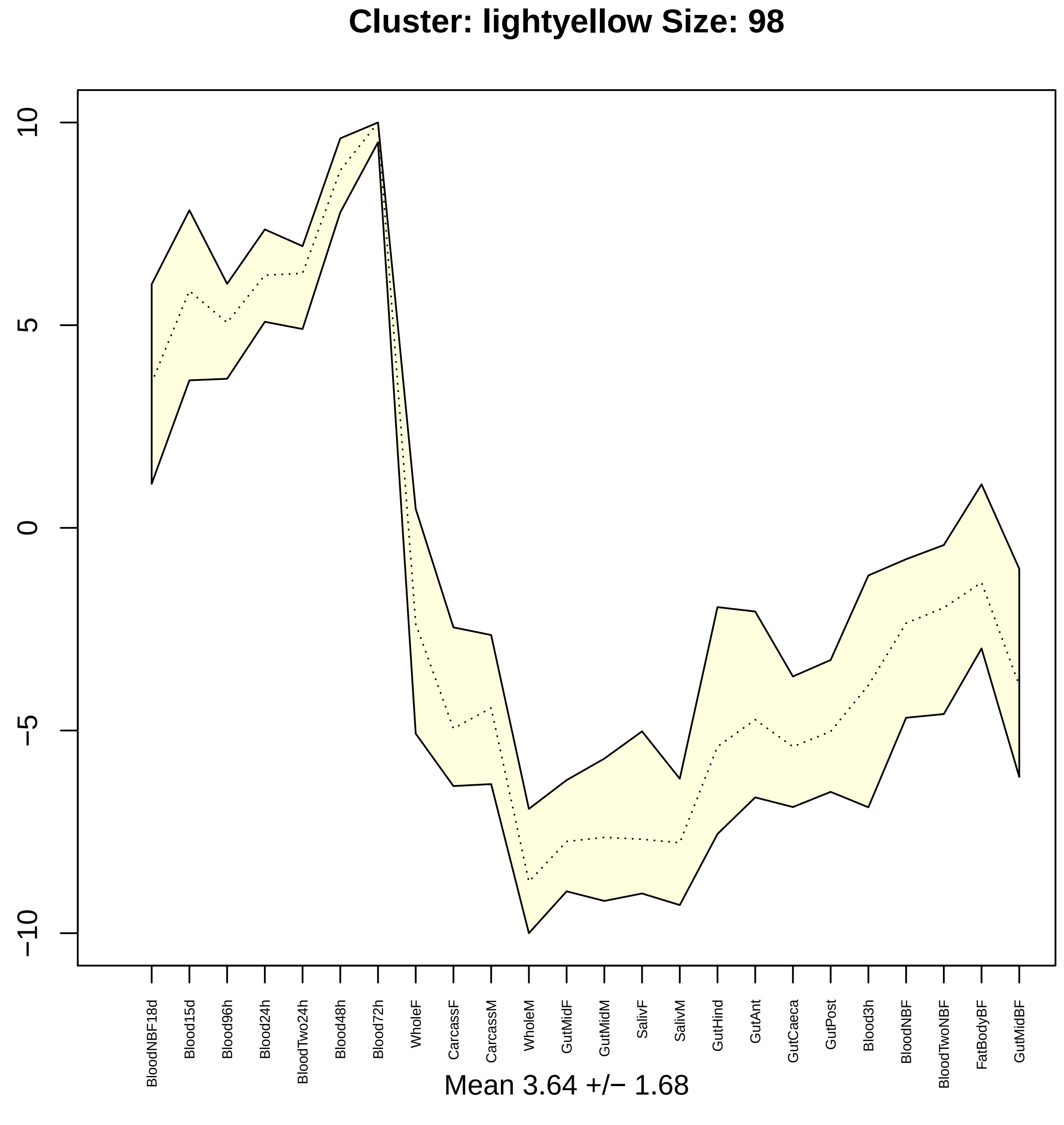

Cluster: lightyellow Size: 98
10
5
0
−5
−10
BloodNBF18d
Blood15d
Blood96h
Blood24h
BloodTwo24h
Blood48h
Blood72h
WholeF
CarcassF
CarcassM
WholeM
GutMidF
GutMidM
SalivF
SalivM
GutHind
GutAnt
GutCaeca
GutPost
Blood3h
BloodNBF
BloodTwoNBF
FatBodyBF
GutMidBF
Mean 3.64 +/− 1.68

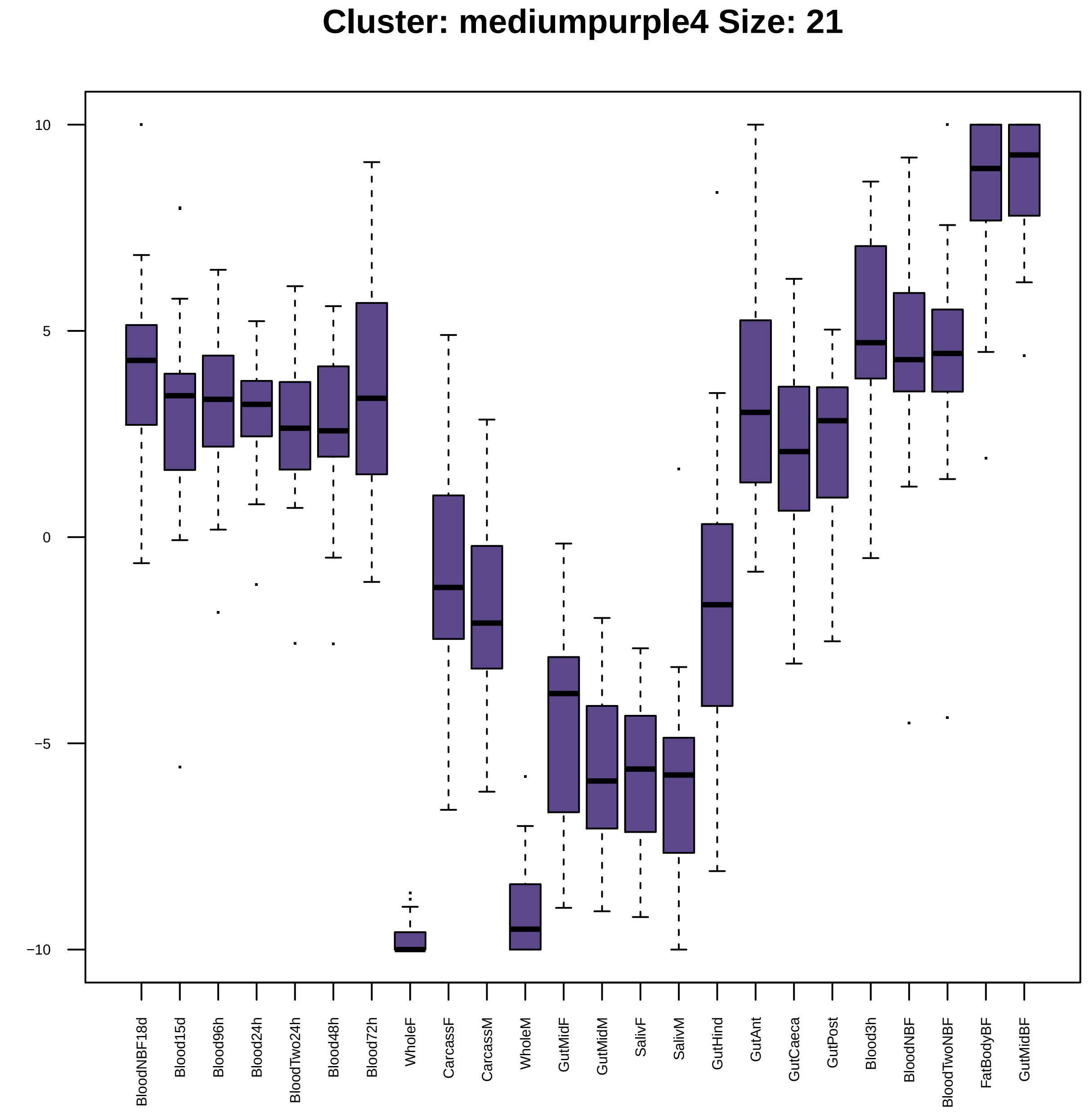

Cluster: mediumpurple4 Size: 21
10
5
0
−5
−10
BloodNBF18d
Blood15d
Blood96h
Blood24h
BloodTwo24h
Blood48h
Blood72h
WholeF
CarcassF
CarcassM
WholeM
GutMidF
GutMidM
SalivF
SalivM
GutHind
GutAnt
GutCaeca
GutPost
Blood3h
BloodNBF
BloodTwoNBF
FatBodyBF
GutMidBF

| | GO.ID | BPCluster: mediumpurple4 Size: 21 | Annotated | Significant | Expected | Rank in ClassicF | Weight01F | ClassicF |
|---|---|---|---|---|---|---|---|---|
| *23* | GO:0016192 | vesicle−mediated transport | 333 | 3 | 0.47 | 2 | 0.114 | 0.0094 |

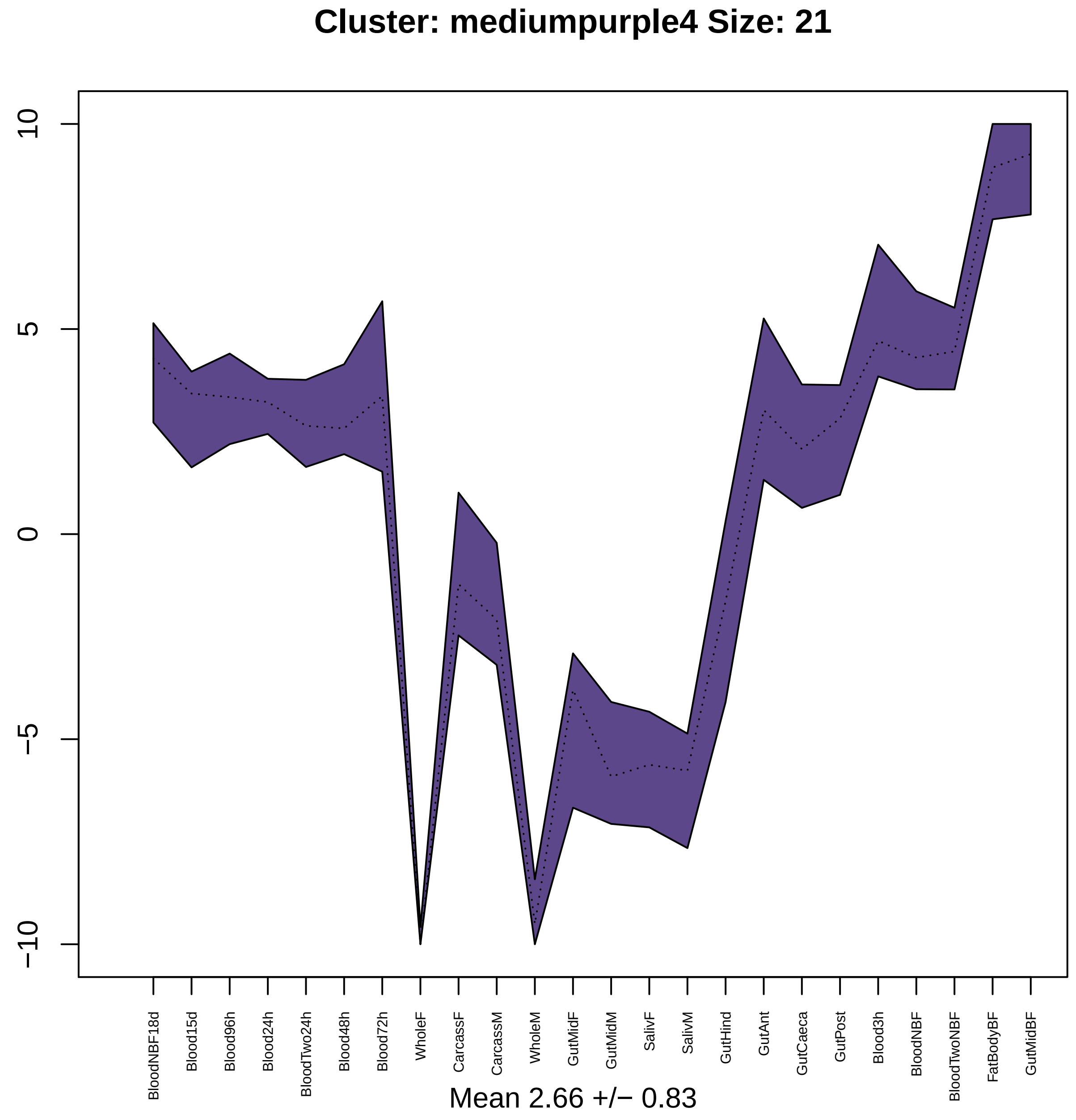

Cluster: mediumpurple4 Size: 21
10
5
0
−5
−10
BloodNBF18d
Blood15d
Blood96h
Blood24h
BloodTwo24h
Blood48h
Blood72h
WholeF
CarcassF
CarcassM
WholeM
GutMidF
GutMidM
SalivF
SalivM
GutHind
GutAnt
GutCaeca
GutPost
Blood3h
BloodNBF
BloodTwoNBF
FatBodyBF
GutMidBF
Mean 2.66 +/− 0.83

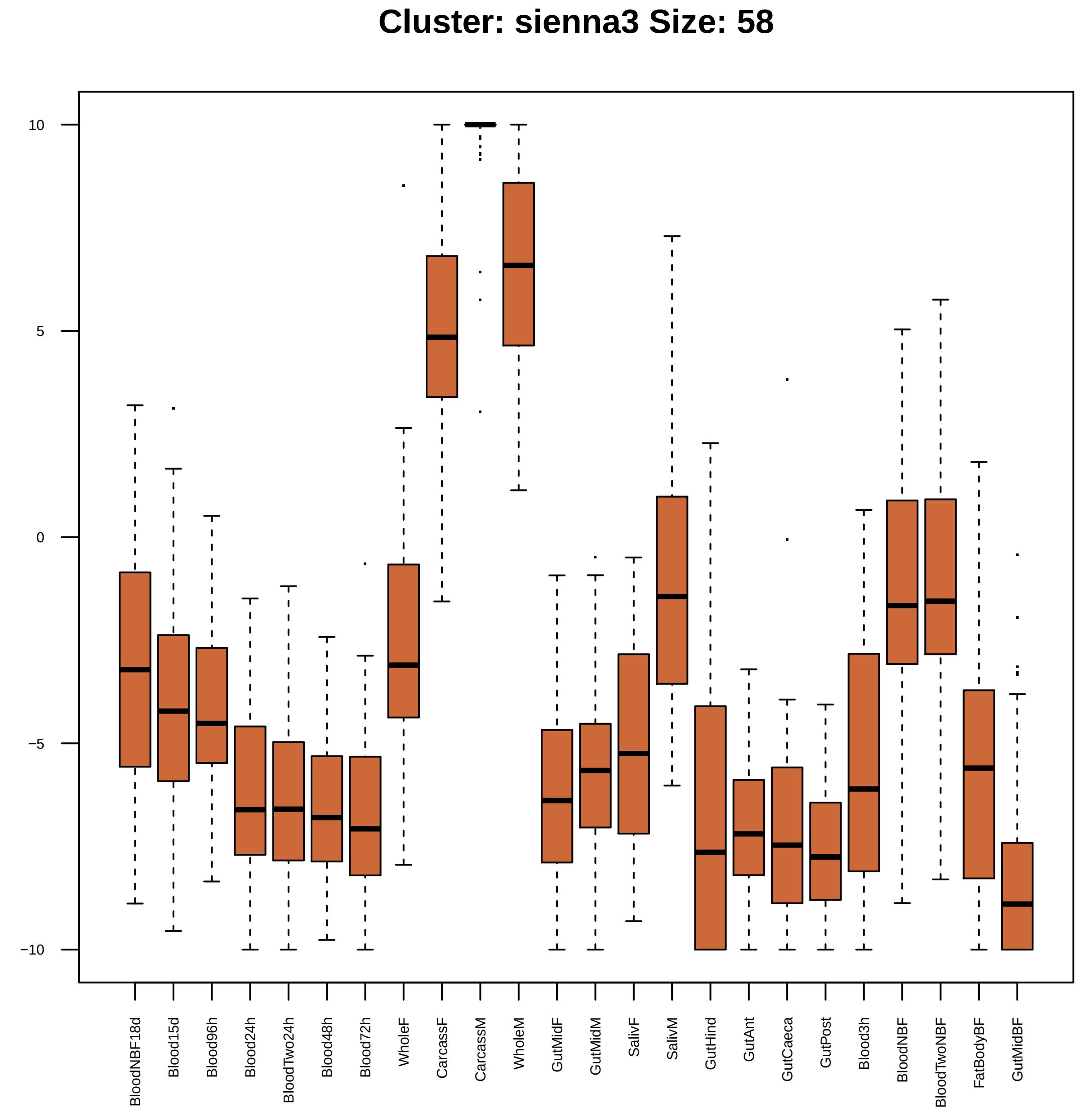

Cluster: sienna3 Size: 58
10
5
0
−5
−10
BloodNBF18d
Blood15d
Blood96h
Blood24h
BloodTwo24h
Blood48h
Blood72h
WholeF
CarcassF
CarcassM
WholeM
GutMidF
GutMidM
SalivF
SalivM
GutHind
GutAnt
GutCaeca
GutPost
Blood3h
BloodNBF
BloodTwoNBF
FatBodyBF
GutMidBF

| | GO.ID | BPCluster: sienna3 Size: 58 | Annotated | Significant | Expected | Rank in ClassicF | Weight01F | ClassicF |
|---|---|---|---|---|---|---|---|---|
| *1* | GO:0055085 | transmembrane transport | 469 | 8 | 2.94 | 1 | 0.0059 | 0.0076 |

| | GO.ID | MFCluster: sienna3 Size: 58 | Annotated | Significant | Expected | Rank in ClassicF | Weight01F | ClassicF |
|---|---|---|---|---|---|---|---|---|
| *1* | GO:0022857 | transmembrane transporter activity | 442 | 6 | 2.70 | 8 | 0.0077 | 0.0502 |
| *8* | GO:1901681 | sulfur compound binding | 23 | 2 | 0.14 | 2 | 0.0750 | 0.0085 |
| *12* | GO:0005215 | transporter activity | 548 | 9 | 3.34 | 1 | 0.0900 | 0.0051 |

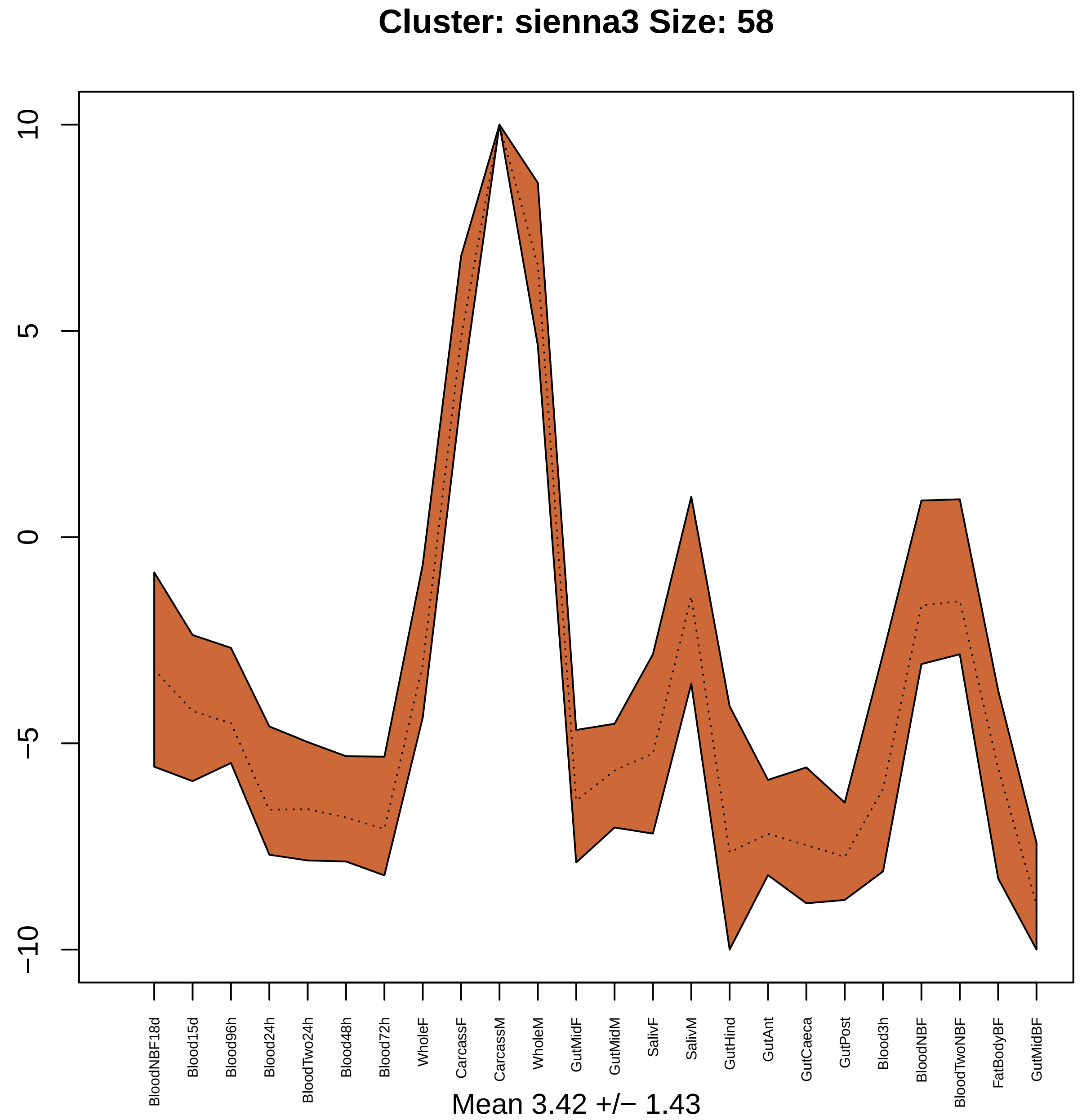

Cluster: sienna3 Size: 58
10
5
0
−5
−10
BloodNBF18d
Blood15d
Blood96h
Blood24h
BloodTwo24h
Blood48h
Blood72h
WholeF
CarcassF
CarcassM
WholeM
GutMidF
GutMidM
SalivF
SalivM
GutHind
GutAnt
GutCaeca
GutPost
Blood3h
BloodNBF
BloodTwoNBF
FatBodyBF
GutMidBF
Mean 3.42 +/− 1.43

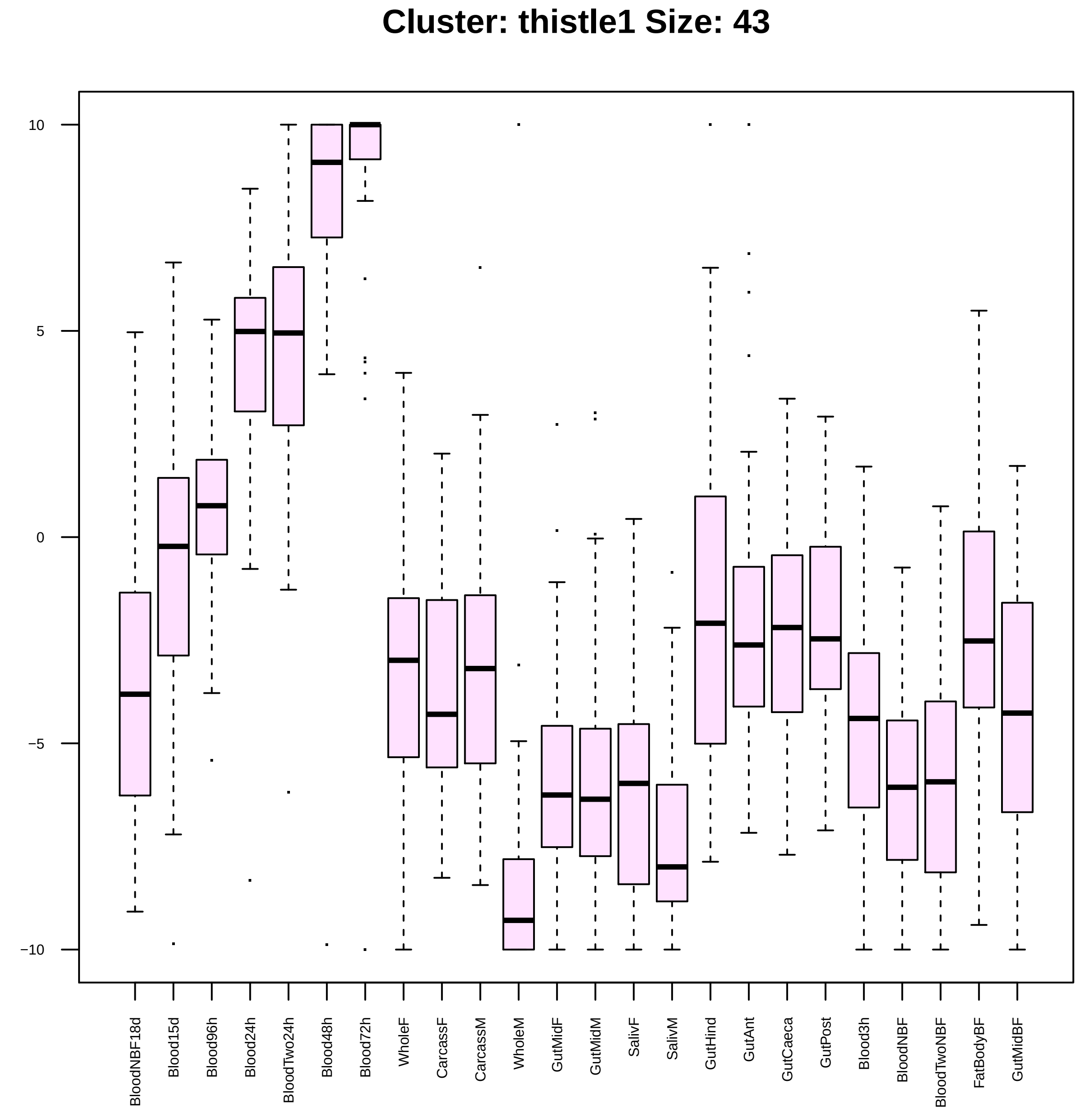
Cluster: thistle1 Size: 43
10
5
0
−5
−10
BloodNBF18d
Blood15d
Blood96h
Blood24h
BloodTwo24h
Blood48h
Blood72h
WholeF
CarcassF
CarcassM
WholeM
GutMidF
GutMidM
SalivF
SalivM
GutHind
GutAnt
GutCaeca
GutPost
Blood3h
BloodNBF
BloodTwoNBF
FatBodyBF
GutMidBF

| | GO.ID | BPCluster: thistle1 Size: 43 | Annotated | Significant | Expected | Rank in ClassicF | Weight01F | ClassicF |
|---|---|---|---|---|---|---|---|---|
| *1* | GO:0000375 | RNA splicing, via transesterification re... | 135 | 2 | 0.47 | 108 | 0.0034 | 0.07802 |
| *2* | GO:0007143 | female meiotic nuclear division | 26 | 2 | 0.09 | 5 | 0.0035 | 0.00351 |
| *3* | GO:0016570 | histone modification | 58 | 3 | 0.20 | 2 | 0.0072 | 0.00097 |
| *4* | GO:0032774 | RNA biosynthetic process | 565 | 4 | 1.95 | 161 | 0.0098 | 0.12460 |

| | GO.ID | MFCluster: thistle1 Size: 43 | Annotated | Significant | Expected | Rank in ClassicF | Weight01F | ClassicF |
|---|---|---|---|---|---|---|---|---|
| *1* | GO:0003682 | chromatin binding | 69 | 3 | 0.27 | 3 | 0.0023 | 0.0023 |
| *2* | GO:0003676 | nucleic acid binding | 1152 | 11 | 4.47 | 4 | 0.0048 | 0.0026 |

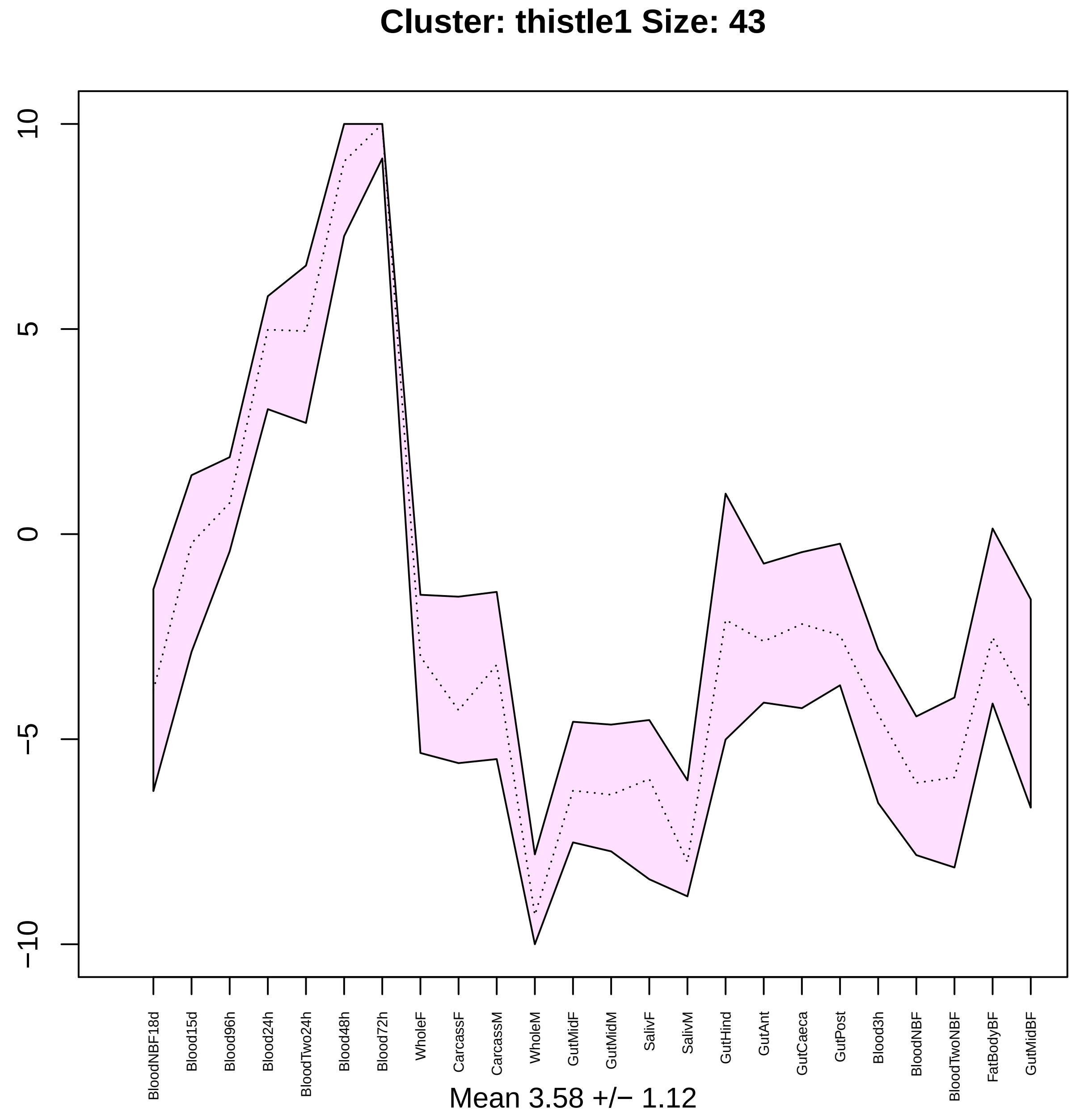

Cluster: thistle1 Size: 43
10
5
0
−5
−10
BloodNBF18d
Blood15d
Blood96h
Blood24h
BloodTwo24h
Blood48h
Blood72h
WholeF
CarcassF
CarcassM
WholeM
GutMidF
GutMidM
SalivF
SalivM
GutHind
GutAnt
GutCaeca
GutPost
Blood3h
BloodNBF
BloodTwoNBF
FatBodyBF
GutMidBF
Mean 3.58 +/− 1.12

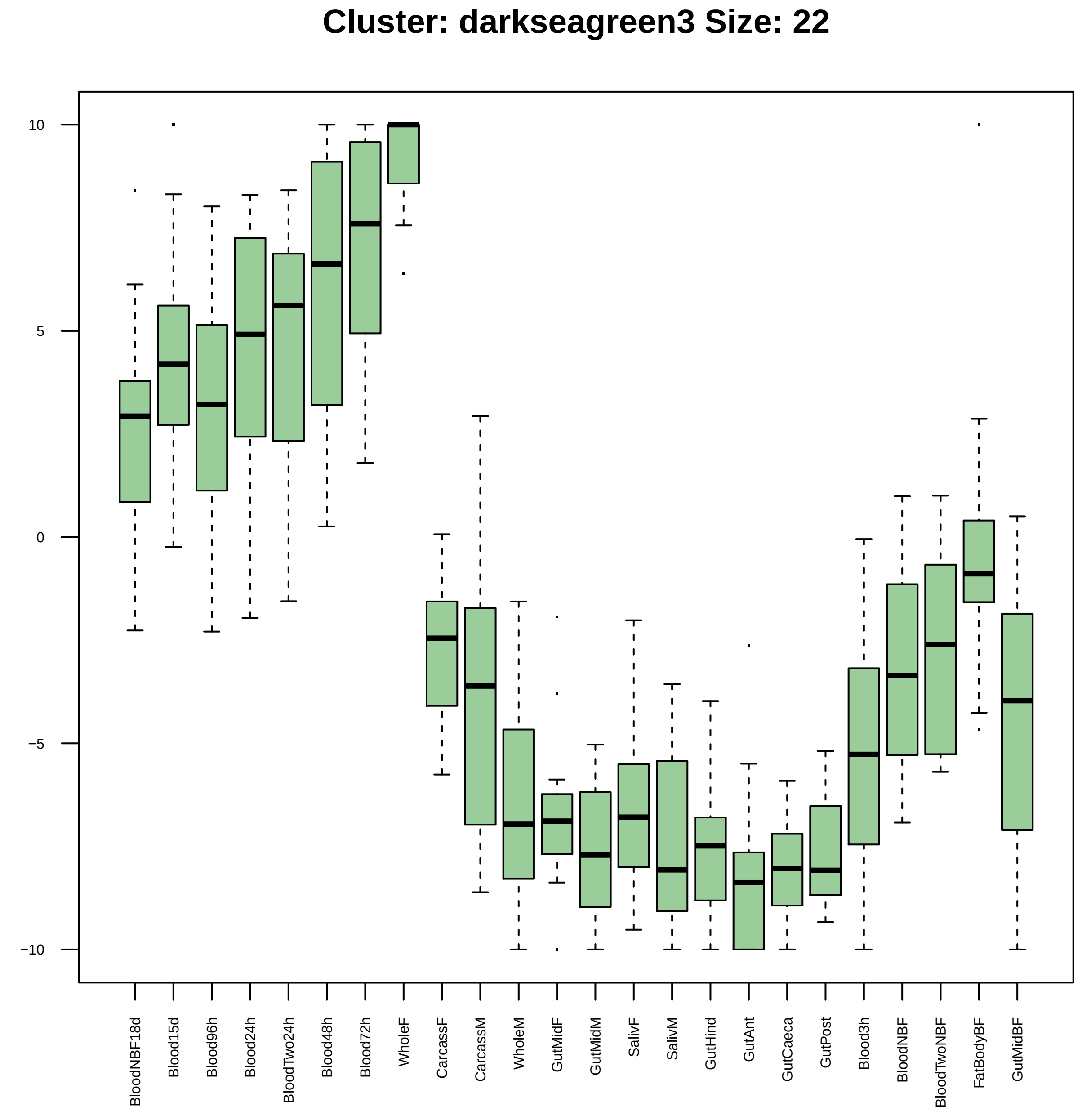
Cluster: darkseagreen3 Size: 22
10
5
0
−5
−10
BloodNBF18d
Blood15d
Blood96h
Blood24h
BloodTwo24h
Blood48h
Blood72h
WholeF
CarcassF
CarcassM
WholeM
GutMidF
GutMidM
SalivF
SalivM
GutHind
GutAnt
GutCaeca
GutPost
Blood3h
BloodNBF
BloodTwoNBF
FatBodyBF
GutMidBF

| | GO.ID | BPCluster: darkseagreen3 Size: 22 | Annotated | Significant | Expected | Rank in ClassicF | Weight01F | ClassicF |
|---|---|---|---|---|---|---|---|---|
| 1 | GO:0061983 | meiosis II cell cycle process | 10 | 2 | 0.02 | 1 | 0.00017 | 0.00017 |
| 2 | GO:0044837 | actomyosin contractile ring organization | 14 | 2 | 0.03 | 2 | 0.00034 | 0.00034 |
| 3 | GO:0032506 | cytokinetic process | 15 | 2 | 0.03 | 3 | 0.00040 | 0.00040 |
| 4 | GO:0072593 | reactive oxygen species metabolic proces... | 21 | 2 | 0.04 | 10 | 0.00079 | 0.00079 |
| 5 | GO:0051225 | spindle assembly | 27 | 2 | 0.06 | 13 | 0.00131 | 0.00131 |
| 6 | GO:0007283 | spermatogenesis | 76 | 3 | 0.15 | 5 | 0.00189 | 0.00043 |
| 7 | GO:0043066 | negative regulation of apoptotic process | 34 | 2 | 0.07 | 15 | 0.00207 | 0.00207 |
| 8 | GO:0006979 | response to oxidative stress | 51 | 2 | 0.10 | 23 | 0.00462 | 0.00462 |
| 9 | GO:0033206 | meiotic cytokinesis | 20 | 2 | 0.04 | 8 | 0.00753 | 0.00071 |

| | GO.ID | MFCluster: darkseagreen3 Size: 22 | Annotated | Significant | Expected | Rank in ClassicF | Weight01F | ClassicF |
|---|---|---|---|---|---|---|---|---|
| *1* | GO:0004601 | peroxidase activity | 26 | 2 | 0.05 | 1 | 0.0013 | 0.0013 |
| *2* | GO:0004674 | protein serine/threonine kinase activity | 150 | 3 | 0.31 | 5 | 0.0033 | 0.0033 |
| *21* | GO:0016684 | oxidoreductase activity, acting on perox... | 26 | 2 | 0.05 | 2 | 1.0000 | 0.0013 |

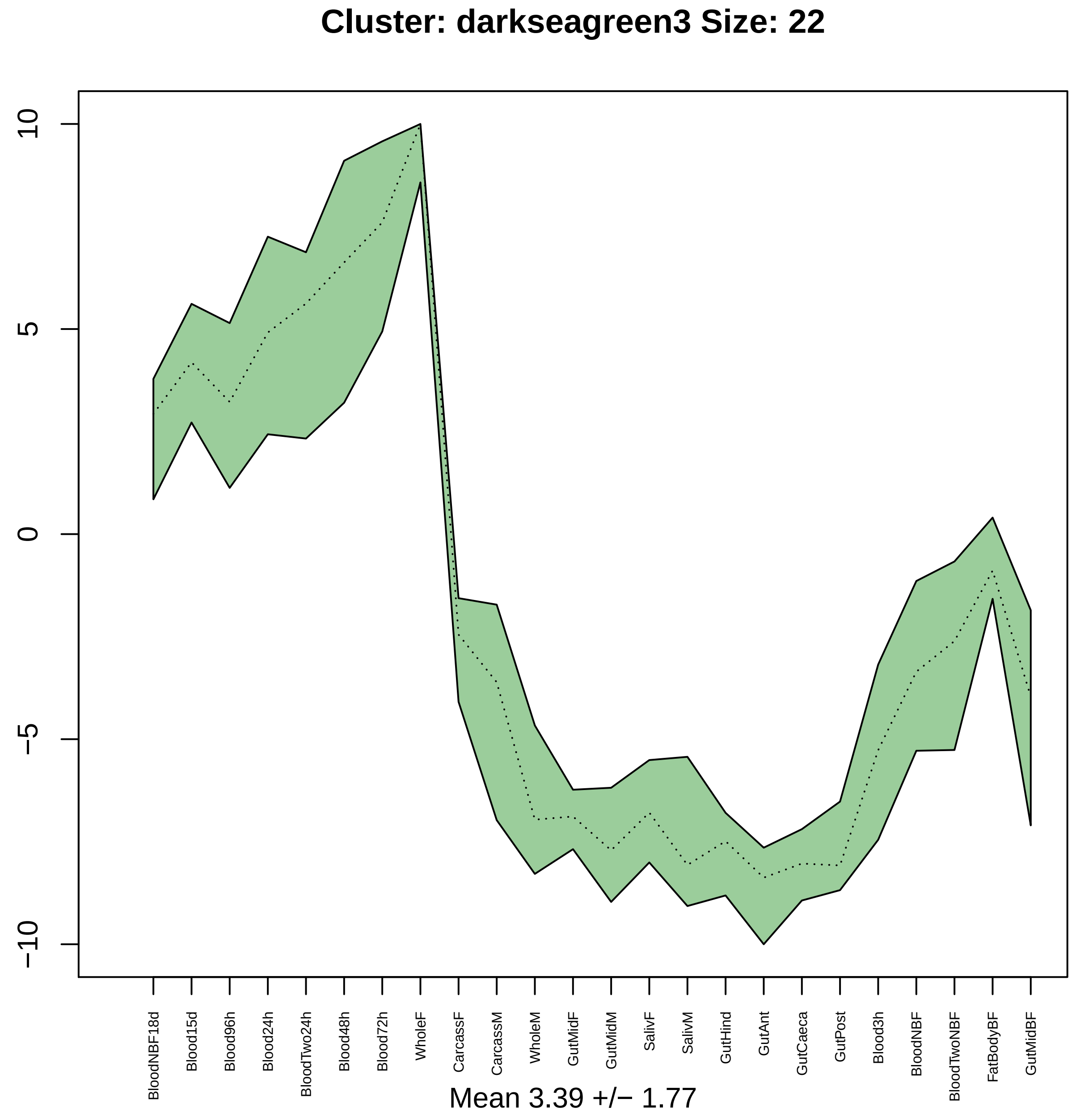

Cluster: darkseagreen3 Size: 22
10
5
0
−5
−10
BloodNBF18d
Blood15d
Blood96h
Blood24h
BloodTwo24h
Blood48h
Blood72h
WholeF
CarcassF
CarcassM
WholeM
GutMidF
GutMidM
SalivF
SalivM
GutHind
GutAnt
GutCaeca
GutPost
Blood3h
BloodNBF
BloodTwoNBF
FatBodyBF
GutMidBF
Mean 3.39 +/− 1.77

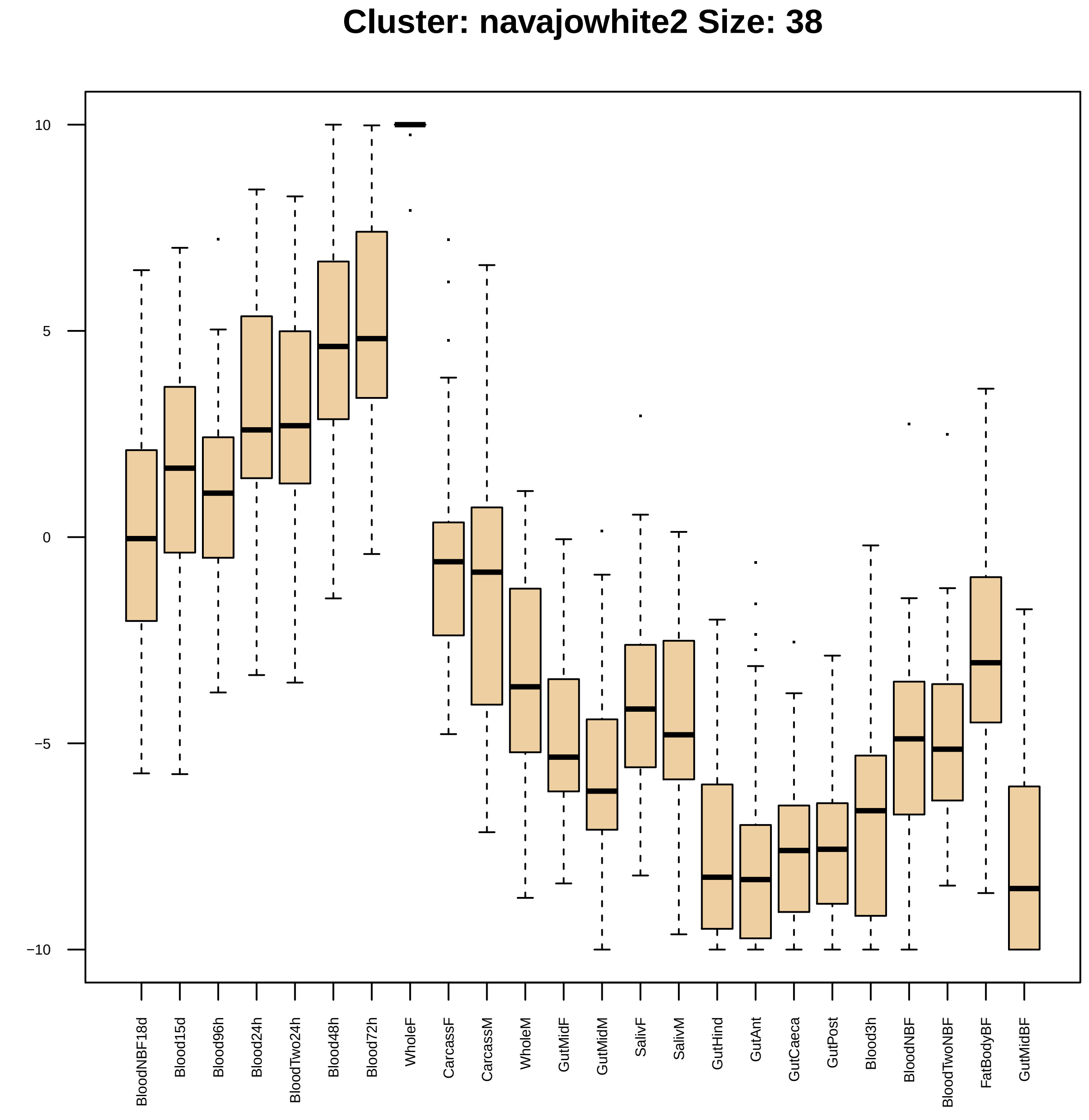

Cluster: navajowhite2 Size: 38
10
5
0
−5
−10
BloodNBF18d
Blood15d
Blood96h
Blood24h
BloodTwo24h
Blood48h
Blood72h
WholeF
CarcassF
CarcassM
WholeM
GutMidF
GutMidM
SalivF
SalivM
GutHind
GutAnt
GutCaeca
GutPost
Blood3h
BloodNBF
BloodTwoNBF
FatBodyBF
GutMidBF

| | GO.ID | BPCluster: navajowhite2 Size: 38 | Annotated | Significant | Expected | Rank in ClassicF | Weight01F | ClassicF |
|---|---|---|---|---|---|---|---|---|
| 1 | GO:0030727 | germarium−derived female germ−line cyst ... | 12 | 2 | 0.04 | 7 | 0.00061 | 0.00061 |
| 2 | GO:0000079 | regulation of cyclin−dependent protein s... | 13 | 2 | 0.04 | 10 | 0.00071 | 0.00071 |
| 3 | GO:0030100 | regulation of endocytosis | 15 | 2 | 0.05 | 13 | 0.00096 | 0.00096 |
| 4 | GO:0022008 | neurogenesis | 706 | 6 | 2.21 | 79 | 0.00193 | 0.01790 |
| 5 | GO:0051171 | regulation of nitrogen compound metaboli... | 775 | 9 | 2.43 | 3 | 0.00641 | 0.00027 |
| 6 | GO:0007314 | oocyte anterior/posterior axis specifica... | 44 | 2 | 0.14 | 43 | 0.00817 | 0.00817 |
| 7 | GO:0051247 | positive regulation of protein metabolic... | 63 | 2 | 0.20 | 75 | 0.00900 | 0.01628 |
| 9 | GO:0007127 | meiosis I | 19 | 2 | 0.06 | 17 | 0.01190 | 0.00155 |
| 11 | GO:0080090 | regulation of primary metabolic process | 774 | 9 | 2.43 | 2 | 0.02119 | 0.00026 |
| 12 | GO:0051028 | mRNA transport | 21 | 2 | 0.07 | 20 | 0.02365 | 0.00189 |
| 15 | GO:0031323 | regulation of cellular metabolic process | 792 | 8 | 2.48 | 19 | 0.03206 | 0.00174 |
| 27 | GO:0021700 | developmental maturation | 91 | 3 | 0.29 | 25 | 0.04205 | 0.00269 |

| | GO.ID | MFCluster: navajowhite2 Size: 38 | Annotated | Significant | Expected | Rank in ClassicF | Weight01F | ClassicF |
|---|---|---|---|---|---|---|---|---|
| *1* | GO:0004693 | cyclin−dependent protein serine/threonin... | 10 | 2 | 0.03 | 1 | 0.00047 | 0.00047 |
| *2* | GO:0019901 | protein kinase binding | 24 | 2 | 0.08 | 7 | 0.00280 | 0.00280 |
| *3* | GO:0016881 | acid−amino acid ligase activity | 38 | 2 | 0.13 | 13 | 0.00694 | 0.00694 |
| *4* | GO:0004536 | deoxyribonuclease activity | 19 | 2 | 0.06 | 4 | 0.02217 | 0.00175 |
| *6* | GO:0008094 | DNA−dependent ATPase activity | 33 | 2 | 0.11 | 11 | 0.03776 | 0.00527 |
| *17* | GO:0008026 | ATP−dependent helicase activity | 76 | 3 | 0.25 | 5 | 0.07987 | 0.00194 |

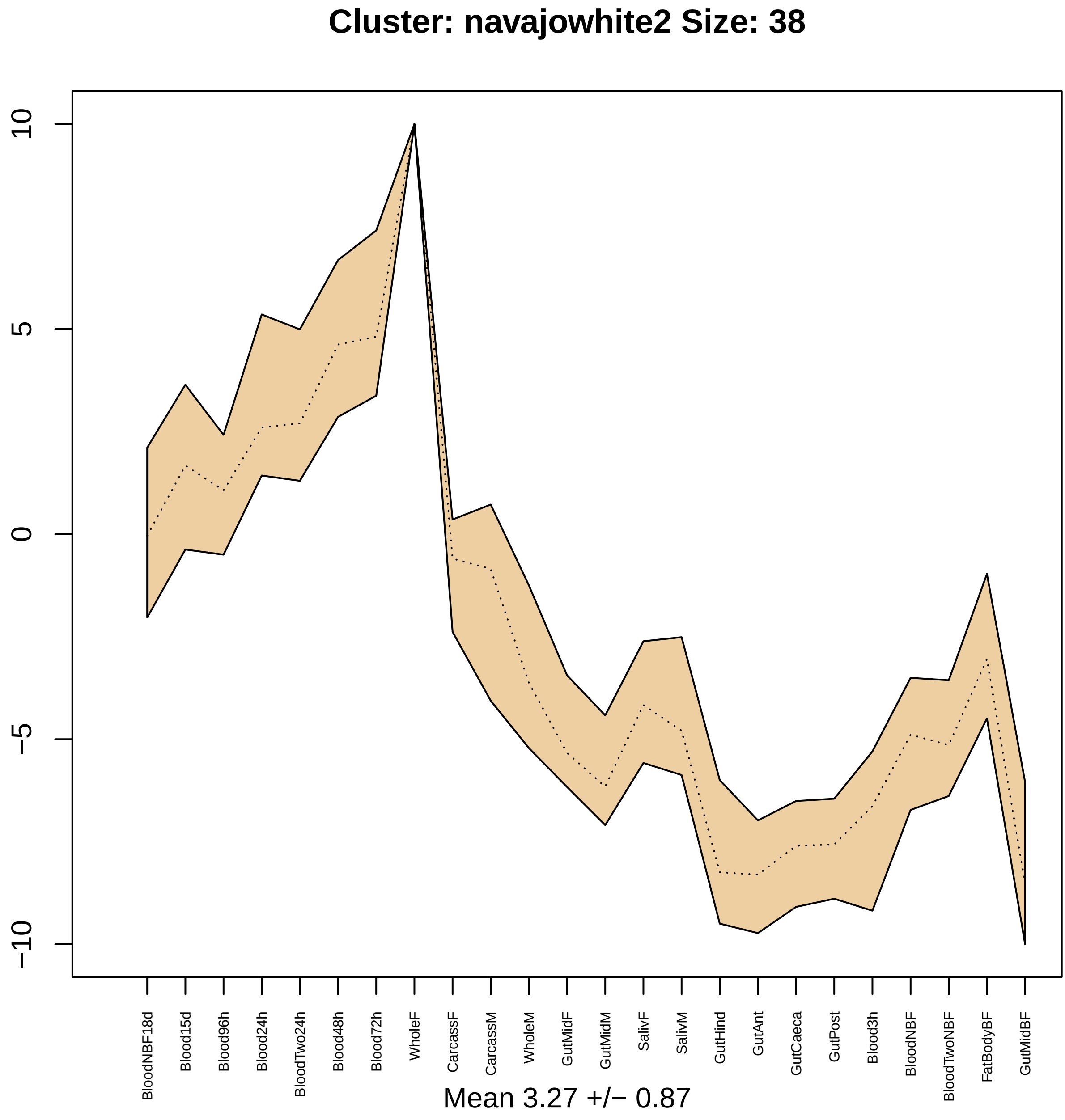

Cluster: navajowhite2 Size: 38
10
5
0
−5
−10
BloodNBF18d
Blood15d
Blood96h
Blood24h
BloodTwo24h
Blood48h
Blood72h
WholeF
CarcassF
CarcassM
WholeM
GutMidF
GutMidM
SalivF
SalivM
GutHind
GutAnt
GutCaeca
GutPost
Blood3h
BloodNBF
BloodTwoNBF
FatBodyBF
GutMidBF
Mean 3.27 +/− 0.87

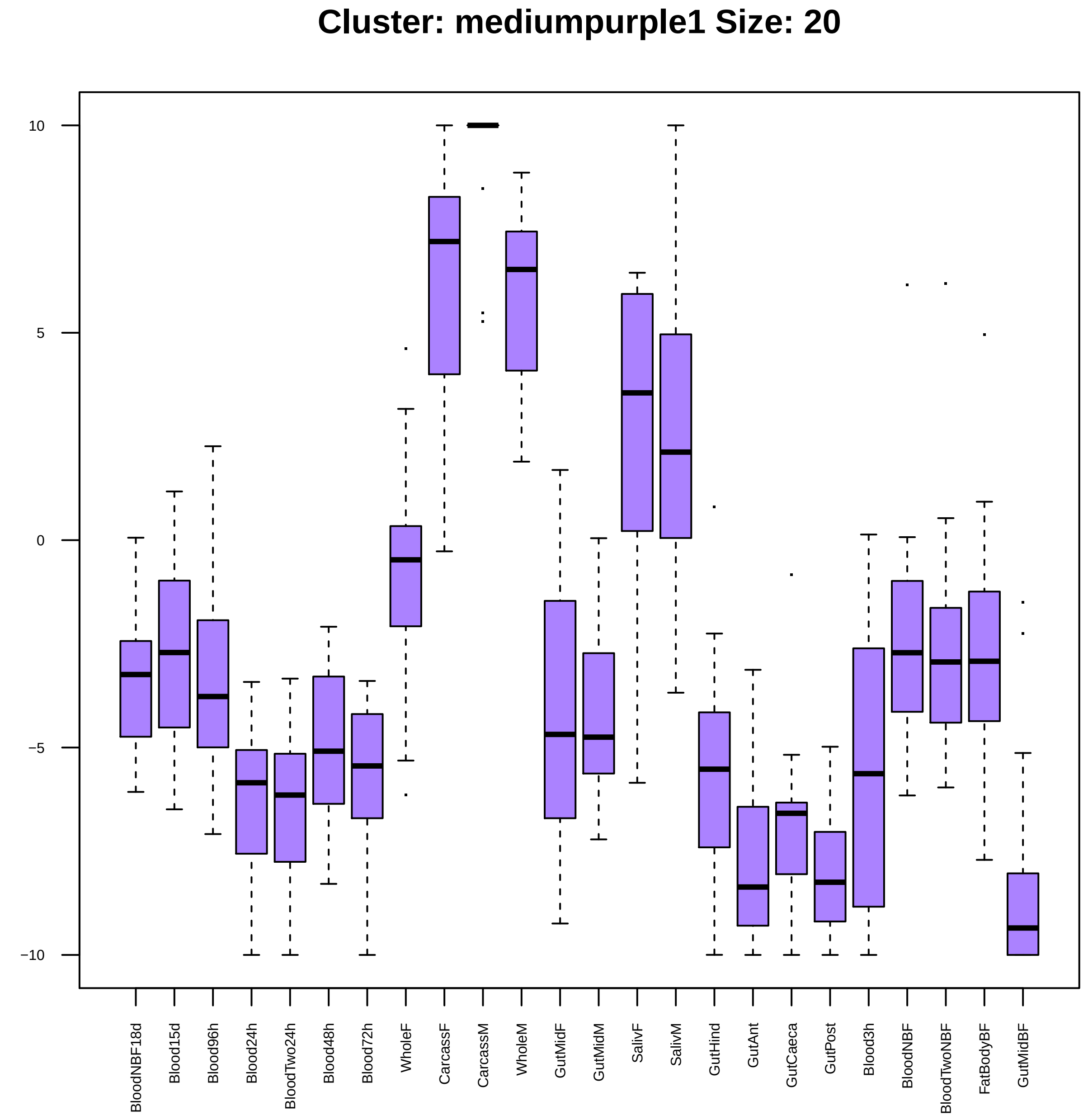

Cluster: mediumpurple1 Size: 20
10
5
0
−5
−10
BloodNBF18d
Blood15d
Blood96h
Blood24h
BloodTwo24h
Blood48h
Blood72h
WholeF
CarcassF
CarcassM
WholeM
GutMidF
GutMidM
SalivF
SalivM
GutHind
GutAnt
GutCaeca
GutPost
Blood3h
BloodNBF
BloodTwoNBF
FatBodyBF
GutMidBF

| | GO.ID | BPCluster: mediumpurple1 Size: 20 | Annotated | Significant | Expected | Rank in ClassicF | Weight01F | ClassicF |
|---|---|---|---|---|---|---|---|---|
| *1* | GO:0044092 | negative regulation of molecular functio... | 65 | 2 | 0.11 | 1 | 0.0095 | 0.0053 |

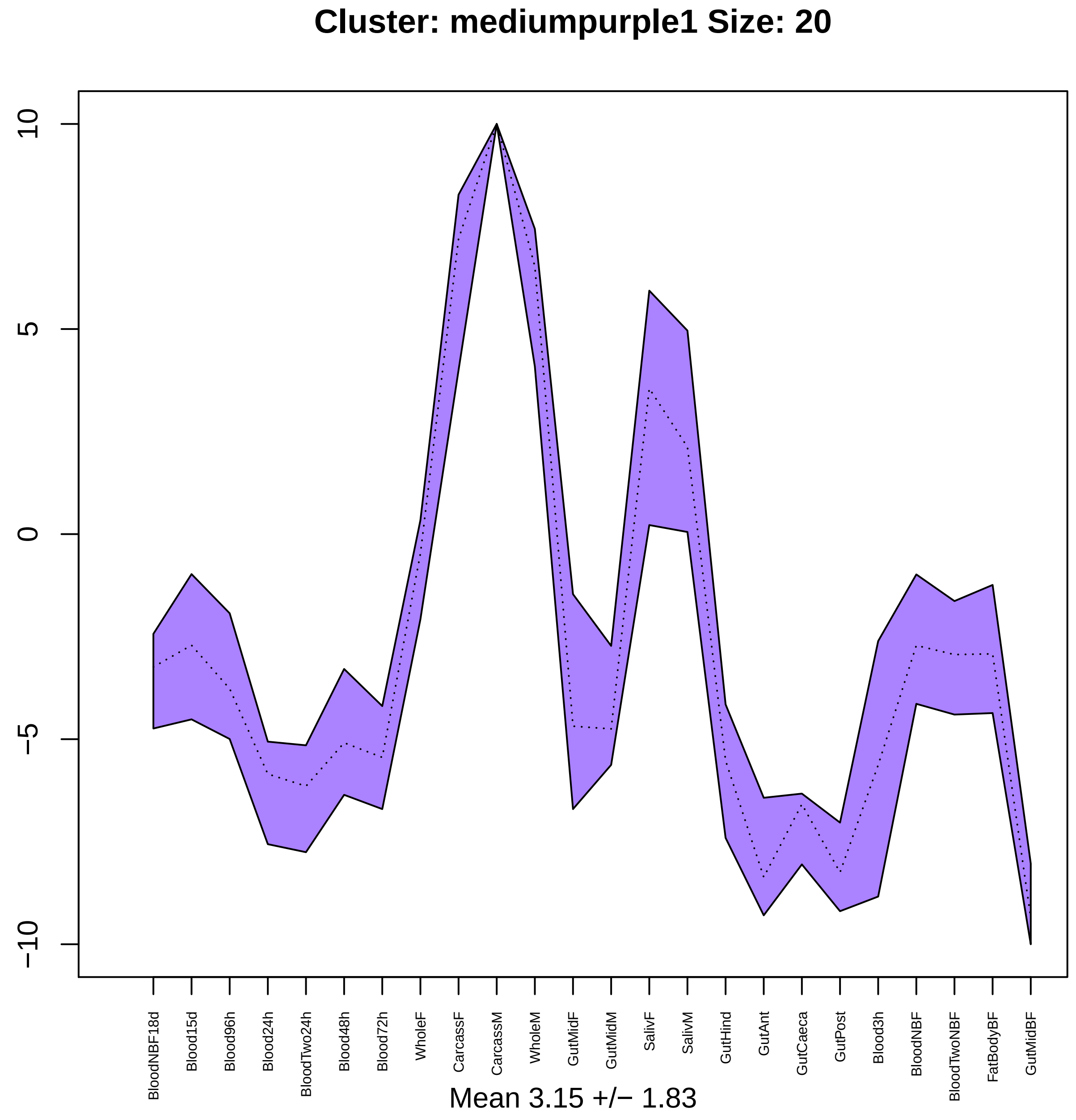

Cluster: mediumpurple1 Size: 20
10
5
0
−5
−10
BloodNBF18d
Blood15d
Blood96h
Blood24h
BloodTwo24h
Blood48h
Blood72h
WholeF
CarcassF
CarcassM
WholeM
GutMidF
GutMidM
SalivF
SalivM
GutHind
GutAnt
GutCaeca
GutPost
Blood3h
BloodNBF
BloodTwoNBF
FatBodyBF
GutMidBF
Mean 3.15 +/− 1.83

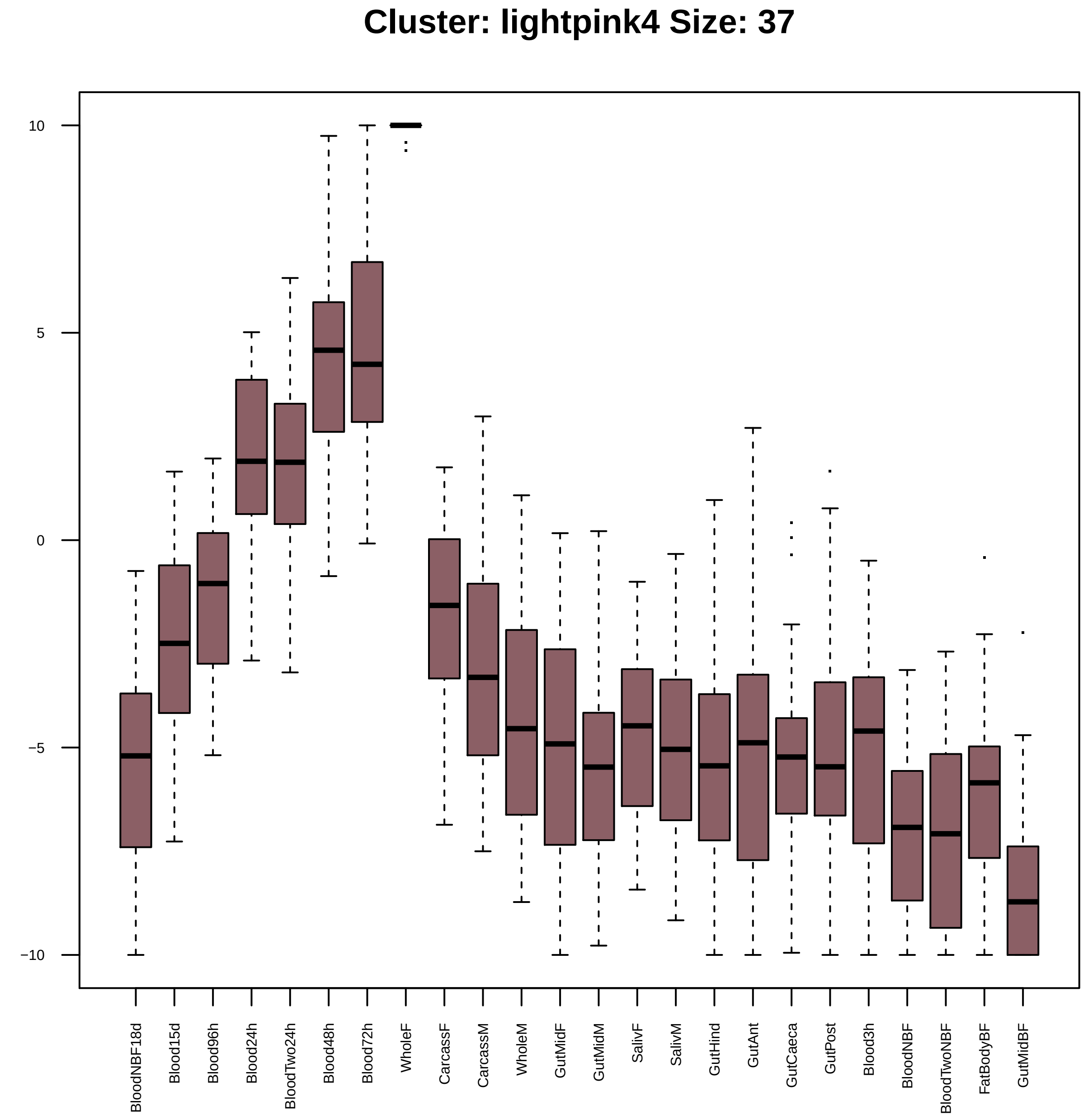

Cluster: lightpink4 Size: 37
10
5
0
−5
−10
BloodNBF18d
Blood15d
Blood96h
Blood24h
BloodTwo24h
Blood48h
Blood72h
WholeF
CarcassF
CarcassM
WholeM
GutMidF
GutMidM
SalivF
SalivM
GutHind
GutAnt
GutCaeca
GutPost
Blood3h
BloodNBF
BloodTwoNBF
FatBodyBF
GutMidBF

| | GO.ID | BPCluster: lightpink4 Size: 37 | Annotated | Significant | Expected | Rank in ClassicF | Weight01F | ClassicF |
|---|---|---|---|---|---|---|---|---|
| 2 | GO:0044786 | cell cycle DNA replication | 18 | 3 | 0.06 | 8 | 0.00013 | 2.1e−05 |
| 3 | GO:0065004 | protein−DNA complex assembly | 29 | 3 | 0.09 | 11 | 0.00055 | 9.2e−05 |
| 4 | GO:0006270 | DNA replication initiation | 12 | 2 | 0.04 | 15 | 0.00061 | 0.00061 |
| 5 | GO:0000724 | double−strand break repair via homologou... | 12 | 2 | 0.04 | 16 | 0.00061 | 0.00061 |
| 6 | GO:0007131 | reciprocal meiotic recombination | 15 | 2 | 0.05 | 18 | 0.00096 | 0.00096 |
| 7 | GO:0032508 | DNA duplex unwinding | 18 | 2 | 0.06 | 24 | 0.00139 | 0.00139 |
| 8 | GO:0046331 | lateral inhibition | 92 | 3 | 0.29 | 35 | 0.00277 | 0.00277 |

|  | GO.ID | MFCluster: lightpink4 Size: 37 | Annotated | Significant | Expected | Rank in ClassicF | Weight01F | ClassicF |
|---|---|---|---|---|---|---|---|---|
| 1 | GO:0005524 | ATP binding | 592 | 8 | 1.64 | 2 | 0.00010 | 0.00010 |
| 2 | GO:0003677 | DNA binding | 523 | 8 | 1.45 | 1 | 0.00051 | 4.2e−05 |
| 3 | GO:0003697 | single−stranded DNA binding | 15 | 2 | 0.04 | 11 | 0.00075 | 0.00075 |
| 4 | GO:0016887 | ATPase activity | 233 | 3 | 0.65 | 30 | 0.00510 | 0.02531 |
| 5 | GO:0044877 | protein−containing complex binding | 50 | 2 | 0.14 | 22 | 0.00827 | 0.00827 |
| 11 | GO:0003678 | DNA helicase activity | 32 | 2 | 0.09 | 18 | 0.05407 | 0.00345 |
| 19 | GO:0017111 | nucleoside−triphosphatase activity | 430 | 6 | 1.19 | 12 | 0.31355 | 0.00082 |
| 25 | GO:1901265 | nucleoside phosphate binding | 998 | 8 | 2.77 | 19 | 1.00000 | 0.00353 |
| 26 | GO:0035639 | purine ribonucleoside triphosphate bindi... | 725 | 8 | 2.01 | 7 | 1.00000 | 0.00042 |
| 27 | GO:0032553 | ribonucleotide binding | 740 | 8 | 2.05 | 9 | 1.00000 | 0.00049 |
| 29 | GO:0032555 | purine ribonucleotide binding | 732 | 8 | 2.03 | 8 | 1.00000 | 0.00045 |
| 30 | GO:0036094 | small molecule binding | 1068 | 8 | 2.96 | 21 | 1.00000 | 0.00541 |

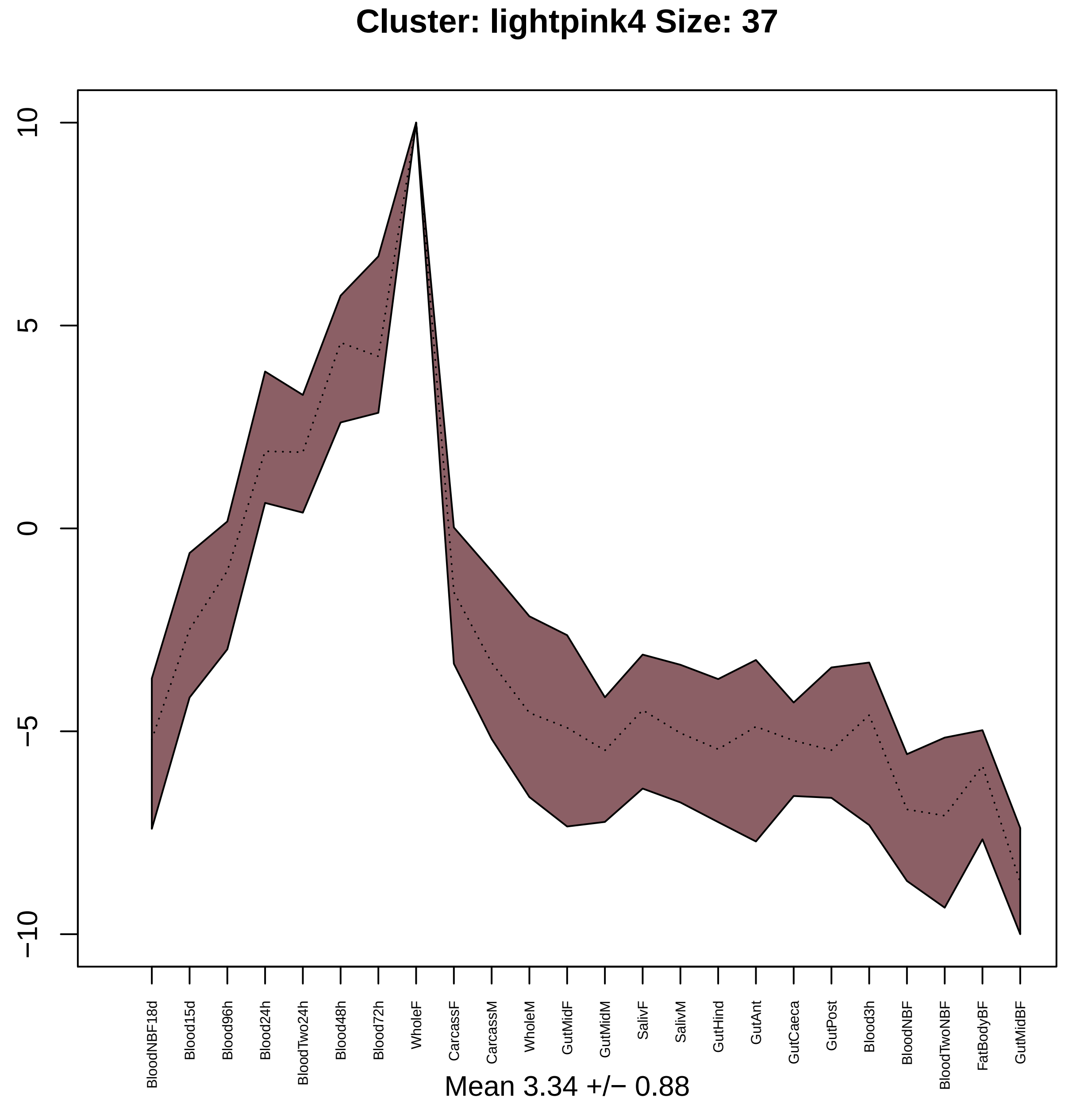

Cluster: lightpink4 Size: 37
10
5
0
−5
−10
BloodNBF18d
Blood15d
Blood96h
Blood24h
BloodTwo24h
Blood48h
Blood72h
WholeF
CarcassF
CarcassM
WholeM
GutMidF
GutMidM
SalivF
SalivM
GutHind
GutAnt
GutCaeca
GutPost
Blood3h
BloodNBF
BloodTwoNBF
FatBodyBF
GutMidBF
Mean 3.34 +/− 0.88

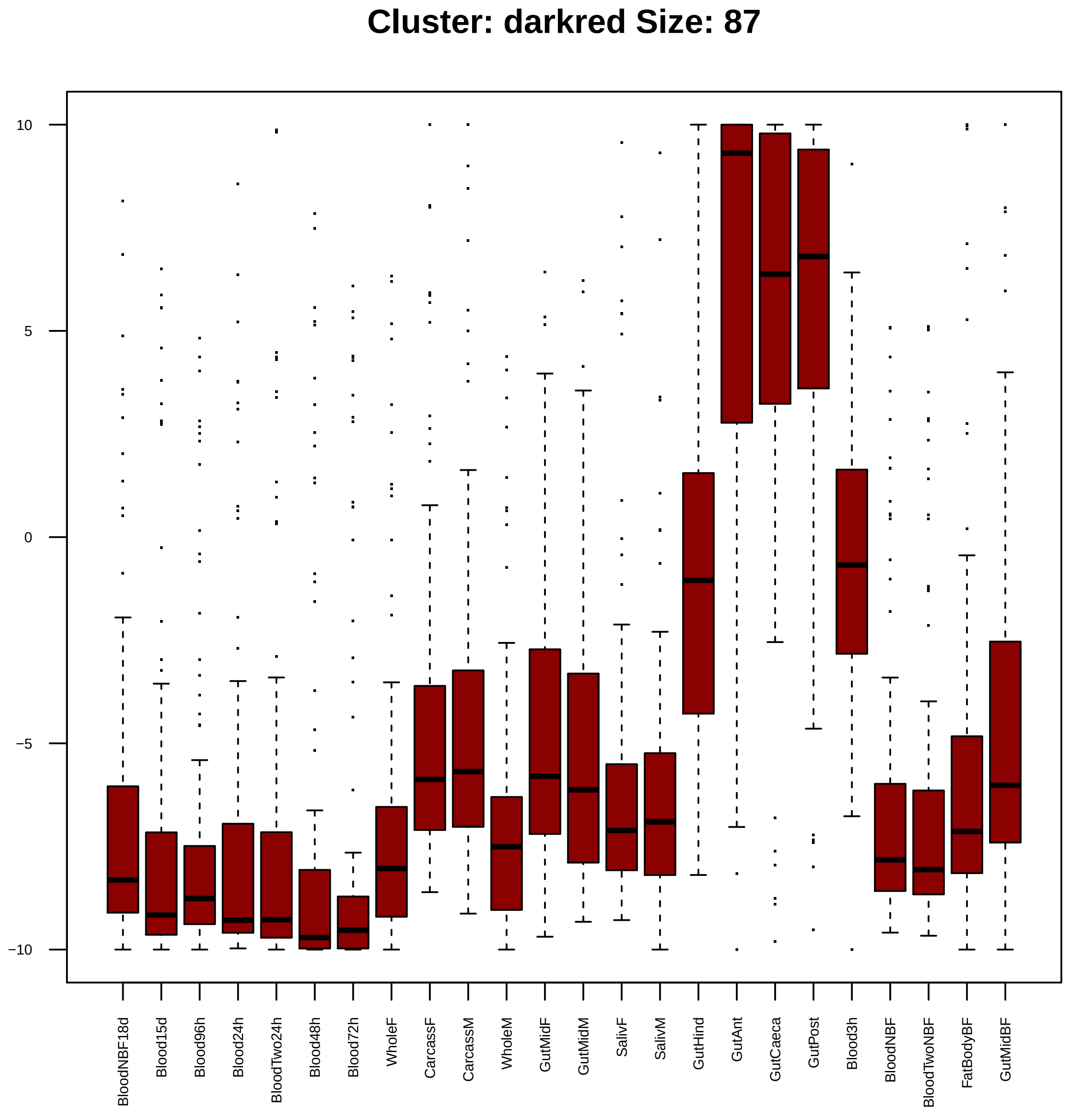
Cluster: darkred Size: 87
10
5
0
−5
−10
BloodNBF18d
Blood15d
Blood96h
Blood24h
BloodTwo24h
Blood48h
Blood72h
WholeF
CarcassF
CarcassM
WholeM
GutMidF
GutMidM
SalivF
SalivM
GutHind
GutAnt
GutCaeca
GutPost
Blood3h
BloodNBF
BloodTwoNBF
FatBodyBF
GutMidBF

| | GO.ID | BPCluster: darkred Size: 87 | Annotated | Significant | Expected | Rank in ClassicF | Weight01F | ClassicF |
|---|---|---|---|---|---|---|---|---|
| *3* | GO:0008152 | metabolic process | 4285 | 59 | 46.36 | 6 | 0.00056 | 0.00045 |

| | GO.ID | MFCluster: darkred Size: 87 | Annotated | Significant | Expected | Rank in ClassicF | Weight01F | ClassicF |
|---|---|---|---|---|---|---|---|---|
| *2* | GO:0008061 | chitin binding | 76 | 6 | 0.76 | 9 | 0.0001 | 0.0001 |
| *3* | GO:0004497 | monooxygenase activity | 110 | 5 | 1.10 | 11 | 0.0047 | 0.0047 |
| *4* | GO:0032934 | sterol binding | 12 | 2 | 0.12 | 12 | 0.0061 | 0.0061 |

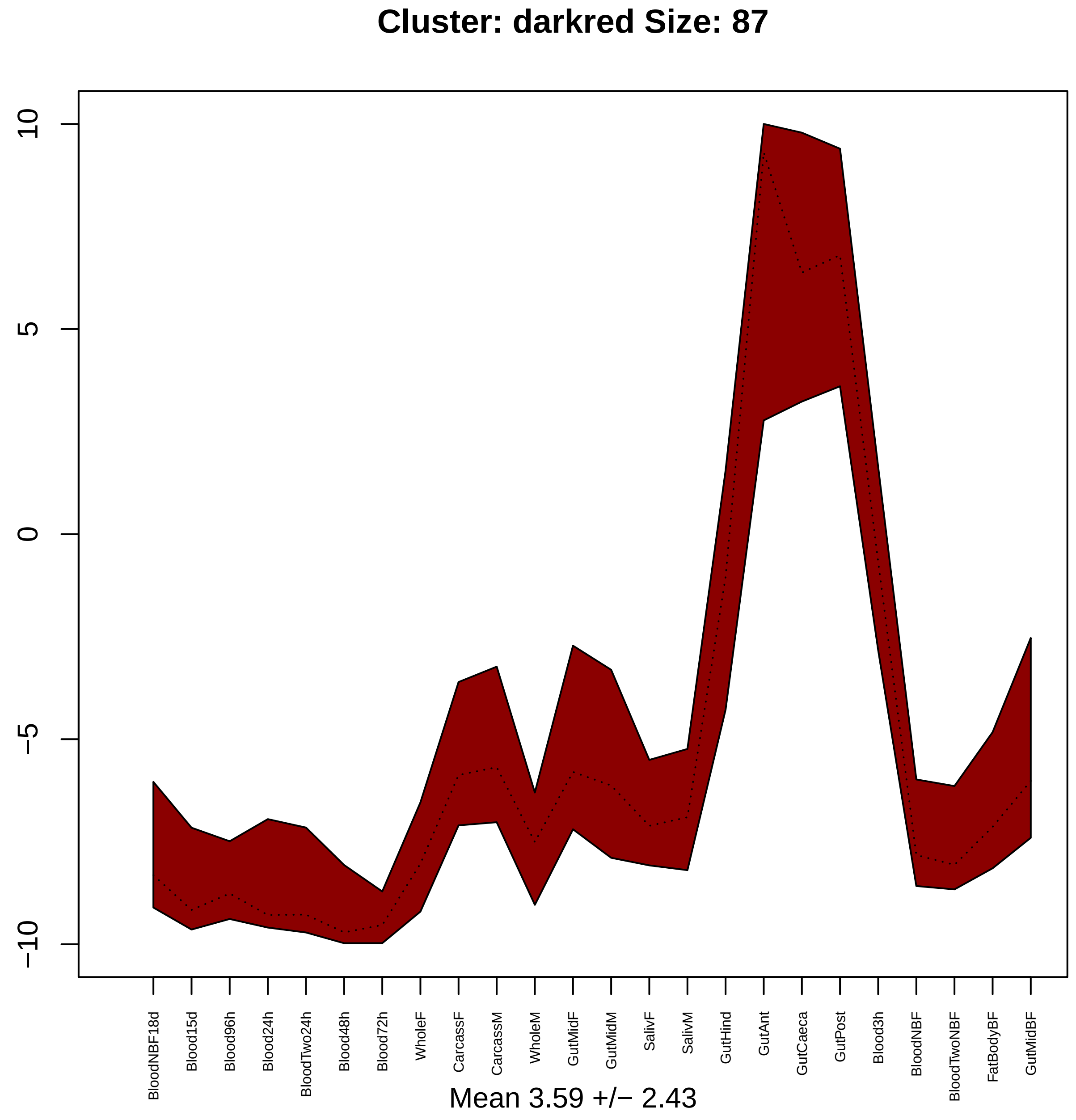

Cluster: darkred Size: 87
10
5
0
−5
−10
BloodNBF18d
Blood15d
Blood96h
Blood24h
BloodTwo24h
Blood48h
Blood72h
WholeF
CarcassF
CarcassM
WholeM
GutMidF
GutMidM
SalivF
SalivM
GutHind
GutAnt
GutCaeca
GutPost
Blood3h
BloodNBF
BloodTwoNBF
FatBodyBF
GutMidBF
Mean 3.59 +/− 2.43

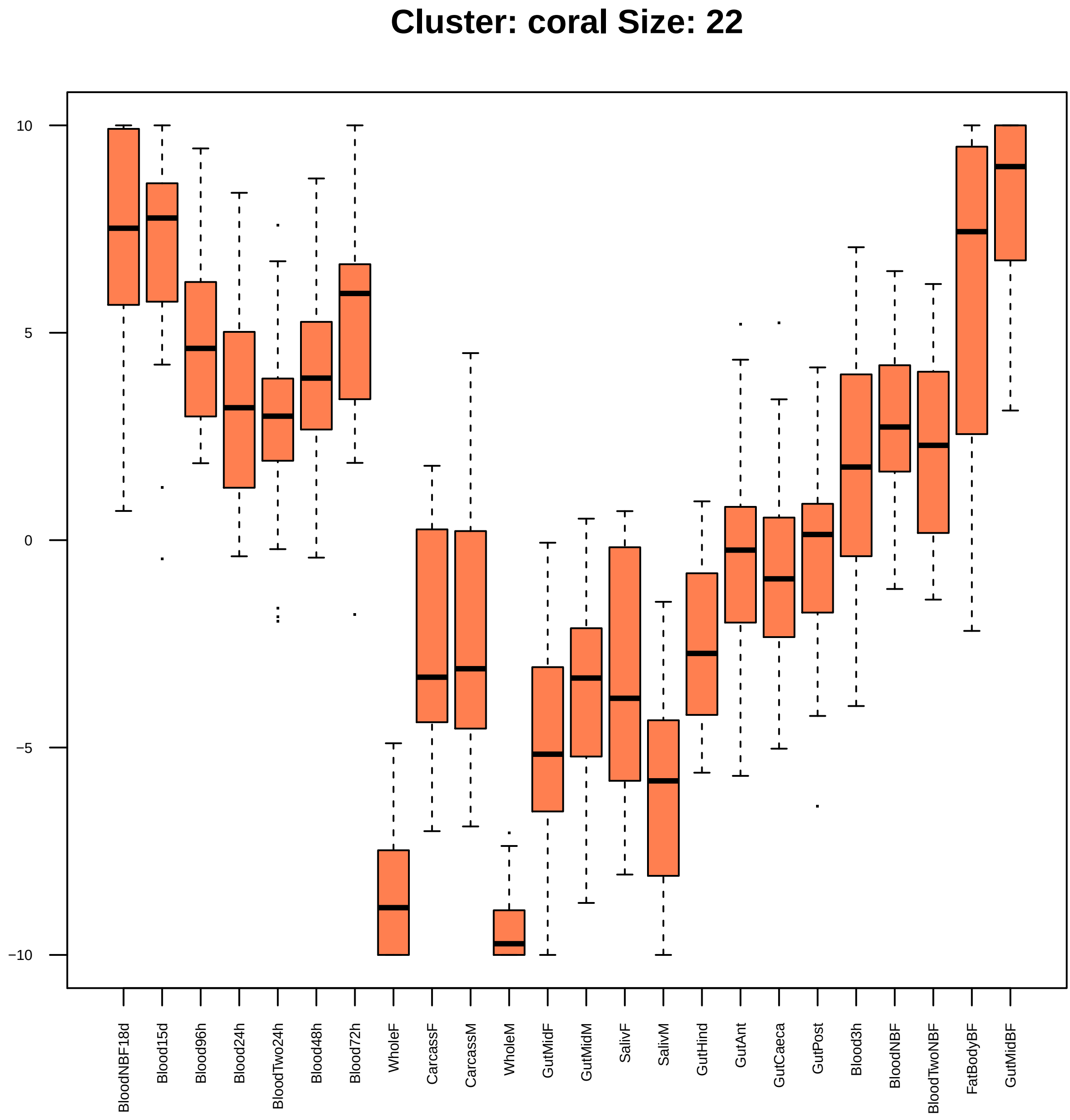

Cluster: coral Size: 22
10
5
0
−5
−10
BloodNBF18d
Blood15d
Blood96h
Blood24h
BloodTwo24h
Blood48h
Blood72h
WholeF
CarcassF
CarcassM
WholeM
GutMidF
GutMidM
SalivF
SalivM
GutHind
GutAnt
GutCaeca
GutPost
Blood3h
BloodNBF
BloodTwoNBF
FatBodyBF
GutMidBF

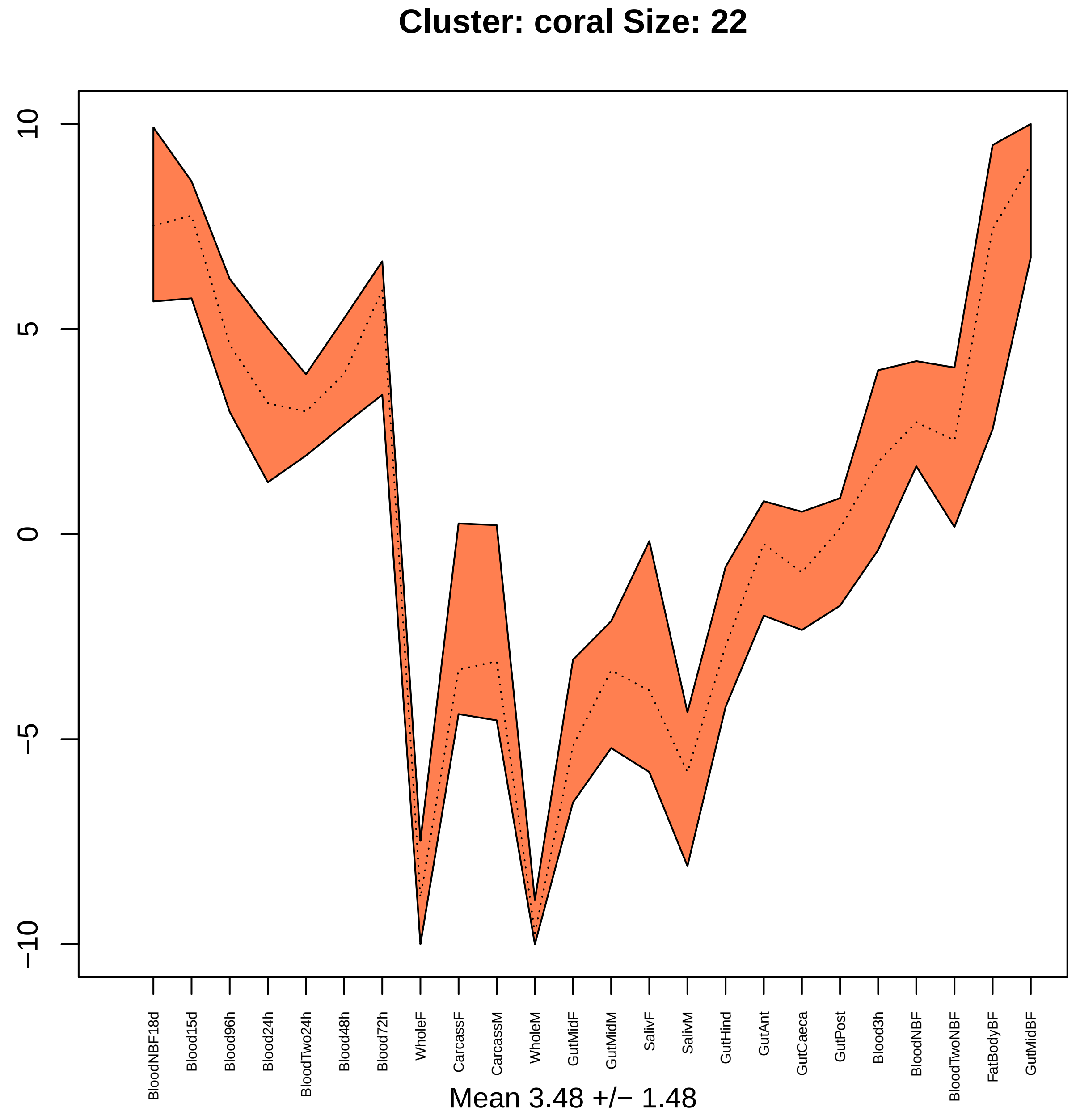

Cluster: coral Size: 22
10
5
0
−5
−10
BloodNBF18d
Blood15d
Blood96h
Blood24h
BloodTwo24h
Blood48h
Blood72h
WholeF
CarcassF
CarcassM
WholeM
GutMidF
GutMidM
SalivF
SalivM
GutHind
GutAnt
GutCaeca
GutPost
Blood3h
BloodNBF
BloodTwoNBF
FatBodyBF
GutMidBF
Mean 3.48 +/− 1.48

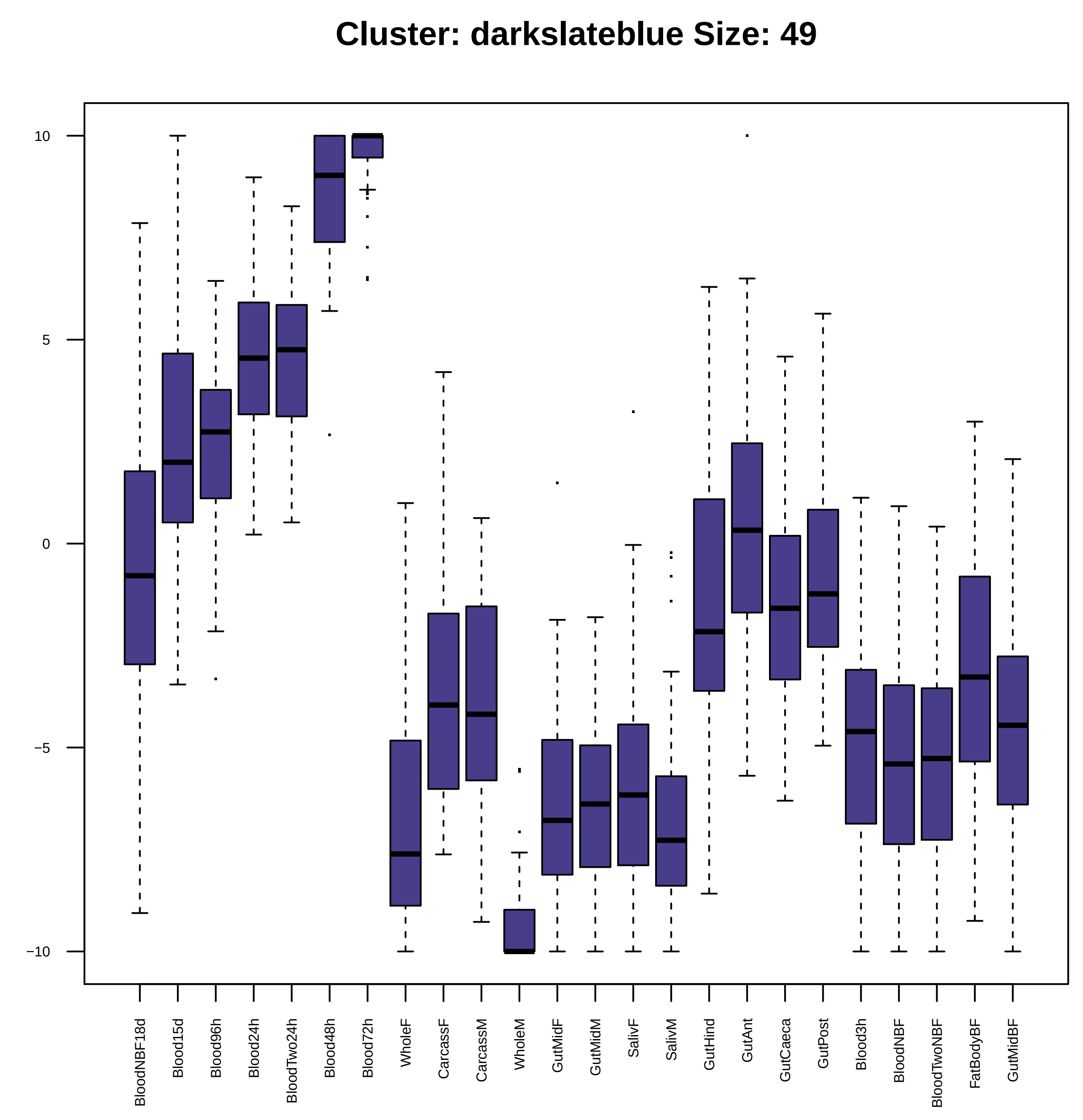

Cluster: darkslateblue Size: 49
10
5
0
−5
−10
BloodNBF18d
Blood15d
Blood96h
Blood24h
BloodTwo24h
Blood48h
Blood72h
WholeF
CarcassF
CarcassM
WholeM
GutMidF
GutMidM
SalivF
SalivM
GutHind
GutAnt
GutCaeca
GutPost
Blood3h
BloodNBF
BloodTwoNBF
FatBodyBF
GutMidBF

| | GO.ID | BPCluster: darkslateblue Size: 49 | Annotated | Significant | Expected | Rank in ClassicF | Weight01F | ClassicF |
|---|---|---|---|---|---|---|---|---|
| 1 | GO:0008589 | regulation of smoothened signaling pathw... | 17 | 2 | 0.07 | 2 | 0.0019 | 0.0019 |
| 2 | GO:0006338 | chromatin remodeling | 37 | 2 | 0.15 | 9 | 0.0090 | 0.0090 |
| 3 | GO:0016571 | histone methylation | 26 | 2 | 0.10 | 4 | 0.0150 | 0.0045 |

| | GO.ID | MFCluster: darkslateblue Size: 49 | Annotated | Significant | Expected | Rank in ClassicF | Weight01F | ClassicF |
|---|---|---|---|---|---|---|---|---|
| *1* | GO:0003729 | mRNA binding | 99 | 4 | 0.43 | 1 | 0.00079 | 0.00079 |
| *2* | GO:0008017 | microtubule binding | 55 | 3 | 0.24 | 2 | 0.00162 | 0.00162 |
| *4* | GO:0042054 | histone methyltransferase activity | 17 | 2 | 0.07 | 6 | 0.01246 | 0.00234 |
| *6* | GO:0003677 | DNA binding | 523 | 7 | 2.25 | 10 | 0.01612 | 0.00577 |
| *9* | GO:0008170 | N−methyltransferase activity | 25 | 2 | 0.11 | 7 | 0.04495 | 0.00505 |
| *20* | GO:0008168 | methyltransferase activity | 88 | 3 | 0.38 | 11 | 0.14328 | 0.00617 |
| *30* | GO:0003676 | nucleic acid binding | 1152 | 12 | 4.95 | 4 | 0.65983 | 0.00191 |

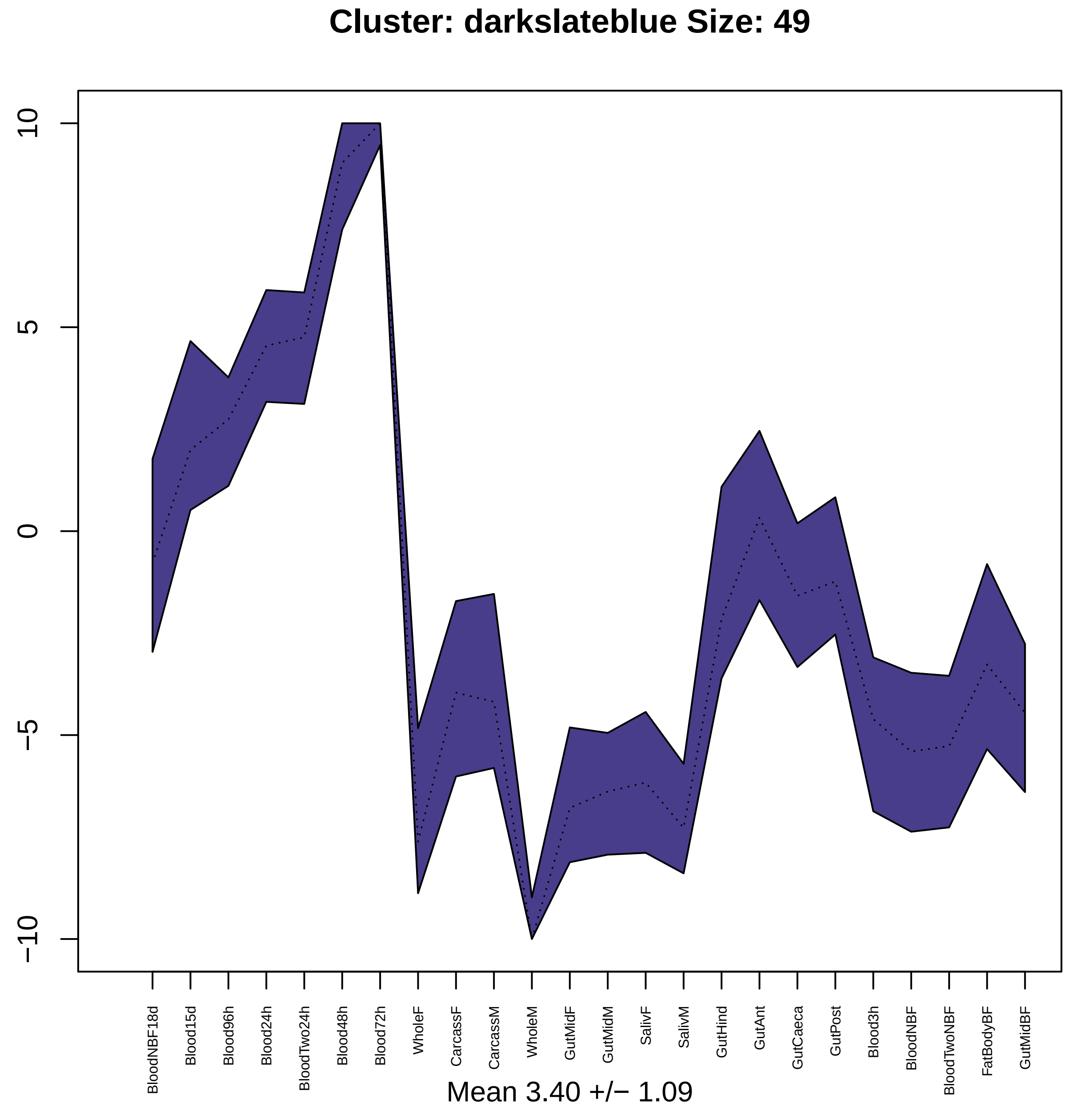
Cluster: darkslateblue Size: 49
10
5
0
−5
−10
BloodNBF18d
Blood15d
Blood96h
Blood24h
BloodTwo24h
Blood48h
Blood72h
WholeF
CarcassF
CarcassM
WholeM
GutMidF
GutMidM
SalivF
SalivM
GutHind
GutAnt
GutCaeca
GutPost
Blood3h
BloodNBF
BloodTwoNBF
FatBodyBF
GutMidBF
Mean 3.40 +/− 1.09

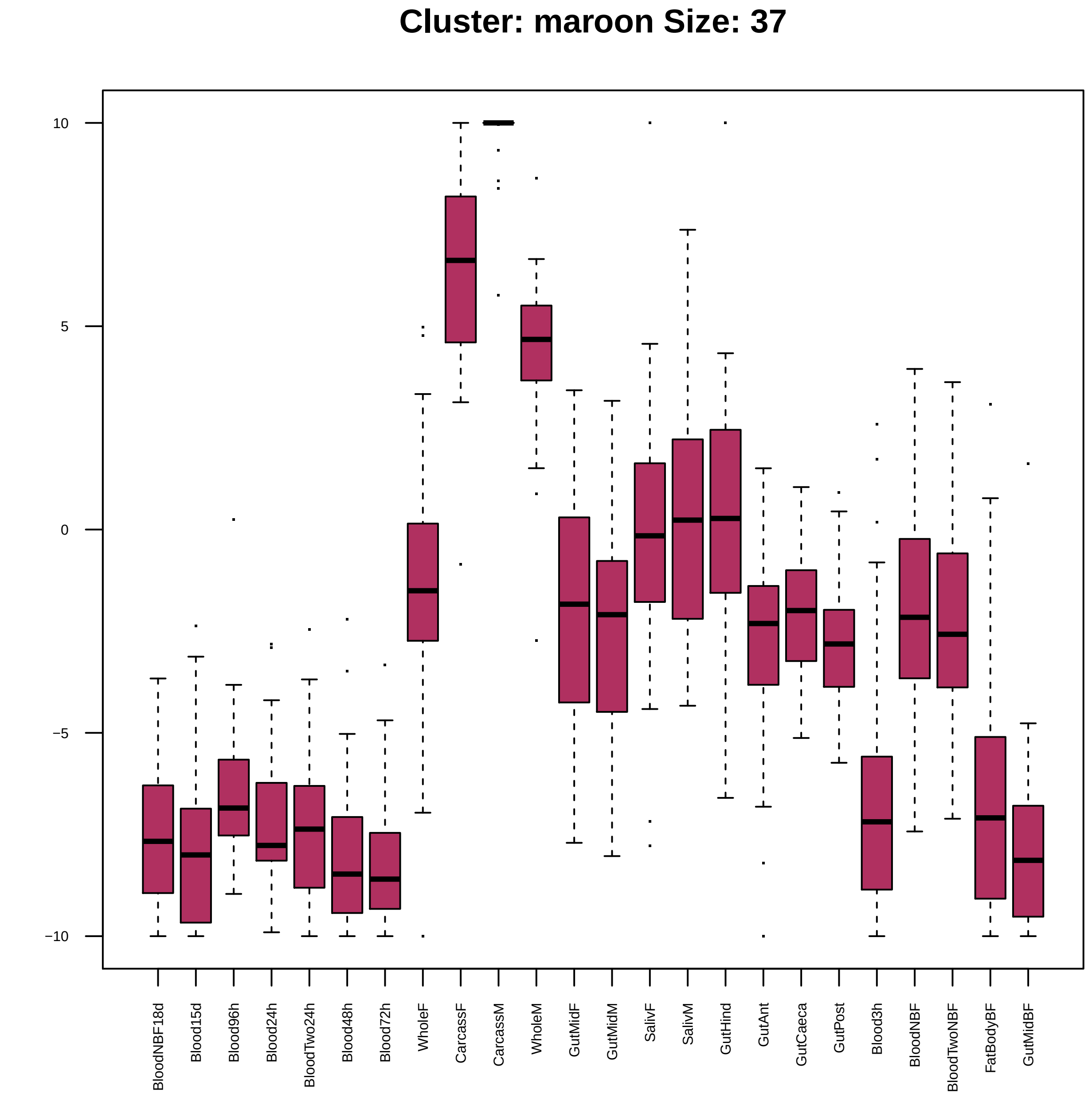
Cluster: maroon Size: 37
10
5
0
−5
−10
BloodNBF18d
Blood15d
Blood96h
Blood24h
BloodTwo24h
Blood48h
Blood72h
WholeF
CarcassF
CarcassM
WholeM
GutMidF
GutMidM
SalivF
SalivM
GutHind
GutAnt
GutCaeca
GutPost
Blood3h
BloodNBF
BloodTwoNBF
FatBodyBF
GutMidBF

| | GO.ID | BPCluster: maroon Size: 37 | Annotated | Significant | Expected | Rank in ClassicF | Weight01F | ClassicF |
|---|---|---|---|---|---|---|---|---|
| 1 | GO:0042775 | mitochondrial ATP synthesis coupled elec... | 29 | 4 | 0.11 | 17 | 0.00074 | 3.4e−06 |
| 2 | GO:0043648 | dicarboxylic acid metabolic process | 16 | 2 | 0.06 | 32 | 0.00158 | 0.0016 |
| 3 | GO:0006120 | mitochondrial electron transport, NADH t... | 17 | 2 | 0.06 | 33 | 0.00178 | 0.0018 |
| 4 | GO:0006099 | tricarboxylic acid cycle | 27 | 2 | 0.10 | 37 | 0.00450 | 0.0045 |
| 5 | GO:0022900 | electron transport chain | 37 | 5 | 0.14 | 9 | 0.00943 | 1.9e−07 |
| 6 | GO:0009060 | aerobic respiration | 31 | 3 | 0.12 | 30 | 0.01379 | 0.0002 |
| 7 | GO:0006733 | oxidoreduction coenzyme metabolic proces... | 34 | 2 | 0.13 | 45 | 0.02509 | 0.0071 |
| 16 | GO:0044237 | cellular metabolic process | 2862 | 17 | 10.77 | 47 | 0.05303 | 0.0091 |

| | GO.ID | MFCluster: maroon Size: 37 | Annotated | Significant | Expected | Rank in ClassicF | Weight01F | ClassicF |
|---|---|---|---|---|---|---|---|---|
| 1 | GO:0008137 | NADH dehydrogenase (ubiquinone) activity | 20 | 2 | 0.06 | 4 | 0.0018 | 0.00179 |
| 2 | GO:0016836 | hydro−lyase activity | 28 | 2 | 0.09 | 8 | 0.0035 | 0.00350 |
| 5 | GO:0016651 | oxidoreductase activity, acting on NAD(P... | 35 | 3 | 0.11 | 2 | 0.0289 | 0.00017 |
| 24 | GO:0003824 | catalytic activity | 3160 | 18 | 10.08 | 3 | 0.2428 | 0.00082 |

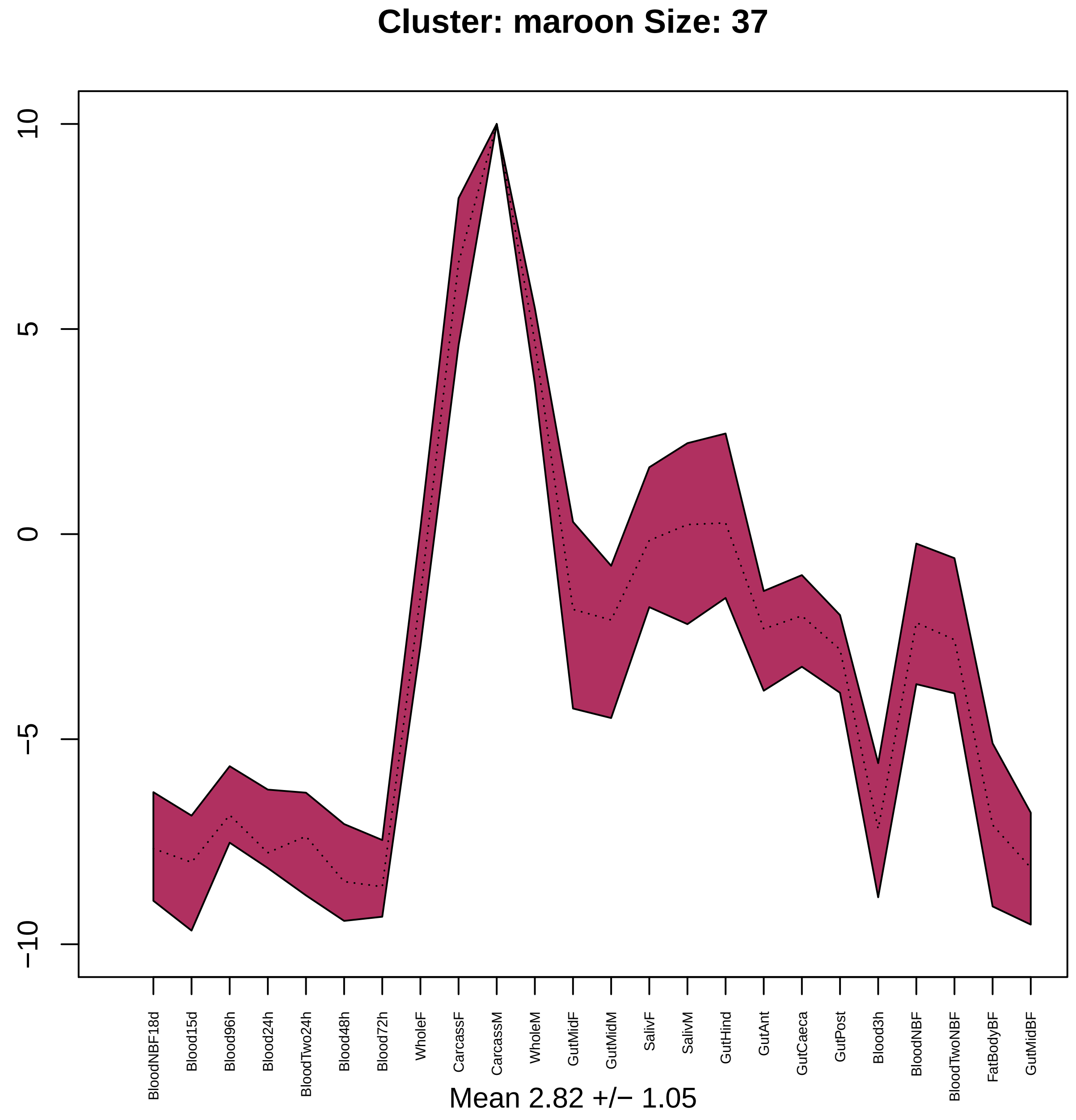
Cluster: maroon Size: 37
10
5
0
−5
−10
BloodNBF18d
Blood15d
Blood96h
Blood24h
BloodTwo24h
Blood48h
Blood72h
WholeF
CarcassF
CarcassM
WholeM
GutMidF
GutMidM
SalivF
SalivM
GutHind
GutAnt
GutCaeca
GutPost
Blood3h
BloodNBF
BloodTwoNBF
FatBodyBF
GutMidBF
Mean 2.82 +/− 1.05

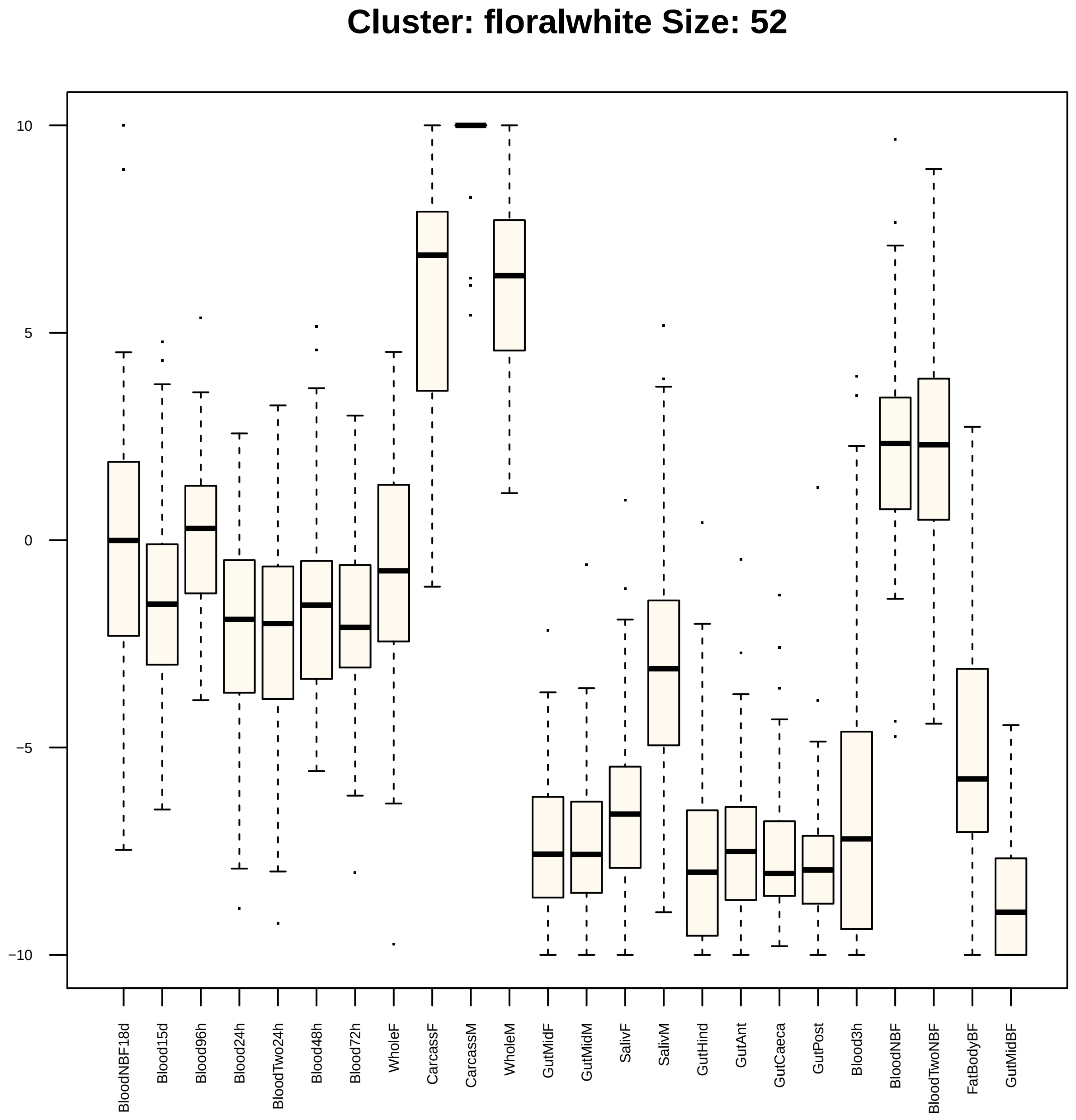

Cluster: floralwhite Size: 52
10
5
0
−5
−10
BloodNBF18d
Blood15d
Blood96h
Blood24h
BloodTwo24h
Blood48h
Blood72h
WholeF
CarcassF
CarcassM
WholeM
GutMidF
GutMidM
SalivF
SalivM
GutHind
GutAnt
GutCaeca
GutPost
Blood3h
BloodNBF
BloodTwoNBF
FatBodyBF
GutMidBF

| | GO.ID | BPCluster: floralwhite Size: 52 | Annotated | Significant | Expected | Rank in ClassicF | Weight01F | ClassicF |
|---|---|---|---|---|---|---|---|---|
| *1* | GO:0035335 | peptidyl−tyrosine dephosphorylation | 30 | 2 | 0.10 | 3 | 0.0047 | 0.0047 |
| *13* | GO:0009166 | nucleotide catabolic process | 25 | 2 | 0.09 | 1 | 0.0421 | 0.0032 |

| | GO.ID | MFCluster: floralwhite Size: 52 | Annotated | Significant | Expected | Rank in ClassicF | Weight01F | ClassicF |
|---|---|---|---|---|---|---|---|---|
| *1* | GO:0004725 | protein tyrosine phosphatase activity | 32 | 2 | 0.12 | 2 | 0.0063 | 0.0063 |
| *17* | GO:0016791 | phosphatase activity | 97 | 3 | 0.36 | 1 | 0.1249 | 0.0055 |

## Cluster: floralwhite Size: 52

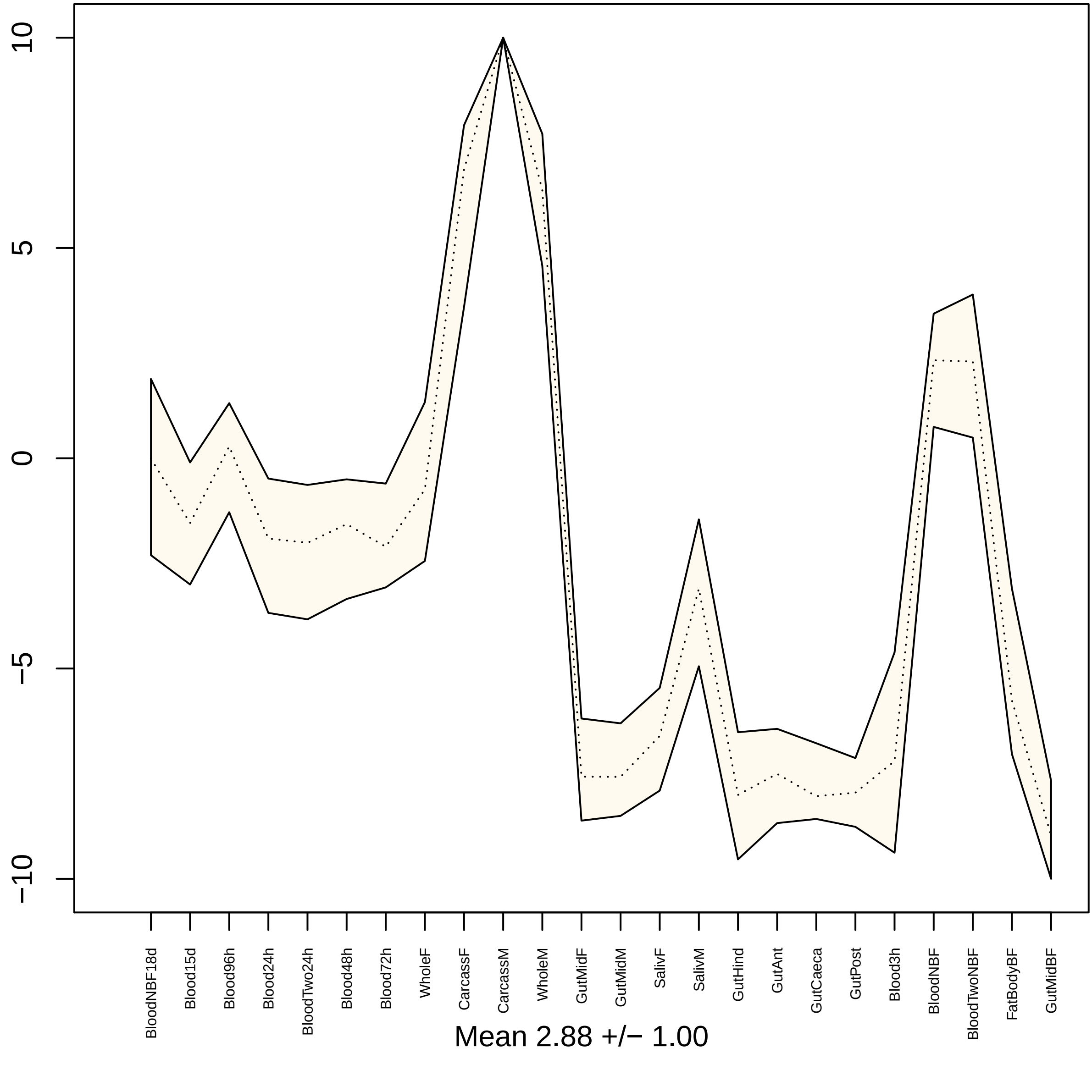

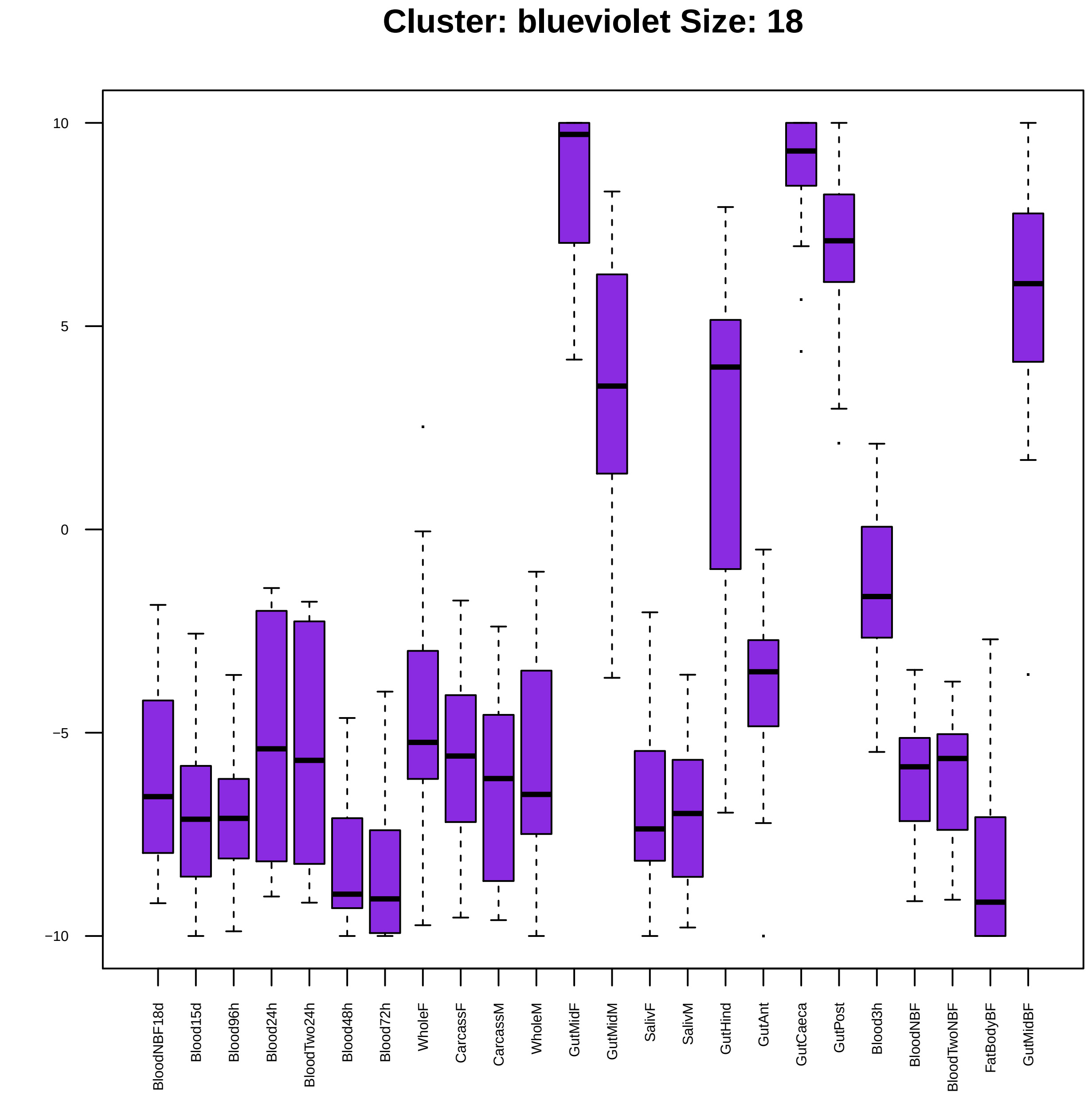
Cluster: blueviolet Size: 18
10
5
0
−5
−10
BloodNBF18d
Blood15d
Blood96h
Blood24h
BloodTwo24h
Blood48h
Blood72h
WholeF
CarcassF
CarcassM
WholeM
GutMidF
GutMidM
SalivF
SalivM
GutHind
GutAnt
GutCaeca
GutPost
Blood3h
BloodNBF
BloodTwoNBF
FatBodyBF
GutMidBF

| | GO.ID | BPCluster: blueviolet Size: 18 | Annotated | Significant | Expected | Rank in ClassicF | Weight01F | ClassicF |
|---|---|---|---|---|---|---|---|---|
| *1* | GO:0016311 | dephosphorylation | 98 | 3 | 0.25 | 1 | 0.0017 | 0.0017 |

| | GO.ID | MFCluster: blueviolet Size: 18 | Annotated | Significant | Expected | Rank in ClassicF | Weight01F | ClassicF |
|---|---|---|---|---|---|---|---|---|
| *1* | GO:0016791 | phosphatase activity | 97 | 3 | 0.19 | 1 | 0.00077 | 0.00077 |
| *9* | GO:0016788 | hydrolase activity, acting on ester bond... | 269 | 4 | 0.52 | 2 | 0.20357 | 0.00141 |
| *11* | GO:0016787 | hydrolase activity | 1445 | 8 | 2.81 | 4 | 0.54006 | 0.00241 |
| *19* | GO:0008238 | exopeptidase activity | 66 | 2 | 0.13 | 5 | 1.00000 | 0.00700 |

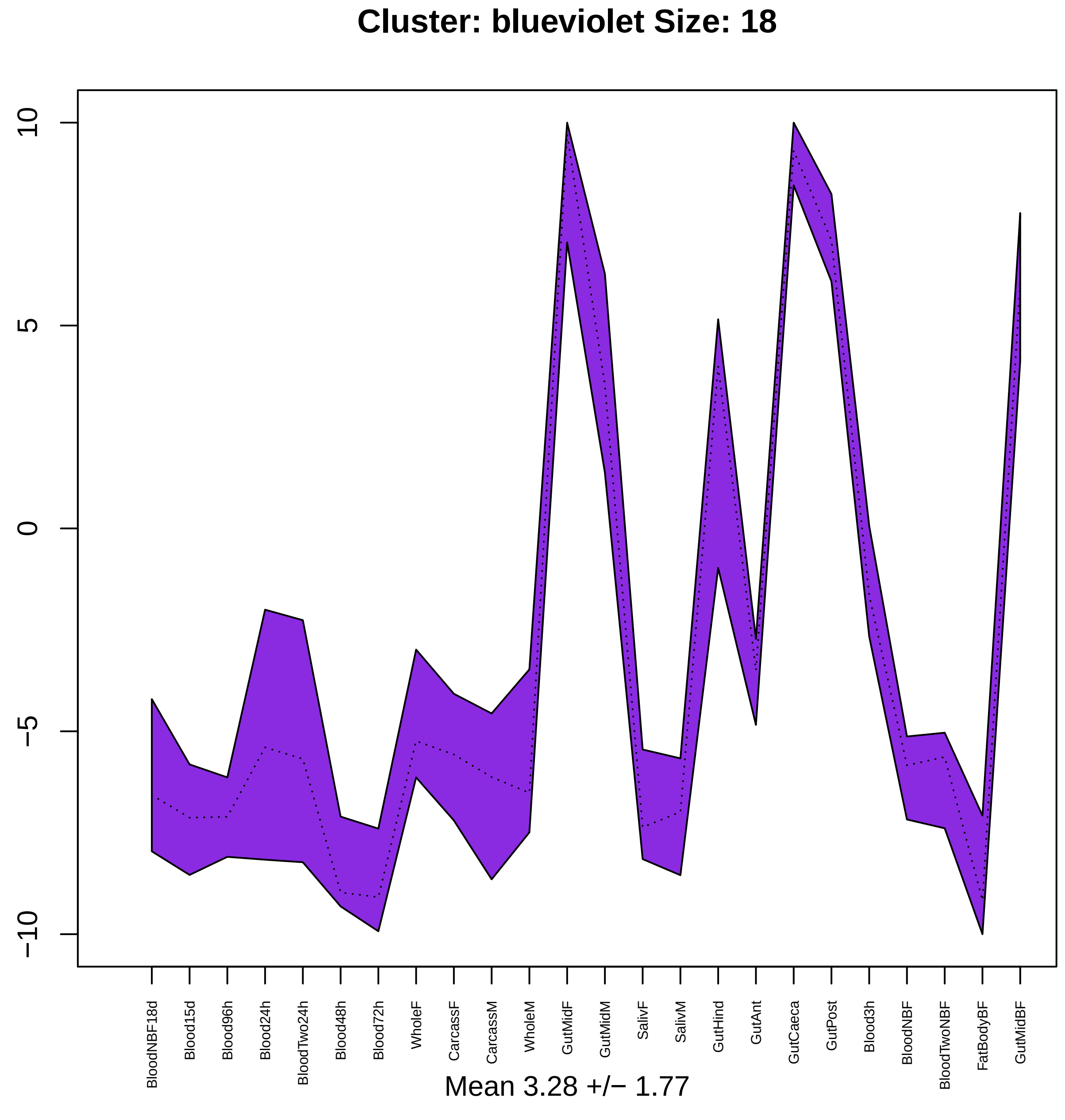
Cluster: blueviolet Size: 18
10
5
0
−5
−10
BloodNBF18d
Blood15d
Blood96h
Blood24h
BloodTwo24h
Blood48h
Blood72h
WholeF
CarcassF
CarcassM
WholeM
GutMidF
GutMidM
SalivF
SalivM
GutHind
GutAnt
GutCaeca
GutPost
Blood3h
BloodNBF
BloodTwoNBF
FatBodyBF
GutMidBF
Mean 3.28 +/− 1.77

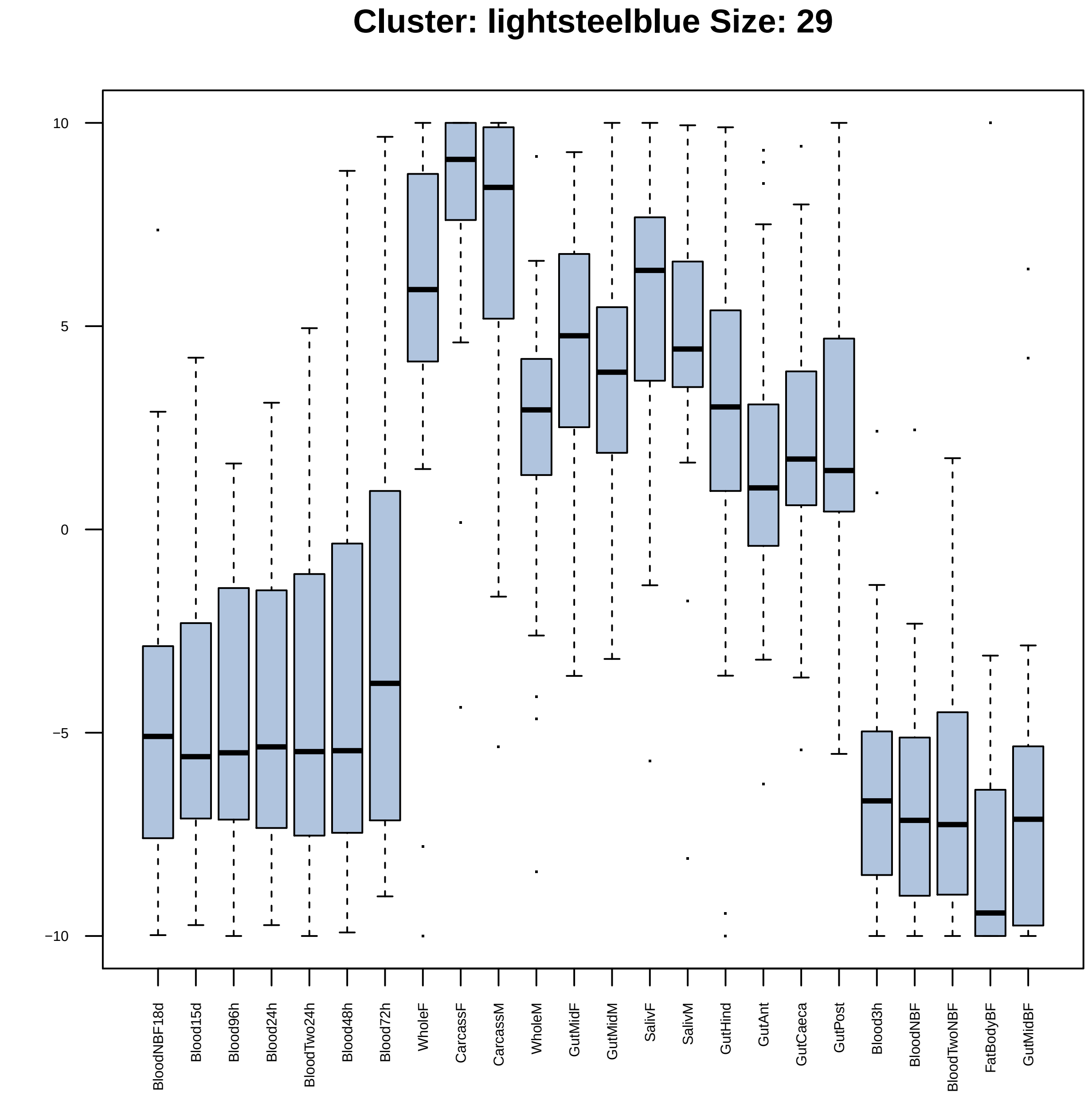
Cluster: lightsteelblue Size: 29
10
5
0
−5
−10
BloodNBF18d
Blood15d
Blood96h
Blood24h
BloodTwo24h
Blood48h
Blood72h
WholeF
CarcassF
CarcassM
WholeM
GutMidF
GutMidM
SalivF
SalivM
GutHind
GutAnt
GutCaeca
GutPost
Blood3h
BloodNBF
BloodTwoNBF
FatBodyBF
GutMidBF

| | GO.ID | BPCluster: lightsteelblue Size: 29 | Annotated | Significant | Expected | Rank in ClassicF | Weight01F | ClassicF |
|---|---|---|---|---|---|---|---|---|
| *1* | GO:0006979 | response to oxidative stress | 51 | 2 | 0.14 | 22 | 0.0088 | 0.00884 |
| *2* | GO:0006412 | translation | 289 | 5 | 0.82 | 6 | 0.0152 | 0.00097 |
| *3* | GO:0000398 | mRNA splicing, via spliceosome | 134 | 3 | 0.38 | 14 | 0.0246 | 0.00588 |

| | GO.ID | MFCluster: lightsteelblue Size: 29 | Annotated | Significant | Expected | Rank in ClassicF | Weight01F | ClassicF |
|---|---|---|---|---|---|---|---|---|
| *1* | GO:0003735 | structural constituent of ribosome | 146 | 3 | 0.43 | 1 | 0.0083 | 0.0083 |

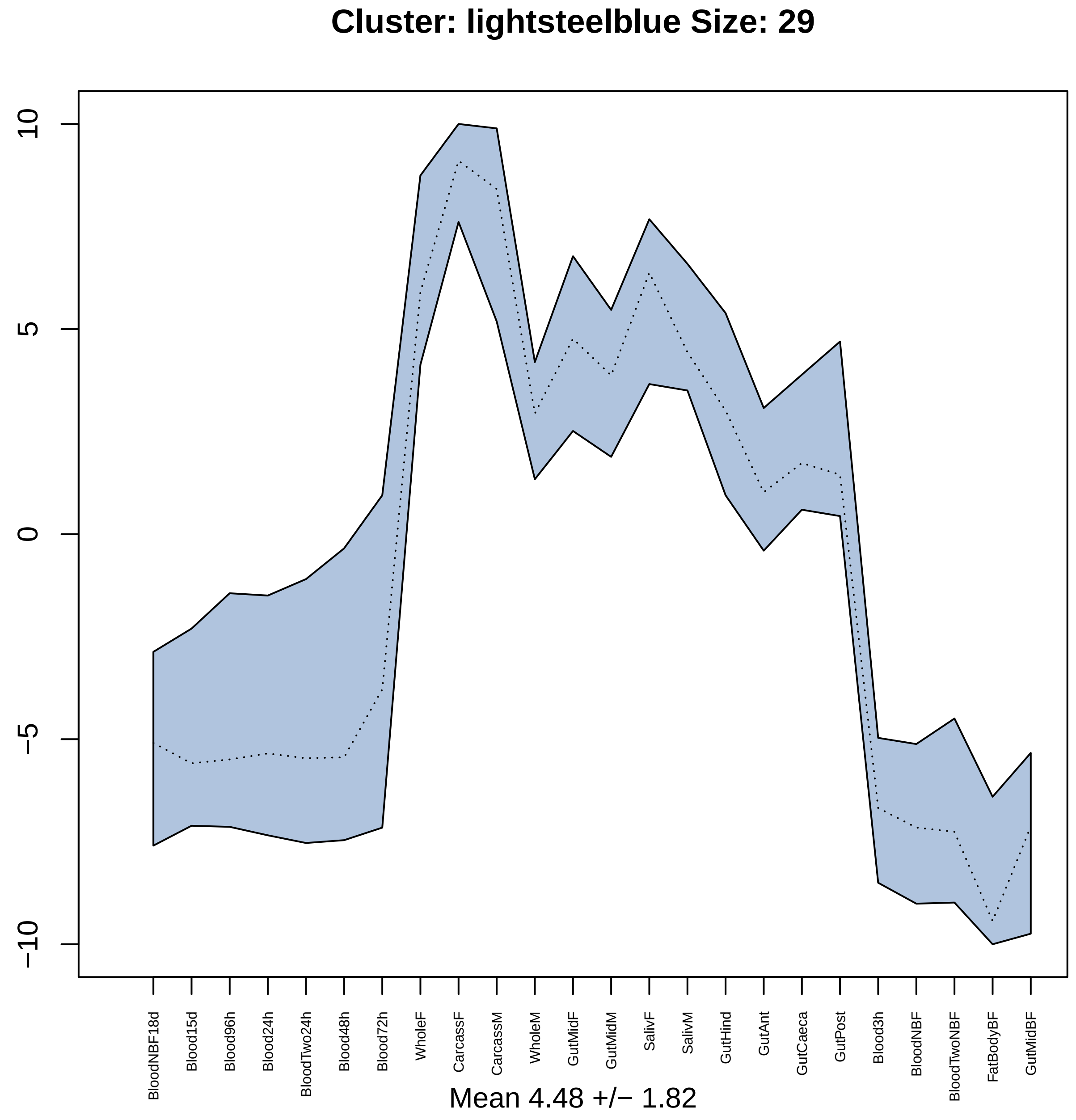

Cluster: lightsteelblue Size: 29
10
5
0
−5
−10
BloodNBF18d
Blood15d
Blood96h
Blood24h
BloodTwo24h
Blood48h
Blood72h
WholeF
CarcassF
CarcassM
WholeM
GutMidF
GutMidM
SalivF
SalivM
GutHind
GutAnt
GutCaeca
GutPost
Blood3h
BloodNBF
BloodTwoNBF
FatBodyBF
GutMidBF
Mean 4.48 +/− 1.82

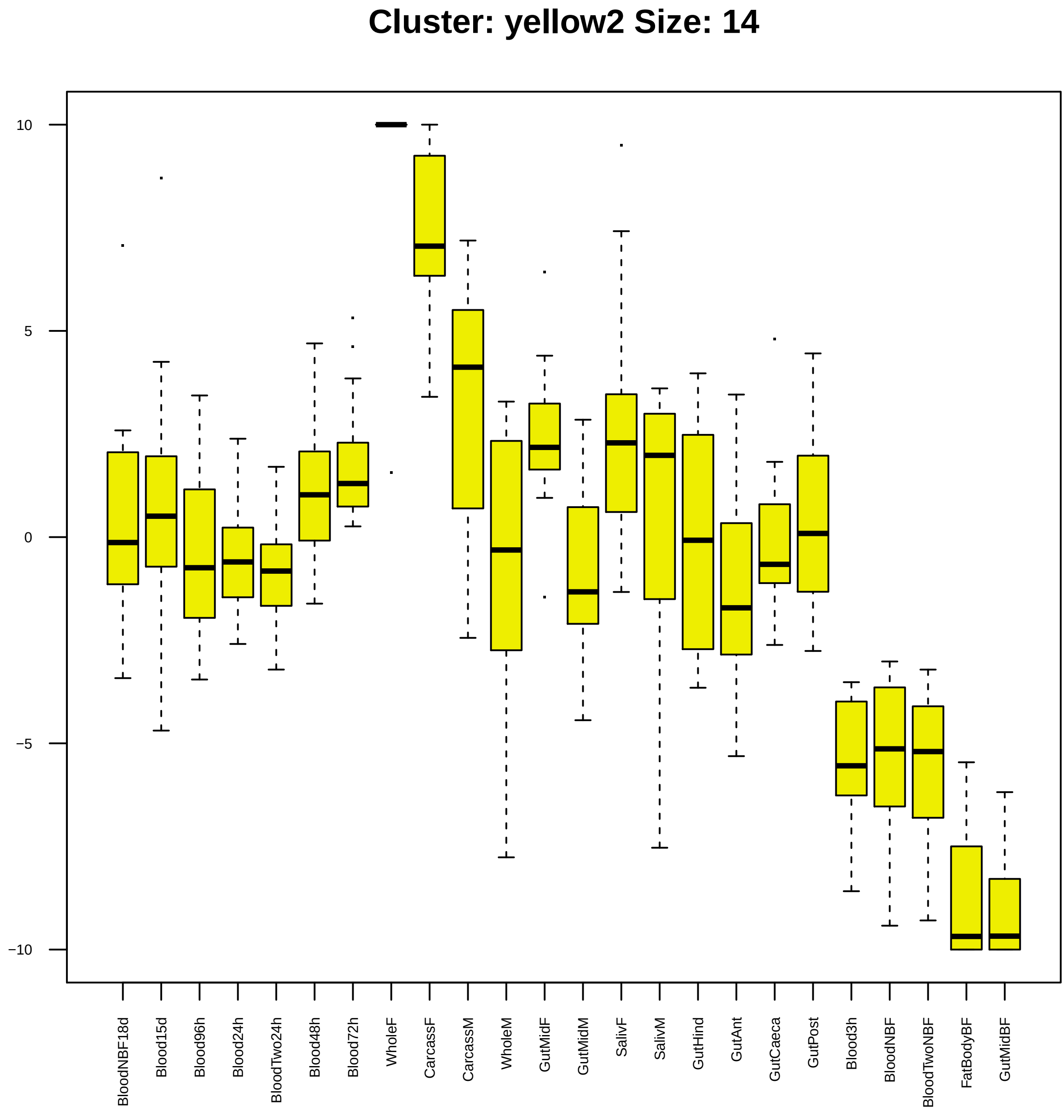

Cluster: yellow2 Size: 14
10
5
0
−5
−10
BloodNBF18d
Blood15d
Blood96h
Blood24h
BloodTwo24h
Blood48h
Blood72h
WholeF
CarcassF
CarcassM
WholeM
GutMidF
GutMidM
SalivF
SalivM
GutHind
GutAnt
GutCaeca
GutPost
Blood3h
BloodNBF
BloodTwoNBF
FatBodyBF
GutMidBF

| | GO.ID | BPCluster: yellow2 Size: 14 | Annotated | Significant | Expected | Rank in ClassicF | Weight01F | ClassicF |
|---|---|---|---|---|---|---|---|---|
| *13* | GO:0016070 | RNA metabolic process | 906 | 4 | 0.85 | 1 | 1.000 | 0.0048 |

| | GO.ID | MFCluster: yellow2 Size: 14 | Annotated | Significant | Expected | Rank in ClassicF | Weight01F | ClassicF |
|---|---|---|---|---|---|---|---|---|
| *7* | GO:0140098 | catalytic activity, acting on RNA | 165 | 2 | 0.09 | 5 | 1.000 | 0.00303 |
| *11* | GO:0016779 | nucleotidyltransferase activity | 61 | 2 | 0.03 | 4 | 1.000 | 0.00042 |
| *13* | GO:0016740 | transferase activity | 911 | 3 | 0.51 | 6 | 1.000 | 0.00729 |

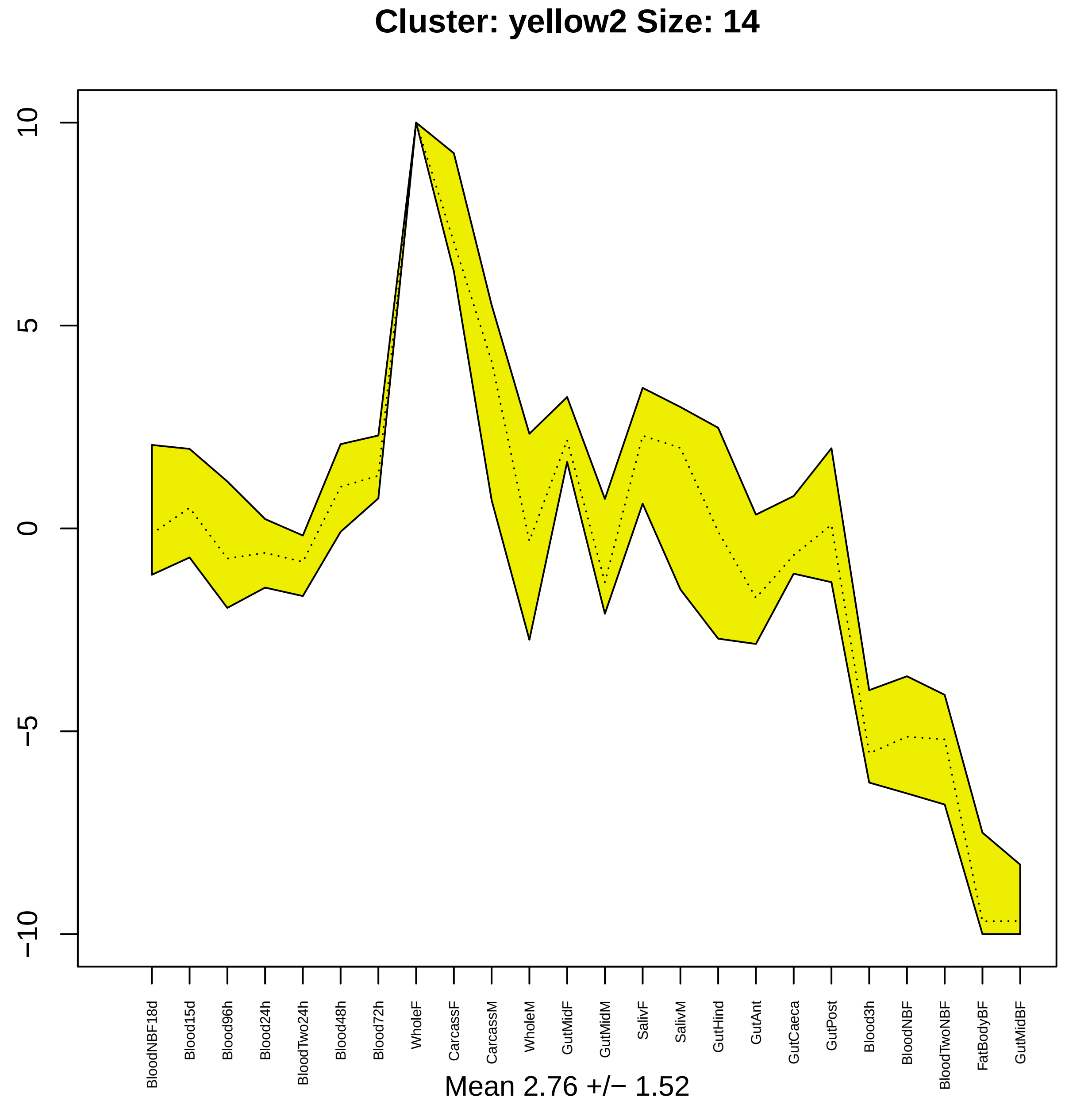

Cluster: yellow2 Size: 14
10
5
0
−5
−10
BloodNBF18d
Blood15d
Blood96h
Blood24h
BloodTwo24h
Blood48h
Blood72h
WholeF
CarcassF
CarcassM
WholeM
GutMidF
GutMidM
SalivF
SalivM
GutHind
GutAnt
GutCaeca
GutPost
Blood3h
BloodNBF
BloodTwoNBF
FatBodyBF
GutMidBF
Mean 2.76 +/− 1.52

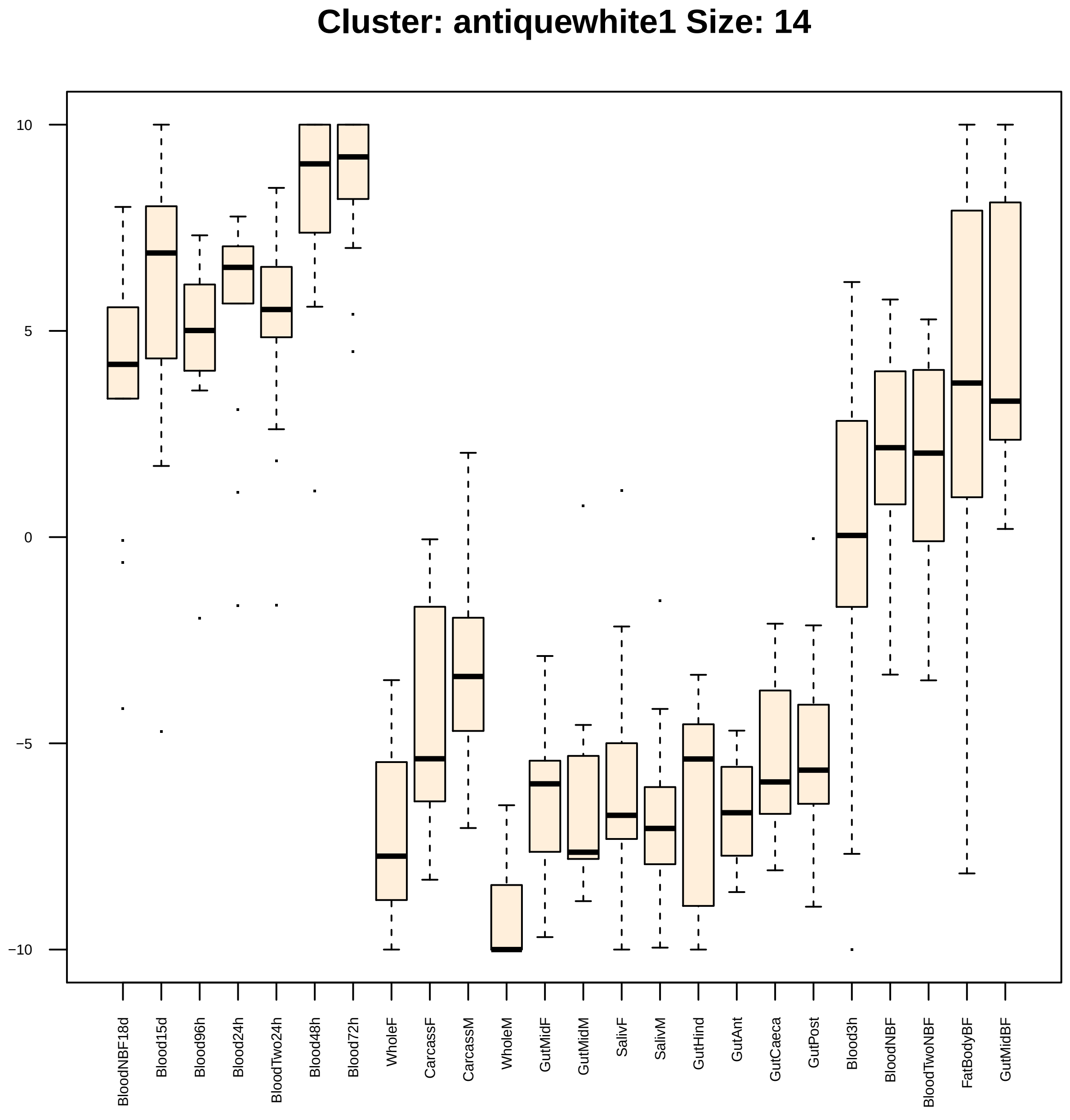
Cluster: antiquewhite1 Size: 14
10
5
0
−5
−10
BloodNBF18d
Blood15d
Blood96h
Blood24h
BloodTwo24h
Blood48h
Blood72h
WholeF
CarcassF
CarcassM
WholeM
GutMidF
GutMidM
SalivF
SalivM
GutHind
GutAnt
GutCaeca
GutPost
Blood3h
BloodNBF
BloodTwoNBF
FatBodyBF
GutMidBF

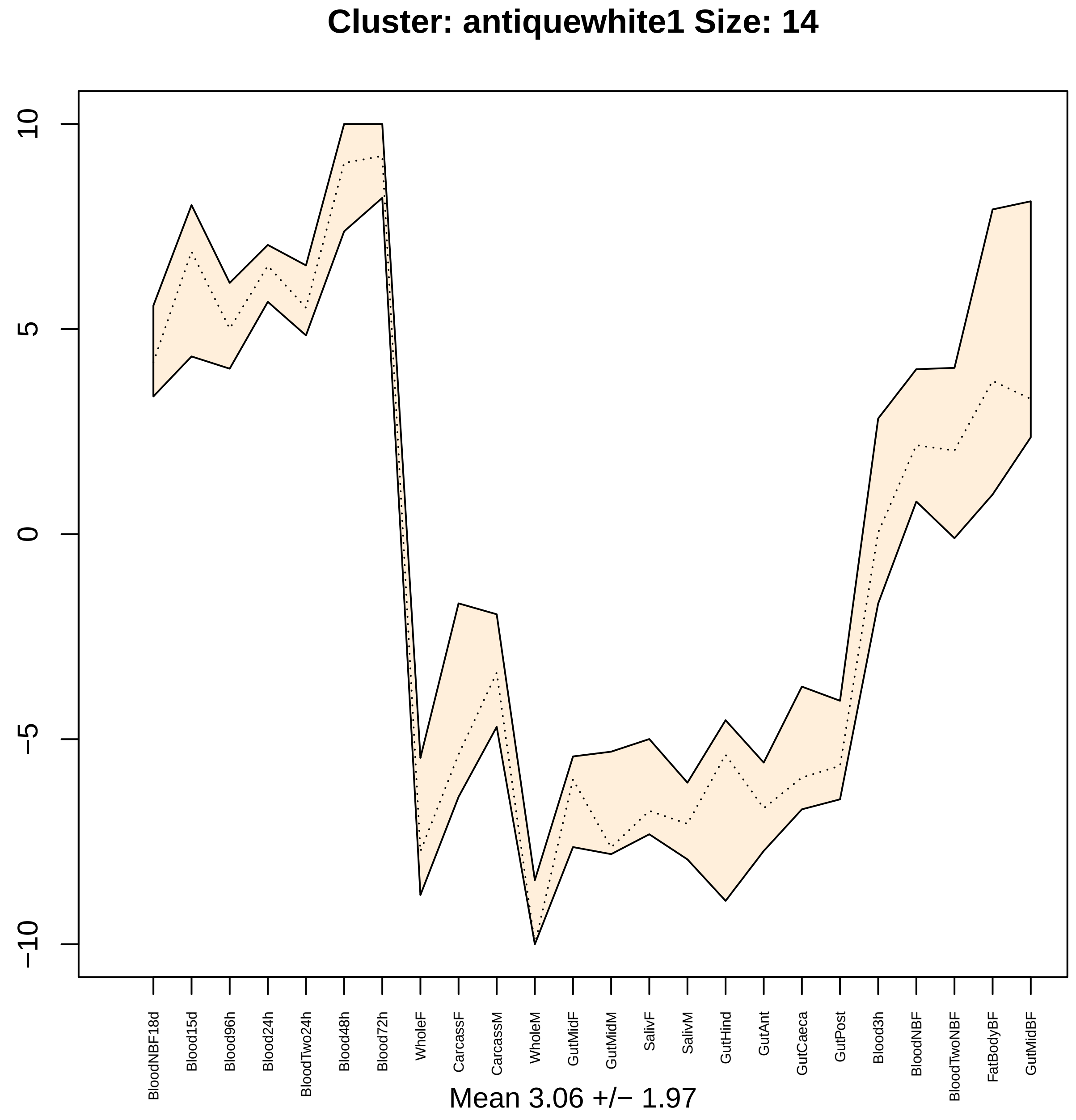
Cluster: antiquewhite1 Size: 14
10
5
0
−5
−10
BloodNBF18d
Blood15d
Blood96h
Blood24h
BloodTwo24h
Blood48h
Blood72h
WholeF
CarcassF
CarcassM
WholeM
GutMidF
GutMidM
SalivF
SalivM
GutHind
GutAnt
GutCaeca
GutPost
Blood3h
BloodNBF
BloodTwoNBF
FatBodyBF
GutMidBF
Mean 3.06 +/− 1.97

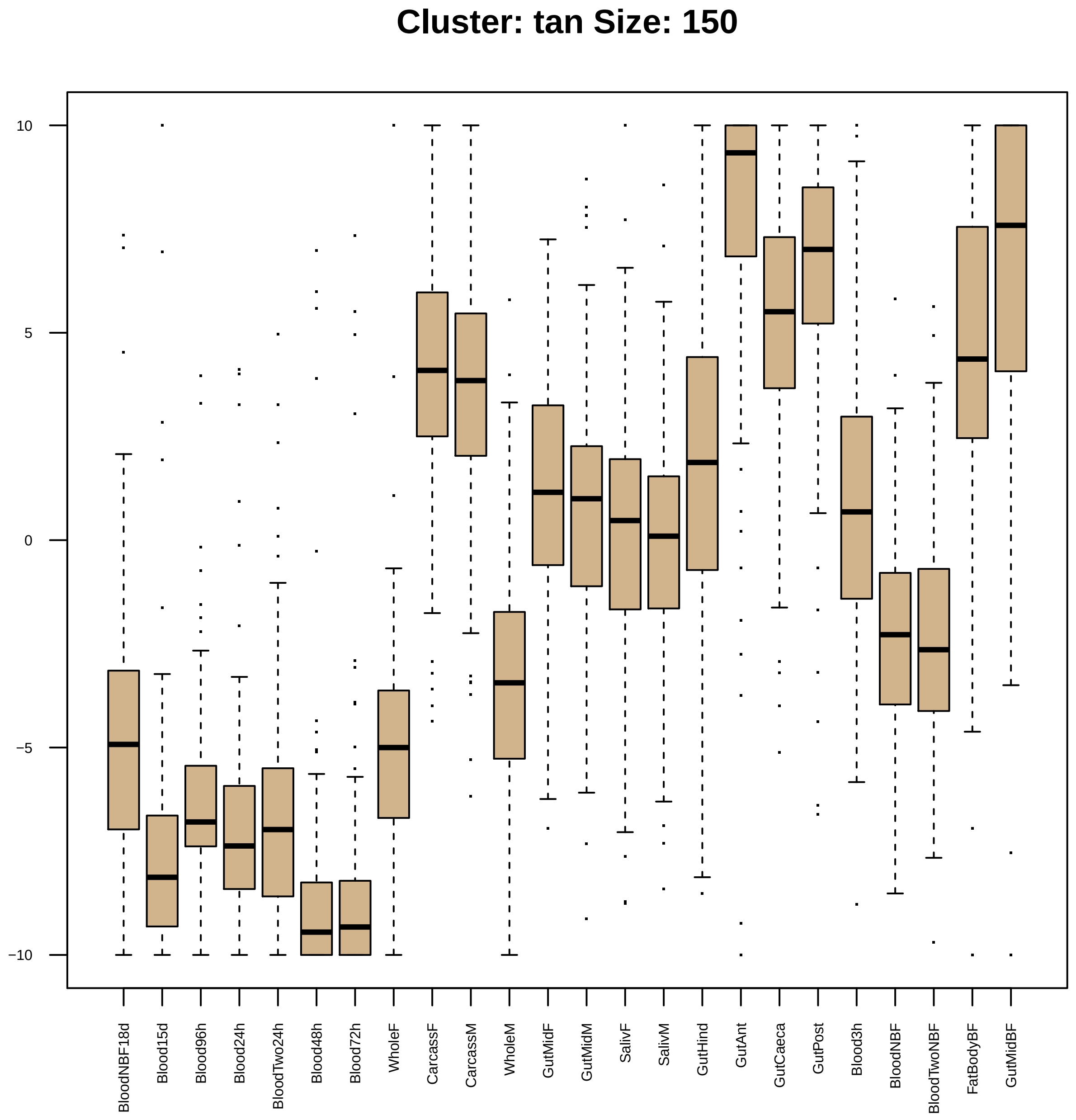
Cluster: tan Size: 150
10
5
0
−5
−10
BloodNBF18d
Blood15d
Blood96h
Blood24h
BloodTwo24h
Blood48h
Blood72h
WholeF
CarcassF
CarcassM
WholeM
GutMidF
GutMidM
SalivF
SalivM
GutHind
GutAnt
GutCaeca
GutPost
Blood3h
BloodNBF
BloodTwoNBF
FatBodyBF
GutMidBF

| | GO.ID | BPCluster: tan Size: 150 | Annotated | Significant | Expected | Rank in ClassicF | Weight01F | ClassicF |
|---|---|---|---|---|---|---|---|---|
| *4* | GO:0035235 | ionotropic glutamate receptor signaling ... | 28 | 3 | 0.34 | 22 | 0.0046 | 0.0046 |
| *11* | GO:0048749 | compound eye development | 157 | 7 | 1.92 | 17 | 0.0419 | 0.0029 |

| | GO.ID | MFCluster: tan Size: 150 | Annotated | Significant | Expected | Rank in ClassicF | Weight01F | ClassicF |
|---|---|---|---|---|---|---|---|---|
| 3 | GO:0005549 | odorant binding | 118 | 8 | 1.57 | 6 | 0.00016 | 0.00016 |
| 4 | GO:0004970 | ionotropic glutamate receptor activity | 29 | 3 | 0.39 | 19 | 0.00651 | 0.00651 |
| 5 | GO:0005234 | extracellularly glutamate−gated ion chan... | 29 | 3 | 0.39 | 20 | 0.00651 | 0.00651 |
| 6 | GO:0022835 | transmitter−gated channel activity | 30 | 4 | 0.40 | 9 | 0.01295 | 0.00062 |
| 8 | GO:0005230 | extracellular ligand−gated ion channel a... | 51 | 5 | 0.68 | 8 | 0.02972 | 0.00055 |
| 11 | GO:0003700 | DNA−binding transcription factor activit... | 282 | 10 | 3.76 | 16 | 0.05047 | 0.00413 |
| 23 | GO:0030594 | neurotransmitter receptor activity | 45 | 5 | 0.60 | 7 | 0.17606 | 0.00030 |

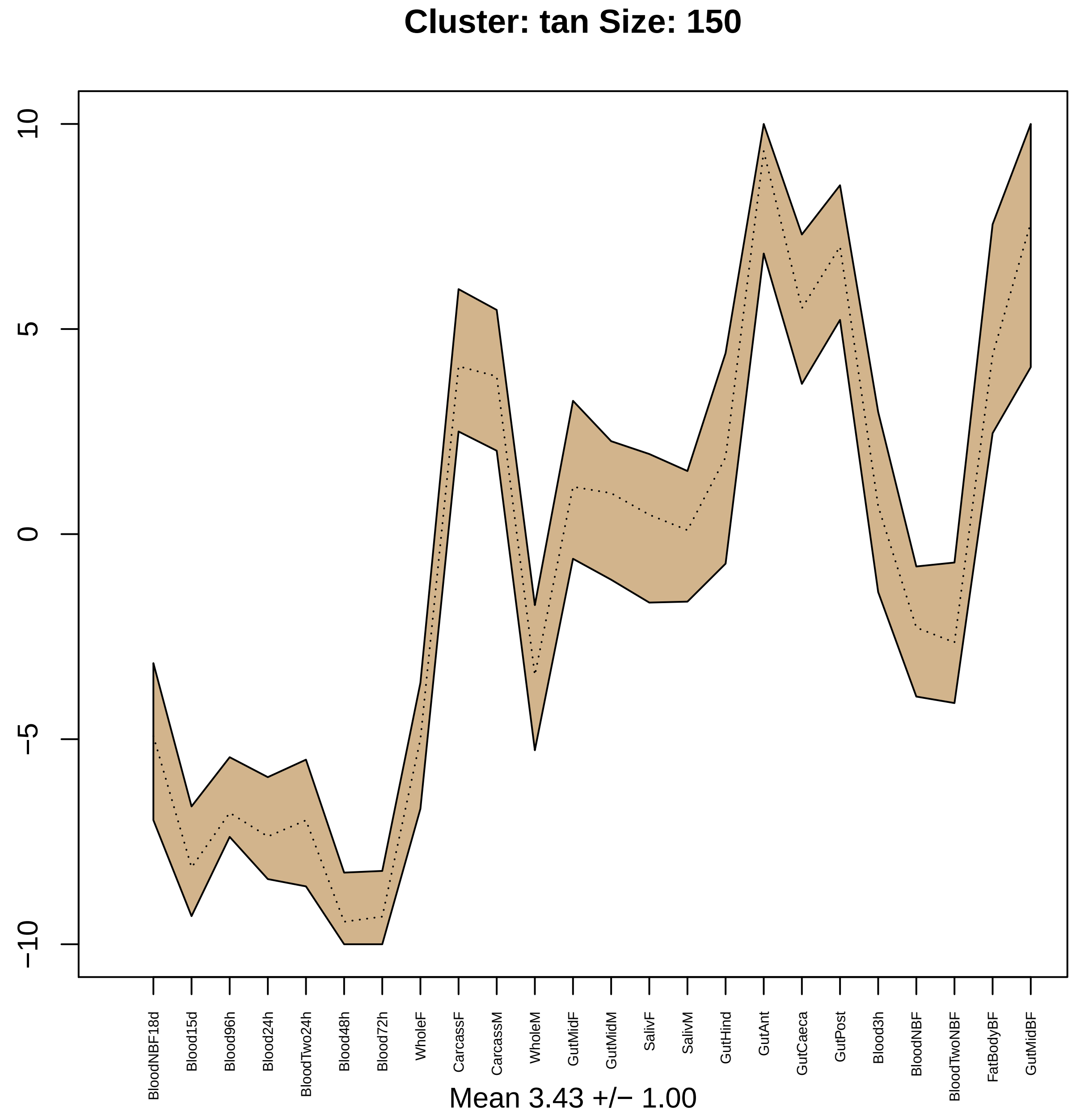

Cluster: tan Size: 150
10
5
0
−5
−10
BloodNBF18d
Blood15d
Blood96h
Blood24h
BloodTwo24h
Blood48h
Blood72h
WholeF
CarcassF
CarcassM
WholeM
GutMidF
GutMidM
SalivF
SalivM
GutHind
GutAnt
GutCaeca
GutPost
Blood3h
BloodNBF
BloodTwoNBF
FatBodyBF
GutMidBF
Mean 3.43 +/− 1.00

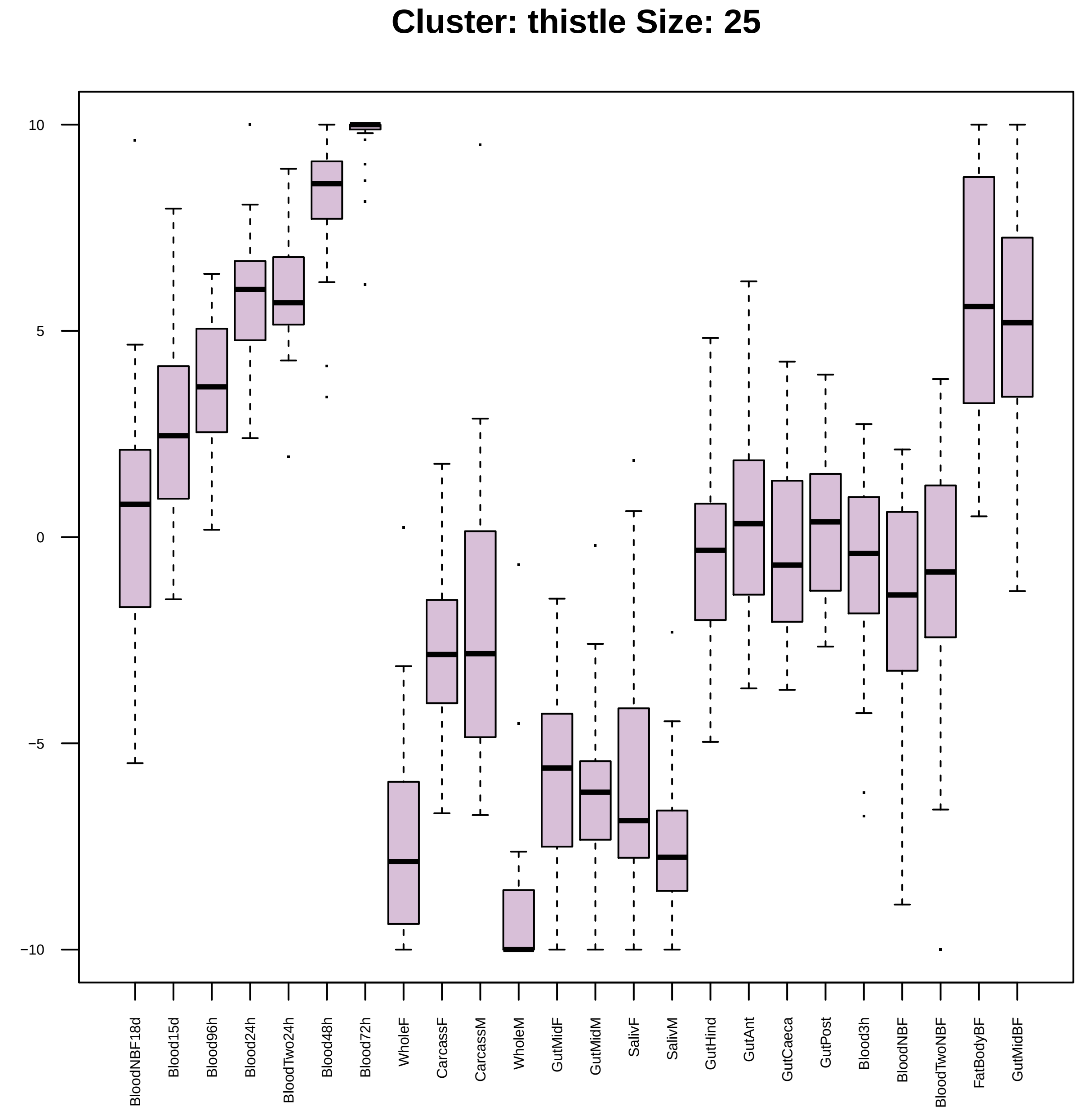

Cluster: thistle Size: 25
10
5
0
−5
−10
BloodNBF18d
Blood15d
Blood96h
Blood24h
BloodTwo24h
Blood48h
Blood72h
WholeF
CarcassF
CarcassM
WholeM
GutMidF
GutMidM
SalivF
SalivM
GutHind
GutAnt
GutCaeca
GutPost
Blood3h
BloodNBF
BloodTwoNBF
FatBodyBF
GutMidBF

| | GO.ID | BPCluster: thistle Size: 25 | Annotated | Significant | Expected | Rank in ClassicF | Weight01F | ClassicF |
|---|---|---|---|---|---|---|---|---|
| 1 | GO:0110110 | positive regulation of animal organ morp... | 12 | 2 | 0.03 | 6 | 0.00043 | 0.00043 |
| 2 | GO:0030833 | regulation of actin filament polymerizat... | 21 | 2 | 0.06 | 13 | 0.00136 | 0.00136 |
| 3 | GO:0008039 | synaptic target recognition | 21 | 2 | 0.06 | 14 | 0.00136 | 0.00136 |
| 4 | GO:0008104 | protein localization | 313 | 3 | 0.83 | 174 | 0.00429 | 0.04777 |
| 5 | GO:0043547 | positive regulation of GTPase activity | 42 | 2 | 0.11 | 48 | 0.00541 | 0.00541 |
| 6 | GO:0007264 | small GTPase mediated signal transductio... | 153 | 3 | 0.41 | 56 | 0.00696 | 0.00719 |
| 7 | GO:0110053 | regulation of actin filament organizatio... | 27 | 3 | 0.07 | 1 | 0.00941 | 4.4e−05 |
| 9 | GO:0044093 | positive regulation of molecular functio... | 79 | 3 | 0.21 | 10 | 0.02122 | 0.00110 |
| 30 | GO:0009266 | response to temperature stimulus | 38 | 2 | 0.10 | 37 | 0.03472 | 0.00444 |

| | GO.ID | MFCluster: thistle Size: 25 | Annotated | Significant | Expected | Rank in ClassicF | Weight01F | ClassicF |
|---|---|---|---|---|---|---|---|---|
| *1* | GO:0008270 | zinc ion binding | 522 | 6 | 1.45 | 1 | 0.0023 | 0.0023 |

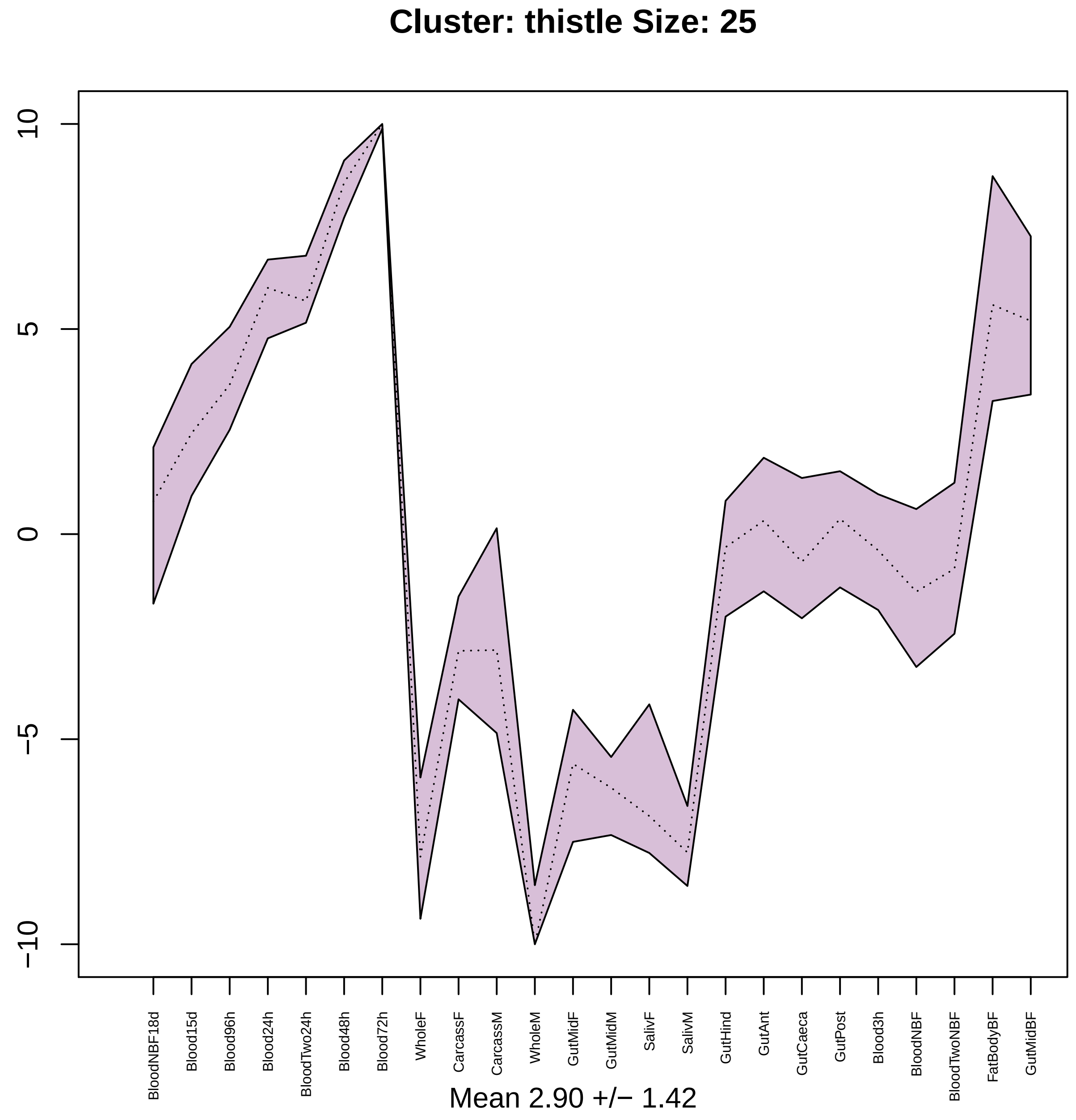

Cluster: thistle Size: 25
10
5
0
−5
−10
BloodNBF18d
Blood15d
Blood96h
Blood24h
BloodTwo24h
Blood48h
Blood72h
WholeF
CarcassF
CarcassM
WholeM
GutMidF
GutMidM
SalivF
SalivM
GutHind
GutAnt
GutCaeca
GutPost
Blood3h
BloodNBF
BloodTwoNBF
FatBodyBF
GutMidBF
Mean 2.90 +/− 1.42

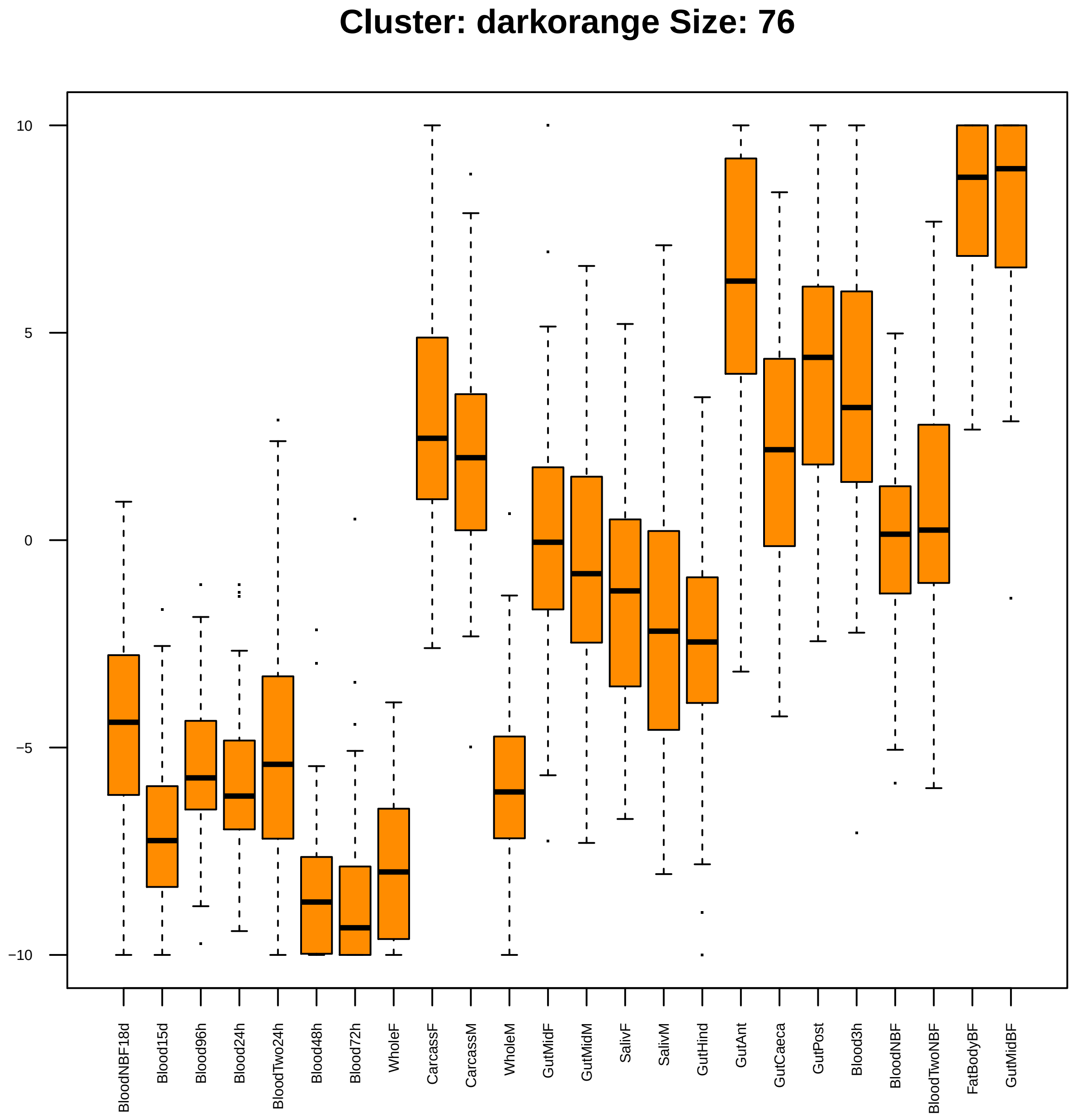

Cluster: darkorange Size: 76
10
5
0
−5
−10
BloodNBF18d
Blood15d
Blood96h
Blood24h
BloodTwo24h
Blood48h
Blood72h
WholeF
CarcassF
CarcassM
WholeM
GutMidF
GutMidM
SalivF
SalivM
GutHind
GutAnt
GutCaeca
GutPost
Blood3h
BloodNBF
BloodTwoNBF
FatBodyBF
GutMidBF

|  | GO.ID | BPCluster: darkorange Size: 76 | Annotated | Significant | Expected | Rank in ClassicF | Weight01F | ClassicF |
|---|---|---|---|---|---|---|---|---|
| 1 | GO:0000212 | meiotic spindle organization | 13 | 2 | 0.08 | 4 | 0.0030 | 0.0030 |
| 2 | GO:0007419 | ventral cord development | 15 | 2 | 0.10 | 5 | 0.0040 | 0.0040 |
| 3 | GO:0007112 | male meiosis cytokinesis | 16 | 2 | 0.10 | 6 | 0.0046 | 0.0046 |
| 4 | GO:0003007 | heart morphogenesis | 17 | 2 | 0.11 | 7 | 0.0052 | 0.0052 |

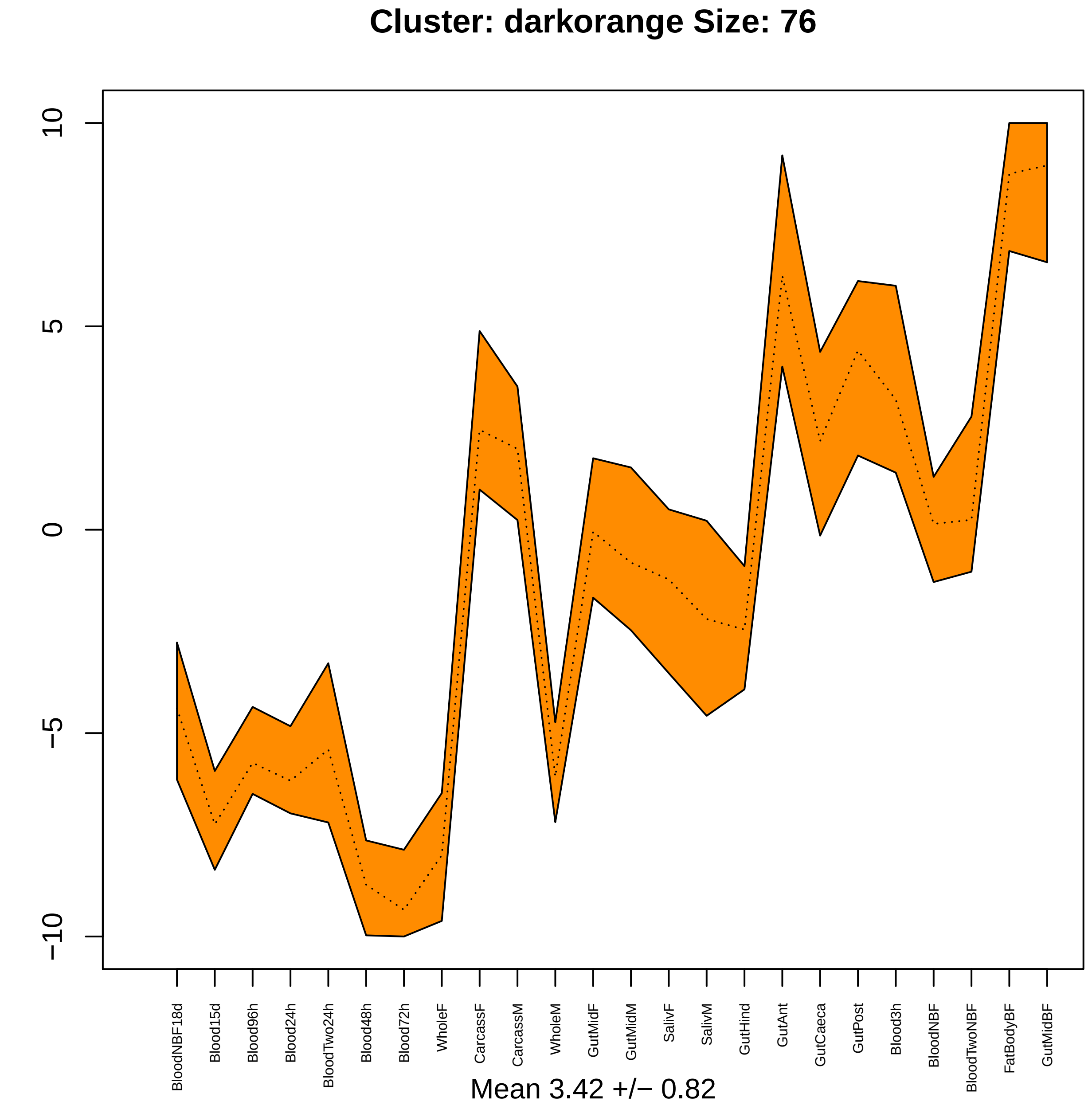

Cluster: darkorange Size: 76
10
5
0
−5
−10
BloodNBF18d
Blood15d
Blood96h
Blood24h
BloodTwo24h
Blood48h
Blood72h
WholeF
CarcassF
CarcassM
WholeM
GutMidF
GutMidM
SalivF
SalivM
GutHind
GutAnt
GutCaeca
GutPost
Blood3h
BloodNBF
BloodTwoNBF
FatBodyBF
GutMidBF
Mean 3.42 +/− 0.82

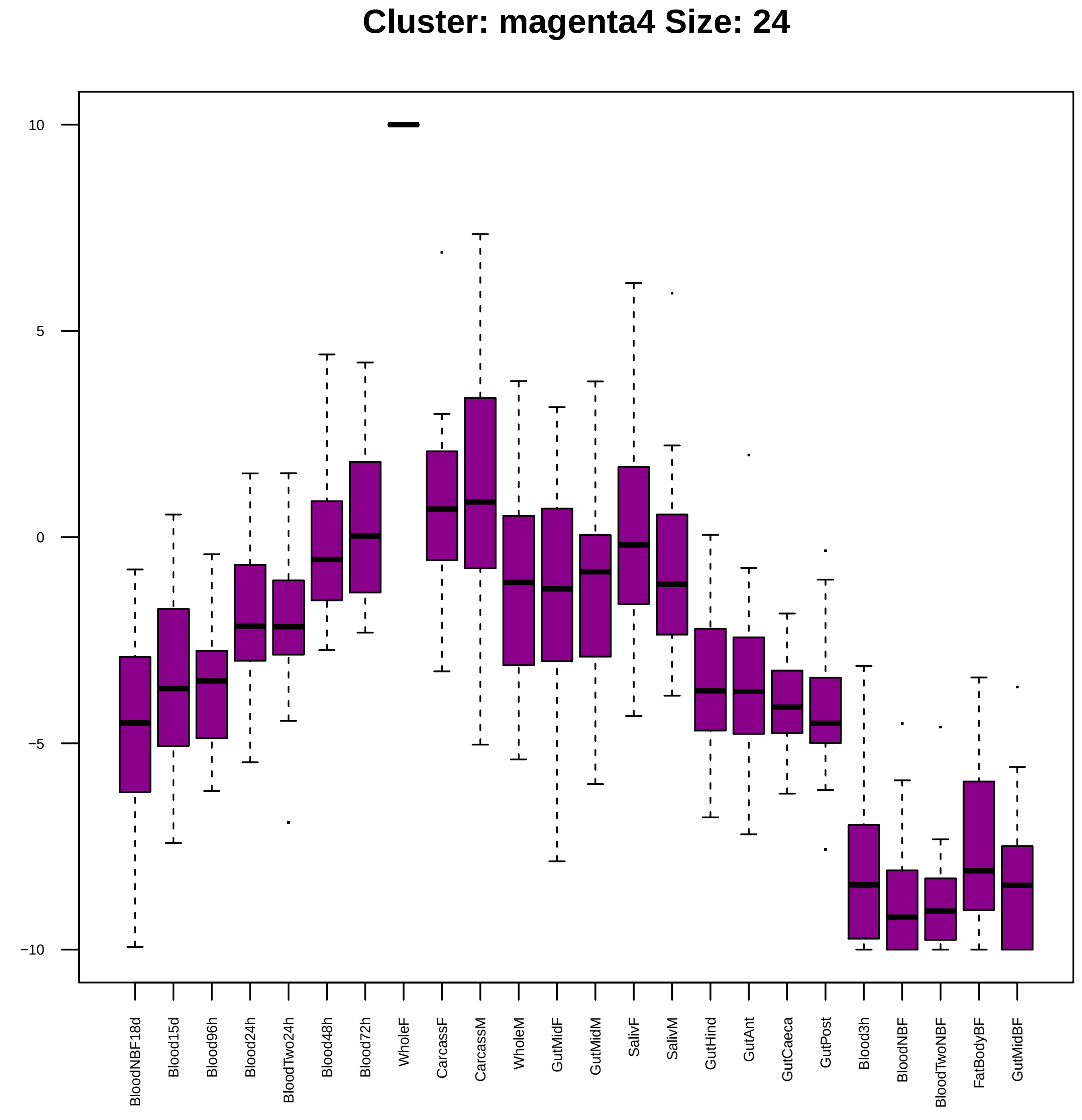

Cluster: magenta4 Size: 24
10
5
0
−5
−10
BloodNBF18d
Blood15d
Blood96h
Blood24h
BloodTwo24h
Blood48h
Blood72h
WholeF
CarcassF
CarcassM
WholeM
GutMidF
GutMidM
SalivF
SalivM
GutHind
GutAnt
GutCaeca
GutPost
Blood3h
BloodNBF
BloodTwoNBF
FatBodyBF
GutMidBF

| | GO.ID | BPCluster: magenta4 Size: 24 | Annotated | Significant | Expected | Rank in ClassicF | Weight01F | ClassicF |
|---|---|---|---|---|---|---|---|---|
| *1* | GO:0032774 | RNA biosynthetic process | 565 | 5 | 1.51 | 23 | 0.0067 | 0.0135 |
| *2* | GO:0019941 | modification−dependent protein catabolic... | 91 | 3 | 0.24 | 3 | 0.0071 | 0.0017 |
| *6* | GO:0031647 | regulation of protein stability | 34 | 2 | 0.09 | 9 | 0.0323 | 0.0036 |
| *14* | GO:0051276 | chromosome organization | 251 | 4 | 0.67 | 10 | 0.0425 | 0.0037 |

| | GO.ID | MFCluster: magenta4 Size: 24 | Annotated | Significant | Expected | Rank in ClassicF | Weight01F | ClassicF |
|---|---|---|---|---|---|---|---|---|
| 1 | GO:0003713 | transcription coactivator activity | 29 | 2 | 0.07 | 2 | 0.0023 | 0.00230 |
| 5 | GO:0003712 | transcription coregulator activity | 44 | 3 | 0.11 | 1 | 0.0329 | 0.00016 |
| 9 | GO:0016779 | nucleotidyltransferase activity | 61 | 2 | 0.15 | 4 | 0.0464 | 0.00988 |

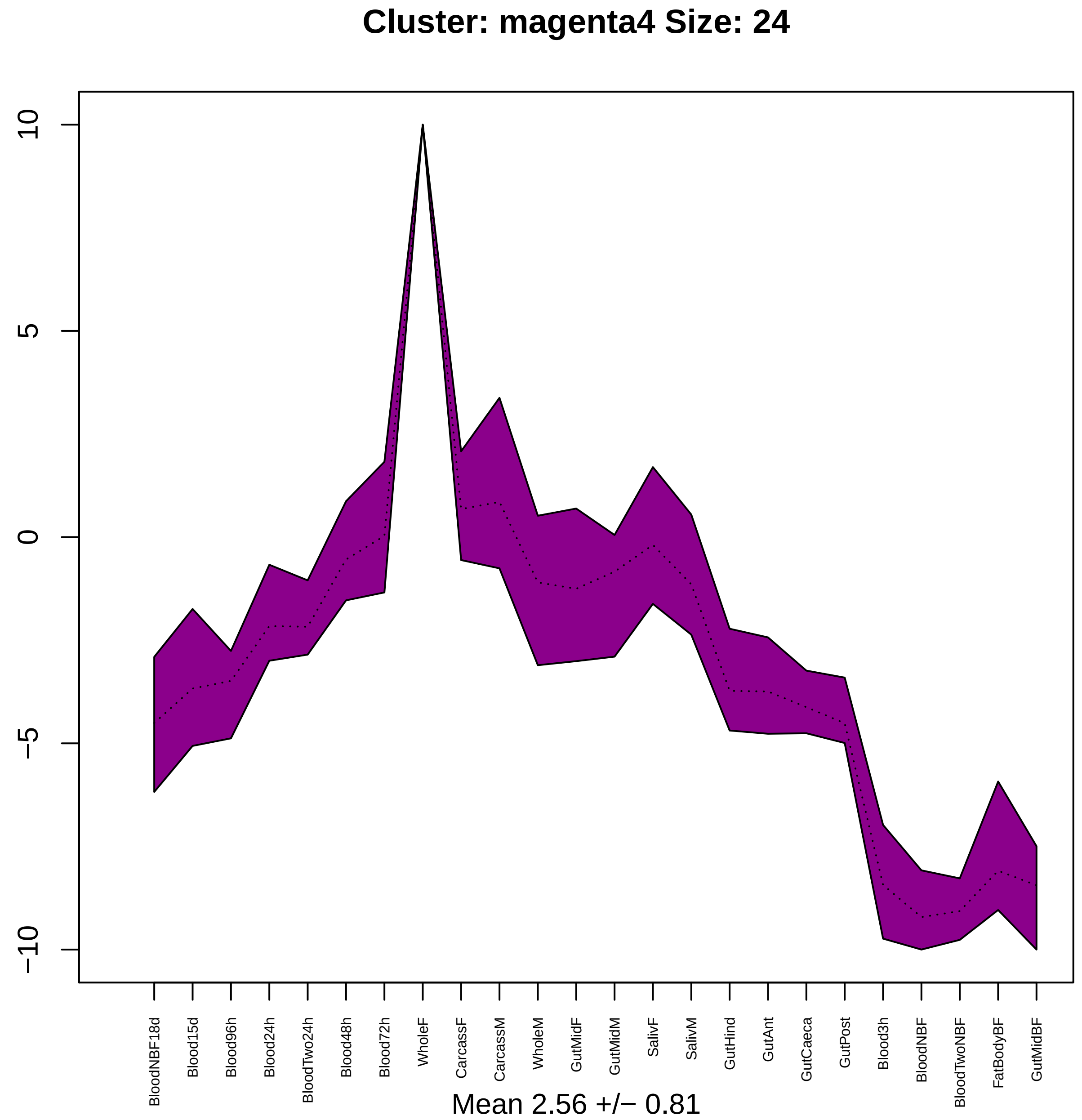
Cluster: magenta4 Size: 24
10
5
0
−5
−10
BloodNBF18d
Blood15d
Blood96h
Blood24h
BloodTwo24h
Blood48h
Blood72h
WholeF
CarcassF
CarcassM
WholeM
GutMidF
GutMidM
SalivF
SalivM
GutHind
GutAnt
GutCaeca
GutPost
Blood3h
BloodNBF
BloodTwoNBF
FatBodyBF
GutMidBF
Mean 2.56 +/− 0.81

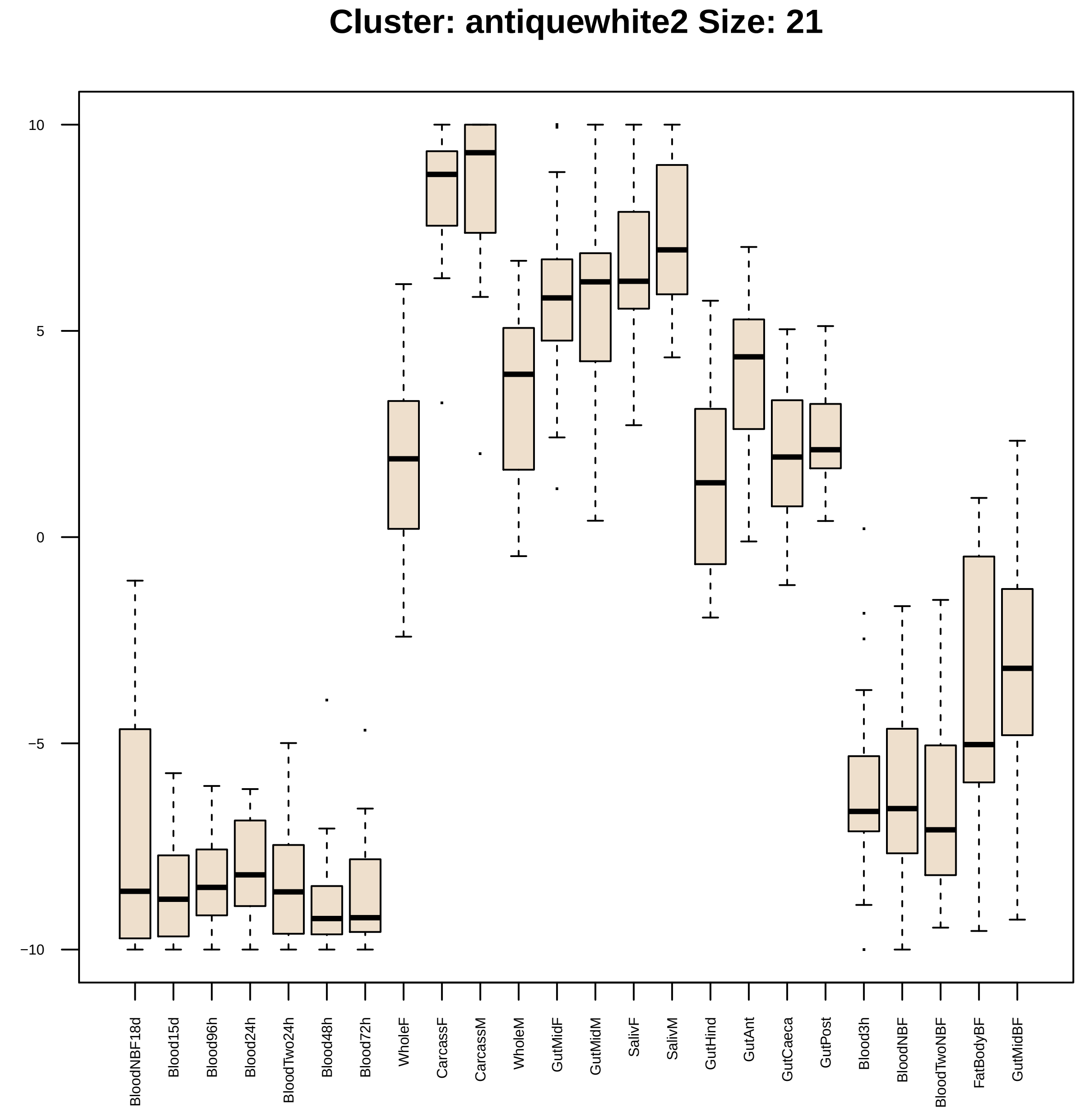
Cluster: antiquewhite2 Size: 21
10
5
0
−5
−10
BloodNBF18d
Blood15d
Blood96h
Blood24h
BloodTwo24h
Blood48h
Blood72h
WholeF
CarcassF
CarcassM
WholeM
GutMidF
GutMidM
SalivF
SalivM
GutHind
GutAnt
GutCaeca
GutPost
Blood3h
BloodNBF
BloodTwoNBF
FatBodyBF
GutMidBF

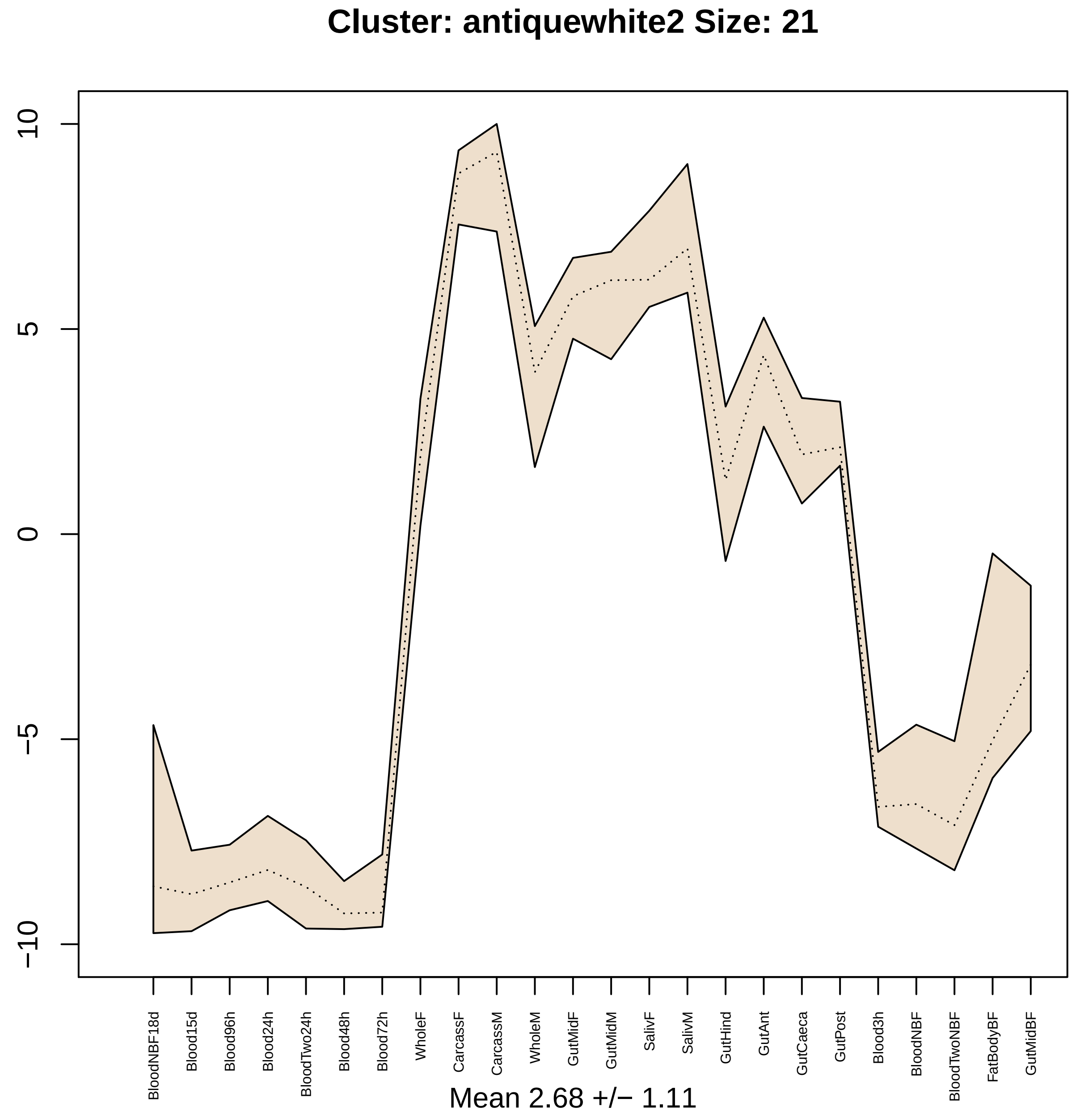

Cluster: antiquewhite2 Size: 21
10
5
0
−5
−10
BloodNBF18d
Blood15d
Blood96h
Blood24h
BloodTwo24h
Blood48h
Blood72h
WholeF
CarcassF
CarcassM
WholeM
GutMidF
GutMidM
SalivF
SalivM
GutHind
GutAnt
GutCaeca
GutPost
Blood3h
BloodNBF
BloodTwoNBF
FatBodyBF
GutMidBF
Mean 2.68 +/− 1.11

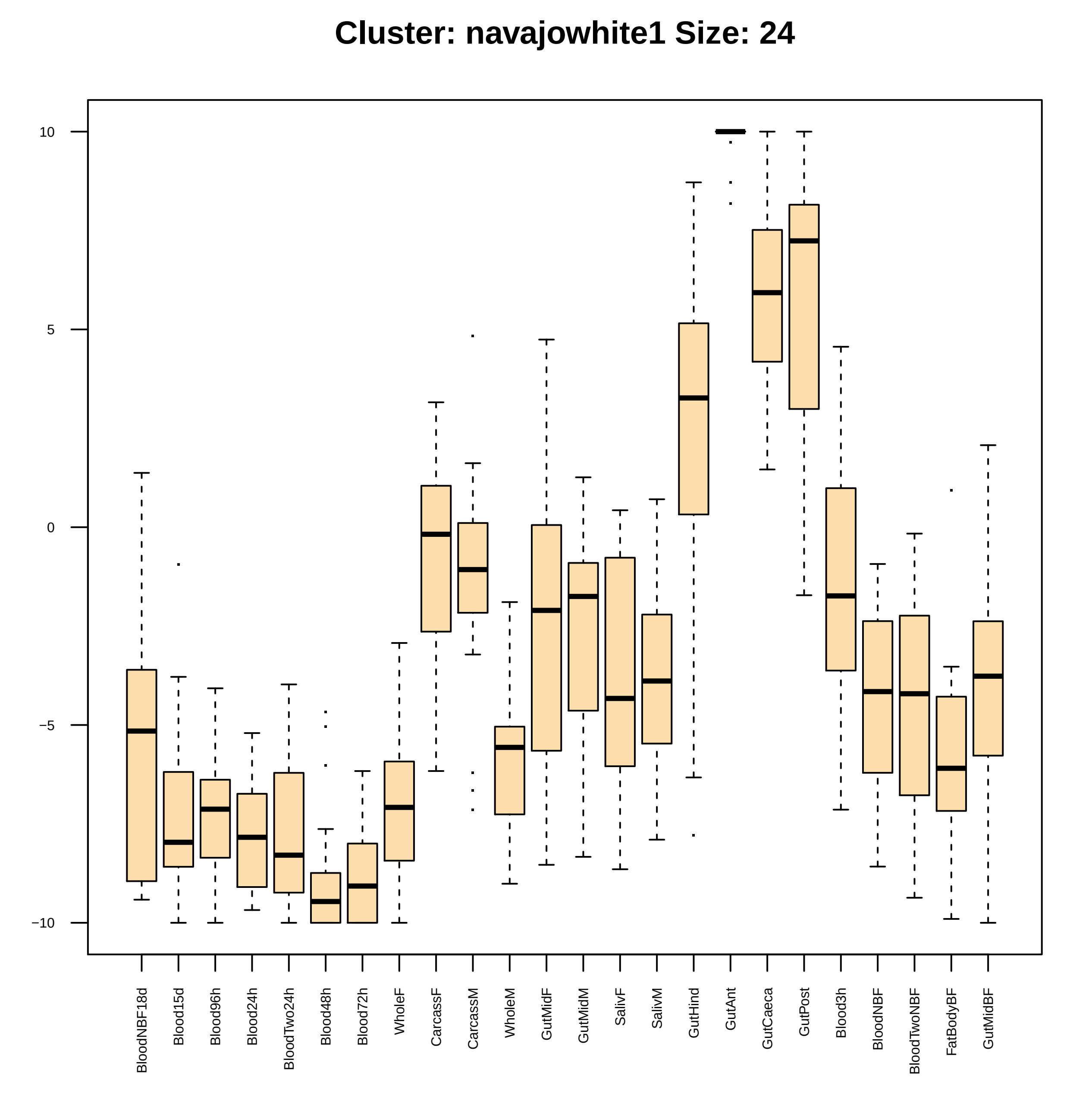

Cluster: navajowhite1 Size: 24
10
5
0
−5
−10
BloodNBF18d
Blood15d
Blood96h
Blood24h
BloodTwo24h
Blood48h
Blood72h
WholeF
CarcassF
CarcassM
WholeM
GutMidF
GutMidM
SalivF
SalivM
GutHind
GutAnt
GutCaeca
GutPost
Blood3h
BloodNBF
BloodTwoNBF
FatBodyBF
GutMidBF

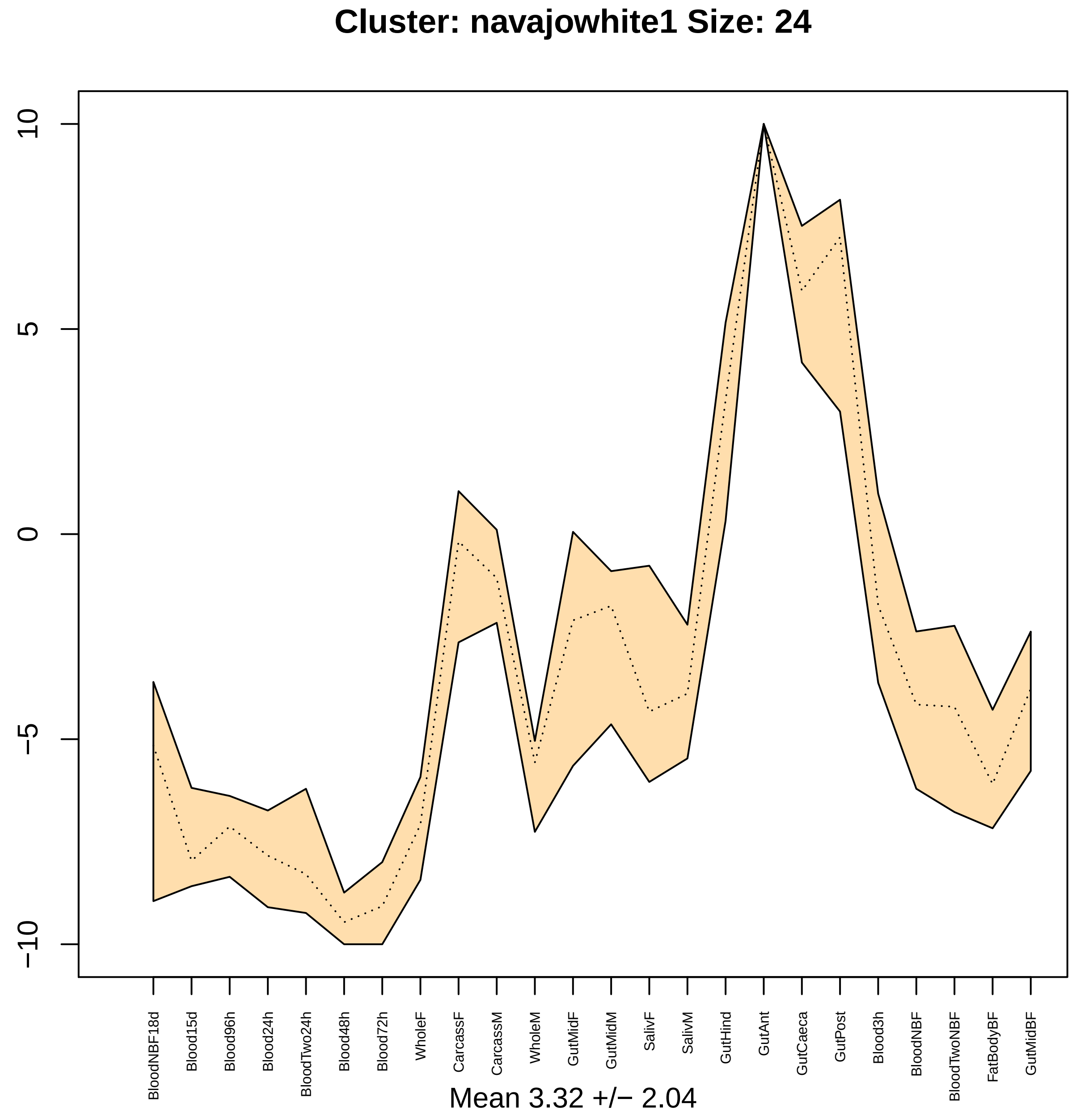

Cluster: navajowhite1 Size: 24
10
5
0
−5
−10
BloodNBF18d
Blood15d
Blood96h
Blood24h
BloodTwo24h
Blood48h
Blood72h
WholeF
CarcassF
CarcassM
WholeM
GutMidF
GutMidM
SalivF
SalivM
GutHind
GutAnt
GutCaeca
GutPost
Blood3h
BloodNBF
BloodTwoNBF
FatBodyBF
GutMidBF
Mean 3.32 +/− 2.04

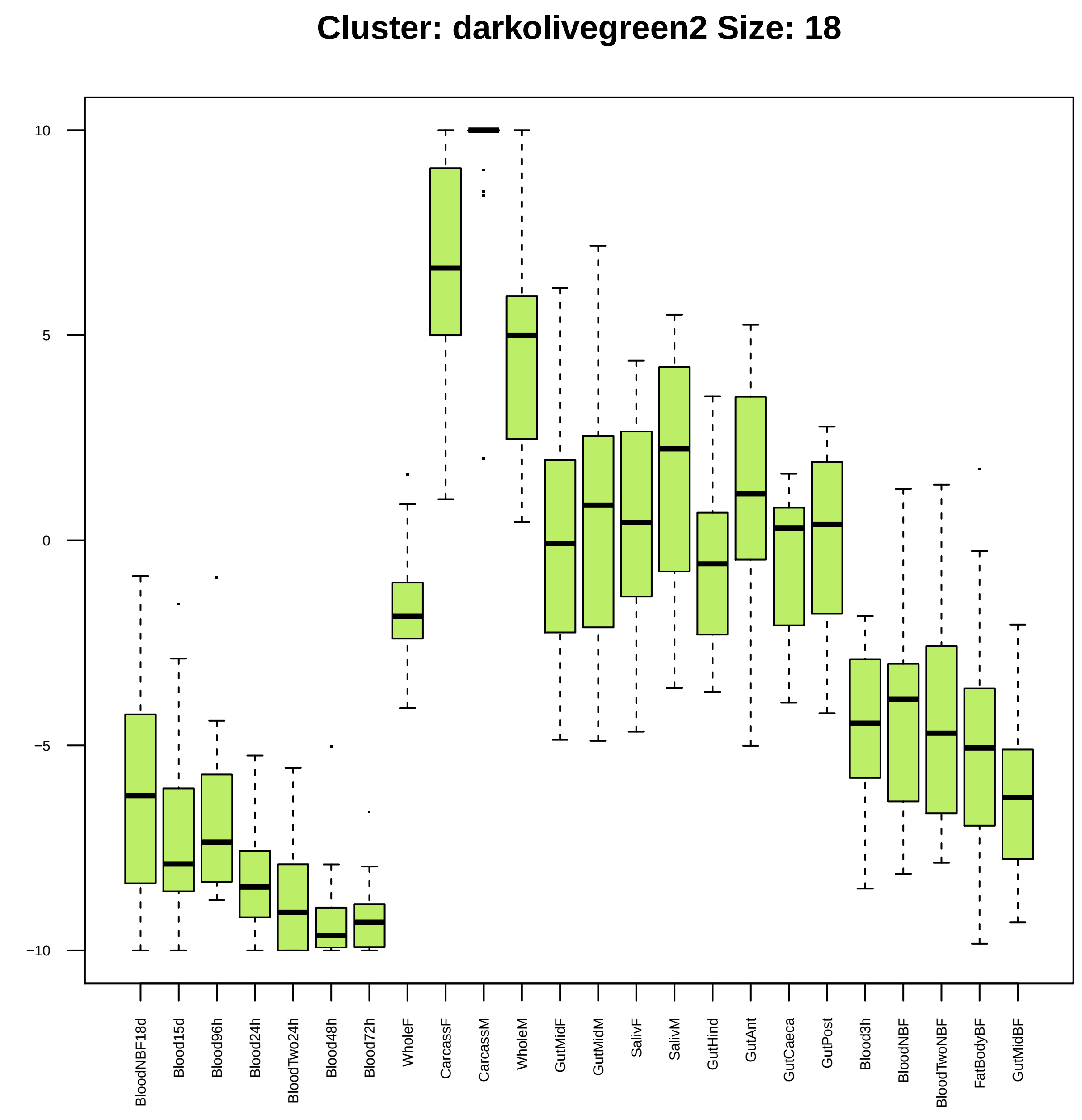

Cluster: darkolivegreen2 Size: 18
10
5
0
−5
−10
BloodNBF18d
Blood15d
Blood96h
Blood24h
BloodTwo24h
Blood48h
Blood72h
WholeF
CarcassF
CarcassM
WholeM
GutMidF
GutMidM
SalivF
SalivM
GutHind
GutAnt
GutCaeca
GutPost
Blood3h
BloodNBF
BloodTwoNBF
FatBodyBF
GutMidBF

| | GO.ID | MFCluster: darkolivegreen2 Size: 18 | Annotated | Significant | Expected | Rank in ClassicF | Weight01F | ClassicF |
|---|---|---|---|---|---|---|---|---|
| 5 | GO:0008238 | exopeptidase activity | 66 | 2 | 0.09 | 1 | 0.051 | 0.0035 |

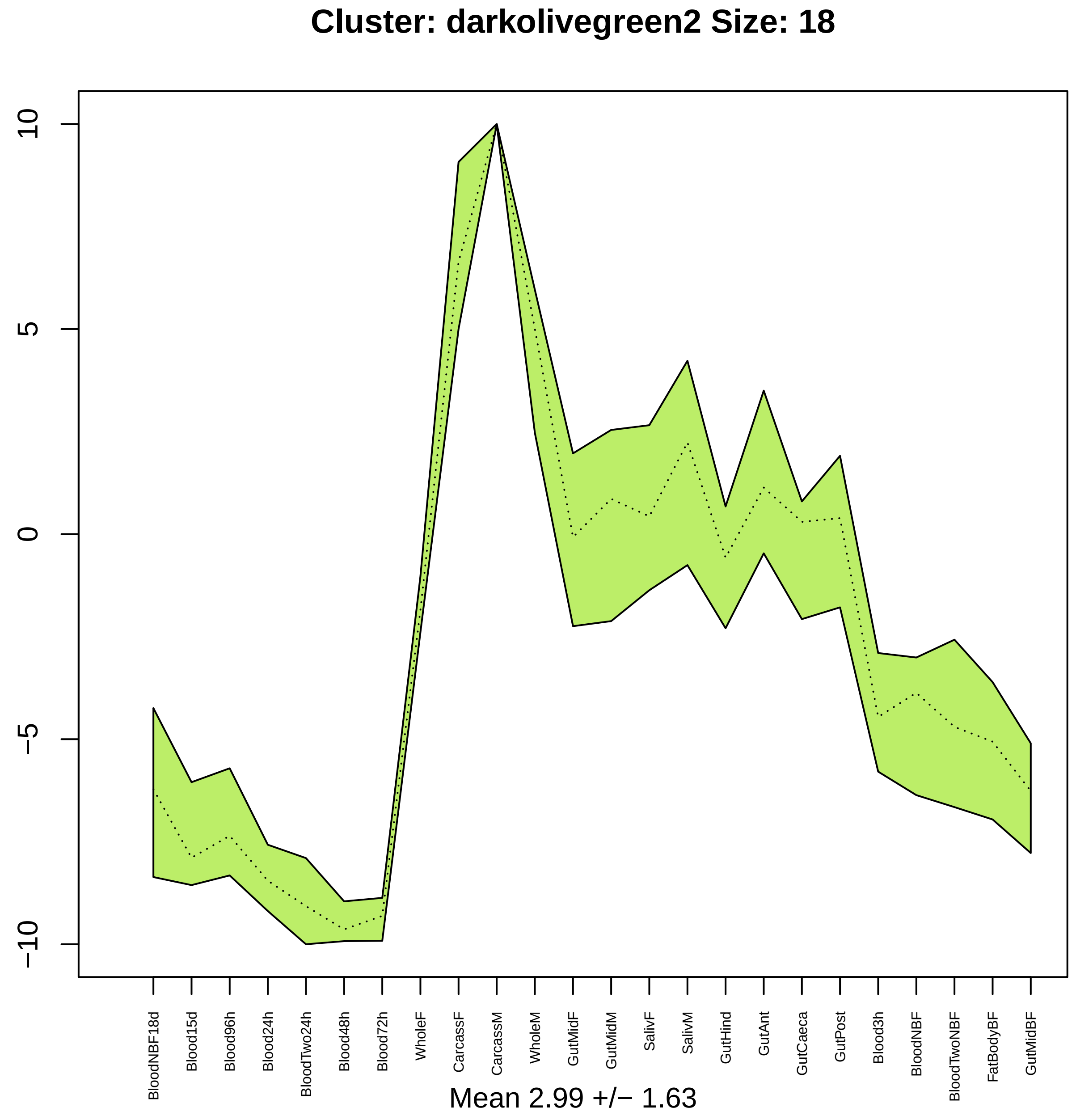

Cluster: darkolivegreen2 Size: 18
10
5
0
−5
−10
BloodNBF18d
Blood15d
Blood96h
Blood24h
BloodTwo24h
Blood48h
Blood72h
WholeF
CarcassF
CarcassM
WholeM
GutMidF
GutMidM
SalivF
SalivM
GutHind
GutAnt
GutCaeca
GutPost
Blood3h
BloodNBF
BloodTwoNBF
FatBodyBF
GutMidBF
Mean 2.99 +/− 1.63

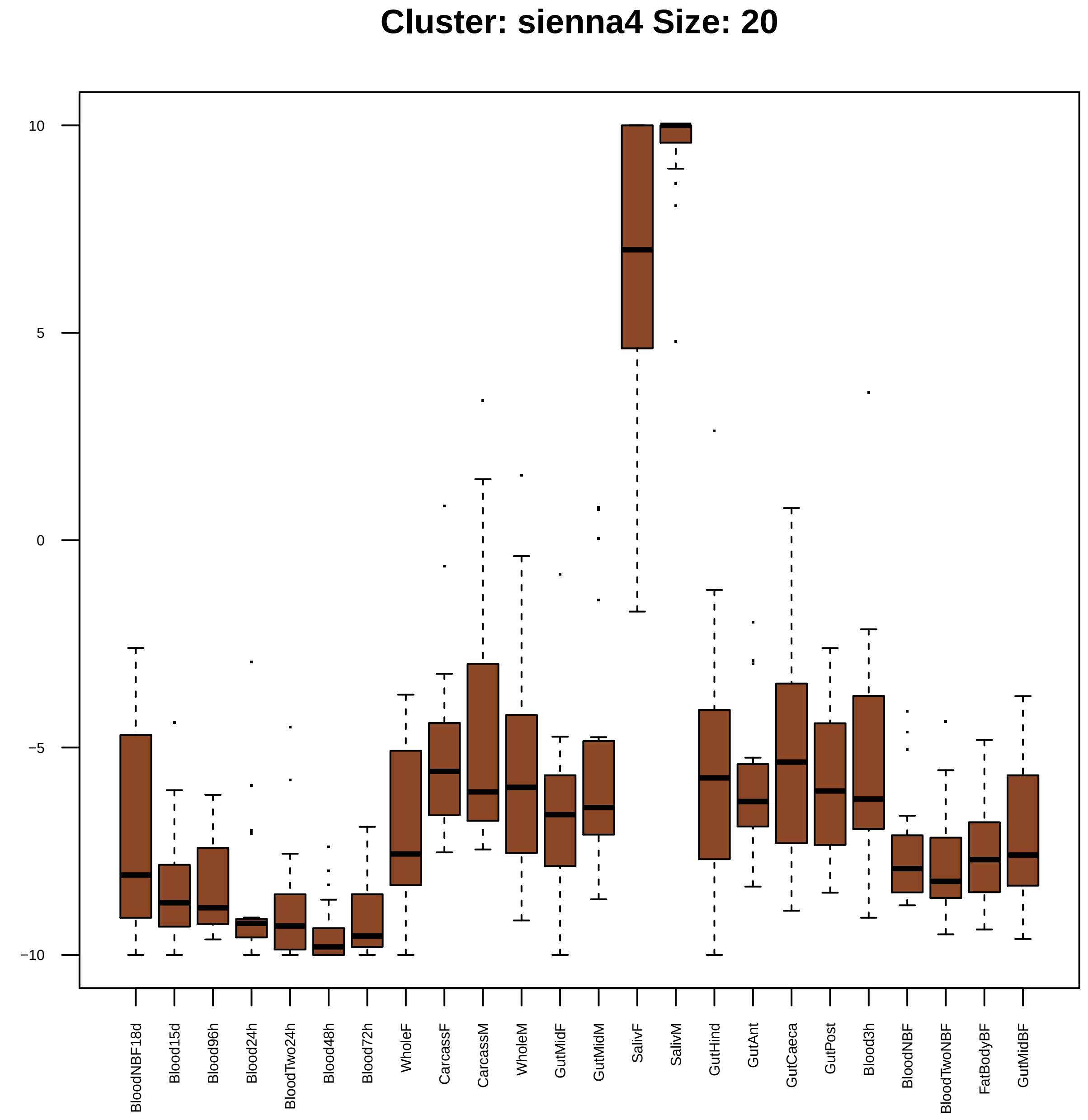

Cluster: sienna4 Size: 20
10
5
0
−5
−10
BloodNBF18d
Blood15d
Blood96h
Blood24h
BloodTwo24h
Blood48h
Blood72h
WholeF
CarcassF
CarcassM
WholeM
GutMidF
GutMidM
SalivF
SalivM
GutHind
GutAnt
GutCaeca
GutPost
Blood3h
BloodNBF
BloodTwoNBF
FatBodyBF
GutMidBF

| | GO.ID | BPCluster: sienna4 Size: 20 | Annotated | Significant | Expected | Rank in ClassicF | Weight01F | ClassicF |
|---|---|---|---|---|---|---|---|---|
| *1* | GO:0006508 | proteolysis | 594 | 6 | 1.3 | 1 | 0.001 | 0.001 |

| | GO.ID | MFCluster: sienna4 Size: 20 | Annotated | Significant | Expected | Rank in ClassicF | Weight01F | ClassicF |
|---|---|---|---|---|---|---|---|---|
| *17* | GO:0070011 | peptidase activity, acting on L−amino ac... | 470 | 6 | 0.91 | 5 | 1.000 | 0.00014 |
| *21* | GO:0016787 | hydrolase activity | 1445 | 8 | 2.81 | 7 | 1.000 | 0.00241 |
| *24* | GO:0008233 | peptidase activity | 500 | 6 | 0.97 | 6 | 1.000 | 0.00020 |

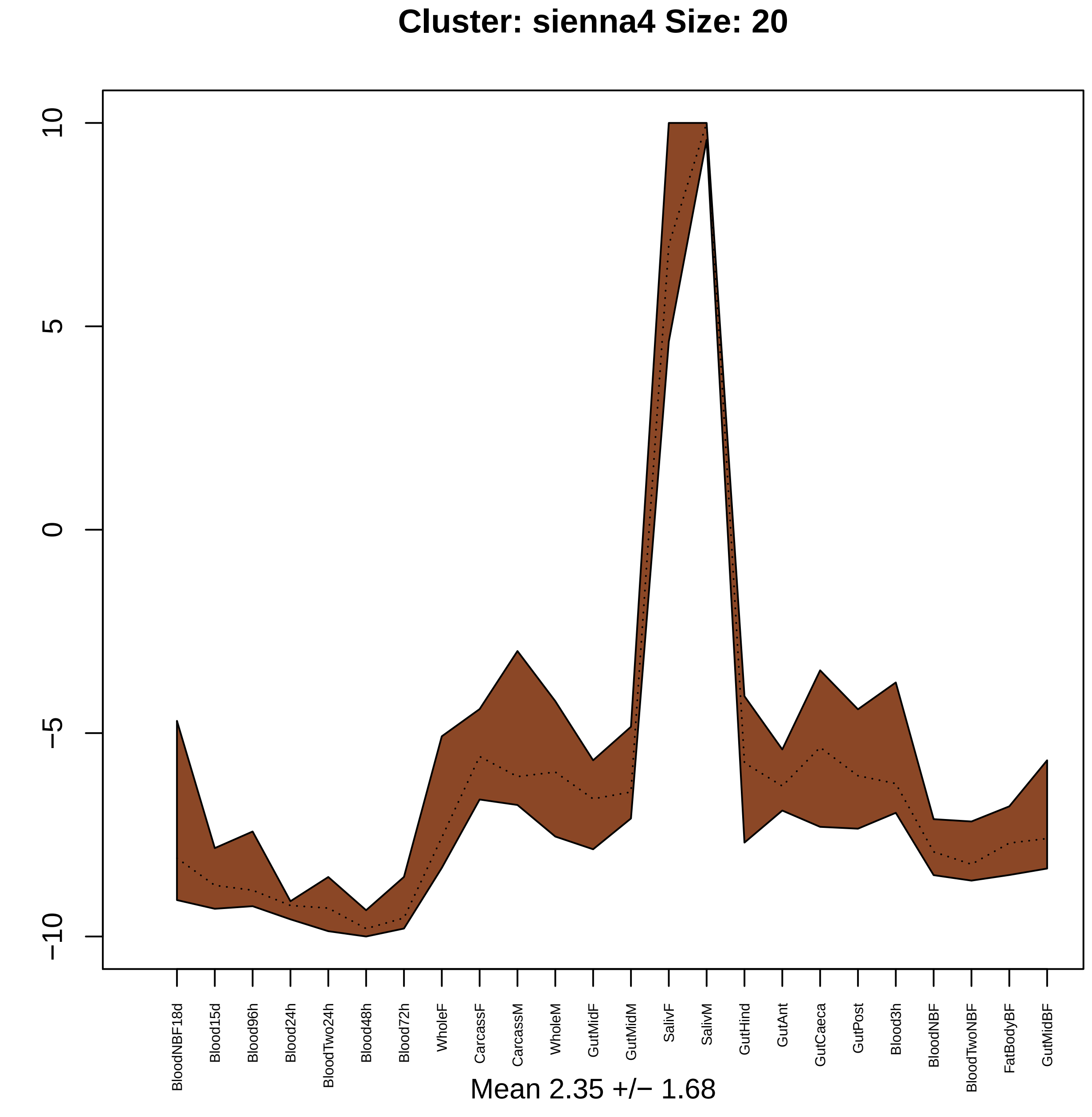
Cluster: sienna4 Size: 20
10
5
0
−5
−10
BloodNBF18d
Blood15d
Blood96h
Blood24h
BloodTwo24h
Blood48h
Blood72h
WholeF
CarcassF
CarcassM
WholeM
GutMidF
GutMidM
SalivF
SalivM
GutHind
GutAnt
GutCaeca
GutPost
Blood3h
BloodNBF
BloodTwoNBF
FatBodyBF
GutMidBF
Mean 2.35 +/− 1.68

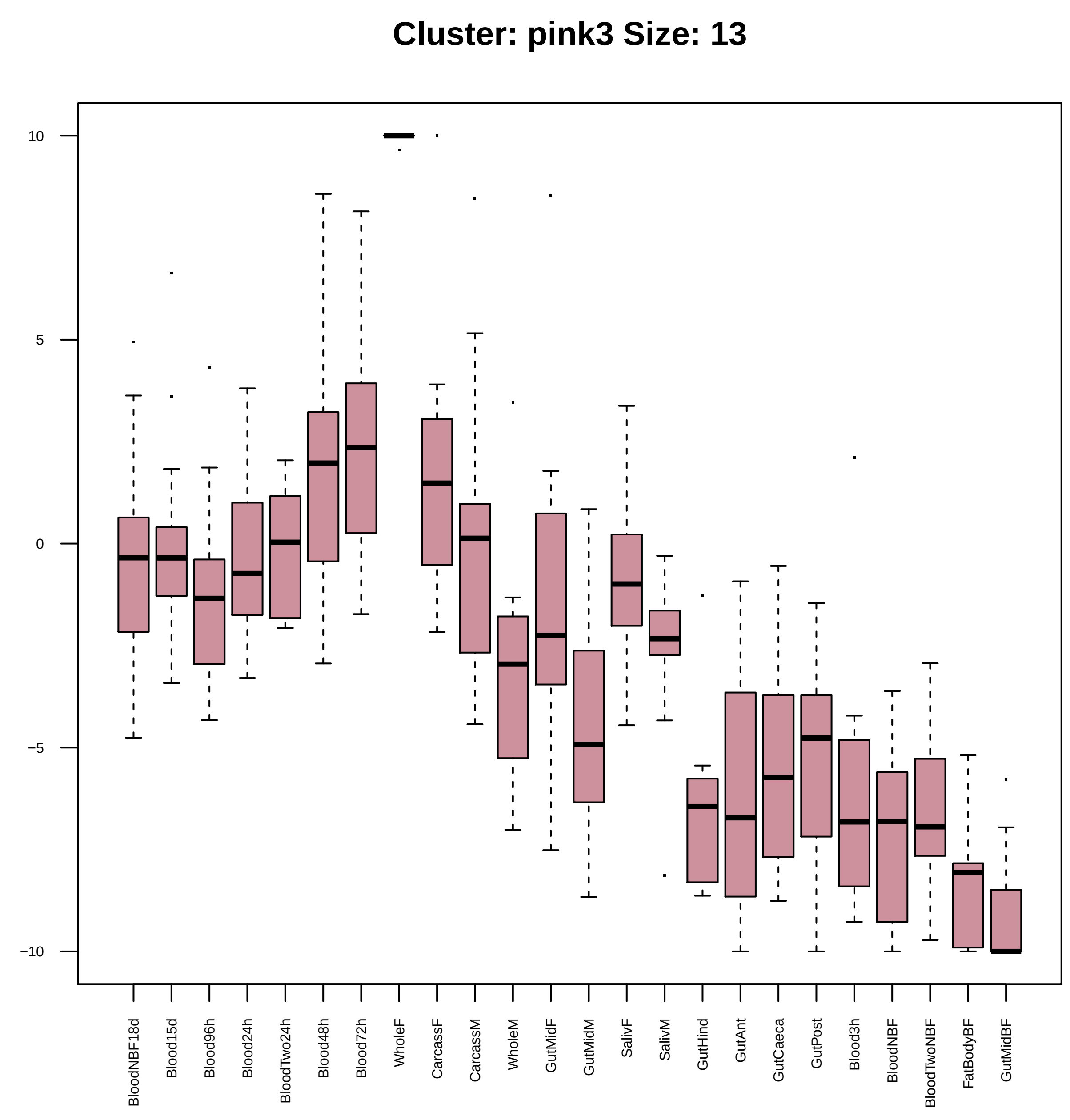

Cluster: pink3 Size: 13
10
5
0
−5
−10
BloodNBF18d
Blood15d
Blood96h
Blood24h
BloodTwo24h
Blood48h
Blood72h
WholeF
CarcassF
CarcassM
WholeM
GutMidF
GutMidM
SalivF
SalivM
GutHind
GutAnt
GutCaeca
GutPost
Blood3h
BloodNBF
BloodTwoNBF
FatBodyBF
GutMidBF

| | GO.ID | BPCluster: pink3 Size: 13 | Annotated | Significant | Expected | Rank in ClassicF | Weight01F | ClassicF |
|---|---|---|---|---|---|---|---|---|
| *1* | GO:0000398 | mRNA splicing, via spliceosome | 134 | 3 | 0.21 | 3 | 0.0078 | 0.00098 |
| *8* | GO:0008033 | tRNA processing | 39 | 2 | 0.06 | 6 | 0.0266 | 0.00159 |
| *26* | GO:0006399 | tRNA metabolic process | 82 | 2 | 0.13 | 11 | 1.0000 | 0.00687 |

| | GO.ID | MFCluster: pink3 Size: 13 | Annotated | Significant | Expected | Rank in ClassicF | Weight01F | ClassicF |
|---|---|---|---|---|---|---|---|---|
| *1* | GO:0140101 | catalytic activity, acting on a tRNA | 66 | 2 | 0.09 | 1 | 0.0035 | 0.0035 |

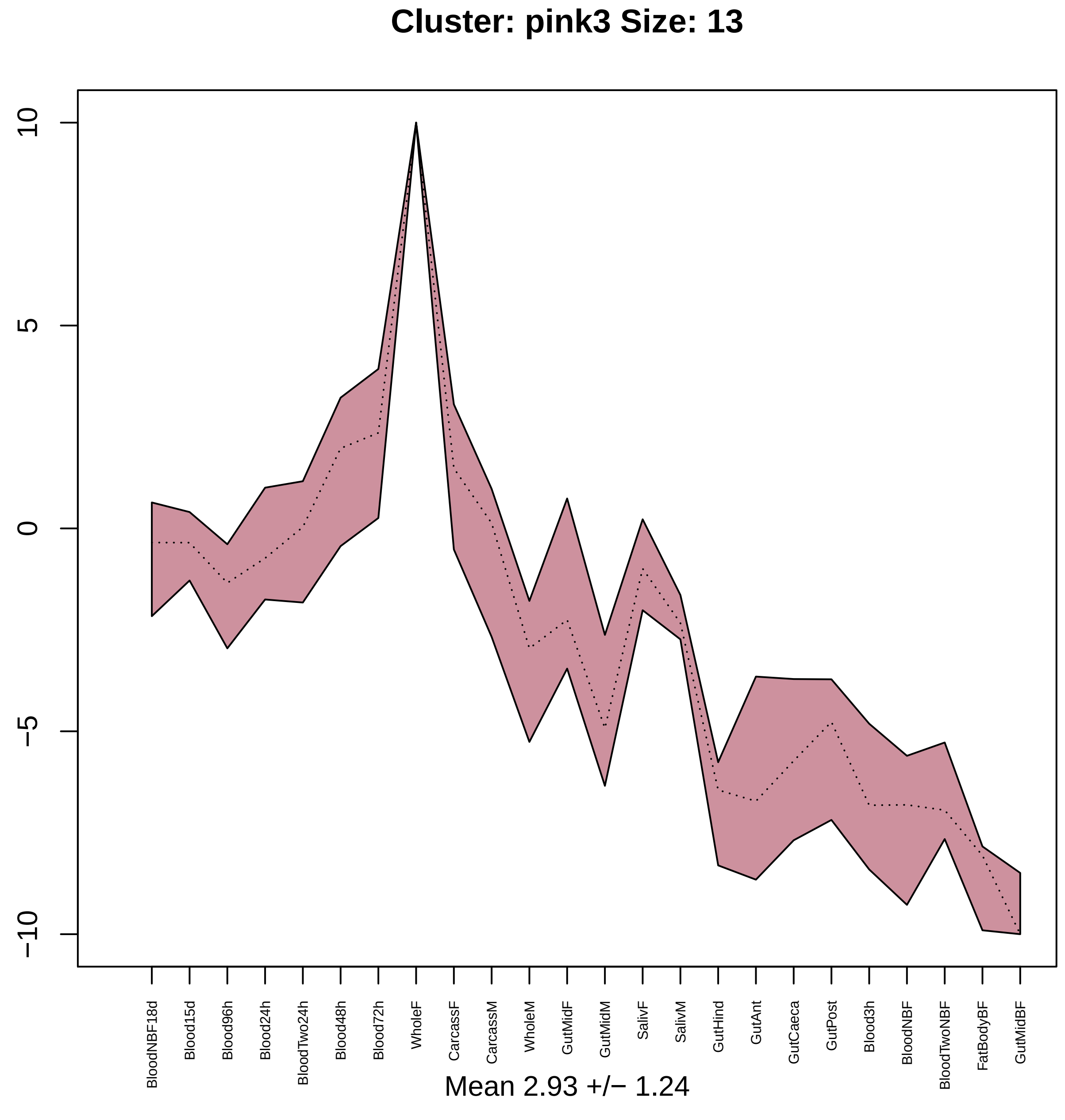

Cluster: pink3 Size: 13
10
5
0
−5
−10
BloodNBF18d
Blood15d
Blood96h
Blood24h
BloodTwo24h
Blood48h
Blood72h
WholeF
CarcassF
CarcassM
WholeM
GutMidF
GutMidM
SalivF
SalivM
GutHind
GutAnt
GutCaeca
GutPost
Blood3h
BloodNBF
BloodTwoNBF
FatBodyBF
GutMidBF
Mean 2.93 +/− 1.24

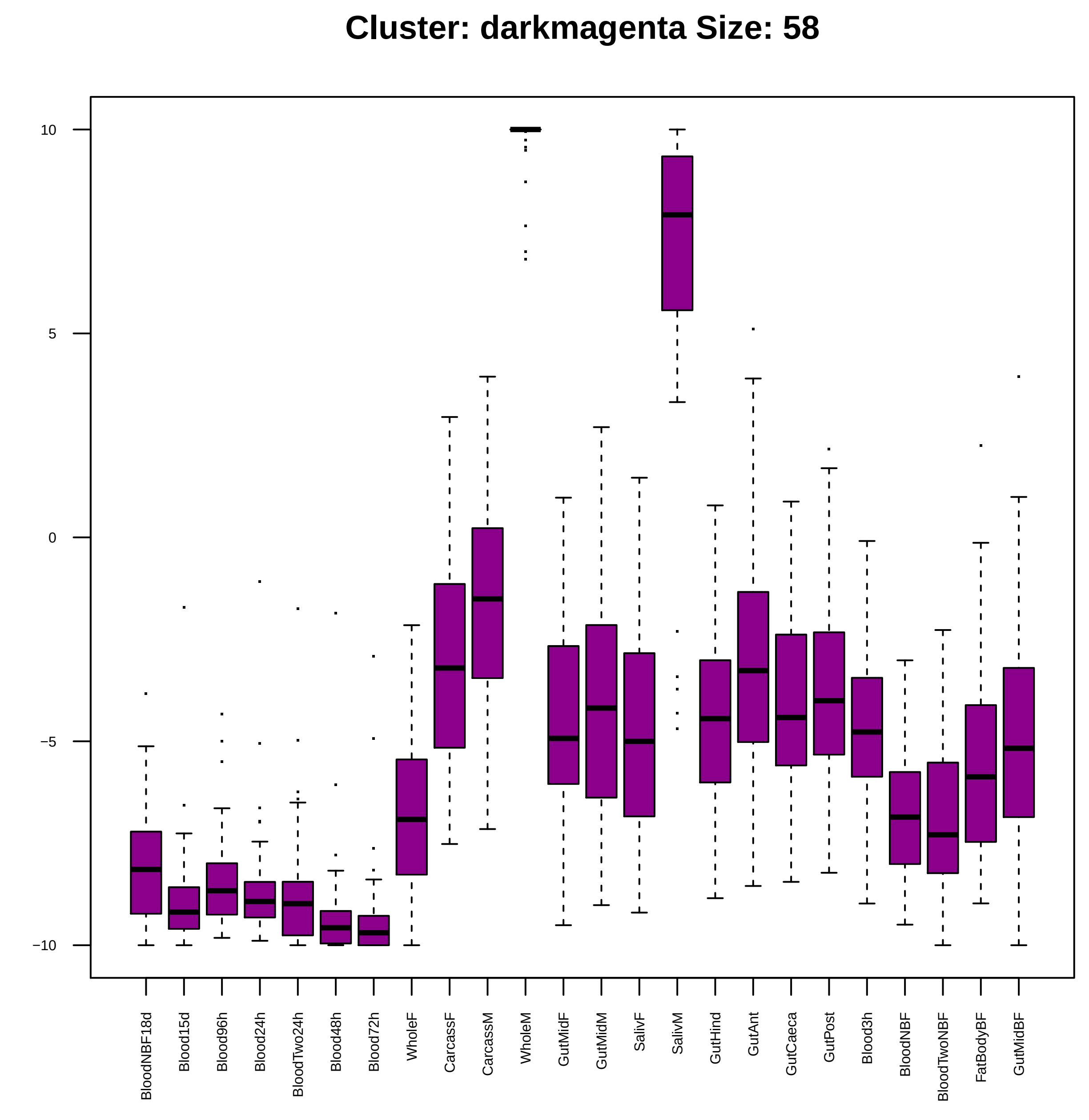
Cluster: darkmagenta Size: 58
10
5
0
−5
−10
BloodNBF18d
Blood15d
Blood96h
Blood24h
BloodTwo24h
Blood48h
Blood72h
WholeF
CarcassF
CarcassM
WholeM
GutMidF
GutMidM
SalivF
SalivM
GutHind
GutAnt
GutCaeca
GutPost
Blood3h
BloodNBF
BloodTwoNBF
FatBodyBF
GutMidBF

| | GO.ID | BPCluster: darkmagenta Size: 58 | Annotated | Significant | Expected | Rank in ClassicF | Weight01F | ClassicF |
|---|---|---|---|---|---|---|---|---|
| *1* | GO:0007017 | microtubule−based process | 282 | 7 | 0.93 | 1 | 0.0011 | 2.1e−05 |
| *2* | GO:0007018 | microtubule−based movement | 65 | 3 | 0.21 | 2 | 0.0012 | 0.0012 |
| *3* | GO:0051258 | protein polymerization | 39 | 2 | 0.13 | 3 | 0.0071 | 0.0071 |

| | GO.ID | MFCluster: darkmagenta Size: 58 | Annotated | Significant | Expected | Rank in ClassicF | Weight01F | ClassicF |
|---|---|---|---|---|---|---|---|---|
| 1 | GO:0030145 | manganese ion binding | 12 | 2 | 0.04 | 2 | 0.00069 | 0.00069 |
| 2 | GO:0005200 | structural constituent of cytoskeleton | 19 | 2 | 0.06 | 4 | 0.00175 | 0.00175 |
| 3 | GO:0004177 | aminopeptidase activity | 25 | 2 | 0.08 | 5 | 0.00304 | 0.00304 |
| 4 | GO:0008235 | metalloexopeptidase activity | 29 | 2 | 0.10 | 6 | 0.00408 | 0.00408 |
| 5 | GO:0016887 | ATPase activity | 233 | 3 | 0.78 | 31 | 0.00743 | 0.04091 |
| 6 | GO:0003777 | microtubule motor activity | 40 | 2 | 0.13 | 9 | 0.00767 | 0.00767 |
| 12 | GO:0003774 | motor activity | 60 | 3 | 0.20 | 3 | 0.05970 | 0.00098 |

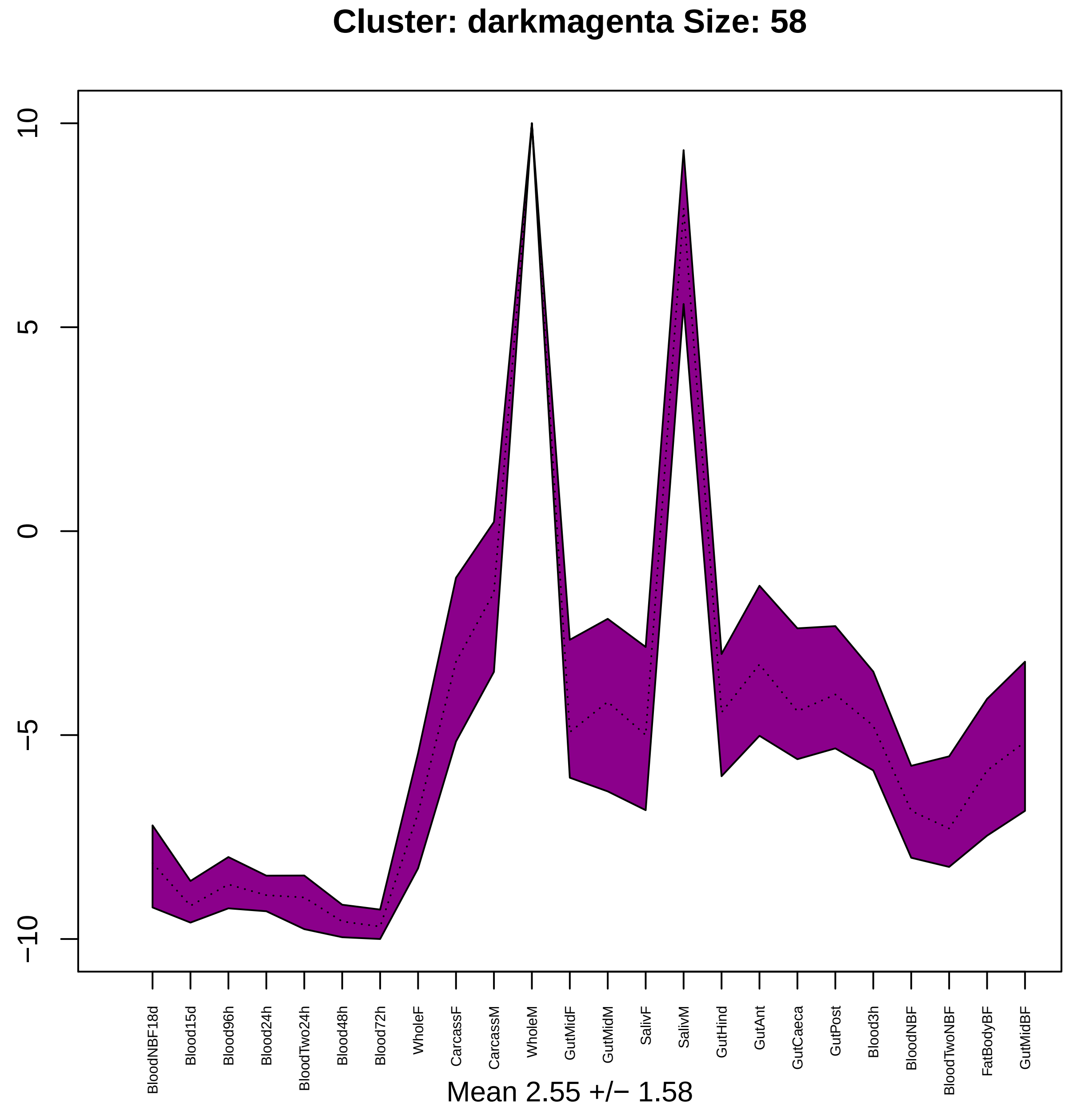

Cluster: darkmagenta Size: 58
10
5
0
−5
−10
BloodNBF18d
Blood15d
Blood96h
Blood24h
BloodTwo24h
Blood48h
Blood72h
WholeF
CarcassF
CarcassM
WholeM
GutMidF
GutMidM
SalivF
SalivM
GutHind
GutAnt
GutCaeca
GutPost
Blood3h
BloodNBF
BloodTwoNBF
FatBodyBF
GutMidBF
Mean 2.55 +/− 1.58

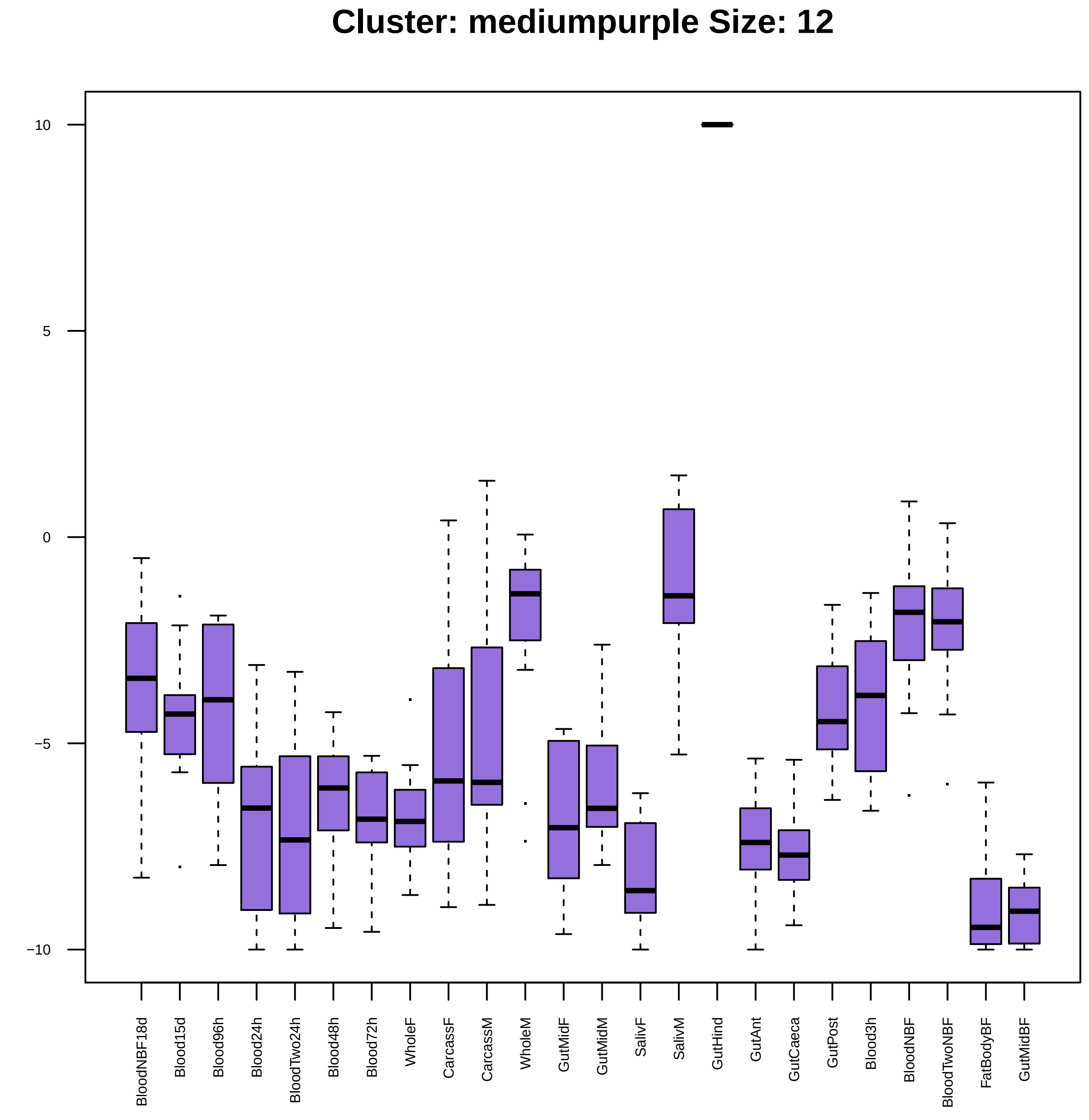
Cluster: mediumpurple Size: 12
10
5
0
−5
−10
BloodNBF18d
Blood15d
Blood96h
Blood24h
BloodTwo24h
Blood48h
Blood72h
WholeF
CarcassF
CarcassM
WholeM
GutMidF
GutMidM
SalivF
SalivM
GutHind
GutAnt
GutCaeca
GutPost
Blood3h
BloodNBF
BloodTwoNBF
FatBodyBF
GutMidBF

| | GO.ID | MFCluster: mediumpurple Size: 12 | Annotated | Significant | Expected | Rank in ClassicF | Weight01F | ClassicF |
|---|---|---|---|---|---|---|---|---|
| *2* | GO:0022857 | transmembrane transporter activity | 442 | 4 | 0.61 | 5 | 0.0031 | 0.00218 |
| *5* | GO:0015370 | solute:sodium symporter activity | 31 | 2 | 0.04 | 2 | 0.0211 | 0.00079 |
| *17* | GO:0015291 | secondary active transmembrane transport... | 60 | 2 | 0.08 | 6 | 1.0000 | 0.00294 |
| *20* | GO:0015293 | symporter activity | 37 | 2 | 0.05 | 3 | 1.0000 | 0.00112 |
| *21* | GO:0015294 | solute:cation symporter activity | 37 | 2 | 0.05 | 4 | 1.0000 | 0.00112 |

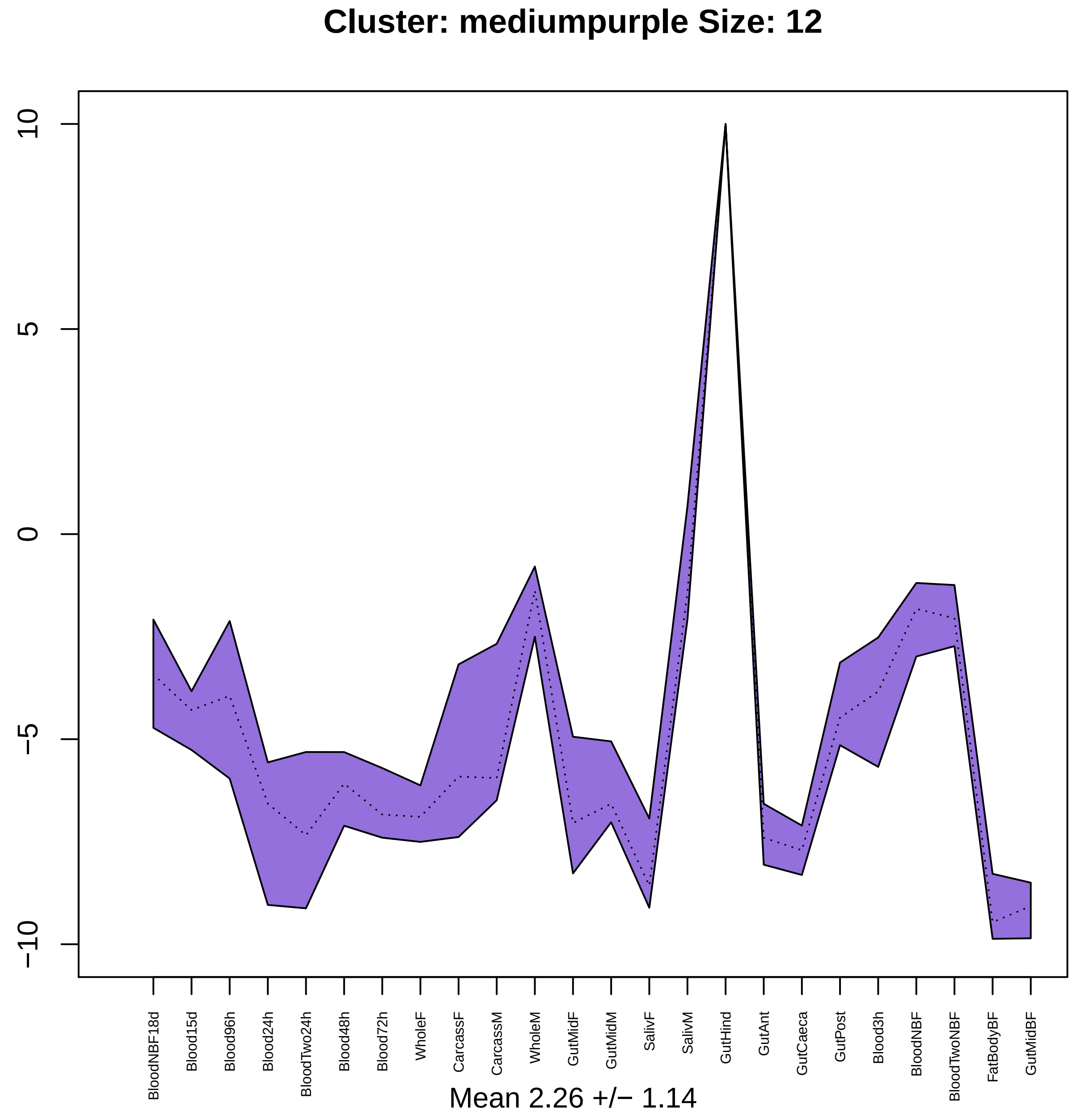

Cluster: mediumpurple Size: 12
10
5
0
−5
−10
BloodNBF18d
Blood15d
Blood96h
Blood24h
BloodTwo24h
Blood48h
Blood72h
WholeF
CarcassF
CarcassM
WholeM
GutMidF
GutMidM
SalivF
SalivM
GutHind
GutAnt
GutCaeca
GutPost
Blood3h
BloodNBF
BloodTwoNBF
FatBodyBF
GutMidBF
Mean 2.26 +/− 1.14

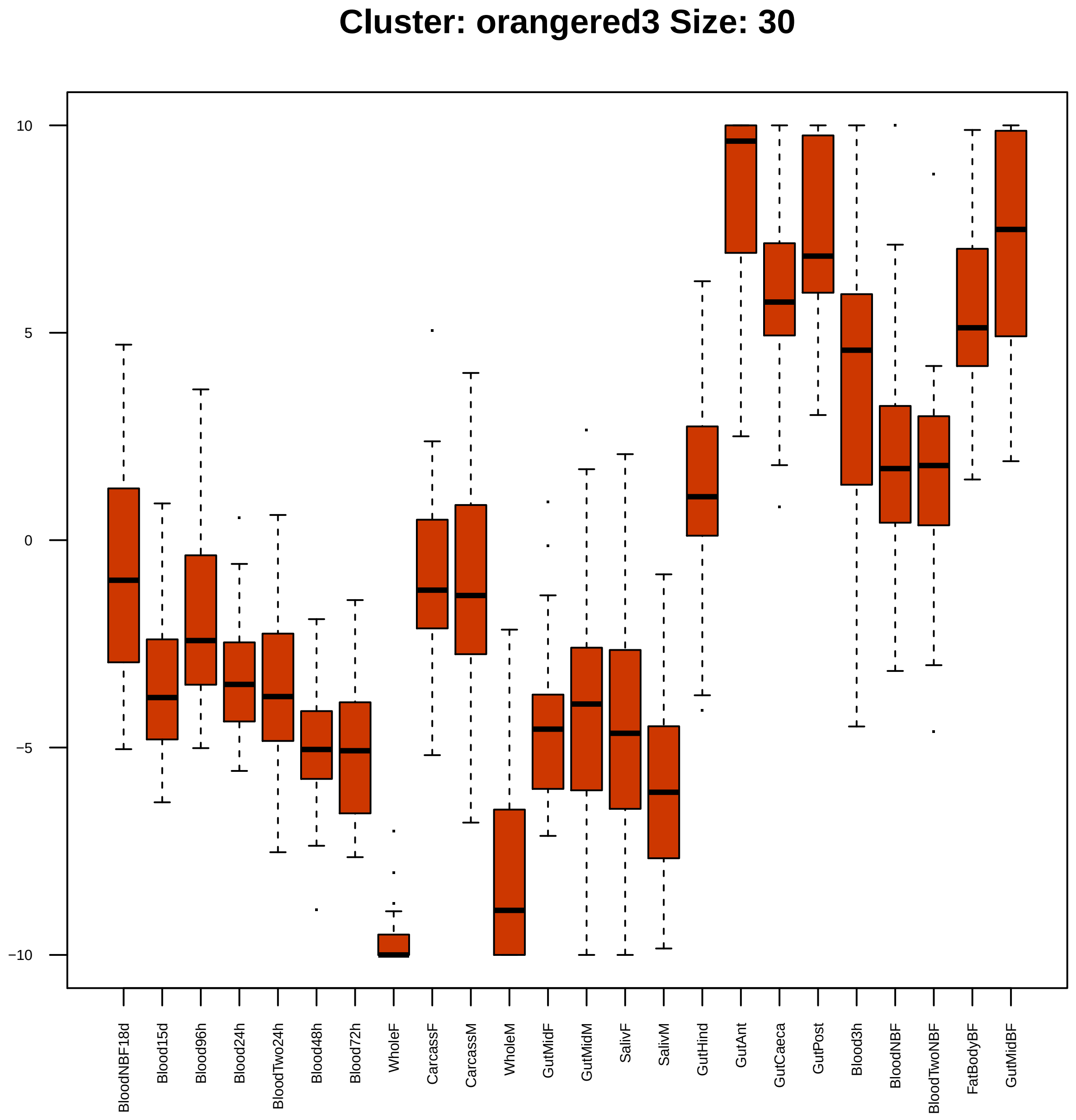
Cluster: orangered3 Size: 30
10
5
0
−5
−10
BloodNBF18d
Blood15d
Blood96h
Blood24h
BloodTwo24h
Blood48h
Blood72h
WholeF
CarcassF
CarcassM
WholeM
GutMidF
GutMidM
SalivF
SalivM
GutHind
GutAnt
GutCaeca
GutPost
Blood3h
BloodNBF
BloodTwoNBF
FatBodyBF
GutMidBF

| | GO.ID | BPCluster: orangered3 Size: 30 | Annotated | Significant | Expected | Rank in ClassicF | Weight01F | ClassicF |
|---|---|---|---|---|---|---|---|---|
| *1* | GO:0007469 | antennal development | 10 | 2 | 0.03 | 3 | 0.00037 | 0.00037 |
| *2* | GO:0009954 | proximal/distal pattern formation | 11 | 2 | 0.03 | 5 | 0.00046 | 0.00046 |
| *3* | GO:0007455 | eye−antennal disc morphogenesis | 17 | 2 | 0.05 | 8 | 0.00111 | 0.00111 |
| *4* | GO:0001708 | cell fate specification | 38 | 3 | 0.11 | 2 | 0.00169 | 0.00018 |
| *5* | GO:0007427 | epithelial cell migration, open tracheal... | 27 | 2 | 0.08 | 21 | 0.00282 | 0.00282 |
| *6* | GO:0008586 | imaginal disc−derived wing vein morphoge... | 28 | 2 | 0.08 | 22 | 0.00304 | 0.00304 |
| *7* | GO:0007548 | sex differentiation | 40 | 2 | 0.12 | 41 | 0.00849 | 0.00613 |
| *8* | GO:0007447 | imaginal disc pattern formation | 47 | 2 | 0.14 | 51 | 0.01413 | 0.00839 |
| *10* | GO:0035218 | leg disc development | 43 | 2 | 0.13 | 45 | 0.01971 | 0.00706 |
| *12* | GO:0045466 | R7 cell differentiation | 29 | 2 | 0.09 | 23 | 0.02521 | 0.00326 |
| *22* | GO:0045595 | regulation of cell differentiation | 112 | 3 | 0.33 | 31 | 0.03467 | 0.00416 |

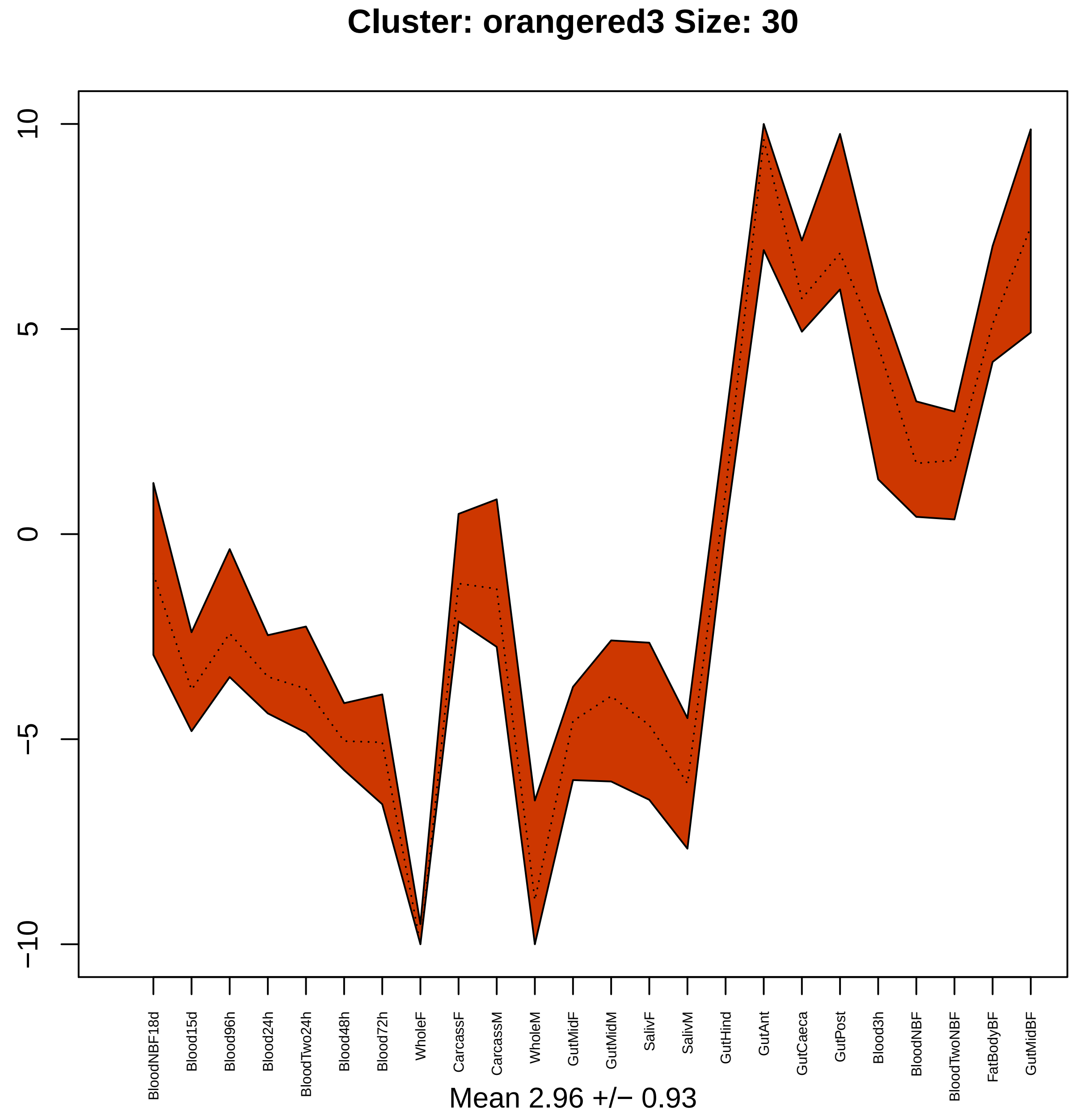

Cluster: orangered3 Size: 30
10
5
0
−5
−10
BloodNBF18d
Blood15d
Blood96h
Blood24h
BloodTwo24h
Blood48h
Blood72h
WholeF
CarcassF
CarcassM
WholeM
GutMidF
GutMidM
SalivF
SalivM
GutHind
GutAnt
GutCaeca
GutPost
Blood3h
BloodNBF
BloodTwoNBF
FatBodyBF
GutMidBF
Mean 2.96 +/− 0.93

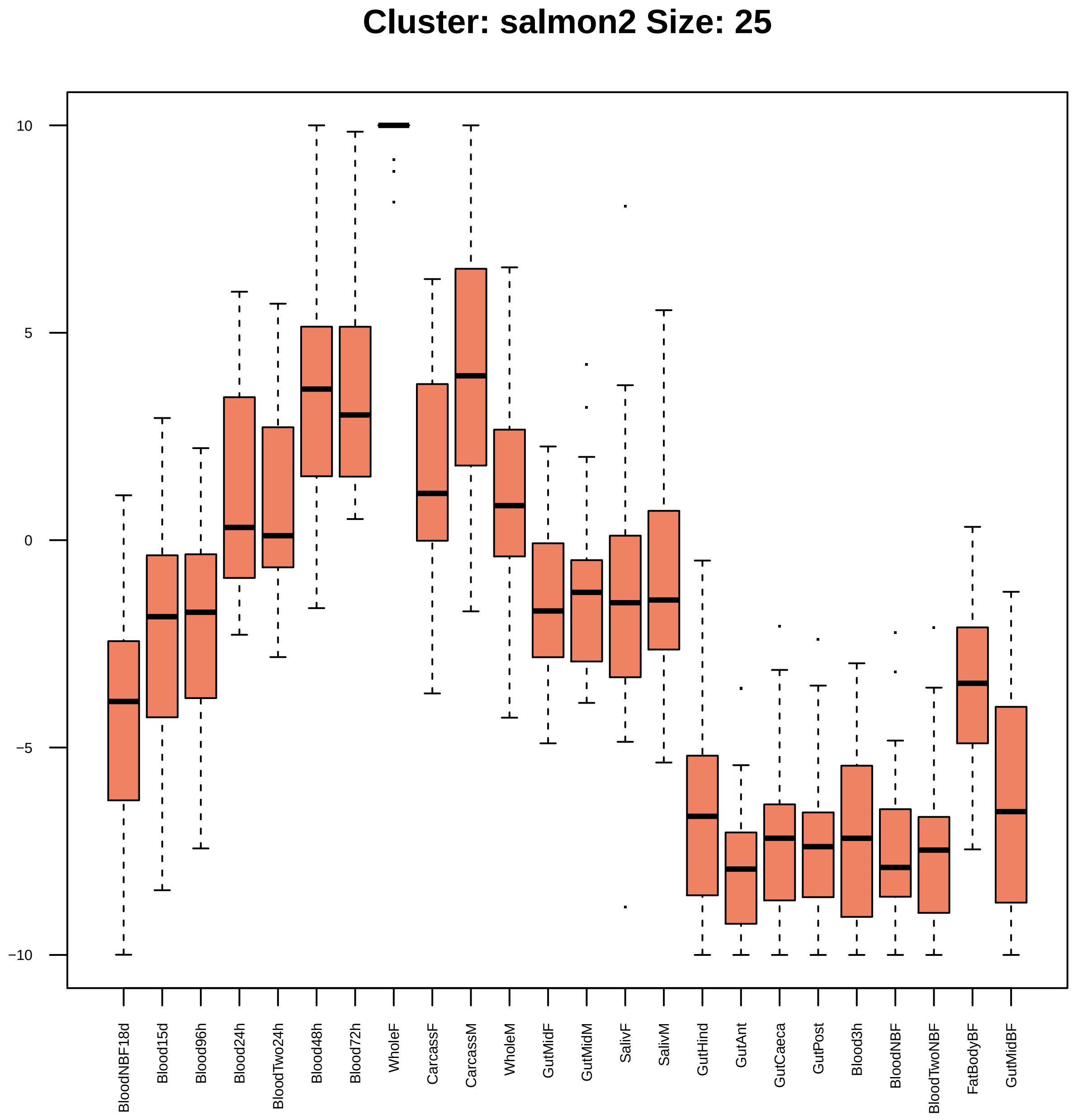

Cluster: salmon2 Size: 25
10
5
0
−5
−10
BloodNBF18d
Blood15d
Blood96h
Blood24h
BloodTwo24h
Blood48h
Blood72h
WholeF
CarcassF
CarcassM
WholeM
GutMidF
GutMidM
SalivF
SalivM
GutHind
GutAnt
GutCaeca
GutPost
Blood3h
BloodNBF
BloodTwoNBF
FatBodyBF
GutMidBF

| | GO.ID | BPCluster: salmon2 Size: 25 | Annotated | Significant | Expected | Rank in ClassicF | Weight01F | ClassicF |
|---|---|---|---|---|---|---|---|---|
| *1* | GO:0006261 | DNA−dependent DNA replication | 52 | 2 | 0.09 | 2 | 0.0034 | 0.0034 |
| *2* | GO:0006281 | DNA repair | 89 | 2 | 0.15 | 5 | 0.0098 | 0.0098 |
| *12* | GO:0007399 | nervous system development | 801 | 5 | 1.38 | 4 | 0.0260 | 0.0074 |

| | GO.ID | MFCluster: salmon2 Size: 25 | Annotated | Significant | Expected | Rank in ClassicF | Weight01F | ClassicF |
|---|---|---|---|---|---|---|---|---|
| *1* | GO:0008270 | zinc ion binding | 522 | 7 | 1.45 | 3 | 0.00034 | 0.00034 |
| *2* | GO:0003677 | DNA binding | 523 | 6 | 1.45 | 7 | 0.00228 | 0.00228 |
| *7* | GO:0046872 | metal ion binding | 1255 | 10 | 3.48 | 4 | 0.04363 | 0.00084 |
| *28* | GO:0046914 | transition metal ion binding | 692 | 7 | 1.92 | 6 | 1.00000 | 0.00185 |

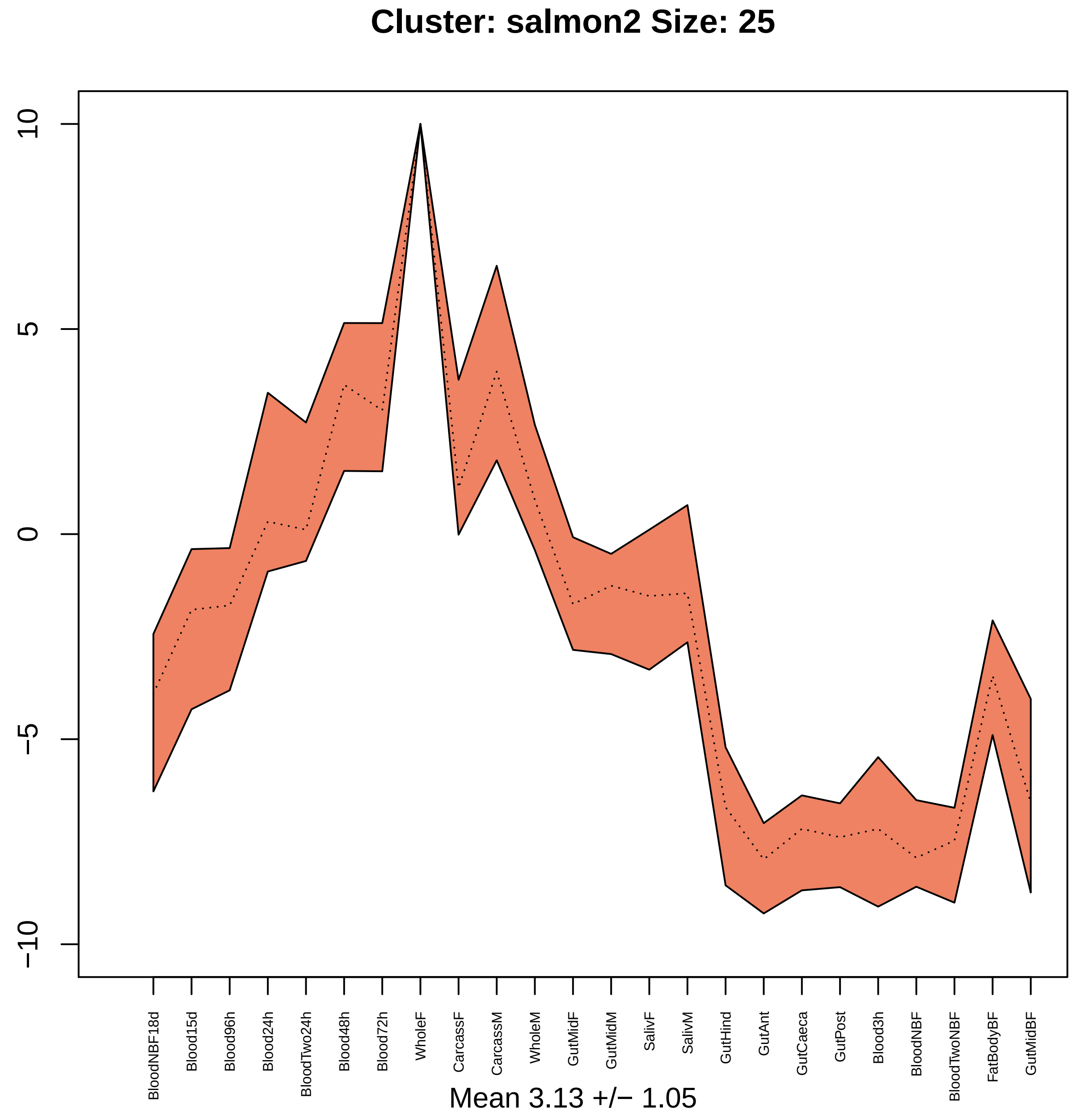

Cluster: salmon2 Size: 25
10
5
0
−5
−10
BloodNBF18d
Blood15d
Blood96h
Blood24h
BloodTwo24h
Blood48h
Blood72h
WholeF
CarcassF
CarcassM
WholeM
GutMidF
GutMidM
SalivF
SalivM
GutHind
GutAnt
GutCaeca
GutPost
Blood3h
BloodNBF
BloodTwoNBF
FatBodyBF
GutMidBF
Mean 3.13 +/− 1.05

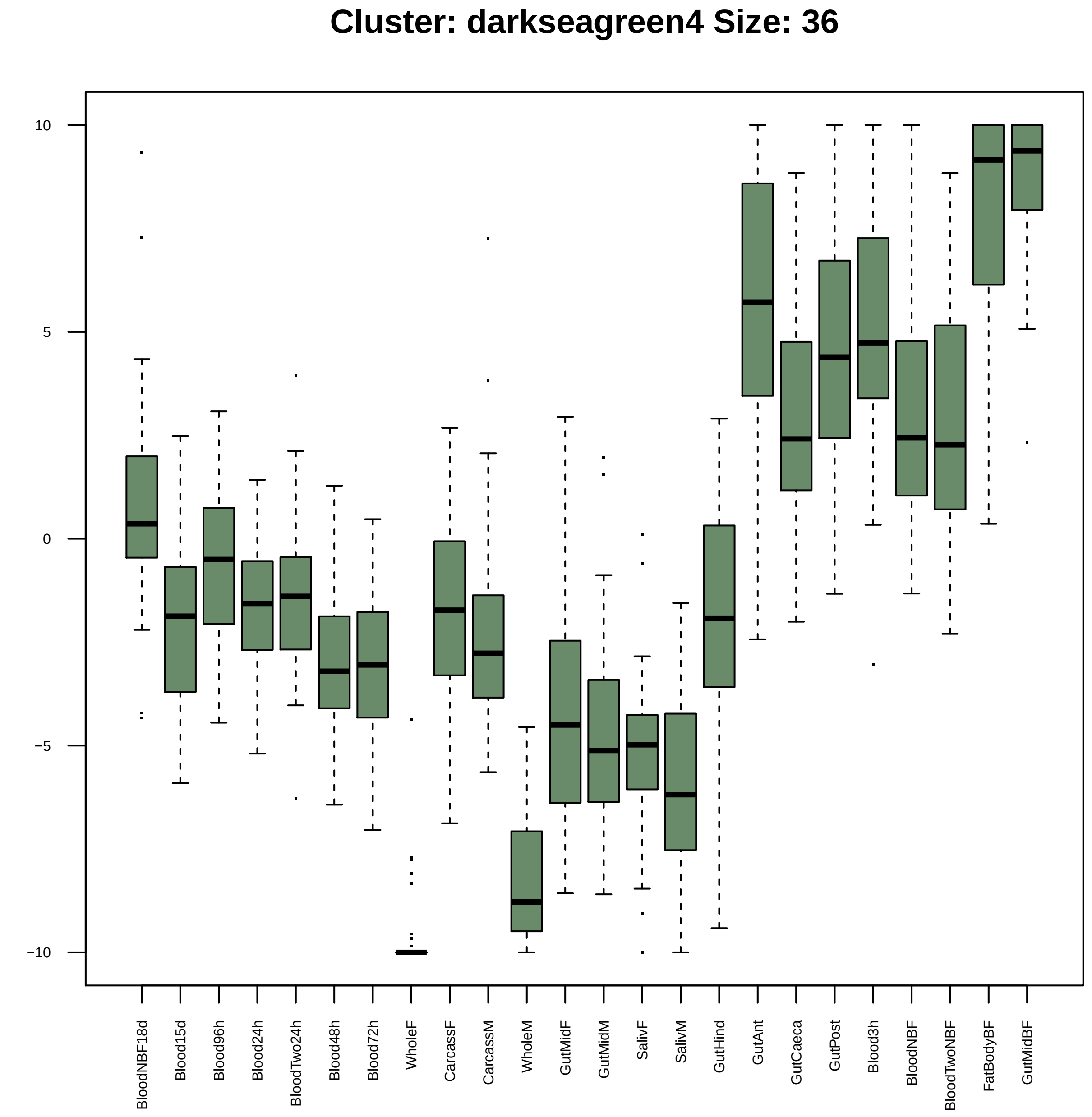
Cluster: darkseagreen4 Size: 36
10
5
0
−5
−10
BloodNBF18d
Blood15d
Blood96h
Blood24h
BloodTwo24h
Blood48h
Blood72h
WholeF
CarcassF
CarcassM
WholeM
GutMidF
GutMidM
SalivF
SalivM
GutHind
GutAnt
GutCaeca
GutPost
Blood3h
BloodNBF
BloodTwoNBF
FatBodyBF
GutMidBF

| | GO.ID | BPCluster: darkseagreen4 Size: 36 | Annotated | Significant | Expected | Rank in ClassicF | Weight01F | ClassicF |
|---|---|---|---|---|---|---|---|---|
| 1 | GO:0043269 | regulation of ion transport | 31 | 2 | 0.09 | 1 | 0.0037 | 0.0037 |

| | GO.ID | MFCluster: darkseagreen4 Size: 36 | Annotated | Significant | Expected | Rank in ClassicF | Weight01F | ClassicF |
|---|---|---|---|---|---|---|---|---|
| *29* | GO:0038023 | signaling receptor activity | 282 | 4 | 0.86 | 2 | 1.000 | 0.0096 |

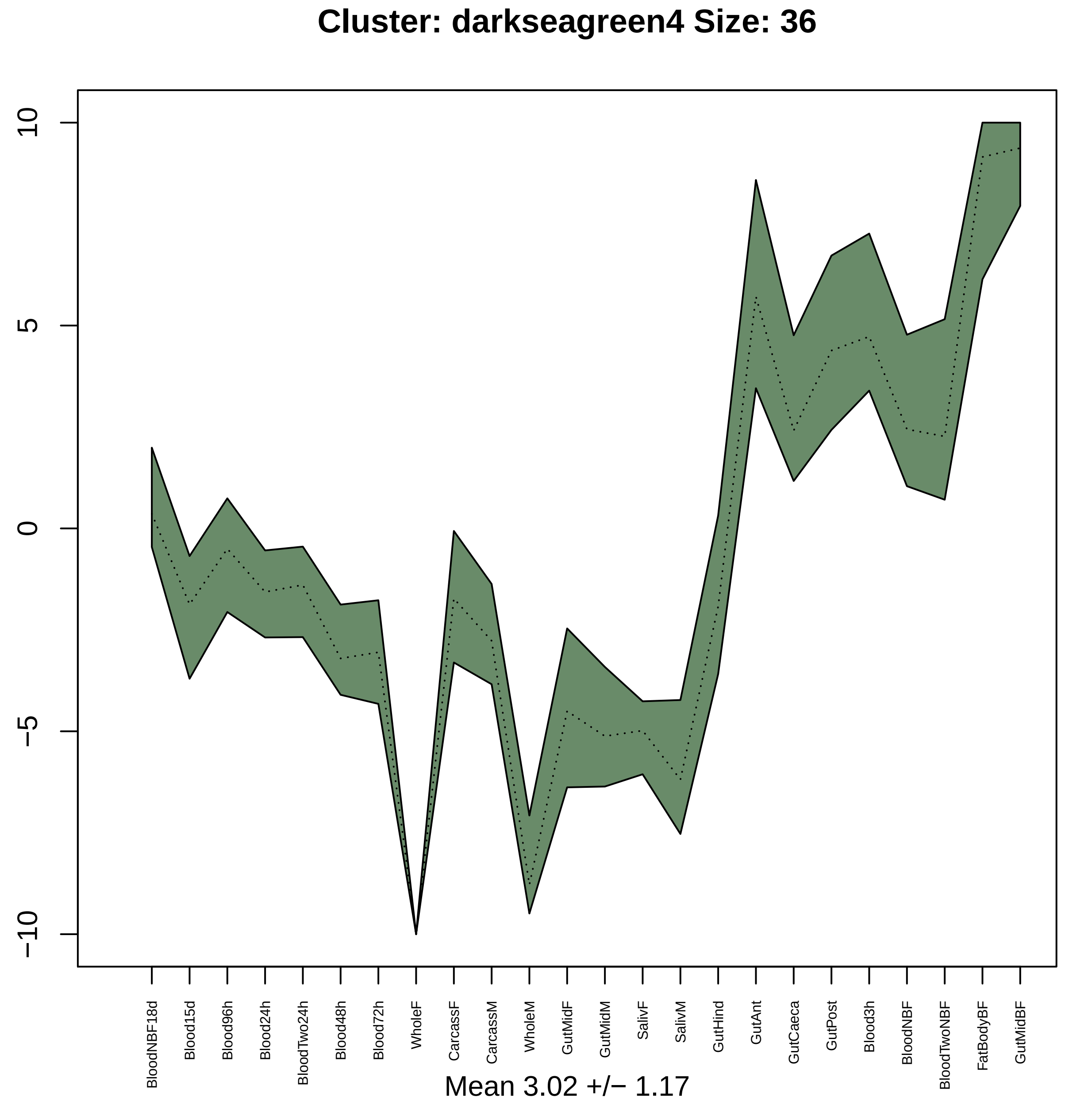
Cluster: darkseagreen4 Size: 36
10
5
0
−5
−10
BloodNBF18d
Blood15d
Blood96h
Blood24h
BloodTwo24h
Blood48h
Blood72h
WholeF
CarcassF
CarcassM
WholeM
GutMidF
GutMidM
SalivF
SalivM
GutHind
GutAnt
GutCaeca
GutPost
Blood3h
BloodNBF
BloodTwoNBF
FatBodyBF
GutMidBF
Mean 3.02 +/− 1.17

# Cluster: thistle4 Size: 17

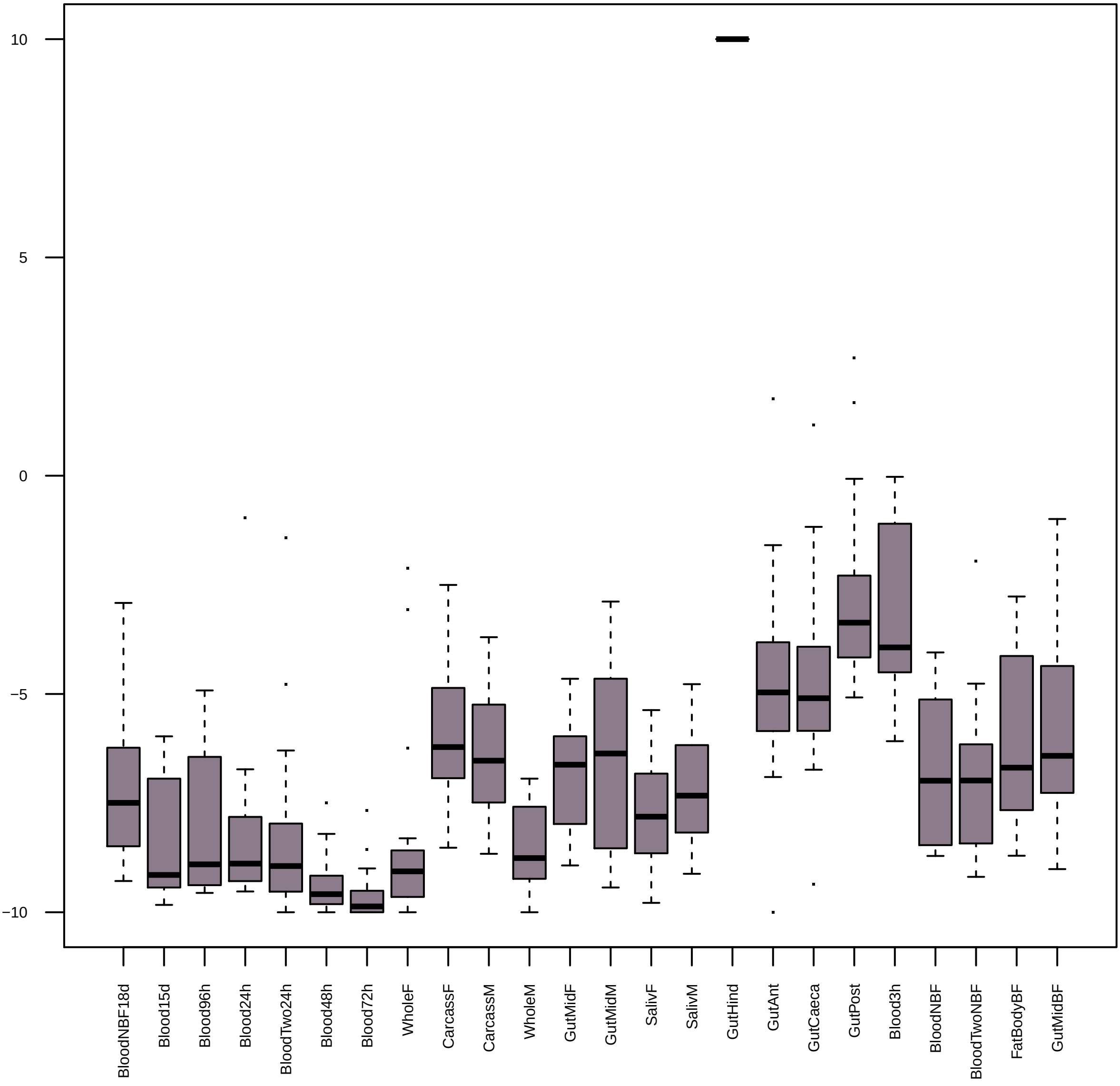

| | GO.ID | MFCluster: thistle4 Size: 17 | Annotated | Significant | Expected | Rank in ClassicF | Weight01F | ClassicF |
|---|---|---|---|---|---|---|---|---|
| *1* | GO:0008061 | chitin binding | 76 | 2 | 0.15 | 1 | 0.0092 | 0.0092 |

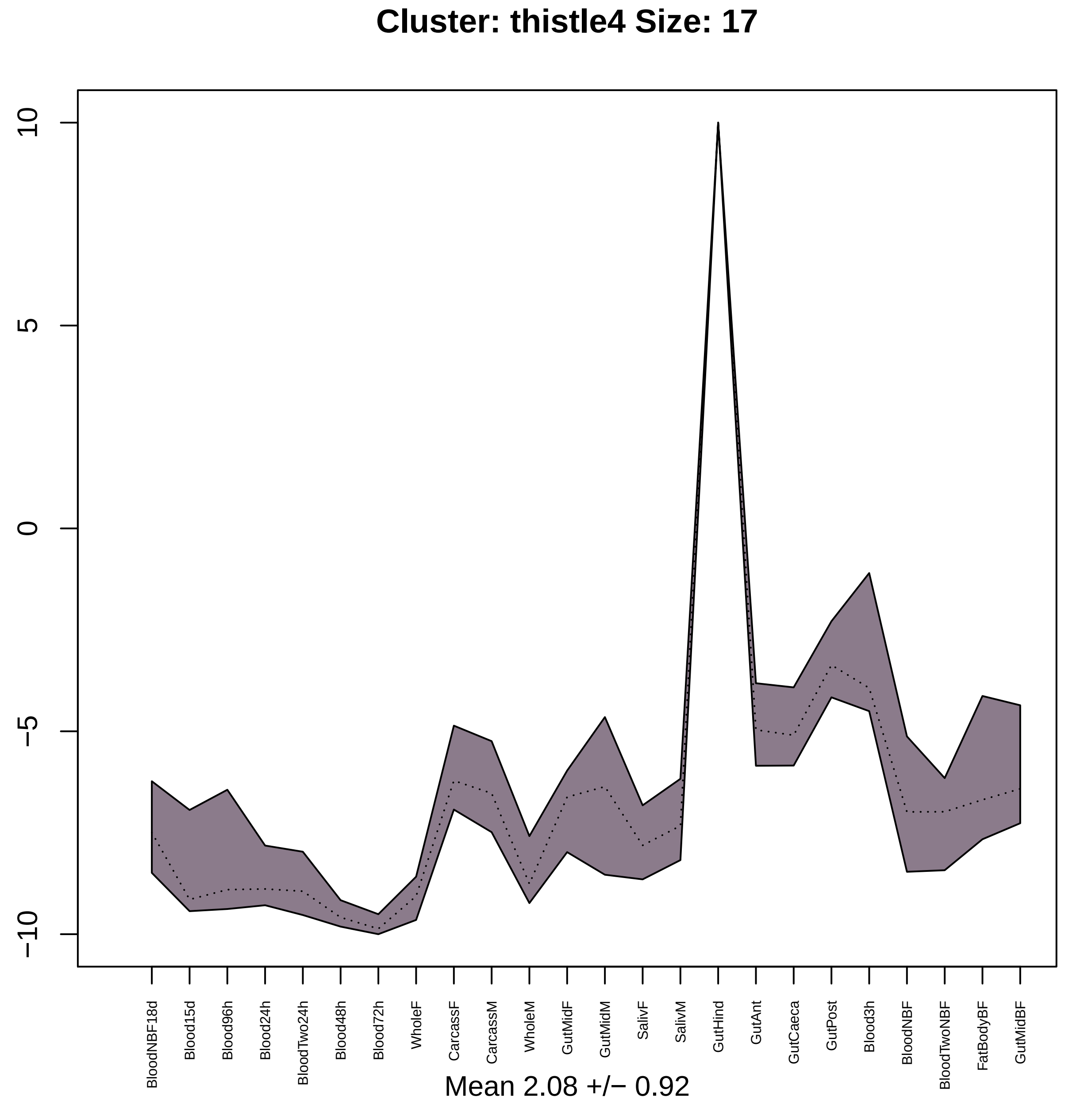

Cluster: thistle4 Size: 17
10
5
0
−5
−10
BloodNBF18d
Blood15d
Blood96h
Blood24h
BloodTwo24h
Blood48h
Blood72h
WholeF
CarcassF
CarcassM
WholeM
GutMidF
GutMidM
SalivF
SalivM
GutHind
GutAnt
GutCaeca
GutPost
Blood3h
BloodNBF
BloodTwoNBF
FatBodyBF
GutMidBF
Mean 2.08 +/− 0.92

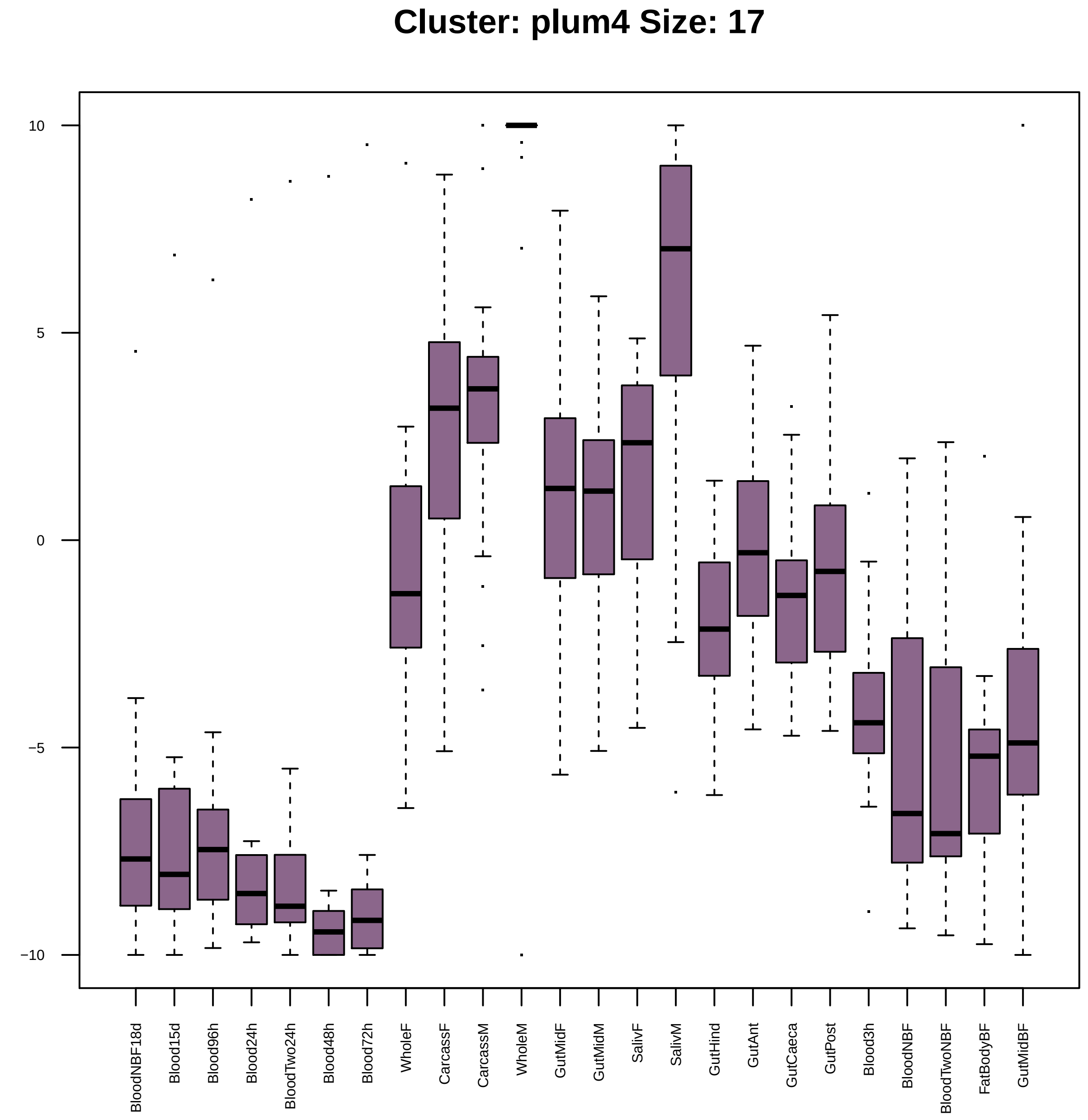

Cluster: plum4 Size: 17
10
5
0
−5
−10
BloodNBF18d
Blood15d
Blood96h
Blood24h
BloodTwo24h
Blood48h
Blood72h
WholeF
CarcassF
CarcassM
WholeM
GutMidF
GutMidM
SalivF
SalivM
GutHind
GutAnt
GutCaeca
GutPost
Blood3h
BloodNBF
BloodTwoNBF
FatBodyBF
GutMidBF

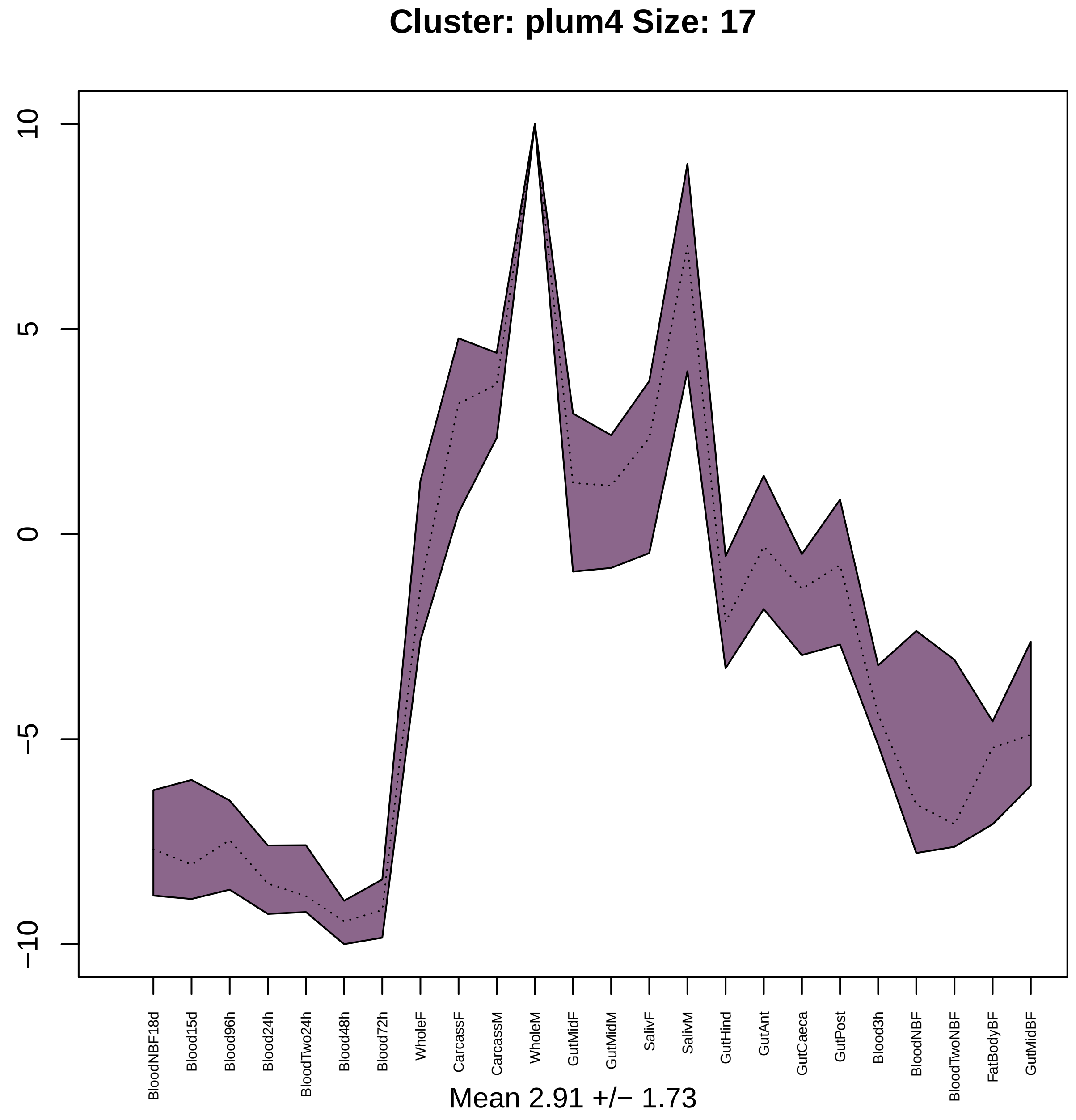

Cluster: plum4 Size: 17
10
5
0
−5
−10
BloodNBF18d
Blood15d
Blood96h
Blood24h
BloodTwo24h
Blood48h
Blood72h
WholeF
CarcassF
CarcassM
WholeM
GutMidF
GutMidM
SalivF
SalivM
GutHind
GutAnt
GutCaeca
GutPost
Blood3h
BloodNBF
BloodTwoNBF
FatBodyBF
GutMidBF
Mean 2.91 +/− 1.73

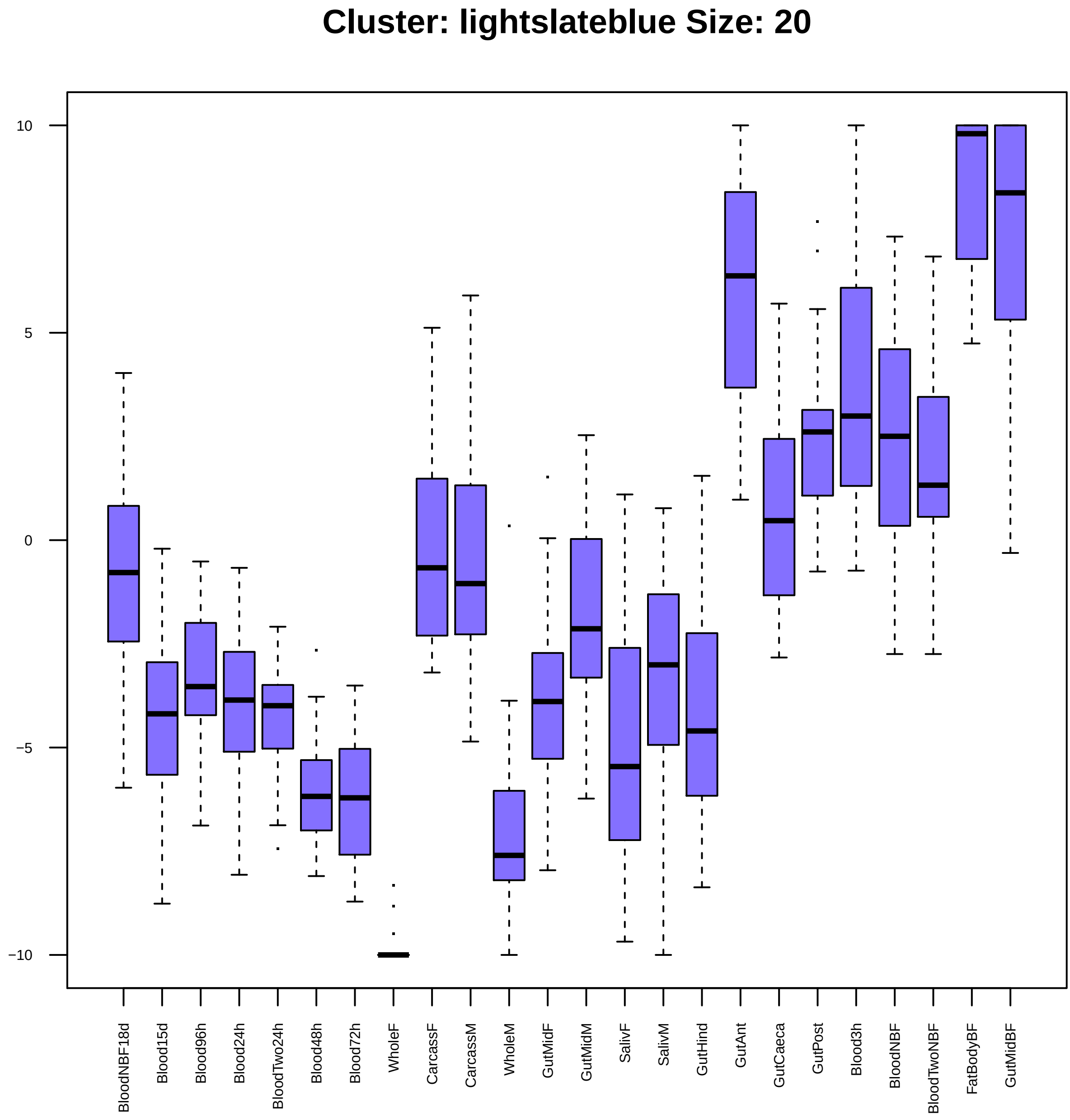
Cluster: lightslateblue Size: 20
10
5
0
−5
−10
BloodNBF18d
Blood15d
Blood96h
Blood24h
BloodTwo24h
Blood48h
Blood72h
WholeF
CarcassF
CarcassM
WholeM
GutMidF
GutMidM
SalivF
SalivM
GutHind
GutAnt
GutCaeca
GutPost
Blood3h
BloodNBF
BloodTwoNBF
FatBodyBF
GutMidBF

| | GO.ID | BPCluster: lightslateblue Size: 20 | Annotated | Significant | Expected | Rank in ClassicF | Weight01F | ClassicF |
|---|---|---|---|---|---|---|---|---|
| *1* | GO:0048639 | positive regulation of developmental gro... | 26 | 2 | 0.05 | 7 | 0.0010 | 0.0010 |
| *2* | GO:0006355 | regulation of transcription, DNA−templat... | 501 | 5 | 0.94 | 10 | 0.0011 | 0.0015 |
| *3* | GO:0048813 | dendrite morphogenesis | 93 | 3 | 0.18 | 5 | 0.0080 | 0.0006 |
| *5* | GO:0006935 | chemotaxis | 129 | 3 | 0.24 | 15 | 0.0143 | 0.0016 |
| *7* | GO:0048518 | positive regulation of biological proces... | 430 | 4 | 0.81 | 43 | 0.0195 | 0.0065 |
| *29* | GO:0007186 | G protein−coupled receptor signaling pat... | 228 | 3 | 0.43 | 47 | 0.0439 | 0.0078 |

| | GO.ID | MFCluster: lightslateblue Size: 20 | Annotated | Significant | Expected | Rank in ClassicF | Weight01F | ClassicF |
|---|---|---|---|---|---|---|---|---|
| *1* | GO:0003700 | DNA−binding transcription factor activit... | 282 | 4 | 0.51 | 3 | 0.0044 | 0.00124 |
| *2* | GO:0008528 | G protein−coupled peptide receptor activ... | 24 | 2 | 0.04 | 1 | 0.0050 | 0.00081 |
| *3* | GO:0003677 | DNA binding | 523 | 5 | 0.94 | 4 | 0.0099 | 0.00155 |
| *11* | GO:0004930 | G protein−coupled receptor activity | 171 | 3 | 0.31 | 6 | 0.2035 | 0.00315 |
| *17* | GO:0140110 | transcription regulator activity | 322 | 4 | 0.58 | 5 | 1.0000 | 0.00203 |

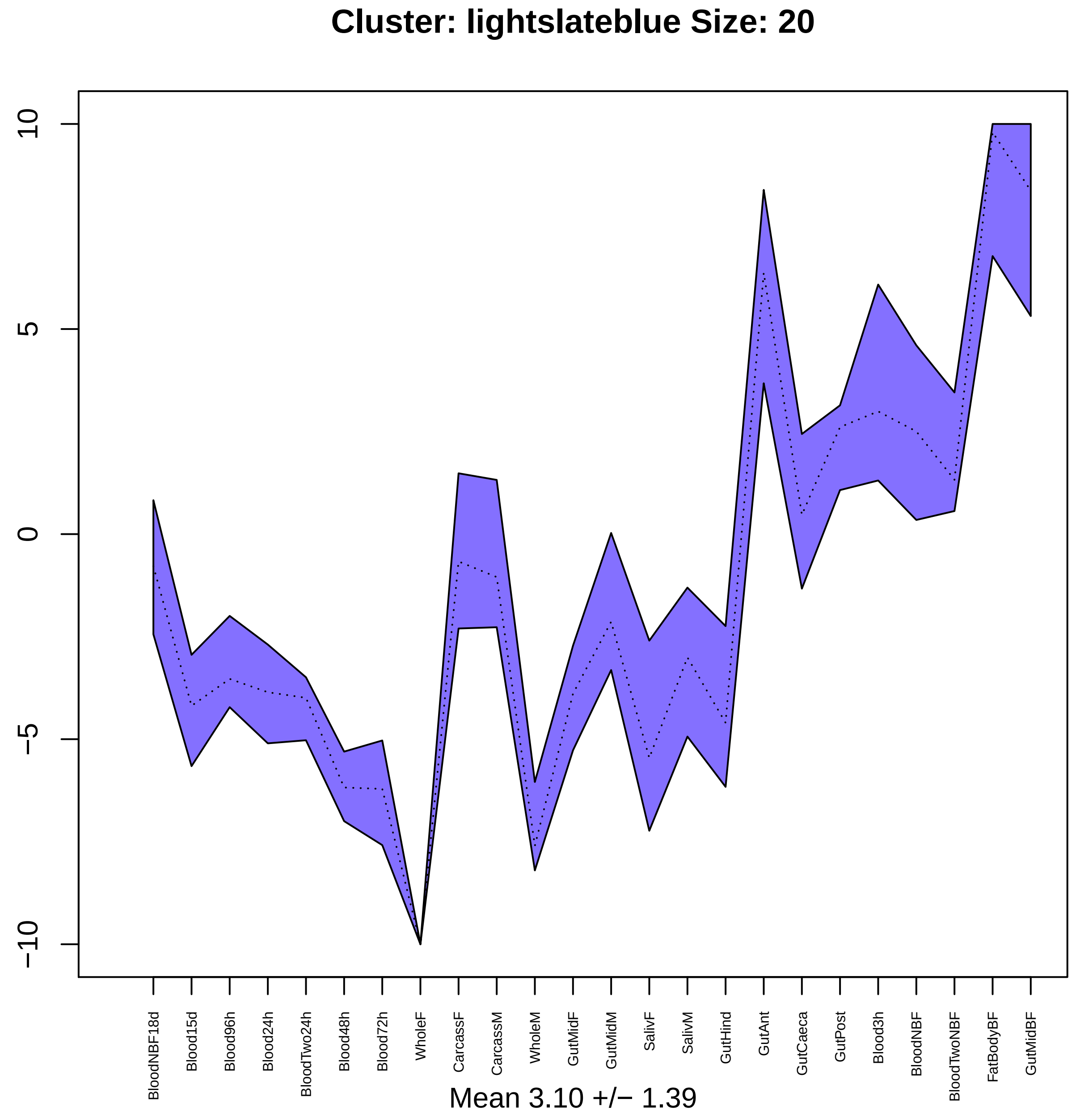
Cluster: lightslateblue Size: 20
10
5
0
−5
−10
BloodNBF18d
Blood15d
Blood96h
Blood24h
BloodTwo24h
Blood48h
Blood72h
WholeF
CarcassF
CarcassM
WholeM
GutMidF
GutMidM
SalivF
SalivM
GutHind
GutAnt
GutCaeca
GutPost
Blood3h
BloodNBF
BloodTwoNBF
FatBodyBF
GutMidBF
Mean 3.10 +/− 1.39

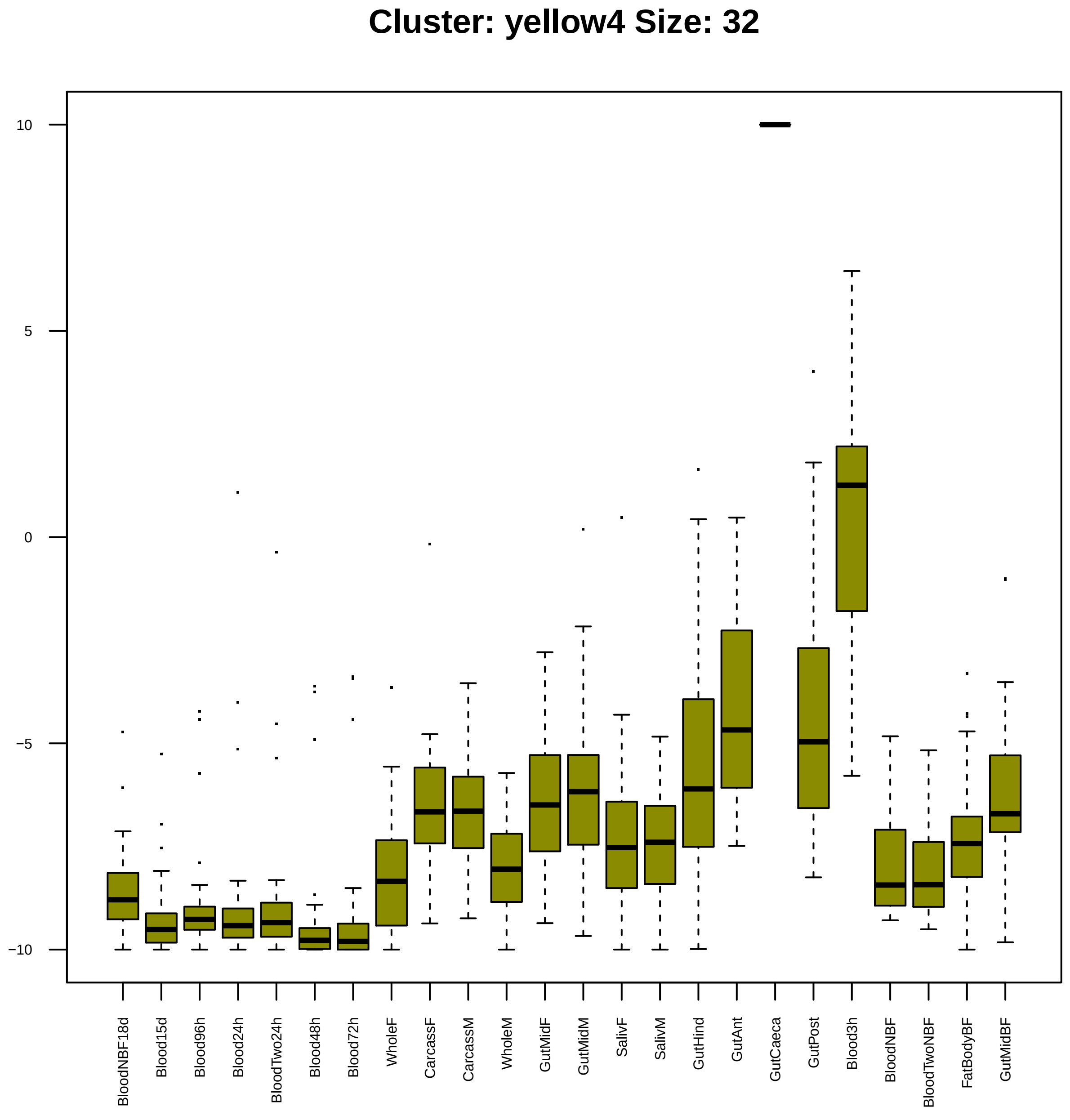

Cluster: yellow4 Size: 32
10
5
0
−5
−10
BloodNBF18d
Blood15d
Blood96h
Blood24h
BloodTwo24h
Blood48h
Blood72h
WholeF
CarcassF
CarcassM
WholeM
GutMidF
GutMidM
SalivF
SalivM
GutHind
GutAnt
GutCaeca
GutPost
Blood3h
BloodNBF
BloodTwoNBF
FatBodyBF
GutMidBF

| | GO.ID | BPCluster: yellow4 Size: 32 | Annotated | Significant | Expected | Rank in ClassicF | Weight01F | ClassicF |
|---|---|---|---|---|---|---|---|---|
| *2* | GO:0006508 | proteolysis | 594 | 7 | 2.33 | 12 | 0.0064 | 0.00635 |
| *23* | GO:0006807 | nitrogen compound metabolic process | 2967 | 20 | 11.63 | 10 | 1.0000 | 0.00064 |
| *26* | GO:0043170 | macromolecule metabolic process | 2534 | 19 | 9.93 | 8 | 1.0000 | 0.00025 |

| | GO.ID | MFCluster: yellow4 Size: 32 | Annotated | Significant | Expected | Rank in ClassicF | Weight01F | ClassicF |
|---|---|---|---|---|---|---|---|---|
| *7* | GO:0008233 | peptidase activity | 500 | 7 | 1.80 | 4 | 0.085 | 0.00153 |
| *20* | GO:0016787 | hydrolase activity | 1445 | 11 | 5.21 | 6 | 0.786 | 0.00794 |
| *21* | GO:0070011 | peptidase activity, acting on L−amino ac... | 470 | 6 | 1.70 | 5 | 1.000 | 0.00558 |
| *24* | GO:0008144 | drug binding | 721 | 10 | 2.60 | 2 | 1.000 | 0.00011 |

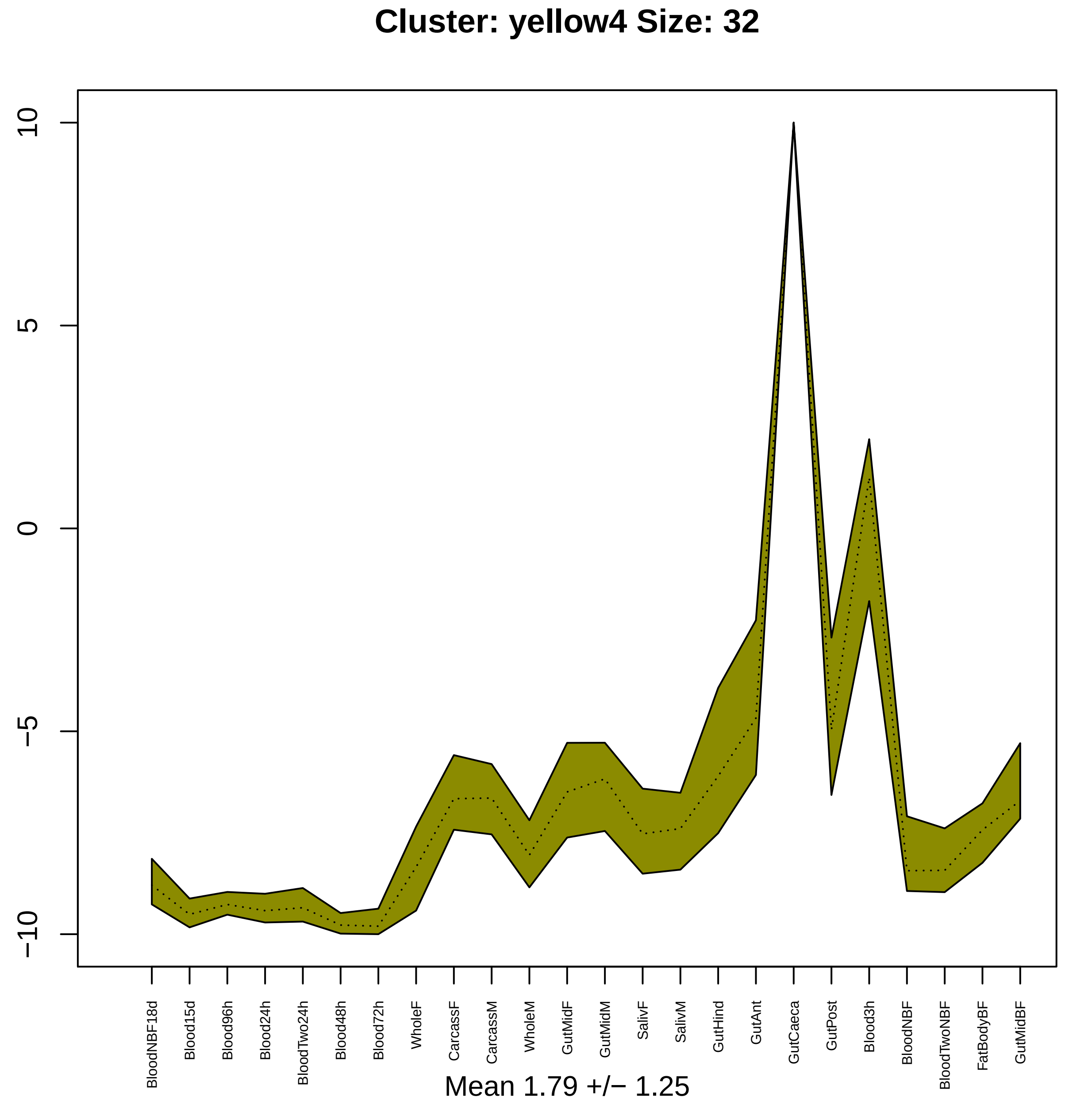
Cluster: yellow4 Size: 32
10
5
0
−5
−10
BloodNBF18d
Blood15d
Blood96h
Blood24h
BloodTwo24h
Blood48h
Blood72h
WholeF
CarcassF
CarcassM
WholeM
GutMidF
GutMidM
SalivF
SalivM
GutHind
GutAnt
GutCaeca
GutPost
Blood3h
BloodNBF
BloodTwoNBF
FatBodyBF
GutMidBF
Mean 1.79 +/− 1.25

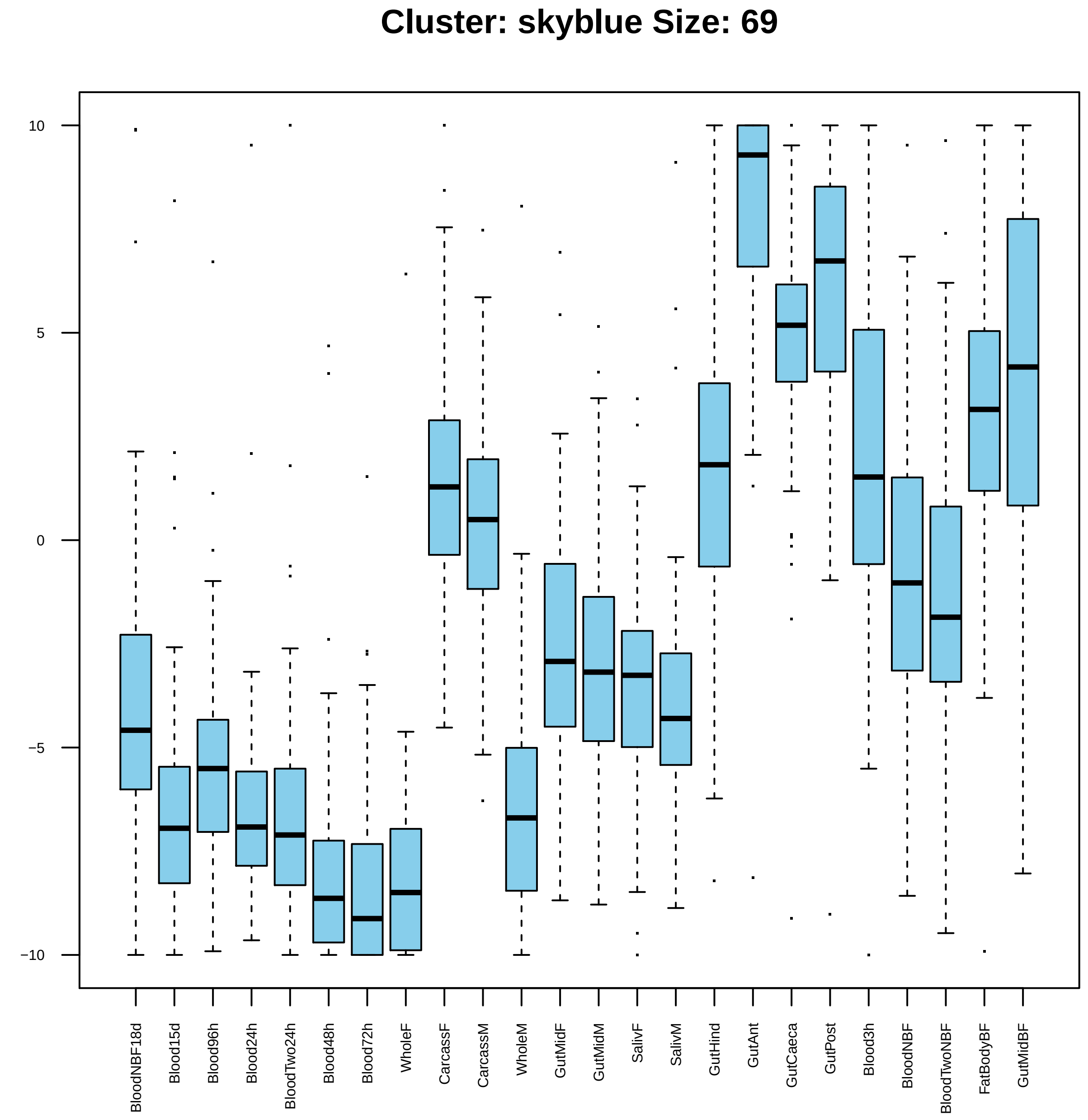
Cluster: skyblue Size: 69
10
5
0
−5
−10
BloodNBF18d
Blood15d
Blood96h
Blood24h
BloodTwo24h
Blood48h
Blood72h
WholeF
CarcassF
CarcassM
WholeM
GutMidF
GutMidM
SalivF
SalivM
GutHind
GutAnt
GutCaeca
GutPost
Blood3h
BloodNBF
BloodTwoNBF
FatBodyBF
GutMidBF

| | GO.ID | BPCluster: skyblue Size: 69 | Annotated | Significant | Expected | Rank in ClassicF | Weight01F | ClassicF |
|---|---|---|---|---|---|---|---|---|
| *1* | GO:0007448 | anterior/posterior pattern specification... | 12 | 2 | 0.08 | 16 | 0.0028 | 0.00281 |
| *2* | GO:0035725 | sodium ion transmembrane transport | 46 | 3 | 0.31 | 17 | 0.0035 | 0.00354 |
| *3* | GO:0007398 | ectoderm development | 14 | 2 | 0.09 | 18 | 0.0038 | 0.00384 |
| *4* | GO:0071310 | cellular response to organic substance | 107 | 5 | 0.72 | 7 | 0.0057 | 0.00070 |
| *11* | GO:0007166 | cell surface receptor signaling pathway | 328 | 8 | 2.21 | 10 | 0.0258 | 0.00134 |
| *23* | GO:0006814 | sodium ion transport | 57 | 4 | 0.38 | 6 | 0.0674 | 0.00055 |

| | GO.ID | MFCluster: skyblue Size: 69 | Annotated | Significant | Expected | Rank in ClassicF | Weight01F | ClassicF |
|---|---|---|---|---|---|---|---|---|
| *1* | GO:0005272 | sodium channel activity | 26 | 3 | 0.17 | 14 | 0.00061 | 0.00061 |
| *2* | GO:0043565 | sequence−specific DNA binding | 212 | 6 | 1.38 | 20 | 0.00381 | 0.00236 |
| *3* | GO:0008188 | neuropeptide receptor activity | 21 | 2 | 0.14 | 27 | 0.00807 | 0.00807 |
| *4* | GO:0003700 | DNA−binding transcription factor activit... | 282 | 7 | 1.84 | 18 | 0.00932 | 0.00213 |
| *5* | GO:0022836 | gated channel activity | 89 | 5 | 0.58 | 10 | 0.01204 | 0.00026 |
| *9* | GO:0008528 | G protein−coupled peptide receptor activ... | 24 | 3 | 0.16 | 13 | 0.01867 | 0.00048 |
| *21* | GO:0005230 | extracellular ligand−gated ion channel a... | 51 | 3 | 0.33 | 22 | 0.12386 | 0.00434 |
| *24* | GO:0022839 | ion gated channel activity | 87 | 4 | 0.57 | 21 | 0.14856 | 0.00238 |

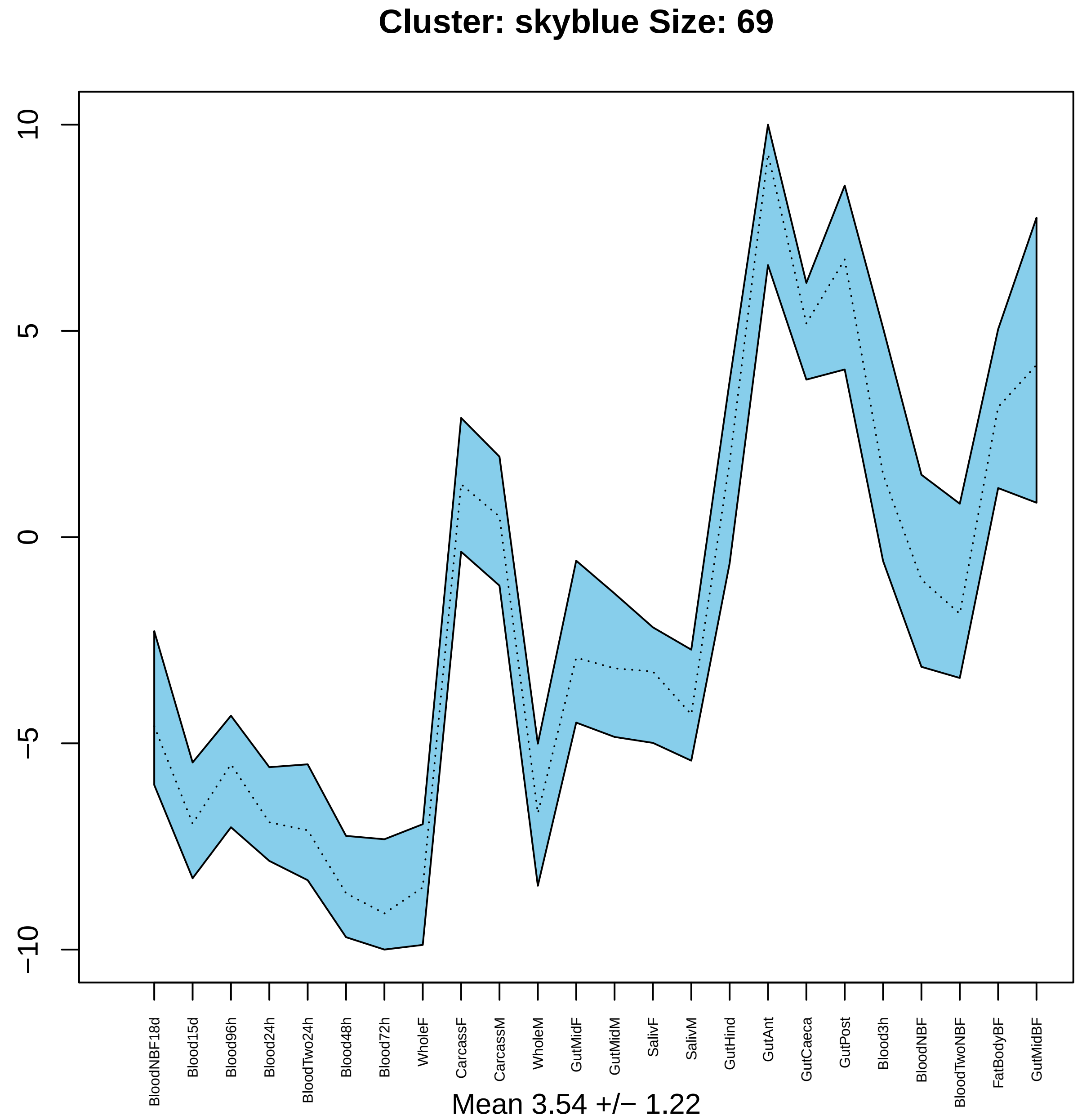
Cluster: skyblue Size: 69
10
5
0
−5
−10
BloodNBF18d
Blood15d
Blood96h
Blood24h
BloodTwo24h
Blood48h
Blood72h
WholeF
CarcassF
CarcassM
WholeM
GutMidF
GutMidM
SalivF
SalivM
GutHind
GutAnt
GutCaeca
GutPost
Blood3h
BloodNBF
BloodTwoNBF
FatBodyBF
GutMidBF
Mean 3.54 +/− 1.22

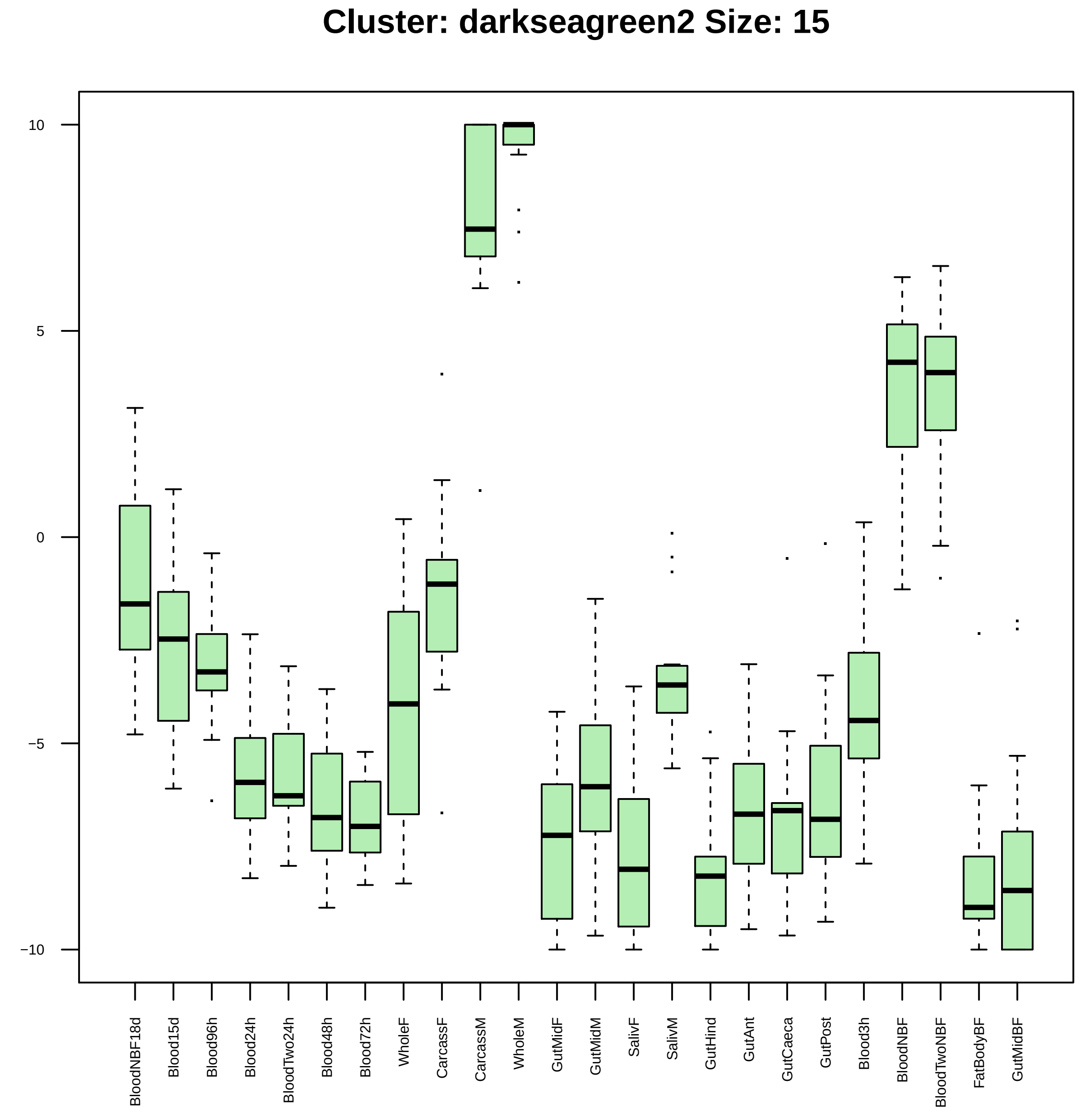
Cluster: darkseagreen2 Size: 15
10
5
0
−5
−10
BloodNBF18d
Blood15d
Blood96h
Blood24h
BloodTwo24h
Blood48h
Blood72h
WholeF
CarcassF
CarcassM
WholeM
GutMidF
GutMidM
SalivF
SalivM
GutHind
GutAnt
GutCaeca
GutPost
Blood3h
BloodNBF
BloodTwoNBF
FatBodyBF
GutMidBF

| | GO.ID | BPCluster: darkseagreen2 Size: 15 | Annotated | Significant | Expected | Rank in ClassicF | Weight01F | ClassicF |
|---|---|---|---|---|---|---|---|---|
| *1* | GO:0006836 | neurotransmitter transport | 81 | 2 | 0.1 | 1 | 0.0042 | 0.0042 |

|  | GO.ID | MFCluster: darkseagreen2 Size: 15 | Annotated | Significant | Expected | Rank in ClassicF | Weight01F | ClassicF |
|---|---|---|---|---|---|---|---|---|
| 1 | GO:0017022 | myosin binding | 19 | 2 | 0.03 | 1 | 0.00029 | 0.00029 |
| 2 | GO:0005516 | calmodulin binding | 19 | 2 | 0.03 | 2 | 0.00029 | 0.00029 |

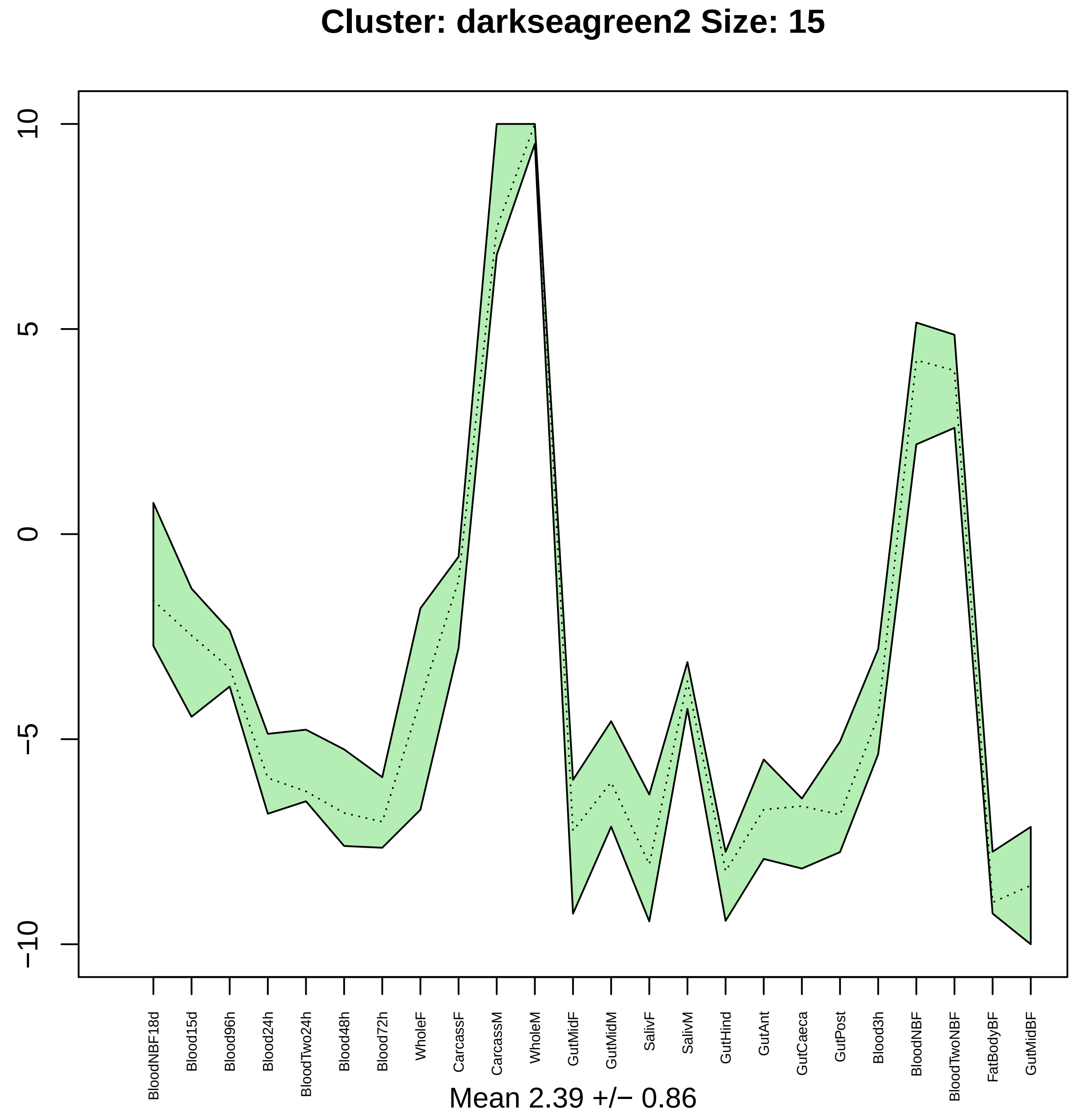

Cluster: darkseagreen2 Size: 15
10
5
0
−5
−10
BloodNBF18d
Blood15d
Blood96h
Blood24h
BloodTwo24h
Blood48h
Blood72h
WholeF
CarcassF
CarcassM
WholeM
GutMidF
GutMidM
SalivF
SalivM
GutHind
GutAnt
GutCaeca
GutPost
Blood3h
BloodNBF
BloodTwoNBF
FatBodyBF
GutMidBF
Mean 2.39 +/− 0.86

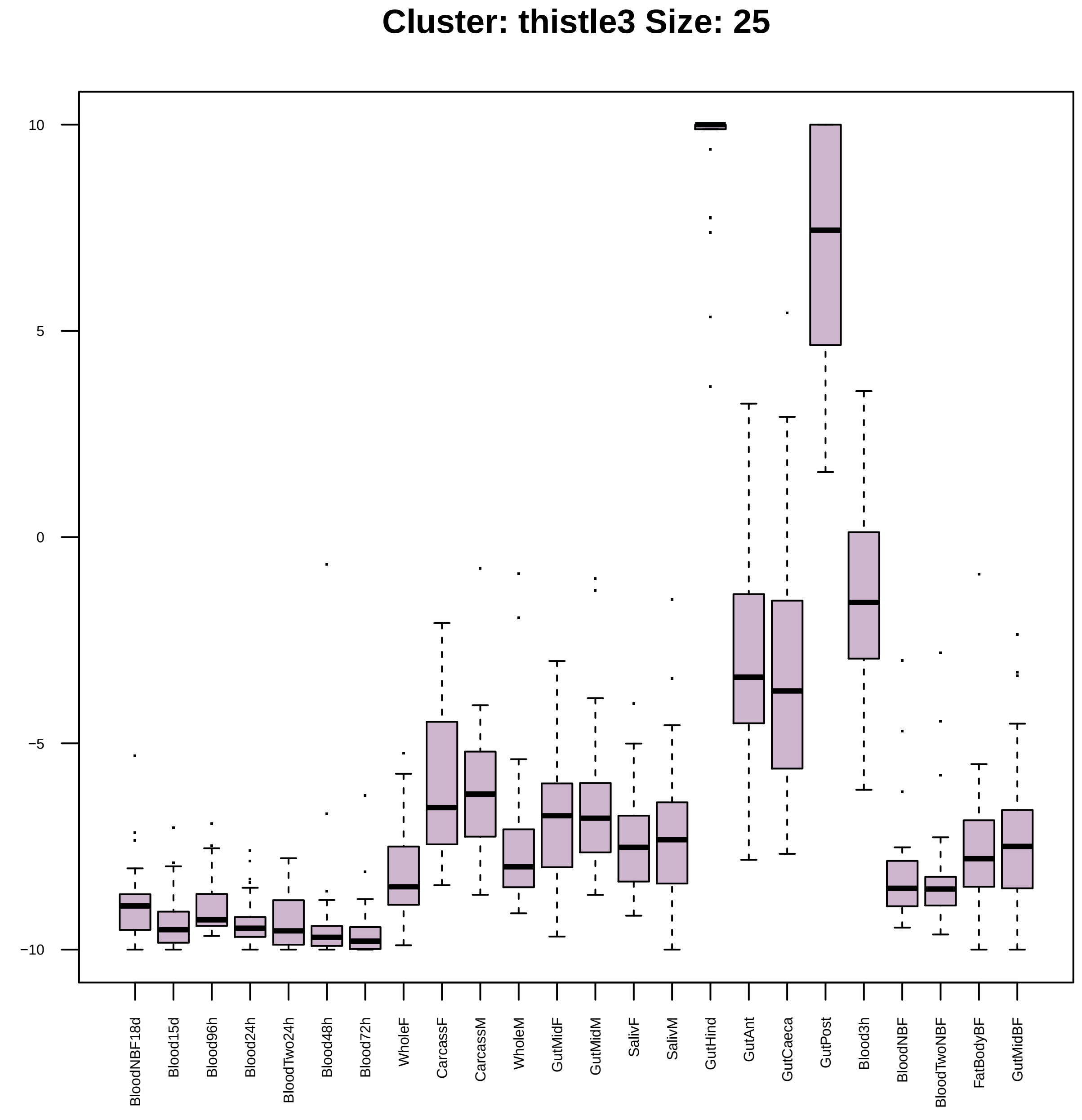

Cluster: thistle3 Size: 25
10
5
0
−5
−10
BloodNBF18d
Blood15d
Blood96h
Blood24h
BloodTwo24h
Blood48h
Blood72h
WholeF
CarcassF
CarcassM
WholeM
GutMidF
GutMidM
SalivF
SalivM
GutHind
GutAnt
GutCaeca
GutPost
Blood3h
BloodNBF
BloodTwoNBF
FatBodyBF
GutMidBF

|  | GO.ID | BPCluster: thistle3 Size: 25 | Annotated | Significant | Expected | Rank in ClassicF | Weight01F | ClassicF |
|---|---|---|---|---|---|---|---|---|
| *2* | GO:0006508 | proteolysis | 594 | 7 | 1.86 | 8 | 0.0015 | 0.00155 |
| *18* | GO:1901564 | organonitrogen compound metabolic proces... | 1889 | 14 | 5.92 | 7 | 1.0000 | 0.00022 |

| | GO.ID | MFCluster: thistle3 Size: 25 | Annotated | Significant | Expected | Rank in ClassicF | Weight01F | ClassicF |
|---|---|---|---|---|---|---|---|---|
| 2 | GO:0004181 | metallocarboxypeptidase activity | 18 | 2 | 0.04 | 4 | 0.00088 | 0.00088 |
| 3 | GO:0008236 | serine−type peptidase activity | 278 | 4 | 0.69 | 8 | 0.00156 | 0.00432 |
| 16 | GO:0017171 | serine hydrolase activity | 279 | 4 | 0.70 | 9 | 1.00000 | 0.00437 |
| 22 | GO:0008233 | peptidase activity | 500 | 7 | 1.25 | 3 | 1.00000 | 0.00012 |
| 24 | GO:0008235 | metalloexopeptidase activity | 29 | 2 | 0.07 | 7 | 1.00000 | 0.00230 |
| 27 | GO:0008237 | metallopeptidase activity | 104 | 3 | 0.26 | 6 | 1.00000 | 0.00203 |
| 29 | GO:0004180 | carboxypeptidase activity | 24 | 2 | 0.06 | 5 | 1.00000 | 0.00157 |

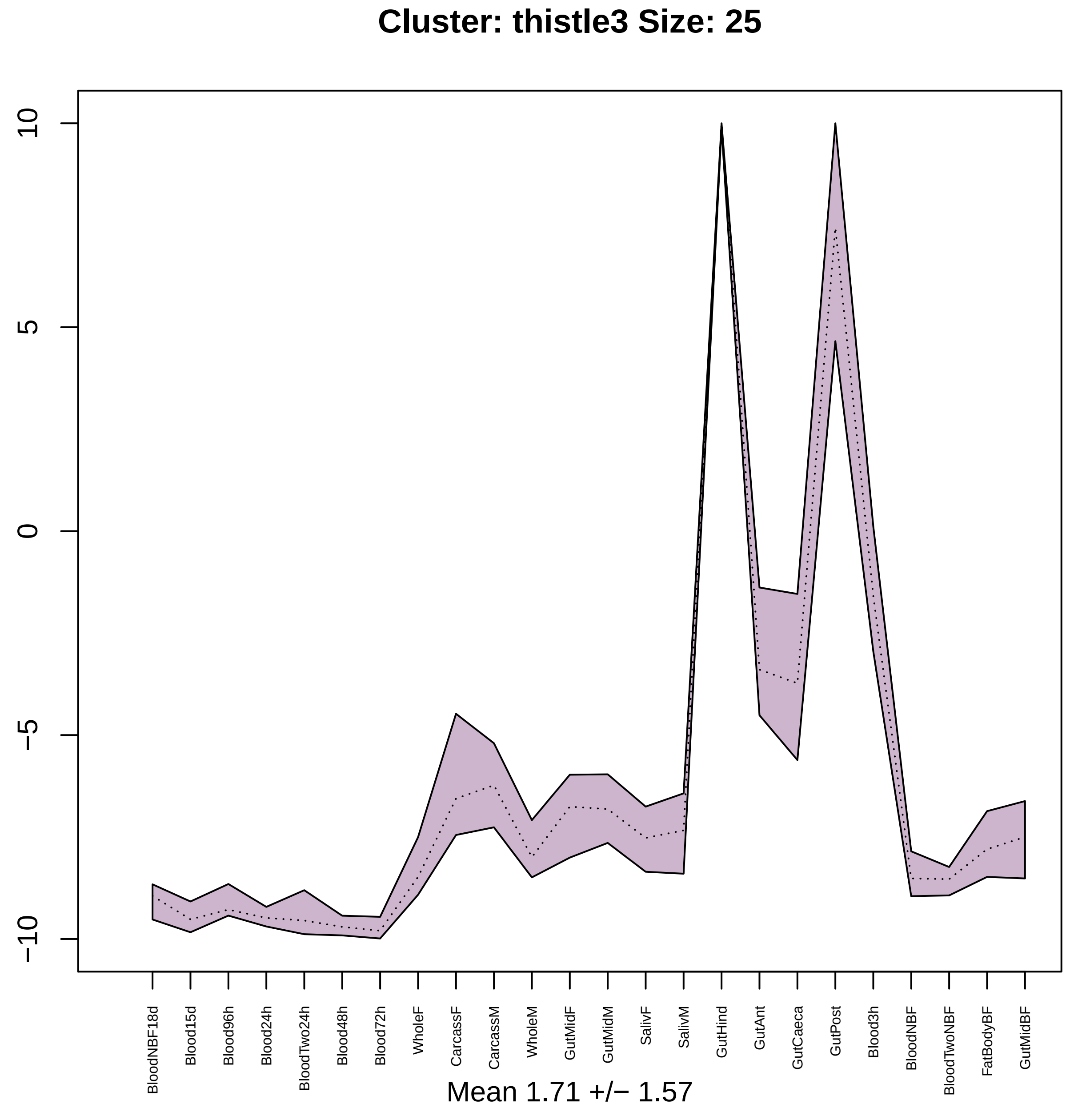

Cluster: thistle3 Size: 25
10
5
0
−5
−10
BloodNBF18d
Blood15d
Blood96h
Blood24h
BloodTwo24h
Blood48h
Blood72h
WholeF
CarcassF
CarcassM
WholeM
GutMidF
GutMidM
SalivF
SalivM
GutHind
GutAnt
GutCaeca
GutPost
Blood3h
BloodNBF
BloodTwoNBF
FatBodyBF
GutMidBF
Mean 1.71 +/− 1.57

## Cluster: lightcyan Size: 107

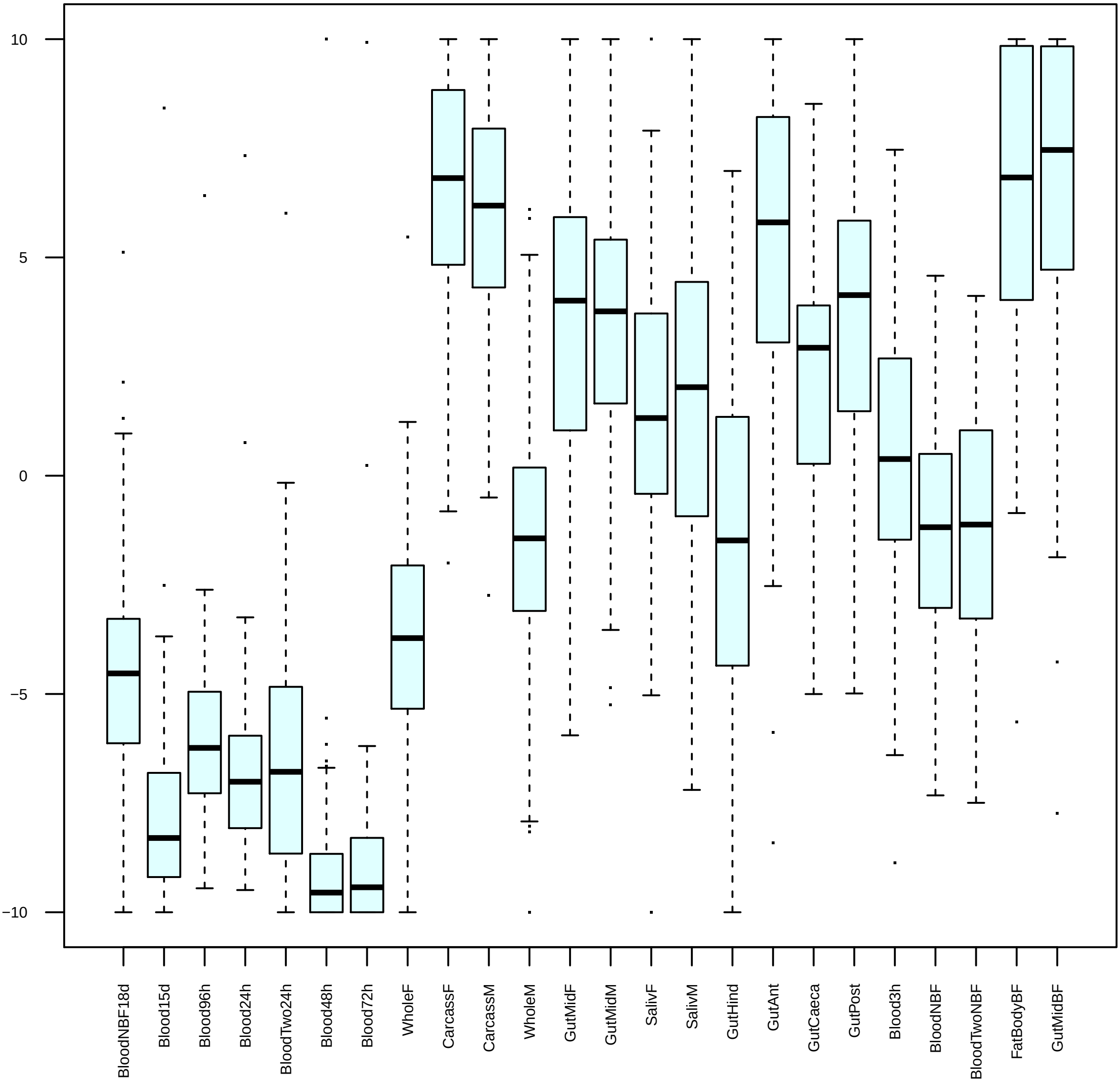

| | GO.ID | BPCluster: lightcyan Size: 107 | Annotated | Significant | Expected | Rank in ClassicF | Weight01F | ClassicF |
|---|---|---|---|---|---|---|---|---|
| 1 | GO:0050877 | nervous system process | 284 | 10 | 2.54 | 8 | 0.00013 | 0.00018 |
| 2 | GO:0000122 | negative regulation of transcription by ... | 57 | 5 | 0.51 | 6 | 0.00014 | 0.00014 |
| 3 | GO:0051052 | regulation of DNA metabolic process | 13 | 3 | 0.12 | 9 | 0.00018 | 0.00018 |
| 4 | GO:0010948 | negative regulation of cell cycle proces... | 65 | 3 | 0.58 | 90 | 0.00076 | 0.02000 |
| 5 | GO:0007186 | G protein−coupled receptor signaling pat... | 228 | 9 | 2.04 | 7 | 0.00143 | 0.00016 |
| 6 | GO:0007411 | axon guidance | 114 | 5 | 1.02 | 37 | 0.00334 | 0.00334 |
| 7 | GO:0050911 | detection of chemical stimulus involved ... | 71 | 4 | 0.63 | 39 | 0.00357 | 0.00357 |
| 8 | GO:0035147 | branch fusion, open tracheal system | 11 | 2 | 0.10 | 42 | 0.00410 | 0.00410 |
| 9 | GO:0048072 | compound eye pigmentation | 11 | 2 | 0.10 | 43 | 0.00410 | 0.00410 |
| 10 | GO:0042023 | DNA endoreduplication | 12 | 2 | 0.11 | 48 | 0.00489 | 0.00489 |
| 11 | GO:0042048 | olfactory behavior | 43 | 3 | 0.38 | 57 | 0.00649 | 0.00649 |
| 12 | GO:0048070 | regulation of developmental pigmentation | 14 | 2 | 0.13 | 58 | 0.00667 | 0.00667 |
| 13 | GO:0007419 | ventral cord development | 15 | 2 | 0.13 | 61 | 0.00765 | 0.00765 |
| 14 | GO:0007422 | peripheral nervous system development | 48 | 3 | 0.43 | 67 | 0.00881 | 0.00881 |
| 15 | GO:0035017 | cuticle pattern formation | 17 | 2 | 0.15 | 69 | 0.00980 | 0.00980 |
| 17 | GO:0006355 | regulation of transcription, DNA−templat... | 501 | 12 | 4.48 | 23 | 0.01284 | 0.00130 |
| 20 | GO:0043473 | pigmentation | 54 | 4 | 0.48 | 25 | 0.02539 | 0.00130 |
| 22 | GO:2001141 | regulation of RNA biosynthetic process | 505 | 13 | 4.51 | 15 | 0.03029 | 0.00039 |
| 24 | GO:0009593 | detection of chemical stimulus | 87 | 6 | 0.78 | 5 | 0.03265 | 0.00011 |
| 29 | GO:0007417 | central nervous system development | 98 | 5 | 0.88 | 29 | 0.03757 | 0.00172 |

| | GO.ID | MFCluster: lightcyan Size: 107 | Annotated | Significant | Expected | Rank in ClassicF | Weight01F | ClassicF |
|---|---|---|---|---|---|---|---|---|
| 1 | GO:0004930 | G protein−coupled receptor activity | 171 | 9 | 1.61 | 4 | 0.00037 | 2.9e−05 |
| 2 | GO:0043565 | sequence−specific DNA binding | 212 | 8 | 2.00 | 7 | 0.00080 | 0.00079 |
| 3 | GO:0003700 | DNA−binding transcription factor activit... | 282 | 10 | 2.66 | 6 | 0.01027 | 0.00028 |

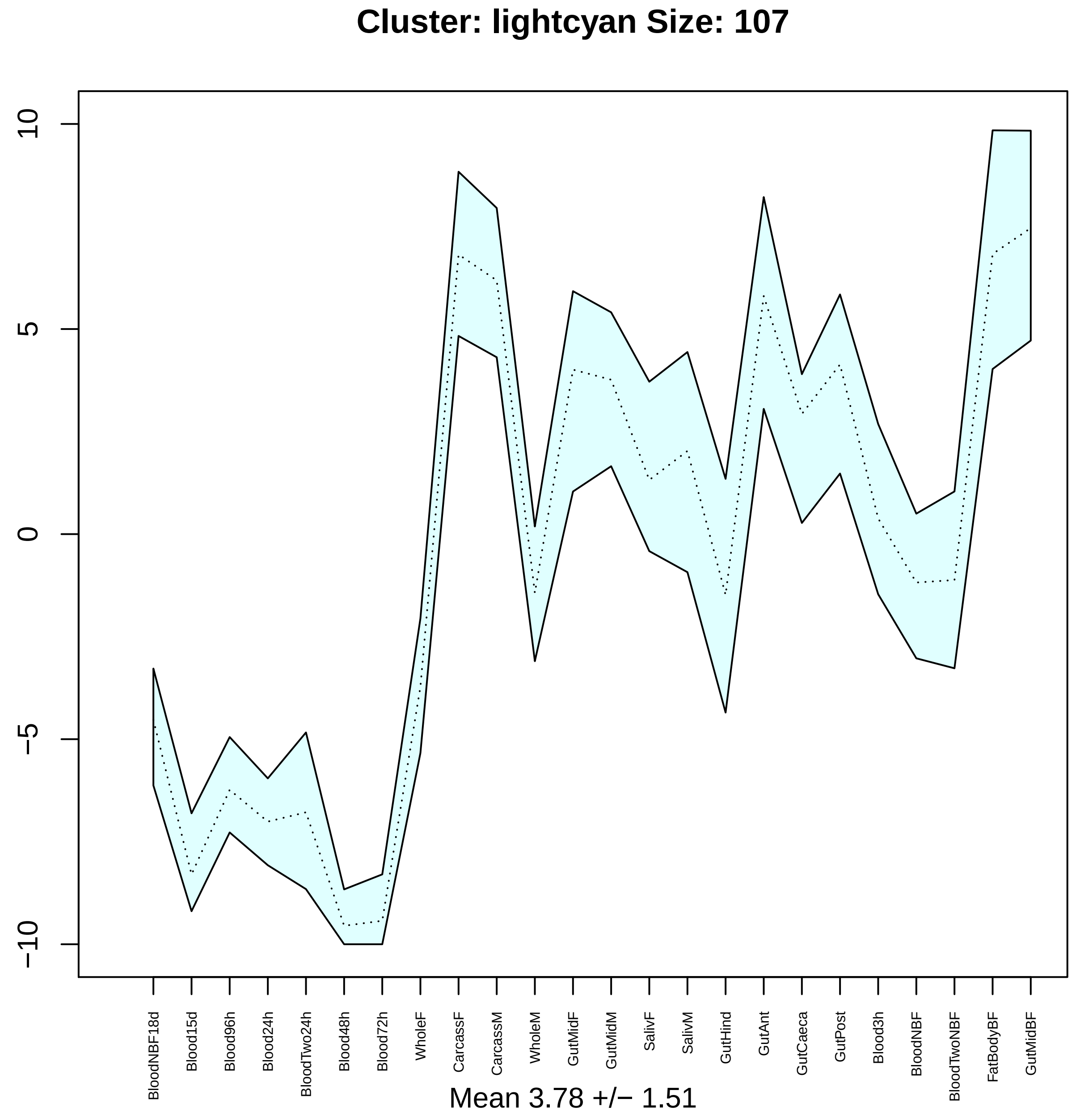
Cluster: lightcyan Size: 107
10
5
0
−5
−10
BloodNBF18d
Blood15d
Blood96h
Blood24h
BloodTwo24h
Blood48h
Blood72h
WholeF
CarcassF
CarcassM
WholeM
GutMidF
GutMidM
SalivF
SalivM
GutHind
GutAnt
GutCaeca
GutPost
Blood3h
BloodNBF
BloodTwoNBF
FatBodyBF
GutMidBF
Mean 3.78 +/− 1.51

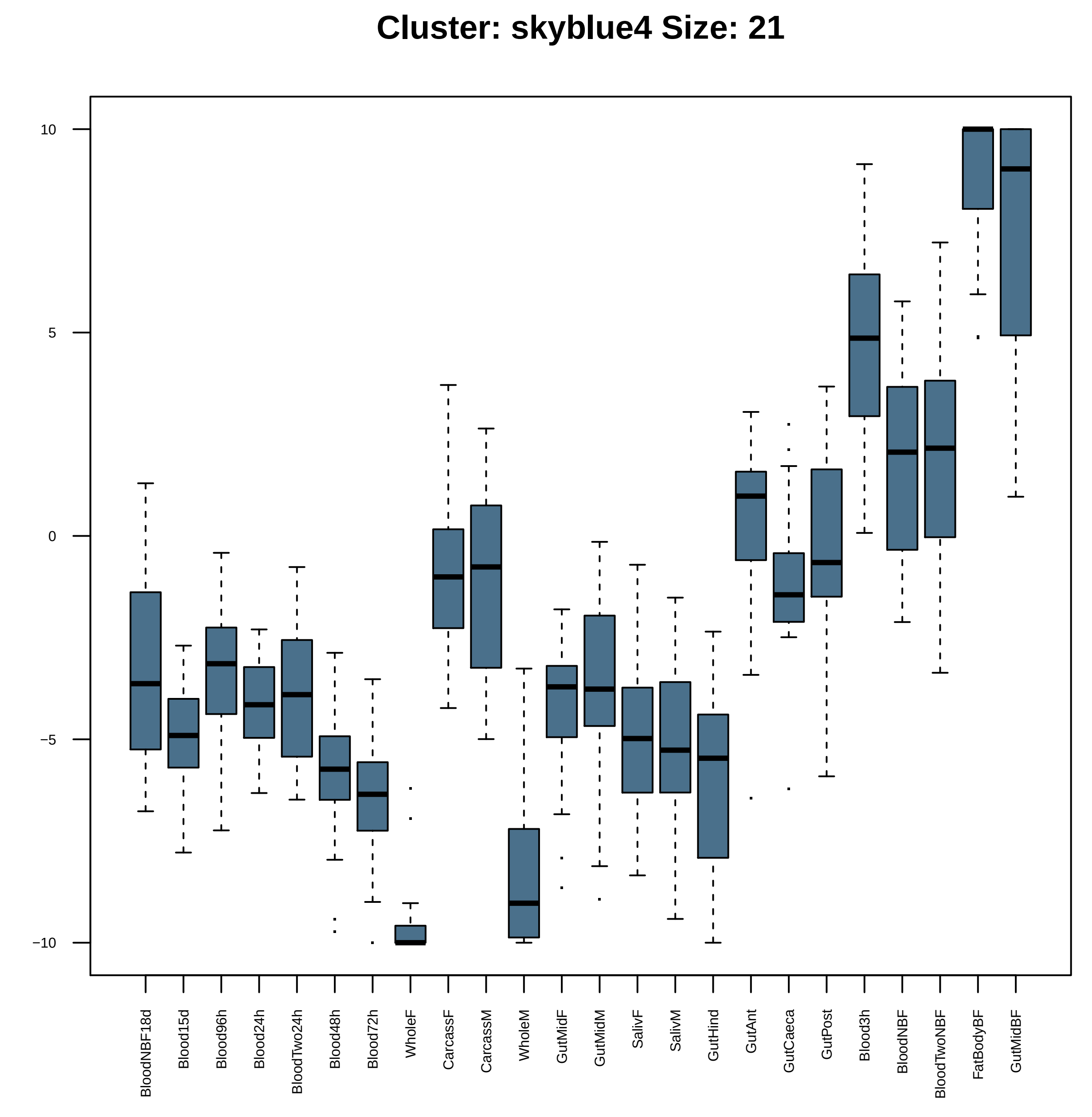

Cluster: skyblue4 Size: 21
10
5
0
−5
−10
BloodNBF18d
Blood15d
Blood96h
Blood24h
BloodTwo24h
Blood48h
Blood72h
WholeF
CarcassF
CarcassM
WholeM
GutMidF
GutMidM
SalivF
SalivM
GutHind
GutAnt
GutCaeca
GutPost
Blood3h
BloodNBF
BloodTwoNBF
FatBodyBF
GutMidBF

| | GO.ID | MFCluster: skyblue4 Size: 21 | Annotated | Significant | Expected | Rank in ClassicF | Weight01F | ClassicF |
|---|---|---|---|---|---|---|---|---|
| *1* | GO:0030545 | receptor regulator activity | 35 | 2 | 0.08 | 1 | 0.0045 | 0.003 |

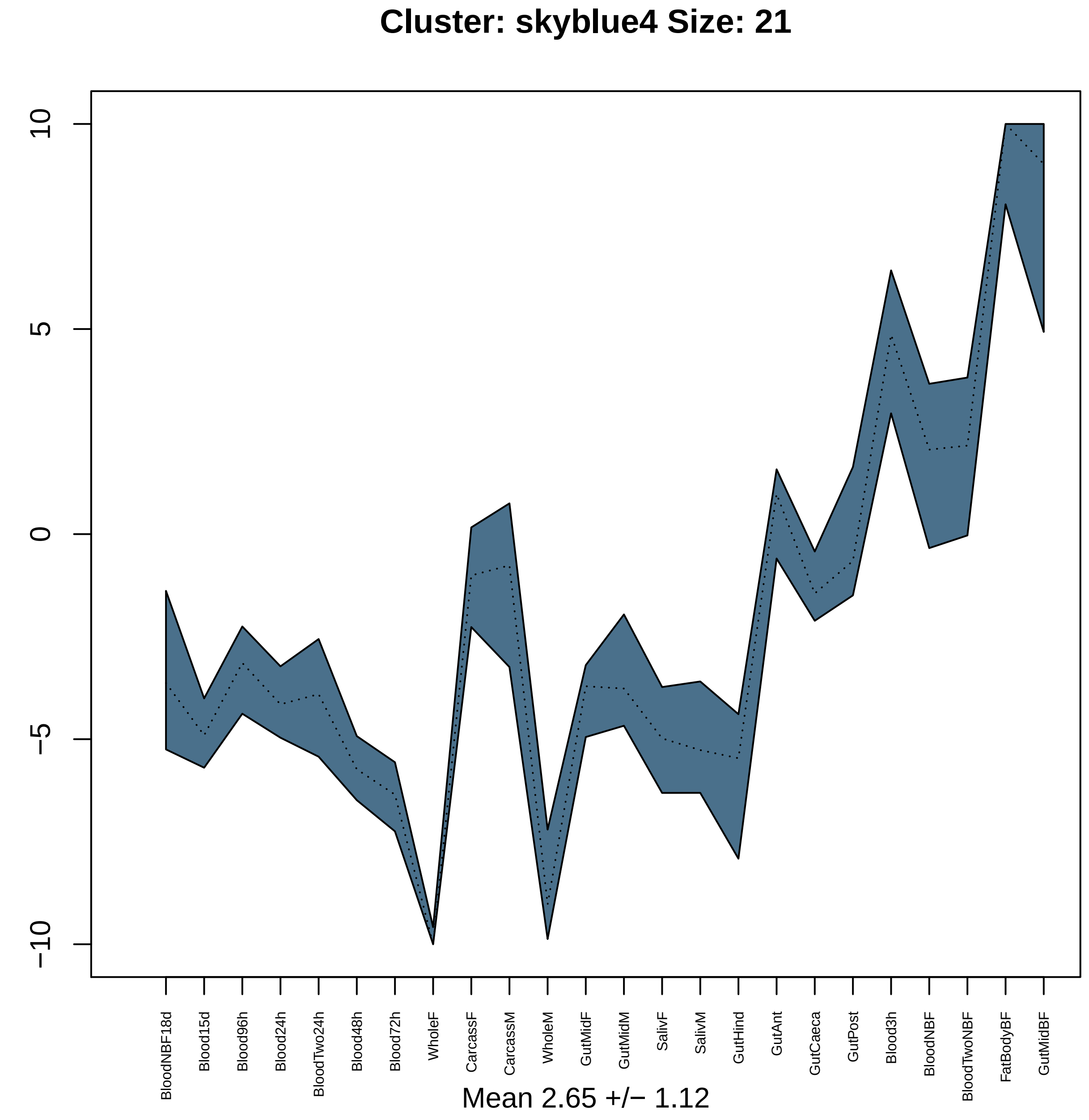
Cluster: skyblue4 Size: 21
10
5
0
−5
−10
BloodNBF18d
Blood15d
Blood96h
Blood24h
BloodTwo24h
Blood48h
Blood72h
WholeF
CarcassF
CarcassM
WholeM
GutMidF
GutMidM
SalivF
SalivM
GutHind
GutAnt
GutCaeca
GutPost
Blood3h
BloodNBF
BloodTwoNBF
FatBodyBF
GutMidBF
Mean 2.65 +/− 1.12

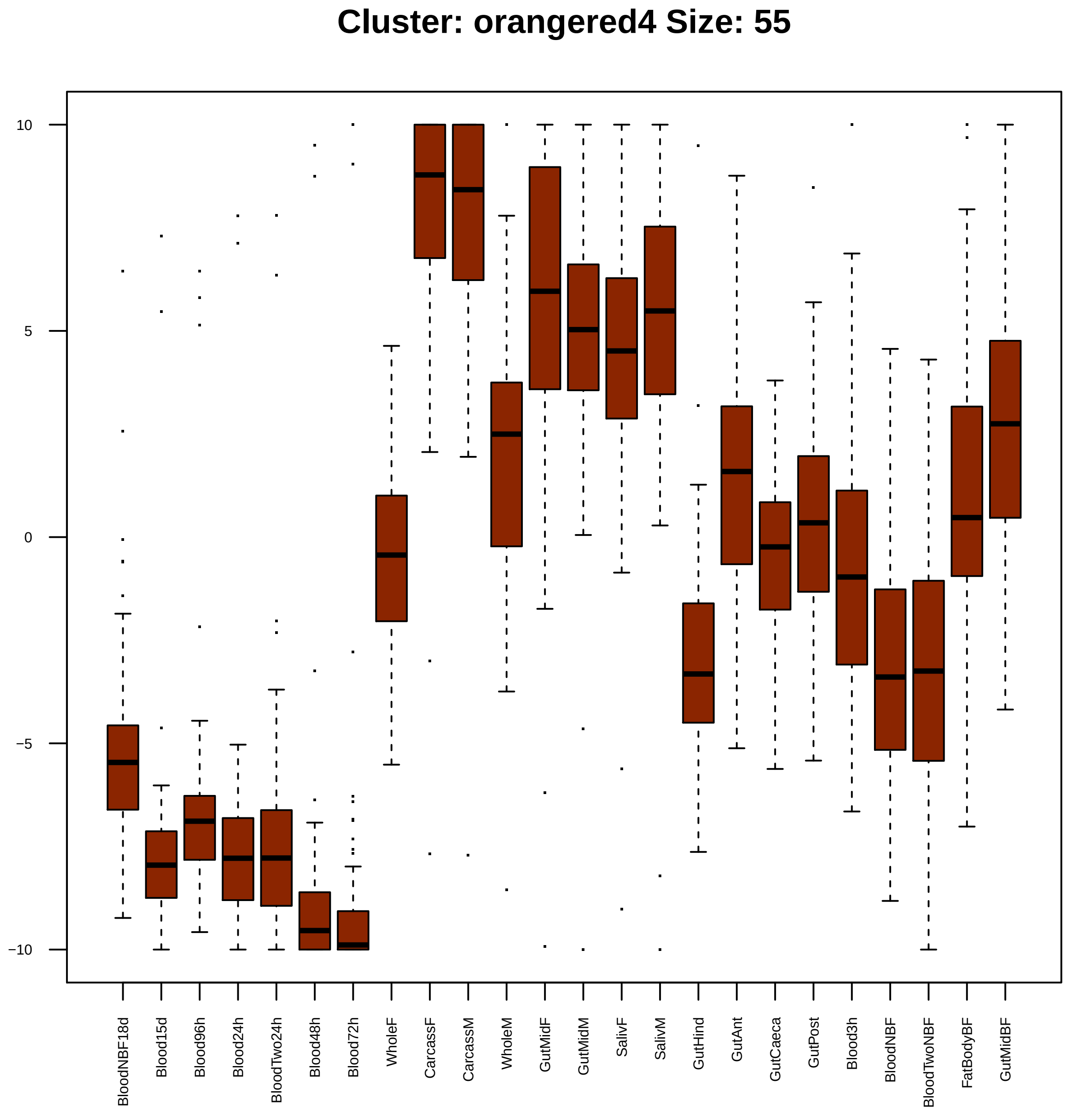
Cluster: orangered4 Size: 55
10
5
0
−5
−10
BloodNBF18d
Blood15d
Blood96h
Blood24h
BloodTwo24h
Blood48h
Blood72h
WholeF
CarcassF
CarcassM
WholeM
GutMidF
GutMidM
SalivF
SalivM
GutHind
GutAnt
GutCaeca
GutPost
Blood3h
BloodNBF
BloodTwoNBF
FatBodyBF
GutMidBF

| | GO.ID | BPCluster: orangered4 Size: 55 | Annotated | Significant | Expected | Rank in ClassicF | Weight01F | ClassicF |
|---|---|---|---|---|---|---|---|---|
| 1 | GO:0007365 | periodic partitioning | 23 | 3 | 0.14 | 1 | 0.00049 | 0.00032 |
| 2 | GO:0035287 | head segmentation | 11 | 2 | 0.07 | 2 | 0.00184 | 0.00184 |
| 4 | GO:0006355 | regulation of transcription, DNA−templat... | 501 | 8 | 2.99 | 5 | 0.01018 | 0.00817 |

| | GO.ID | MFCluster: orangered4 Size: 55 | Annotated | Significant | Expected | Rank in ClassicF | Weight01F | ClassicF |
|---|---|---|---|---|---|---|---|---|
| *1* | GO:0003700 | DNA−binding transcription factor activit... | 282 | 8 | 1.56 | 2 | 0.00071 | 0.00013 |
| *2* | GO:0043565 | sequence−specific DNA binding | 212 | 8 | 1.18 | 1 | 0.00143 | 1.7e−05 |
| *3* | GO:0000976 | transcription regulatory region sequence... | 45 | 3 | 0.25 | 9 | 0.00416 | 0.00191 |
| *12* | GO:0003690 | double−stranded DNA binding | 60 | 4 | 0.33 | 3 | 0.06032 | 0.00032 |
| *24* | GO:0005261 | cation channel activity | 73 | 3 | 0.41 | 16 | 0.21714 | 0.00753 |

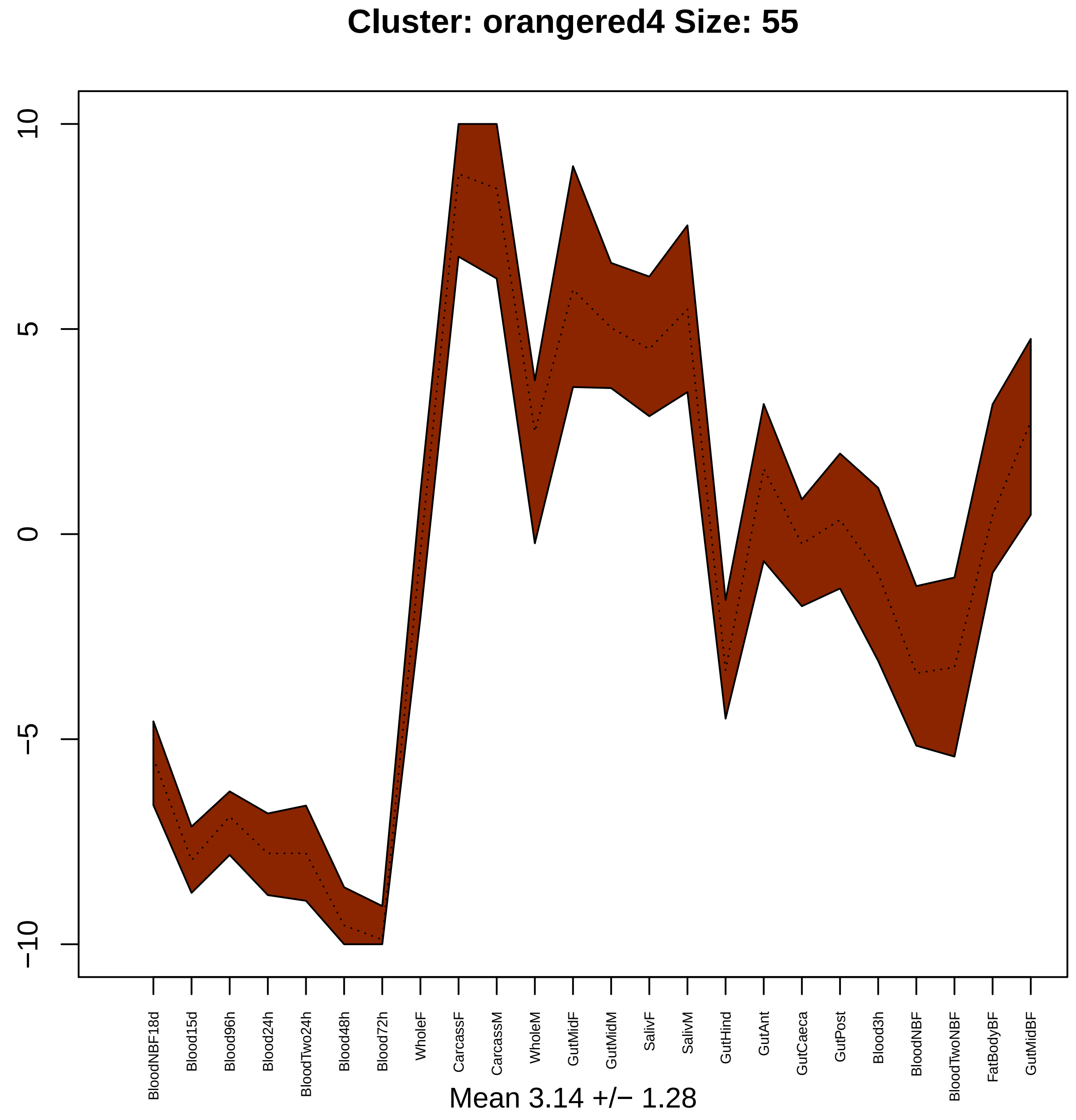
Cluster: orangered4 Size: 55
10
5
0
−5
−10
BloodNBF18d
Blood15d
Blood96h
Blood24h
BloodTwo24h
Blood48h
Blood72h
WholeF
CarcassF
CarcassM
WholeM
GutMidF
GutMidM
SalivF
SalivM
GutHind
GutAnt
GutCaeca
GutPost
Blood3h
BloodNBF
BloodTwoNBF
FatBodyBF
GutMidBF
Mean 3.14 +/− 1.28

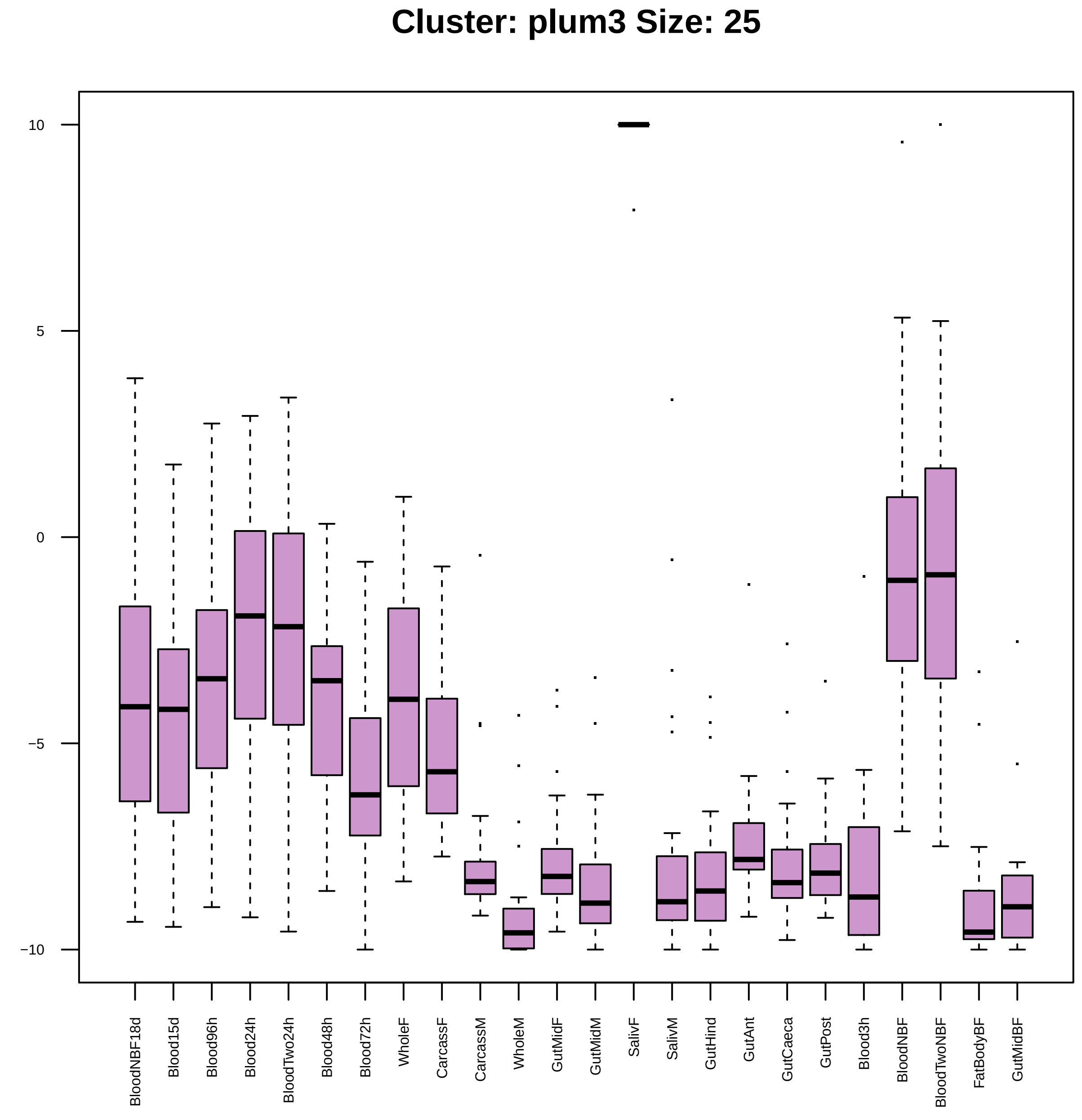
Cluster: plum3 Size: 25
10
5
0
−5
−10
BloodNBF18d
Blood15d
Blood96h
Blood24h
BloodTwo24h
Blood48h
Blood72h
WholeF
CarcassF
CarcassM
WholeM
GutMidF
GutMidM
SalivF
SalivM
GutHind
GutAnt
GutCaeca
GutPost
Blood3h
BloodNBF
BloodTwoNBF
FatBodyBF
GutMidBF

| | GO.ID | BPCluster: plum3 Size: 25 | Annotated | Significant | Expected | Rank in ClassicF | Weight01F | ClassicF |
|---|---|---|---|---|---|---|---|---|
| *1* | GO:0009166 | nucleotide catabolic process | 25 | 2 | 0.02 | 2 | 0.00015 | 0.00015 |
| *12* | GO:0006796 | phosphate−containing compound metabolic ... | 642 | 4 | 0.50 | 9 | 0.09994 | 0.00047 |
| *24* | GO:0044248 | cellular catabolic process | 545 | 3 | 0.43 | 12 | 1.00000 | 0.00545 |
| *28* | GO:0055086 | nucleobase−containing small molecule met... | 193 | 3 | 0.15 | 6 | 1.00000 | 0.00026 |
| *30* | GO:0006753 | nucleoside phosphate metabolic process | 171 | 2 | 0.13 | 14 | 1.00000 | 0.00678 |

| | GO.ID | MFCluster: plum3 Size: 25 | Annotated | Significant | Expected | Rank in ClassicF | Weight01F | ClassicF |
|---|---|---|---|---|---|---|---|---|
| 7 | GO:0016788 | hydrolase activity, acting on ester bond... | 269 | 3 | 0.37 | 2 | 0.114 | 0.0051 |

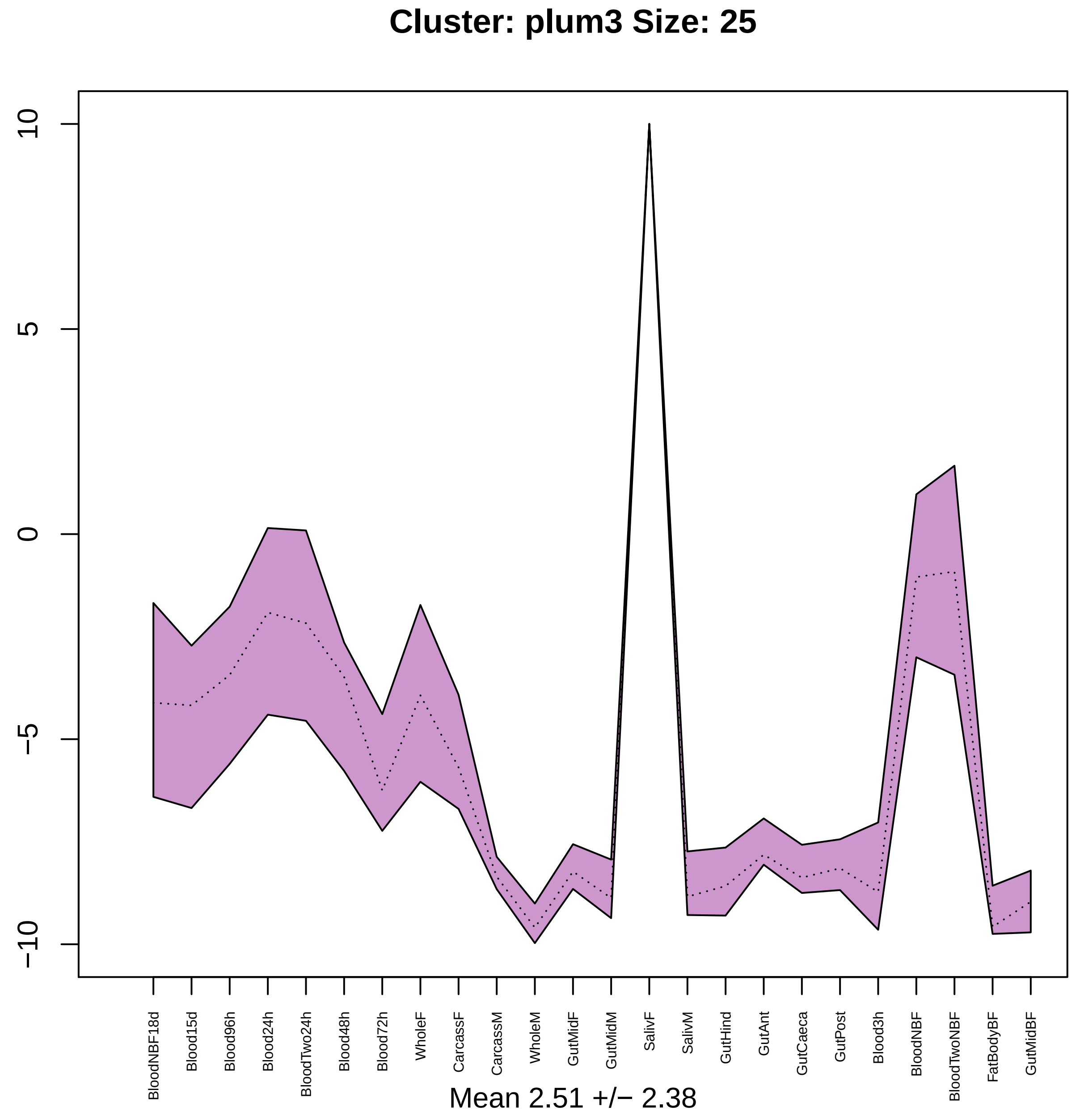

Cluster: plum3 Size: 25
10
5
0
−5
−10
BloodNBF18d
Blood15d
Blood96h
Blood24h
BloodTwo24h
Blood48h
Blood72h
WholeF
CarcassF
CarcassM
WholeM
GutMidF
GutMidM
SalivF
SalivM
GutHind
GutAnt
GutCaeca
GutPost
Blood3h
BloodNBF
BloodTwoNBF
FatBodyBF
GutMidBF
Mean 2.51 +/− 2.38

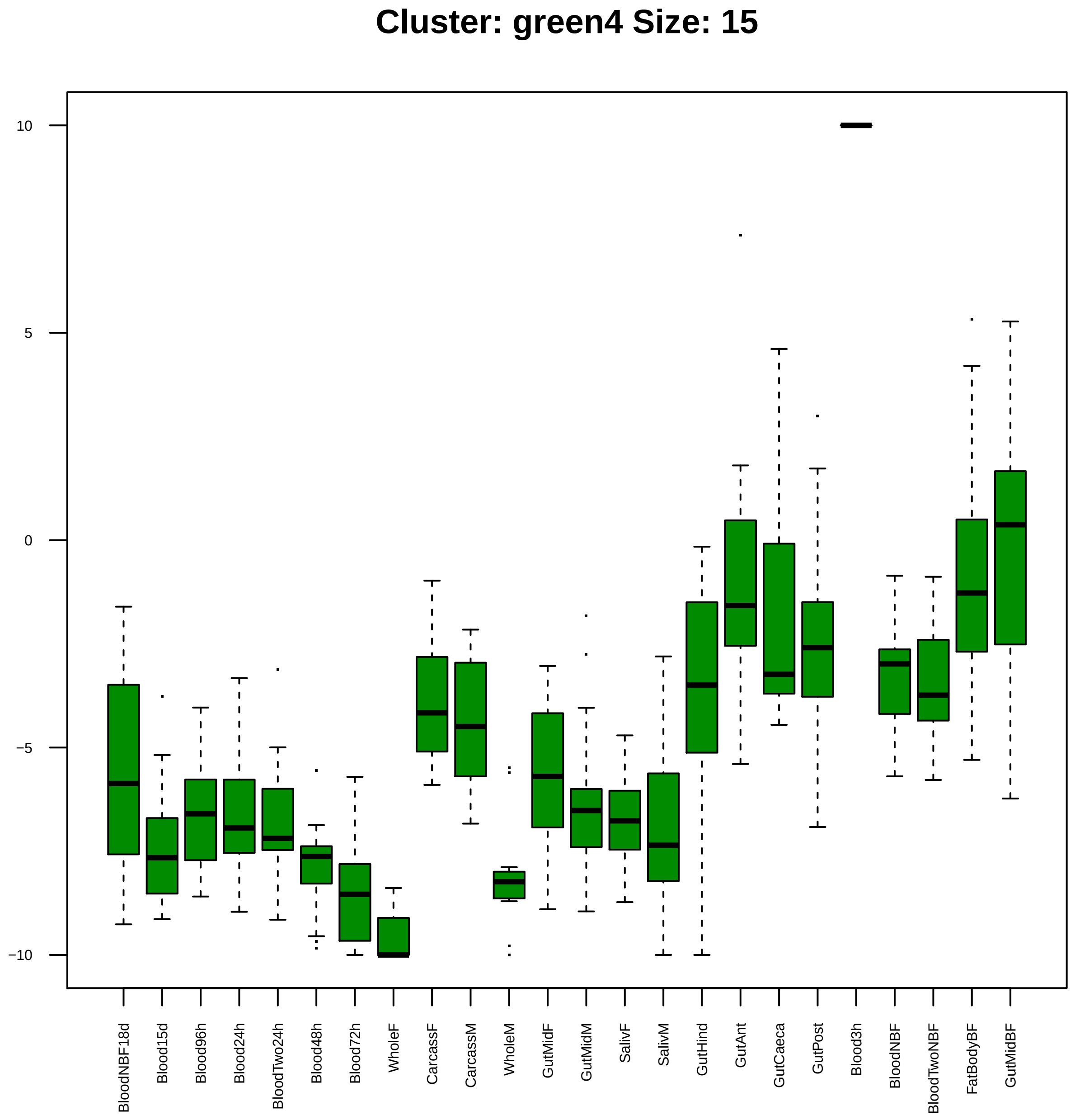
Cluster: green4 Size: 15
10
5
0
−5
−10
BloodNBF18d
Blood15d
Blood96h
Blood24h
BloodTwo24h
Blood48h
Blood72h
WholeF
CarcassF
CarcassM
WholeM
GutMidF
GutMidM
SalivF
SalivM
GutHind
GutAnt
GutCaeca
GutPost
Blood3h
BloodNBF
BloodTwoNBF
FatBodyBF
GutMidBF

|  | GO.ID | MFCluster: green4 Size: 15 | Annotated | Significant | Expected | Rank in ClassicF | Weight01F | ClassicF |
|---|---|---|---|---|---|---|---|---|
| *1* | GO:0042302 | structural constituent of cuticle | 99 | 3 | 0.10 | 1 | 0.0017 | 8.4e−05 |
| *10* | GO:0005198 | structural molecule activity | 318 | 3 | 0.31 | 2 | 1.0000 | 0.0026 |

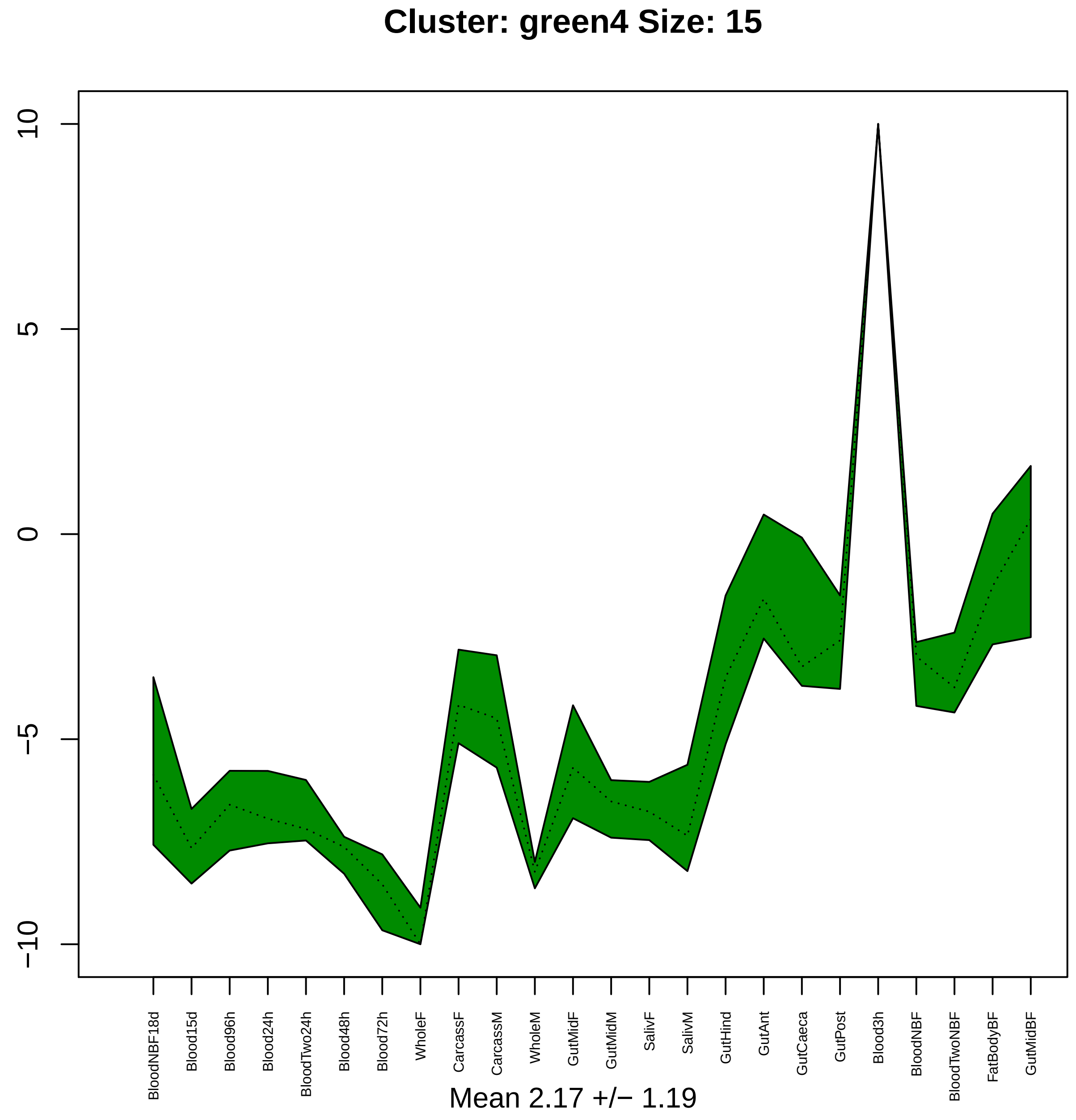

Cluster: green4 Size: 15
10
5
0
−5
−10
BloodNBF18d
Blood15d
Blood96h
Blood24h
BloodTwo24h
Blood48h
Blood72h
WholeF
CarcassF
CarcassM
WholeM
GutMidF
GutMidM
SalivF
SalivM
GutHind
GutAnt
GutCaeca
GutPost
Blood3h
BloodNBF
BloodTwoNBF
FatBodyBF
GutMidBF
Mean 2.17 +/− 1.19

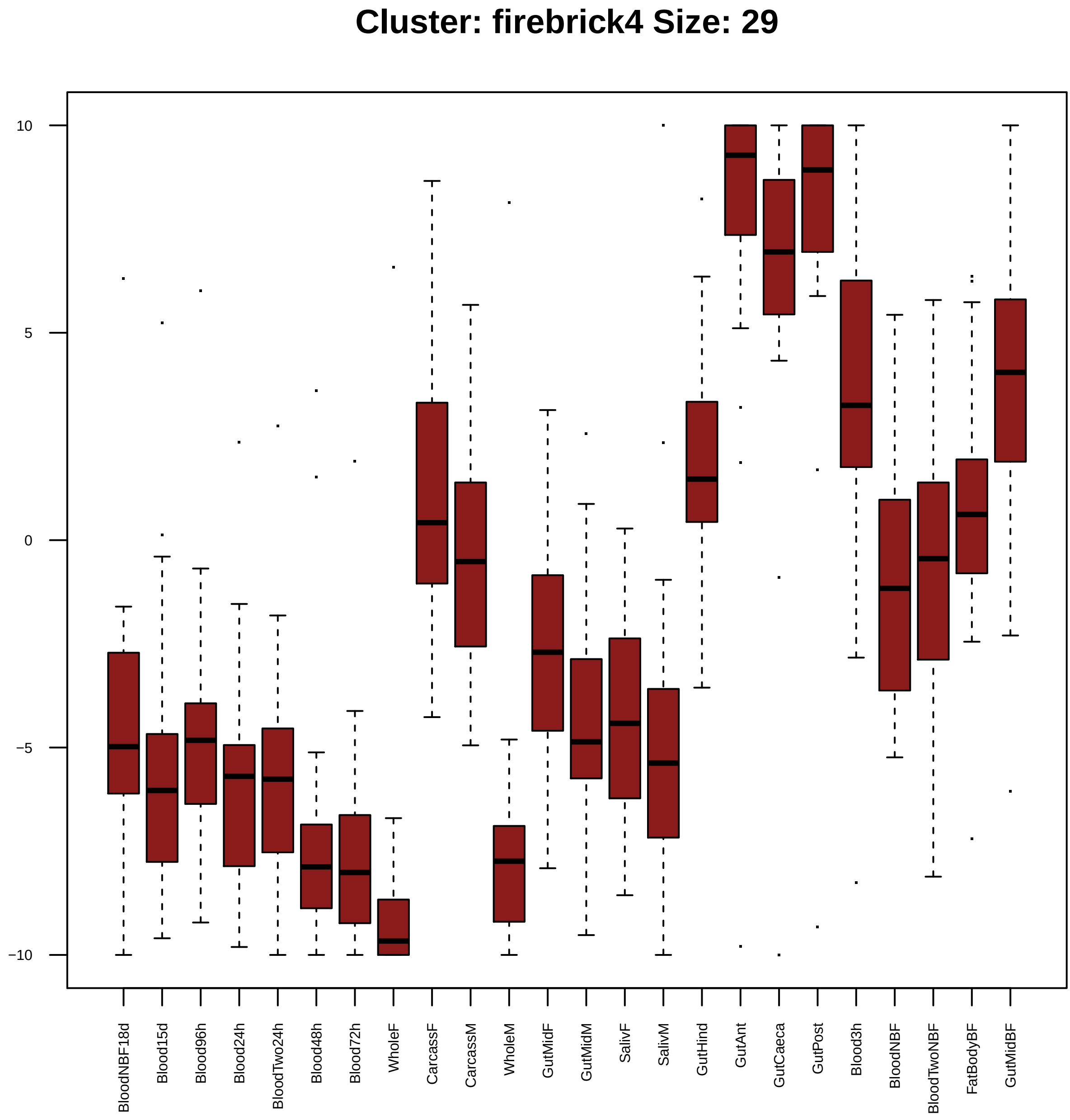

Cluster: firebrick4 Size: 29
10
5
0
−5
−10
BloodNBF18d
Blood15d
Blood96h
Blood24h
BloodTwo24h
Blood48h
Blood72h
WholeF
CarcassF
CarcassM
WholeM
GutMidF
GutMidM
SalivF
SalivM
GutHind
GutAnt
GutCaeca
GutPost
Blood3h
BloodNBF
BloodTwoNBF
FatBodyBF
GutMidBF

| | GO.ID | BPCluster: firebrick4 Size: 29 | Annotated | Significant | Expected | Rank in ClassicF | Weight01F | ClassicF |
|---|---|---|---|---|---|---|---|---|
| *1* | GO:0007186 | G protein−coupled receptor signaling pat... | 228 | 4 | 0.43 | 1 | 0.004 | 0.00063 |

| | GO.ID | MFCluster: firebrick4 Size: 29 | Annotated | Significant | Expected | Rank in ClassicF | Weight01F | ClassicF |
|---|---|---|---|---|---|---|---|---|
| *1* | GO:0004930 | G protein−coupled receptor activity | 171 | 4 | 0.43 | 1 | 0.0046 | 0.00072 |
| *29* | GO:0038023 | signaling receptor activity | 282 | 4 | 0.70 | 3 | 1.0000 | 0.00454 |

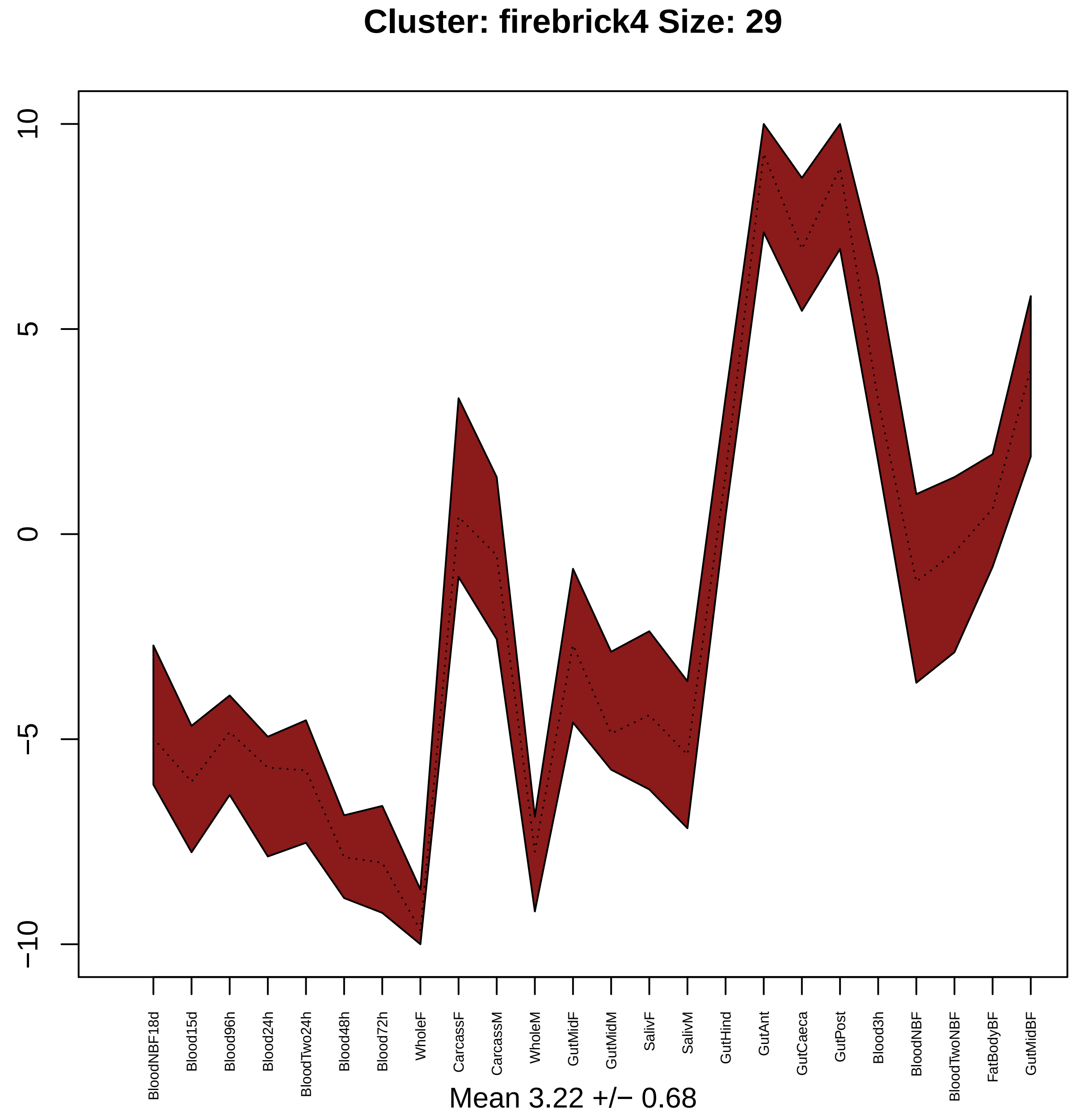

Cluster: firebrick4 Size: 29
10
5
0
−5
−10
BloodNBF18d
Blood15d
Blood96h
Blood24h
BloodTwo24h
Blood48h
Blood72h
WholeF
CarcassF
CarcassM
WholeM
GutMidF
GutMidM
SalivF
SalivM
GutHind
GutAnt
GutCaeca
GutPost
Blood3h
BloodNBF
BloodTwoNBF
FatBodyBF
GutMidBF
Mean 3.22 +/− 0.68

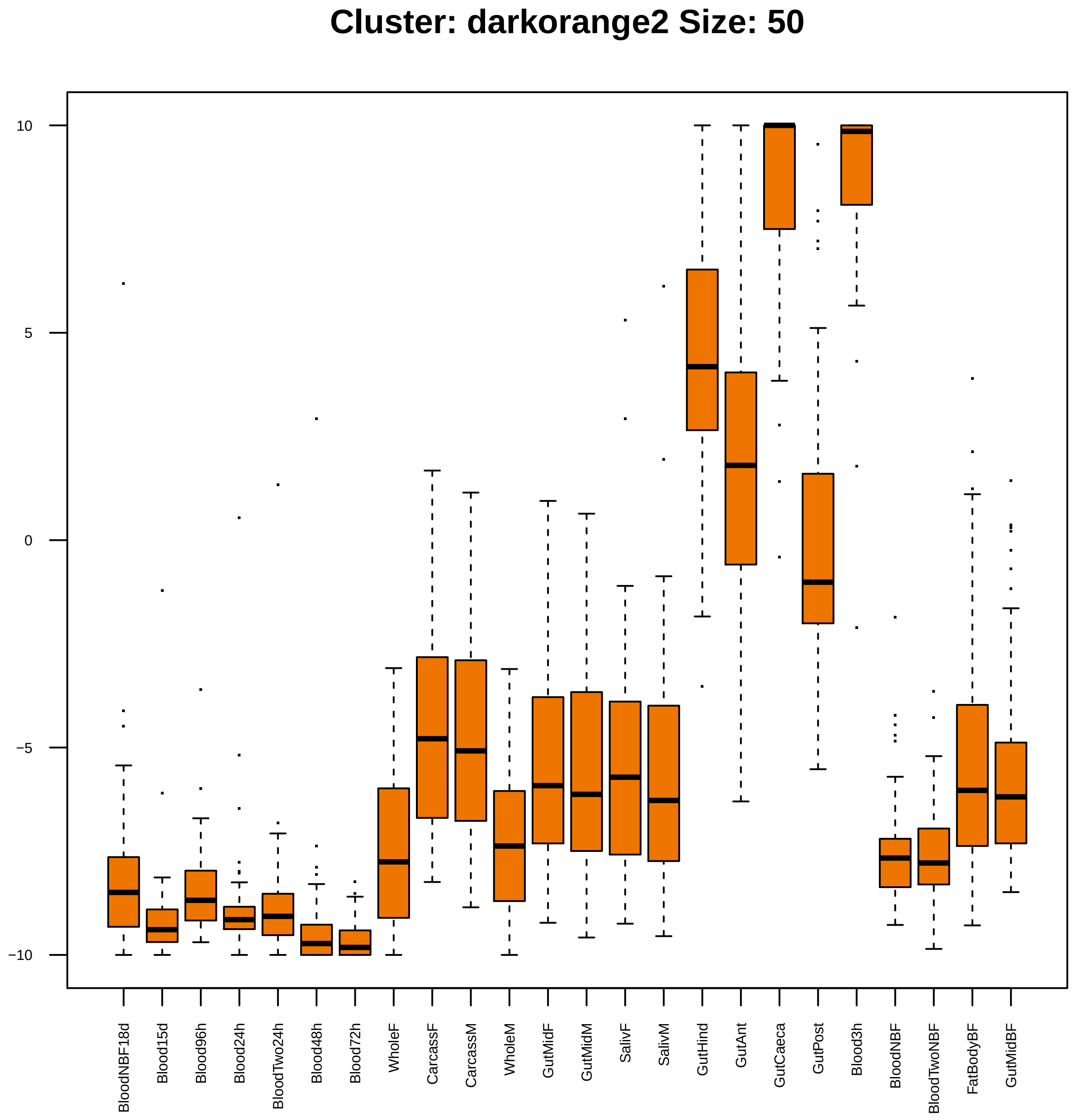

Cluster: darkorange2 Size: 50
10
5
0
−5
−10
BloodNBF18d
Blood15d
Blood96h
Blood24h
BloodTwo24h
Blood48h
Blood72h
WholeF
CarcassF
CarcassM
WholeM
GutMidF
GutMidM
SalivF
SalivM
GutHind
GutAnt
GutCaeca
GutPost
Blood3h
BloodNBF
BloodTwoNBF
FatBodyBF
GutMidBF

| | GO.ID | BPCluster: darkorange2 Size: 50 | Annotated | Significant | Expected | Rank in ClassicF | Weight01F | ClassicF |
|---|---|---|---|---|---|---|---|---|
| *1* | GO:0006979 | response to oxidative stress | 51 | 3 | 0.18 | 1 | 0.00076 | 0.00076 |

| | GO.ID | MFCluster: darkorange2 Size: 50 | Annotated | Significant | Expected | Rank in ClassicF | Weight01F | ClassicF |
|---|---|---|---|---|---|---|---|---|
| *1* | GO:0004601 | peroxidase activity | 26 | 3 | 0.1 | 1 | 0.00012 | 0.00012 |

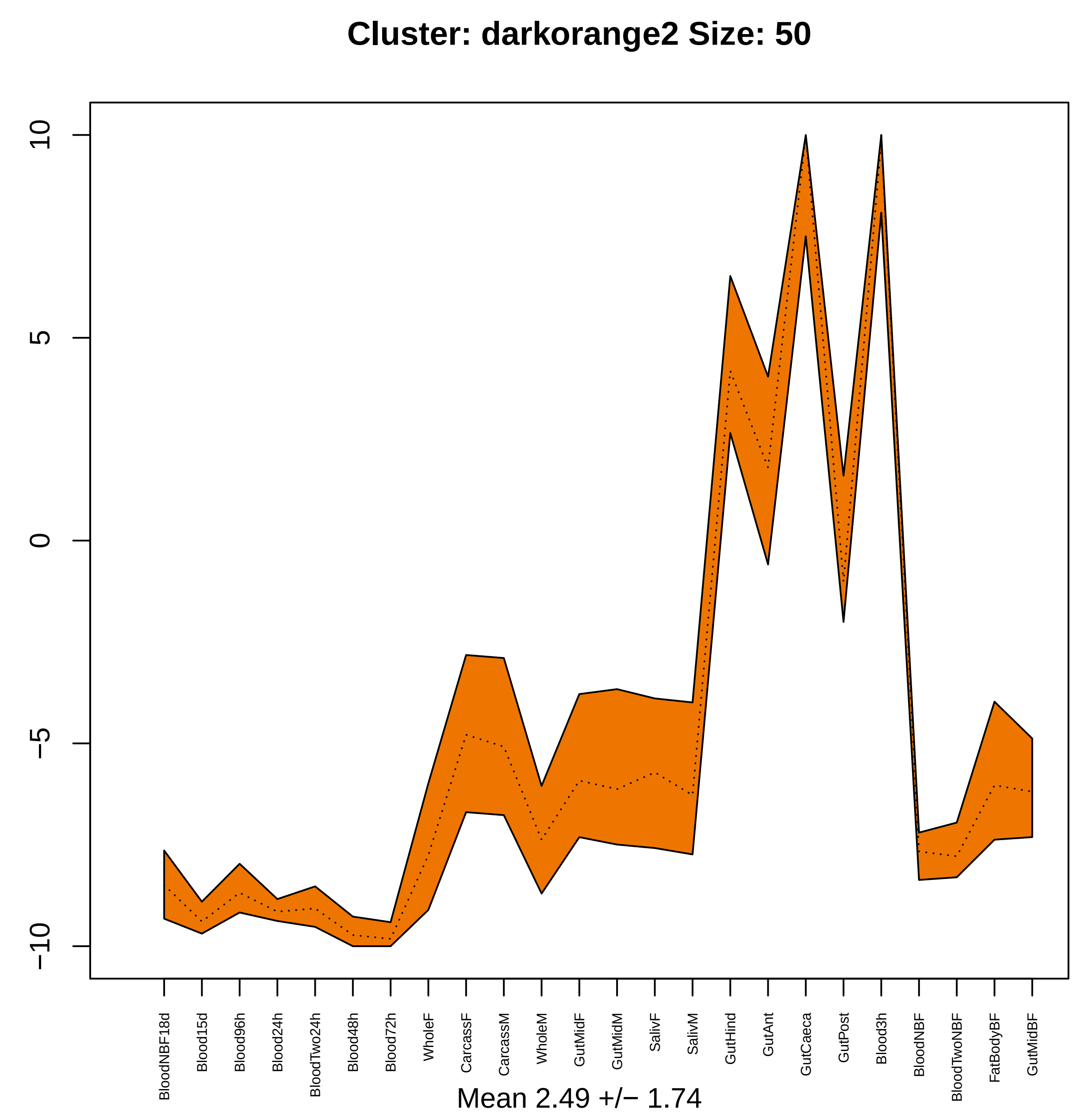
Cluster: darkorange2 Size: 50
10
5
0
−5
−10
BloodNBF18d
Blood15d
Blood96h
Blood24h
BloodTwo24h
Blood48h
Blood72h
WholeF
CarcassF
CarcassM
WholeM
GutMidF
GutMidM
SalivF
SalivM
GutHind
GutAnt
GutCaeca
GutPost
Blood3h
BloodNBF
BloodTwoNBF
FatBodyBF
GutMidBF
Mean 2.49 +/− 1.74

# Cluster: lightskyblue4 Size: 12

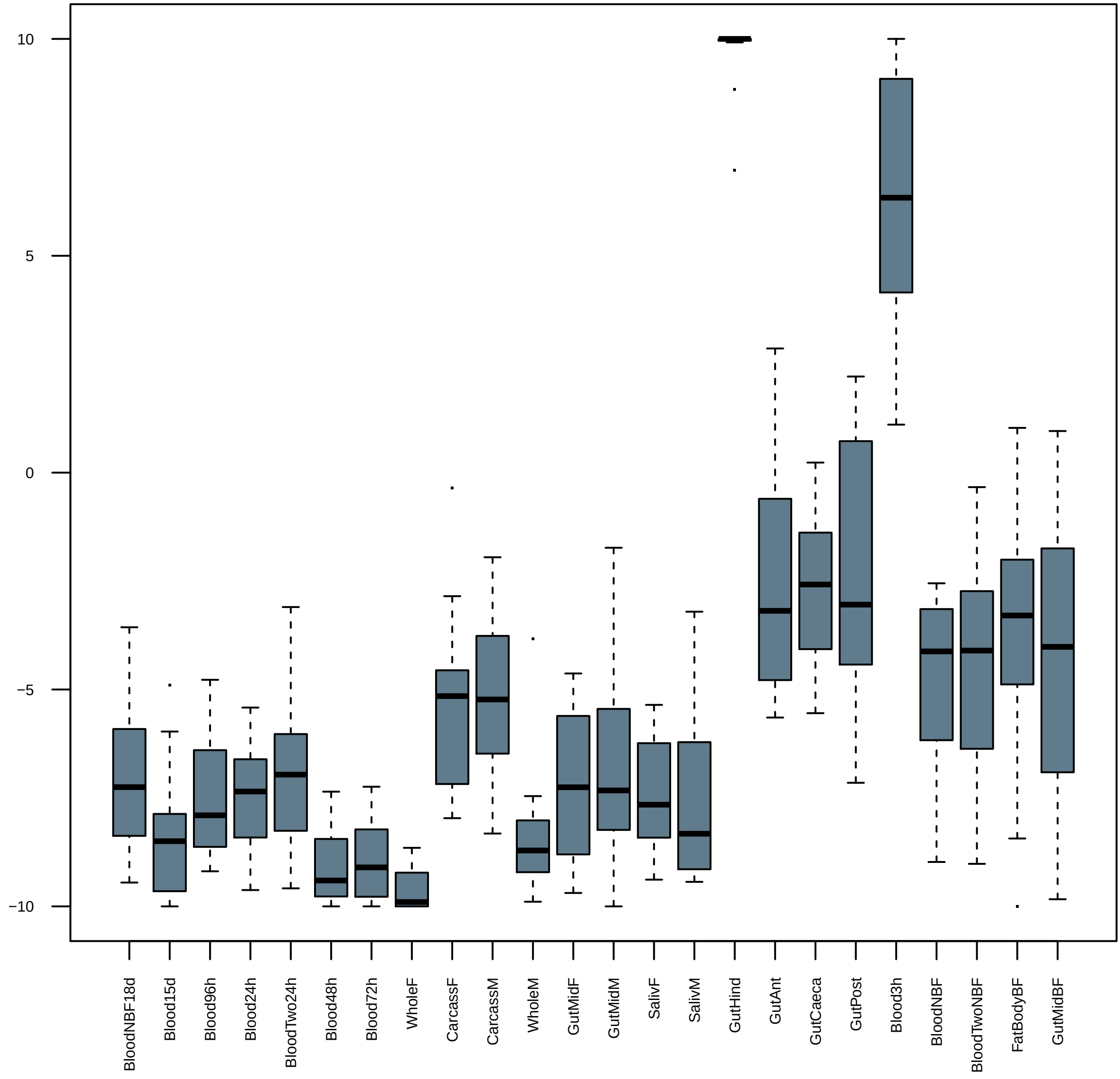

| | GO.ID | MFCluster: lightskyblue4 Size: 12 | Annotated | Significant | Expected | Rank in ClassicF | Weight01F | ClassicF |
|---|---|---|---|---|---|---|---|---|
| *1* | GO:0042302 | structural constituent of cuticle | 99 | 2 | 0.07 | 1 | 0.0018 | 0.0018 |

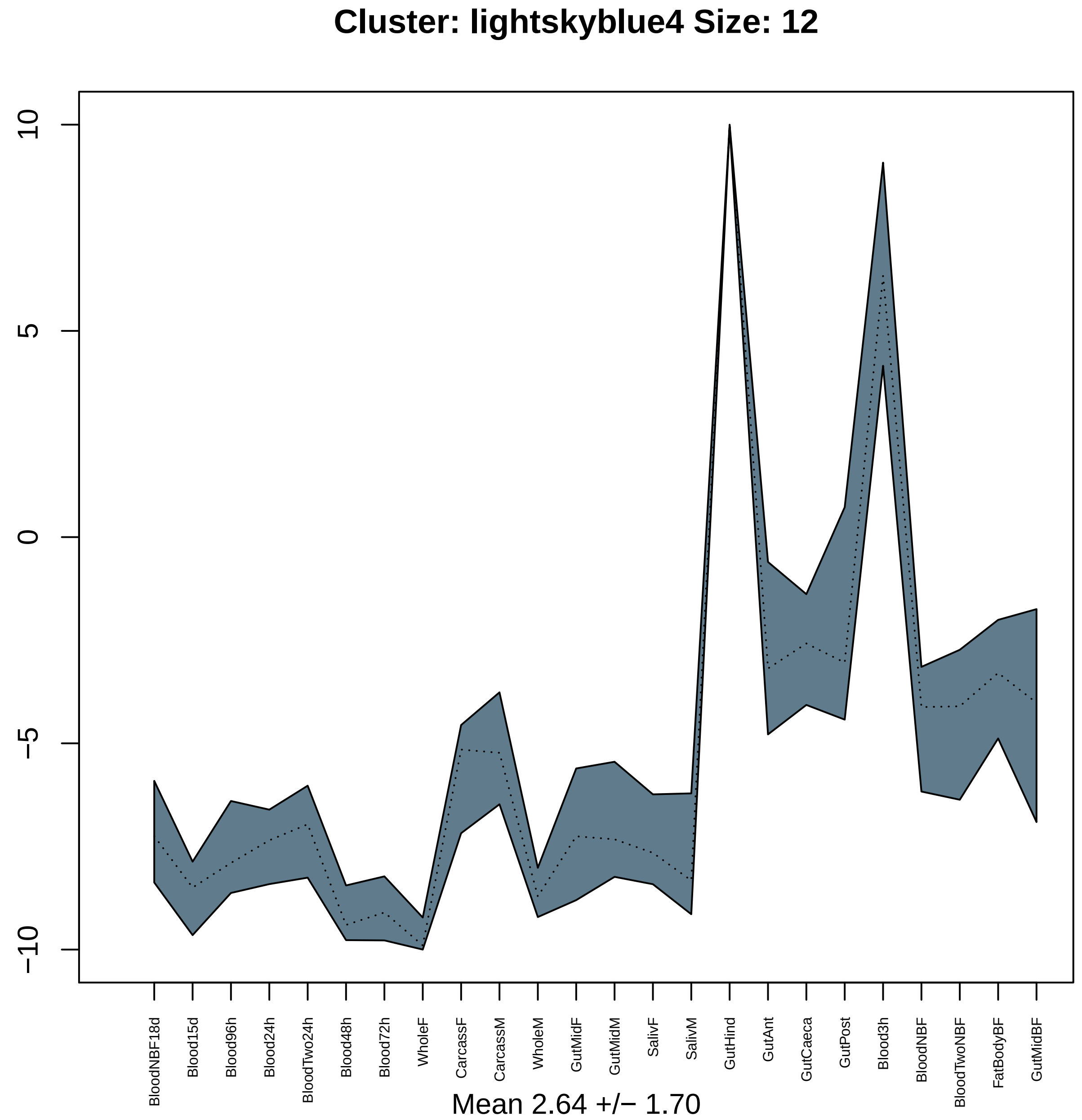

Cluster: lightskyblue4 Size: 12
10
5
0
−5
−10
BloodNBF18d
Blood15d
Blood96h
Blood24h
BloodTwo24h
Blood48h
Blood72h
WholeF
CarcassF
CarcassM
WholeM
GutMidF
GutMidM
SalivF
SalivM
GutHind
GutAnt
GutCaeca
GutPost
Blood3h
BloodNBF
BloodTwoNBF
FatBodyBF
GutMidBF
Mean 2.64 +/− 1.70

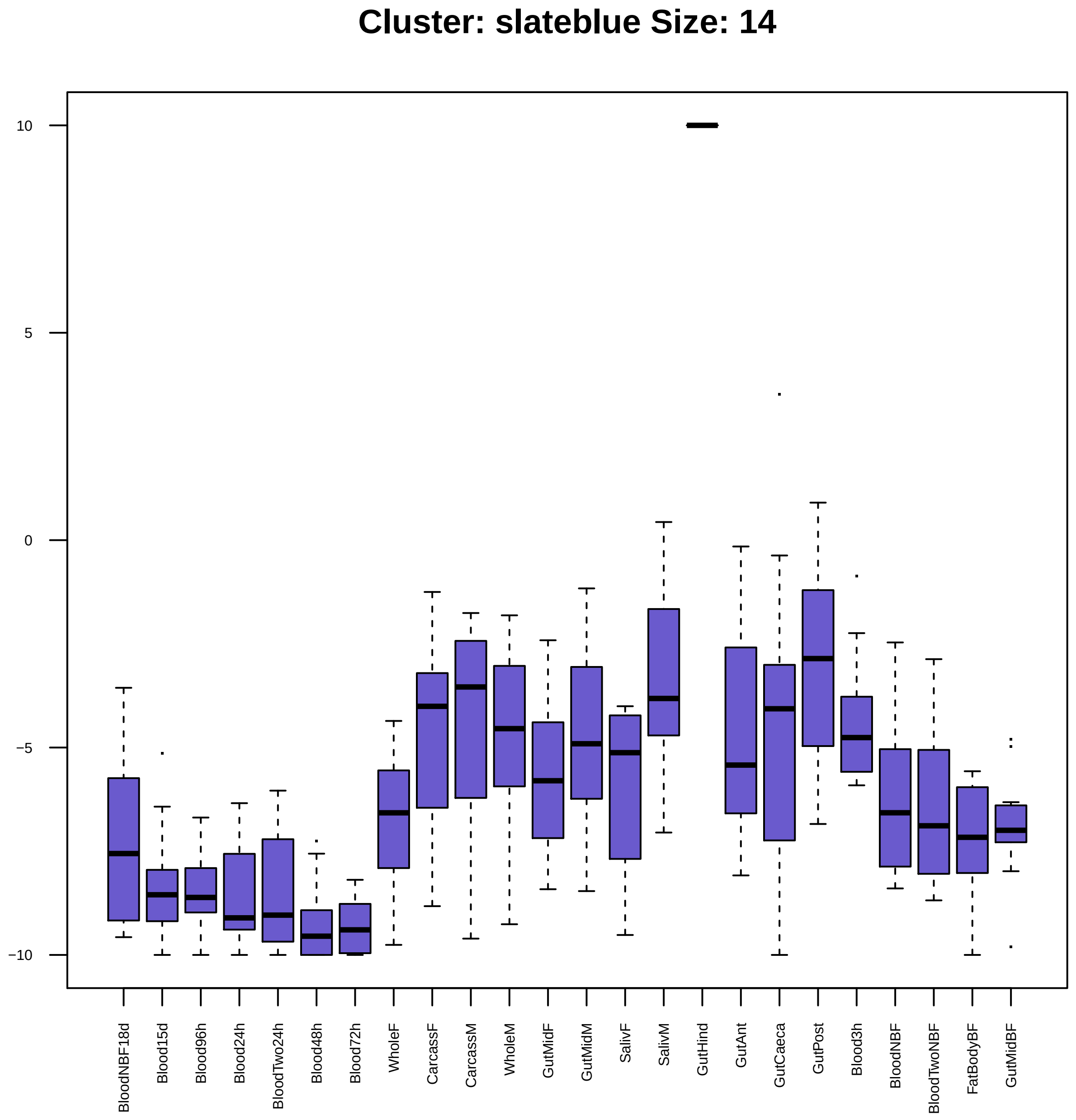

Cluster: slateblue Size: 14
10
5
0
−5
−10
BloodNBF18d
Blood15d
Blood96h
Blood24h
BloodTwo24h
Blood48h
Blood72h
WholeF
CarcassF
CarcassM
WholeM
GutMidF
GutMidM
SalivF
SalivM
GutHind
GutAnt
GutCaeca
GutPost
Blood3h
BloodNBF
BloodTwoNBF
FatBodyBF
GutMidBF

| | GO.ID | MFCluster: slateblue Size: 14 | Annotated | Significant | Expected | Rank in ClassicF | Weight01F | ClassicF |
|---|---|---|---|---|---|---|---|---|
| *1* | GO:0004252 | serine−type endopeptidase activity | 252 | 3 | 0.35 | 1 | 0.0042 | 0.0042 |
| *8* | GO:0017171 | serine hydrolase activity | 279 | 3 | 0.39 | 3 | 1.0000 | 0.0056 |
| *19* | GO:0008236 | serine−type peptidase activity | 278 | 3 | 0.39 | 2 | 1.0000 | 0.0056 |

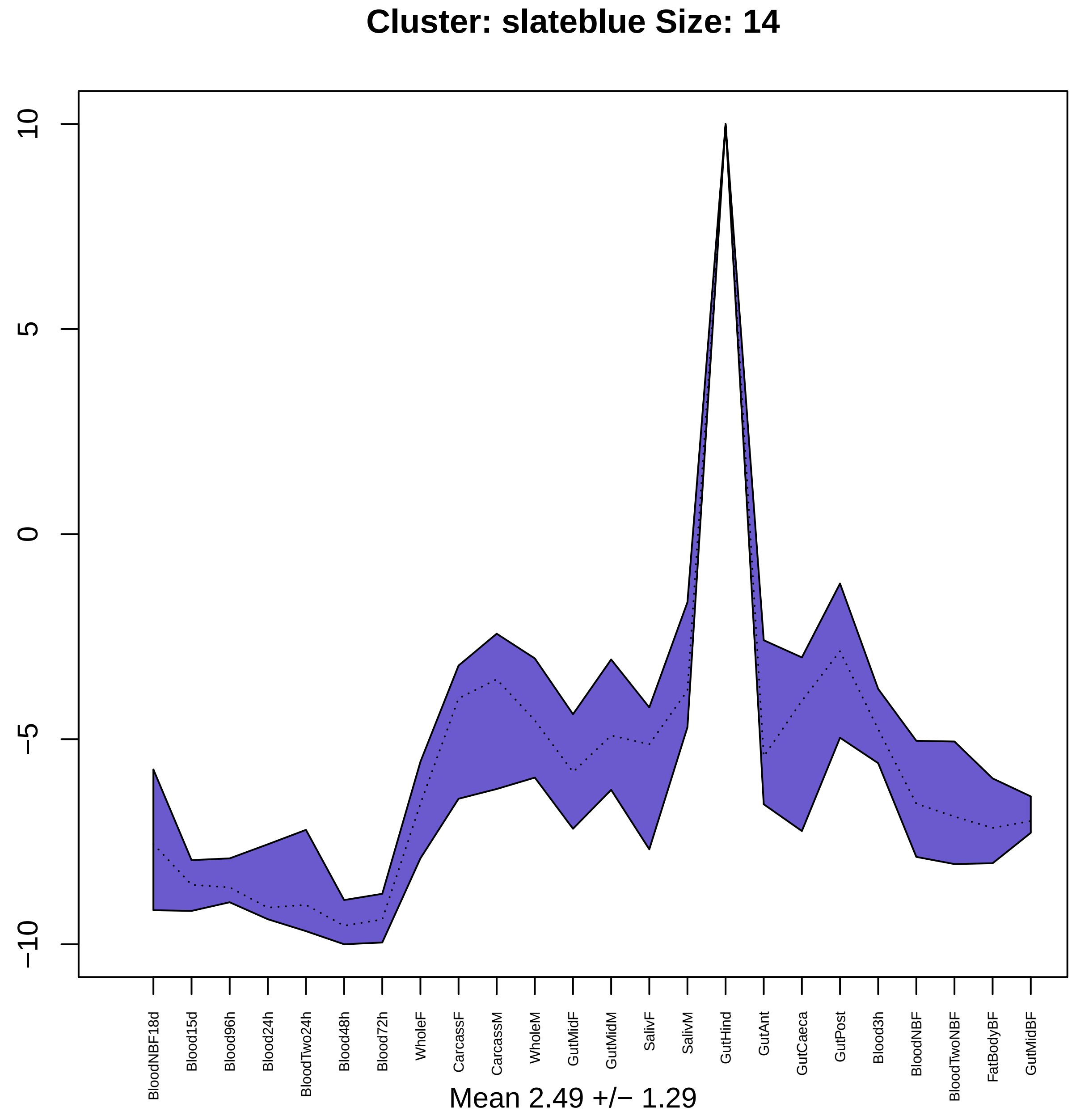
Cluster: slateblue Size: 14
10
5
0
−5
−10
BloodNBF18d
Blood15d
Blood96h
Blood24h
BloodTwo24h
Blood48h
Blood72h
WholeF
CarcassF
CarcassM
WholeM
GutMidF
GutMidM
SalivF
SalivM
GutHind
GutAnt
GutCaeca
GutPost
Blood3h
BloodNBF
BloodTwoNBF
FatBodyBF
GutMidBF
Mean 2.49 +/− 1.29

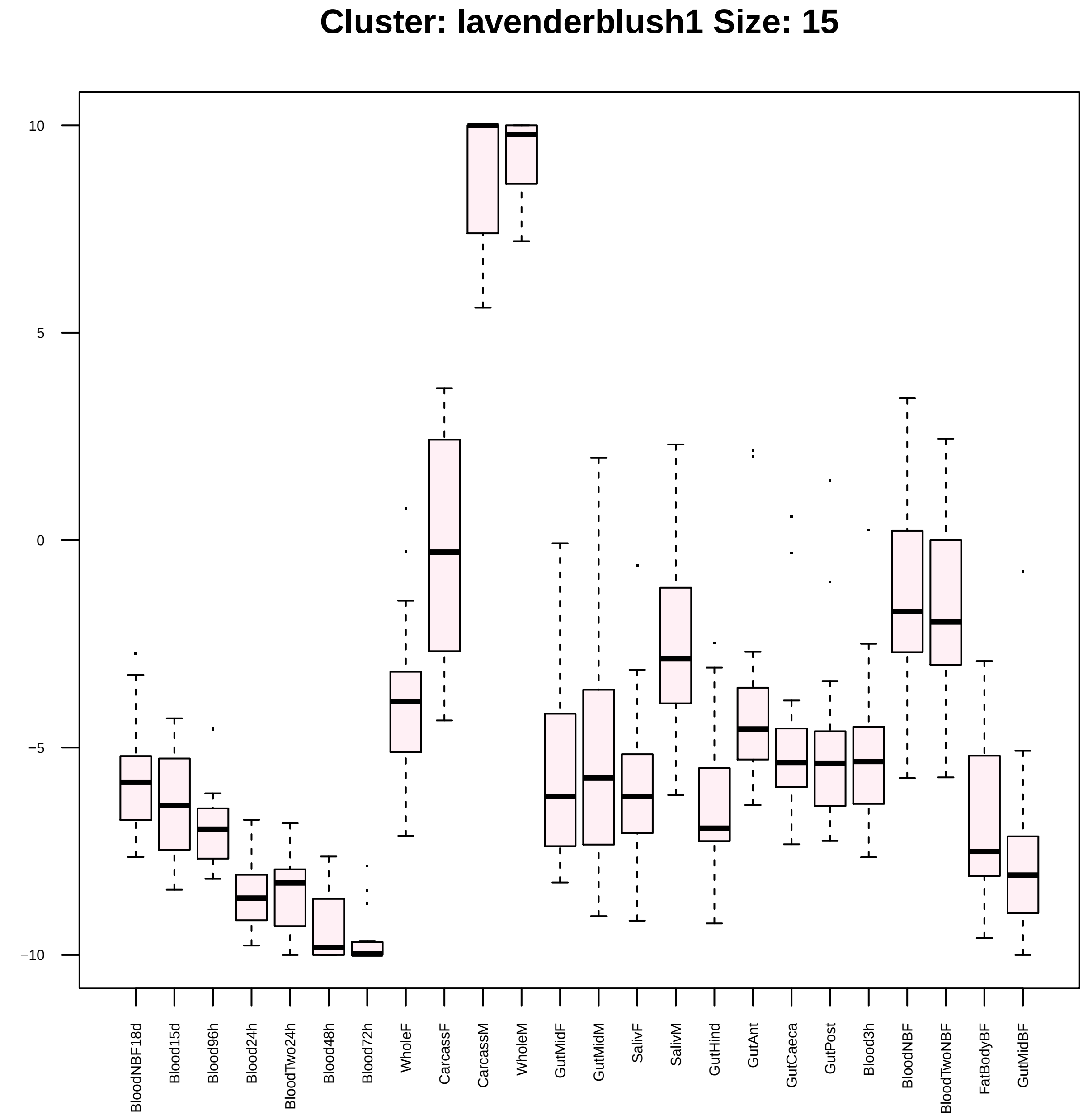

Cluster: lavenderblush1 Size: 15
10
5
0
−5
−10
BloodNBF18d
Blood15d
Blood96h
Blood24h
BloodTwo24h
Blood48h
Blood72h
WholeF
CarcassF
CarcassM
WholeM
GutMidF
GutMidM
SalivF
SalivM
GutHind
GutAnt
GutCaeca
GutPost
Blood3h
BloodNBF
BloodTwoNBF
FatBodyBF
GutMidBF

| | GO.ID | BPCluster: lavenderblush1 Size: 15 | Annotated | Significant | Expected | Rank in ClassicF | Weight01F | ClassicF |
|---|---|---|---|---|---|---|---|---|
| *1* | GO:0007186 | G protein−coupled receptor signaling pat... | 228 | 3 | 0.25 | 1 | 0.0014 | 0.0014 |

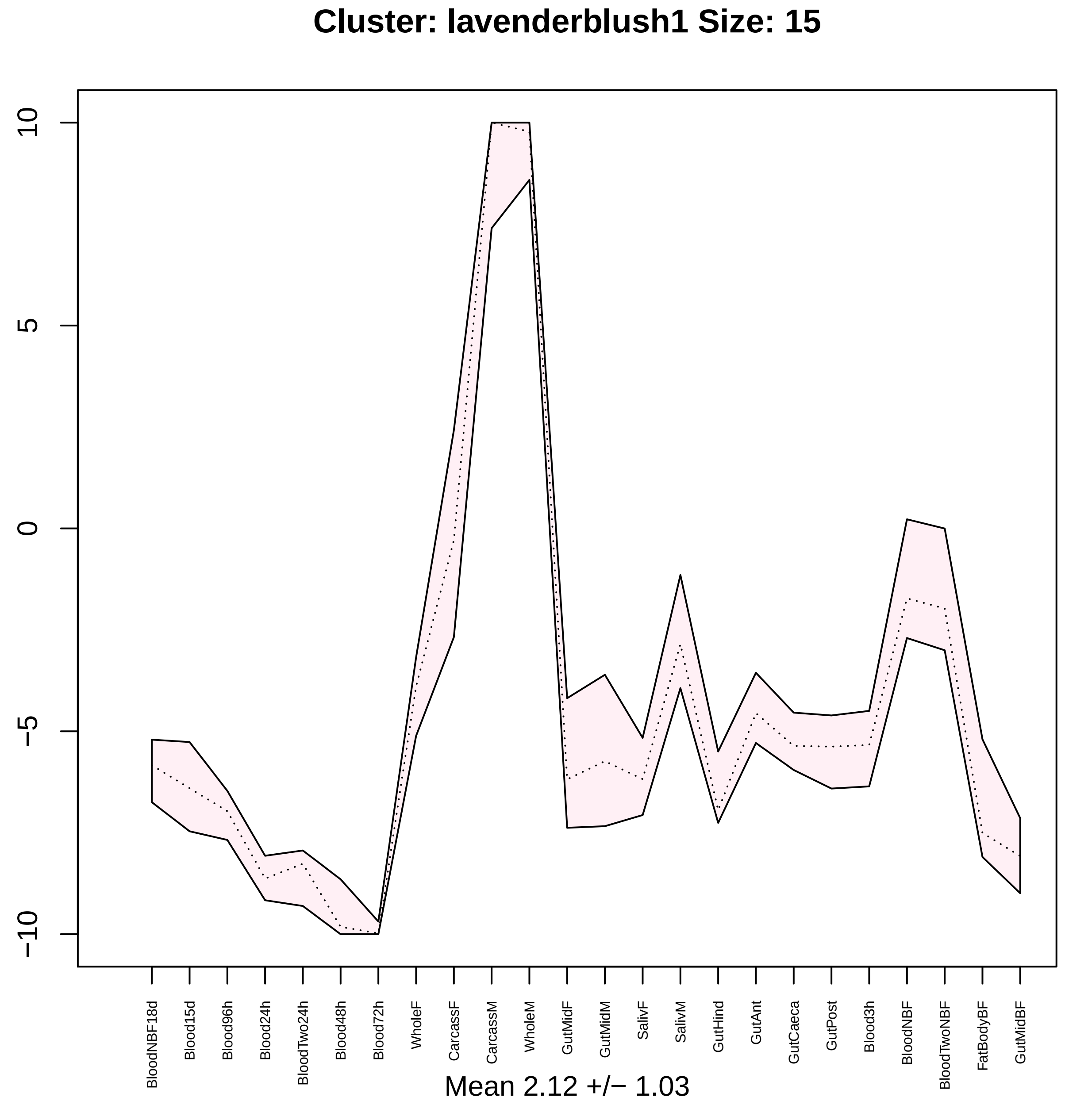
Cluster: lavenderblush1 Size: 15
10
5
0
−5
−10
BloodNBF18d
Blood15d
Blood96h
Blood24h
BloodTwo24h
Blood48h
Blood72h
WholeF
CarcassF
CarcassM
WholeM
GutMidF
GutMidM
SalivF
SalivM
GutHind
GutAnt
GutCaeca
GutPost
Blood3h
BloodNBF
BloodTwoNBF
FatBodyBF
GutMidBF
Mean 2.12 +/− 1.03

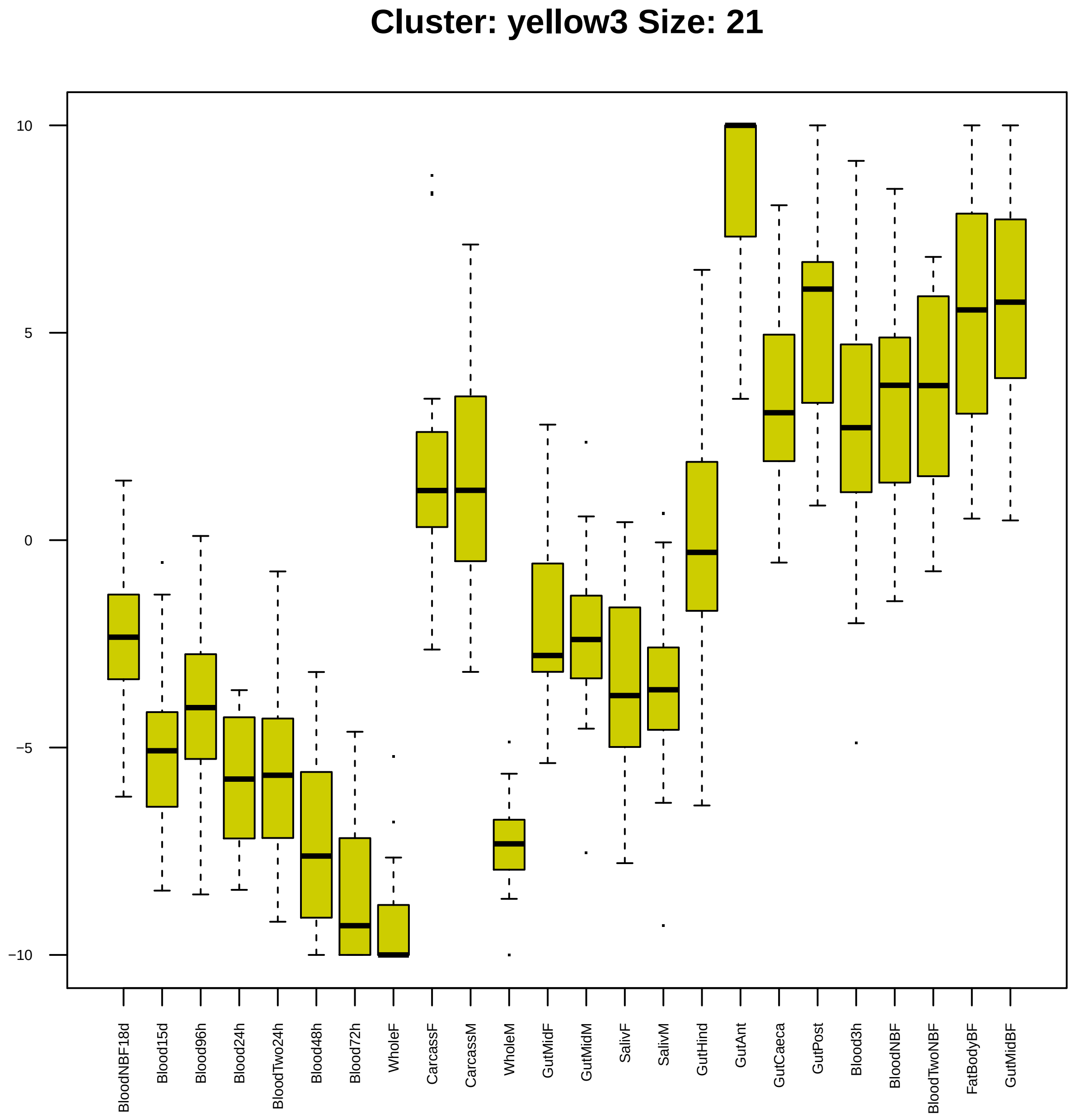

Cluster: yellow3 Size: 21
10
5
0
−5
−10
BloodNBF18d
Blood15d
Blood96h
Blood24h
BloodTwo24h
Blood48h
Blood72h
WholeF
CarcassF
CarcassM
WholeM
GutMidF
GutMidM
SalivF
SalivM
GutHind
GutAnt
GutCaeca
GutPost
Blood3h
BloodNBF
BloodTwoNBF
FatBodyBF
GutMidBF

| | GO.ID | BPCluster: yellow3 Size: 21 | Annotated | Significant | Expected | Rank in ClassicF | Weight01F | ClassicF |
|---|---|---|---|---|---|---|---|---|
| *1* | GO:0046068 | cGMP metabolic process | 11 | 2 | 0.02 | 1 | 0.00012 | 0.00012 |
| *2* | GO:0052652 | cyclic purine nucleotide metabolic proce... | 14 | 2 | 0.02 | 2 | 0.00020 | 0.00020 |
| *3* | GO:0009152 | purine ribonucleotide biosynthetic proce... | 67 | 2 | 0.11 | 10 | 0.00463 | 0.00463 |
| *4* | GO:0006468 | protein phosphorylation | 271 | 3 | 0.42 | 15 | 0.00729 | 0.00729 |

| | GO.ID | MFCluster: yellow3 Size: 21 | Annotated | Significant | Expected | Rank in ClassicF | Weight01F | ClassicF |
|---|---|---|---|---|---|---|---|---|
| *1* | GO:0004383 | guanylate cyclase activity | 11 | 2 | 0.03 | 1 | 0.00032 | 0.00032 |
| *2* | GO:0001653 | peptide receptor activity | 26 | 2 | 0.06 | 4 | 0.00473 | 0.00185 |
| *30* | GO:0016849 | phosphorus−oxygen lyase activity | 23 | 2 | 0.06 | 3 | 1.00000 | 0.00144 |

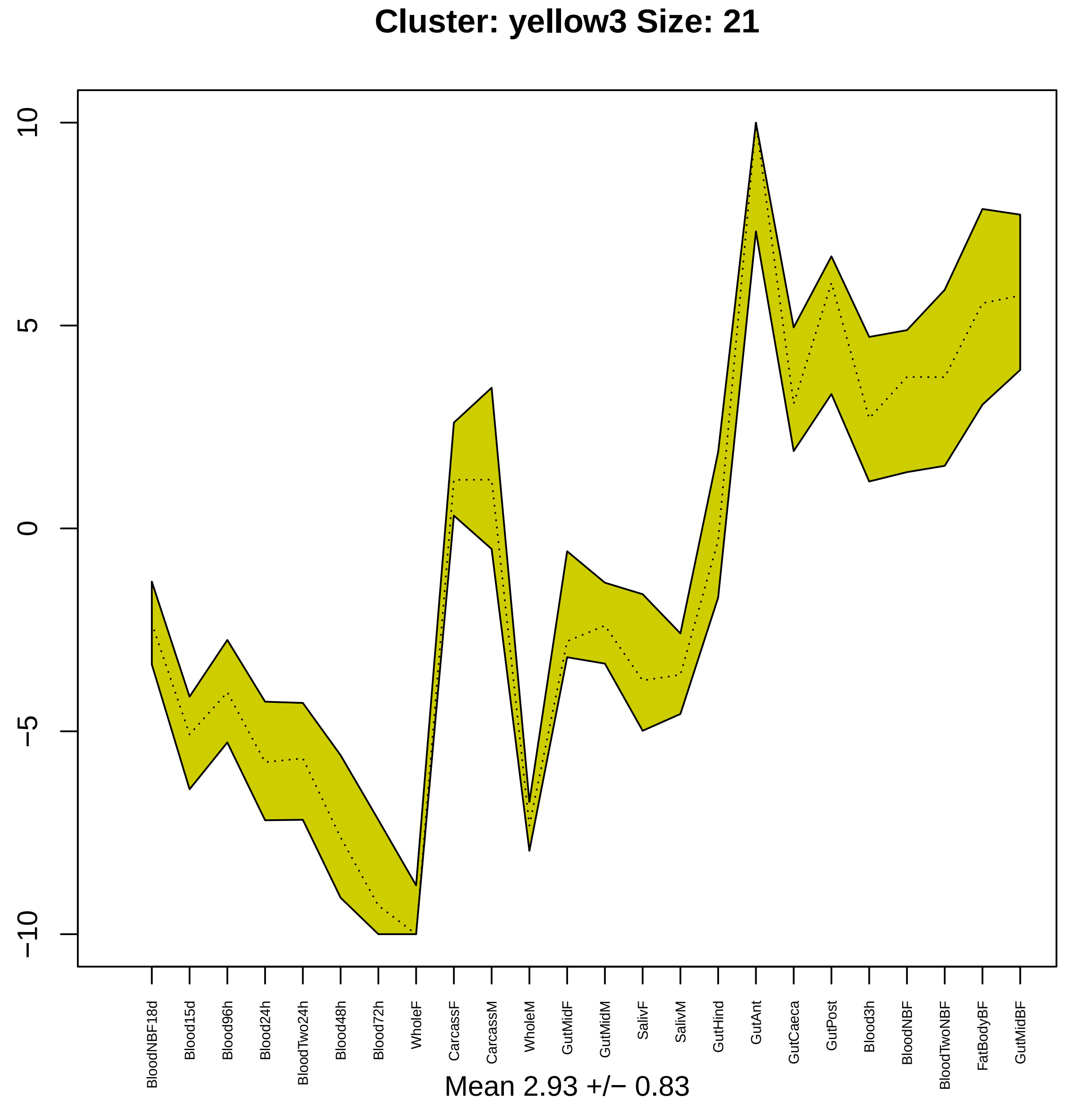
Cluster: yellow3 Size: 21
10
5
0
−5
−10
BloodNBF18d
Blood15d
Blood96h
Blood24h
BloodTwo24h
Blood48h
Blood72h
WholeF
CarcassF
CarcassM
WholeM
GutMidF
GutMidM
SalivF
SalivM
GutHind
GutAnt
GutCaeca
GutPost
Blood3h
BloodNBF
BloodTwoNBF
FatBodyBF
GutMidBF
Mean 2.93 +/− 0.83

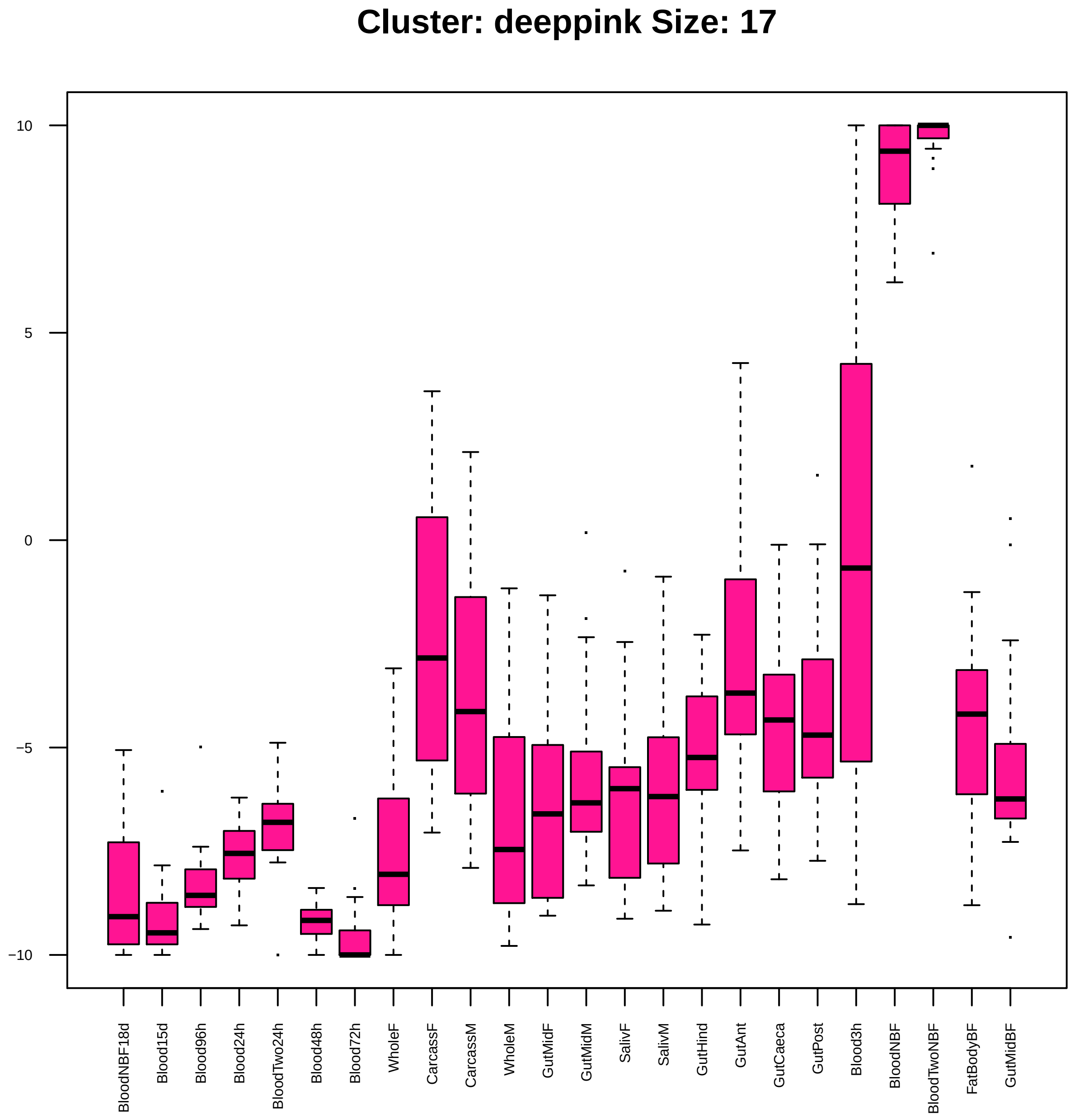

Cluster: deeppink Size: 17
10
5
0
−5
−10
BloodNBF18d
Blood15d
Blood96h
Blood24h
BloodTwo24h
Blood48h
Blood72h
WholeF
CarcassF
CarcassM
WholeM
GutMidF
GutMidM
SalivF
SalivM
GutHind
GutAnt
GutCaeca
GutPost
Blood3h
BloodNBF
BloodTwoNBF
FatBodyBF
GutMidBF

| | GO.ID | BPCluster: deeppink Size: 17 | Annotated | Significant | Expected | Rank in ClassicF | Weight01F | ClassicF |
|---|---|---|---|---|---|---|---|---|
| *1* | GO:0006030 | chitin metabolic process | 95 | 2 | 0.06 | 1 | 0.0013 | 0.0013 |
| *2* | GO:0005975 | carbohydrate metabolic process | 210 | 2 | 0.13 | 5 | 0.0062 | 0.0062 |
| *14* | GO:0017144 | drug metabolic process | 246 | 2 | 0.15 | 6 | 1.0000 | 0.0084 |
| *28* | GO:0006040 | amino sugar metabolic process | 99 | 2 | 0.06 | 2 | 1.0000 | 0.0014 |
| *30* | GO:1901071 | glucosamine−containing compound metaboli... | 99 | 2 | 0.06 | 3 | 1.0000 | 0.0014 |

| | GO.ID | MFCluster: deeppink Size: 17 | Annotated | Significant | Expected | Rank in ClassicF | Weight01F | ClassicF |
|---|---|---|---|---|---|---|---|---|
| *1* | GO:0042302 | structural constituent of cuticle | 99 | 3 | 0.11 | 1 | 0.0023 | 0.00013 |
| *2* | GO:0008061 | chitin binding | 76 | 2 | 0.08 | 3 | 0.0029 | 0.00295 |
| *4* | GO:0016810 | hydrolase activity, acting on carbon−nit... | 56 | 2 | 0.06 | 2 | 0.0232 | 0.00161 |
| *13* | GO:0005198 | structural molecule activity | 318 | 3 | 0.35 | 4 | 1.0000 | 0.00404 |

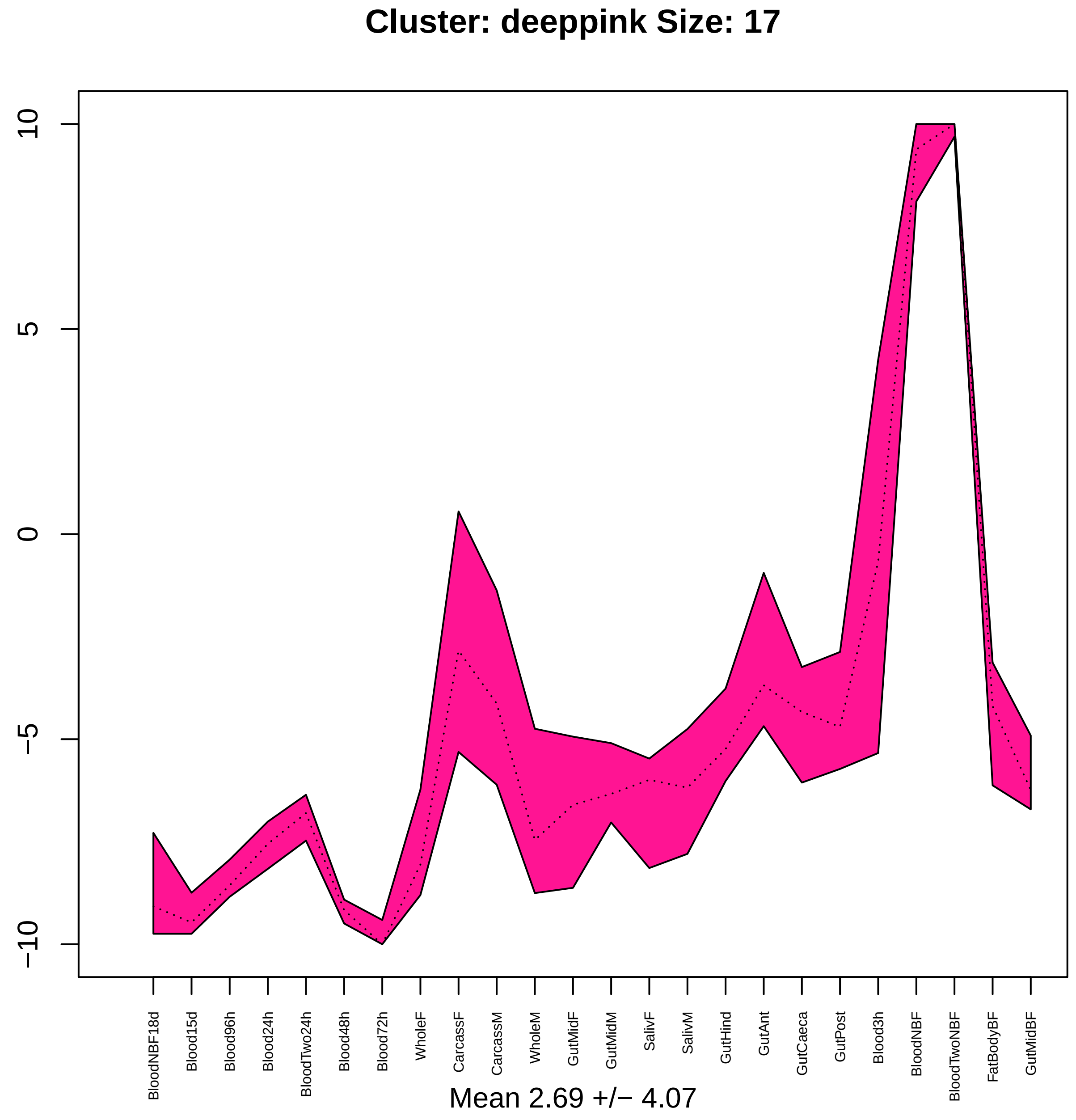

Cluster: deeppink Size: 17
10
5
0
−5
−10
BloodNBF18d
Blood15d
Blood96h
Blood24h
BloodTwo24h
Blood48h
Blood72h
WholeF
CarcassF
CarcassM
WholeM
GutMidF
GutMidM
SalivF
SalivM
GutHind
GutAnt
GutCaeca
GutPost
Blood3h
BloodNBF
BloodTwoNBF
FatBodyBF
GutMidBF
Mean 2.69 +/− 4.07

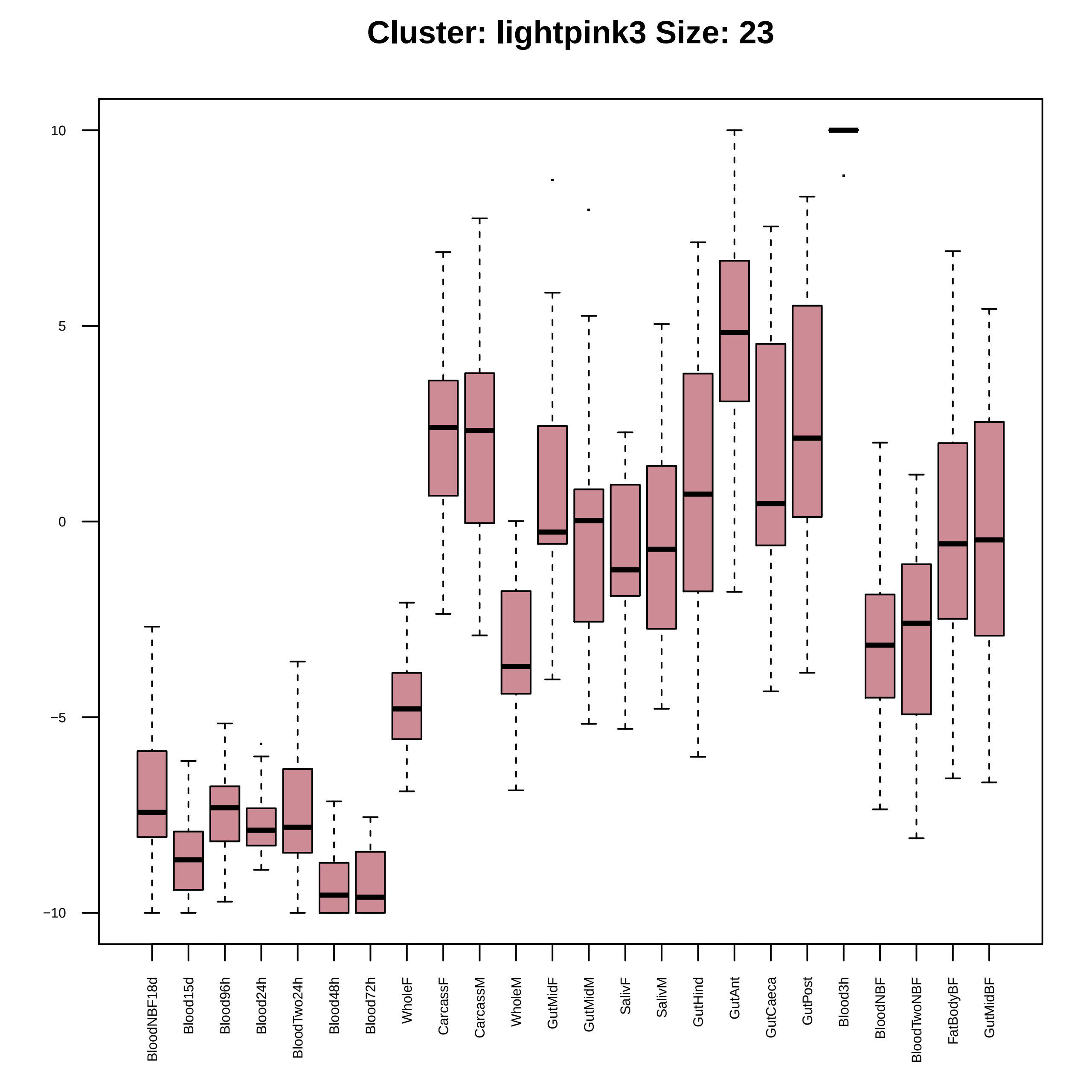
Cluster: lightpink3 Size: 23
10
5
0
−5
−10
BloodNBF18d
Blood15d
Blood96h
Blood24h
BloodTwo24h
Blood48h
Blood72h
WholeF
CarcassF
CarcassM
WholeM
GutMidF
GutMidM
SalivF
SalivM
GutHind
GutAnt
GutCaeca
GutPost
Blood3h
BloodNBF
BloodTwoNBF
FatBodyBF
GutMidBF

| | GO.ID | BPCluster: lightpink3 Size: 23 | Annotated | Significant | Expected | Rank in ClassicF | Weight01F | ClassicF |
|---|---|---|---|---|---|---|---|---|
| 1 | GO:0035159 | regulation of tube length, open tracheal... | 21 | 2 | 0.03 | 1 | 0.00029 | 0.00029 |
| 2 | GO:0006030 | chitin metabolic process | 95 | 2 | 0.12 | 6 | 0.00580 | 0.00580 |

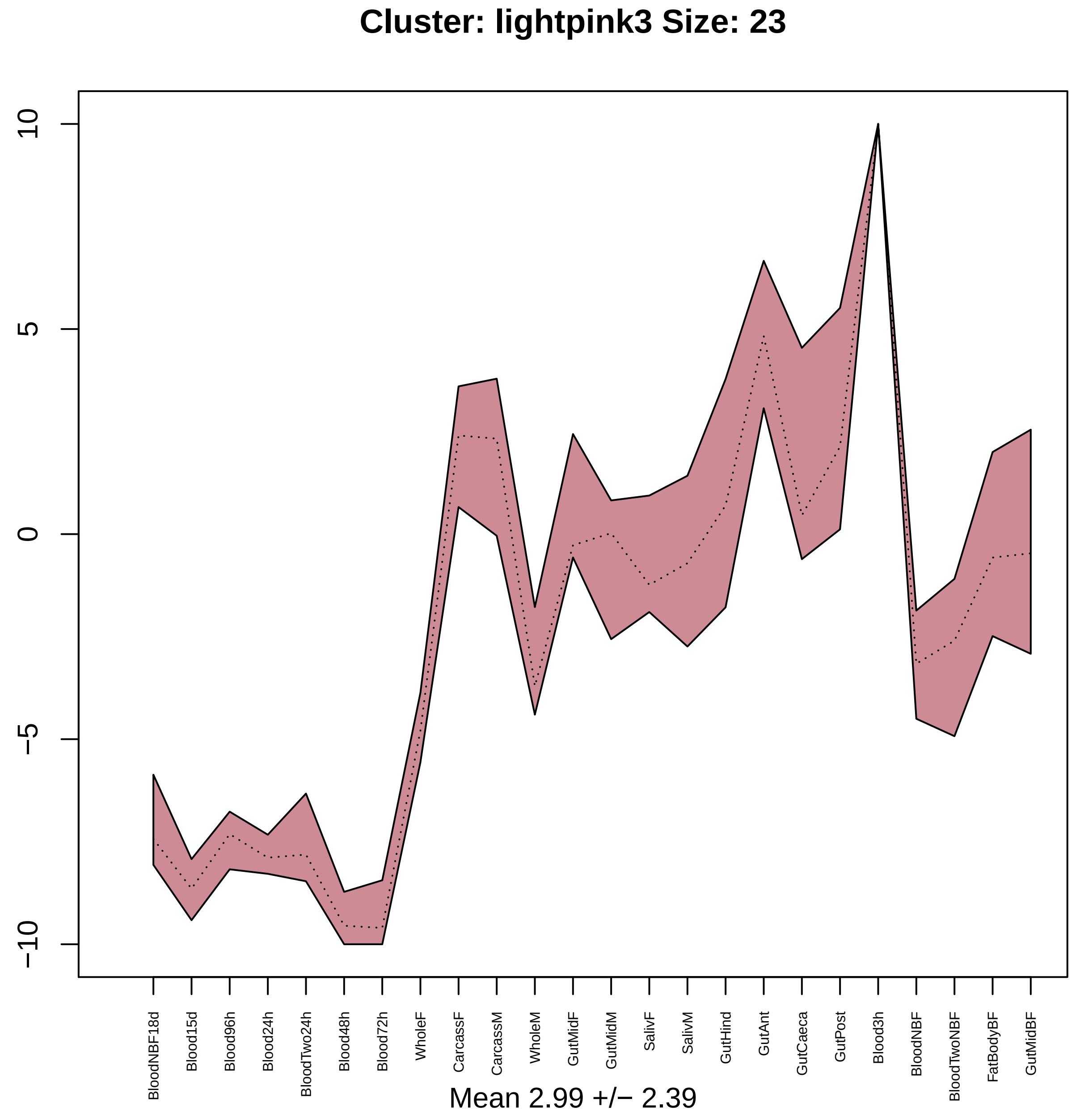

Cluster: lightpink3 Size: 23
10
5
0
−5
−10
BloodNBF18d
Blood15d
Blood96h
Blood24h
BloodTwo24h
Blood48h
Blood72h
WholeF
CarcassF
CarcassM
WholeM
GutMidF
GutMidM
SalivF
SalivM
GutHind
GutAnt
GutCaeca
GutPost
Blood3h
BloodNBF
BloodTwoNBF
FatBodyBF
GutMidBF
Mean 2.99 +/− 2.39

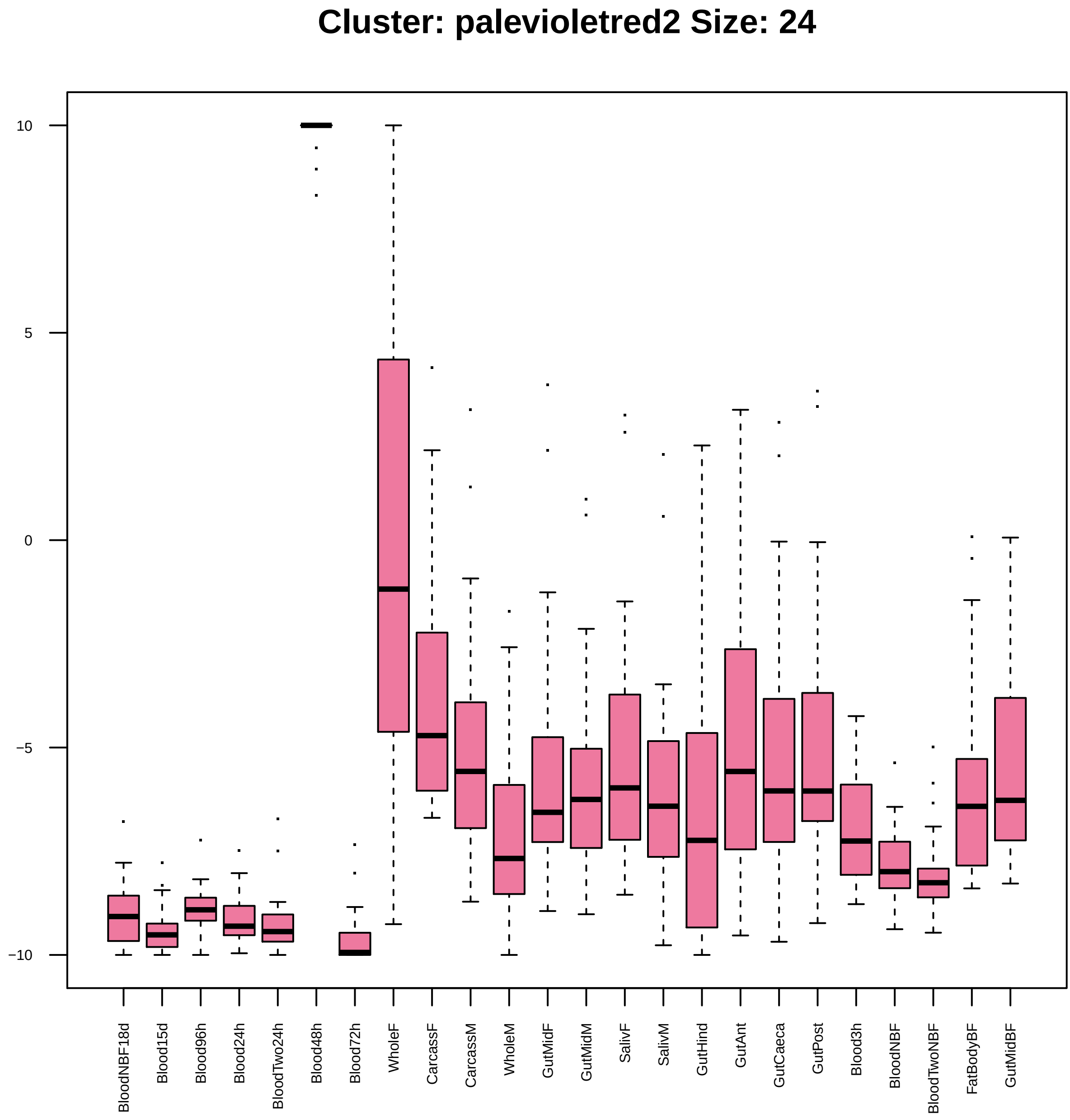
Cluster: palevioletred2 Size: 24
10
5
0
−5
−10
BloodNBF18d
Blood15d
Blood96h
Blood24h
BloodTwo24h
Blood48h
Blood72h
WholeF
CarcassF
CarcassM
WholeM
GutMidF
GutMidM
SalivF
SalivM
GutHind
GutAnt
GutCaeca
GutPost
Blood3h
BloodNBF
BloodTwoNBF
FatBodyBF
GutMidBF

| | GO.ID | BPCluster: palevioletred2 Size: 24 | Annotated | Significant | Expected | Rank in ClassicF | Weight01F | ClassicF |
|---|---|---|---|---|---|---|---|---|
| *1* | GO:0006979 | response to oxidative stress | 51 | 2 | 0.04 | 1 | 0.00062 | 0.00062 |
| *2* | GO:0006030 | chitin metabolic process | 95 | 2 | 0.07 | 2 | 0.00213 | 0.00213 |

| | GO.ID | MFCluster: palevioletred2 Size: 24 | Annotated | Significant | Expected | Rank in ClassicF | Weight01F | ClassicF |
|---|---|---|---|---|---|---|---|---|
| 2 | GO:0004601 | peroxidase activity | 26 | 2 | 0.06 | 2 | 0.0015 | 0.0015 |
| 8 | GO:0005488 | binding | 4627 | 15 | 10.27 | 5 | 1.0000 | 0.0082 |
| 9 | GO:0016684 | oxidoreductase activity, acting on perox... | 26 | 2 | 0.06 | 3 | 1.0000 | 0.0015 |
| 11 | GO:0016209 | antioxidant activity | 33 | 2 | 0.07 | 4 | 1.0000 | 0.0023 |

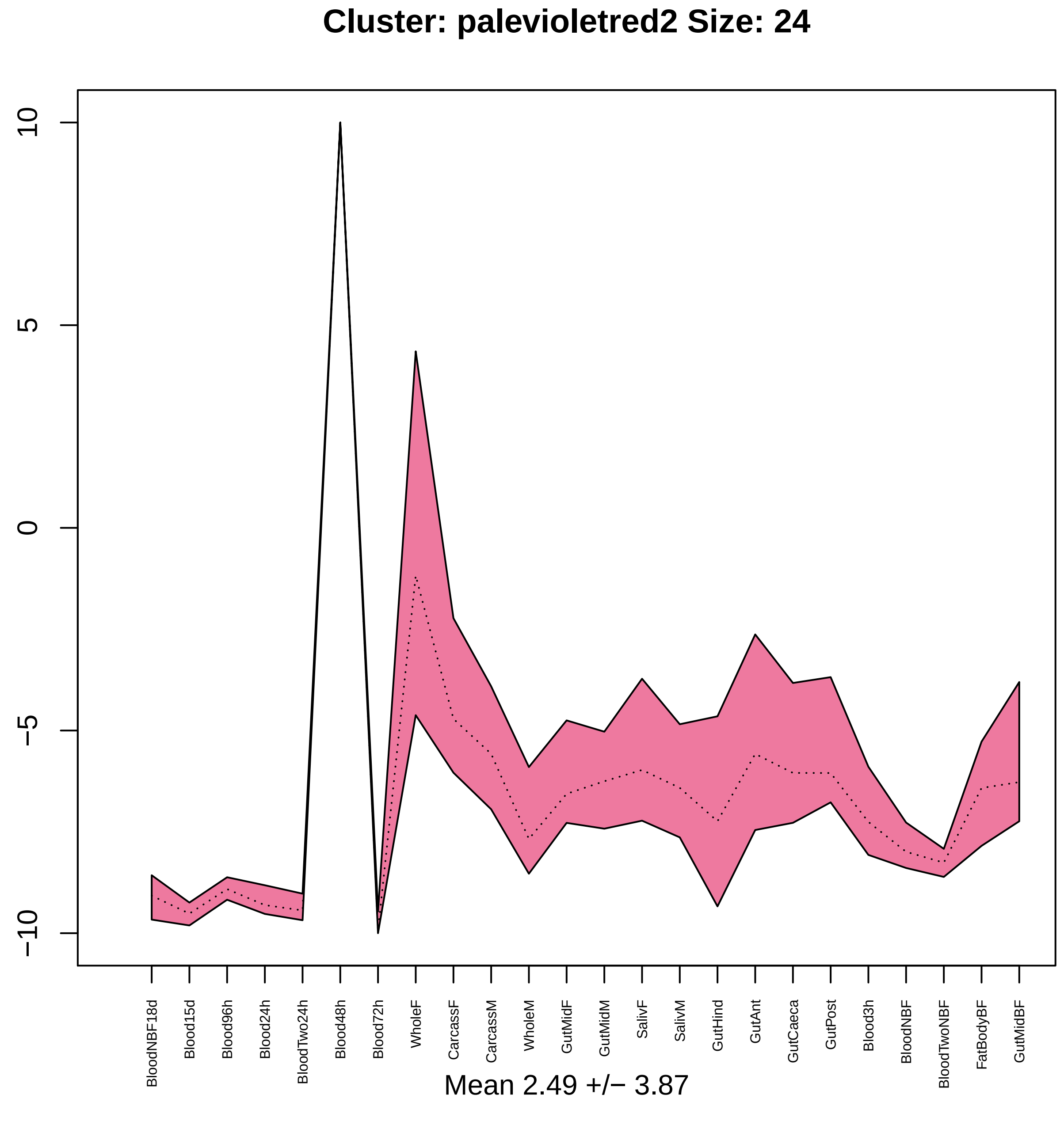

Cluster: palevioletred2 Size: 24
10
5
0
−5
−10
BloodNBF18d
Blood15d
Blood96h
Blood24h
BloodTwo24h
Blood48h
Blood72h
WholeF
CarcassF
CarcassM
WholeM
GutMidF
GutMidM
SalivF
SalivM
GutHind
GutAnt
GutCaeca
GutPost
Blood3h
BloodNBF
BloodTwoNBF
FatBodyBF
GutMidBF
Mean 2.49 +/− 3.87

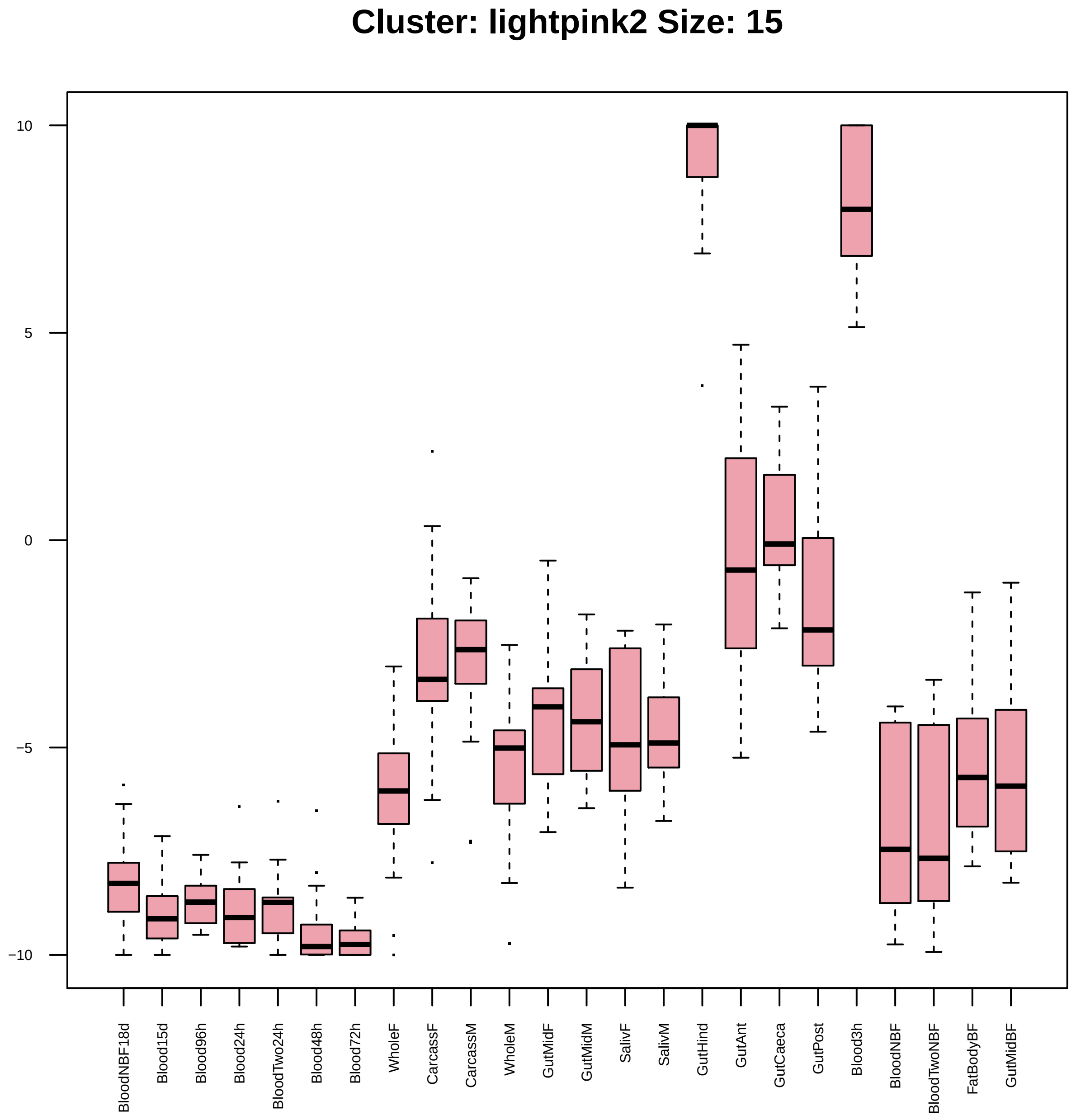

Cluster: lightpink2 Size: 15
10
5
0
−5
−10
BloodNBF18d
Blood15d
Blood96h
Blood24h
BloodTwo24h
Blood48h
Blood72h
WholeF
CarcassF
CarcassM
WholeM
GutMidF
GutMidM
SalivF
SalivM
GutHind
GutAnt
GutCaeca
GutPost
Blood3h
BloodNBF
BloodTwoNBF
FatBodyBF
GutMidBF

| | GO.ID | BPCluster: lightpink2 Size: 15 | Annotated | Significant | Expected | Rank in ClassicF | Weight01F | ClassicF |
|---|---|---|---|---|---|---|---|---|
| *2* | GO:0006030 | chitin metabolic process | 95 | 2 | 0.06 | 1 | 0.033 | 0.0013 |
| *8* | GO:1901071 | glucosamine−containing compound metaboli... | 99 | 2 | 0.06 | 2 | 1.000 | 0.0014 |
| *9* | GO:0006022 | aminoglycan metabolic process | 113 | 2 | 0.07 | 4 | 1.000 | 0.0018 |

| | GO.ID | MFCluster: lightpink2 Size: 15 | Annotated | Significant | Expected | Rank in ClassicF | Weight01F | ClassicF |
|---|---|---|---|---|---|---|---|---|
| 1 | GO:0008061 | chitin binding | 76 | 2 | 0.05 | 1 | 0.0011 | 0.0011 |

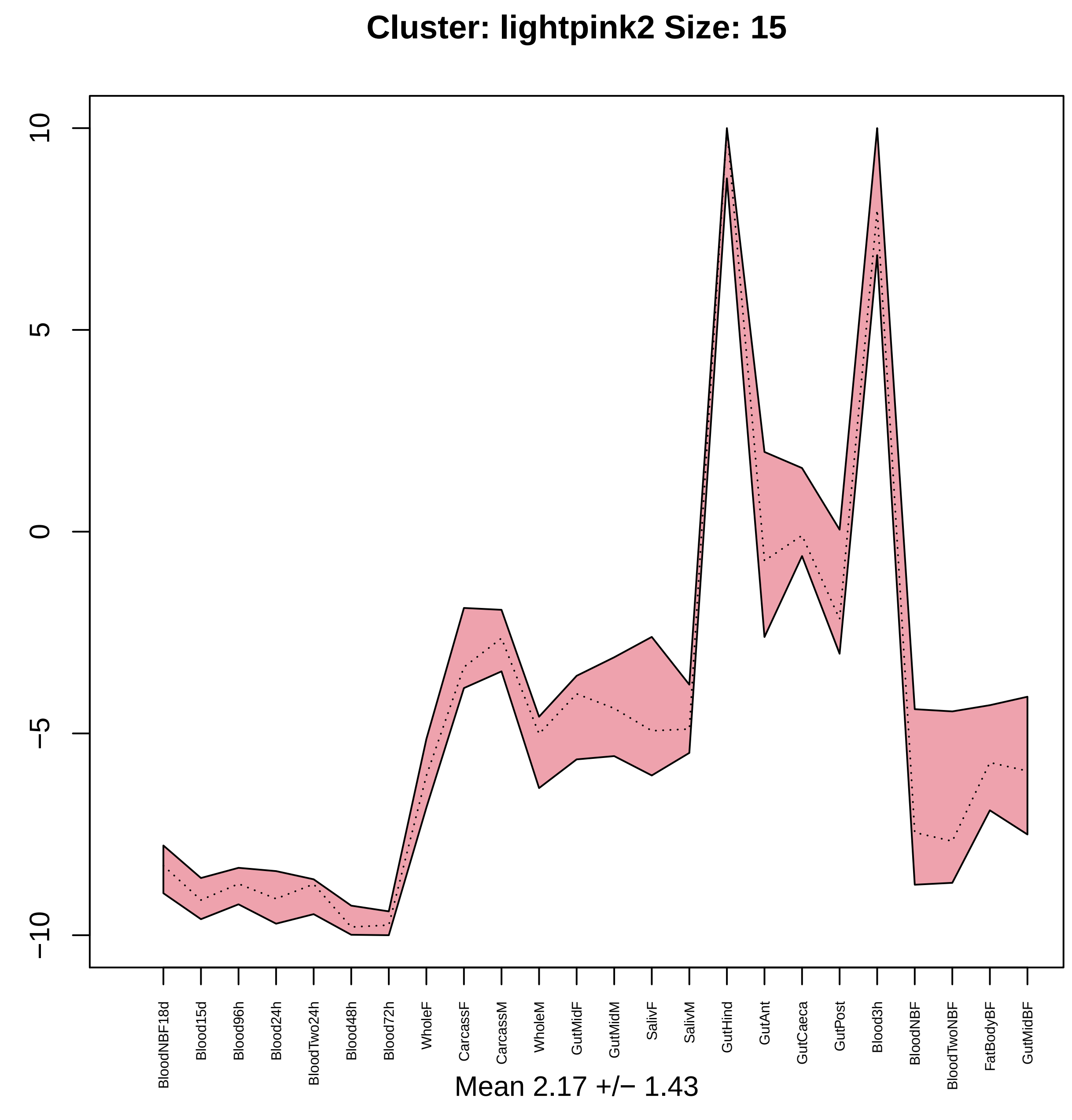
Cluster: lightpink2 Size: 15
10
5
0
−5
−10
BloodNBF18d
Blood15d
Blood96h
Blood24h
BloodTwo24h
Blood48h
Blood72h
WholeF
CarcassF
CarcassM
WholeM
GutMidF
GutMidM
SalivF
SalivM
GutHind
GutAnt
GutCaeca
GutPost
Blood3h
BloodNBF
BloodTwoNBF
FatBodyBF
GutMidBF
Mean 2.17 +/− 1.43

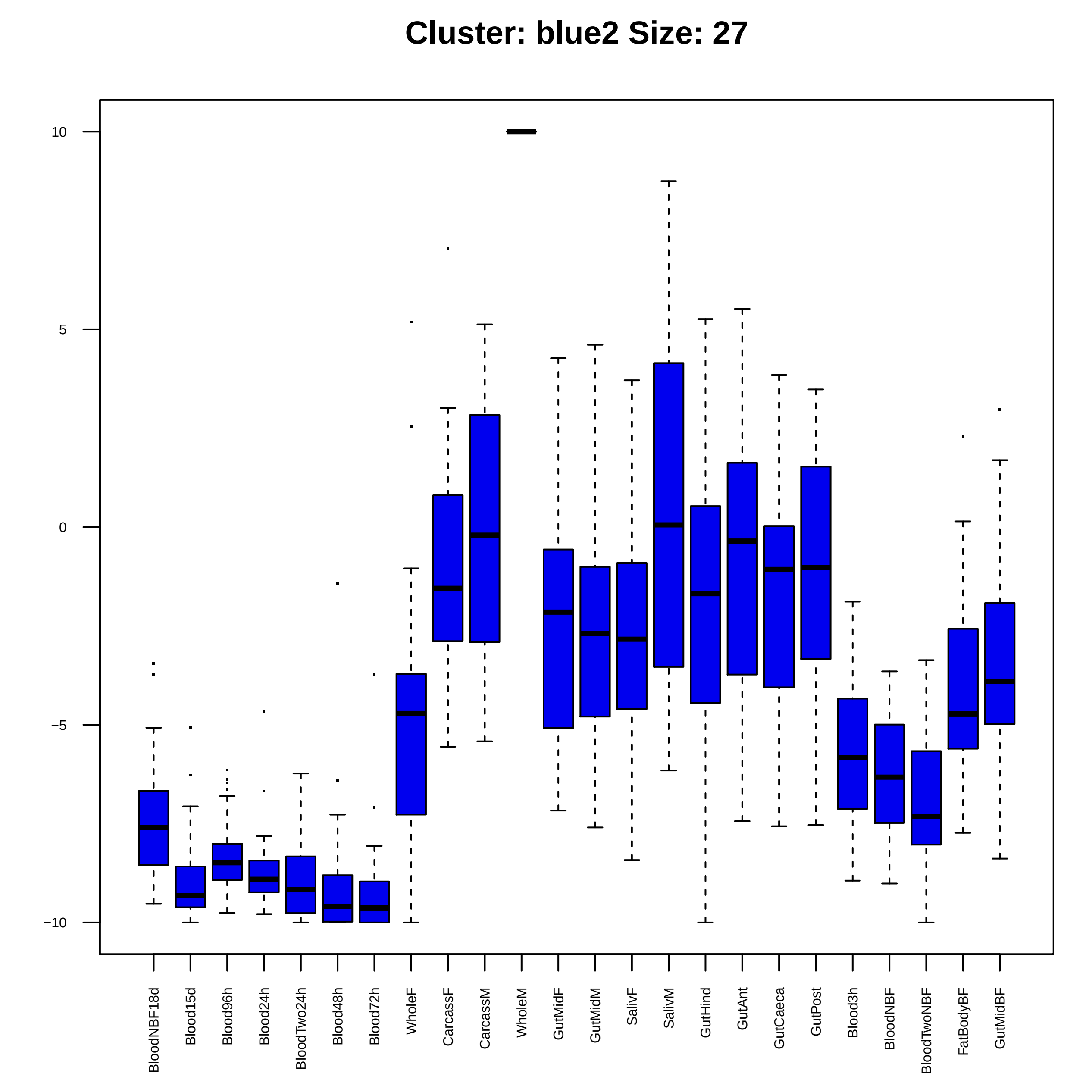

Cluster: blue2 Size: 27
10
5
0
−5
−10
BloodNBF18d
Blood15d
Blood96h
Blood24h
BloodTwo24h
Blood48h
Blood72h
WholeF
CarcassF
CarcassM
WholeM
GutMidF
GutMidM
SalivF
SalivM
GutHind
GutAnt
GutCaeca
GutPost
Blood3h
BloodNBF
BloodTwoNBF
FatBodyBF
GutMidBF

| | GO.ID | BPCluster: blue2 Size: 27 | Annotated | Significant | Expected | Rank in ClassicF | Weight01F | ClassicF |
|---|---|---|---|---|---|---|---|---|
| *1* | GO:0007018 | microtubule−based movement | 65 | 3 | 0.14 | 1 | 0.00034 | 0.00034 |

| | GO.ID | MFCluster: blue2 Size: 27 | Annotated | Significant | Expected | Rank in ClassicF | Weight01F | ClassicF |
|---|---|---|---|---|---|---|---|---|
| 1 | GO:0016887 | ATPase activity | 233 | 4 | 0.52 | 2 | 0.0028 | 0.00142 |
| 2 | GO:0003777 | microtubule motor activity | 40 | 2 | 0.09 | 3 | 0.0034 | 0.00343 |
| 4 | GO:0003774 | motor activity | 60 | 3 | 0.13 | 1 | 0.0384 | 0.00028 |

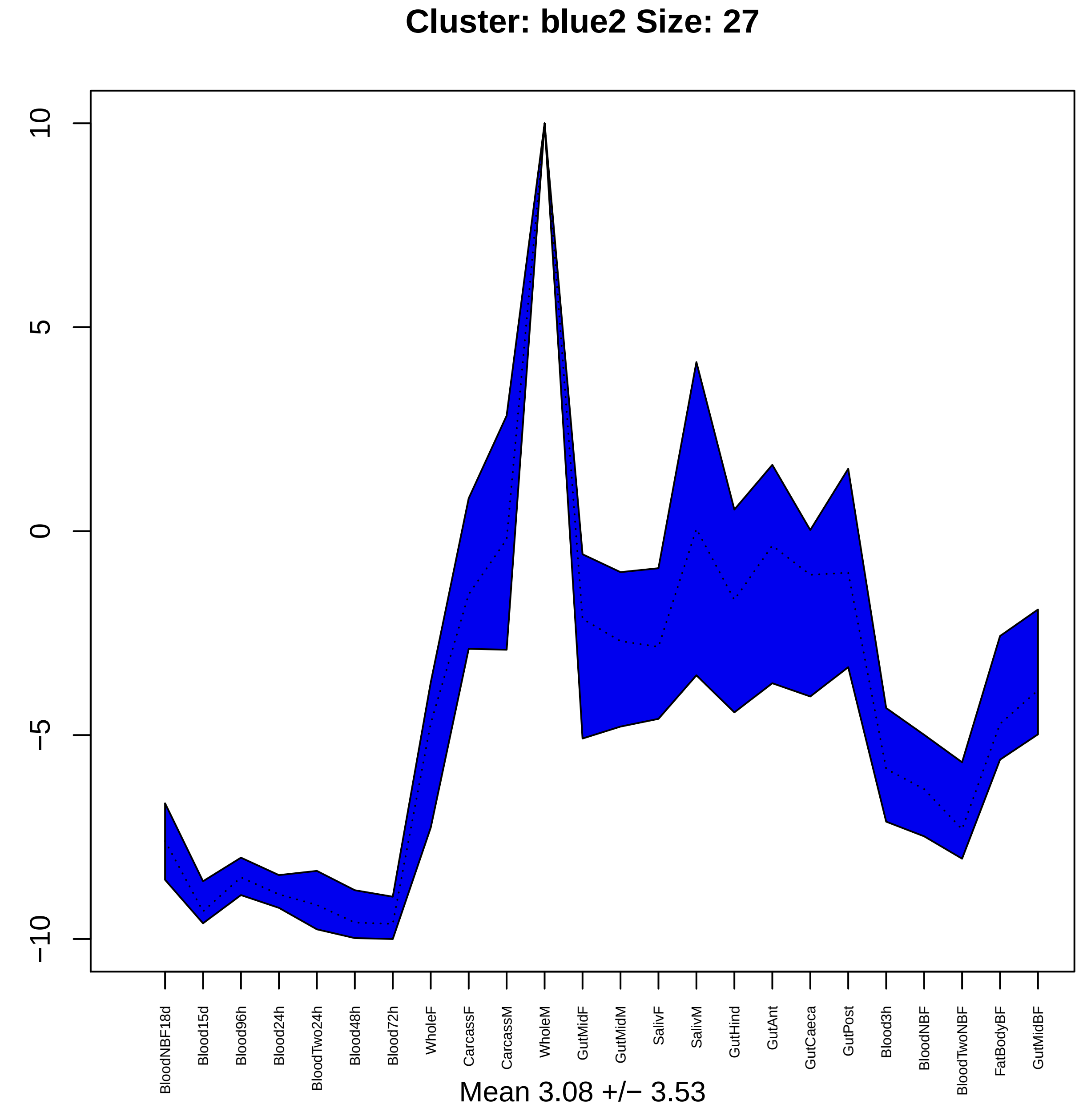

Cluster: blue2 Size: 27
10
5
0
−5
−10
BloodNBF18d
Blood15d
Blood96h
Blood24h
BloodTwo24h
Blood48h
Blood72h
WholeF
CarcassF
CarcassM
WholeM
GutMidF
GutMidM
SalivF
SalivM
GutHind
GutAnt
GutCaeca
GutPost
Blood3h
BloodNBF
BloodTwoNBF
FatBodyBF
GutMidBF
Mean 3.08 +/− 3.53

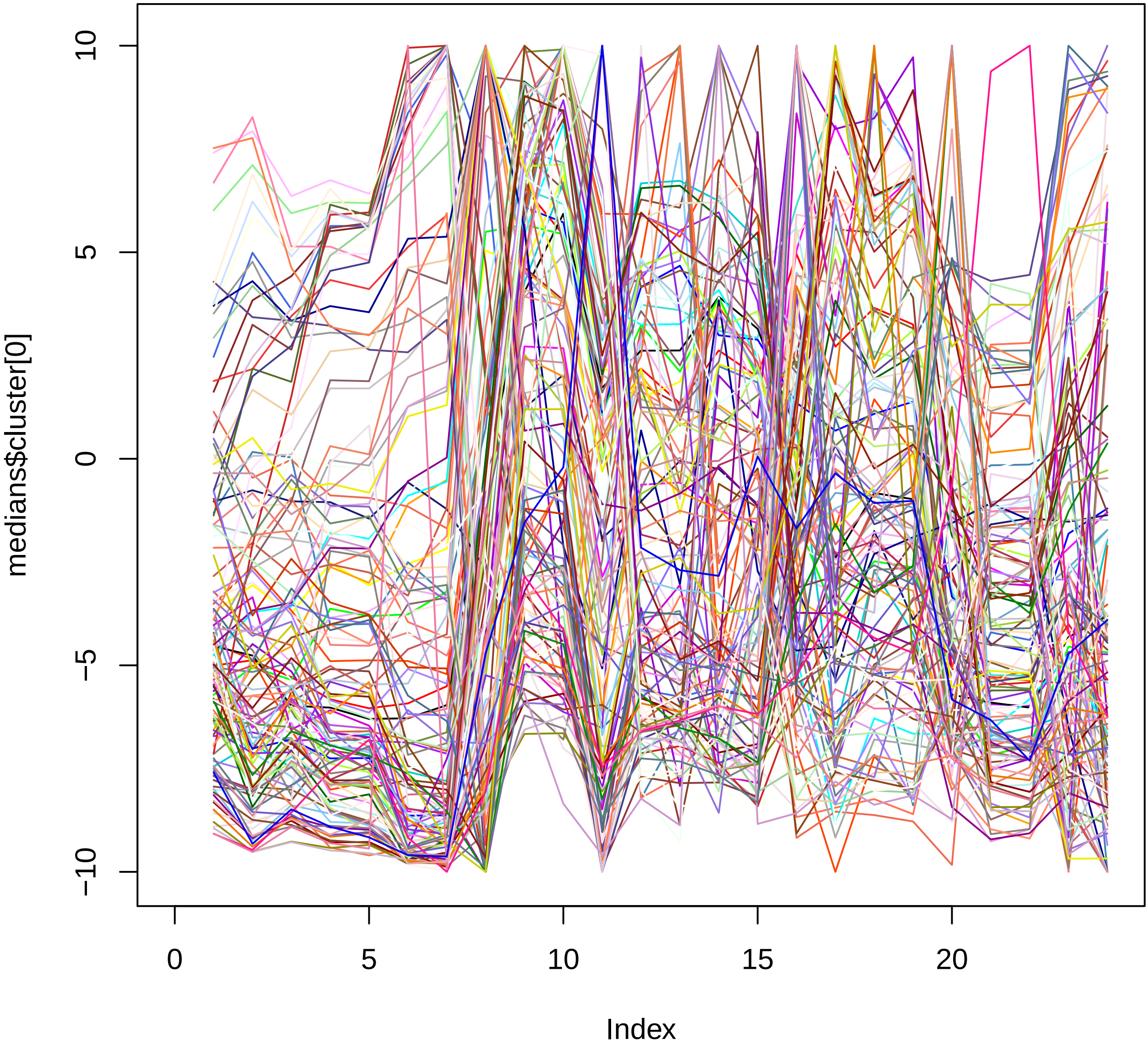

10
5
0
−5
−10
medians$cluster[0]
0
5
10
15
20
Index
