# Supplementary material for: Functional Constraints on Insect Immune System Components Govern Their Evolutionary Trajectories
Source: Mol Biol Evol. 2021 Dec 10;39(1):msab352. doi: 10.1093/molbev/msab352 (PMC8788225; doi:10.1093/molbev/msab352)

Additional File 4

Evolutionary similarities (based on feature metric medians) of pairs of gene families are compared with their expression similarities as in main text Figure 4. Additional comparisons of evolutionary similarities versus expression similarities are presented showing evolutionary similarities computed with mean (EvolMean) and median (EvolMedian) metrics for the VectorBase cells (ExprCell) and supercells (ExprSupercell) as well as for the WGCNA expression modules for levels 0-4 (ExprModuleN). Plots are built as for main text Figure 4, but with automatic label placement and without colouring by immune categories. Median values are shown as horizontal and vertical lines in yellow for all points and in orange for significant (purple) points. Family acronyms are defined in Table 2 (main text). See supplementary materials for details.

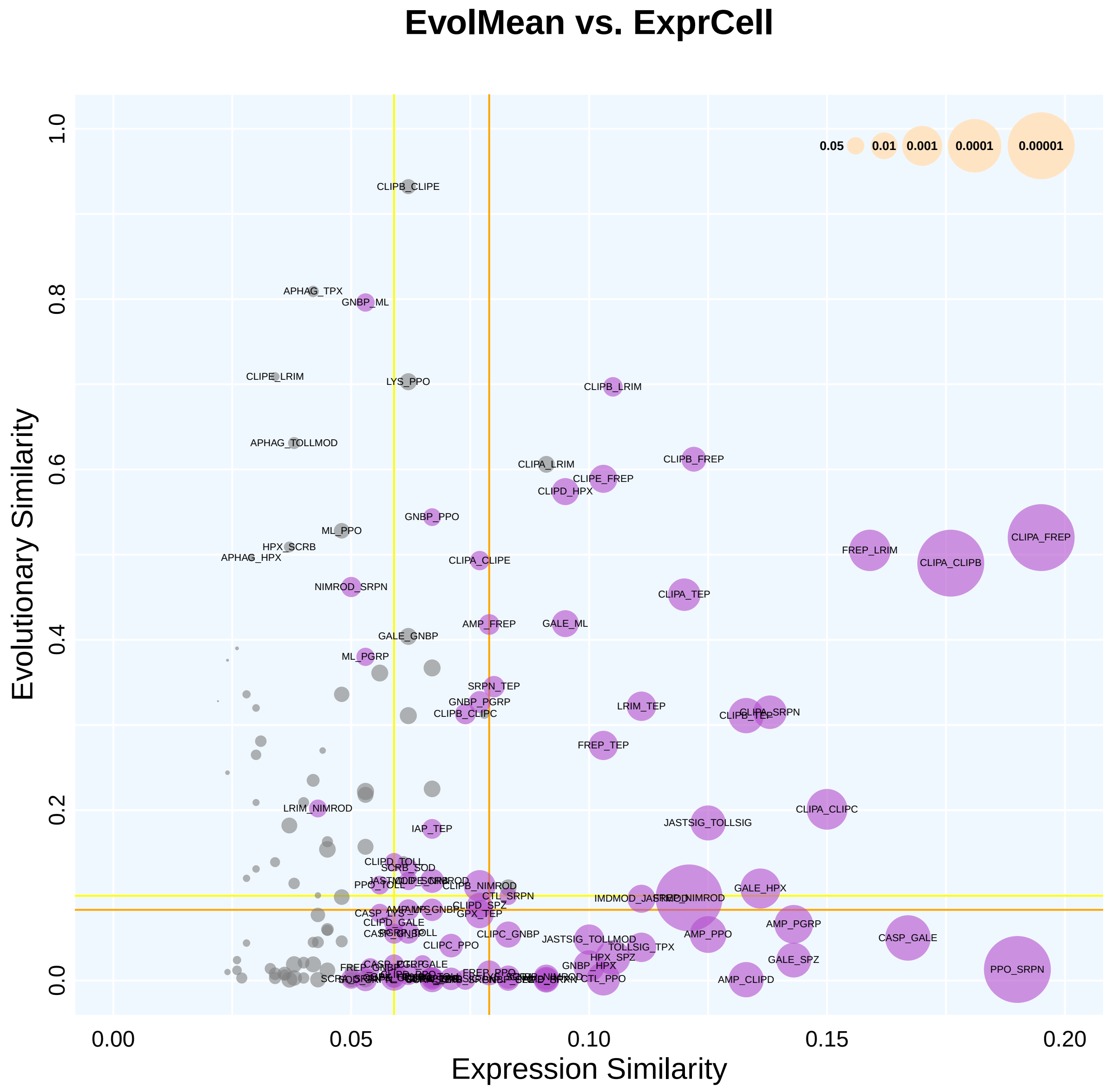

EvolMean vs. ExprCell
Evolutionary Similarity
Expression Similarity
0.0
0.2
0.4
0.6
0.8
1.0
0.00
0.05
0.10
0.15
0.20
0.05
0.01
0.001
0.0001
0.00001
CLIPB_CLIPE
APHAG_TPX
GNBP_ML
CLIPE_LRIM
LYS_PPO
CLIPB_LRIM
APHAG_TOLLMOD
CLIPB_FREP
CLIPA_LRIM
CLIPE_FREP
CLIPD_HPX
GNBP_PPO
ML_PPO
HPX_SCRB
APHAG_HPX
CLIPA_CLIPE
FREP_LRIM
CLIPA_FREP
CLIPA_CLIPB
NIMROD_SRPN
CLIPA_TEP
AMP_FREP
GALE_ML
GALE_GNBP
ML_PGRP
SRPN_TEP
GNBP_PGRP
CLIPB_CLIPC
LRIM_TEP
CLIPA_SRPN
FREP_TEP
CLIPA_CLIPC
LRIM_NIMROD
JASTSIG_TOLLSIG
IAP_TEP
CLIPD_TOLL
SCRB_SOD
PPO_TOLL
CLIPB_NIMROD
GALE_HPX
CTL_SRPN
IMDMOD_JASTSIG
CLIPD_SPZ
GPX_TEP
CASP_LYS
AMP_PGRP
CLIPD_GALE
AMP_PPO
CLIPC_GNBP
JASTSIG_TOLLMOD
CASP_GALE
TOLLSIG_TPX
CLIPC_PPO
HPX_SPZ
GALE_SPZ
GNBP_HPX
CTL_PPO
PPO_SRPN
AMP_CLIPD

EvolMean vs. ExprSupercell

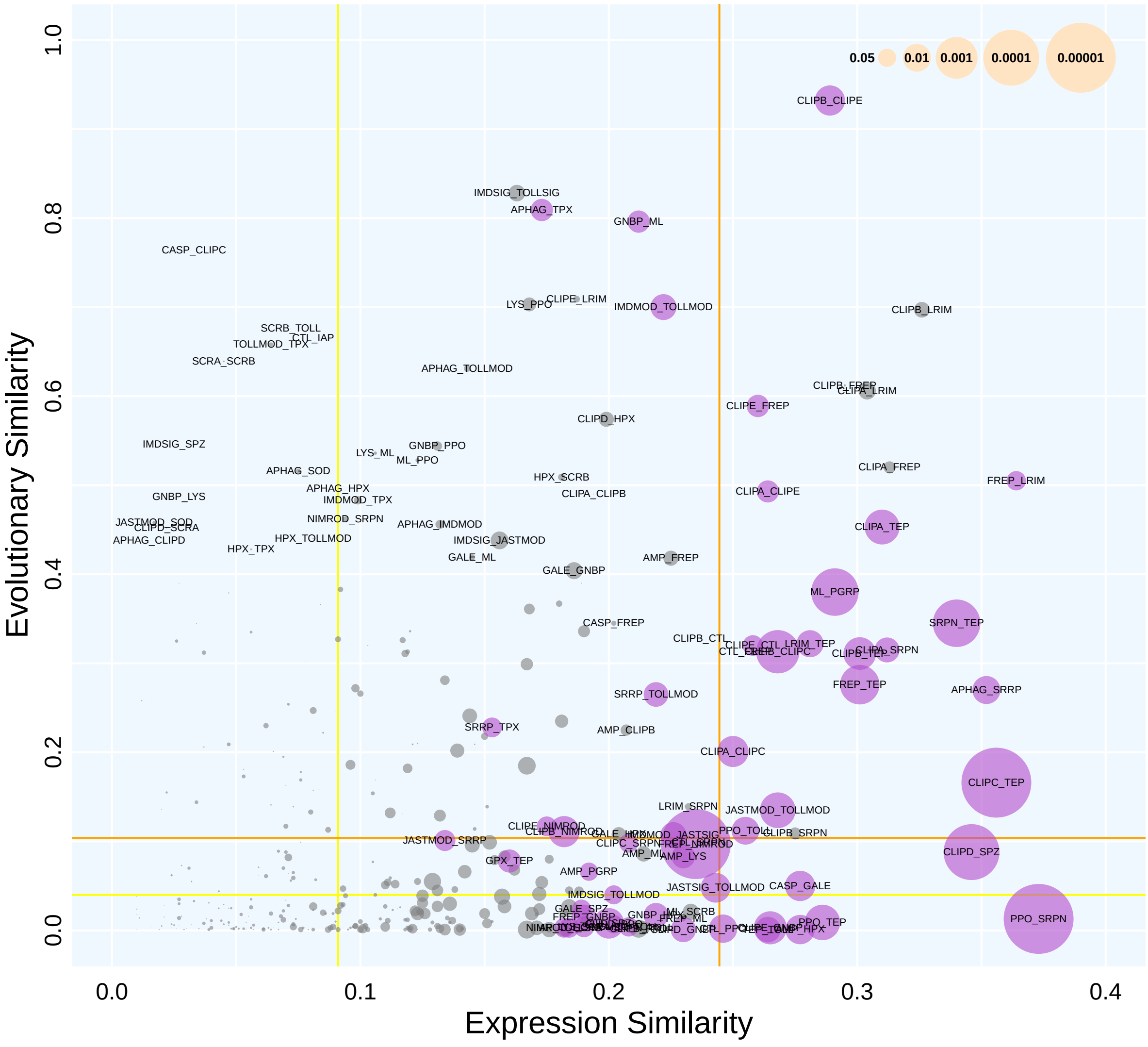

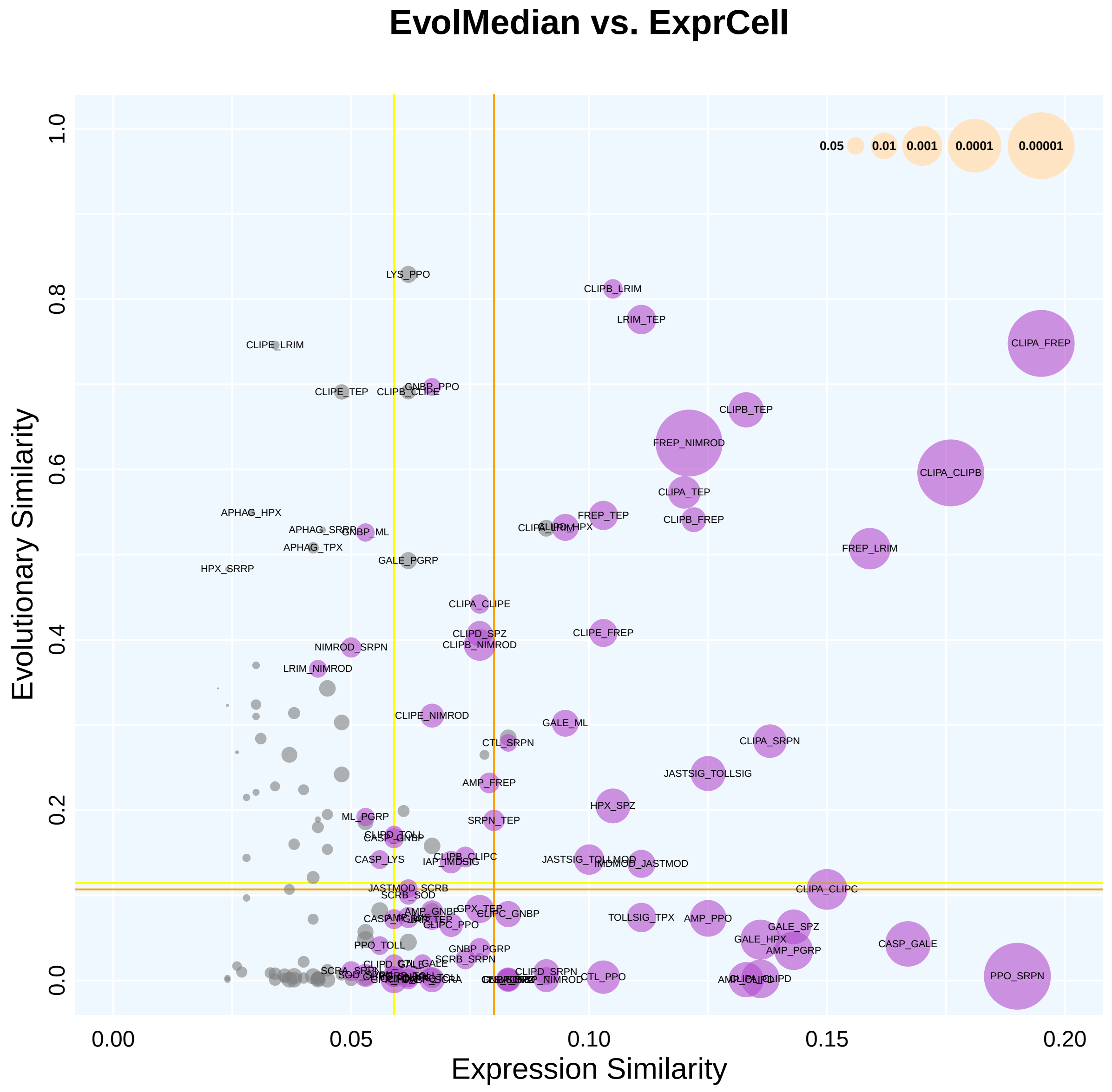
EvolMedian vs. ExprCell
Evolutionary Similarity
Expression Similarity
0.0
0.2
0.4
0.6
0.8
1.0
0.00
0.05
0.10
0.15
0.20
0.05
0.01
0.001
0.0001
0.00001
LYS_PPO
CLIPB_LRIM
LRIM_TEP
CLIPE_LRIM
CLIPA_FREP
CLIPE_TEP
GNBP_PPO
CLIPB_CLIPE
CLIPB_TEP
FREP_NIMROD
CLIPA_CLIPB
CLIPA_TEP
APHAG_HPX
FREP_TEP
CLIPB_FREP
APHAG_SRRP
GNBP_ML
FREP_LRIM
APHAG_TPX
GALE_PGRP
HPX_SRRP
CLIPA_CLIPE
CLIPD_SPZ
CLIPE_FREP
NIMROD_SRPN
CLIPB_NIMROD
LRIM_NIMROD
CLIPE_NIMROD
GALE_ML
CTL_SRPN
CLIPA_SRPN
JASTSIG_TOLLSIG
AMP_FREP
HPX_SPZ
ML_PGRP
SRPN_TEP
CASP_GNBP
CASP_LYS
IAP_IMDSIG
CLIPB_CLIPC
JASTSIG_TOLLMOD
IMDMOD_JASTMOD
JASTMOD_SCRB
SCRB_SOD
CLIPA_CLIPC
GPX_TEP
CLIPC_GNBP
AMP_GNBP
TOLLSIG_TPX
AMP_PPO
CLIPC_PPO
GALE_SPZ
GALE_HPX
CASP_GALE
AMP_PGRP
PPO_TOLL
GNBP_PGRP
SCRB_SRPN
CLIPD_SRPN
CTL_PPO
PPO_SRPN

EvolMedian vs. ExprSupercell

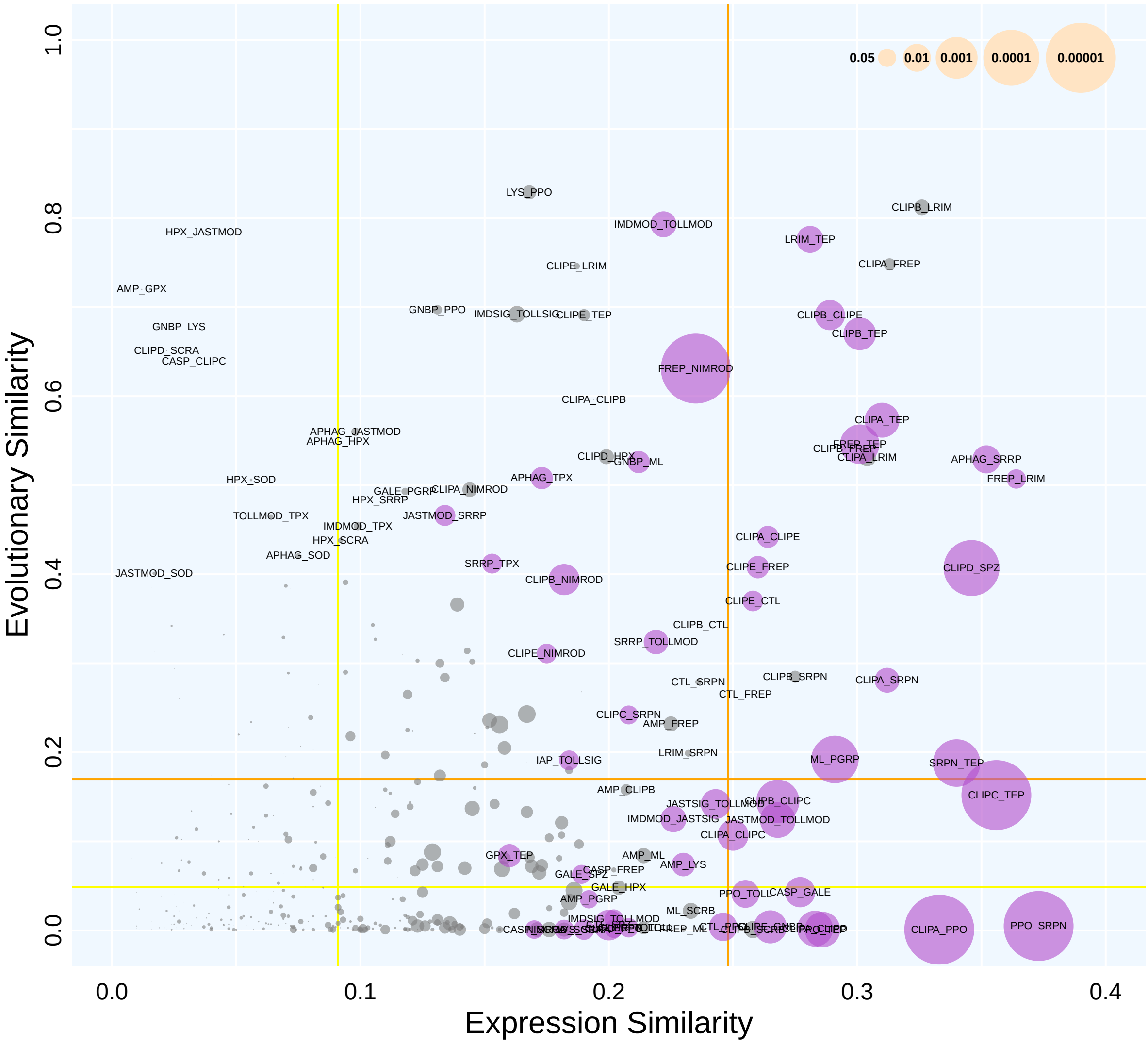

EvolMean vs. ExprModule0

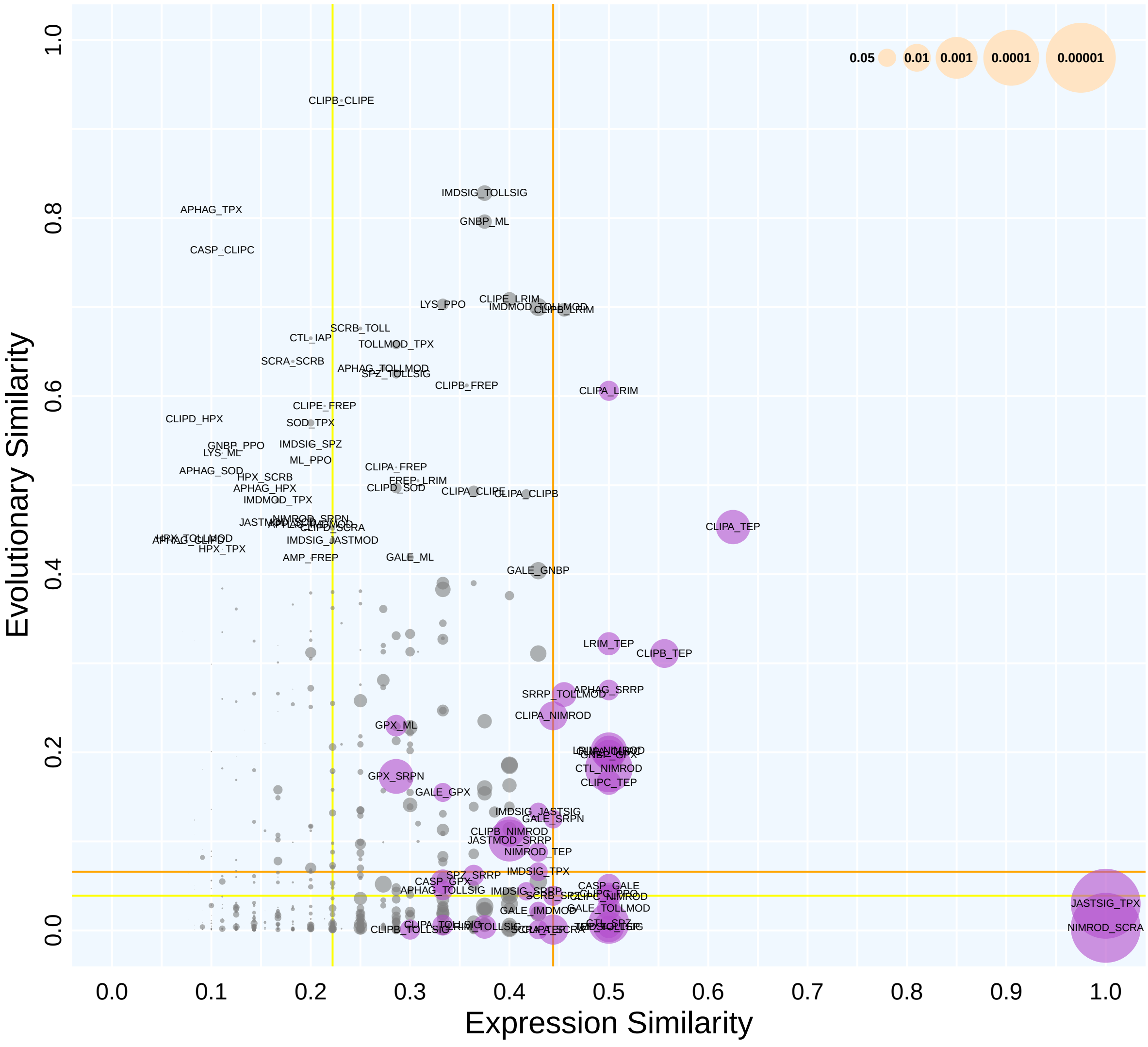

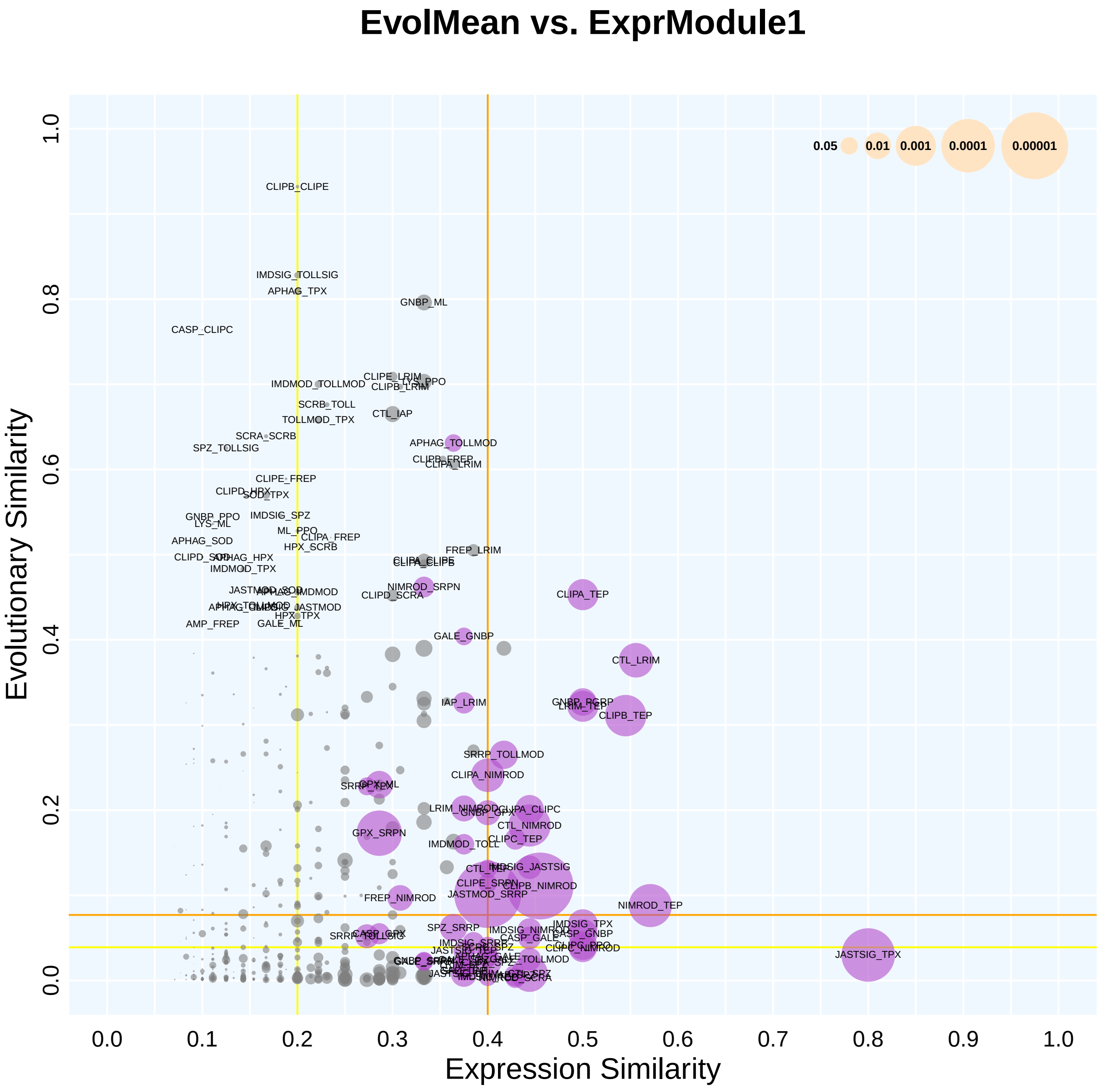
EvolMean vs. ExprModule1
Evolutionary Similarity
Expression Similarity
0.05
0.01
0.001
0.0001
0.00001
0.0
0.1
0.2
0.3
0.4
0.5
0.6
0.7
0.8
0.9
1.0
CLIPB_CLIPE
IMDSIG_TOLLSIG
APHAG_TPX
GNBP_ML
CASP_CLIPC
IMDMOD_TOLLMOD
CLIPE_LRIM
LYS_PPO
CLIPB_LRIM
SCRB_TOLL
CTL_IAP
TOLLMOD_TPX
SCRA_SCRB
SPZ_TOLLSIG
APHAG_TOLLMOD
CLIPA_LRIM
CLIPE_FREP
GNBP_PPO
LYS_ML
IMDSIG_SPZ
ML_PPO
CLIPA_FREP
APHAG_SOD
HPX_SCRB
FREP_LRIM
IMDMOD_TPX
NIMROD_SRPN
CLIPD_SCRA
CLIPA_TEP
HPX_TPX
AMP_FREP
GALE_ML
GALE_GNBP
CTL_LRIM
IAP_LRIM
CLIPB_TEP
SRRP_TOLLMOD
CLIPA_NIMROD
GPX_SRPN
CTL_NIMROD
CLIPC_TEP
IMDMOD_TOLL
FREP_NIMROD
JASTMOD_SRRP
NIMROD_TEP
SPZ_SRRP
IMDSIG_TPX
JASTSIG_TPX

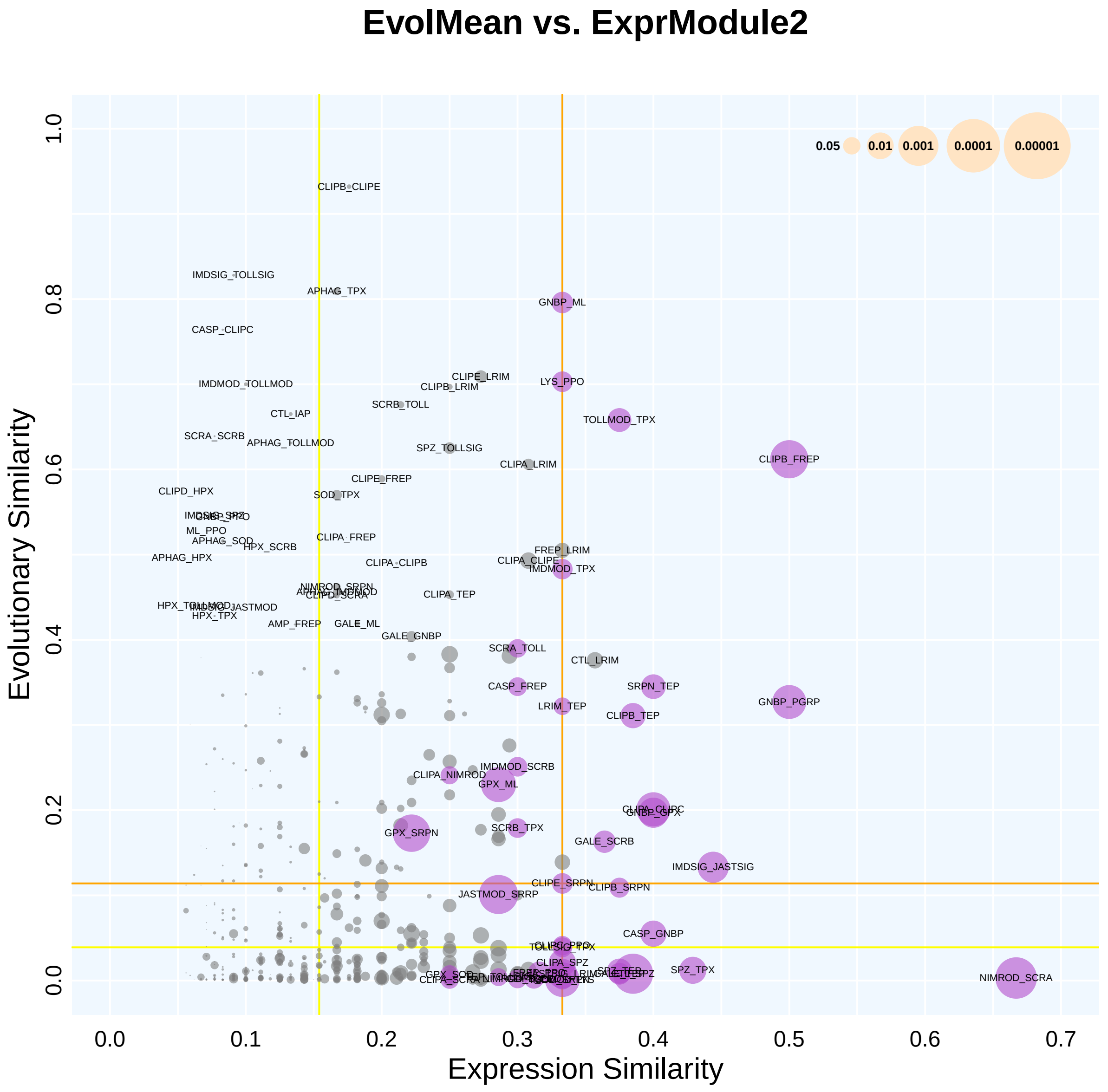
EvolMean vs. ExprModule2
Evolutionary Similarity
Expression Similarity
0.0
0.2
0.4
0.6
0.8
1.0
0.0
0.1
0.2
0.3
0.4
0.5
0.6
0.7
0.05
0.01
0.001
0.0001
0.00001
CLIPB_CLIPE
IMDSIG_TOLLSIG
APHAG_TPX
GNBP_ML
CASP_CLIPC
CLIPE_LRIM
CLIPB_LRIM
LYS_PPO
IMDMOD_TOLLMOD
SCRB_TOLL
CTL_IAP
TOLLMOD_TPX
SCRA_SCRB
APHAG_TOLLMOD
SPZ_TOLLSIG
CLIPA_LRIM
CLIPB_FREP
CLIPE_FREP
CLIPD_HPX
SOD_TPX
ML_PPO
APHAG_SOD
HPX_SCRB
CLIPA_FREP
APHAG_HPX
CLIPA_CLIPB
CLIPA_CLIPE
FREP_LRIM
IMDMOD_TPX
NIMROD_SRPN
CLIPD_SCRA
CLIPA_TEP
IMDSIG_JASTMOD
HPX_TPX
AMP_FREP
GALE_ML
GALE_GNBP
SCRA_TOLL
CTL_LRIM
CASP_FREP
SRPN_TEP
LRIM_TEP
GNBP_PGRP
CLIPB_TEP
IMDMOD_SCRB
CLIPA_NIMROD
GPX_ML
GNBP_GPX
GPX_SRPN
SCRB_TPX
GALE_SCRB
IMDSIG_JASTSIG
CLIPE_SRPN
CLIPB_SRPN
JASTMOD_SRRP
CASP_GNBP
TOLLSIG_TPX
CLIPA_SPZ
SPZ_TPX
GPX_SOD
CLIPA_SCRA
NIMROD_SCRA

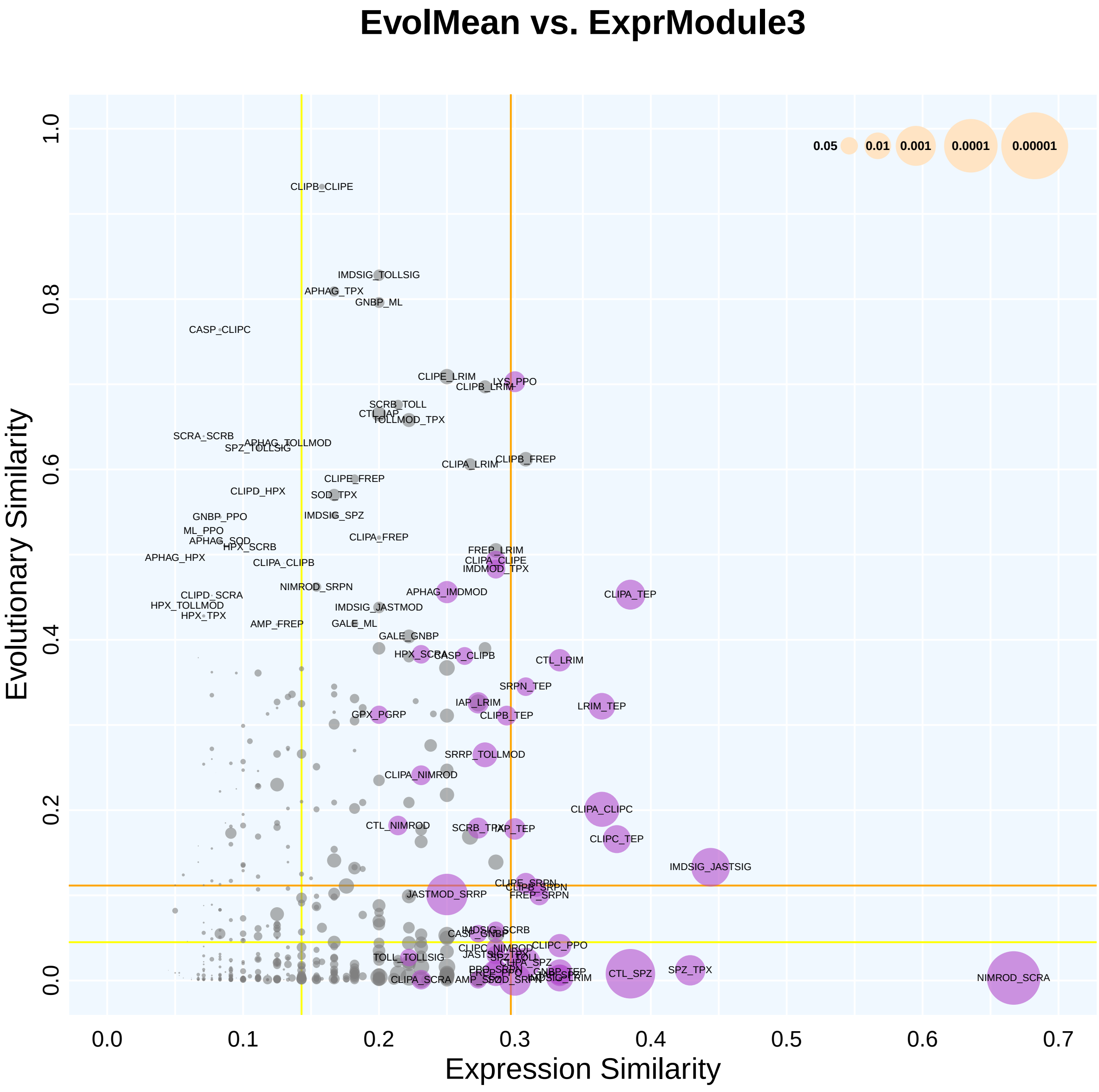

EvolMean vs. ExprModule3
Evolutionary Similarity
Expression Similarity
0.05
0.01
0.001
0.0001
0.00001
CLIPB_CLIPE
IMDSIG_TOLLSIG
APHAG_TPX
GNBP_ML
CASP_CLIPC
CLIPE_LRIM
LYS_PPO
CLIPB_LRIM
SCRB_TOLL
CTL_IAP
TOLLMOD_TPX
SCRA_SCRB
APHAG_TOLLMOD
SPZ_TOLLSIG
CLIPA_LRIM
CLIPB_FREP
CLIPE_FREP
CLIPD_HPX
SOD_TPX
IMDSIG_SPZ
GNBP_PPO
ML_PPO
APHAG_SOD
HPX_SCRB
CLIPA_FREP
APHAG_HPX
CLIPA_CLIPB
FREP_LRIM
CLIPA_CLIPE
IMDMOD_TPX
NIMROD_SRPN
APHAG_IMDMOD
CLIPA_TEP
CLIPD_SCRA
HPX_TOLLMOD
HPX_TPX
IMDSIG_JASTMOD
AMP_FREP
GALE_ML
GALE_GNBP
HPX_SCRA
CASP_CLIPB
CTL_LRIM
SRPN_TEP
IAP_LRIM
LRIM_TEP
GPX_PGRP
CLIPB_TEP
SRRP_TOLLMOD
CLIPA_NIMROD
CLIPA_CLIPC
CTL_NIMROD
SCRB_TPX
IAP_TEP
CLIPC_TEP
IMDSIG_JASTSIG
CLIPE_SRPN
CLIPB_SRPN
JASTMOD_SRRP
FREP_SRPN
IMDSIG_SCRB
CASP_GNBP
CLIPC_PPO
CLIPC_NIMROD
TOLL_TOLLSIG
CLIPA_SPZ
GNBP_TEP
CTL_SPZ
SPZ_TPX
CLIPA_SCRA
AMP_SOD
NIMROD_SCRA

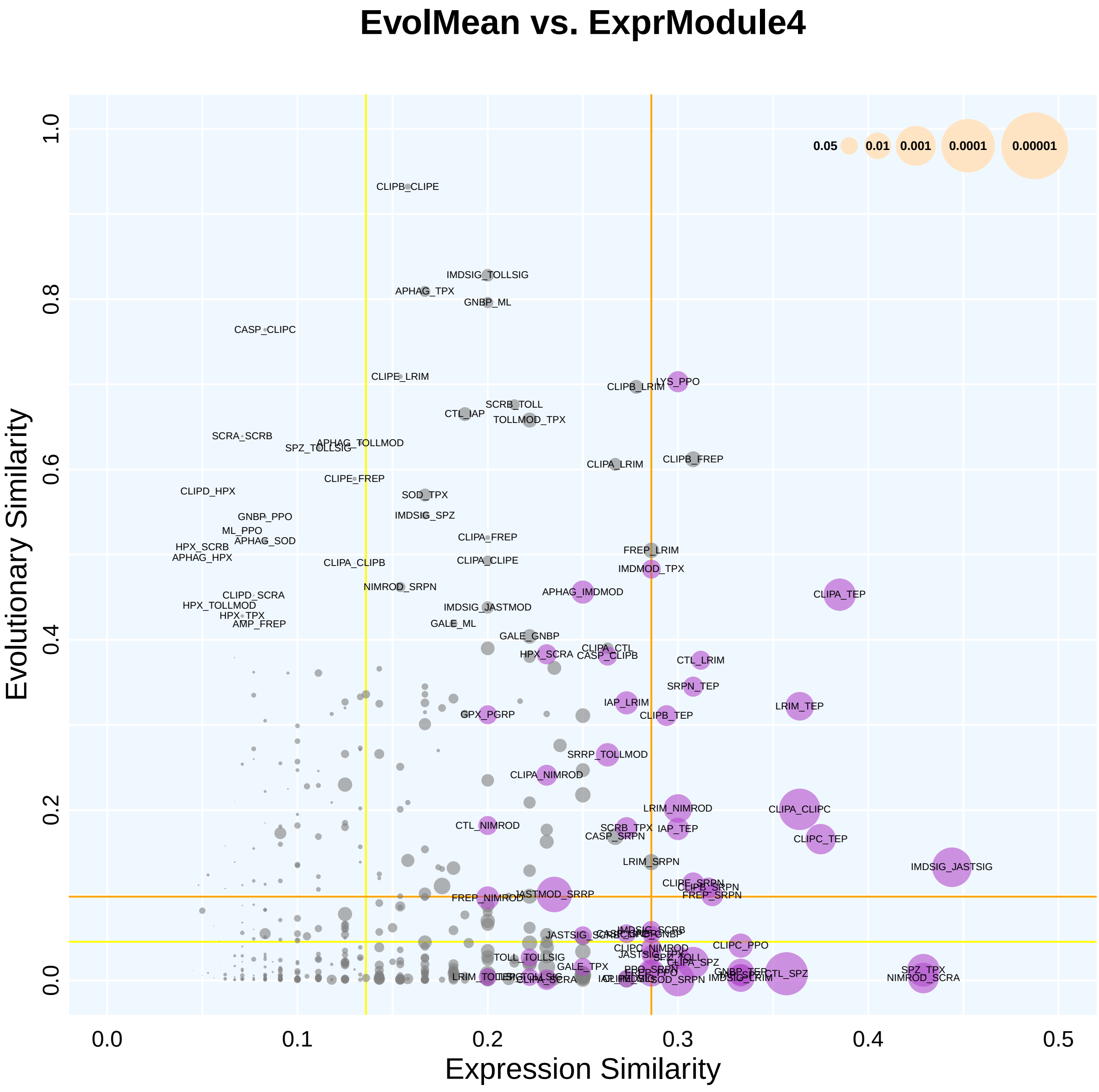
EvolMean vs. ExprModule4
Evolutionary Similarity
Expression Similarity
0.0
0.1
0.2
0.3
0.4
0.5
0.2
0.4
0.6
0.8
1.0
0.05
0.01
0.001
0.0001
0.00001
CLIPB_CLIPE
IMDSIG_TOLLSIG
APHAG_TPX
GNBP_ML
CASP_CLIPC
CLIPE_LRIM
CLIPB_LRIM
LYS_PPO
SCRB_TOLL
CTL_IAP
TOLLMOD_TPX
SCRA_SCRB
APHAG_TOLLMOD
SPZ_TOLLSIG
CLIPA_LRIM
CLIPB_FREP
CLIPE_FREP
CLIPD_HPX
SOD_TPX
GNBP_PPO
IMDSIG_SPZ
ML_PPO
APHAG_SOD
CLIPA_FREP
HPX_SCRB
APHAG_HPX
FREP_LRIM
CLIPA_CLIPB
CLIPA_CLIPE
IMDMOD_TPX
NIMROD_SRPN
APHAG_IMDMOD
CLIPA_TEP
CLIPD_SCRA
HPX_TOLLMOD
IMDSIG_JASTMOD
HPX_TPX
AMP_FREP
GALE_ML
GALE_GNBP
CLIPA_CTL
HPX_SCRA
CASP_CLIPB
CTL_LRIM
SRPN_TEP
IAP_LRIM
LRIM_TEP
GPX_PGRP
CLIPB_TEP
SRRP_TOLLMOD
CLIPA_NIMROD
LRIM_NIMROD
CLIPA_CLIPC
CTL_NIMROD
SCRB_TPX
IAP_TEP
CASP_SRPN
CLIPC_TEP
LRIM_SRPN
IMDSIG_JASTSIG
FREP_NIMROD
JASTMOD_SRRP
FREP_SRPN
JASTSIG_SCRB
CLIPC_PPO
CLIPC_NIMROD
TOLL_TOLLSIG
CLIPA_SPZ
GALE_TPX
CTL_SPZ
SPZ_TPX
NIMROD_SCRA
LRIM_TOLL
CLIPA_SCRA
SOD_SRPN
IMDSIG_LRIM

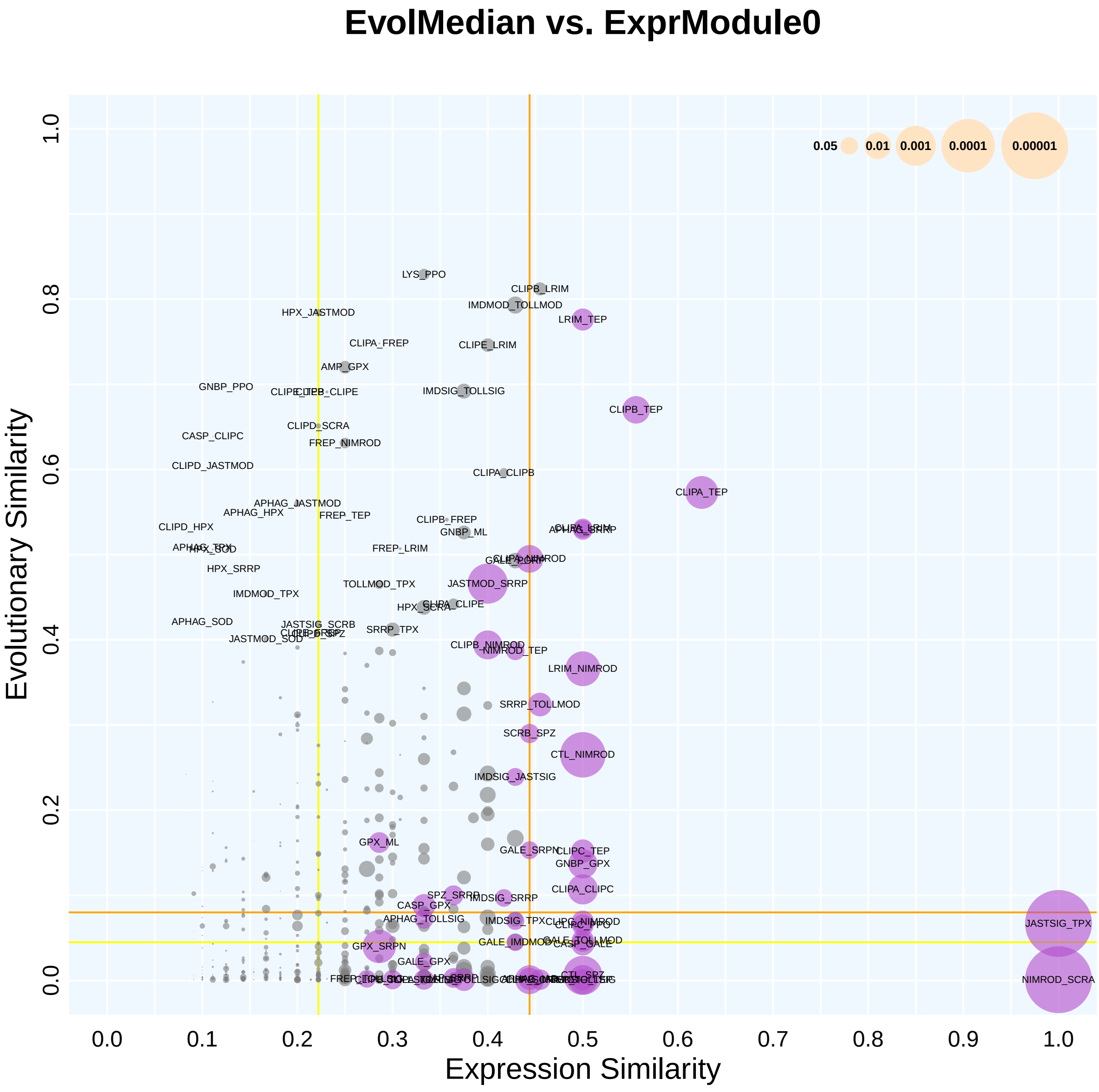
EvolMedian vs. ExprModule0
Evolutionary Similarity
Expression Similarity
0.0
0.2
0.4
0.6
0.8
1.0
0.1
0.3
0.5
0.7
0.9
0.05
0.01
0.001
0.0001
0.00001
LYS_PPO
CLIPB_LRIM
IMDMOD_TOLLMOD
HPX_JASTMOD
LRIM_TEP
CLIPA_FREP
CLIPE_LRIM
AMP_GPX
GNBP_PPO
IMDSIG_TOLLSIG
CLIPB_TEP
CLIPD_SCRA
CASP_CLIPC
FREP_NIMROD
CLIPD_JASTMOD
CLIPA_CLIPB
CLIPA_TEP
APHAG_JASTMOD
APHAG_HPX
FREP_TEP
CLIPD_HPX
CLIPB_FREP
GNBP_ML
FREP_LRIM
HPX_SRRP
TOLLMOD_TPX
JASTMOD_SRRP
IMDMOD_TPX
APHAG_SOD
JASTSIG_SCRB
JASTMOD_SOD
SRRP_TPX
LRIM_NIMROD
SRRP_TOLLMOD
SCRB_SPZ
CTL_NIMROD
IMDSIG_JASTSIG
GPX_ML
GALE_SRPN
CLIPC_TEP
GNBP_GPX
CLIPA_CLIPC
IMDSIG_SRRP
CASP_GPX
APHAG_TOLLSIG
GPX_SRPN
GALE_GPX
JASTSIG_TPX
NIMROD_SCRA

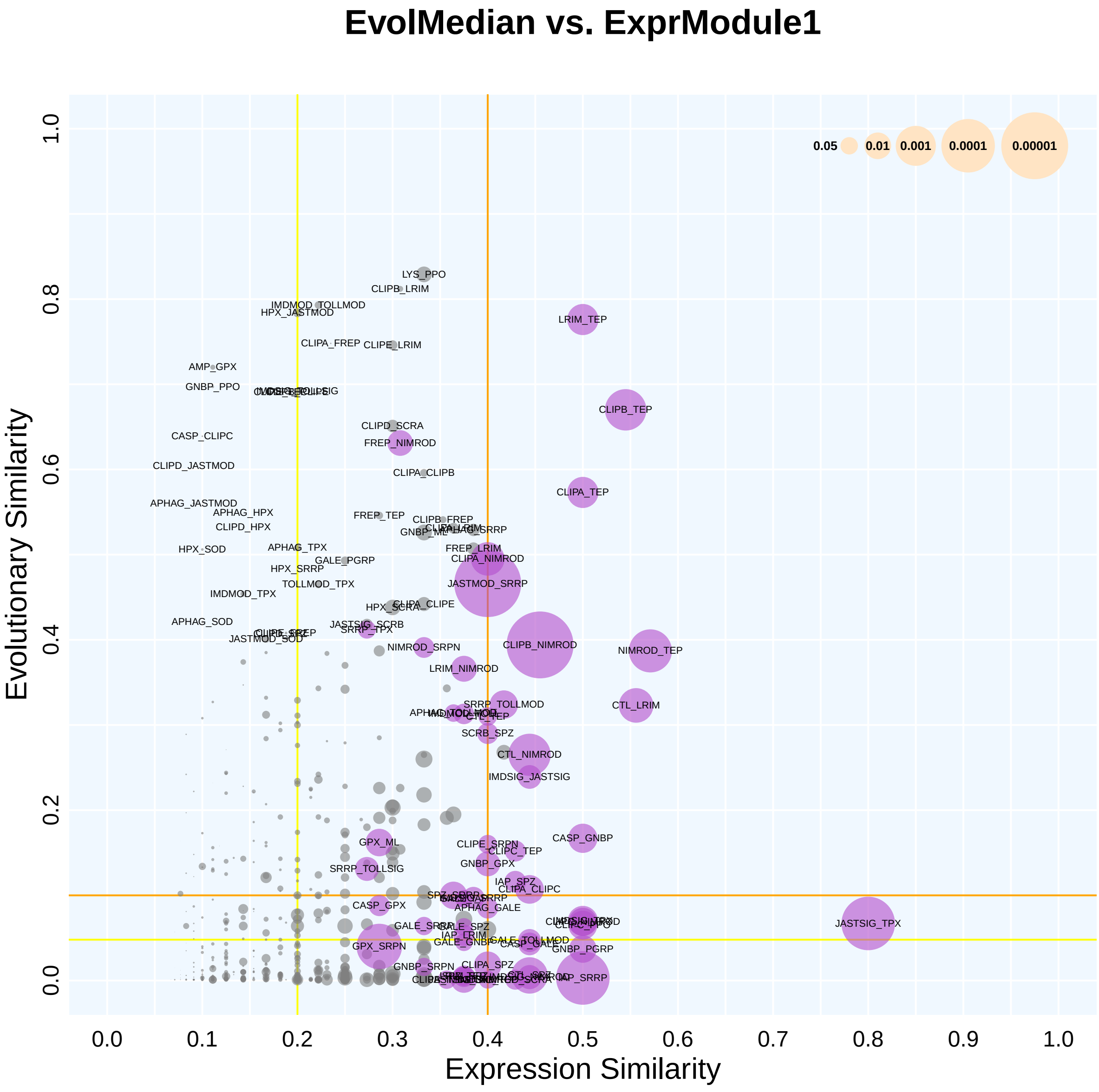

EvolMedian vs. ExprModule1
Evolutionary Similarity
Expression Similarity
0.0
0.2
0.4
0.6
0.8
1.0
0.1
0.3
0.5
0.7
0.9
0.05
0.01
0.001
0.0001
0.00001
LYS_PPO
CLIPB_LRIM
IMDMOD_TOLLMOD
HPX_JASTMOD
LRIM_TEP
CLIPA_FREP
CLIPE_LRIM
AMP_GPX
GNBP_PPO
CLIPB_TEP
CLIPD_SCRA
CASP_CLIPC
FREP_NIMROD
CLIPD_JASTMOD
CLIPA_CLIPB
CLIPA_TEP
APHAG_JASTMOD
APHAG_HPX
FREP_TEP
CLIPB_FREP
CLIPD_HPX
GNBP_ML
HPX_SOD
APHAG_TPX
FREP_LRIM
CLIPA_NIMROD
GALE_PGRP
HPX_SRRP
TOLLMOD_TPX
JASTMOD_SRRP
IMDMOD_TPX
HPX_SCRA
CLIPA_CLIPE
APHAG_SOD
JASTSIG_SCRB
SRRP_TPX
JASTMOD_SOD
CLIPB_NIMROD
NIMROD_TEP
NIMROD_SRPN
LRIM_NIMROD
SRRP_TOLLMOD
CTL_LRIM
CTL_TEP
SCRB_SPZ
CTL_NIMROD
IMDSIG_JASTSIG
CASP_GNBP
GPX_ML
CLIPE_SRPN
CLIPC_TEP
SRRP_TOLLSIG
GNBP_GPX
IAP_SPZ
CLIPA_CLIPC
CASP_GPX
APHAG_GALE
JASTSIG_TPX
GALE_SRPN
GALE_SPZ
IAP_LRIM
CLIPC_PPO
GPX_SRPN
GALE_GNBP
GALE_TOLLMOD
CASP_GALE
GNBP_PGRP
CLIPA_SPZ
GNBP_SRPN
IAP_SRRP

EvolMedian vs. ExprModule2

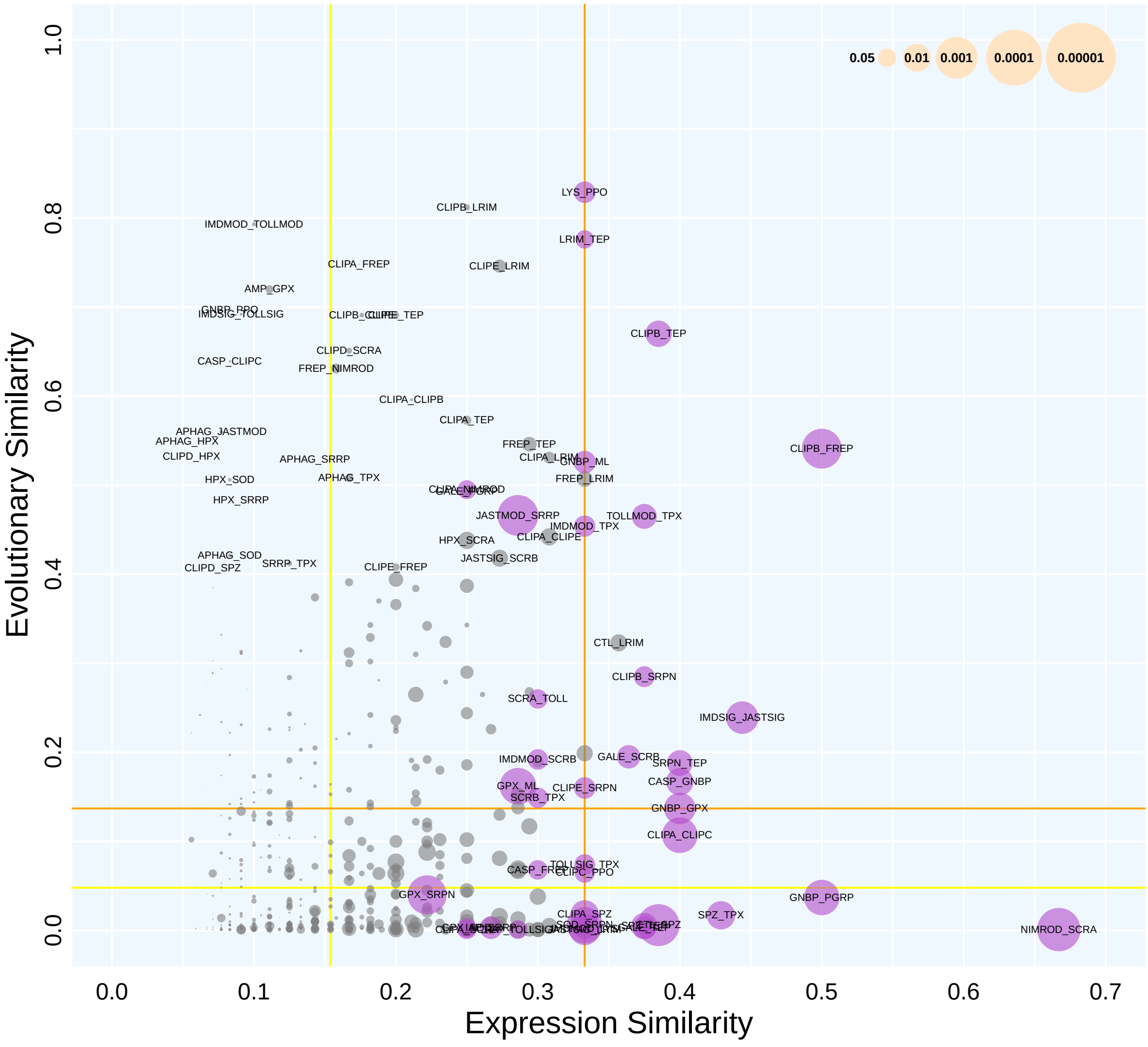

EvolMedian vs. ExprModule3

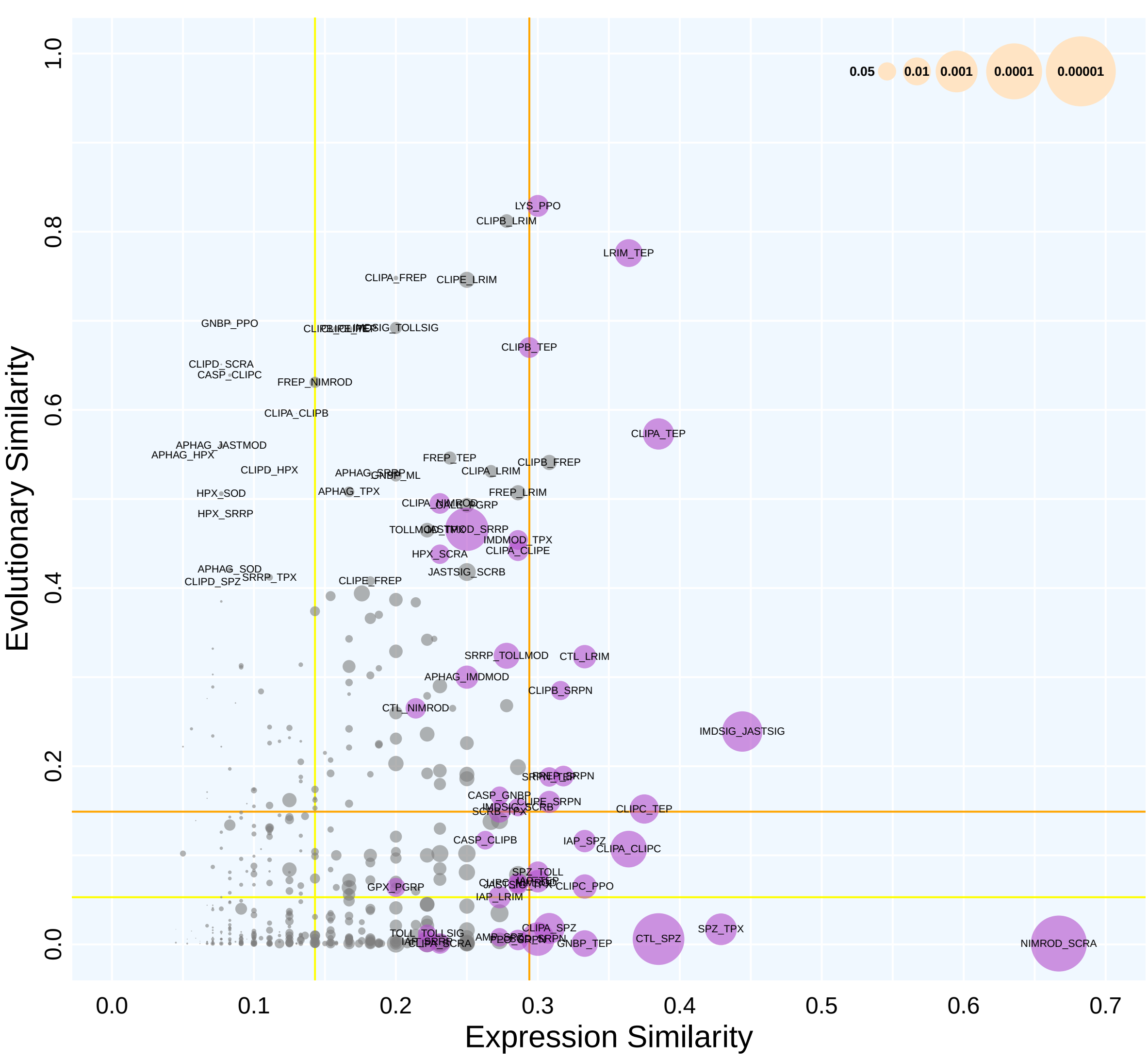

EvolMedian vs. ExprModule4

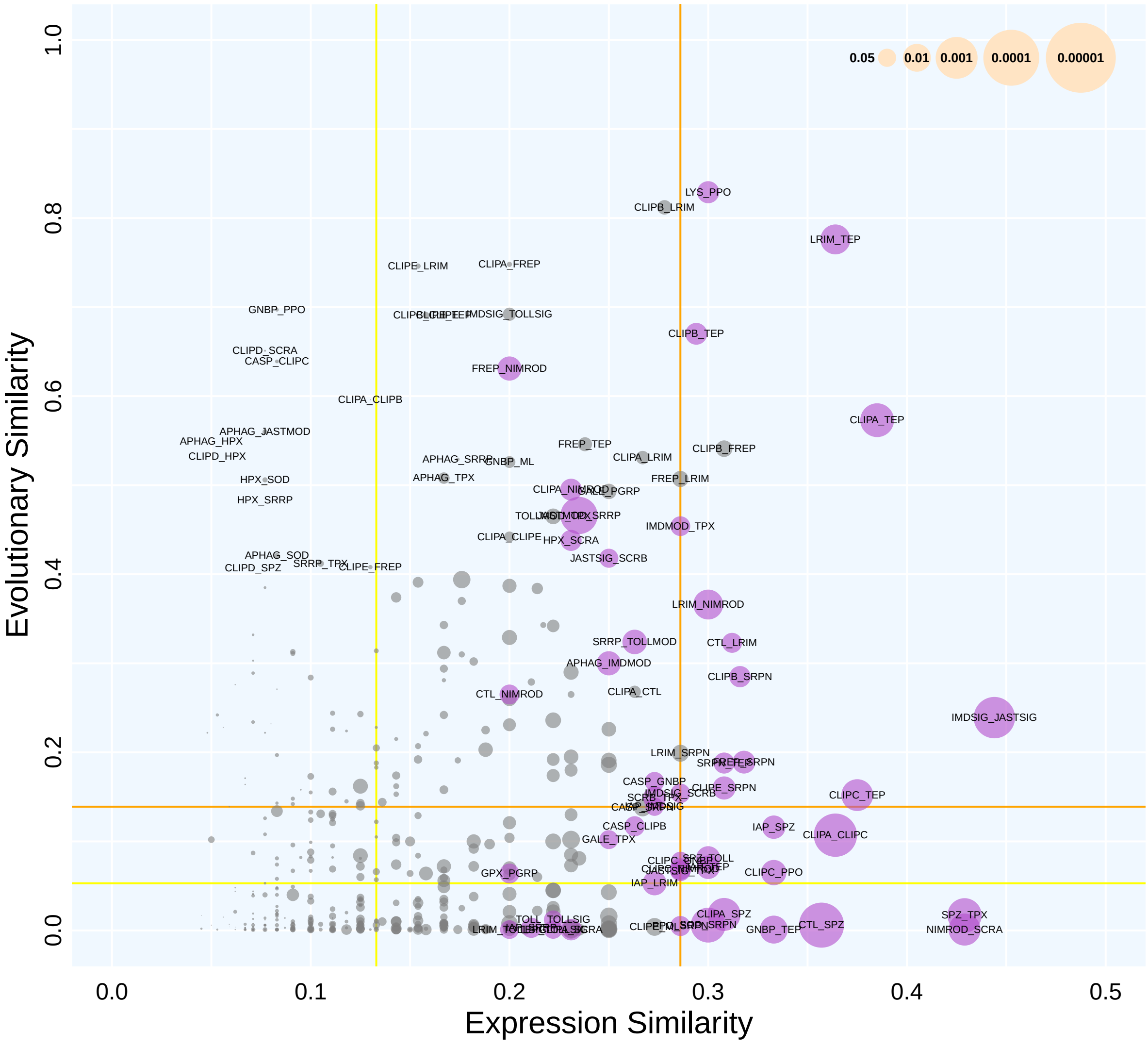

Supplement: msab352_Supplementary_Data [file msab352_supplementary_data.zip › Additional_File_4_evolutionary-expression-similarities-all.pdf]
